# Stereoselective Pudovik reaction of aldehydes, aldimines, and nitroalkenes with CAMDOL-derived *H*-phosphonate

**DOI:** 10.1038/s42004-025-01735-4

**Published:** 2025-11-14

**Authors:** Ning Li, Qian Wu, Yu Huang, Li Pan, Junchen Li, Enxue Shi, Junhua Xiao

**Affiliations:** State Key Laboratory of NBC Protection for Civilian, Beijing, PR China

**Keywords:** Stereochemistry, Synthetic chemistry methodology

## Abstract

Optically pure phosphonates featuring an α-functional motif serve as core structures of versatile pharmaceuticals. Pudovik reaction employing P-stereogenic *H*-phosphonates derived from recyclable chiral auxiliary (CA) is one of the most practical and efficient asymmetric synthetic strategies. Although several centre and axial CAs have been investigated in this field, the development of novel chiral *H*-phosphonates has remained largely unexplored over the past 25 years. Here, we developed a kind of *H*-phosphonate (CAMDOL-PHO) derived from camphor-derived 2,3-diol (CAMDOL). Stereoselective Pudovik reactions of CAMDOL-PHO with diverse aldehydes, aldimines, and nitroalkenes were well developed, affording a library of molecules of α-hydroxyl, α-amino, and β-nitro phosphonates respectively with high *dr* values (up to 99:1) and yields (up to 97%) as well as broad scopes. Comparative DFT and experimental studies demonstrated that CAMDOL-PHO possessed significant superiority to install the P-adjacent C-stereogenic centre, mainly benefiting from its unique diphenyl-substituted camphor skeleton with an angular methyl group.

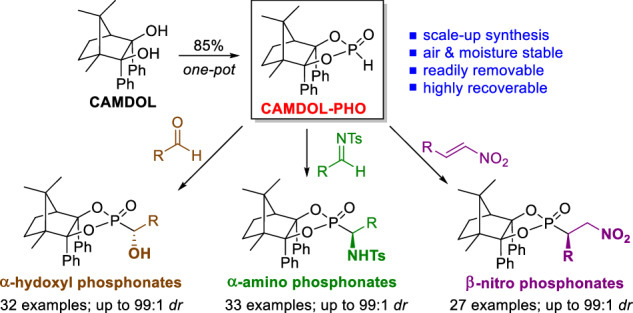

## Abstract (web summary)

Optically pure phosphonates are crucial in pharmaceuticals, yet the development of chiral H-phosphonates has been stagnant for decades. Here, the authors report an H-phosphonate derived from camphor-derived 2,3-diols (CAMDOL), that enables efficient stereoselective Pudovik reactions and yields diverse bioactive molecules, with significant implications for asymmetric synthesis.

## Introduction

**Fig. 1:**
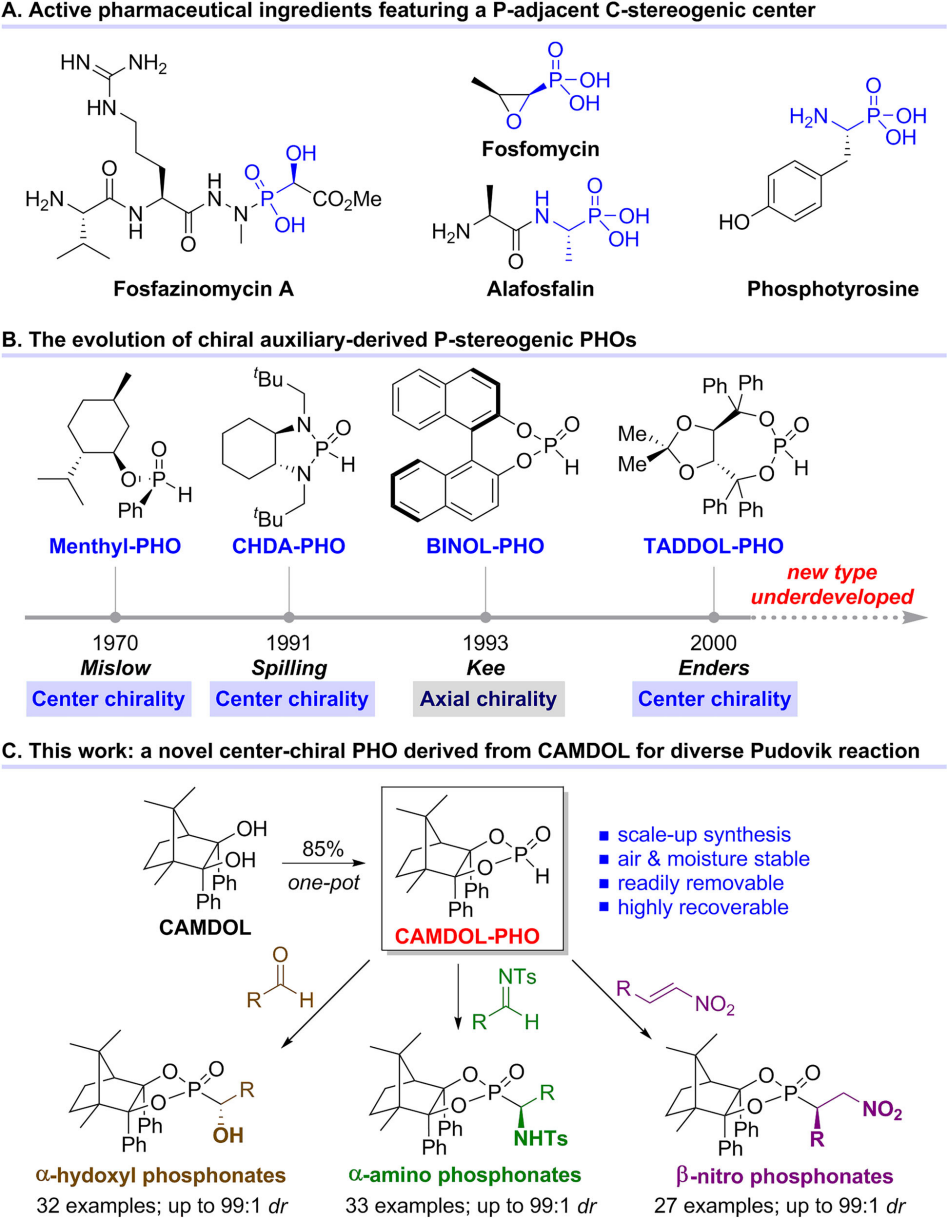
Background and concept. **A** Selected pharmaceutical compounds containing P-adjacent C-stereogenic center. **B** Previous reports for the development of CA-derived P-chiral *H*-phosphonates. **C** Our work about the design and application of center-chiral CAMDOL-derived *H*-phosphonate (abbrev. CAMDOL-PHO) for stereoselective Pudovik reaction.

Organophosphonates are important structural units frequently found in biologically active compounds, including natural products, active pharmaceutical ingredients, and pesticides^1–10^. In particular, optically pure α-hydroxyl, α-amino, and β-amino phosphonic acids, and the related derivatives serve as core motifs of versatile pharmaceuticals^11–17^, such as Fosfazinomycin A^18,19^, Fosfomycin^20,21^, Alafosfalin^22,23^, and Phosphotyrosine^24,25^ (Fig. 1A). Accordingly, numerous synthetic approaches have been developed to meet the increasing need for those enantiopure organophosphorus compounds^26–30^. Pudovik reaction, known as the addition of dialkyl phosphites or the relevant P(O)H compounds to unsaturated chemical bonds (C=C, C≡C, C=O, C=N, etc.) to generate P–C functionalized phosphonates, is one of the most powerful and direct methods for the induction of phosphorus groups^31–35^. In this context, three types of asymmetric hydrophosphonylation methodologies were mainly put forward: chiral Lewis acid complex catalysis, organocatalysis, and chiral auxiliary (CA) induction^36–39^. More or less, the practicability of the catalytic asymmetric methods is generally limited by the use of special reagents and relatively narrow substrate scopes (always to be prepared with much effort)^40^. In comparison, the recyclable CA induced stereoselective Pudovik reaction employing P-stereogenic *H*-phosphonates has been substantiated by significant progress in the evolution of different chiral skeletons^41^, arising from the pioneering studies of Mislow’s menthyl-PHO^40,42^, Spilling’s CHDA-PHO^43^, Kee’s BINOL-PHO^44^, and Enders’s TADDOL-PHO^45^ (Fig. 1B). These P-chiral CA-PHOs could readily react with versatile partners to provide structurally diverse P-adjacent C-stereogenic molecules. What’s more, diastereomeric pairs in the products from CA-PHO could be unambiguously distinguished by NMR, thereby enabling the easy real-time monitoring of the stereochemical process of these reactions by ^31^P/^1^H NMR. More importantly, the CA-induction methods can generate the P–C bond and the desired C–X (X=C, O, N, etc.) motifs concurrently and generally avoid the inconvenient pre-synthesis of the relevant P-substrates. However, to some extent, further applications of these CA-PHO are much limited as a result of the relatively harsh preparation conditions, low diastereoselectivities, or confined scopes.

Therefore, a critical objective for advancing stereoselective Pudovik reactions lies in developing novel induction strategies to enable chirality installation at carbon centers, particularly through rationally designed chiral auxiliaries with enhanced efficiency. However, despite the utility of the four classic CA-PHOs, almost no newly CA-PHOs have been reported over the past 25 years.

Very recently, we established a novel pattern of CA platform—camphor-derived 2,3-diols (CAMDOL)—which demonstrates exceptional utility in diastereoselective reactions, including synthesis of diverse chiral P(III)/P(V)-compounds^46^ and asymmetric α-functionalization of phosphonates^47^. In this study, we further advance CAMDOL to synthesize a centrally chiral P-chiral *H*-phosphonate through a streamlined one-pot protocol. This strategy enables subsequent asymmetric Pudovik additions with well-characterized aldehydes, aldimines, and nitroalkenes, thereby achieving diastereoselective construction of high-value α-hydroxyl-, α-amino-, and β-amino-phosphonates (Fig. 1C).

## Results

### Preparation of P-chiral CAMDOL-PHO

**Fig. 2:**
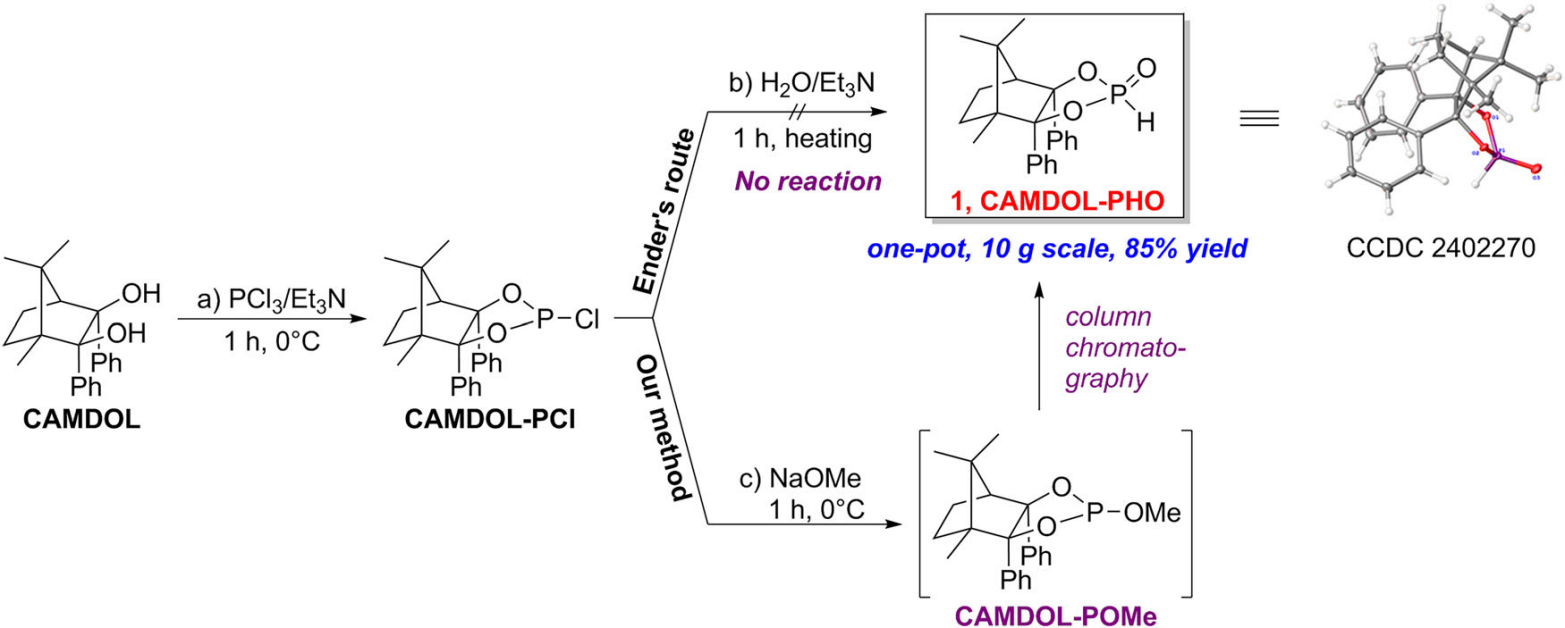
One-pot scale-up synthesis of CAMDOL-PHO 1. **a** CAMDOL (30 mmol), PCl_3_ (1.05 equiv.), Et_3_N (3 equiv.), THF, 0 °C, 1 h, >99% conversion; **b** H_2_O (10 equiv.), Et_3_N (10 equiv.), THF, 0 °C, 1 h; **c** NaOMe (2 equiv.), 0 °C, 1 h, >99% conversion; then quenched with saturated NH_4_Cl aqueous, and purified by flash column chromatography to give **1** in 85% isolated yield and >99:1 *dr*.

Inspired by Ender’s creative work about TADDOL-PHO synthesis^45^, we first attempted to prepare the enantiomeric CAMDOL-PHO **1** in the same manner (Fig. 2). As expected, CAMDOL could be efficiently converted to CAMDOL-PCl in nearly quantitative yield by treatment with PCl_3_/Et_3_N. Moreover, CAMDOL-PCl was comparatively stable for several months at room temperature, and could even be easily purified by silica gel column chromatography. However, the subsequent selective hydrolysis of exocyclic P–Cl bond of CAMDOL-PCl proved to be a formidable challenge. Even under severe conditions including usage of high temperature and concentrated hydrochloric acid, no P–Cl bond cleavage reaction was observed, which is much different from the behaviours of reported TADDOL-PCl or BINOL-PCl^48^. According to our recent work about SiO_2_-promoted efficient hydrolysis of phosphonites^49^, we rationalized that CAMDOL-POR, as an alternative substrate, might obey the P–OR detaching rule to give the desired CAMDOL-PHO. To our delight, CAMDOL-POMe, derived from CAMDOL-PCl and NaOMe did transform into CAMDOL-PHO completely when managing it by routine column isolation directly. Moreover, this two-step synthesis could be conducted readily by a one-pot process without isolation of the intermediates CAMDOL-PCl and CAMDOL-POMe, affording the CAMDOL-PHO **1** in 85% isolated yield at a decagram scale. The absolute configuration of the P-chirality center for CAMDOL-PHO **1** was assigned as *R*-configuration based on its X-ray crystallography.

### Pudovik reaction of aldehydes with CAMDOL-PHO

At the outset, to evaluate the validity of this center-chiral CAMDOL-PHO **1** in stereoselective Pudovik addition with aldehydes, benzaldehyde (PhCHO) **2a** was selected as the model substrate to optimize the reaction conditions (Table 1). Initially, no desired α-hydroxyphsphonate **3a** was observed when employing either inorganic base (K_2_CO_3_) or organic base (Et_3_N, pyridine, DBU, or BTMG) at 0 °C (entries 1–5). Further evaluation of other stronger bases (LiHMDS, NaHMDS, nBuLi, and LDA) at a relatively lower temperature (−40 °C) revealed that LDA exhibited superior conversion and selectivity (entries 6–9). Lowering the temperature from −40 °C to −78 °C with LDA as the base, the diastereomeric ratio of 3a increased from 93:7 to 97:3 without any loss in yield, indicating that a lower temperature has a beneficial impact on diastereoselective induction for this reaction (entries 9–11). Other types of solvents, such as PhMe, Et_2_O, DCM, MeOH, and EtOAc, all resulted in a remarkable reduction in both yields and diastereoselectivities, especially for the protonic solvent (MeOH) (entries 12–16). By augmenting the dosage of LDA from 1 to 1.2 equivalent, the best yield of 85% and *dr* value of 99:1 of 3a were achieved (entriy 17). Nevertheless, an identical yield but a lower *dr* was obtained when 1.5 equivalent of LDA was used (entry 18).

**Table 1:** Optimization of aldehyde-based Pudovik reaction^a^


Entry	Base	Solvent	Temp (°C)	Yield^b^ (%)	*dr*^c^
1	K_2_CO_3_	THF	0	ND	-
2	Et_3_N	THF	0	ND	-
3	pyridine	THF	0	ND	-
4	DBU	THF	0	ND	-
5	BTMG	THF	0	ND	-
6	LiHMDS	THF	−40	50	85:15
7	NaHMDS	THF	−40	65	83:17
8	^*n*^BuLi	THF	−40	30	80:20
9	LDA	THF	−40	75	93:7
10	LDA	THF	−50	75	95:5
11	LDA	THF	−78	75	97:3
12	LDA	PhMe	−78	60	70:30
13	LDA	Et_2_O	−78	45	85:15
14	LDA	DCM	−78	70	75:25
15	LDA	MeOH	−78	20	-
16	LDA	EtOAc	−78	70	70:30
**17**^**d**^	**LDA**	**THF**	**−78**	**85**	**99:1**
18^e^	LDA	THF	−78	85	97:3

The bolded data represent the optimal reaction conditions.

*ND* not detected.

^a^Reaction conditions: **1** (0.4 mmol, 1 equiv.), **2a** (0.48 mmol, 1.2 equiv.), base (0.4 mmol, 1 equiv.), solvent (8 mL), N_2_, 24 h.

^b^Isolated yields.

^c^Determined by ^31^P NMR.

^d^0.48 mmol (1.2 equiv.) of LDA was employed.

^e^0.6 mmol (1.5 equiv.) of LDA was employed.

**Fig. 3:**
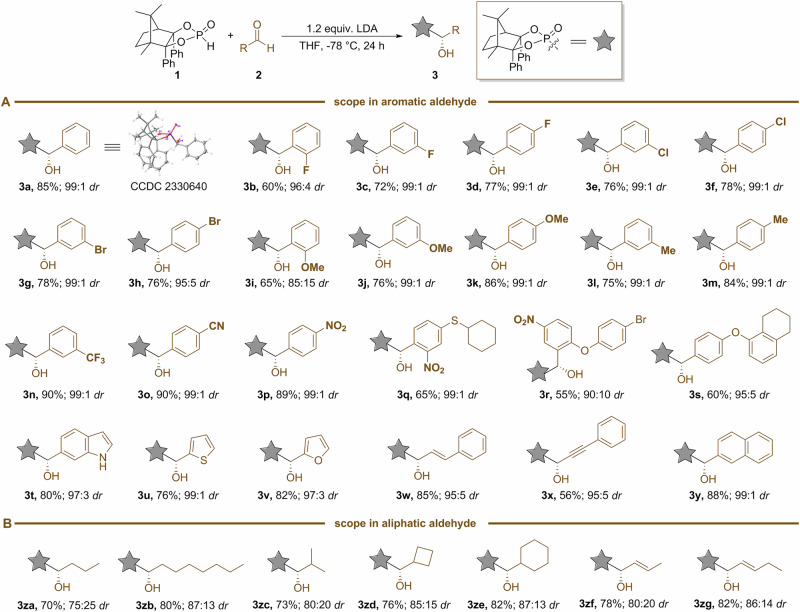
Scope of α-hydroxyl phosphonates. **A** Scope in aromatic aldehyde. **B** Scope in aliphatic aldehyde.

With the optimized conditions in hand, we then performed a series of experiments to define the scope of functional groups and electronic constraints that are readily tolerated on the aldehyde component. As shown in Fig. 3, numerous aromatic aldehydes can be readily employed with CAMDOL-PHO to produce the target chiral α-hydroxyphosphonates without any detectable loss in diastereopurity (**3a**–**3y**). An *ortho*-fluoro substituent of benzaldehyde, as well as an *ortho*-methoxyl group, brought about a little decrease in the yield and *dr* (**3b** and **3i**). In comparison, those *meta*- or *para*-substituents (such as F, Cl, Br, OMe, Me, CF_3_, CN, and NO_2_) in benzaldehyde’s ring can be incorporated without significant impact on yield or selectivity, and all of these substrates could give nearly stereospecific products (**3c**–**3h,** **3j**–**3p**) with 99:1 *dr* values. Surprisingly, nitrobenzaldehyde featuring other types of groups provided different results that *ortho*-nitrobenzaldehyde featuring *para*-sulfide substituent gave **3q** in 65% yield and 99:1 *dr*, whereas *meta*-nitrobenzaldehyde featuring *para*-ether group gave **3r** in only 55% yield and 90:10 *dr*. It should be mentioned that aromatic aldehydes bearing a reactive Br or Cl group do not undergo lithiation at the halogenated site or interfere with the reaction efficiency, allowing all the desired addition products in good yields and up to 99:1 *dr* values (**3e**–**3h**). Heteroaromatic aldehydes with an indole, thiophene, or furan ring, all occurred smoothly to deliver α-hydroxyphosphonates with good yields and high levels of enantiocontrol (**3t**–**3v**). The conjugated aromatic vinyl or acetylenyl aldehyde reacted selectively at the carbonyl site to generate the corresponding α-hydroxyphosphonates with good yields and excellent stereoseletivities (**3w**–**3x**). The fused aromatic aldehyde, 2-naphthaldehyde, allowed the formation of **3y** in 88% yield and 99:1 *dr*. As for aliphatic (long chain alkyl, α-branched alkyl, cycloalkyl, and conjugated vinyl) aldehyde, generally a slight decrease in diastereopurity was observed due to their well-recognized less steric hindrance (**3za–3zc,** **3zd–3zg**). The absolute configuration of the new chiral center for α-hydroxyphosphonate **3a** was assigned as *R*-configuration based on its X-ray crystallography.

**Fig. 4:**
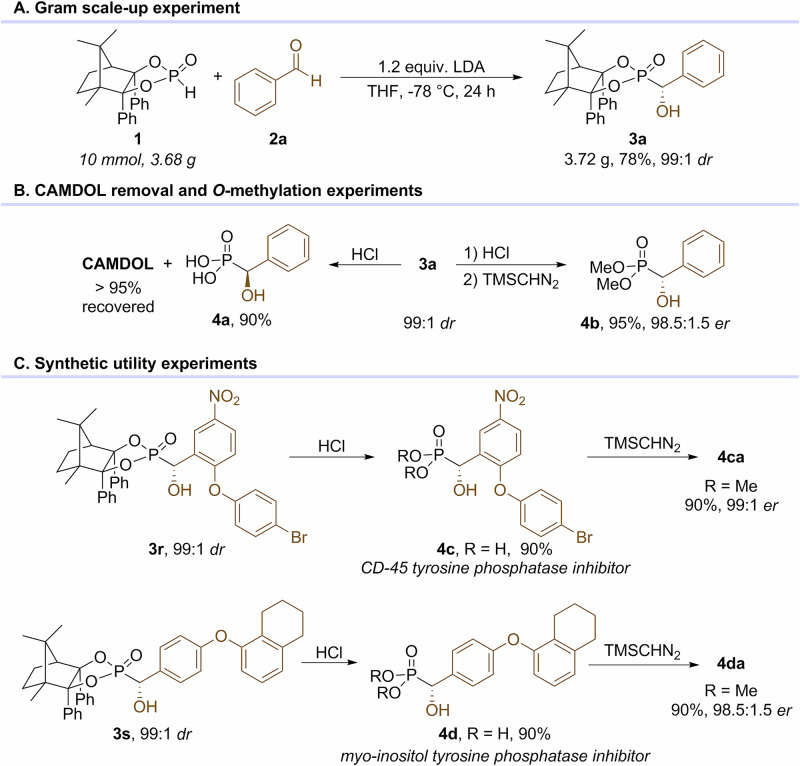
Synthetic utility experiments of aldehyde-based Pudovik reaction. **A** Gram scale-up experiment. **B** CAMDOL removal and *O*-methylation experiments. **C** Synthetic utility experiments.

To demonstrate the preparative utility of our reaction, a 10 mmol scale-up synthesis was performed, and **3a** was afforded in 78% yield and 99:1 *dr* (Fig. 4A). One of the most advantages of CA induction methods is the recoverability, so did the CAMDOL. Hydrolysis of **3a** with conc. HCl afforded the α-hydroxyphosphonic acid **4a** in 90% yield. In the meanwhile, more than 95% CAMDOL was recovered in this process. Methylation of crude **4a** with diazomethane gave the dimethyl α-hydroxyphosphonate **4b** in 98.5:1.5 *dr* with an almost quantitative conversion (Fig. 4B). The acidic hydrolysis of **3r** and **3 s** offered the CD-45 tyrosin phosphatase inhibitor **4c**^50^ and *myo*-inositol tyrosine phosphatase inhibitor **4d**^51,52^ both in 90% yields without loss of the *C*-chirality, as further confirmed by diazomethylation with TMSCHN_2_ (Fig. 4C).

### Pudovik reaction of aldimines with CAMDOL-PHO

Based on the above investigations, our diastereoselective Pudovik reaction of aldimines was first evaluated by using *N*-benzylidene-*p*-toluenesulfonamide **5a** with **1** and a series of bases (Table 2). A survey of the base in THF revealed that BTMG provided the highest yield and level of enantiocontrol for α-amino phosphonate **6a** at −40 °C (entries 1–7). As the temperature of the reaction decreased, a dramatic increase in disastereoselectivity and reaction efficiency was achieved using BTMG as the base (entries 4, 8–11). Solvent screening experiments displayed that THF was the optimal solvent, indicating that reaction media indeed influence this stereoselective process remarkably (entries 11–16). By augmenting the dosage of BTMG from 1 to 1.2 equivalent, a yield of 95% and a *dr* of 99:1 of **6a** were obtained for this transformation (entry 17), whereas a slight decrease in yield and diastereoselectivity was monitored when 1.5 equivalent LDA was used (entry 18).

**Table 2:** Optimization of aldimine-based Pudovik reaction^a^


Entry	Base	Solvent	Temp (°C)	Yield^b^ (%)	*dr*^c^
1	K_2_CO_3_	THF	0	ND	-
2	Et_3_N	THF	0	ND	-
3	DBU	THF	0	40	65:35
4	BTMG	THF	0	65	75:25
5	LiHMDS	THF	−40	35	60:40
6	^*n*^BuLi	THF	−40	30	57:43
7	LDA	THF	−40	25	55:45
8	BTMG	THF	−40	80	90:10
9	BTMG	THF	−50	85	93:7
10	BTMG	THF	−60	88	93:7
11	BTMG	THF	−78	90	95:5
12	BTMG	PhMe	−78	78	85:15
13	BTMG	Et_2_O	−78	45	85:15
14	BTMG	DCM	−78	88	75:25
15	BTMG	MTBE	−78	76	70:30
16	BTMG	EtOAc	−78	70	73:27
**17**^**d**^	**BTMG**	**THF**	−**78**	**95**	**99:1**
18^e^	BTMG	THF	−78	85	95:5

The bolded data represent the optimal reaction conditions.

*ND* not detected.

^a^Reaction conditions: **1** (0.4 mmol), **5a** (0.48 mmol), base (0.4 mmol), solvent (8 mL), N_2_, 24 h.

^b^Isolated yield.

^c^Determined by ^31^P NMR.

^d^0.48 mmol of BTMG was employed.

^e^0.6 mmol of BTMG was employed.

**Fig. 5:**
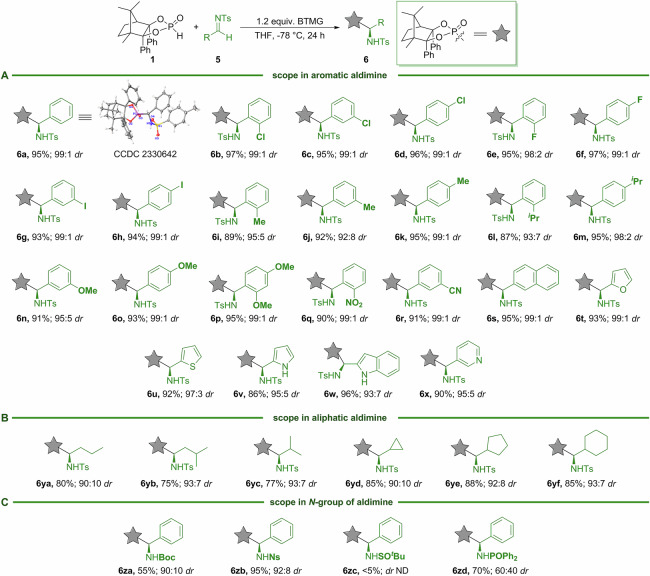
Scope of α-amino phosphonates. **A** Scope in aromatic aldimine. **B** Scope in aliphatic aldimine. **C** Scope in *N*-group of aldimine.

Having identified optimal conditions of this diastereoselective Pudovik reaction for the synthesis of α-aminophosphonates, we aimed to define the scope of the aldimine precursors. As revealed in Fig. 5, a series of differently substituted aromatic and aliphatic aldimines were well tolerated with CAMDOL-PHO **1**. Surprisingly, aromatic aldimines bearing a variety of electronic and steric groups, such as halo, alkyl, alkoxyl, and nitro, can be incorporated without significant impact on yield or selectivity (**6a**–**6r**). The fused aromatic aldimine, for example, 2-naphthaldimine, enabled the synthesis of **6 s** in 95% yield and 99:1 *dr*. Heteroaromatic aldimines with a furan, thiophene, pyrrole, or indole ring, all occurred successfully to provide α-aminophosphonates with excellent yields and high levels of enantiocontrol (**6t**–**6w**). Notably, a strong electron-deficient pyridine ring on the aldimine component is readily accommodated to furnish **6x** in a yield of 90% and a *dr* of 95:5. Differing from the aforementioned aldehyde-based reaction, aliphatic substituents were not detrimental to this process with good diastereoselectivites (up to 93:7 *dr*), illustrating the high-value of CAMDOL-PHO-induced diastereocontrol for Pudovik reaction. Specifically, linear, branched, and cyclic aliphatic carbon skeletons were all well tolerated (**6ya**–**6yf**). In comparison, several *N*-protecting groups were explored and the results showed that the Ns group (**6zb**) had a comparable effect on this reaction, whereas other groups (Boc, ^*t*^BuSO, and PhPO) were all not beneficial for the conversion (**6za,** **6zc**–**6zd**). The absolute configuration of the new chiral center for α-aminophosphonate of **6a** was assigned as *S*-configuration by X-ray crystallography.

**Fig. 6:**
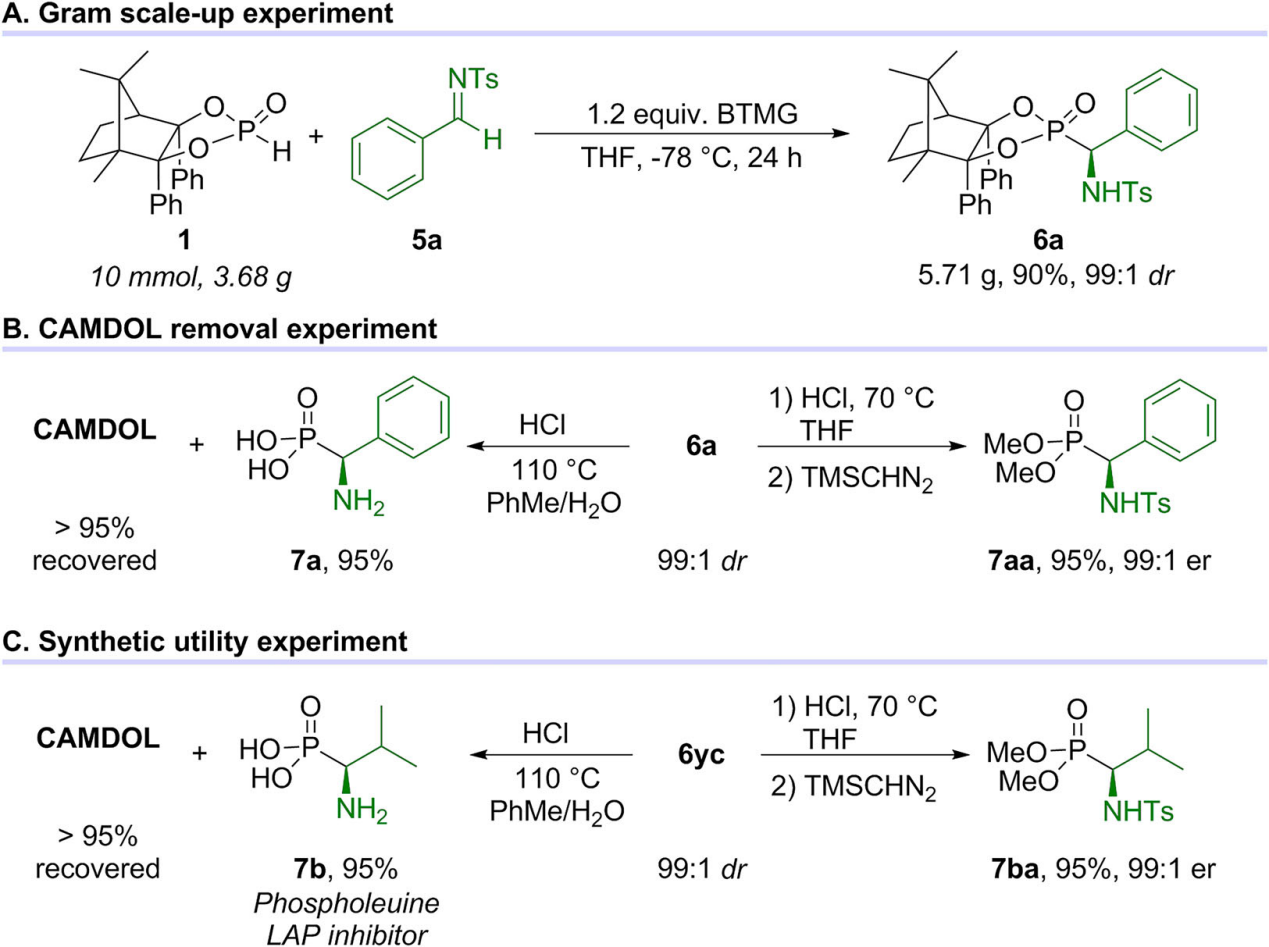
Synthetic utility experiments of aldimine-based Pudovik reaction. **A** Gram scale-up experiment. **B** CAMDOL removal experiment. **C** Synthetic utility experiment.

In order to examine the preparative utility of aldimine-based Pudovik reaction, the addition of CAMDOL-PHO **1** to **5a** was performed on a 10 mmol scale with BTMG as the base to afford **6a** in 90% yield and 99:1 *dr* (Fig. 6A). Enantiopure α-amino phenylphosphonic acid **7a** was obtained in 95% yield from **6a** using aqueous HCl in toluene at 110 °C, along with the recovery of CAMDOL in high yield (>95%). The ‌CAMDOL skeleton of **6a** could be selectively removed with HCl at a lower temperature (70 °C), followed by diazomethylation with TMSCHN_2_ to give **7aa** without loss of the C-chirality (Fig. 6B). Hydrolysis of another α-amino phosphonate **6yc** afforded the bioactive phospholeuine **7b**, a LAP inhibitor^53,54^, whose *er* was determined via a manipulation similar to **7aa**, yielding **7ba** with an *er* of 99:1 (Fig. 6C).

### Pudovik reaction of nitroalkenes with CAMDOL-PHO

Investigations towards the development of nitroalkene-based Pudovik reaction began by reacting CAMDOL-PHO with trans-β-nitrostyrene **8a** under the above similar conditions (Table 3). We were pleased to find that K_2_CO_3_ promoted the reaction in a yield of 60% and a dr of 55:45, recommending the feasibility of the asymmetric synthesis of β-nitrophosphonates **9a** (entry 1). Then, base screening experiments disclosed that NaHMDS could provide the high yield and level of enantiocontrol for **9a** in yield of 75% and *dr* of 83:17 (entries 1–9). Decreasing the reaction temperature resulted in an increase of both conversion and efficiency (−78 °C, 90% yield, 93:7 *dr*; entries 10–11). The impact of reaction media on diastereoselectivity was probed with several aprotic solvents, and MeTHF was proved as the optimal solvent with a superior diastereomeric ratio of 95:5 (entries 11–16). Defining examples of the conversion were found that the optimization of the dosage of NaHMDS of 0.8 equivalent afforded a yield of 95% and a *dr* of 95:5, whereas 1.1 equivalent led to a slight decrease in both yield and diastereoselectivity of **9a** (entries 17–18).

**Table 3:** Optimization of nitroalkene-based Pudovik reaction^a^


Entry	Base	Solvent	Temp (°C)	Yield^b^ (%)	*dr*^c^
1	K_2_CO_3_	THF	0	60	55:45
2	Et_3_N	THF	0	55	67:43
3	DBU	THF	0	50	65:35
4	BTMG	THF	0	45	75:25
5	LiHMDS	THF	−40	45	70:30
6	^*n*^BuLi	THF	−40	30	57:43
7	LDA	THF	−40	45	55:45
8	NaHMDS	THF	−40	75	83:17
9	KHMDS	THF	−40	50	60:40
10	NaHMDS	THF	−60	88	90:10
11	NaHMDS	THF	−78	90	93:7
12	NaHMDS	PhMe	−78	82	85:15
13	NaHMDS	Et_2_O	−78	56	80:20
14	NaHMDS	MeTHF	−78	85	95:5
15	NaHMDS	MTBE	−78	70	70:30
16	NaHMDS	EtOAc	−78	72	73:27
**17**^**d**^	**NaHMDS**	**MeTHF**	**−78**	**95**	**95:5**
18^e^	NaHMDS	MeTHF	−78	90	93:7

The bolded data represent the optimal reaction conditions.

*ND* not detected.

^a^Reaction conditions: **1** (0.2 mmol), **8a** (0.24 mmol), base (0.2 mmol), solvent (4 mL), N_2_, 24 h.

^b^Isolated yield.

^c^Determined by ^31^P NMR.

^d^0.16 mmol (0.8 equiv.) of NaHMDS was employed.

^e^0.22 mmol (1.1 equiv.) of NaHMDS was employed.

**Fig. 7:**
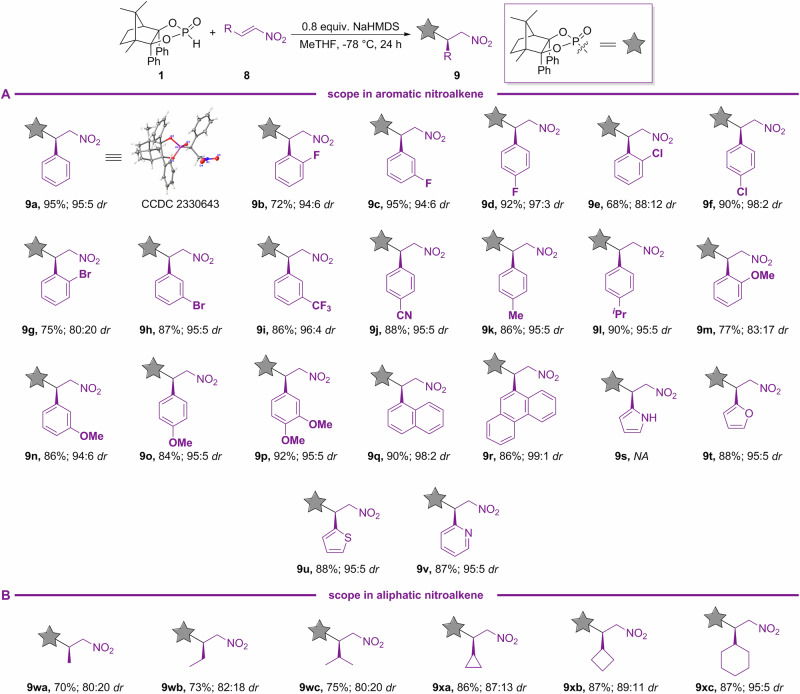
Scope of β-nitro phosphonates. **A** Scope in aromatic nitroalkene. **B** Scope in aliphatic nitroalkene.

Having developed the optimal reaction conditions for diastereoselective Pudovik reaction for the synthesis of β-aminophosphonates, we next turned our attention to the nitroalkene substrate scope (Fig. 7). As disclosed in Fig. 7, a diverse range of nitroalkenes can be readily tolerated to furnish the desired enantioenriched β-nitrophosphonates. In most cases of the aromatic nitroalkenes, excellent levels of asymmetric induction and good conversion were obtained with either a *meta-* or a *para-*substituent (F, Cl, Br, CF_3_, CN, Me, ^*i*^Pr, and OMe) of **8a** (**9c**–**9d,** **9f,** **9h**–**9l,** **9n**–**9p**). Comparatively, an *ortho*-group of **8a**, such as F, Br, and OMe, gave the products in relatively low yield and *dr*, suggesting that a neighboring group of **8a** is adverse to this transformation (**9b,** **9g,** and **9m**). Nitroalkene incorporated with a naphthyl or phenanthryl ring was readily tolerated with even better diastereoselectivity (**9q**–**9r**). Especially for a more rigid phenanthryl ring, a *dr* of 99:1 was observed (**9r**). Except pyrrole (**9s**), other heteroaromatic nitroalkenes with a furan, thiophene, or pyridine ring, occurred successfully to provide β-nitrophosphonates with excellent yields and high levels of enantiocontrol (**9t**–**9v**). As for aliphatic nitroalkenes, straight-chain alkyl (**9wa**–**9wb**), branched alkyl (**9wc**), and cycloalkyl (**9xa**–**9xc**) all delivered the corresponding products with good yield and stereocontrol. The absolute configuration of the new chiral center for β-nitrophosphonate of **9a** was assigned as *R*-configuration by X-ray crystallography.

**Fig. 8:**
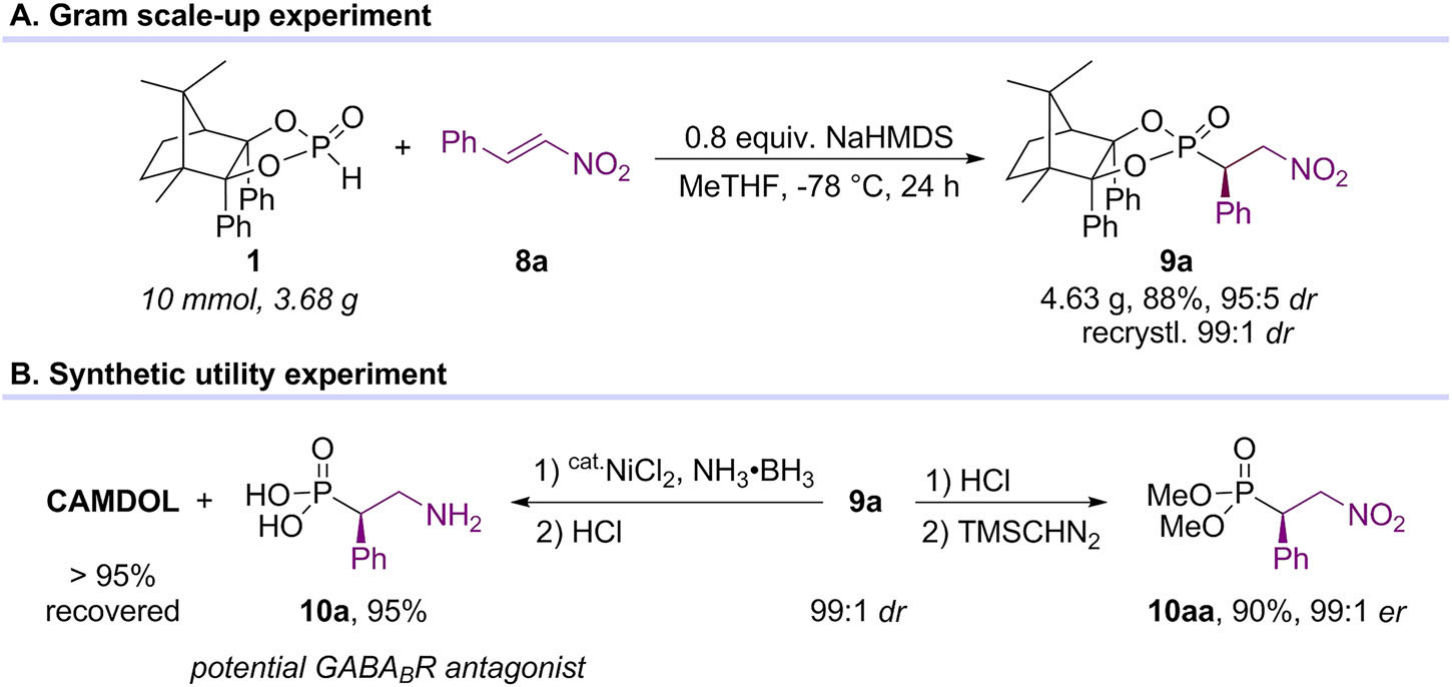
Synthetic utility experiments of nitroalkene-based Pudovik reaction. **A** Gram scale-up experiment. **B** Synthetic utility experiment.

A demonstration of the preparative utility of nitroalkene-based Pudovik reaction was presented by the addition of CAMDOL-PHO **1**–**8a**, which was performed on a 10 mmol scale with NaHMDS as the base to afford **9a** in 88% yield and 95:5 *dr* (Fig. 8A). After recrystallization, the *dr* value of **9a** could be improved to 99:1. Exposure of **9a** by catalytic NiCl_2_ with NH_3_·BH_3_, followed by removal of CAMDOL using HCl, provided the corresponding β-amino phenylphosphonic acid **10a**—a potential GABA_B_ receptor antagonist^55,56^—in 95% yield. Simultaneously, >95% of CAMDOL was recovered. The *er* of **10a** was further confirmed by CAMDOL cleavage, followed by diazomethylation with TMSCHN_2_, to yield **10aa** with 99:1 *er* (Fig. 8B).

### Mechanistic insights

**Fig. 9:**
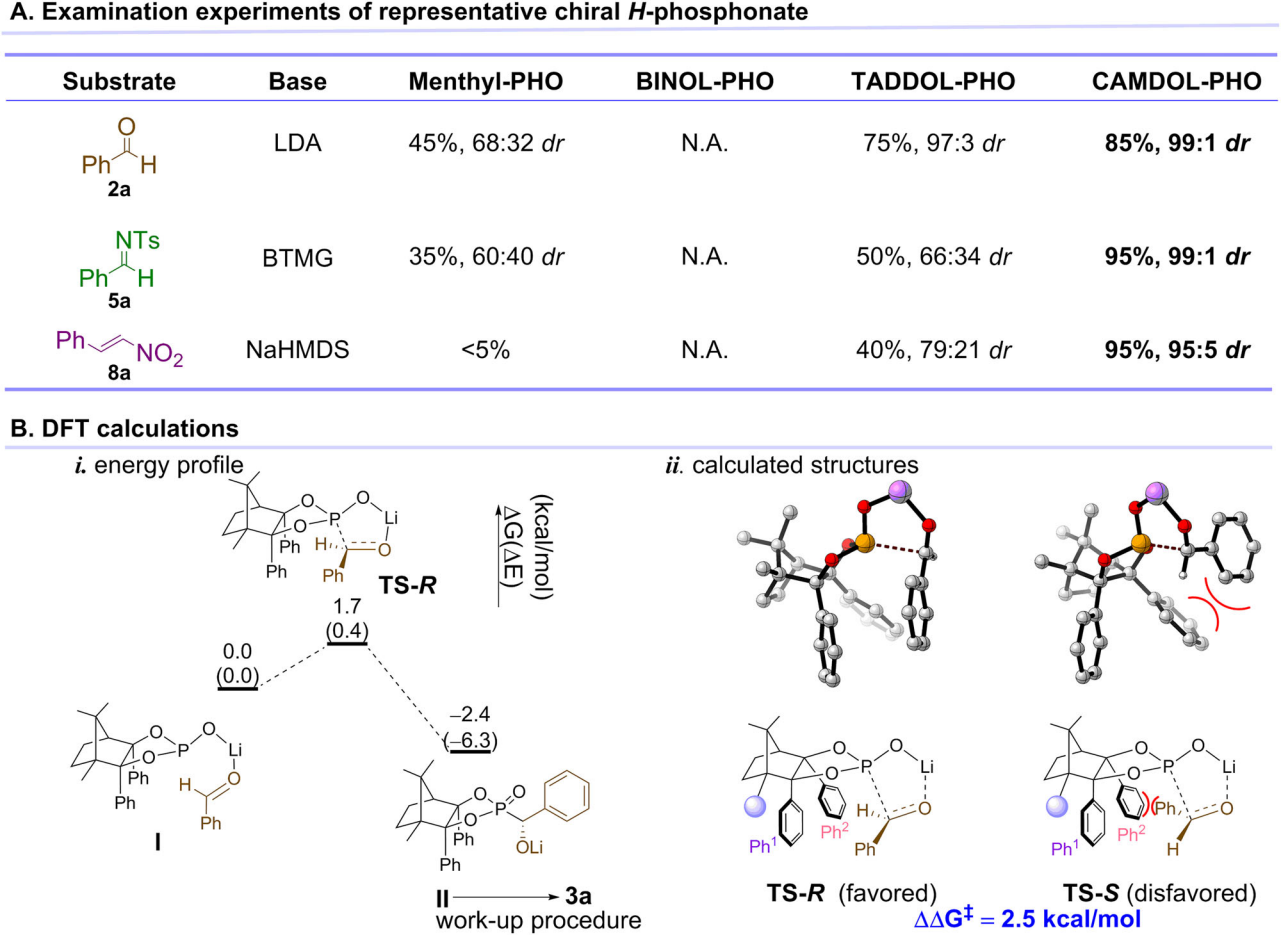
Mechanistic studies. **A** Control experiments of the representative chiral *H*-phosphonates for Pudovik reaction under our optimal conditions. The *dr* of Menthyl-PHO, BINOL-PHO, TADDOL-PHO are all >99:1. **B** DFT calculations. **i** Energy profile for the P–C formation; **ii** Calculated structures of the transition states.

Control experiments using Menthyl-PHO, TADDOL-PHO, and BINOL-PHO as representative chiral *H*-phosphonates were conducted for asymmetric Pudovik reaction with aldehyde **2a**, aldimine **5a**, and nitroalkene **8a**, respectively. The results demonstrated that TADDOL-PHO exhibited higher conversion and diastereomeric ratio than Menthyl-PHO and BINOL-PHO, though still lower than CAMDOL-PHO (Fig. 9A). Notably, BINOL-PHO failed to react with any substrate under our optimal conditions. These findings highlight the significantly superior stereoinductive capability of centrally chiral CAMDOL-PHO over other CA-derived H-phosphonates in Pudovik reaction.

The results showed that both the conversion and diastereomeric ratio of TADDOL-PHO were superior to Menthyl-PHO and BINOL-PHO, albeit inferior to those of CAMDOL-PHO (Fig. 9A). Notably, BINOL-PHO did not react with any tested substrate under the corresponding optimal conditions. Obviously, the center-chiral CAMDOL-PHO has a very superior stereoinduction ability for Pudovik reaction than other types of CA derived *H*-phosphonates.

Density functional theory (DFT) calculations were carried out to further investigate the origin of the stereoselectivity in the Pudovik reaction of **2a** with CAMDOL-PHO (Fig. 9B). Deprotonation of the phosphoric acid with LDA is barrierless and irreversible, yielding Li-phosphonate (Supplementary Fig. S349^†^). Coordination of **2a** to the Li^+^ facilitates the formation of a stable complex **I**, which is prone to the hydride transfer process. Driven by alkali metal ion activation, the nucleophilic attack of phosphonate on **2a** requires only a barrier of 1.7 kcal/mol to form the P–C bond. The work-up procedure with NH_4_Cl is performed to convert compound **II** into the final product **3a** (Fig. 9B-i).

The privileged scaffold of CAMDOL induces an asymmetric reaction pocket (Fig. 9B-ii). The methyl group on the chiral carbon causes the adjacent phenyl group (Ph^1^) to adopt a vertical orientation, while the other phenyl group (Ph^2^) protrudes from the plane. This geometry divides the pocket into two distinct sections: a large area and a small area, allowing for the differentiation of substituents on the substrates. Upon examining the enantiomeric transition states for P–C bond formation, it was observed that significant steric hindrance occurs between the phenyl group of **2a** and the extended Ph^2^ of CAMDOL-PHO (Fig. 9B-ii, **TS-*S***). On the contrary, the orientation of Ph^1^ reserves ample space for the bulky substituent of the substrate, resulting in a lower energy transition state **TS-*R*** that favors the production of the *R*-configured product **II**. The energy barrier difference between the two transition states is 2.5 kcal/mol, corresponding to a calculated *ee* value of 97%, which closely aligns with the experimental value of 98%.

## Discussion

In summary, we have developed CAMDOL-PHO, a novel centrally chiral H-phosphonate, for diastereoselective Pudovik reaction, allowing direct access to carbon-phosphorus stereogenicity in a wide variety of structural contexts. Based on CAMDOL-PHO, three subtypes of Pudovik reaction, involving aldehydes, aldimines, and nitroalkenes were established, displaying broad functional group tolerance, operational simplicity, scalable practicability, and diverse applicability for the preparation of bioactive molecules. Furthermore, these hydrophosphorylations of unsaturated bonds demonstrate atom economy, as all key starting materials are incorporated into the products, coupled with facile recovery of the CA for late-stage diversification. We anticipate that this P-chiral *H*-phosphonate will complement existing CA-PHOs in asymmetric induction and emerge as a privileged scaffold for constructing high-value molecular architectures.

## Methods

### Synthesis of CAMDOL-PHO (1)

A 500 mL three-necked flask equipped with a stirrer was charged with CAMDOL (6.44 g, 20 mmol). The flask was sealed with a gas-tight septum and subjected to evacuation, followed by three cycles of backfilling with N₂. Subsequently, anhydrous THF (200 mL) was added, followed by the addition of Et_3_N (8.4 mL, 3 equiv.) using a syringe. The reaction mixture was stirred at 0 °C for 0.5 h. Thereafter, PCl_3_ (19.2 mL, 1 M in THF, 1.1 equiv.) was introduced dropwise via syringe at 0 °C and stirred for an additional 2 h. During this period, ^31^P NMR was employed to confirm the complete conversion of the reaction. Then, NaOMe (7.5 mL, 5.4 M, 3 equiv.) was added at 0 °C for another 2 h. After the full conversion confirmed by ^31^P NMR, the reaction mixture was quenched with saturated aqueous NH_4_Cl solution and allowed to stir until no further effervescence occurred. The phases were then separated, the aqueous layer underwent extraction twice with EtOAc. The combined organic layers were dried over Na_2_SO_4_, filtered, and concentrated under reduced pressure. Finally, purification of the crude product was achieved through flash column chromatography.

### General procedure for Pudovik reaction

As for the aldehyde-based Pudovik reaction, a 25 mL Schlenk tube equipped with a stirrer was charged with CAMDOL-PHO (**1**, 0.4 mmol, 1 equiv.). When a solid aldehyde was employed, it was added at this stage. The vial was sealed with a gas-tight septum and subjected to evacuation followed by three cycles of backfilling with N_2_. Subsequently, dry THF (4 mL) was introduced, followed by the addition of aldehyde (**2**, 1.2 equiv.) using a microsyringe. The reaction mixture was stirred at −78 °C for 0.5 h. Thereafter, LDA (0.48 mL, 1 M in THF, 1.2 equiv.) was administered dropwise via syringe at −78 °C and stirred for an additional 24 h. During this period, ^31^P NMR was utilized to verify the complete conversion of the reaction. Next, the reaction was quenched with saturated aqueous NH_4_Cl solution and allowed to stir until no further effervescence was observed. The phases were then separated, and the aqueous layer underwent extraction twice with EtOAc. The combined organic layers were dried over Na_2_SO_4_, filtered, concentrated, and finally purified by flash column chromatography.

As for the aldimine-based Pudovik reaction, aldehyde was replaced with aldimine (**5**, 1.2 equiv.), and LDA was substituted with BTMG (1.2 equiv.) on the basis of above conditions. As for the nitroalkene-based Pudovik reaction, THF was substituted with 2-MeTHF, aldehyde was replaced with nitroalkene (**8**, 1.2 equiv.), and LDA was replaced with LiHMDS (0.8 equiv.).

## Supplementary information

Supplementary Information

Description of Additional Supplementary Files

Supplementary Data 1

Supplementary Data 2

Supplementary Data 3

Supplementary Data 4

Supplementary Data 5

Supplementary Data 6

## Data Availability

General information, experimental details, and analytical data: NMR spectra, HRMS data and computational details can be found in the Supplementary Information. The X-ray crystallographic coordinates for structures reported in this study have been deposited at the Cambridge Crystallographic Data Centre (CCDC), under deposition numbers 2402270 (**1**), 2330640 (**3a**), 2330642 (**6a**), and 2330643 (**9a**), respectively. These data can be obtained free of charge from The Cambridge Crystallographic Data Centre via www.ccdc.cam.ac.uk/data_request/cif and are enclosed as Supplementary Data 1–4. Computational chemistry details are available in Supplementary Data 5–6. All data are available from the corresponding author upon request. Source data are provided with this paper.
