# Supplementary material for: Stereoselective Pudovik reaction of aldehydes, aldimines, and nitroalkenes with CAMDOL-derived H-phosphonate
Source: Commun Chem. 2025 Nov 14;8:349. doi: 10.1038/s42004-025-01735-4 (PMC12618634; doi:10.1038/s42004-025-01735-4)
Supplement: Supplementary file 1 — Supplementary Information [file 42004_2025_1735_MOESM1_ESM.pdf]

Supporting Information

Stereoselective Pudovik Reaction of Aldehydes, Aldimines, and Nitroalkenes with CAMDOL-Derived *H*-Phosphonate

Ning Li, Qian Wu, Yu Huang, Li Pan, Junchen Li*, Enxue Shi* and Junhua Xiao*

State Key Laboratory of NBC Protection for Civilian, Beijing102205, P. R. China.

E-mails: lijch07@lzu.edu.cn; exshi@sina.com; xiao.junhua@pku.edu.cn

Contents

1. General information 1

2. Experimental section 1

2.1 General procedure for the preparation of CAMDOL-PHO **1** 1

2.2 General procedure for aldehyde-based asymmetric Pudovik reaction 4

2.2.1 Graphical procedure for synthesis of α-hydroxyphosphonate **3**...............4

2.2.2 General procedure for synthesis of compounds **4a, 4c** & **4d** 7

2.2.3 General procedure for synthesis of compound **4b, 4ca** & **4da**..................9

2.3 General procedure for Aldimine-based asymmetric Pudovik reaction 10

2.3.1 General procedure for synthesis of compounds **6** 10

2.3.2 General procedure for synthesis of compounds **7a &7b**.........................11

2.3.3 General procedure for synthesis of compounds **7aa & 7ba**....................11

2.4 General procedure for nitroalkene-based asymmetric Pudovik reaction........12

2.4.1 General procedure for synthesis of compounds **9** 12

2.4.2 General procedure for synthesis of compound **10a**................................13

2.4.3 General procedure for synthesis of compound **10aa**..............................14

2.5 Other tested substrates 14

2.5.1 Carbonyl substrates...14

2.5.2 Aldimine substrates 15

2.5.3 Alkene substrates..16

2.5.4 Other P-chiral CA-PHOs..17

3. Characterization data of compounds 20

3.1 Characterization data of CAMDOL-PHO **1** 20

3.2 Characterization data of aldehyde-based Pudovik reaction 20

3.3 Characterization data of aldimine-based Pudovik reaction 45

3.4 Characterization data of nitroalkene-based Pudovik reaction 71

3.5 Characterization data of Menthyl-PHO, TADDOL-PHO adducts..................92

3.6 Characterization data of the recovered epoxide-CAMDOL 95

4. Copies of NMR spectra...96

5. HPLC Diagrams...268

6. X-ray crystallography data 276

6.1 X-ray crystallography data of CAMDOL-PHO **1**..276

6.2 X-ray crystallography data of **3a** 278

6.3 X-ray crystallography data of **6a** 280

6.4 X-ray crystallography data of **9a** 282

7. Results of DFT calculations..284

8. References..285

1. General information

Reagents were purchased at the highest commercial quality and used without further purification, unless otherwise stated. All reactions were performed in oven-dried glassware with magnetic stirring. NMR spectra were recorded on a Bruker 300 instrument and calibrated using residual undeuterated solvents. The following abbreviations were used to explain multiplicities: *s* = singlet, *d* = doublet, *t* = triplet, *q* = quartet, *m* = multiplet, *br* = broad. Reactions were monitored by LC/MS, thin layer chromatography (TLC), or ^{31}P NMR. Flash column chromatography was performed using Qingdao Haiyang silica (silica gel for thin-layer chromatography, HG/T2354-2010). High-resolution mass spectra (HRMS) were obtained on an Agilent 6545 Q-TOF HPLC and mass spectrometry. HPLC analysis were conducted on an Agilent 1260 LC/MSC or Waters UPC2 SFC equipped with a photodiode array detector. Preparative HPLC were conducted on a Buchi Pure C850 instrument with a MZ PerfectChrom 100 C8 Prep column (30 ID*150 mm, 5 μm).

The enantiomeric ratios were determined with Waters UPC2 SFC equipped with a photodiode array detector or an Agilent1260 LC/MSC. The diastereomeric ratio were determined with ^{31}P NMR or Agilent1260 LC/MSC. Melting points (mp) were recorded using a Shanghai Yidian Wuguang SGW X-4A. Melting point apparatus and are uncorrected. Optical rotations were measured on an Austria Anton Paar MCP 200 polarimeter using a Na lamp (λ = 589 nm, D-line). $[\alpha]_D^{25}$ values were measured at 25 ℃ and in a 1 mg/mL concentration. The single crystal X-ray diffraction data was collected at 170 K on a XtaLAB AFC12 (RINC): Kappa single or XtaLAB Synergy, Dualflex, HyPix or SuperNova, Dual, Cu at home/near, EosS2 diffractometer.

2. Experimental section

2.1 General procedure for the preparation of CAMDOL-PHO 1

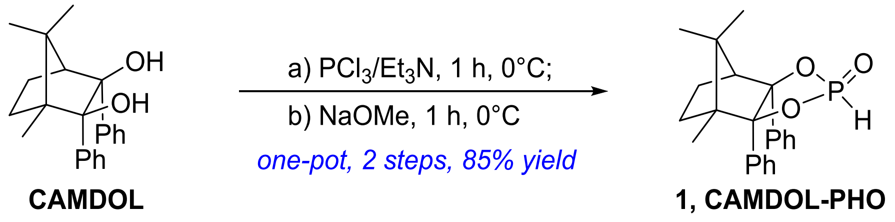

A 500 mL two-necked flask equipped with a stirrer was charged with **CAMDOL**[1] (6.44 g, 20 mmol). The flask was sealed with a gas-tight septum and subjected to evacuation, followed by three cycles of backfilling with N_2. Subsequently, anhydrous THF (200 mL) was added, followed by the addition of triethylamine (8.4 mL, 3 equiv.) using a syringe. The reaction mixture was stirred at 0 ℃ for 30 minutes. Thereafter, PCl_3 (19.2 mL, 1 M in THF; 1.1 equiv.) was introduced dropwise via syringe while maintaining stirring at 0 ℃ for an additional two hours. Following this period, ^{31}P NMR was employed to confirm the complete conversion of the reaction system.

Subsequently, NaOMe (7.5 mL, 5.4 M, 3 equiv.) was added while continuing to stir at 0 ℃ for another two hours. After this duration, ^{31}P NMR again confirmed the complete conversion of the reaction system. The reaction mixture was quenched with saturated aqueous ammonium chloride solution and allowed to stir until no further effervescence occurred. The phases were then separated; the aqueous layer underwent extraction twice with ethyl acetate (EA). The combined organic layers were dried over Na_2SO_4, filtered, and concentrated under reduced pressure. Finally, purification of the crude product was achieved through flash column chromatography.

Graphical guide for the synthesis of CAMDOL-PHO 1

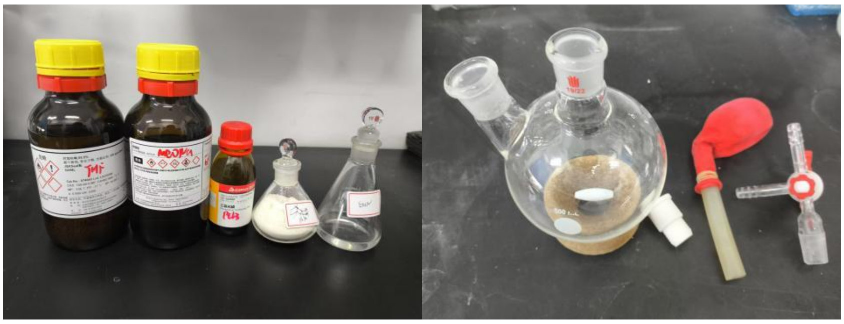

(Left) Reagents needed. All solids were weight out: CAMDOL (6.44 g, 20 mmol). THF (200 mL), PCl_3 (19.2 mL, 1 M in THF; 1.1 equiv.) Et_3N (8.4 mL, 3 equiv.). MeONa (7.5 mL, 5.4 M, 3 equiv.). (**Right**) Clean 500 mL two-necked flas with stir bar, a rubber stopper, a balloon attached to a hose and a triple valve.

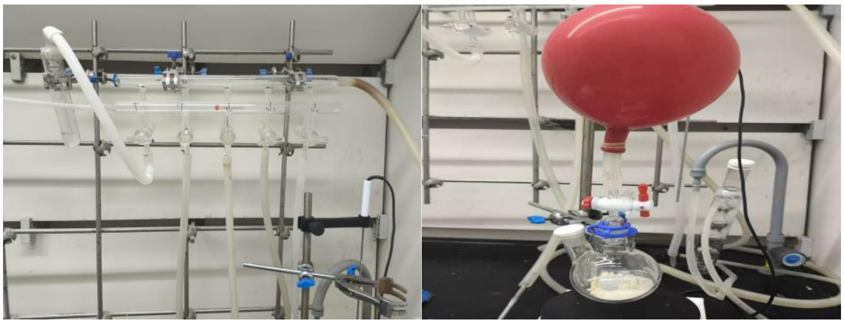

(**Left**) Double rows of tubes for the experiment. (**Right**) The state in which the reaction system is filled with nitrogen.

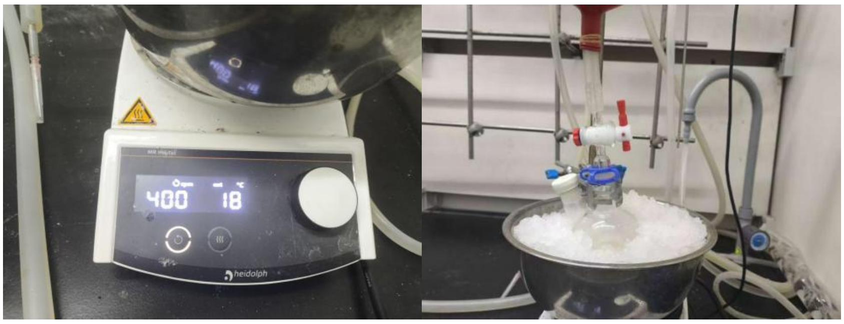

In the nitrogen atmosphere, add THF, Et_3N, PCl_3, and MeONa reagent in sequence. (**Left**) The reaction system reacts on the agitator. (**Right**) The reaction system is carried out under an ice bath.

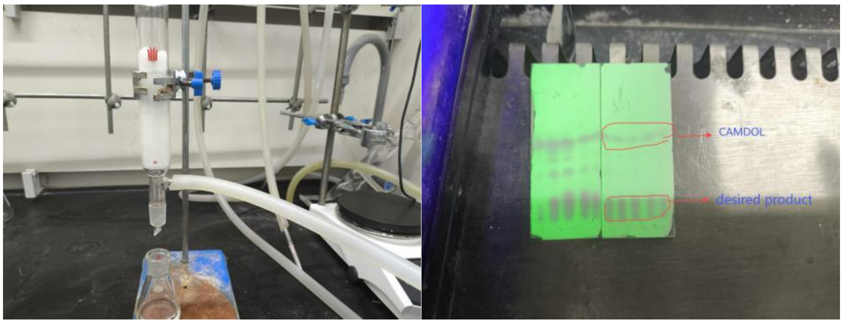

After the reaction completion, it is quenched, extracted, dried and concentrated to obtain the crude product. Then it was purified through silica gel column chromatography. **(Left)** The crude product was purified by silica gel column chromatography. **(Right)** TLC condition (5:1 Hexanes: EtOAc).

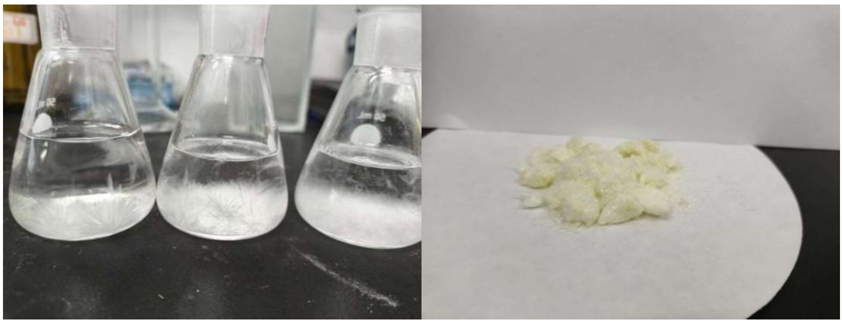

Under the hydrolysis of SiO_2[2], we obtained the final product. **(Left)** The product precipitates from the eluent and forms crystals. **(Right)** The final product **CAMDOL-PHO 1. Notes:** (The crystals precipitating on the left side at this time can be used directly for X-ray crystallography testing, but the products obtained cannot be concentrated and re-cultivated with the EA/hexane system to obtain X-ray crystallography testing again, otherwise only camphor-epoxide[1] compounds can be obtained.)

2.2 General procedure for aldehyde-based asymmetric Pudovik reaction

2.2.1 Graphical procedure for synthesis of α-hydroxyphosphonate 3

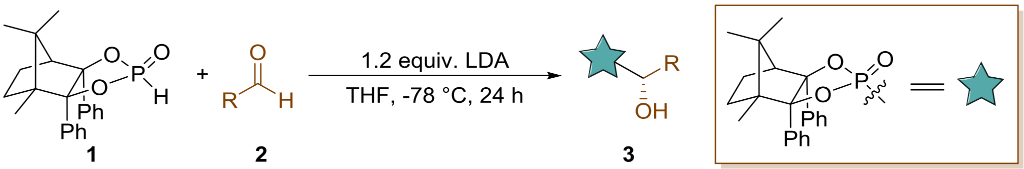

A 25 mL Schlenk tube equipped with a stirrer was charged with **CAMDOL-PHO**

(**1**, 0.4 mmol, 1 equiv.). If solid aldehydes were employed, they were introduced at this stage. The vial was sealed with a gas-tight septum and subjected to evacuation followed by three cycles of backfilling with N_2. Subsequently, dry THF (8 mL) was added, followed by the addition of aldehydes (**2**, 0.48 mmol, 1.2 equiv.) using a microsyringe. The reaction mixture was stirred at -78 ℃ for 30 minutes. Thereafter, the LDA (0.48 mL, 1M in THF, 1.2 equiv.) reagent was administered dropwise via syringe while maintaining stirring at -78 ℃ for an additional 24 hours. Following this period, ^{31}P NMR spectroscopy was utilized to verify the complete conversion of the reaction system. The reaction was quenched with saturated aqueous NH_4Cl solution and allowed to stir until no further effervescence was observed. The phases were then separated, and the aqueous layer underwent extraction twice with ethyl acetate. The combined organic layers were dried over Na_2SO_4, filtered, and concentrated under reduced pressure. Finally, the crude product was purified by column chromatography.

Graphical guide for the synthesis of compound 3a

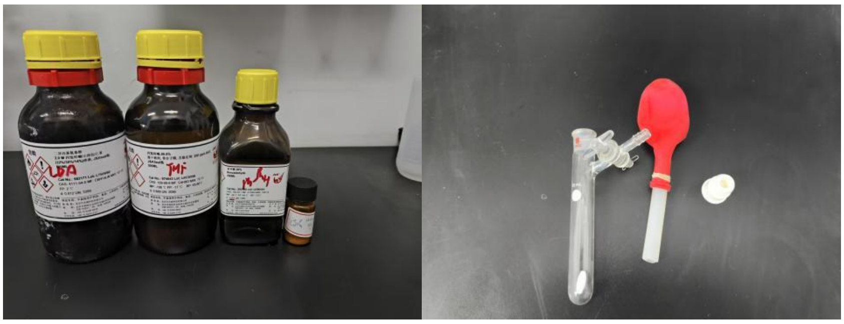

(Left) Reagents needed. All solids were weight out: **CAMDOL-PHO 1** (0.15 g, 0.4 mmol), THF (8 mL), Benzaldehyde (0.05 ml, 0.48 mmol, 1.2 equiv.), LDA (0.48 mL, 1M in THF, 1.2 equiv.). (**Right**) Clean 25 mL Schlenk tubes with stir bar, a rubber stopper and a balloon attached to a hose.

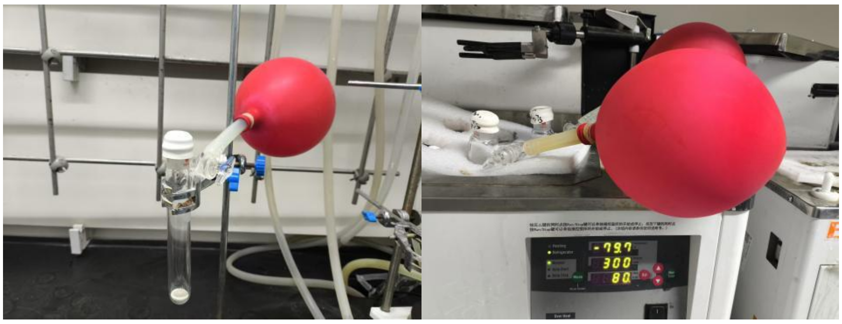

(Left) The state in which the reaction system is filled with nitrogen. (**Right)** The reaction system is carried out under -78 ℃.

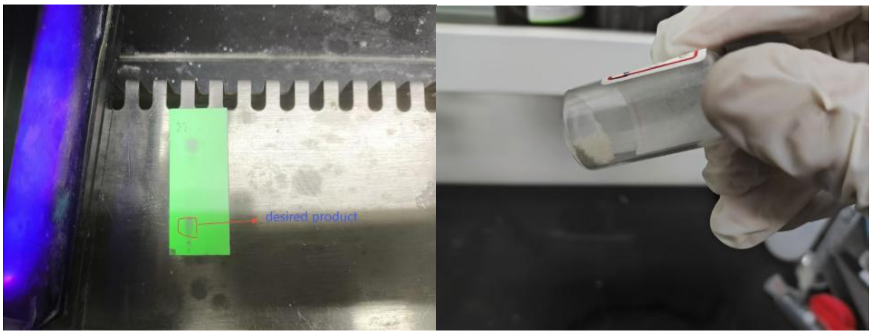

After the reaction is complete, it is quenched, extracted, dried and concentrated obtain the crude product. Then the crude product was purified by column chromatography. **(Left)** TLC condition (2:1 Hexanes: EtOAc). (**Right)** The final product **3a** (162 mg, 85% yield).

2.2.2 General procedure for synthesis of compounds 4a, 4c & 4d

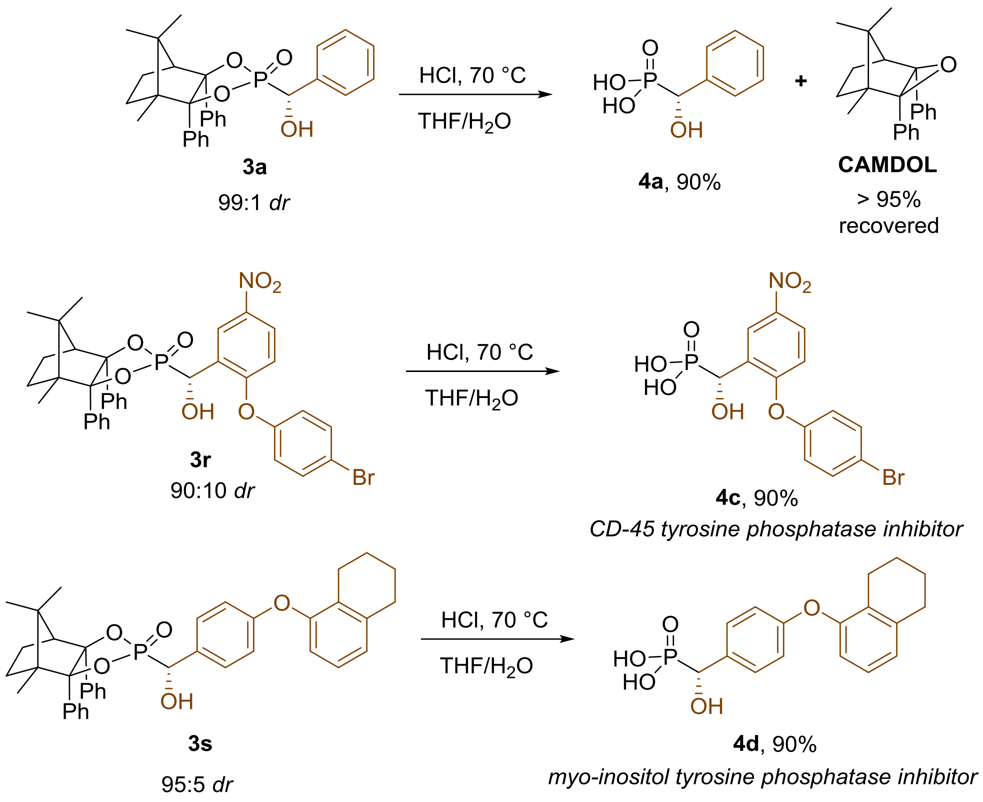

A 50 mL Schlenk tube was charged with 0.4 mmol of compound **3** (**3a**, **3r**, or **3s**), followed by the addition of anhydrous THF (4 mL). The solution was stirred for a duration of 5 minutes. Subsequently, aqueous HCl (8M, 4 mL) was introduced, and the mixture was heated at 70 ℃ for a period of 6 hours. Upon completion of the transformation, as confirmed by ^{31}P NMR, the Schlenk tube was allowed to cool to room temperature. The resulting biphasic mixture was transferred to a separating funnel; the aqueous phase was collected while discarding the organic phase. The aqueous layer was then concentrated under reduced pressure. Finally, purification of the crude product[3-6] was achieved through a series of three washes with dichloromethane. The organic phase is concentrated and recrystallized to obtain camphor-epoxide[1] (CAMDOL).

Graphical guide for the synthesis of compound 4a

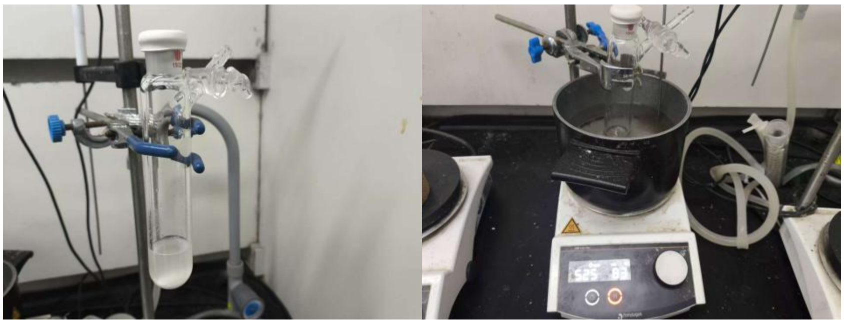

(Left) The product **3a** was dissolved back into the THF solution. (**Right)** Add the hydrochloric acid solution, and remove the CAMDOL at 70 ℃.

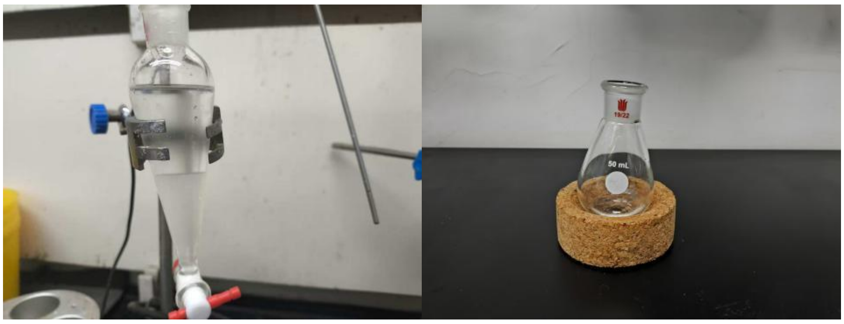

(Left) After the reaction is complete, add ethyl acetate for extraction. (**Right)** Remove the organic phase and retain the aqueous phase.

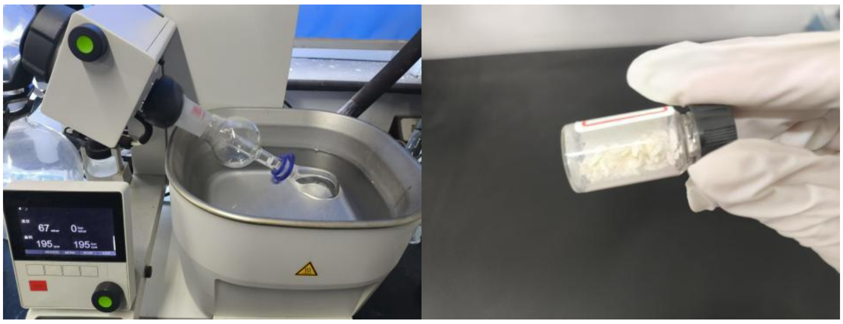

(Left) The aqueous layer was then concentrated under reduced pressure. (**Right)** The final product **4a** (68 mg, 90% yield).

2.2.3 General procedure for synthesis of compound 4b, 4ca & 4da

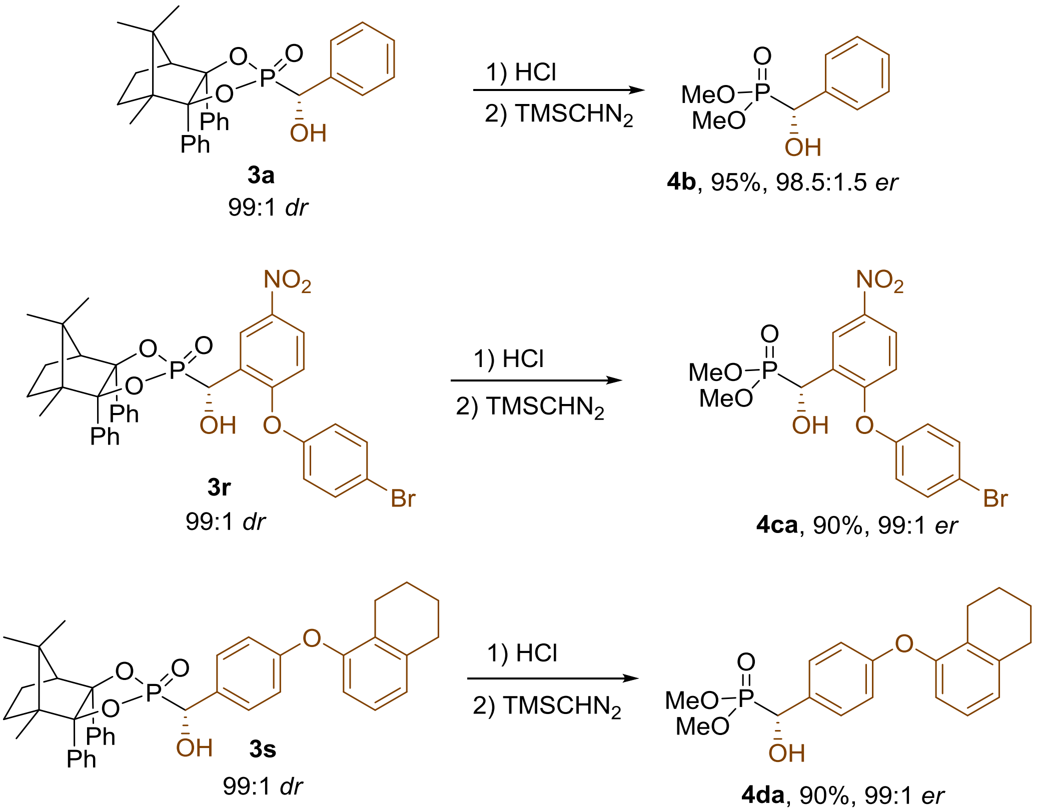

After hydrolysis of compound **3a**, **3r**, or **3s** (0.4 mmol) to yield crude in the presence of HCl, the reaction mixture was concentrated. Then $TMSCHN_2$ (2 mL, 1M in THF, 10 equiv.) and MeOH (8 mL) was subsequently added, and the resulting solution was stirred at room temperature for 12 hours. Upon completion of the transformation, as confirmed by ^{31}P NMR, the reaction mixture was concentrated again. Finally, the product[7] was purified through column chromatography.

Graphical guide for the synthesis of compound 4b

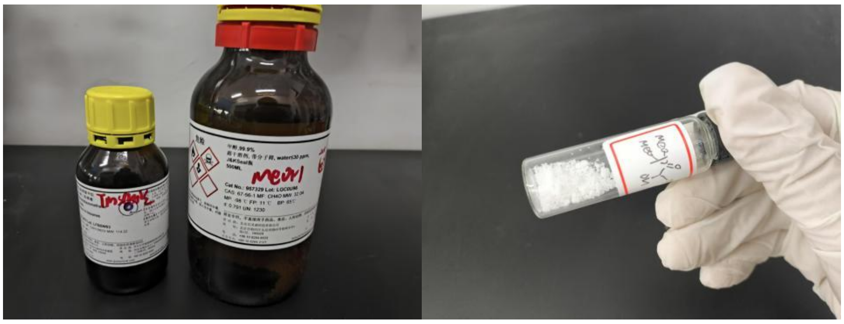

After removing CAMDOL, the crude product **4a** obtained was dissolved again in

methanol and treated with $TMSCH_2N_2$. After the reaction is complete, the final product is obtained through column chromatography. **(Left)** Reagents needed. (**Right)** The final product **4b** (82 mg, 95% yield).

2.3 General procedure for Aldimine-based asymmetric Pudovik reaction

2.3.1 General procedure for synthesis of compounds 6

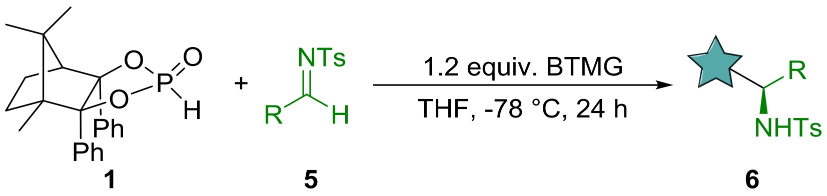

A 25 mL Schlenk tube equipped with a stirrer was charged with **CAMDOL-PHO** (**1**, 0.4 mmol, 1 equiv.), aldimines (**5**, 1.2 equiv.). The vial was sealed with a gas-tight septum and subjected to evacuation followed by three cycles of backfilling with N_2. Subsequently, dry THF (4 mL) was added using a syringe. The reaction mixture was stirred at -78 ℃ for 30 minutes. Thereafter, BTMG (0.1 mL, 1.2 equiv.) reagent was administered dropwise via syringe while maintaining stirring at -78 ℃ for an additional 24 hours. Following this period, ^{31}P NMR was utilized to verify the complete conversion of the reaction system. The reaction was quenched with saturated aqueous NH_4Cl solution and allowed to stir until no further effervescence was observed. The phases were then separated, and the aqueous layer underwent extraction twice with ethyl acetate. The combined organic layers were dried over Na_2SO_4, filtered, and concentrated under reduced pressure. Finally, the crude product was purified by column chromatography.

2.3.2 General procedure for synthesis of compounds 7a & 7b

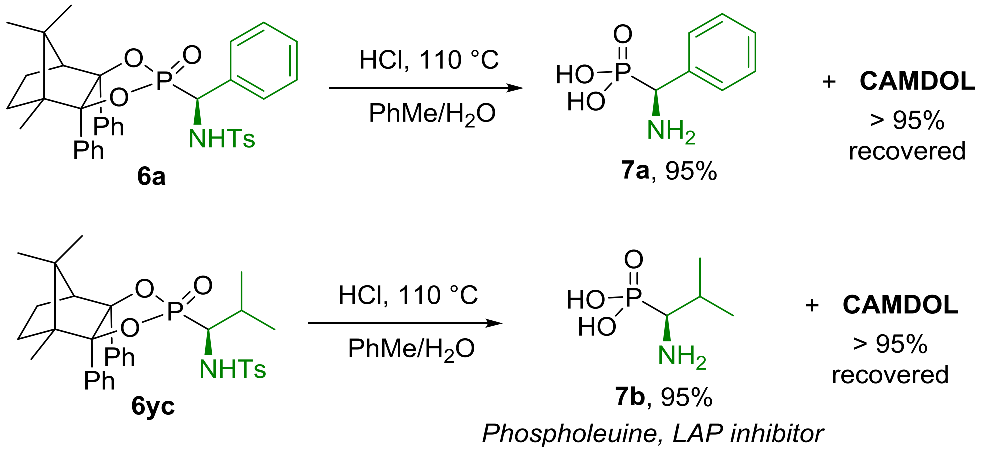

A 50 mL Schlenk tube was charged with 0.4 mmol of compound **6 (6a**, **6ya)** and dry PhMe (4 mL) was added. The solution was stirred for 5 minutes. Subsequently, aqueous HCl (8M, 4 mL) was introduced, and the mixture was heated at 110 ℃ for a period of 48 hours. Upon completion of the heating process, the Schlenk tube was allowed to cool to room temperature. The resulting biphasic mixture was transferred to a separating funnel; the aqueous phase was collected while discarding the organic phase. The aqueous layer was then concentrated under reduced pressure. Finally, purification of the crude product [8-13] was achieved through a series of three washes with dichloromethane.

2.3.3 General procedure for synthesis of compounds 7aa & 7ba

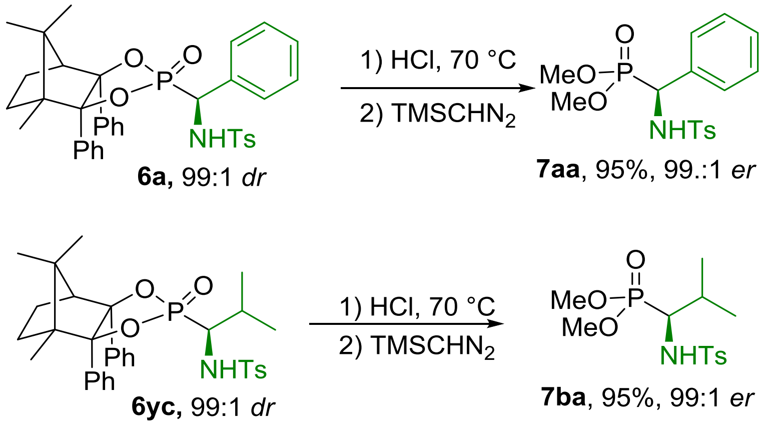

A 50 mL Schlenk tube was charged with 0.4 mmol of compound **6 (6a**, **6ya)** and dry THF(4 mL) was added. The solution was stirred for 5 minutes. Subsequently, aqueous HCl (8M, 4 mL) was introduced, and the mixture was heated at 70 ℃ for a period of 6

hours. Upon completion of the heating process, the Schlenk tube was allowed to cool to room temperature. Transfer the resulting two-phase mixture to a rotary evaporator and dry it to obtain an oily product. Then $TMSCHN_2$ (2 mL, 1M in THF, 10 equiv.) and MeOH (8 mL) was subsequently added, and the resulting solution was stirred at room temperature for 12 hours. Upon completion of the transformation, as confirmed by ^{31}P NMR, the reaction mixture was concentrated again. Finally, the product was purified through column chromatography.

2.4 General procedure for nitroalkene-based asymmetric Pudovik reaction

2.4.1 General procedure for synthesis of compounds 9

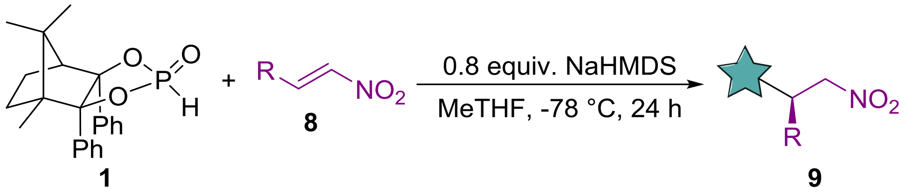

A 10 mL Schlenk tube equipped with a stirrer was charged with **CAMDOL-PHO** (**1**, 0.2 mmol, 1 equiv.). If solid nitroalkenes were employed, they were introduced at this stage. The vial was sealed with a gas-tight septum and subjected to evacuation followed by three cycles of backfilling with N_2. Subsequently, dry THF (4 mL) was added, followed by the addition of nitroalkenes (**8**, 1.2 equiv.) using a microsyringe. The reaction mixture was stirred at -78 ℃ for 30 minutes. Thereafter, the NaHMDS (0.16 mL, 1M in THF, 0.8 equiv.) reagent was administered dropwise via syringe while maintaining stirring at -78 ℃ for an additional 24 hours. Following this period, ^{31}P NMR was utilized to verify the complete conversion of the reaction system. The reaction was quenched with saturated aqueous NH_4Cl solution and allowed to stir until no further effervescence was observed. The phases were then separated, and the aqueous layer underwent extraction twice with ethyl acetate. The combined organic layers were dried over Na_2SO_4, filtered, and concentrated under reduced pressure. Finally, the crude product was purified by column chromatography.

2.4.2 General procedure for synthesis of compound 10a

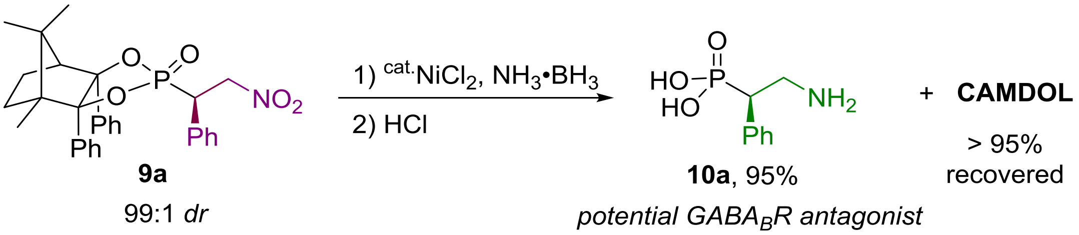

A 50 mL Schlenk tube was charged with **9a** (0.2 mmol), NH_3BH_3 (1.6 mmol, 8 equiv.), and $NiCl_2$ (0.1 mmol, equiv.). Anhydrous THF (4 mL) and H_2O (4 mL) were subsequently added to the mixture. The resulting solution was stirred at room temperature for a duration of 12 hours. Upon completion of the transformation, as confirmed by ^{31}P NMR, the mixture was filtered and concentrated under reduced pressure.

The crude product was then re-dissolved in tetrahydrofuran (4 mL), followed by the addition of aqueous HCl (8M, 4 mL). The mixture was heated at 70 ℃ for a period of 6 hours. After heating, the Schlenk tube was allowed to cool to room temperature. The resulting biphasic mixture was transferred to a separating funnel; the aqueous phase was collected while discarding the organic phase. The aqueous layer underwent concentration under reduced pressure once more. Finally, purification of the crude product[14] was achieved through a series of three washes with dichloromethane.

Graphical guide for the synthesis of compound 10a

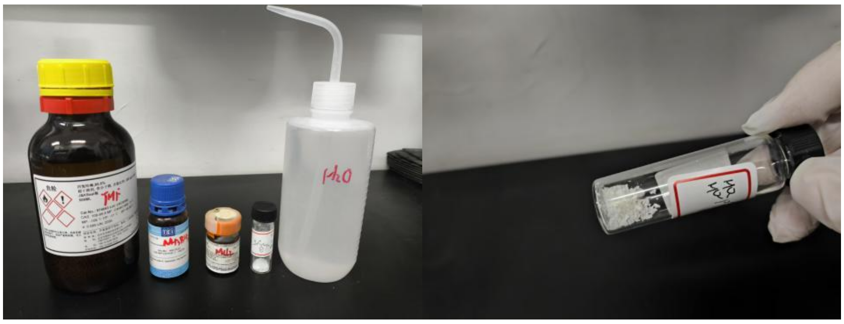

The resulting product **9a** was dissolved again in THF, then NH_3BH_3, $NiCl_2$ and water are added to initiate the reaction, in which the nitro group is reduced to the amino group. After the reaction is complete, saturated ammonium chloride is added to quench the reaction, the organic phase is extracted, retained, and the organic phase is rotated to dry, yielding a crude product. Then, the steps of product **4a** are repeated to obtain the final

product. **(Left)** Reagents needed. (**Right)** The final product **10a** (44 mg, 95% yield).

2.4.3 General procedure for synthesis of compound 10aa

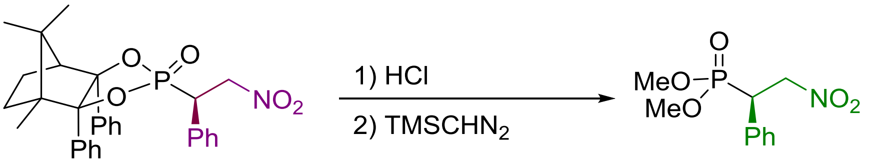

9a
99:1 *dr*

10aa, 90%, 99:1 *er*

A 50 mL Schlenk tube was charged with 0.4 mmol of compound **9a** and dry THF(4 mL) was added. The solution was stirred for 5 minutes. Subsequently, aqueous HCl (8M, 4 mL) was introduced, and the mixture was heated at 70 ℃ for a period of 6 hours. Upon completion of the heating process, the Schlenk tube was allowed to cool to room temperature. Transfer the resulting two-phase mixture to a rotary evaporator and dry it to obtain an oily product. Then $TMSCHN_2$ (2 mL, 1M in THF, 10 equiv.) and MeOH (8 mL) was subsequently added, and the resulting solution was stirred at room temperature for 12 hours. Upon completion of the transformation, as confirmed by ^{31}P NMR, the reaction mixture was concentrated again. Finally, the product was purified through column chromatography.

2.5 Other tested substrates

2.5.1 Carbonyl substrates

The alkenyl aldehydes used in the experiment were all of the *E* stereoisomer. We also tried other types of carbonyl substrates, but unfortunately, no reaction occurred.

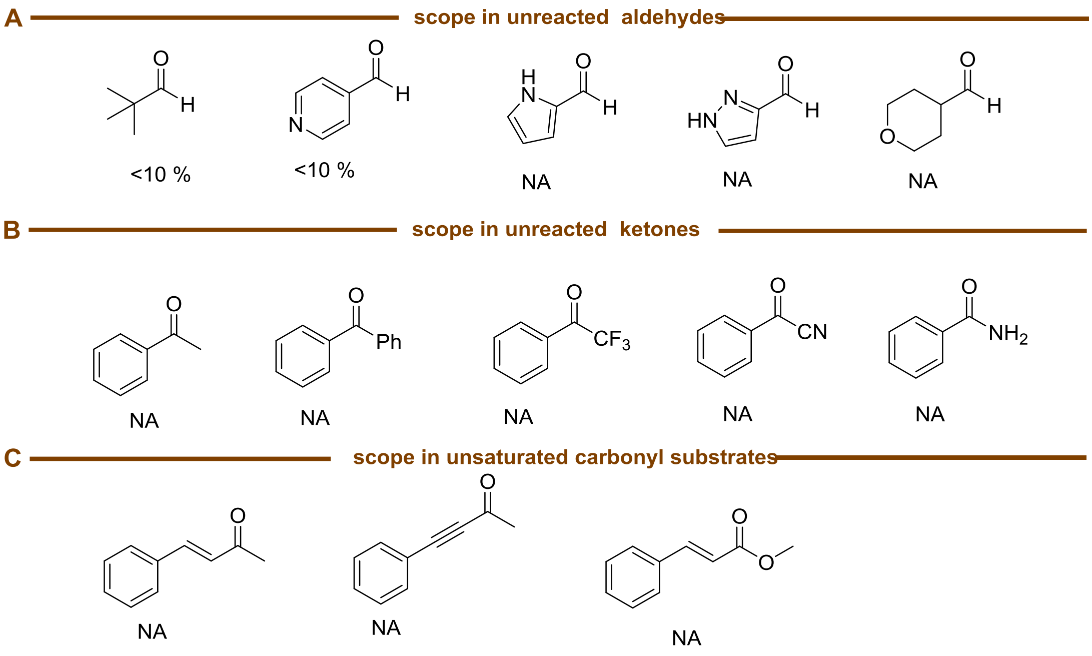

2.5.2 Aldimine substrates

The aldimine substrates employed in this experiment, with the exception of those depicted as follows, are mixtures of *Z* and *E* stereoisomers.

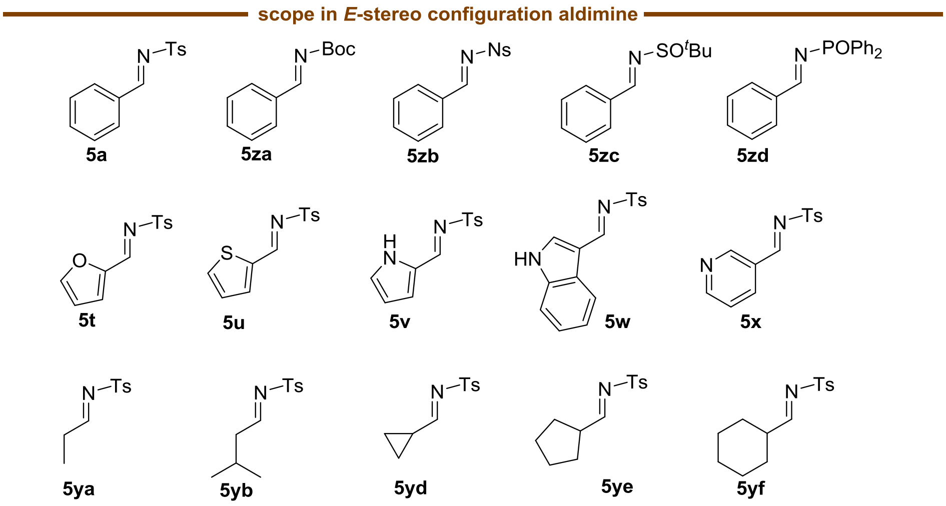

We tried other types of imines as substrates, but unfortunately, no reaction occurred.

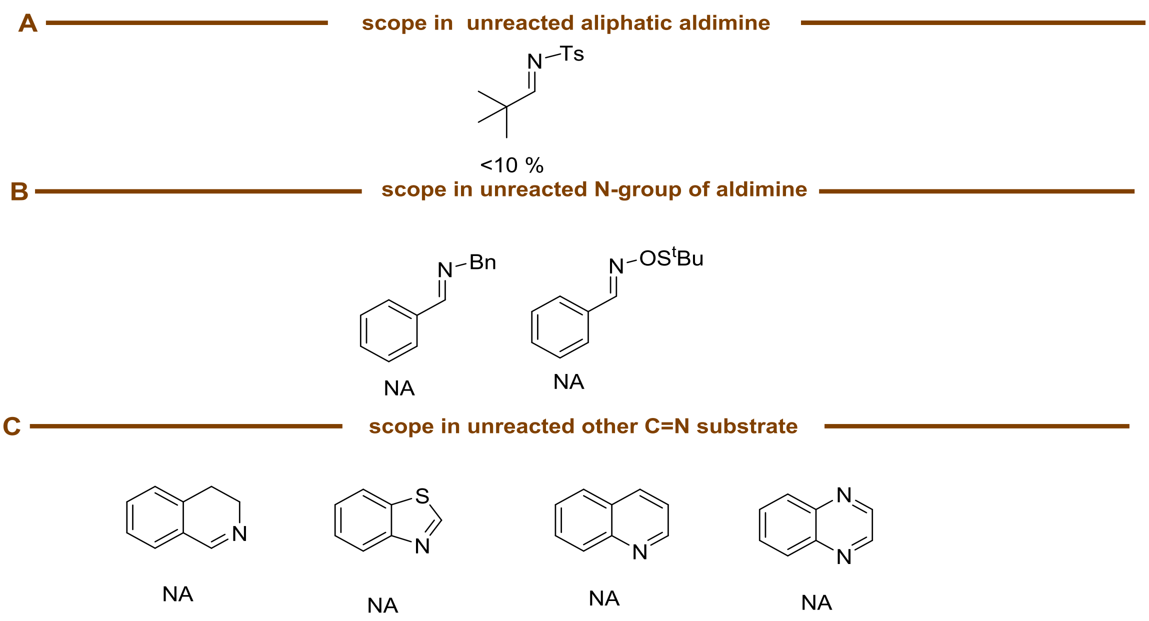

2.5.3 Alkene substrates

The nitroalkene substrates employed in this experiment, with the exception of those depicted as follows, are mixtures of *Z* and *E* stereoisomers.

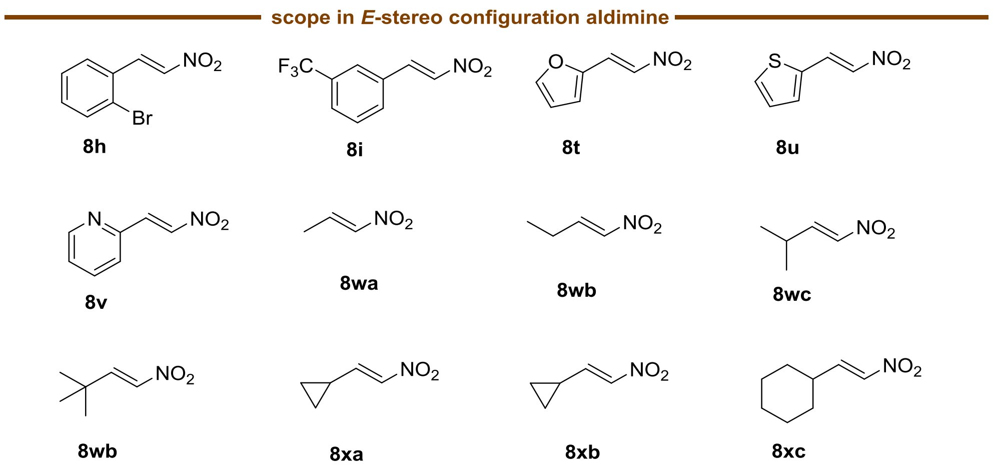

We tried other types of alkene substrates, but unfortunately, the reaction did not occur.

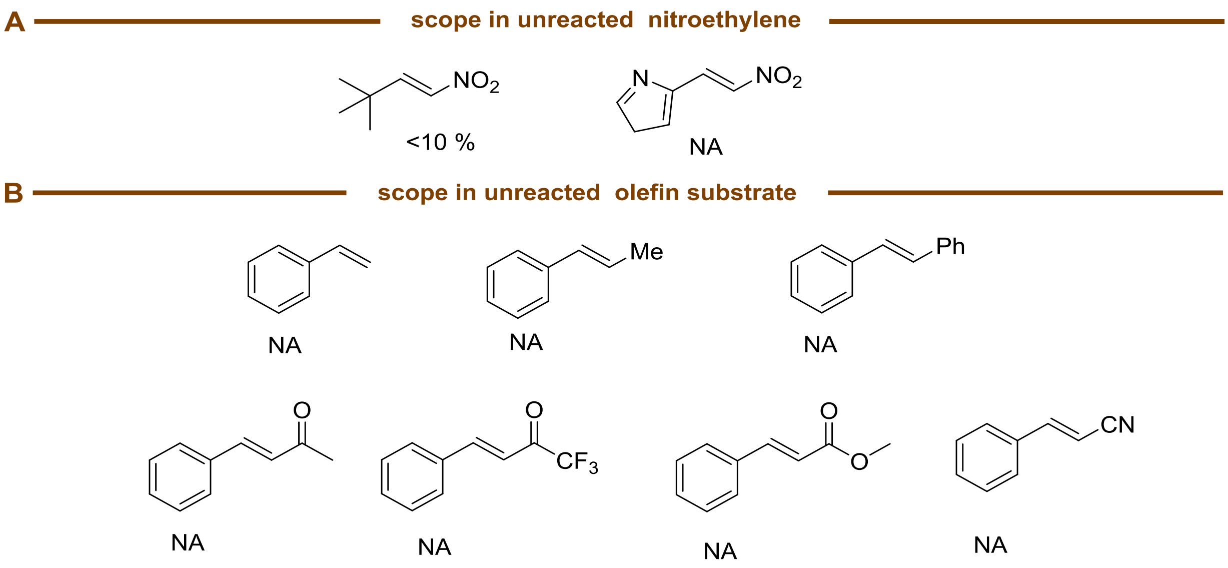

2.5.4 Other P-chiral CA-PHO

We repeated the experimental procedures for synthesizing compounds **3a, 6a**, and **9a** using other P-Chiral CA-PHO (Menthyl-PHO, BINOL-PHO and TADDOL-PHO) and the results showed that BINOL-PHO did not undergo any reaction, while Menthyl-PHO, TADDOL-PHO underwent a reaction, resulting in the synthesis of compounds.

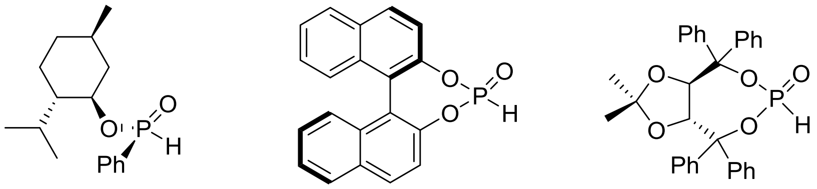

Menthyl-PHO **BINOL-PHO** **TADDOL-PHO**

2.5.4.1 Pudovik reaction with Menthyl-PHO

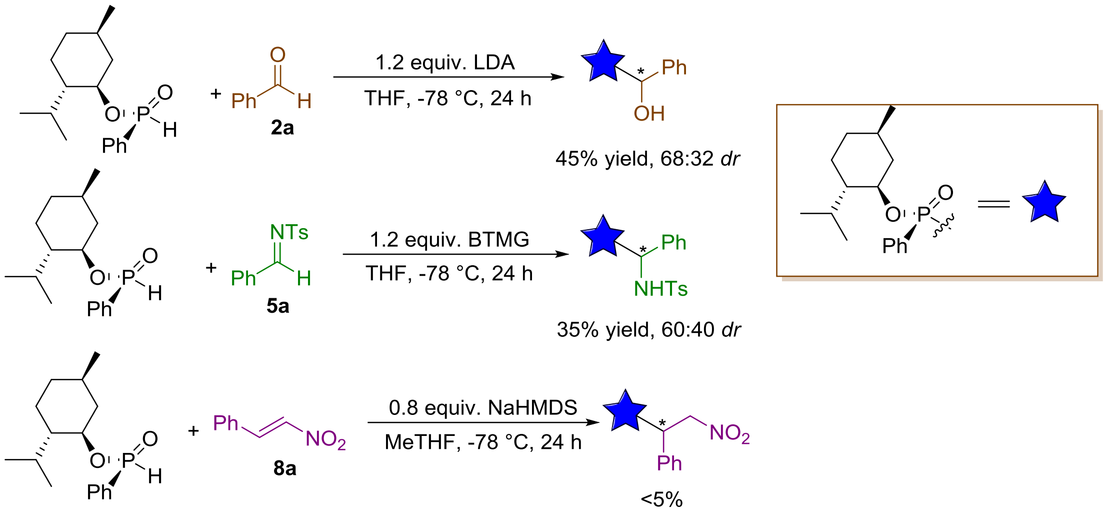

2.5.4.2 Pudovik reaction with BINOL-PHO

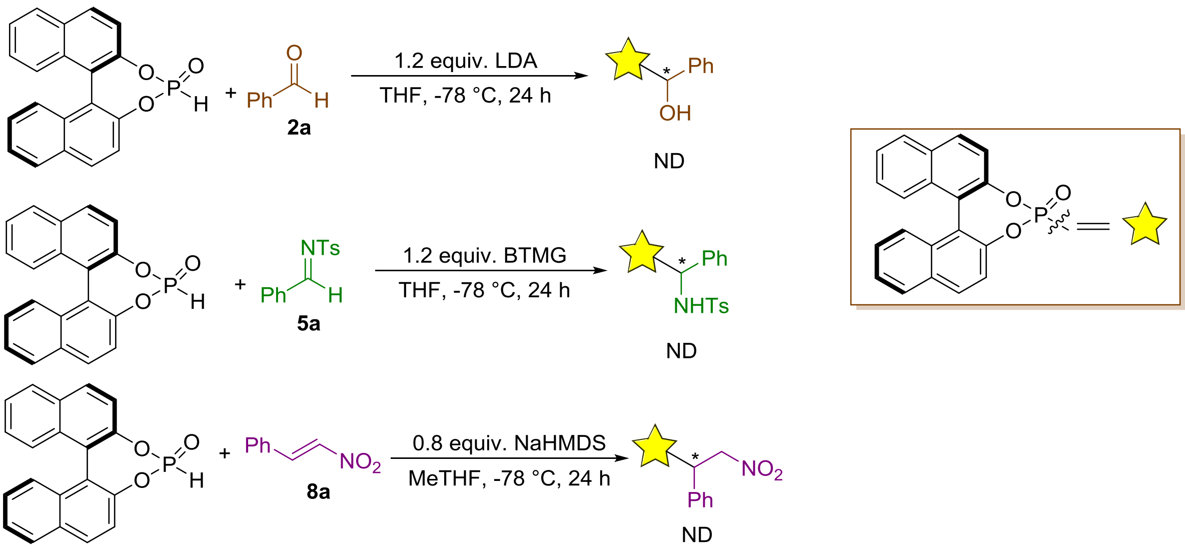

2.5.4.3 Pudovik reaction with BINOL-PHO

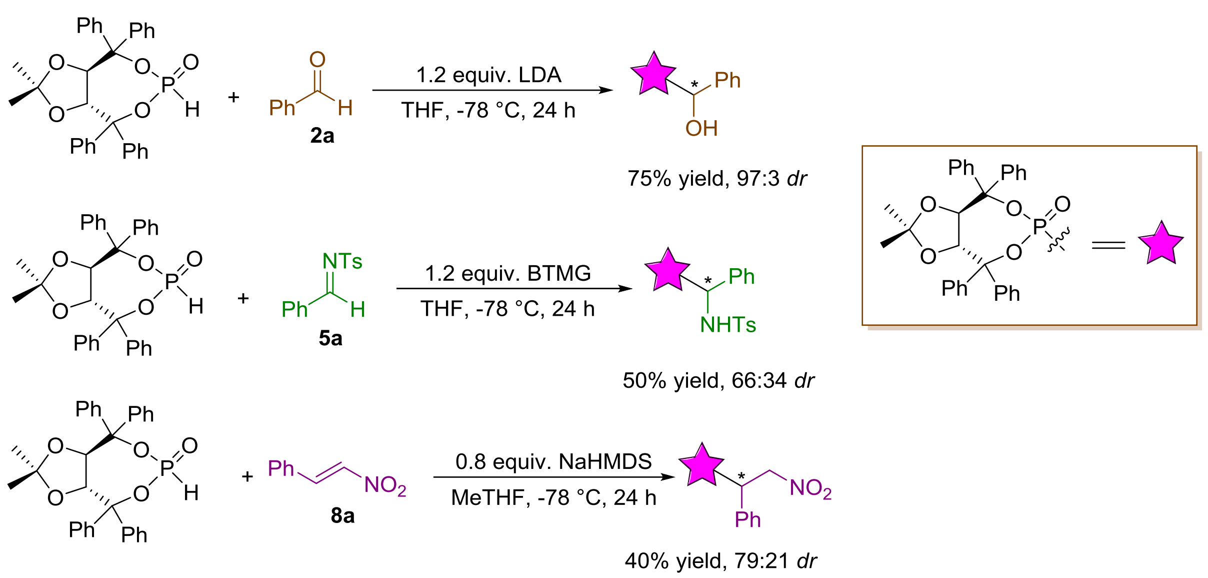

3. Characterization data of compounds

3.1 Characterization data of CAMDOL-PHO 1

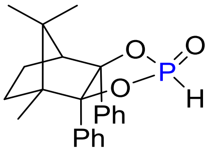

CAMDOL-PHO 1

(3a*S*,4*S*,7*S*,7a*S*)-4,8,8-Trimethyl-3a,7a-diphenylhexahydro-4,7-methanobenzo[*d*][1,3,2]-dioxaphosphole 2-oxide (**CAMDOL-PHO 1**).

$[\alpha]_D^{25}$ = +42.00 °(c = 1.00 in MeOH);

The residue was purified by flash chromatography (PE/EtOAc = 5:1) on silica gel to afford the product (6.26g, 85% yield), white solid. R_f = 0.42 (PE/EA = 5:1).

m.p. = 119.8-120.8 ℃, R_f = 0.42 (PE/EA = 5:1).

^{1}H NMR (300 MHz, Chloroform-*d*) δ 7.38 (d, *J* = 698.3 Hz, 1H), 7.97 -6.51 (m, 10H), 2.96 (d, *J* = 5.4 Hz, 1H), 2.23 - 2.07 (m, 2H), 1.79 - 1.62 (m, 4H), 1.40 - 1.20 (m, 2H), 1.16 (s, 3H), 0.94 (s, 3H).

^{13}C NMR (75 MHz, Chloroform-*d*) δ 137.05, 136.83, 130.53, 128.01, 127.64, 127.10, 126.14, 101.81 (d, *J* = 12.0 Hz), 100.62 (d, *J* = 10.2 Hz), 56.08 (d, *J* = 3.7 Hz), 52.46 (d, *J* = 3.4 Hz), 48.20, 29.32, 26.13, 24.35, 21.84, 21.58, 10.53.

^{31}P NMR (122 MHz, Chloroform-*d*) δ 16.27.

HRMS (ESI-MS) calculated for $C_{22}H_{25}O_3PNa[M+Na]^+$: 391.1434, found: 391.1437.

3.2 Characterization data of aldehyde-based Pudovik reaction

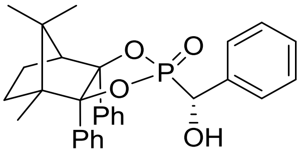

3a

(3a*S*,4*S*,7*S*,7a*S*)-2-((*R*)-Hydroxy(phenyl)methyl)-4,8,8-trimethyl-3a,7a-diphenylhexahy-dro-4,7-methanobenzo[*d*][1,3,2]dioxaphosphole 2-oxide (3a).

$[\alpha]_D^{25}$ = +2.98 °(c = 1.00 in MeOH);

The residue was purified by flash chromatography (PE/EtOAc = 2:1) on silica gel to afford the product (161 mg, 85% yield), white solid. R_f = 0.35 (PE/EA = 2:1).

m.p. = 116.8-118.8 ℃

^{1}H NMR (300 MHz, Chloroform-*d*) δ 7.56 - 6.69 (m, 15H), 4.78 (d, *J* = 7.4 Hz, 1H), 3.06 (d, *J* = 5.4 Hz, 1H), 2.16 - 1.99 (m, 1H), 1.77 (s, 3H), 1.65 - 1.50 (m, 1H), 1.31- 1.22 (m, 2H), 1.15 (s, 3H), 0.79 (s, 3H).

^{13}C NMR (75 MHz, Chloroform-*d*) δ 138.16 (d, *J* = 3.3 Hz), 136.59, 136.03 (d, *J* = 4.8 Hz), 130.31 (d, *J* = 5.3 Hz), 128.80 (d, *J* = 2.7 Hz), 128.71 (d, *J* = 2.3 Hz), 128.28 (d, *J* = 6.9 Hz), 128.01 (d, *J* = 5.2 Hz), 126.24, 101.81, 99.32 (d, *J* = 1.8 Hz), 73.87, 71.98, 55.68 (d, *J* = 2.6 Hz), 52.06 (d, *J* = 3.3 Hz), 49.12, 48.29, 29.89, 26.56, 24.52, 21.64, 9.85.

^{31}P NMR (122 MHz, Chloroform-*d*) δ36.76.

HRMS (ESI-MS) calculated for $C_{29}H_{32}O_4P$ $[M+H]^+$: 475.2038, found: 475.2033.

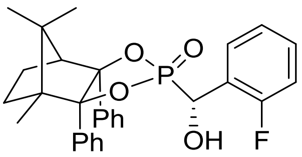

3b

(3a*S*,4*S*,7*S*,7a*S*)-2-((*R*)-(2-Fluorophenyl)(hydroxy)methyl)-4,8,8-trimethyl-3a,7a-diphenyl-hexahydr-o-4,7-methanobenzo[*d*][1,3,2]dioxaphosphole 2-oxide (3b).

$[\alpha]_D^{25}$ = +7.00 °(c = 1.00 in MeOH);

The residue was purified by flash chromatography (PE/EtOAc = 2:1) on silica gel to afford the product (118 mg, 60% yield), white solid. R_f = 0.40 (PE/EA = 2:1).

m.p. = 68.0-70.0 ℃

^{1}H NMR (300 MHz, Chloroform-*d*) δ 7.78 – 7.63 (m, 1H), 7.46 – 6.86 (m, 12H), 6.72 (s, 1H), 5.14 (d, *J* = 9.3 Hz, 1H), 3.07 (d, *J* = 5.4 Hz, 1H), 2.15 – 1.94 (m, 1H), 1.76 (s, 3H), 1.62 – 1.48 (m, 3H), 1.31 – 1.21 (m, 3H), 1.15 (s, 3H), 0.77 (s, 3H).

^{13}C NMR (75 MHz, Chloroform-*d*) δ 161.63 (d, *J* = 8.4 Hz), 158.34 (d, *J* = 11.9 Hz), 137.76 (d, *J* = 3.0 Hz), 135.79 (d, *J* = 4.8 Hz), 130.60-130.18 (m), 129.97 (d, *J* = 14.9 Hz), 128.72, 128.01, 127.78, 125.22-124.33 (m), 115.81, 115.51, 101.81, 99.74 (d, *J* = 2.1 Hz), 66.15 (d, *J* = 3.3 Hz), 64.24 (d, *J* = 3.2 Hz), 55.55 (d, *J* = 2.7 Hz), 51.83 (d, *J* = 3.4 Hz), 48.29, 29.89, 26.52, 24.34, 21.64, 9.73.

^{19}F NMR (282 MHz, Chloroform-*d*) δ -112.60 (td, *J* = 9.3, 6.1 Hz).

^{31}P NMR (121 MHz, Chloroform-*d*) δ 37.33 (d, *J* = 5.3 Hz).

HRMS (ESI-MS) calculated for $C_{29}H_{31}FO_4P$ $[M+H]^+$: 493.1944, found: 493.19445.

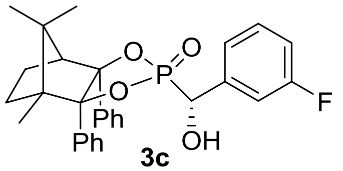

3c

(3a*S*,4*S*,7*S*,7a*S*)-2-((*R*)-(3-Fluorophenyl)(hydroxy)methyl)-4,8,8-trimethyl-3a,7a-diphenyl-hexahydr-o-4,7-methanobenzo[*d*][1,3,2]dioxaphosphole 2-oxide (3c).

$[\alpha]_D^{25}$ = +11.97 °(c = 1.00 in MeOH);

The residue was purified by flash chromatography (PE/EtOAc = 2:1) on silica gel to afford the product (142 mg, 72% yield), white solid. R_f = 0.40 (PE/EA = 2:1).

m.p. = 99.5.-101.5 ℃

^{1}H NMR (300 MHz, Chloroform-*d*) δ 7.49 - 6.71 (m, 14H), 5.21 (s, 1H), 4.72 (d, *J* = 7.9 Hz, 1H), 2.98 (d, *J* = 5.3 Hz, 1H), 2.15 - 1.86 (m, 1H), 1.64 (s, 3H), 1.55 -1.38 (m, 1H), 1.28 - 1.16 (m, 2H), 1.07 (s, 3H), 0.73 (s, 3H).

^{13}C NMR (75 MHz, Chloroform-*d*) δ 164.30, 161.05 (d, *J* = 2.2 Hz), 139.10 (d, *J* = 7.2 Hz), 137.99 (d, *J* = 3.3 Hz), 135.95 (d, *J* = 4.7 Hz), 130.43-129.90 (m), 128.64, 128.19, 127.93, 126.28, 123.63 (dd, *J* = 6.4, 3.0 Hz), 115.63 (d, *J* = 2.8 Hz), 115.43-115.19 (m), 115.02 (d, *J* = 6.5 Hz), 101.97, 99.40 (d, *J* = 1.9 Hz), 73.21 (d, *J* = 2.0 Hz), 71.30 (d, *J* = 1.9 Hz), 55.61 (d, *J* = 2.7 Hz), 51.96 (d, *J* = 3.3 Hz), 48.13, 29.79, 26.49, 24.50, 21.44, 9.83.

^{19}F NMR (282 MHz, Chloroform-*d*) δ -112.60 (td, *J* = 9.3, 6.1 Hz).

^{31}P NMR (122 MHz, Chloroform-*d*) δ 35.87.

HRMS (ESI-MS) calculated for $C_{29}H_{31}FO_4P$ $[M+H]^+$: 493.1944, found: 493.1946.

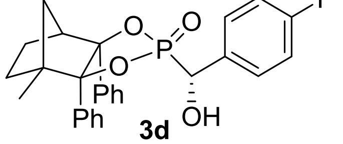

3d

(3a*S*,4*S*,7*S*,7a*S*)-2-((*R*)-(4-Fluorophenyl)(hydroxy)methyl)-4,8,8-trimethyl-3a,7a-diphenyl-hexahydr-o-4,7-methanobenzo[*d*][1,3,2]dioxaphosphole 2-oxide (3d).

$[\alpha]_D^{25}$ = +20.99 °(c = 1.00 in MeOH);

The residue was purified by flash chromatography (PE/EtOAc = 2:1) on silica gel to afford the product (152 mg, 77% yield), white solid. R_f = 0.42 (PE/EA = 2:1).

m.p. = 108.0-109 ℃

^{1}H NMR (300 MHz, Chloroform-*d*) δ 7.50-6.81 (m, 14H), 4.77 (d, *J* = 7.2 Hz, 1H), 3.30 (s, 1H), 3.05 (d, *J* = 5.3 Hz, 1H), 2.17-1.99 (m, 1H), 1.71 (s, 3H), 1.56 (dt, *J* = 14.7, 6.9 Hz, 1H), 1.28 (tt, *J* = 9.7, 7.0 Hz, 2H), 1.14 (s, 3H), 0.79 (s, 3H).

^{13}C NMR (75 MHz, Chloroform-*d*) δ 164.65, 161.38, 138.07 (d, *J* = 3.3 Hz), 136.00 (d, *J* = 4.7 Hz), 132.48 (d, *J* = 3.2 Hz), 130.66-129.73 (m), 128.68, 128.17, 127.97, 126.26, 115.69 (d, *J* = 1.8 Hz), 115.41 (d, *J* = 1.8 Hz), 101.86, 99.38, 99.35, 73.09, 71.17, 55.63 (d, J = 2.7 Hz), 51.99 (d, *J* = 3.2 Hz), 48.20, 29.78, 26.50, 24.50, 21.51, 9.84.

^{19}F NMR (282 MHz, Chloroform-*d*) δ -113.19 (tq, *J* = 9.2, 5.0 Hz).

^{31}P NMR (121 MHz, Chloroform-*d*) δ 36.32 (d, *J* = 4.4 Hz).

HRMS (ESI-MS) calculated for $C_{29}H_{31}FO_4P$ $[M+H]^+$: 493.1944, found: 493.1943.

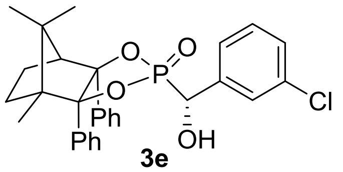

3e

(3a*S*,4*S*,7*S*,7a*S*)-2-((*R*)-(3-Chlorophenyl)(hydroxy)methyl)-4,8,8-trimethyl-3a,7a-diphenyl-hexahydr-o-4,7-methanobenzo[*d*][1,3,2]dioxaphosphole 2-oxide (**3e**).

$[\alpha]_D^{25}$ = +10.00 °(c = 1.00 in MeOH);

The residue was purified by flash chromatography (PE/EtOAc = 2:1) on silica gel to afford the product (154 mg, 76% yield), white solid. R_f = 0.38 (PE/EA = 2:1).

m.p. = 135.1-137.1 ℃

^{1}H NMR (300 MHz, Chloroform-*d*) δ 7.34-7.13 (m, 12H), 7.09-6.85 (m, 2H), 4.77 (d, *J* = 8.1 Hz, 1H), 3.79 (s, 1H), 3.03 (d, *J* = 5.3 Hz, 1H), 2.09 (dt, *J* = 10.3, 6.0 Hz, 1H), 1.69 (s, 3H), 1.56 (ddd, *J* = 14.6, 8.0, 5.4 Hz, 1H), 1.27 (t, *J* = 7.8 Hz, 2H), 1.13 (s, 3H), 0.80 (s, 3H).

^{13}C NMR (75 MHz, Chloroform-*d*) δ 138.66, 138.01, 135.94 (d, *J* = 4.6 Hz), 134.18, 130.24 (d, *J* = 7.6 Hz), 129.91, 128.66, 128.25 (d, *J* = 5.6 Hz), 127.93, 126.24 (d, *J* = 6.3 Hz), 102.09, 99.47, 73.18, 71.27, 55.64, 52.02, 48.13, 29.83, 26.50, 24.51, 21.46, 9.83.

^{31}P NMR (121 MHz, Chloroform-*d*) δ 35.78.

HRMS (ESI-MS) calculated for $C_{29}H_{31}ClO_4P$ $[M+H]^+$: 509.1648, found: 509.1645.

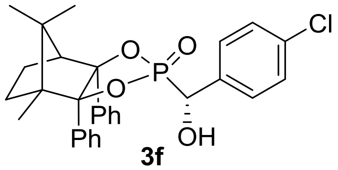

3f

(3a*S*,4*S*,7*S*,7a*S*)-2-((*R*)-(4-Chlorophenyl)(hydroxy)methyl)-4,8,8-trimethyl-3a,7a-diphenyl-hexahydr-o-4,7-methanobenzo[*d*][1,3,2]dioxaphosphole 2-oxide (**3f**).

$[\alpha]_D^{25}$ = +22.99 °(c = 1.00 in MeOH);

The residue was purified by flash chromatography (PE/EtOAc = 2:1) on silica gel to afford the product (158 mg, 78% yield), white solid. R_f = 0.42 (PE/EA = 2:1).

m.p. = 97.0-99.0 ℃

^{1}H NMR (300 MHz, Chloroform-*d*) δ 7.47-7.36 (m, 2H), 7.36-7.15 (m, 8H), 6.98 (d, *J* = 8.2 Hz, 2H), 6.80 (dd, *J* = 8.6, 2.3 Hz, 2H), 4.74 (d, *J* = 9.5 Hz, 1H), 3.04 (d, *J* = 5.2 Hz, 1H), 2.09 (dt, *J* = 12.6, 6.4 Hz, 1H), 1.82 (s, 3H), 1.54 (h, *J* = 8.2 Hz, 2H), 1.38 (dq, *J* = 12.9, 7.0, 4.6 Hz, 1H), 1.19 (s, 3H), 1.01 (s, 3H).

^{13}C NMR (75 MHz, Chloroform-*d*) δ 138.09, 136.27, 135.27, 133.72, 130.53, 130.24, 128.64, 128.41 (d, *J* = 6.1 Hz), 128.24, 127.83, 126.65, 100.66, 99.89, 72.52, 70.61, 60.47, 55.92, 52.24, 48.40, 29.57, 26.56, 24.46, 21.53, 14.27, 10.05.

^{31}P NMR (121 MHz, Chloroform-*d*) δ 34.82.

HRMS (ESI-MS) calculated for $C_{29}H_{30}ClO_4PNa$ $[M+Na]^+$: 531.1463, found: 531.1468.

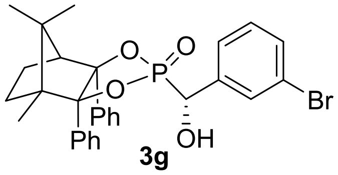

3g

(3a*S*,4*S*,7*S*,7a*S*)-2-((*R*)-(3-Bromophenyl)(hydroxy)methyl)-4,8,8-trimethyl-3a,7a-dipheny-lhexahydr-o-4,7-methanobenzo[*d*][1,3,2]dioxaphosphole 2-oxide (3g).

$[\alpha]_D^{25}$ = +8.00 °(c = 1.00 in MeOH);

The residue was purified by flash chromatography (PE/EtOAc = 2:1) on silica gel to afford the product (172 mg, 78% yield), white solid. R_f = 0.38 (PE/EA = 2:1).

m.p. = 145.0-146.5 ℃

^{1}H NMR (300 MHz, Chloroform-*d*) δ 7.31 (td, *J* = 28.7, 27.8, 12.2 Hz, 12H), 7.08-6.87 (m, 2H), 4.77 (d, *J* = 8.0 Hz, 1H), 3.51 (s, 1H), 3.04 (d, *J* = 5.4 Hz, 1H), 2.16-1.99 (m, 1H), 1.69 (s, 3H), 1.62-1.48 (m, 1H), 1.33-1.19 (m, 2H), 1.13 (s, 3H), 0.81 (s, 3H).

^{13}C NMR (75 MHz, Chloroform-*d*) δ 138.90, 138.02 (d, *J* = 3.4 Hz), 135.97 (d, *J* = 4.8 Hz), 131.64, 131.08 (d, *J* = 7.3 Hz), 130.29, 128.68, 128.24, 127.96, 126.75 (d, *J* = 5.7 Hz), 126.41, 122.37, 102.13, 99.50, 73.13, 71.22, 55.67 (d, *J* = 2.8 Hz), 52.08, 48.16, 29.85, 26.52, 24.53, 21.50, 9.86.

^{31}P NMR (121 MHz, Chloroform-*d*) δ 35.72.

HRMS (ESI-MS) calculated for $C_{29}H_{30}BrO_4PNa$ $[M+Na]^+$: 575.0958, found: 575.0956.

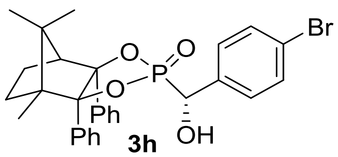

3h

(3a*S*,4*S*,7*S*,7a*S*)-2-((*R*)-(4-Bromophenyl)(hydroxy)methyl)-4,8,8-trimethyl-3a,7a-dipheny-lhexahyd-ro-4,7-methanobenzo[*d*][1,3,2]dioxaphosphole 2-oxide (**3h**).

$[\alpha]_D^{25}$ = +24.00 °(c = 1.00 in MeOH);

The residue was purified by flash chromatography (PE/EtOAc = 2:1) on silica gel to afford the product (168 mg, 76% yield), white solid. R_f = 0.45 (PE/EA = 2:1).

m.p. = 126.0-128.0 ℃

^{1}H NMR (300 MHz, Chloroform-*d*) δ 77.50-7.19 (m, 10H), 7.14 (d, *J* = 8.1 Hz, 2H), 6.79-6.70 (m, 2H), 4.71 (d, *J* = 9.8 Hz, 1H), 3.04 (d, *J* = 5.3 Hz, 1H), 2.09 (td, *J* = 12.0, 11.1, 5.7 Hz, 1H), 1.80 (s, 3H), 1.53 (dq, *J* = 15.1, 8.4, 6.9 Hz, 2H), 1.43-1.32 (m, 1H), 1.19 (s, 3H), 0.99 (s, 3H).

^{13}C NMR (75 MHz, Chloroform-*d*) δ 138.10 (d, *J* = 3.4 Hz), 136.31 (d, *J* = 4.1 Hz), 135.77, 131.25 (d, *J* = 2.3 Hz), 130.56, 130.26, 128.79, 128.69 (d, *J* = 2.1 Hz), 128.27, 127.88, 126.69, 122.09 (d, *J* = 3.7 Hz), 100.68, 99.92, 72.61, 70.69, 55.95 (d, *J* = 3.0 Hz), 52.29, 48.45, 29.79, 29.61, 26.58, 24.49, 21.57, 10.08.

^{31}P NMR (121 MHz, Chloroform-*d*) δ 34.60.

HRMS (ESI-MS) calculated for $C_{29}H_{30}BrO_4PNa$ [M+Na]$^+$: 575.0958, found: 575.0956.

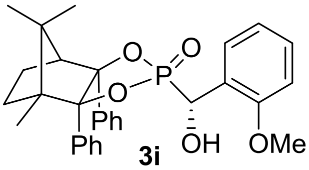

3i

(3a*S*,4*S*,7*S*,7a*S*)-2-((*R*)-Hydroxy(2-methoxyphenyl)methyl)-4,8,8-trimethyl-3a,7a-dipheny-lhexahyd-ro-4,7-methanobenzo[*d*][1,3,2]dioxaphosphole 2-oxide (**3i**).

$[\alpha]_D^{25}$ = +11.97 °(c = 1.00 in MeOH);

The residue was purified by flash chromatography (PE/EtOAc = 2:1) on silica gel to afford the product (131 mg, 65% yield), white solid. R_f = 0.40 (PE/EA = 2:1).

m.p. = 119.3-121.3 ℃

^{1}H NMR (300 MHz, Chloroform-*d*) δ 7.81 (d, *J* = 8.3 Hz, 1H), 7.38-7.27 (m, 5H), 7.25-7.07 (m, 5H), 6.95-6.61 (m, 3H), 4.86 (d, *J* = 9.6 Hz, 1H), 3.84 (s, 3H), 3.01 (d, *J* = 5.3 Hz, 1H), 2.43 (s, 1H), 2.08 (ddt, *J* = 15.3, 10.4, 5.3 Hz, 1H), 1.79 (s, 3H), 1.59 (dt, *J* = 14.6, 6.9 Hz, 1H), 1.39-1.31 (m, 2H), 1.16 (s, 3H), 0.93 (s, 3H).

^{13}C NMR (75 MHz, Chloroform-*d*) δ 156.47 (d, *J* = 8.0 Hz), 137.97 (d, *J* = 3.2 Hz), 136.33 (d, *J* = 4.7 Hz), 131.98 – 129.19 (m), 128.50, 127.54 (d, *J* = 27.0 Hz), 125.78 (d, *J* = 2.0 Hz), 121.19 (d, *J* = 2.2 Hz), 110.61, 101.31, 99.38 (d, *J* = 2.2 Hz), 66.94, 65.04, 55.51 (d, *J* = 2.6 Hz), 55.06, 51.84 (d, *J* = 3.3 Hz), 48.25, 29.95, 26.56, 24.38, 21.68, 9.73.

^{31}P NMR (121 MHz, Chloroform-*d*) δ 37.24.

HRMS (ESI-MS) calculated for $C_{30}H_{34}O_5P$ [M+H]$^+$: 505.2144, found: 505.2148.

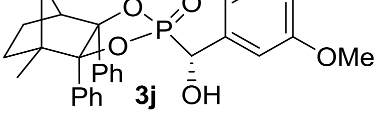

3j

(3a*S*,4*S*,7*S*,7a*S*)-2-((*R*)-Hydroxy(3-methoxyphenyl)methyl)-4,8,8-trimethyl-3a,7a-dipheny-lhexahyd-ro-4,7-methanobenzo[*d*][1,3,2]dioxaphosphole 2-oxide (**3j**).

$[\alpha]_D^{25}$ = +21.96 °(c = 1.00 in MeOH);

The residue was purified by flash chromatography (PE/EtOAc = 2:1) on silica gel to afford the product (153 mg, 76% yield), white solid. R_f = 0.40 (PE/EA = 2:1).

m.p. = 118.5-120.0 ℃

^{1}H NMR (300 MHz, Chloroform-*d*) δ 7.47-7.03 (m, 10H), 6.94-6.82 (m, 4H), 4.76 (d, *J* = 7.3 Hz, 1H), 3.75 (s, 3H), 3.06 (d, *J* = 5.4 Hz, 1H), 2.51 (s, 1H), 2.15-1.99 (m, 1H), 1.75 (s, 3H), 1.56 (dt, *J* = 14.5, 6.8 Hz, 1H), 1.31-1.25 (m, 2H), 1.15 (s, 3H), 0.80 (s, 3H).

^{13}C NMR (75 MHz, Chloroform-*d*) δ 159.77 (d, *J* = 1.7 Hz), 138.12 (d, *J* = 3.2 Hz), 137.95, 135.98 (d, *J* = 4.7 Hz), 130.29 (d, *J* = 8.5 Hz), 129.67 (d, *J* = 1.9 Hz), 128.64, 127.97 (d, *J* = 5.7 Hz), 126.22, 120.54 (d, *J* = 7.0 Hz), 115.01 (d, *J* = 2.6 Hz), 113.08 (d, *J* = 6.8 Hz), 101.80, 99.29 (d, *J* = 1.9 Hz), 73.75, 71.86, 55.63 (d, *J* = 2.7 Hz), 55.31, 51.99 (d, *J* = 3.3 Hz), 48.23, 29.83, 26.54, 24.51, 21.59, 9.85.

^{31}P NMR (121 MHz, Chloroform-*d*) δ 36.58.

HRMS (ESI-MS) calculated for $C_{30}H_{34}O_5P$ $[M+H]^+$: 505.2144, found: 505.2145.

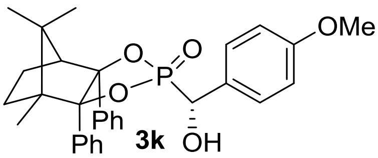

3k

(3a*S*,4*S*,7*S*,7a*S*)-2-((*R*)-Hydroxy(4-methoxyphenyl)methyl)-4,8,8-trimethyl-3a,7a-dipheny-lhexahyd-ro-4,7-methanobenzo[*d*][1,3,2]dioxaphosphole 2-oxide (**3k**).

$[\alpha]_D^{25}$ = +33.99 °(c = 1.00 in MeOH);

The residue was purified by flash chromatography (PE/EtOAc = 2:1) on silica gel to afford the product (173 mg, 86% yield), white solid. R_f = 0.42 (PE/EA = 2:1).

m.p. = 105.0-107.0 ℃

^{1}H NMR (300 MHz, Chloroform-*d*) δ 7.35-7.21 (m, 8H), 7.12 (t, *J* = 7.7 Hz, 2H), 6.88 (t, *J* = 7.6 Hz, 4H), 4.71 (d, *J* = 6.7 Hz, 1H), 3.83 (s, 3H), 3.06 (d, *J* = 5.5 Hz, 1H), 2.07 (ddd, *J* = 13.9, 8.0, 4.2 Hz, 1H), 1.75 (s, 3H), 1.63-1.46 (m, 1H), 1.32-1.24 (m, 2H), 1.14 (s, 3H), 0.78 (s, 3H).

^{13}C NMR (75 MHz, Chloroform-*d*) δ 160.05 (d, *J* = 2.4 Hz), 138.20 (d, *J* = 3.2 Hz), 136.01 (d, *J* = 4.6 Hz), 130.28, 129.77 (d, *J* = 7.2 Hz), 128.63 (d, *J* = 3.3 Hz), 127.97 (d, *J* = 5.6 Hz), 126.18, 114.08 (d, *J* = 1.5 Hz), 101.60, 99.16 (d, J = 1.9 Hz), 73.36, 71.44, 55.61 (d, *J* = 2.8 Hz), 55.46, 52.01 (d, *J* = 3.3 Hz), 48.25, 29.79, 26.51, 24.51, 21.59, 9.85.

^{31}P NMR (121 MHz, Chloroform-*d*) δ 36.87.

HRMS (ESI-MS) calculated for $C_{30}H_{33}O_5PNa$ $[M+Na]^+$: 527.1958, found 527.1957.

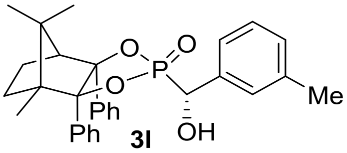

3l

(3a*S*,4*S*,7*S*,7a*S*)-2-((*R*)-Hydroxy(m-tolyl)methyl)-4,8,8-trimethyl-3a,7a-diphenylhexahy-dro-4,7-met-hanobenzo[*d*][1,3,2]dioxaphosphole 2-oxide (3l).

$[\alpha]_D^{25}$ = +1.99 °(c = 1.00 in MeOH);

The residue was purified by flash chromatography (PE/EtOAc = 2:1) on silica gel to afford the product (146 mg, 75% yield), white solid. R_f = 0.45 (PE/EA = 2:1).

m.p. = 120.9-121.9 ℃

^{1}H NMR (300 MHz, Chloroform-*d*) δ 7.39-6.69 (m, 14H), 4.67 (d, *J* = 7.5 Hz, 1H), 3.05 (s, 1H), 2.97 (d, *J* = 5.3 Hz, 1H), 2.24 (s, 3H), 2.05-1.91 (m, 1H), 1.67 (s, 3H), 1.55-1.36 (m, 1H), 1.19 (t, *J* = 7.6 Hz, 2H), 1.06 (s, 3H), 0.71 (s, 3H).

^{13}C NMR (75 MHz, Chloroform-*d*) δ 138.11 (d, *J* = 3.3 Hz), 136.47, 136.03 (d, *J* = 4.7 Hz), 130.31 (d, *J* = 6.4 Hz), 129.46 (d, *J* = 2.7 Hz), 128.85 (d, *J* = 7.0 Hz), 128.60-128.36 (m), 127.88 (d, *J* = 4.7 Hz), 126.12, 125.32 (d, *J* = 6.9 Hz), 101.71, 99.19 (d, *J* = 2.0 Hz), 73.78, 71.89, 55.60 (d, *J* = 2.7 Hz), 52.01 (d, *J* = 3.3 Hz), 48.19, 29.84, 26.51, 24.47, 21.53 (d, *J* = 5.8 Hz), 9.81.

^{31}P NMR (121 MHz, Chloroform-*d*) δ 36.92.

HRMS (ESI-MS) calculated for $C_{30}H_{33}O_4PNa$ $[M+Na]^+$: 511.2009, found: 511.2013.

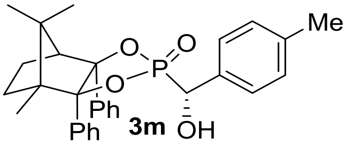

3m

(3a*S*,4*S*,7*S*,7a*S*)-2-((*R*)-Hydroxy(p-tolyl)methyl)-4,8,8-trimethyl-3a,7a-diphenylhexahy-dro-4,7-met-hanobenzo[*d*][1,3,2]dioxaphosphole 2-oxide (**3m**).

$[\alpha]_D^{25}$ = +16.00 °(c = 1.00 in MeOH);

The residue was purified by flash chromatography (PE/EtOAc = 2:1) on silica gel to afford the product (164 mg, 84% yield), white solid. R_f = 0.45 (PE/EA = 2:1).

m.p. = 107.0-109.0 ℃

^{1}H NMR (300 MHz, Chloroform-*d*) δ 7.37 - 7.02 (m, 12H), 6.94 - 6.82 (m, 2H), 4.73 (d, *J* = 7.0 Hz, 1H), 3.73 (t, *J* = 6.1 Hz, 1H), 3.05 (d, *J* = 5.4 Hz, 1H), 2.38 (s, 3H), 2.19 - 1.96 (m, 1H), 1.75 (s, 3H), 1.63 -1.45 (m, 1H), 1.28 (t, *J* = 7.4 Hz, 2H), 1.14 (s, 3H), 0.78 (s, 3H).

^{13}C NMR (75 MHz, Chloroform-*d*) δ 138.59 (d, *J* = 2.7 Hz), 138.22 (d, *J* = 3.4 Hz), 136.07 (d, *J* = 4.7 Hz), 133.60, 130.31, 129.35 (d, *J* = 1.8 Hz), 128.59, 128.29, 128.20, 127.97, 127.91, 126.17, 101.65, 99.17 (d, *J* = 2.1 Hz), 73.68, 71.78, 68.07, 55.63 (d, *J* = 2.7 Hz), 52.05 (d, *J* = 3.2 Hz), 48.27, 29.84, 26.53, 25.72, 24.52, 21.61, 21.38, 9.85.

^{31}P NMR (121 MHz, Chloroform-*d*) δ 36.90.

HRMS (ESI-MS) calculated for $C_{30}H_{33}O_4PNa$ $[M+Na]^+$: 511.2009, found: 511.2010.

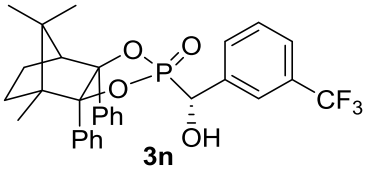

3n

(3a*S*,4*S*,7*S*,7a*S*)-2-((*R*)-Hydroxy(3-(trifluoromethyl)phenyl)methyl)-4,8,8-trimethyl-3a,7a-diphenyl-hexahydro-4,7-methanobenzo[*d*][1,3,2]dioxaphosphole 2-oxide (**3n**).

$[\alpha]_D^{25}$ = +6.97 °(c = 1.00 in MeOH);

The residue was purified by flash chromatography (PE/EtOAc = 2:1) on silica gel to afford the product (195 mg, 90% yield), white solid. R_f = 0.35 (PE/EA = 2:1).

m.p. = 134.0-135.5 ℃

^{1}H NMR (300 MHz, Chloroform-*d*) δ 7.73-6.95 (m, 14H), 4.89 (d, *J* = 7.2 Hz, 1H), 3.06 (d, *J* = 5.4 Hz, 1H), 2.10 (dq, *J* = 14.7, 4.9 Hz, 1H), 1.70 (s, 3H), 1.58 (ddd, *J* = 14.6, 8.4, 6.2 Hz, 1H), 1.28 (ddd, *J* = 8.6, 5.8, 3.0 Hz, 2H), 1.14 (s, 3H), 0.82 (s, 3H).

^{13}C NMR (75 MHz, Chloroform-*d*) δ 138.14 (d, *J* = 3.3 Hz), 137.49, 136.07 (d, *J* = 4.6 Hz), 131.63 (d, *J* = 5.3 Hz), 130.43, 130.21, 130.05, 129.24 (d, *J* = 2.3 Hz), 128.78, 128.37, 128.07, 126.48, 125.71-125.16 (m), 124.81 (dd, *J* = 7.4, 3.8 Hz), 122.29, 102.23, 99.55 (d, *J* = 1.8 Hz), 73.30, 71.39, 55.72 (d, *J* = 2.7 Hz), 52.07 (d, *J* = 3.3 Hz), 48.18, 29.82, 26.51, 24.58, 21.49, 9.89.

^{19}F NMR (282 MHz, Chloroform-*d*) δ -62.49.

^{31}P NMR (121 MHz, Chloroform-*d*) δ 35.02.

HRMS (ESI-MS) calculated for $C_{30}H_{31}F_3O_4P$ $[M+H]^+$: 543.1907, found: 543.1906.

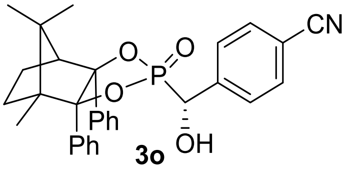

3o

4-((1*R*)-Hydroxy((3a*S*,4*S*,7*S*,7a*S*)-4,8,8-trimethyl-2-oxido-3a,7a-diphenylhexahydro-4,7-methanobe-nzo[*d*][1,3,2]dioxaphosphol-2-yl)methyl)benzonitrile (**3o**).

$[\alpha]_D^{25}$ = +6.98 °(c = 1.00 in MeOH);

The residue was purified by flash chromatography (PE/EtOAc = 2:1) on silica gel to afford the product (180 mg, 90% yield), white solid. R_f = 0.40 (PE/EA = 2:1).

m.p. = 117.5-109.0 ℃

^{1}H NMR (300 MHz, Chloroform-*d*) δ 7.66-6.90 (m, 14H), 4.92 (d, *J* = 8.5 Hz, 1H), 3.05 (d, *J* = 5.4 Hz, 1H), 2.19-2.01 (m, 1H), 1.69 (s, 3H), 1.57 (ddd, *J* = 14.6, 8.8, 5.9 Hz, 1H), 1.32-1.23 (m, 2H), 1.15 (s, 3H), 0.82 (s, 3H).

^{13}C NMR (75 MHz, Chloroform-*d*) δ 141.82, 137.95 (d, *J* = 3.3 Hz), 136.05 (d, *J* = 4.7 Hz), 132.25 (d, *J* = 2.3 Hz), 130.24, 130.09, 128.83, 128.62-128.36 (m), 128.06, 126.45, 118.76 (d, *J* = 1.8 Hz), 112.24 (d, *J* = 3.1 Hz), 102.40, 99.77 (d, *J* = 1.9 Hz), 73.19, 71.30, 55.73 (d, *J* = 2.7 Hz), 52.02 (d, *J* = 3.3 Hz), 48.14, 29.81, 26.52, 24.55, 21.44, 9.86.

^{31}P NMR (121 MHz, Chloroform-*d*) δ 34.83.

HRMS (ESI-MS) calculated for $C_{30}H_{31}NO_4P$ $[M+H]^+$: 500.1986, found: 500.1979.

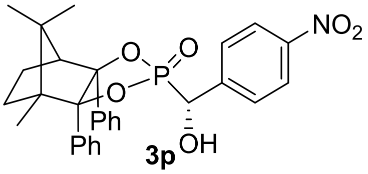

3p

(3a*S*,4*S*,7*S*,7a*S*)-2-((*R*)-Hydroxy(4-nitrophenyl)methyl)-4,8,8-trimethyl-3a,7a-diphenyl-hexahydro-4,7-methanobenzo[*d*][1,3,2]dioxaphosphole 2-oxide (**3p**).

$[\alpha]_D^{25}$ = +7.99 °(c = 1.00 in MeOH);

The residue was purified by flash chromatography (PE/EtOAc = 2:1) on silica gel to afford the product (185 mg, 89% yield), white solid. R_f = 0.32 (PE/EA = 2:1).

m.p. = 116.0-117.5 ℃

^{1}H NMR (300 MHz, Chloroform-*d*) δ 8.16 (d, *J* = 8.4 Hz, 2H), 7.50 (dd, *J* = 8.8, 2.2 Hz, 2H), 7.40-7.10 (m, 9H), 7.02 (s, 1H), 4.99 (d, *J* = 8.7 Hz, 1H), 3.05 (d, *J* = 5.4 Hz, 1H), 2.11 (ddd, *J* = 14.4, 10.2, 5.1 Hz, 1H), 1.69 (s, 3H), 1.57 (ddd, *J* = 14.6, 8.8, 6.0 Hz, 1H), 1.28 (dq, *J* = 8.6, 4.9, 4.3 Hz, 2H), 1.15 (s, 3H), 0.83 (s, 3H).

^{13}C NMR (75 MHz, Chloroform-*d*) δ 147.90 (d, *J* = 3.4 Hz), 143.71, 137.93 (d, *J* = 3.5 Hz), 136.06 (d, *J* = 4.8 Hz), 130.24, 128.88, 128.74-128.45 (m), 128.09, 126.50, 123.64 (d, *J* = 2.3 Hz), 102.55, 99.89 (d, *J* = 1.9 Hz), 73.04, 71.15, 55.77 (d, *J* = 2.7 Hz), 52.05 (d, J = 3.3 Hz), 48.13, 29.83, 26.54, 24.57, 21.44, 9.87.

^{31}P NMR (121 MHz, Chloroform-*d*) δ 34.43.

HRMS (ESI-MS) calculated for $C_{29}H_{31}NO_6P$ $[M+H]^+$: 520.1884, found: 520.1879.

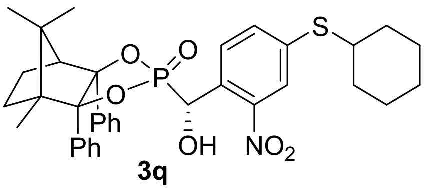

3q

(3a*S*,4*S*,7*S*,7a*S*)-2-((*R*)-(4-(Cyclohexylthio)-2-nitrophenyl)(hydroxy)methyl)-4,8,8-trimethyl-3a,7a-d-iphenylhexahydro-4,7-methanobenzo[*d*][1,3,2]dioxaphosphole 2-oxide (**3q**).

$[\alpha]_D^{25}$ = +32.00 °(c = 1.00 in MeOH);

The residue was purified by flash chromatography (PE/EtOAc = 3:1) on silica gel to afford the product (165 mg, 65% yield), white solid. R_f = 0.35 (PE/EA = 3:1).

m.p. = 121.5-123.5 ℃

^{1}H NMR (300 MHz, Chloroform-*d*) δ 8.44 (t, *J* = 2.2 Hz, 1H), 8.03 (dd, *J* = 9.1, 2.5 Hz, 1H), 7.43-7.33 (m, 3H), 7.26 (dd, *J* = 10.4, 7.0 Hz, 3H), 7.14 (d, *J* = 7.3 Hz, 2H), 6.99 (t, *J* = 7.7 Hz, 2H), 6.78 (d, *J* = 7.9 Hz, 2H), 5.41 (d, *J* = 9.3 Hz, 1H), 3.22 (s, 1H), 3.07 (qd, *J* = 6.8, 4.7, 3.6 Hz, 1H), 2.99 (s, 1H), 2.01 (ddd, *J* = 19.0, 9.5, 5.1 Hz, 1H), 1.90-1.81 (m, 1H), 1.80-1.71 (m, 1H), 1.68 (s, 3H), 1.50 (td, *J* = 14.5, 13.0, 6.6 Hz, 2H), 1.32-1.13 (m, 9H), 1.07 (s, 3H), 0.73 (s, 3H).

^{13}C NMR (75 MHz, Chloroform- *d*) δ 145.95 (d, *J* = 2.2 Hz), 145.66, 145.53, 138.40 (d, *J* = 2.1 Hz), 137.81 (d, *J* = 3.5 Hz), 135.99 (d, *J* = 4.7 Hz), 130.59, 130.26, 129.93, 128.77, 127.88, 126.20, 124.76 (d, *J* = 4.3 Hz), 123.30 (d, *J* = 1.8 Hz), 102.31, 99.90 (d, *J* = 2.1 Hz), 69.83, 67.90, 55.65 (d, *J*

= 2.6 Hz), 52.00 (d, *J* = 3.2 Hz), 48.10, 47.15, 33.10, 31.63, 29.71, 26.55, 25.89 (d, *J* = 6.5 Hz), 25.58, 24.57, 22.71, 21.53, 14.20, 9.84.

^{31}P NMR (121 MHz, Chloroform-*d*) δ 35.54.

HRMS (ESI-MS) calculated for $C_{35}H_{41}NO_6PS$ [M+H]$^+$:634.2387, found: 634.2393.

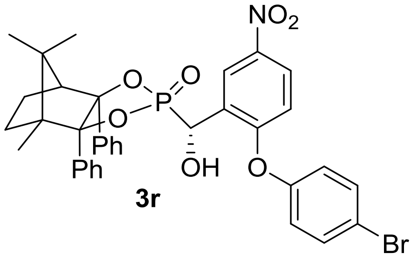

3r

(3a*S*,4*S*,7*S*,7a*S*)-2-((*R*)-(2-(4-Bromophenoxy)-5-nitrophenyl)(hydroxy)methyl)-4,8,8-trimethyl-3a,7a-diphenylhexahydro-4,7-methanobenzo[*d*][1,3,2]dioxaphosphole 2-oxide (**3r**).

$[\alpha]_D^{25}$ = +11.99 °(c = 1.00 in MeOH);

The residue was purified by flash chromatography (PE/EtOAc = 3:1) on silica gel to afford the product (152 mg, 55% yield), white solid. R_f = 0.40 (PE/EA = 3:1).

m.p. = 111.8-123.8 ℃

^{1}H NMR (300 MHz, Chloroform-*d*) δ 8.45 (t, *J* = 2.5 Hz, 1H), 8.12-7.97 (m, 1H), 7.63-7.51 (m, 2H), 7.35-7.11 (m, 6H), 7.09-6.73 (m, 6H), 6.67 (d, *J* = 9.1 Hz, 1H), 5.23 (d, *J* = 9.5 Hz, 1H), 3.07 (d, *J* = 5.3 Hz, 1H), 2.09 (ddd, *J* = 17.8, 8.7, 4.4 Hz, 1H), 1.66 (s, 3H), 1.61-1.56 (m, 1H), 1.28-1.22 (m, 2H), 1.14 (s, 3H), 0.74 (s, 3H).

^{13}C NMR (75 MHz, Chloroform-*d*) δ 159.88, 159.79, 153.48, 143.07 (d, *J* = 2.2 Hz), 136.40 (d, *J* = 4.8 Hz), 133.57, 130.03, 129.16, 128.87, 128.79-128.06 (m), 127.93, 126.20, 125.23 (d, *J* = 8.5 Hz), 122.76, 118.90, 115.29, 101.87, 99.60 (d, *J* = 1.9 Hz), 68.29, 66.39, 55.65 (d, *J* = 2.7 Hz), 51.84 (d, *J* = 3.3 Hz), 48.23, 29.82, 26.54, 24.49, 21.47, 9.73.

^{31}P NMR (121 MHz, Chloroform-*d*) δ 36.19.

HRMS (ESI-MS) calculated for $C_{35}H_{34}BrNO_7P$ [M+H]$^+$: 690.1251, found 690.1252.

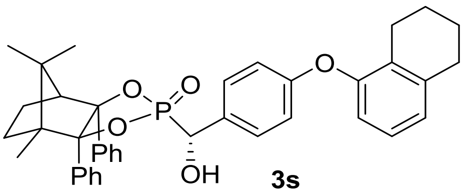

(3a*S*,4*S*,7*S*,7a*S*)-2-((*R*)-Hydroxy(4-((5,6,7,8-tetrahydronaphthalen-1-yl)oxy)phenyl)methyl)-4,8,8-trimethyl-3a,7a-diphenylhexahydro-4,7-methanobenzo[*d*][1,3,2]dioxaphosphole 2-oxide (**3s**).

$[\alpha]_D^{25}$ = +47.97 °(c = 1.00 in MeOH);

The residue was purified by flash chromatography (PE/EtOAc = 3:1) on silica gel to afford the product (149 mg, 60% yield), white solid. R_f = 0.36 (PE/EA = 3:1).

m.p. = 105.0-107.0 ℃

^{1}H NMR (300 MHz, Chloroform-*d*) δ 8.20-5.96 (m, 17H), 4.66 (d, *J* = 6.6 Hz, 1H), 2.99 (d, *J* = 5.3 Hz, 1H), 2.75 (d, *J* = 5.1 Hz, 2H), 2.61 (d, *J* = 5.8 Hz, 2H), 2.00 (ddd, *J* = 14.5, 8.6, 5.5 Hz, 1H), 1.84-1.57 (m, 6H), 1.56-1.41 (m, 1H), 1.35-1.14 (m, 3H), 1.08 (s, 3H), 0.73 (s, 3H).

^{13}C NMR (75 MHz, Chloroform-*d*) δ 158.51 (d, *J* = 2.5 Hz), 153.90, 139.68, 138.17 (d, *J* = 3.3 Hz), 136.03 (d, *J* = 4.6 Hz), 130.26, 130.14, 129.84 (d, *J* = 7.0 Hz), 129.63, 128.66, 128.04 (d, *J* = 9.6 Hz), 126.22, 125.23, 117.25, 117.23, 117.06, 101.73, 99.24 (d, *J* = 1.9 Hz), 73.35, 71.43, 55.66 (d, *J* = 2.7 Hz), 52.03 (d, *J* = 3.2 Hz), 48.27, 29.74 (d, *J* = 10.7 Hz), 26.52, 24.51, 23.47, 22.83 (d, *J* = 14.7 Hz), 21.60, 9.89.

^{31}P NMR (121 MHz, Chloroform-*d*) δ 36.59.

HRMS (ESI-MS) calculated for $C_{39}H_{42}O_5P$ $[M+H]^+$: 621.2764, found: 621.2765.

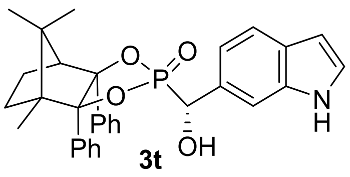

(3a*S*,4*S*,7*S*,7a*S*)-2-((*R*)-Hydroxy(1H-indol-6-yl)methyl)-4,8,8-trimethyl-3a,7a-diphenylhexahydro-4,7-methanobenzo[*d*][1,3,2]dioxaphosphole 2-oxide (**3t**).

$[\alpha]_D^{25}$ = +25.00 °(c = 1.00 in MeOH);

The residue was purified by flash chromatography (PE/EtOAc = 2:1) on silica gel to afford the product (164 mg, 80% yield), white solid. R_f = 0.35 (PE/EA = 2:1).

m.p. = 104.9-106.9 ℃

^{1}H NMR (300 MHz, Chloroform-*d*) δ 8.57 (s, 1H), 7.44 (d, *J* = 2.0 Hz, 1H), 7.38-7.15 (m, 9H), 7.00 (t, *J* = 7.7 Hz, 2H), 6.80 (d, *J* = 7.8 Hz, 2H), 6.49 (t, *J* = 2.7 Hz, 1H), 4.85 (d, *J* = 6.6 Hz, 1H), 3.07 (d, *J* = 5.3 Hz, 1H), 2.07 (tdd, *J* = 11.4, 9.4, 8.1, 4.1 Hz, 1H), 1.78 (s, 3H), 1.56 (dt, *J* = 14.1, 7.3 Hz, 1H), 1.31-1.24 (m, 2H), 1.14 (s, 3H), 0.73 (s, 3H).

^{13}C NMR (75 MHz, Chloroform-*d*) δ 138.26 (d, *J* = 3.2 Hz), 136.24, 135.84 (d, *J* = 4.6 Hz), 130.40, 128.59, 127.94, 127.86, 127.67, 126.04, 125.06, 122.12 (d, J = 5.9 Hz), 121.26 (d, *J* = 8.9 Hz), 111.67, 102.95, 101.56, 99.10 (d, *J* = 2.0 Hz), 74.53, 72.61, 55.60 (d, *J* = 2.8 Hz), 52.04 (d, *J* = 3.1 Hz), 48.29, 29.81, 26.53, 24.51, 21.65, 9.85.

^{31}P NMR (121 MHz, Chloroform-*d*) δ 37.73.

HRMS (ESI-MS) calculated for $C_{31}H_{32}NO_4PNa$ $[M+Na]^+$: 536.1962, found: 536.1962.

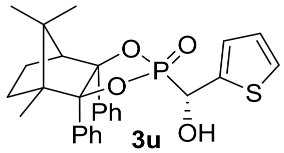

3u

(3a*S*,4*S*,7*S*,7a*S*)-2-((*R*)-Hydroxy(thiophen-2-yl)methyl)-4,8,8-trimethyl-3a,7a-diphenyl-hexahydro-4,7-methanobenzo[*d*][1,3,2]dioxaphosphole 2-oxide (**3u**).

$[\alpha]_D^{25}$ = +12.00 °(c = 1.00 in MeOH);

The residue was purified by flash chromatography (PE/EtOAc = 3:1) on silica gel to afford the product (146 mg, 76% yield), white solid. R_f = 0.40 (PE/EA = 3:1).

m.p. = 155.8-157.8 ℃

^{1}H NMR (300 MHz, Chloroform-*d*) δ 7.36 – 7.21 (m, 7H), 7.16 – 7.07 (m, 3H), 7.03 – 6.86 (m, 3H), 4.97 (d, *J* = 8.7 Hz, 1H), 3.07 (d, *J* = 5.4 Hz, 1H), 2.69 (s, 1H), 2.17 – 1.97 (m, 1H), 1.76 (s, 3H), 1.64 – 1.49 (m, 1H), 1.34 – 1.26 (m, 2H), 1.16 (s, 3H), 0.81 (s, 3H).

^{13}C NMR (75 MHz, Chloroform-*d*) δ 138.80, 138.00 (d, *J* = 3.3 Hz), 135.88 (d, *J* = 4.6 Hz), 130.14 (d, *J* = 12.1 Hz), 128.63, 128.16 – 127.80 (m), 127.03, 126.27, 101.75, 99.39 (d, *J* = 1.8 Hz), 69.09, 67.08, 55.66 (d, *J* = 2.9 Hz), 52.04 (d, *J* = 3.3 Hz), 48.30, 29.70, 26.48, 24.52, 21.66, 9.89.

^{31}P NMR (121 MHz, Chloroform-*d*) δ 34.85.

HRMS (ESI-MS) calculated for $C_{27}H_{29}O_4PSNa$ $[M+Na]^+$: 503.1417, found: 503.1416.

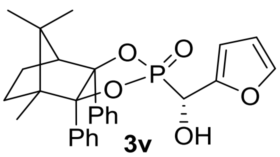

3v

(3a*S*,4*S*,7*S*,7a*S*)-2-((*R*)-Furan-2-yl(hydroxy)methyl)-4,8,8-trimethyl-3a,7a-diphenylhexa-hydro-4,7-methanobenzo[*d*][1,3,2]dioxaphosphole 2-oxide (**3v**).

$[\alpha]_D^{25}$ = +90.98 °(c = 1.00 in MeOH);

The residue was purified by flash chromatography (PE/EtOAc = 3:1) on silica gel to afford the product (152 mg, 82% yield), white solid. R_f = 0.42 (PE/EA = 3:1).

m.p. = 108.0-109.0 ℃

^{1}H NMR (300 MHz, Chloroform-*d*) δ 7.49-7.01 (m, 11H), 6.89 (t, *J* = 2.1 Hz, 1H), 5.91 (d, *J* = 1.8 Hz, 1H), 4.65 (d, *J* = 8.3 Hz, 1H), 3.86 (s, 1H), 3.04 (d, *J* = 5.4 Hz, 1H), 2.16-2.04 (m, 1H), 1.79 (s, 3H), 1.62-1.50 (m, 1H), 1.48-1.29 (m, 2H), 1.18 (s, 3H), 0.94 (s, 3H).

^{13}C NMR (75 MHz, Chloroform-*d*) δ 142.89, 140.79 (d, *J* = 10.5 Hz), 138.16 (d, *J* = 3.3 Hz), 136.20 (d, *J* = 4.0 Hz), 130.21 (d, *J* = 13.0 Hz), 128.40 (d, *J* = 24.3 Hz), 127.81, 126.73, 121.22, 109.21 (d, *J* = 4.7 Hz), 100.55, 99.41, 66.48, 64.50, 55.86 (d, *J* = 3.2 Hz), 52.13 (d, *J* = 3.2 Hz), 48.47, 29.51, 26.46, 24.55, 21.70, 10.09.

^{31}P NMR (121 MHz, Chloroform-*d*) δ 35.10.

HRMS (ESI-MS) calculated for $C_{27}H_{30}O_5P$ $[M+H]^+$: 465.1826, found: 465.1819.

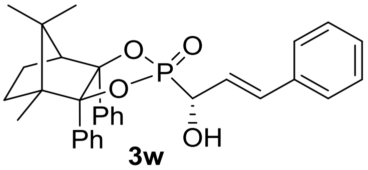

3w

(3a*S*,4*S*,7*S*,7a*S*)-2-((*R,E*)-1-Hydroxy-3-phenylallyl)-4,8,8-trimethyl-3a,7a-diphenylhexa-hydro-4,7-methanobenzo[*d*][1,3,2]dioxaphosphole 2-oxide (**3w**).

$[\alpha]_D^{25}$ = +63.97 °(c = 1.00 in MeOH);

The residue was purified by flash chromatography (PE/EtOAc = 3:1) on silica gel to afford the product (170 mg, 85% yield), white solid. R_f = 0.35 (PE/EA = 3:1).

m.p. = 113.0-114.0 ℃

^{1}H NMR (300 MHz, Chloroform-*d*) δ 7.45-6.99 (m, 15H), 6.56 (dd, *J* = 15.8, 4.3 Hz, 1H), 6.31 (ddd, *J* = 15.9, 7.3, 5.4 Hz, 1H), 4.45 (t, *J* = 8.0 Hz, 1H), 3.24 (s, 1H), 3.02 (d, *J* = 5.2 Hz, 1H), 2.12-

1.97 (m, 1H), 1.74 (s, 3H), 1.56 (dt, J = 14.8, 7.2 Hz, 1H), 1.27 (t, J = 7.1 Hz, 2H), 1.12 (s, 3H), 0.84 (s, 3H).

^{13}C NMR (75 MHz, Chloroform-*d*) δ 137.97 (d, J = 3.2 Hz), 136.14 (dd, J = 6.4, 3.4 Hz), 133.88 (d, J = 14.1 Hz), 130.25, 128.55, 128.43, 128.09, 127.76, 126.88 (d, J = 1.8 Hz), 126.19, 124.04 (d, J = 3.4 Hz), 101.42, 99.12 (d, J = 1.8 Hz), 72.77, 70.83, 55.50 (d, J = 2.8 Hz), 51.90 (d, J = 3.3 Hz), 48.13, 31.58, 29.65, 26.39, 24.47, 22.66, 21.56, 14.17, 9.88.

^{31}P NMR (121 MHz, Chloroform-*d*) δ 36.79.

HRMS (ESI-MS) calculated for $C_{31}H_{34}O_4P$ $[M+H]^+$: 501.2195, found: 501.2191;

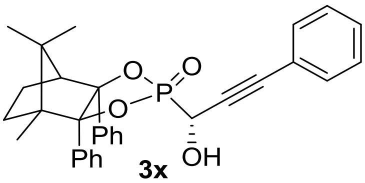

3x

(3a*S*,4*S*,7*S*,7a*S*)-2-((*R*)-1-Hydroxy-3-phenylprop-2-yn-1-yl)-4,8,8-trimethyl-3a,7a-diphenylhexahy-dro-4,7-methanobenzo[*d*][1,3,2]dioxaphosphole 2-oxide (**3x**).

$[\alpha]_D^{25}$ = +3.00 °(c = 1.00 in MeOH);

The residue was purified by flash chromatography (PE/EtOAc = 3:1) on silica gel to afford the product (112 mg, 56% yield), white solid. R_f = 0.40 (PE/EA = 3:1).

m.p. = 118.3-120.3 ℃

^{1}H NMR (300 MHz, Chloroform-*d*) δ 7.47-6.89 (m, 15H), 4.62 (d, J = 14.0 Hz, 1H), 2.99 (d, J = 5.4 Hz, 1H), 2.10-1.95 (m, 1H), 1.72 (s, 3H), 1.57-1.41 (m, 1H), 1.25 (t, J = 7.5 Hz, 2H), 1.10 (s, 3H), 0.82 (s, 3H).

^{13}C NMR (75 MHz, Chloroform-*d*) δ 137.55 (d, J = 3.3 Hz), 135.79 (d, J = 4.6 Hz), 132.17 (d, J = 2.9 Hz), 130.25 (d, J = 5.9 Hz), 129.04, 128.72, 128.38, 128.02 (d, J = 5.2 Hz), 126.24, 122.11, 101.90, 99.85, 88.52 (d, J = 10.4 Hz), 83.47, 62.13, 60.11, 55.76 (d, J = 2.9 Hz), 51.95 (d, J = 3.3 Hz), 48.38, 29.81, 26.50, 24.38, 21.80, 9.93.

^{31}P NMR (121 MHz, Chloroform-*d*) δ 32.94.

HRMS (ESI-MS) calculated for $C_{31}H_{32}O_4P$ $[M+H]^+$: 499.2033, found: 499.2036.

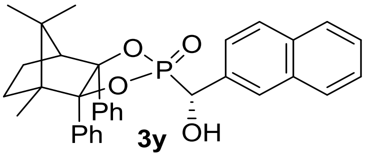

3y

(3a*S*,4*S*,7*S*,7a*S*)-2-((*R*)-Hydroxy(naphthalen-2-yl)methyl)-4,8,8-trimethyl-3a,7a-diphenyl-hexahydro-4,7-methanobenzo[*d*][1,3,2]dioxaphosphole 2-oxide (**3y**).

$[\alpha]_D^{25}$ = +61.00 °(c = 1.00 in MeOH);

The residue was purified by flash chromatography (PE/EtOAc = 2:1) on silica gel to afford the product (184 mg, 88% yield), white solid. R_f = 0.37 (PE/EA = 2:1).

m.p. = 115.0-116.0 ℃

^{1}H NMR (300 MHz, Chloroform-*d*) δ 7.80 (t, J = 7.1 Hz, 2H), 7.75-7.66 (m, 1H), 7.64-7.54 (m, 2H), 7.54-7.40 (m, 2H), 7.31 (d, *J* = 4.1 Hz, 5H), 7.21 (t, *J* = 7.4 Hz, 1H), 6.98 (t, *J* = 7.7 Hz, 2H), 6.84 (d, J = 8.0 Hz, 2H), 4.96 (d, *J* = 7.9 Hz, 1H), 3.25 (s, 1H), 3.00 (d, *J* = 5.2 Hz, 1H), 2.03 (dq, *J* = 14.0, 7.4 Hz, 1H), 1.69 (s, 3H), 1.53 (dt, *J* = 14.4, 7.1 Hz, 1H), 1.22 (t, *J* = 7.5 Hz, 2H), 1.08 (s, 3H), 0.72 (s, 3H).

^{13}C NMR (75 MHz, Chloroform-*d*) δ 138.00 (d, *J* = 3.2 Hz), 135.85 (d, *J* = 4.7 Hz), 134.09, 133.41 (d, *J* = 1.9 Hz), 132.97 (d, *J* = 1.6 Hz), 130.35, 130.18, 128.50, 128.42 (d, *J* = 1.7 Hz), 128.20, 127.88, 127.81, 127.72, 127.66, 127.54, 126.33, 126.12, 125.57 (d, *J* = 4.8 Hz), 101.72, 99.18 (d, *J* = 2.0 Hz), 73.90, 71.99, 55.48 (d, *J* = 2.7 Hz), 51.86 (d, *J* = 3.2 Hz), 48.04, 29.70, 26.42, 24.41, 21.43, 9.73.

^{31}P NMR (121 MHz, Chloroform-*d*) δ 36.92.

HRMS (ESI-MS) calculated for $C_{33}H_{34}O_4P$ $[M+H]^+$: 525.2195, found: 525.2193.

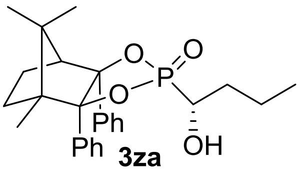

3za

(3a*S*,4*S*,7*S*,7a*S*)-2-((*R*)-1-Hydroxybutyl)-4,8,8-trimethyl-3a,7a-diphenylhexahydro-4,7-met-hanobenzo-[*d*][1,3,2]dioxaphosphole 2-oxide (**3za**).

$[\alpha]_D^{25}$ = +67.99 °(c = 1.00 in MeOH);

The residue was purified by flash chromatography (PE/EtOAc = 4:1) on silica gel to afford the product (123 mg, 70% yield), white solid. R_f = 0.40 (PE/EA = 4:1).

m.p. = 130.0-131.0 ℃

^{1}H NMR (300 MHz, Chloroform-*d*) δ 7.23 (dh, *J* = 16.1, 9.2, 8.0 Hz, 10H), 3.76 (dd, *J* = 9.1, 4.6 Hz, 1H), 3.03 (d, *J* = 5.3 Hz, 1H), 2.36 (s, 1H), 2.08 (ddt, *J* = 14.7, 9.3, 5.6 Hz, 1H), 1.77 (s, 5H), 1.55 (ddd, *J* = 14.4, 11.8, 6.7 Hz, 2H), 1.46-1.35 (m, 1H), 1.29 (dd, *J* = 8.9, 5.3 Hz, 2H), 1.15 (s, 3H), 0.91-0.81 (m, 6H).

^{13}C NMR (75 MHz, Chloroform-*d*) δ 138.25 (d, *J* = 3.3 Hz), 136.48 (d, *J* = 4.5 Hz), 130.06 (d, *J* = 18.3 Hz), 128.39, 128.05, 127.73, 126.31, 101.33, 98.70 (d, *J* = 1.8 Hz), 70.41, 68.50, 55.46 (d, *J* = 2.8 Hz), 51.93 (d, *J* = 3.2 Hz), 48.14, 34.12, 29.68, 26.40, 24.54, 21.54, 18.61, 18.45, 13.69, 9.94.

^{31}P NMR (121 MHz, Chloroform-*d*) δ 40.67.

HRMS (ESI-MS) calculated for $C_{26}H_{34}O_4P$ $[M+H]^+$: 441.2195, found: 441.2196.

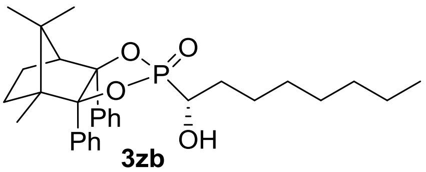

3zb

(3a*S*,4*S*,7*S*,7a*S*)-2-((*R*)-1-Hydroxyoctyl)-4,8,8-trimethyl-3a,7a-diphenylhexahydro-4,7-me-thanoben-zo[*d*][1,3,2]dioxaphosphole 2-oxide (**3zb**).

$[\alpha]_D^{25}$ = +46.00 °(c = 1.00 in MeOH);

The residue was purified by flash chromatography (PE/EtOAc = 4:1) on silica gel to afford the product (159 mg, 80% yield), white solid. R_f = 0.42 (PE/EA = 4:1).

m.p. = 71.0-72.0 ℃

^{1}H NMR (300 MHz, Chloroform-*d*) δ 7.16 (dq, *J* = 12.2, 7.1 Hz, 10H), 3.67 (dd, *J* = 9.3, 4.3 Hz, 1H), 2.95 (d, *J* = 5.2 Hz, 1H), 2.24 (s, 1H), 2.01 (tt, *J* = 9.0, 4.6 Hz, 1H), 1.69 (s, 3H), 1.66-1.42 (m, 3H), 1.31-1.09 (m, 12H), 1.08 (s, 3H), 0.87-0.75 (m, 6H).

^{13}C NMR (75 MHz, Chloroform-*d*) δ 138.25 (d, *J* = 3.2 Hz), 136.53 (d, *J* = 4.5 Hz), 130.21, 129.95, 128.41, 128.05, 127.74, 126.31, 101.35 (d, *J* = 1.3 Hz), 98.72 (d, *J* = 1.8 Hz), 70.77, 68.87, 55.50, 55.46, 51.97, 51.92, 48.16, 32.07 (d, J = 1.8 Hz), 31.82, 29.72, 29.12, 29.01, 26.42, 25.28 (d, *J* = 11.8 Hz), 24.54, 22.66, 21.57, 14.19, 9.95.

^{31}P NMR (121 MHz, Chloroform-*d*) δ 40.68.

HRMS (ESI-MS) calculated for $C_{30}H_{42}O_4P$ $[M+H]^+$: 497.2821, found: 497.2820.

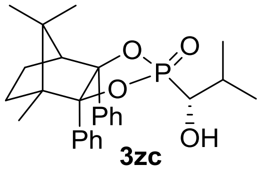

3zc

(3a*S*,4*S*,7*S*,7a*S*)-2-((*R*)-1-Hydroxy-2-methylpropyl)-4,8,8-trimethyl-3a,7a-diphenylhexa-hydro-4,7-methanobenzo[*d*][1,3,2]dioxaphosphole 2-oxide (**3zc**).

$[\alpha]_D^{25}$ = +115.00 °(c = 1.00 in MeOH);

The residue was purified by flash chromatography (PE/EtOAc = 4:1) on silica gel to afford the product (128 mg, 73% yield), white solid. R_f = 0.39 (PE/EA = 4:1).

m.p. = 96.0-97.5 ℃

^{1}H NMR (300 MHz, Chloroform-*d*) δ 7.40-7.06 (m, 10H), 3.45 (d, *J* = 6.6 Hz, 1H), 3.04 (d, *J* = 5.3 Hz, 1H), 2.14-1.96 (m, 2H), 1.79 (s, 3H), 1.57 (ddd, *J* = 14.6, 8.9, 5.9 Hz, 1H), 1.40-1.21 (m, 2H), 1.16 (s, 3H), 1.02 (dd, *J* = 11.2, 6.7 Hz, 6H), 0.90 (s, 3H).

^{13}C NMR (75 MHz, Chloroform-*d*) δ 138.33 (d, *J* = 3.2 Hz), 136.61 (d, *J* = 4.6 Hz), 130.04, 128.55, 128.15, 127.94, 126.33, 101.53 (d, *J* = 1.6 Hz), 98.58 (d, *J* = 1.8 Hz), 76.47, 74.62, 55.55 (d, *J* = 2.6 Hz), 51.89 (d, *J* = 3.3 Hz), 48.22, 30.55 (d, *J* = 2.0 Hz), 29.89, 26.50, 24.51, 21.63, 19.79 (d, *J* = 8.2 Hz), 17.98 (d, *J* = 9.8 Hz), 9.94.

^{31}P NMR (121 MHz, Chloroform-*d*) δ 40.23.

HRMS (ESI-MS) calculated for $C_{26}H_{34}O_4P$ $[M+H]^+$:441.2190, found: 441.2185.

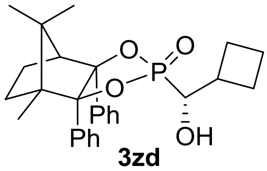

3zd

(3a*S*,4*S*,7*S*,7a*S*)-2-((*R*)-Cyclobutyl(hydroxy)methyl)-4,8,8-trimethyl-3a,7a-diphenylhexa-hydro-4,7-methanobenzo[*d*][1,3,2]dioxaphosphole 2-oxide (**3zd**).

$[\alpha]_D^{25}$ = +62.00 °(c = 1.00 in MeOH);

The residue was purified by flash chromatography (PE/EtOAc = 4:1) on silica gel to afford the product (137 mg, 76% yield), white solid. R_f = 0.40 (PE/EA = 4:1).

m.p. = 153.0-155.0 ℃

^{1}H NMR (300 MHz, Chloroform-*d*) δ 7.34-7.09 (m, 10H), 3.68 (d, *J* = 6.8 Hz, 1H), 3.02 (d, *J* = 5.3 Hz, 1H), 2.70 (hept, *J* = 7.9, 7.3 Hz, 1H), 2.00 (dddd, *J* = 34.4, 26.0, 11.9, 5.0 Hz, 8H), 1.75 (s, 3H), 1.58 (ddd, *J* = 14.5, 8.8, 5.9 Hz, 1H), 1.37-1.23 (m, 2H), 1.15 (s, 3H), 0.89 (s, 3H).

^{13}C NMR (75 MHz, Chloroform-*d*) δ 138.37 (d, *J* = 3.3 Hz), 136.64 (d, *J* = 4.6 Hz), 130.07 (d, *J* = 14.0 Hz), 128.47, 128.12, 127.85, 126.36, 101.52 (d, *J* = 1.6 Hz), 98.76 (d, *J* = 1.9 Hz), 73.84, 71.99, 55.53 (d, J = 2.6 Hz), 51.92 (d, *J* = 3.2 Hz), 48.15, 36.73, 29.81, 26.47, 24.59, 24.54, 24.39, 24.04 (d, *J* = 9.1 Hz), 21.54, 18.45, 9.95.

^{31}P NMR (121 MHz, Chloroform-*d*) δ 39.01.

HRMS (ESI-MS) calculated for $C_{27}H_{34}O_4P$ $[M+H]^+$: 453.2195, found: 453.2195.

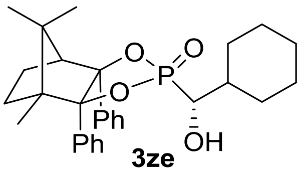

3ze

(3a*S*,4*S*,7*S*,7a*S*)-2-((*R*)-Cyclohexyl(hydroxy)methyl)-4,8,8-trimethyl-3a,7a-diphenylhexa-hydro-4,7-methanobenzo[*d*][1,3,2]dioxaphosphole 2-oxide (**3ze**).

$[\alpha]_D^{25}$ = +45.00 °(c = 1.00 in MeOH);

The residue was purified by flash chromatography (PE/EtOAc = 4:1) on silica gel to afford the product (157 mg, 82% yield), white solid. R_f = 0.35 (PE/EA = 4:1).

m.p. = 96.0-98.0 ℃

^{1}H NMR (300 MHz, Chloroform-*d*) δ 7.35-7.24 (m, 10H), 4.12 (dd, *J* = 7.1 Hz, 1H), 3.42 (dd, *J* = 6.1, 4.1 Hz, 1H), 3.04 (d, *J* = 5.3 Hz, 1H), 2.17-2.07 (m, 1H), 1.79 (s, 3H), 1.74 (s, 1H), 1.57 (p, *J* = 8.2, 7.4 Hz, 2H), 1.39 (qd, *J* = 11.6, 6.9 Hz, 4H), 1.26 (t, *J* = 7.1 Hz, 2H), 1.17 (s, 3H), 0.88 (d, *J* = 8.3 Hz, 8H).

^{13}C NMR (75 MHz, Chloroform-*d*) δ 137.99 (d, *J* = 3.3 Hz), 136.34 (d, *J* = 4.1 Hz), 130.01, 128.38, 128.03, 126.56, 99.88, 99.10 (d, *J* = 1.5 Hz), 75.79, 73.96, 60.48, 55.62 (d, *J* = 3.1 Hz), 51.98

(d, J = 3.0 Hz), 48.38, 39.49 (d, J = 4.3 Hz), 29.54 (t, J = 6.0 Hz), 26.83 (d, J = 6.1 Hz), 26.39, 26.00 (d, J = 1.9 Hz), 25.74 (d, J = 6.5 Hz), 24.48, 21.63, 14.27, 9.99.

^{31}P NMR (121 MHz, Chloroform-*d*) δ 39.98.

HRMS (ESI-MS) calculated for $C_{29}H_{38}O_4P$ [M+H]$^+$: 481.2508, found: 481.2510.

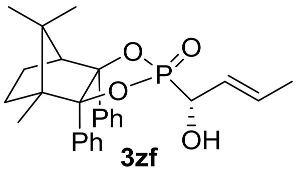

3zf

(3a*S*,4*S*,7*S*,7a*S*)-2-((*R*,*E*)-1-Hydroxybut-2-en-1-yl)-4,8,8-trimethyl-3a,7a-diphenylhexahydro-4,7-methanobenzo[*d*][1,3,2]dioxaphosphole 2-oxide (**3zf**).

$[\alpha]_D^{25}$ = +30.99 °(c = 1.00 in MeOH);

The residue was purified by flash chromatography (PE/EtOAc = 4:1) on silica gel to afford the product (137 mg, 78% yield), white oily liquid. R_f = 0.40 (PE/EA = 4:1).

^{1}H NMR (300 MHz, Chloroform-*d*) δ 7.21 (qd, J = 17.6, 16.5, 9.5 Hz, 10H), 5.79-5.53 (m, 2H), 4.20 (t, J = 6.6 Hz, 1H), 3.02 (d, J = 5.6 Hz, 2H), 2.07 (dq, J = 14.0, 7.2 Hz, 1H), 1.73 (d, J = 8.8 Hz, 6H), 1.56 (dd, J = 14.6, 7.0 Hz, 1H), 1.29 (t, J = 7.7 Hz, 2H), 1.14 (s, 3H), 0.87 (s, 3H).

^{13}C NMR (75 MHz, Chloroform-*d*) δ 138.02 (d, J = 3.3 Hz), 136.07 (d, J = 4.4 Hz), 131.44, 131.25, 130.16, 128.31, 127.99, 127.65, 125.98 (d, J = 5.5 Hz), 101.19, 98.85 (d, J = 1.8 Hz), 72.48, 70.54, 55.42 (d, J = 2.8 Hz), 51.83 (d, J = 3.2 Hz), 48.09, 29.57, 26.36, 24.46, 21.49, 17.98 (d, J = 1.4 Hz), 9.87.

^{31}P NMR (121 MHz, Chloroform-*d*) δ 37.56.

HRMS (ESI-MS) calculated for $C_{26}H_{32}O_4P$ [M+H]$^+$:439.2038, found: 439.2036.

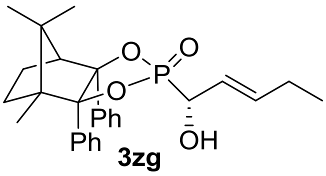

3zg

(3a*S*,4*S*,7*S*,7a*S*)-2-((*R*,*E*)-1-Hydroxypent-2-en-1-yl)-4,8,8-trimethyl-3a,7a-diphenylhexahydro-4,7-methanobenzo[*d*][1,3,2]dioxaphosphole 2-oxide (**3zg**).

$[\alpha]_D^{25}$ = +23.00 °(c = 1.00 in MeOH);

The residue was purified by flash chromatography (PE/EtOAc = 4:1) on silica gel to afford the product (148 mg, 82% yield), white oily liquid. R_f = 0.42 (PE/EA = 4:1).

^{1}H NMR (300 MHz, Chloroform-*d*) δ 7.25-6.98 (m, 10H), 5.65 (dtd, *J* = 15.6, 5.9, 3.7 Hz, 1H), 5.51 (ddd, *J* = 15.3, 7.3, 5.2 Hz, 1H), 4.12 (t, *J* = 7.0 Hz, 1H), 3.03 (s, 1H), 2.94 (d, *J* = 5.2 Hz, 1H), 2.09-1.87 (m, 3H), 1.66 (s, 3H), 1.47 (dd, *J* = 14.6, 7.0 Hz, 1H), 1.20 (t, *J* = 7.6 Hz, 2H), 1.06 (s, 3H), 0.93 (t, *J* = 7.4 Hz, 3H), 0.78 (s, 3H).

^{13}C NMR (75 MHz, Chloroform-*d*) δ 138.16, 138.04, 137.99 (d, *J* = 1.6 Hz), 136.11 (d, J = 4.4 Hz), 130.15, 128.30, 128.00, 127.65, 126.06, 123.64 (d, *J* = 2.7 Hz), 101.19, 98.84 (d, *J* = 1.9 Hz), 72.53, 70.59, 55.43 (d, *J* = 2.9 Hz), 51.84 (d, *J* = 3.1 Hz), 48.09, 29.58, 26.35, 25.36, 24.45, 21.49, 12.97 (d, *J* = 2.4 Hz), 9.86.

^{31}P NMR (121 MHz, Chloroform-*d*) δ 37.57.

HRMS (ESI-MS) calculated for $C_{27}H_{34}O_4P$ $[M+H]^+$: 453.2195, found: 453.2197.

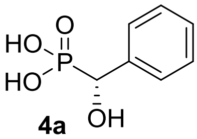

4a

(*R*)-(Hydroxy(phenyl)methyl)phosphonic acid[3] (**4a**).

$[\alpha]_D^{25}$ = +18.00 °(c = 1.00 in MeOH);

68 mg, 90% yield, white solid,

m.p. = 220.0-221.0 ℃.

^{1}H NMR (300 MHz, Deuterium Oxide) δ 7.51-6.91 (m, 5H), 4.85 (d, J = 12.3 Hz, 1H).

^{13}C NMR (75 MHz, Deuterium Oxide) δ 136.93 (d, *J* = 2.2 Hz), 128.44 (d, *J* = 2.4 Hz), 128.21 (d, *J* = 3.0 Hz), 127.03 (d, *J* = 5.8 Hz), 71.63, 69.52.

^{31}P NMR (121 MHz, Deuterium Oxide) δ 19.90.

HRMS (ESI-MS) calculated for $C_7H_{10}O_4P$ $[M+H]^+$: 189.0312, found: 189.0305.

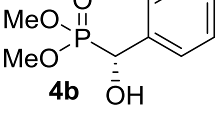

4b

Dimethyl (*R*)-(Hydroxy(phenyl)methyl)phosphonate[7] (**4b**).

$[\alpha]_D^{25}$ = +32.00 °(c = 1.00 in MeOH);

The residue was purified by flash chromatography (PE/EtOAc = 1:1) on silica gel to afford the product (83mg, 95% yield), white solid. R_f = 0.30 (PE/EA = 1:1).

m.p. = 120.0-121.0 ℃

^{1}H NMR (300 MHz, Chloroform-*d*) δ 7.49 (dt, *J* = 8.0, 2.0 Hz, 2H), 7.42-7.25 (m, 3H), 5.05 (d, *J* = 11.2 Hz, 1H), 4.76 (s, 1H), 3.67 (dd, J = 10.4, 8.2 Hz, 6H).

^{13}C NMR (75 MHz, Chloroform-*d*) δ 136.59 (d, *J* = 1.9 Hz), 128.45 (d, *J* = 2.5 Hz), 128.28 (d, *J* = 3.2 Hz), 127.16 (d, *J* = 5.9 Hz), 71.67, 69.55, 54.07 (d, *J* = 7.1 Hz), 53.73 (d, *J* = 7.4 Hz).

^{31}P NMR (121 MHz, Chloroform-*d*) δ 23.65.

HRMS (ESI-MS) calculated for $C_9H_{14}O_4PNa$ $[M+Na]^+$:239.0444; found: 239.0443.

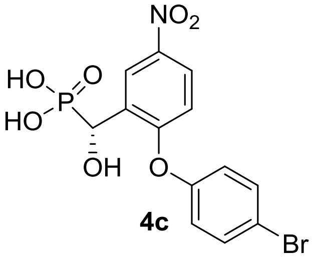

4c

(*R*)-((2-(4-Bromophenoxy)-5-nitrophenyl)(hydroxy)methyl)phosphonic acid[4] (**4c**).

$[\alpha]_D^{25}$ = +41.00 °(c = 1.00 in MeOH);

145 mg, 90% yield, white solid.

m.p. = 106.0-107.0 ℃, R_f = 0.42

^{1}H NMR (300 MHz, Methanol-*d*4) δ 8.64 (s, 1H), 8.11 (d, *J* = 8.9 Hz, 1H), 7.59 (d, *J* = 8.0 Hz, 2H), 7.10 (d, *J* = 8.1 Hz, 2H), 6.84 (d, *J* = 8.9 Hz, 1H), 5.51 (d, *J* = 14.1 Hz, 1H).

^{13}C NMR (75 MHz, Methanol-*d*4) δ 160.98, 134.34, 132.11, 126.12, 125.51, 123.66, 118.96, 116.88, 66.04, 63.90.

^{31}P NMR (121 MHz, Methanol-*d*4) δ 18.84.

HRMS (ESI-MS) calculated for $C_{13}H_{12}BrNO_7P$ $[M+H]^+$: 403.9530, found: 403.9531.

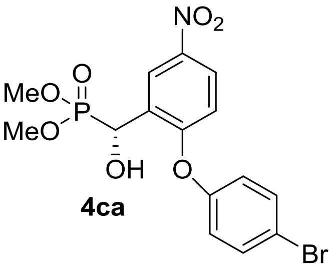

4ca

dimethyl (R)-((2-(4-bromophenoxy)-5-nitrophenyl)(hydroxy)methyl) phosphonate (4ca)

155 mg, 90% yiedld, white solid.

^{1}H NMR (300 MHz, Chloroform-*d*) δ 8.70 (t, *J* = 2.7 Hz, 1H), 8.07 (dt, *J* = 9.2, 2.3 Hz, 1H), 7.60 – 7.49 (m, 2H), 7.04 – 6.93 (m, 2H), 6.78 (d, *J* = 9.0 Hz, 1H), 6.06 (s, 1H), 5.76 – 5.65 (m, 1H), 3.81 (dd, *J* = 16.4, 10.5 Hz, 6H).

^{13}C NMR (75 MHz, Chloroform-*d*) δ 159.28 (d, *J* = 5.6 Hz), 153.98, 143.30 (d, *J* = 3.3 Hz), 133.46, 128.81 (d, *J* = 1.3 Hz), 125.21 (d, *J* = 4.2 Hz), 124.98 (d, *J* = 2.9 Hz), 122.12, 118.34, 115.66 (d, *J* = 2.2 Hz), 65.17, 63.03, 54.56 (d, *J* = 7.3 Hz), 53.93 (d, *J* = 7.2 Hz).

^{31}P NMR (121 MHz, Chloroform-*d*) δ 22.66.

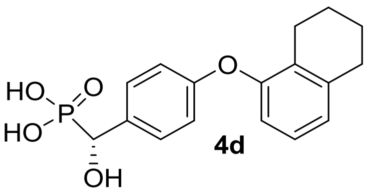

4d

***R*-(Hydroxy(4-((5,6,7,8-tetrahydronaphthalen-1-yl)oxy)phenyl)methyl)phosphonic acid** [5-6](**4d**).

$[\alpha]_D^{25}$ = +12.00 °(c = 1.00 in MeOH);

119 mg, 90% yield, white solid.

m.p. = 140.8-141.8 ℃, R_f = 0.42

^{1}H NMR (300 MHz, Methanol-d_4) δ 7.48-7.39 (m, 2H), 7.05 (t, *J* = 7.8 Hz, 1H), 6.89 (d, *J* = 7.6 Hz, 1H), 6.82 (d, *J* = 8.3 Hz, 2H), 6.66 (d, *J* = 7.9 Hz, 1H), 4.85 (d, *J* = 12.7 Hz, 1H), 2.84-2.74 (m, 2H), 2.61 (d, *J* = 5.3 Hz, 2H), 1.76 (p, *J* = 3.2 Hz, 4H).

^{13}C NMR (75 MHz, Methanol-*d*4) δ 130.06 (d, J = 5.7 Hz), 127.24, 126.08, 117.91, 117.55, 117.52, 30.48, 24.39, 24.02, 23.82.

^{31}P NMR (121 MHz, Methanol-d_4) δ 20.48.

HRMS (ESI-MS) $[M-H]^-$: calculated for $C_{17}H_{18}O_5P$: 333.0892. found: 333.0893.

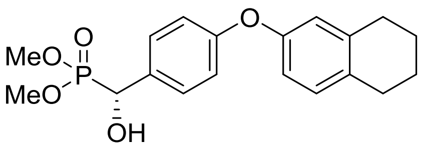

4da

dimethyl (R)-(hydroxy(4-((5,6,7,8-tetrahydronaphthalen-2-yl)oxy)phenyl)methyl) phosphonate (4da)

130 mg, 90% yield, white solid.

^{1}H NMR (300 MHz, Chloroform-*d*) δ 7.46 – 7.36 (m, 2H), 7.07 (t, *J* = 7.8 Hz, 1H), 6.91 (dd, *J* = 7.5, 1.4 Hz, 3H), 6.72 (d, *J* = 7.9 Hz, 1H), 5.00 (d, *J* = 10.2 Hz, 1H), 3.72 (dd, *J* = 15.4, 10.4 Hz, 6H), 3.18 (s, 1H), 2.80 (s, 2H), 2.64 (d, *J* = 6.6 Hz, 2H), 1.76 (p, *J* = 3.1 Hz, 4H).

^{1}H NMR (300 MHz, Chloroform-*d*) δ 7.41 (dd, *J* = 8.7, 2.3 Hz, 2H), 7.07 (t, *J* = 7.8 Hz, 1H), 6.91 (d, *J* = 8.1 Hz, 3H), 6.72 (d, *J* = 8.0 Hz, 1H), 5.00 (d, *J* = 10.2 Hz, 1H), 3.74 (d, *J* = 10.4 Hz, 1H), 3.69 (d, *J* = 10.4 Hz, 1H), 2.88 – 2.51 (m, 4H), 1.82 – 1.71 (m, 4H).

^{13}C NMR (75 MHz, Chloroform-*d*) δ 158.12 (d, *J* = 3.1 Hz), 153.95, 139.65, 130.16 (d, *J* = 2.0 Hz), 129.51, 128.69 (d, *J* = 6.1 Hz), 126.15, 125.14, 117.24 (d, *J* = 2.3 Hz), 116.87, 71.35, 69.21, 54.03 (d, *J* = 7.1 Hz), 53.82, 53.72, 29.63, 23.42, 22.89, 22.70.

^{31}P NMR (121 MHz, Chloroform-*d*) δ 23.51.

3.3 Characterization data of aldimine-based Pudovik reaction

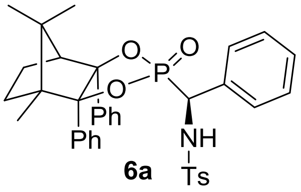

6a

4-Methyl-*N*-((1*S*)-phenyl((3a*S*,4*S*,7*S*,7a*S*)-4,8,8-trimethyl-2-oxido-3a,7a-diphenylhexa-hydro-4,7-methanobenzo[*d*][1,3,2]dioxaphosphol-2-yl)methyl)benzenesulfonamide (**6a**).

$[\alpha]_D^{25}$ = +157.97 °(c = 1.00 in MeOH);

The residue was purified by flash chromatography (PE/EtOAc = 5:1) on silica gel to afford the product (238 mg, 95% yield), white solid. R_f = 0.40 (PE/EA = 5:1).

m.p. = 155.0-157.0 ℃

^{1}H NMR (300 MHz, Chloroform-*d*) δ 7.48 – 7.29 (m, 9H), 7.24 (d, *J* = 7.4 Hz, 2H), 6.87 – 6.72 (m, 4H), 6.66 (t, *J* = 7.6 Hz, 2H), 6.55 – 6.48 (m, 2H), 4.76 (dd, *J* = 20.9, 10.4 Hz, 1H), 3.00 (d, *J* =

5.3 Hz, 1H), 2.17 (s, 3H), 2.13 – 2.00 (m, 1H), 1.80 (s, 0H), 1.64 – 1.46 (m, 2H), 1.44 – 1.32 (m, 1H), 1.17 (s, 3H), 0.97 (s, 3H).

^{13}C NMR (75 MHz, Chloroform-*d*) δ 142.44, 138.44 (d, *J* = 2.2 Hz), 137.81 (d, *J* = 3.4 Hz), 135.56 (d, *J* = 4.2 Hz), 132.47, 130.52, 128.89, 128.69, 128.33 (d, *J* = 5.8 Hz), 127.86 (d, *J* = 2.2 Hz), 127.43, 126.83, 101.11, 100.32, 57.80, 56.03 (d, *J* = 3.2 Hz), 55.86, 52.33 (d, *J* = 3.5 Hz), 48.52, 29.74, 26.59, 24.44, 21.94, 21.38, 10.01.

^{31}P NMR (121 MHz, Chloroform-*d*) δ 32.25.

HRMS (ESI-MS) calculated for $C_{36}H_{39}NO_5PS$:628.2282 [M+H]: found: 628.2284.

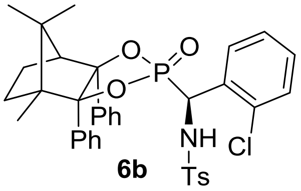

6b

***N*-((1*S*)-(2-Chlorophenyl)((3a*S*,4*S*,7*S*,7a*S*)-4,8,8-trimethyl-2-oxido-3a,7a-diphenylhexahydro-4,7-methanobenzo[*d*][1,3,2]dioxaphosphol-2-yl)methyl)-4-methylbenzenesulfonamide** (**6b**).

$[\alpha]_D^{25}$ = +127.98 °(c = 1.00 in MeOH);

The residue was purified by flash chromatography (PE/EtOAc = 5:1) on silica gel to afford the product (256 mg, 97% yield), white solid. R_f = 0.42 (PE/EA = 5:1).

m.p. = 162.2-164.2 ℃

^{1}H NMR (300 MHz, Chloroform-*d*) δ 7.70 – 7.14 (m, 13H), 6.96 – 6.81 (m, 1H), 6.74 – 6.63 (m, 4H), 6.47 (dt, *J* = 8.3, 4.1 Hz, 1H), 5.18 (dd, *J* = 21.9, 10.8 Hz, 1H), 2.99 (d, *J* = 5.2 Hz, 1H), 2.10 – 1.95 (m, 4H), 1.85 (s, 3H), 1.55 – 1.42 (m, 2H), 1.40 – 1.26 (m, 1H), 1.15 (s, 3H), 1.07 (s, 3H).

^{13}C NMR (75 MHz, Chloroform-*d*) δ 142.38, 137.81 (d, *J* = 2.2 Hz), 136.78 (d, *J* = 3.3 Hz), 135.66 (d, *J* = 4.4 Hz), 132.72 (d, *J* = 8.5 Hz), 131.80, 131.48 (d, *J* = 4.0 Hz), 130.60, 128.88, 128.80, 128.44 (d, *J* = 2.7 Hz), 128.29, 128.06, 127.93, 126.79, 126.60, 126.50, 101.57, 101.40, 55.97 (d, *J* = 3.2 Hz), 52.23 (d, *J* = 3.5 Hz), 48.50, 29.96, 26.72, 24.49, 22.16, 21.29, 10.02.

^{31}P NMR (121 MHz, Chloroform- *d*) δ 32.82.

HRMS (ESI-MS) calculated for $C_{36}H_{38}ClNO_5PS$ $[M+H]^+$: 662.1892, found: 662.1894.

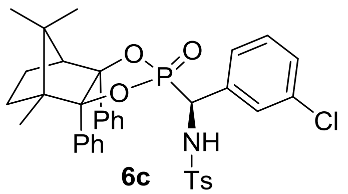

6c

***N*-((1*S*)-(3-Chlorophenyl)((3a*S*,4*S*,7*S*,7a*S*)-4,8,8-trimethyl-2-oxido-3a,7a-diphenylhexahydro-4,7-methanobenzo[*d*][1,3,2]dioxaphosphol-2-yl)methyl)-4-methylbenzenesulfonamide** (**6c**).

$[\alpha]_D^{25}$ = +150.97 °(c = 1.00 in MeOH);

The residue was purified by flash chromatography (PE/EtOAc = 5:1) on silica gel to afford the product (251 mg, 95% yield), white solid. R_f = 0.40 (PE/EA = 5:1).

m.p. = 134.0-136.0 ℃

^{1}H NMR (300 MHz, Chloroform-*d*) δ 7.61 (dd, *J* = 10.4, 3.9 Hz, 1H), 7.48-7.21 (m, 9H), 7.20-7.11 (m, 3H), 6.68 (dd, *J* = 15.3, 6.6 Hz, 3H), 6.50-6.36 (m, 2H), 6.05 (d, *J* = 2.5 Hz, 1H), 4.67 (dd, *J* = 21.8, 10.3 Hz, 1H), 2.96 (d, *J* = 5.1 Hz, 1H), 2.05 (s, 3H), 2.02-1.96 (m, 1H), 1.79 (s, 3H), 1.60-1.39 (m, 2H), 1.37-1.25 (m, 1H), 1.09 (s, 3H), 0.95 (s, 3H).

^{13}C NMR (75 MHz, Chloroform-*d*) δ 142.35, 138.36 (d, *J* = 2.2 Hz), 137.71 (d, *J* = 3.1 Hz), 135.31 (d, *J* = 4.1 Hz), 134.40, 133.29 (d, *J* = 2.2 Hz), 130.53, 129.06 (d, *J* = 2.4 Hz), 128.92, 128.72, 128.47 (d, *J* = 6.4 Hz), 128.33, 127.17 (d, *J* = 3.0 Hz), 126.71, 126.29 (d, *J* = 5.0 Hz), 101.20, 100.33, 57.19, 56.08 (d, *J* = 3.1 Hz), 55.24, 52.32 (d, J = 3.4 Hz), 48.51, 29.68, 26.48, 24.23, 22.18, 21.26, 9.92.

^{31}P NMR (121 MHz, Chloroform-*d*) δ 31.25.

HRMS (ESI-MS) calculated for $C_{36}H_{38}ClNO_5PS$: $[M+H]^+$: 662.1892, found: 662.1890.

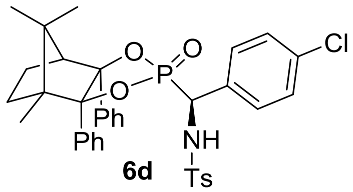

6d

***N*-((1*S*)-(4-Chlorophenyl)((3a*S*,4*S*,7*S*,7a*S*)-4,8,8-trimethyl-2-oxido-3a,7a-diphenylhexahydro-4,7-methanobenzo[*d*][1,3,2]dioxaphosphol-2-yl)methyl)-4-methylbenzenesulfonamide** (**6d**).

$[\alpha]_D^{25}$ = +97.98 °(c = 1.00 in MeOH);

The residue was purified by flash chromatography (PE/EtOAc = 5:1) on silica gel to afford the product (254 mg, 96% yield), white solid. R_f = 0.40 (PE/EA = 5:1).

m.p. = 124.0-126.0 ℃

^{1}H NMR (300 MHz, Chloroform-*d*) δ 7.78 (dd, *J* = 10.6, 3.4 Hz, 1H), 7.55-7.20 (m, 9H), 7.15 (d, *J* = 8.2 Hz, 3H), 6.69 (d, *J* = 8.0 Hz, 2H), 6.44 (d, *J* = 8.2 Hz, 2H), 6.25 (dd, *J* = 8.6, 2.2 Hz, 2H), 4.71 (dd, *J* = 21.8, 10.5 Hz, 1H), 2.95 (d, *J* = 5.1 Hz, 1H), 2.07 (s, 3H), 2.02-1.95 (m, 1H), 1.78 (s, 3H), 1.55-1.36 (m, 2H), 1.35-1.23 (m, 1H), 1.08 (s, 3H), 0.94 (s, 3H).

^{13}C NMR (75 MHz, Chloroform-*d*) δ 142.39, 138.45 (d, *J* = 2.3 Hz), 137.73 (d, *J* = 3.2 Hz), 135.26 (d, *J* = 4.1 Hz), 133.16 (d, *J* = 3.6 Hz), 131.04 (d, *J* = 1.5 Hz), 130.47, 129.59 (d, *J* = 5.7 Hz), 128.80, 128.66, 128.27, 127.66 (d, *J* = 2.3 Hz), 126.77, 101.16, 100.24, 57.08, 56.00 (d, *J* = 3.0 Hz), 55.11, 52.25 (d, *J* = 3.4 Hz), 48.45, 31.56, 29.65, 26.45, 24.19, 22.64, 22.06, 21.20, 14.17, 9.88.

^{31}P NMR (121 MHz, Chloroform-*d*) δ 31.41.

HRMS (ESI-MS) calculated for $C_{36}H_{38}ClNO_5PS$ $[M+H]^+$: 662.1892, found: 662.1891.

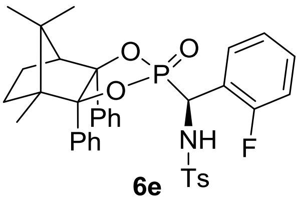

6e

***N*-((1*S*)-(2-Fluorophenyl)((3a*S*,4*S*,7*S*,7a*S*)-4,8,8-trimethyl-2-oxido-3a,7a-diphenylhexahydro-4,7-methanobenzo[*d*][1,3,2]dioxaphosphol-2-yl)methyl)-4-methylbenzenesulfonamide** (**6e**).

$[\alpha]_D^{25}$ = +92.98 °(c = 1.00 in MeOH);

The residue was purified by flash chromatography (PE/EtOAc = 5:1) on silica gel to afford the product (245 mg, 95% yield), white solid. R_f = 0.38 (PE/EA = 5:1).

m.p. = 169.5-171.5 ℃

^{1}H NMR (300 MHz, Chloroform-*d*) δ 7.35 (d, *J* = 8.3 Hz, 8H), 7.23 (s, 3H), 7.12 (t, *J* = 7.7 Hz, 2H), 6.84 (t, *J* = 7.2 Hz, 3H), 6.59 (dt, *J* = 9.9, 5.8 Hz, 2H), 6.36 (t, *J* = 9.2 Hz, 1H), 5.01 (dd, *J* = 21.0, 10.7 Hz, 1H), 2.98 (d, *J* = 5.2 Hz, 1H), 2.18 (s, 3H), 2.08 (d, *J* = 5.7 Hz, 1H), 1.80 (s, 3H), 1.62-1.46 (m, 2H), 1.33-1.24 (m, 1H), 1.16 (s, 3H), 0.97 (s, 3H).

^{13}C NMR (75 MHz, Chloroform-*d*) δ 142.62, 137.61 (d, *J* = 2.1 Hz), 137.33 (d, *J* = 3.5 Hz), 135.27 (d, *J* = 4.1 Hz), 130.34, 130.19, 129.58 (d, *J* = 8.6 Hz), 128.82, 128.27 (d, *J* = 4.0 Hz), 127.74, 126.80, 126.67, 124.12, 120.45 (d, *J* = 13.7 Hz), 114.97 (d, *J* = 22.3 Hz), 101.47, 100.73, 55.90 (d, *J* = 3.3 Hz), 52.14 (d, *J* = 3.3 Hz), 51.74, 49.73, 48.34, 29.60, 26.60, 24.68, 21.81, 21.35, 10.01.

^{19}F NMR (282 MHz, Chloroform-*d*) δ -113.70 (dq, *J* = 11.1, 5.3 Hz).

^{31}P NMR (121 MHz, Chloroform-*d*) δ 31.92 (d, *J* = 4.6 Hz).

HRMS (ESI-MS) calculated for $C_{36}H_{38}FNO_5PS$ $[M+H]^+$: 646.2187, found: 646.2187.

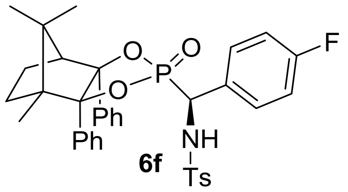

6f

***N*-((1*S*)-(4-Fluorophenyl)((3a*S*,4*S*,7*S*,7a*S*)-4,8,8-trimethyl-2-oxido-3a,7a-diphenylhexa-hydro-4,7-methanobenzo[*d*][1,3,2]dioxaphosphol-2-yl)methyl)-4-methylbenzenesulfonamide (6f).**

$[\alpha]_D^{25}$ = +119.98 °(c = 1.00 in MeOH);

The residue was purified by flash chromatography (PE/EtOAc = 5:1) on silica gel to afford the product (250 mg, 97% yield), white solid. R_f = 0.40 (PE/EA = 5:1).

m.p. = 120.0-121.0 ℃

^{1}H NMR (300 MHz, Chloroform-*d*) δ 7.72 – 7.31 (m, 10H), 7.25 (d, *J* = 8.1 Hz, 2H), 6.81 (d, *J* = 8.1 Hz, 2H), 6.53 – 6.38 (m, 2H), 6.29 (t, *J* = 8.5 Hz, 2H), 4.80 (dd, *J* = 21.5, 10.4 Hz, 1H), 3.04 (d, *J* = 5.2 Hz, 1H), 2.17 (s, 3H), 2.13 – 2.00 (m, 1H), 1.85 (s, 3H), 1.69 – 1.32 (m, 3H), 1.18 (s, 3H), 1.02 (s, 3H).

^{13}C NMR (75 MHz, Chloroform-*d*) δ 142.40, 138.61 (d, *J* = 2.4 Hz), 137.84 (d, *J* = 3.2 Hz), 135.41 (d, *J* = 4.2 Hz), 130.55, 130.38 – 129.75 (m), 128.77, 128.35, 126.83, 114.55 (d, *J* = 21.0 Hz), 101.22, 100.32, 57.07, 56.08 (d, *J* = 3.1 Hz), 55.10, 52.35 (d, *J* = 3.3 Hz), 48.54, 29.74, 26.54, 24.31, 22.09, 21.28, 9.94.

^{19}F NMR (282 MHz, Chloroform-*d*) δ -114.80 (tt, *J* = 8.7, 4.6 Hz).

^{31}P NMR (121 MHz, Chloroform-*d*) δ 31.83

HRMS (ESI-MS) calculated for $C_{36}H_{38}FNO_5PS$ $[M+H]^+$: 646.2187, found: 646.2188.

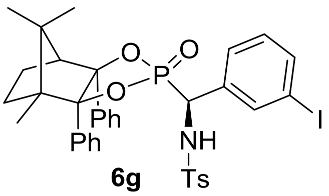

6g

***N*-((1*S*)-(3-Iodophenyl)((3a*S*,4*S*,7*S*,7a*S*)-4,8,8-trimethyl-2-oxido-3a,7a-diphenylhexahydro-4,7-methanobenzo[*d*][1,3,2]dioxaphosphol-2-yl)methyl)-4-methylbenzenesulfonamide (6g)**.

$[\alpha]_D^{25}$ = +118.98 °(c = 1.00 in MeOH);

The residue was purified by flash chromatography (PE/EtOAc = 5:1) on silica gel to afford the product (280 mg, 93% yield), white solid. R_f = 0.36 (PE/EA = 5:1).

m.p. = 107.0-109.0 ℃

^{1}H NMR (300 MHz, Chloroform-*d*) δ 7.38 (dt, *J* = 13.1, 6.5 Hz, 9H), 7.24 (d, *J* = 8.7 Hz, 3H), 7.16-7.08 (m, 1H), 6.95-6.89 (m, 1H), 6.86 (d, *J* = 8.2 Hz, 2H), 6.71 (dd, *J* = 7.7, 2.3 Hz, 1H), 6.48 (q, *J* = 1.8 Hz, 1H), 6.41 (t, *J* = 7.9 Hz, 1H), 4.61 (dd, *J* = 21.2, 10.3 Hz, 1H), 3.01 (d, *J* = 5.3 Hz, 1H), 2.21 (s, 3H), 2.10-2.01 (m, 1H), 1.79 (s, 3H), 1.55 (td, *J* = 13.8, 4.4 Hz, 2H), 1.45-1.33 (m, 1H), 1.17 (s, 3H), 0.98 (s, 3H).

^{13}C NMR (75 MHz, Chloroform-*d*) δ 142.79, 138.01 (d, *J* = 2.1 Hz), 137.62 (d, *J* = 3.2 Hz), 137.36 (d, *J* = 7.6 Hz), 136.39, 135.35 (d, *J* = 4.1 Hz), 134.51, 130.46, 129.74, 129.09, 129.05, 128.46, 127.31 (d, *J* = 4.9 Hz), 126.77, 101.29, 100.44, 93.64, 57.06, 56.09 (d, *J* = 3.3 Hz), 55.11, 52.30, 48.51, 29.68, 26.54, 24.37, 22.00, 21.55, 9.99.

^{31}P NMR (121 MHz, Chloroform-*d*) δ 31.49.

HRMS (ESI-MS) calculated for $C_{36}H_{38}INO_5PS$ $[M+H]^+$: 754.2148, found: 754.2151;

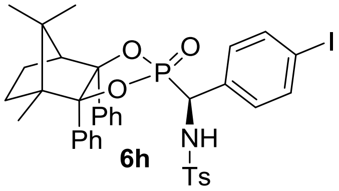

6h

***N*-((1*S*)-(4-Iodophenyl)((3a*S*,4*S*,7*S*,7aS)-4,8,8-trimethyl-2-oxido-3a,7a-diphenylhexahydro-4,7-methanobenzo[*d*][1,3,2]dioxaphosphol-2-yl)methyl)-4-methylbenzenesulfonamide** (**6h**).

$[\alpha]_D^{25}$ = +158.97 °(c = 1.00 in MeOH);

The residue was purified by flash chromatography (PE/EtOAc = 5:1) on silica gel to afford the product (283 mg, 94% yield), white solid. R_f = 0.35 (PE/EA = 5:1).

m.p. = 133.0-135.0 ℃

^{1}H NMR (300 MHz, Chloroform-*d*) δ 7.47-7.29 (m, 9H), 7.28-7.14 (m, 4H), 6.90 (d, *J* = 8.1 Hz, 2H), 6.81 (d, *J* = 8.1 Hz, 2H), 6.18-6.09 (m, 2H), 4.70 (dd, *J* = 21.5, 10.5 Hz, 1H), 3.03 (d, *J* = 5.2 Hz, 1H), 2.23 (s, 3H), 2.10 (dd, *J* = 13.2, 5.7 Hz, 1H), 1.82 (s, 3H), 1.57 (ddt, *J* = 18.3, 14.1, 6.6 Hz, 2H), 1.47-1.35 (m, 1H), 1.18 (s, 3H), 1.01 (s, 3H).

^{13}C NMR (75 MHz, Chloroform-*d*) δ 142.70, 138.31 (d, *J* = 2.2 Hz), 137.96-137.69 (m), 136.75 (d, *J* = 2.3 Hz), 135.33 (d, *J* = 4.1 Hz), 132.12 (d, *J* = 1.6 Hz), 130.50, 130.18 (d, J = 5.8 Hz), 128.84, 128.39, 126.83, 101.24, 100.32, 93.28 (d, *J* = 4.0 Hz), 57.29, 56.07 (d, *J* = 3.3 Hz), 55.33, 52.35, 48.52, 29.70, 26.53, 24.29, 22.03, 21.51, 10.02.

^{31}P NMR (121 MHz, Chloroform-*d*) δ 31.43.

HRMS (ESI-MS) calculated for $C_{36}H_{38}INO_5PS$ $[M+H]^+$: 754.2148, found: 754.2152.

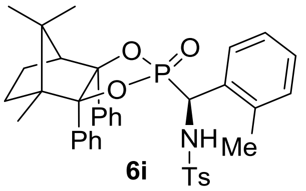

6i

4-Methyl-*N*-((1*S*)-o-tolyl((3a*S*,4*S*,7*S*,7aS)-4,8,8-trimethyl-2-oxido-3a,7a-diphenylhexahydro-4,7-methanobenzo[*d*][1,3,2]dioxaphosphol-2-yl)methyl)benzenesulfonamide (**6i**).

$[\alpha]_D^{25}$ = +64 °(c = 1.00 in MeOH);

The residue was purified by flash chromatography (PE/EtOAc = 5:1) on silica gel to afford the product (228 mg, 89% yield), white solid. R_f = 0.45 (PE/EA = 5:1).

m.p. = 117.0-119.0 ℃

^{1}H NMR (300 MHz, Chloroform-*d*) δ 7.58-7.22 (m, 11H), 7.19 (d, *J* = 8.0 Hz, 3H), 6.90 (dd, *J* = 10.7, 3.4 Hz, 1H), 6.74 (t, *J* = 8.6 Hz, 3H), 6.63-6.48 (m, 2H), 5.00 (dd, *J* = 21.6, 10.4 Hz, 1H), 3.05 (d, *J* = 5.2 Hz, 1H), 2.12 (s, 3H), 2.05 (d, *J* = 6.4 Hz, 1H), 1.92 (s, 3H), 1.86 (s, 1H), 1.54 (q, *J* = 6.7, 4.9 Hz, 2H), 1.38 (s, 3H), 1.22 (s, 3H), 1.14 (s, 3H).

^{13}C NMR (75 MHz, Chloroform-*d*) δ 142.30, 138.14, 137.03, 135.91 (d, *J* = 4.5 Hz), 135.69, 131.96, 130.68, 130.15, 129.75, 128.79, 128.49, 128.02 (d, *J* = 8.3 Hz), 127.28, 126.54, 126.48, 126.08, 101.18, 56.00, 54.14, 52.31, 48.58, 30.05, 26.75, 24.44, 22.15, 21.29, 19.06, 10.01.

^{31}P NMR (121 MHz, Chloroform-*d*) δ 34.09.

HRMS (ESI-MS) calculated for $C_{37}H_{41}NO_5PS$ $[M+H]^+$: 642.2438, found: 642.2439.

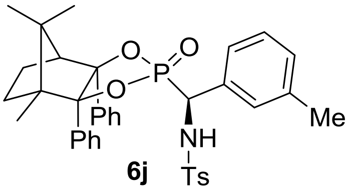

6j

4-Methyl-*N*-((1*S*)-m-tolyl((3a*S*,4*S*,7*S*,7a*S*)-4,8,8-trimethyl-2-oxido-3a,7a-diphenylhexahydro-4,7-methanobenzo[*d*][1,3,2]dioxaphosphol-2-yl)methyl)benzenesulfonamide (**6j**).

$[\alpha]_D^{25}$ = +122.98 °(c = 1.00 in MeOH);

The residue was purified by flash chromatography (PE/EtOAc = 5:1) on silica gel to afford the product (236 mg, 92% yield), white solid. R_f = 0.45 (PE/EA = 5:1).

m.p. = 117.0-118.0 ℃

^{1}H NMR (300 MHz, Chloroform-*d*) δ 7.53-7.06 (m, 13H), 6.78 (d, *J* = 8.1 Hz, 2H), 6.63-6.46 (m, 2H), 6.46-6.37 (m, 1H), 6.07 (d, *J* = 2.6 Hz, 1H), 4.72 (dd, *J* = 21.2, 10.4 Hz, 1H), 3.01 (d, *J* = 5.2 Hz, 1H), 2.14 (s, 3H), 2.06 (d, *J* = 5.3 Hz, 1H), 1.83 (s, 3H), 1.74 (s, 3H), 1.64-1.45 (m, 2H), 1.35 (tt, *J* = 13.9, 11.5, 4.6 Hz, 1H), 1.16 (s, 3H), 0.99 (s, 3H).

^{13}C NMR (75 MHz, Chloroform-*d*) δ 142.04, 138.50 (d, *J* = 2.2 Hz), 137.80 (d, *J* = 3.3 Hz), 136.87 (d, *J* = 2.1 Hz), 135.50 (d, *J* = 4.1 Hz), 131.97 (d, *J* = 1.5 Hz), 130.51, 129.06 (d, *J* = 6.6 Hz), 128.55, 128.23, 127.87 (d, *J* = 2.8 Hz), 127.71 (d, *J* = 2.3 Hz), 126.78, 125.23 (d, *J* = 5.5 Hz), 100.88, 100.05, 57.68, 55.96 (d, *J* = 3.1 Hz), 55.72, 52.27 (d, *J* = 3.3 Hz), 48.44, 29.65, 26.51, 24.31, 22.02, 21.25, 20.78, 9.93.

^{31}P NMR (121 MHz, Chloroform-*d*) δ 32.20.

HRMS (ESI-MS) calculated for $C_{37}H_{41}NO_5PS$ $[M+H]^+$: 642.2438, found: 642.2435.

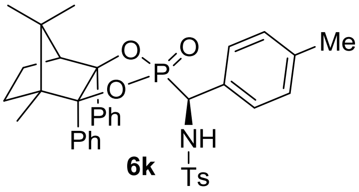

6k

4-Methyl-*N*-((1*S*)-p-tolyl((3a*S*,4*S*,7*S*,7a*S*)-4,8,8-trimethyl-2-oxido-3a,7a-diphenylhexahydro-4,7-methanobenzo[*d*][1,3,2]dioxaphosphol-2-yl)methyl)benzenesulfonamide (**6k**).

$[\alpha]_D^{25}$ = +184.97 °(c = 1.00 in MeOH);

The residue was purified by flash chromatography (PE/EtOAc = 5:1) on silica gel to afford the product (244 mg, 95% yield), white solid. R_f = 0.43 (PE/EA = 5:1).

m.p. = 135.2-137.2 ℃

^{1}H NMR (300 MHz, Chloroform-*d*) δ 7.51-7.17 (m, 12H), 6.84 (d, *J* = 8.0 Hz, 2H), 6.75 (dd, *J* = 10.6, 4.3 Hz, 1H), 6.50-6.40 (m, 2H), 6.19 (d, *J* = 8.4 Hz, 2H), 4.69 (dd, *J* = 20.6, 10.3 Hz, 1H), 3.53 (s, 3H), 3.00 (d, *J* = 5.3 Hz, 1H), 2.19 (s, 3H), 2.04 (dd, *J* = 13.2, 5.7 Hz, 1H), 1.79 (s, 3H), 1.53 (tt, *J* = 13.5, 5.7 Hz, 2H), 1.37 (dt, *J* = 10.3, 5.9 Hz, 1H), 1.16 (s, 3H), 0.96 (s, 3H).

^{13}C NMR (75 MHz, Chloroform-*d*) δ 158.66 (d, *J* = 2.7 Hz), 141.77, 138.66 (d, *J* = 2.4 Hz), 137.74 (d, *J* = 3.3 Hz), 135.43 (d, *J* = 4.1 Hz), 130.48, 129.49 (d, *J* = 5.9 Hz), 128.53, 128.12, 126.77, 124.41 (d, *J* = 1.5 Hz), 113.04 (d, *J* = 2.2 Hz), 100.81, 99.91, 60.35, 57.11, 55.88 (d, *J* = 3.0 Hz), 54.90, 52.17 (d, *J* = 3.3 Hz), 48.37, 29.60, 26.46, 24.23, 22.02, 21.17, 14.18, 9.87.

^{31}P NMR (121 MHz, Chloroform-*d*) δ 32.51.

HRMS (ESI-MS) calculated for $C_{37}H_{41}NO_5PS$ $[M+H]^+$: 642.2438, found: 642.2439.

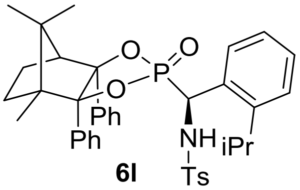

6l

***N*-((1*S*)-(2-Isopropylphenyl)((3a*S*,4*S*,7*S*,7a*S*)-4,8,8-trimethyl-2-oxido-3a,7a-diphenylhexahydro-4,7-methanobenzo[*d*][1,3,2]dioxaphosphol-2-yl)methyl)-4-methylbenzenesulfonamide (6l)**.

$[\alpha]_D^{25}$ = +144.98 °(c = 1.00 in MeOH);

The residue was purified by flash chromatography (PE/EtOAc = 5:1) on silica gel to afford the product (233 mg, 87% yield), white solid. R_f = 0.42 (PE/EA = 5:1).

m.p. = 141.0-143.0 ℃

^{1}H NMR (300 MHz, Chloroform-*d*) δ 7.80-6.82 (m, 14H), 6.72 (p, *J* = 6.9, 6.0 Hz, 2H), 6.62 (d, *J* = 8.0 Hz, 2H), 6.45-6.33 (m, 1H), 5.16 (dt, *J* = 22.1, 5.3 Hz, 1H), 3.00 (d, *J* = 5.1 Hz, 1H), 2.05 (d, *J* = 11.1 Hz, 1H), 1.99 (s, 3H), 1.89 (s, 3H), 1.67 (p, *J* = 6.5 Hz, 1H), 1.57-1.41 (m, 2H), 1.41-1.27 (m, 1H), 1.15 (s, 6H), 0.59 (d, *J* = 6.6 Hz, 3H), 0.38 (d, *J* = 6.5 Hz, 3H).

^{13}C NMR (75 MHz, Chloroform-*d*) δ 145.99 (d, *J* = 7.1 Hz), 141.90, 138.43 (t, *J* = 2.7 Hz),

136.50 (d, J = 3.0 Hz), 136.04 (d, J = 4.6 Hz), 130.75, 130.35-129.96 (m), 128.74, 128.64, 127.88, 127.15, 126.46, 125.89, 124.56, 101.65 (d, J = 1.8 Hz), 101.03, 56.04 (d, J = 2.9 Hz), 52.24 (d, J = 3.3 Hz), 48.60, 30.15, 27.82, 26.84, 24.50, 24.01, 22.42, 21.22, 10.08.

^{31}P NMR (121 MHz, Chloroform-*d*) δ 34.24.

HRMS (ESI-MS) calculated for $C_{39}H_{45}NO_5PS$ [M+H]$^+$: 670.2751, found: 670.2751.

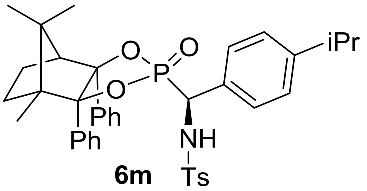

6m

***N*-((1*S*)-(4-Isopropylphenyl)((3a*S*,4*S*,7*S*,7a*S*)-4,8,8-trimethyl-2-oxido-3a,7a-diphenylhexahydro-4,7-methanobenzo[*d*][1,3,2]dioxaphosphol-2-yl)methyl)-4-methylbenzenesulfonamide (6m)**.

$[\alpha]_D^{25}$ = +210.96 °(c = 1.00 in MeOH);

The residue was purified by flash chromatography (PE/EtOAc = 5:1) on silica gel to afford the product (254 mg, 95% yield), white solid. R_f = 0.42 (PE/EA = 5:1).

m.p. = 145.5-147.5 ℃

^{1}H NMR (300 MHz, Chloroform-*d*) δ 7.75 (dd, J = 10.7, 3.1 Hz, 1H), 7.67-7.28 (m, 10H), 7.22 (d, J = 8.0 Hz, 2H), 6.71 (d, J = 8.0 Hz, 2H), 6.38 (s, 4H), 4.81 (dd, J = 21.6, 10.7 Hz, 1H), 3.05 (d, J = 5.1 Hz, 1H), 2.50 (p, J = 6.9 Hz, 1H), 2.07 (s, 4H), 1.89 (s, 3H), 1.58 (dh, J = 22.6, 8.1 Hz, 2H), 1.38 (td, J = 12.1, 11.2, 4.3 Hz, 1H), 1.17 (s, 3H), 1.04 (s, 3H), 0.95 (dd, J = 7.0, 4.7 Hz, 6H).

^{13}C NMR (75 MHz, Chloroform-*d*) δ 147.49 (d, J = 3.1 Hz), 141.43, 138.63 (d, J = 2.4 Hz), 137.79 (d, J = 3.2 Hz), 135.42 (d, J = 4.1 Hz), 130.56, 129.53 (d, J = 1.4 Hz), 128.60, 128.41, 128.22, 128.13, 126.78, 125.53, 100.79, 100.01, 57.51, 55.93 (d, J = 3.1 Hz), 55.54, 52.21, 48.42, 33.47, 29.62, 26.46, 24.26, 23.78 (d, J = 6.1 Hz), 22.08, 21.17, 9.96.

^{31}P NMR (121 MHz, Chloroform-*d*) δ 32.12.

HRMS (ESI-MS) calculated for $C_{39}H_{45}NO_5PS$ [M+H]$^+$: 670.2751, found: 670.2751.

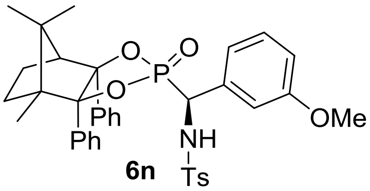

6n

***N*-((1*S*)-(3-Methoxyphenyl)((3a*S*,4*S*,7*S*,7a*S*)-4,8,8-trimethyl-2-oxido-3a,7a-diphenylhexahydro-4,7-methanobenzo[*d*][1,3,2]dioxaphosphol-2-yl)methyl)-4-methylbenzenesulfonamide (6n)**.

$[\alpha]_D^{25}$ = +255.96 °(c = 1.00 in MeOH);

The residue was purified by flash chromatography (PE/EtOAc = 5:1) on silica gel to afford the product (239 mg, 91% yield), white solid. R_f = 0.44 (PE/EA = 5:1).

m.p. = 119.0-121.0 ℃

^{1}H NMR (300 MHz, Chloroform-*d*) δ 7.60-7.29 (m, 9H), 7.24 (d, *J* = 8.3 Hz, 3H), 7.02 (dd, *J* = 10.6, 3.8 Hz, 1H), 6.81 (d, *J* = 8.0 Hz, 2H), 6.54 (t, *J* = 7.9 Hz, 1H), 6.35 (dt, *J* = 8.3, 2.4 Hz, 1H), 6.04 (d, *J* = 7.7 Hz, 1H), 5.97 (q, *J* = 2.1 Hz, 1H), 4.81 (dd, *J* = 21.6, 10.5 Hz, 1H), 3.12 (s, 3H), 3.04 (d, *J* = 5.3 Hz, 1H), 2.16 (s, 3H), 2.13-2.06 (m, 1H), 1.84 (s, 3H), 1.65-1.47 (m, 2H), 1.47-1.33 (m, 1H), 1.18 (s, 3H), 1.02 (s, 3H).

^{13}C NMR (75 MHz, Chloroform-*d*) δ 158.93 (d, *J* = 2.1 Hz), 142.39, 138.49 (d, *J* = 2.4 Hz), 137.81 (d, *J* = 3.2 Hz), 135.50 (d, *J* = 4.1 Hz), 133.70, 130.58, 128.79, 128.74, 128.29, 126.83, 120.47 (d, *J* = 5.8 Hz), 114.50, 112.14 (d, *J* = 5.9 Hz), 101.06, 100.38, 57.83, 56.04 (d, *J* = 3.1 Hz), 55.87, 54.83, 52.32 (d, *J* = 3.3 Hz), 48.51, 29.75, 26.60, 24.42, 22.07, 21.33, 9.96.

^{31}P NMR (121 MHz, Chloroform-*d*) δ 32.19.

HRMS (ESI-MS) calculated for $C_{37}H_{41}NO_6PS$ $[M+H]^+$: 658.2387, found: 658.2385.

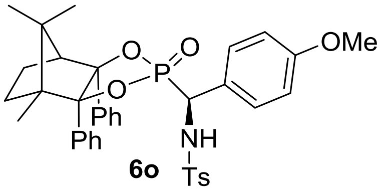

6o

***N*-((1*S*)-(4-Methoxyphenyl)((3a*S*,4*S*,7*S*,7a*S*)-4,8,8-trimethyl-2-oxido-3a,7a-diphenylhexahydro-4,7-methanobenzo[*d*][1,3,2]dioxaphosphol-2-yl)methyl)-4-methylbenzenesulfonamide (6o)**.

$[\alpha]_D^{25}$ = +162.97 °(c = 1.00 in MeOH);

The residue was purified by flash chromatography (PE/EtOAc = 5:1) on silica gel to afford the product (244 mg, 93% yield), white solid. R_f = 0.40 (PE/EA = 5:1).

m.p. = 138.0-140.0 ℃

^{1}H NMR (300 MHz, Chloroform-*d*) δ 7.44-7.03 (m, 13H), 6.70 (d, *J* = 8.0 Hz, 2H), 6.41-6.22 (m, 4H), 4.66 (dd, *J* = 21.0, 10.4 Hz, 1H), 2.92 (d, *J* = 5.0 Hz, 1H), 2.07 (s, 3H), 1.92 (d, *J* = 1.8 Hz, 4H), 1.74 (s, 3H), 1.55-1.37 (m, 2H), 1.35-1.23 (m, 1H), 1.07 (s, 3H), 0.91 (s, 3H).

^{13}C NMR (75 MHz, Chloroform-*d*) δ 142.01, 138.59 (d, *J* = 2.3 Hz), 137.79 (d, *J* = 3.2 Hz), 136.91 (d, *J* = 3.0 Hz), 135.53 (d, *J* = 4.1 Hz), 130.50, 129.28 (d, *J* = 1.7 Hz), 128.61, 128.57, 128.42-128.03 (m), 126.84, 100.87, 100.02, 57.52, 55.93 (d, *J* = 3.1 Hz), 55.56, 52.24 (d, *J* = 3.4 Hz), 48.43, 31.62,,29.66, 26.52, 24.34, 21.97, 21.26, 20.87,14.22 (d, *J* = 3.8 Hz), 9.95.

^{31}P NMR (121 MHz, Chloroform-*d*) δ 32.46.

HRMS (ESI-MS) calculated for $C_{37}H_{41}NO_6PS$ $[M+H]^+$: 658.2387, found: 658.2394.

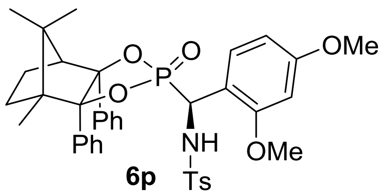

6p

***N*-((1*S*)-(2,4-Dimethoxyphenyl)((3a*S*,4*S*,7*S*,7a*S*)-4,8,8-trimethyl-2-oxido-3a,7a-dipheny-lhexahydro-4,7-methanobenzo[*d*][1,3,2]dioxaphosphol-2-yl)methyl)-4-methylbenzenesulfon-amide** (**6p**).

$[\alpha]_D^{25}$ = +116.98 °(c = 1.00 in MeOH),

The residue was purified by flash chromatography (PE/EtOAc = 5:1) on silica gel to afford the product (261 mg, 95% yield), white solid. R_f = 0.40 (PE/EA = 5:1).

m.p. = 115.0-116.5 ℃

^{1}H NMR (300 MHz, Chloroform-*d*) δ 7.52-7.13 (m, 12H), 6.86 (d, *J* = 7.9 Hz, 2H), 6.24 (d, *J* = 8.5 Hz, 1H), 6.11 (dd, *J* = 10.9, 2.7 Hz, 1H), 5.92 (dd, *J* = 8.5, 2.4 Hz, 1H), 5.80 (d, *J* = 2.4 Hz, 1H), 4.85 (dd, *J* = 21.7, 10.8 Hz, 1H), 3.57 (s, 3H), 3.40 (s, 3H), 2.98 (d, *J* = 5.3 Hz, 1H), 2.20 (s, 3H), 2.08-2.02 (m, 1H), 1.75 (s, 3H), 1.52 (qd, *J* = 9.7, 9.2, 4.0 Hz, 2H), 1.38-1.28 (m, 1H), 1.15 (s, 3H), 0.93 (s, 3H).

^{13}C NMR (75 MHz, Chloroform-*d*) δ 160.79 (d, *J* = 2.5 Hz), 157.97 (d, *J* = 5.6 Hz), 142.57, 137.84 (d, *J* = 3.5 Hz), 137.57 (d, *J* = 2.3 Hz), 135.70 (d, *J* = 4.3 Hz), 130.49, 130.39, 128.57, 128.35, 128.08, 127.63, 126.75, 126.56, 113.29, 104.28 (d, *J* = 1.7 Hz), 101.19, 100.00 (d, *J* = 1.4 Hz), 98.62, 55.77 (d, *J* = 3.0 Hz), 55.23 (d, *J* = 6.9 Hz), 52.26 (d, *J* = 3.5 Hz), 48.32, 29.77, 26.64, 24.51, 21.63, 21.36, 9.97.

^{31}P NMR (121 MHz, Chloroform-*d*) δ 32.81.

HRMS (ESI-MS) calculated for $C_{38}H_{43}NO_7PS$ $[M+H]^+$: 688.2493, found: 688.2495.

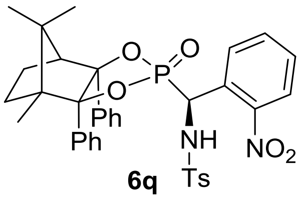

6q

4-Methyl-*N*-((1*S*)-(2-nitrophenyl)((3a*S*,4*S*,7*S*,7a*S*)-4,8,8-trimethyl-2-oxido-3a,7a-diphenylhexahydr-o-4,7-methanobenzo[*d*][1,3,2]dioxaphosphol-2-yl)methyl)benzenesulfonamide (**6q**).

$[\alpha]_D^{25}$ = +45.99 °(c = 1.00 in MeOH);

The residue was purified by flash chromatography (PE/EtOAc = 5:1) on silica gel to afford the product (242 mg, 90% yield), white solid. R_f = 0.36 (PE/EA = 5:1).

m.p. = 119.0-120.0 ℃

^{1}H NMR (300 MHz, Chloroform-*d*) δ 8.19 (d, *J* = 7.9 Hz, 1H), 8.03-7.94 (m, 1H), 7.88-7.46 (m, 3H), 7.31 (s, 5H), 7.17 (d, *J* = 7.9 Hz, 3H), 6.93 (t, *J* = 7.8 Hz, 2H), 6.84 (d, *J* = 8.1 Hz, 2H), 6.53 (d, *J* = 7.8 Hz, 1H), 5.92 (dd, *J* = 19.2, 9.6 Hz, 1H), 5.79 (dd, *J* = 9.6, 7.1 Hz, 1H), 3.01 (d, *J* = 5.3 Hz, 1H), 2.27 (s, 3H), 2.05 (ddd, *J* = 14.4, 9.7, 5.4 Hz, 1H), 1.71 (s, 3H), 1.63-1.47 (m, 1H), 1.27-1.19 (m, 2H), 1.13 (s, 3H), 0.71 (s, 3H).

^{13}C NMR (75 MHz, Chloroform-*d*) δ 147.77 (d, *J* = 9.0 Hz), 143.00, 137.33 (d, *J* = 3.5 Hz), 136.54, 135.32 (d, *J* = 4.6 Hz), 134.29, 132.03 (d, *J* = 4.1 Hz), 131.20, 129.51 (d, *J* = 1.6 Hz), 129.31, 128.64, 128.15, 128.03, 126.92, 126.12, 125.50, 102.84, 100.89, 55.64 (d, *J* = 2.9 Hz), 51.84 (d, *J* = 3.3 Hz), 48.01, 29.68, 26.64, 24.67, 21.52 (d, *J* = 14.0 Hz), 9.81.

^{31}P NMR (121 MHz, Chloroform-*d*) δ 33.31.

HRMS (ESI-MS) calculated for $C_{36}H_{38}N_2O_7PS$ $[M+H]^+$: 673.2132, found: 673.2135.

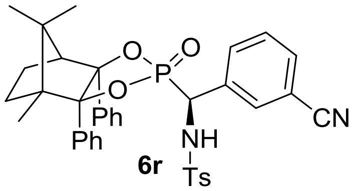

6r

***N*-((1*S*)-(3-Cyanophenyl)((3a*S*,4*S*,7*S*,7a*S*)-4,8,8-trimethyl-2-oxido-3a,7a-diphenylhexahy-dro-4,7-methanobenzo[*d*][1,3,2]dioxaphosphol-2-yl)methyl)-4-methylbenzenesulfonamide** (**6r**).

$[\alpha]_D^{25}$ = +103.98 °(c = 1.00 in MeOH);

The residue was purified by flash chromatography (PE/EtOAc = 5:1) on silica gel to afford the product (237 mg, 91% yield), white solid. R_f = 0.30 (PE/EA = 5:1).

m.p. = 115.0-116.5 ℃

^{1}H NMR (300 MHz, Chloroform-*d*) δ 7.70 (dd, *J* = 10.4, 4.0 Hz, 1H), 7.45 (h, *J* = 14.8, 13.8 Hz, 9H), 7.26 (s, 1H), 7.21 (d, *J* = 8.0 Hz, 2H), 7.13-7.04 (m, 1H), 6.85-6.66 (m, 4H), 6.27 (s, 1H), 4.82 (dd, *J* = 22.1, 10.3 Hz, 1H), 3.08 (d, *J* = 5.2 Hz, 1H), 2.17 (s, 3H), 2.13-2.07 (m, 1H), 1.86 (s, 3H), 1.70-1.58 (m, 1H), 1.59-1.37 (m, 2H), 1.20 (s, 3H), 1.05 (s, 3H).

^{13}C NMR (75 MHz, Chloroform- *d*) δ 142.83, 138.31 (d, *J* = 2.2 Hz), 137.71 (d, *J* = 3.0 Hz), 135.17 (d, *J* = 4.2 Hz), 134.29, 132.28 (d, *J* = 4.9 Hz), 131.68 (d, *J* = 6.2 Hz), 130.75, 130.52, 129.29, 128.98, 128.66, 128.53, 126.74, 111.74 (d, *J* = 2.3 Hz), 101.63, 100.66, 56.96, 56.22 (d, *J* = 3.1 Hz), 55.01, 52.42 (d, *J* = 3.6 Hz), 48.61, 29.76, 26.49, 24.15, 22.20, 21.32, 9.90.

^{31}P NMR (121 MHz, Chloroform-*d*) δ 30.76.

HRMS (ESI-MS) calculated for $C_{37}H_{38}N_2O_5PS$ $[M+H]^+$: 653.2234, found: 653.2233.

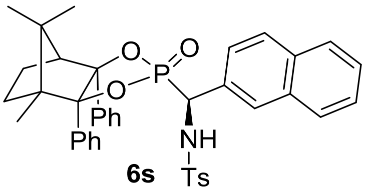

6s

4-Methyl-*N*-((1*S*)-naphthalen-2-yl((3a*S*,4*S*,7*S*,7a*S*)-4,8,8-trimethyl-2-oxido-3a,7a-diphenylhexahyd-ro-4,7-methanobenzo[*d*][1,3,2]dioxaphosphol-2-yl)methyl)benzenesulfonamide (**6s**).

$[\alpha]_D^{25}$ = +143.98 °(c = 1.00 in MeOH);

The residue was purified by flash chromatography (PE/EtOAc = 5:1) on silica gel to afford the product (257 mg, 95% yield), white solid. R_f = 0.40 (PE/EA = 5:1).

m.p. = 135.0-136.0 ℃

^{1}H NMR (300 MHz, Chloroform-*d*) δ 7.66 (dd, *J* = 10.5, 3.6 Hz, 1H), 7.49-7.27 (m, 10H), 7.18-6.97 (m, 5H), 6.87 (d, *J* = 8.0 Hz, 2H), 6.67 (d, *J* = 8.6 Hz, 1H), 6.56 (d, *J* = 2.6 Hz, 1H), 6.31 (d, *J* = 7.9 Hz, 2H), 4.90 (dd, *J* = 21.8, 10.5 Hz, 1H), 2.94 (d, *J* = 5.2 Hz, 1H), 1.99 (dd, *J* = 13.9, 5.3 Hz, 1H), 1.82 (s, 3H), 1.63 (s, 3H), 1.59-1.37 (m, 2H), 1.30 (td, *J* = 12.2, 11.6, 4.5 Hz, 1H), 1.07 (s, 3H), 0.97 (s, 3H).

^{13}C NMR (75 MHz, Chloroform-*d*) δ 141.95, 138.48 (d, *J* = 2.2 Hz), 137.99 (d, *J* = 3.2 Hz), 135.50 (d, *J* = 4.1 Hz), 132.31 (t, *J* = 2.1 Hz), 130.62, 129.61 (d, *J* = 1.9 Hz), 128.70, 128.30 (d, *J* = 3.4 Hz), 127.83, 127.54, 126.93, 126.68, 125.70, 125.54-125.12 (m), 101.06, 100.14, 57.97, 56.08 (d, J = 3.2 Hz), 52.38 (d, *J* = 3.5 Hz), 48.52, 31.59, 29.72, 26.49, 24.26, 22.21, 20.80, 14.19, 9.96.

^{31}P NMR (121 MHz, Chloroform-*d*) δ 31.88.

HRMS (ESI-MS) calculated for $C_{40}H_{41}NO_5PS$ $[M+H]^+$: 678.2438, found: 678.2438.

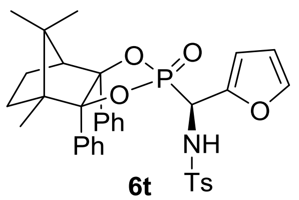

6t

***N*-((1*S*)-Furan-2-yl((3a*S*,4*S*,7*S*,7a*S*)-4,8,8-trimethyl-2-oxido-3a,7a-diphenylhexahydro-4,7-methano-benzo[*d*][1,3,2]dioxaphosphol-2-yl)methyl)-4-methylbenzenesulfonamide** (**6t**).

$[\alpha]_D^{25}$ = +120.98 °(c = 1.00 in MeOH);

The residue was purified by flash chromatography (PE/EtOAc = 5:1) on silica gel to afford the product (230 mg, 93% yield), white solid. R_f = 0.42 (PE/EA = 5:1).

m.p. = 116.0-117.0 ℃

^{1}H NMR (300 MHz, Chloroform-*d*) δ 7.52 – 7.14 (m, 12H), 7.04 (d, *J* = 8.0 Hz, 2H), 6.75 (s, 1H), 5.93 (dd, *J* = 10.4, 3.9 Hz, 1H), 5.77 – 5.70 (m, 1H), 5.35 (t, *J* = 3.2 Hz, 1H), 4.76 (dd, *J* = 21.1, 10.4 Hz, 1H), 2.98 (d, *J* = 5.3 Hz, 1H), 2.29 (s, 3H), 2.14 – 2.01 (m, 1H), 1.73 (s, 3H), 1.60 – 1.45 (m, 2H), 1.40 – 1.29 (m, 1H), 1.16 (s, 3H), 0.90 (s, 3H).

^{13}C NMR (75 MHz, Chloroform-*d*) δ 145.33, 142.69, 142.19 (d, *J* = 2.9 Hz), 137.70 (d, *J* = 1.9 Hz), 137.53 (d, *J* = 3.3 Hz), 135.10 (d, *J* = 4.0 Hz), 130.17, 129.05, 128.35, 127.71, 126.81, 110.14 (d, *J* = 2.2 Hz), 109.74 (d, *J* = 6.6 Hz), 101.35, 99.93, 55.87 (d, *J* = 3.4 Hz), 52.08 (d, *J* = 3.3 Hz), 50.02, 48.36, 29.47, 26.42, 24.45, 21.76, 21.44, 9.95.

^{31}P NMR (121 MHz, Chloroform-*d*) δ 29.25.

HRMS (ESI-MS) calculated for $C_{34}H_{37N}O_6PS$ $[M+H]^+$: 618.2074, found: 618.2075.

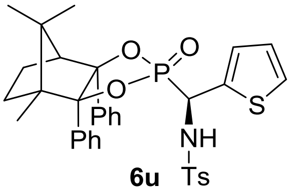

6u

4-Methyl-*N*-((1*S*)-thiophen-2-yl((3a*S*,4*S*,7*S*,7a*S*)-4,8,8-trimethyl-2-oxido-3a,7a-diphenylhexahydro-4,7-methanobenzo[*d*][1,3,2]dioxaphosphol-2-yl)methyl)benzenesulfonamide (**6u**).

$[\alpha]_D^{25}$ = +112.98 °(c = 1.00 in MeOH);

The residue was purified by flash chromatography (PE/EtOAc = 5:1) on silica gel to afford the product (233 mg, 92% yield), white solid. R_f = 0.38 (PE/EA = 5:1).

m.p. = 146.0-147.5 ℃

^{1}H NMR (300 MHz, Chloroform-*d*) δ 7.61-7.14 (m, 13H), 6.93 (d, *J* = 7.9 Hz, 2H), 6.72 (d, *J* = 5.0 Hz, 1H), 6.46 (d, *J* = 3.5 Hz, 1H), 6.29 (t, *J* = 4.4 Hz, 1H), 5.04 (dd, *J* = 20.9, 10.2 Hz, 1H), 3.06 (d, *J* = 5.2 Hz, 1H), 2.24 (s, 3H), 2.10 (dt, *J* = 13.4, 7.8 Hz, 1H), 1.86 (s, 3H), 1.57 (q, *J* = 6.0, 5.0 Hz, 2H), 1.47-1.27 (m, 1H), 1.19 (s, 3H), 1.00 (s, 3H).

^{13}C NMR (75 MHz, Chloroform- *d*) δ 142.24, 138.57 (d, *J* = 2.2 Hz), 137.73 (d, *J* = 3.2 Hz), 135.26 (d, *J* = 4.1 Hz), 134.31, 130.44, 128.75, 128.54 (d, *J* = 2.5 Hz), 128.43, 128.24, 128.08, 126.79, 126.18 (d, *J* = 2.5 Hz), 125.69 (d, *J* = 3.4 Hz), 101.21, 100.01, 55.93 (d, *J* = 3.1 Hz), 53.69, 52.16 (d, *J* = 3.3 Hz), 51.64, 48.40, 29.63, 26.47, 24.31, 22.02, 21.35, 9.92.

^{31}P NMR (121 MHz, Chloroform-*d*) δ 30.36.

HRMS (ESI-MS) calculated for $C_{34}H_{37}NO_5PS_2$ $[M+H]^+$: 634.1846, found: 634.1845.

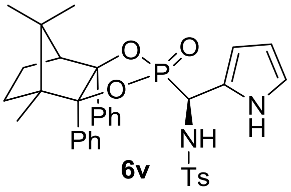

6v

***N*-((1*S*)-(1H-pyrrol-2-yl)((3a*S*,4*S*,7*S*,7a*S*)-4,8,8-trimethyl-2-oxido-3a,7a-diphenylhexahydro-4,7-methanobenzo[*d*][1,3,2]dioxaphosphol-2-yl)methyl)-4-methylbenzenesulfonamide** (**6v**).

$[\alpha]_D^{25}$ = +115.97 °(c = 1.00 in MeOH);

The residue was purified by flash chromatography (PE/EtOAc = 5:1) on silica gel to afford the product (212 mg, 86% yield), white solid. R_f = 0.40 (PE/EA = 5:1).

m.p. = 158.0-160.0 ℃

^{1}H NMR (300 MHz, Chloroform-*d*) δ 8.87 (s, 1H), 7.58 (t, *J* = 9.4 Hz, 3H), 7.48-7.22 (m, 10H), 6.81 (d, *J* = 8.0 Hz, 2H), 5.91-5.83 (m, 1H), 5.37 (t, *J* = 2.9 Hz, 1H), 5.05-4.88 (m, 1H), 4.44 (s, 1H), 3.06 (d, *J* = 5.2 Hz, 1H), 2.17 (s, 3H), 2.12-1.99 (m, 1H), 1.80 (s, 3H), 1.66-1.49 (m, 2H), 1.41 (dt, *J* = 18.8, 5.7 Hz, 1H), 1.17 (s, 3H), 1.04 (s, 3H).

^{13}C NMR (75 MHz, Chloroform-*d*) δ 142.14, 137.85 (dd, *J* = 13.4, 2.8 Hz), 135.02 (d, *J* = 4.1 Hz), 130.73, 129.88, 129.15, 128.89, 128.69, 128.35, 126.73, 121.20 (d, *J* = 4.1 Hz), 119.28, 108.05 (d, *J* = 8.5 Hz), 107.34, 101.92, 100.19, 56.17 (d, *J* = 3.0 Hz), 52.43, 52.28 (d, *J* = 3.7 Hz), 50.35, 48.51, 29.82, 26.54, 24.26, 22.30, 21.40, 10.04.

^{31}P NMR (121 MHz, Chloroform-*d*) δ 31.53.

HRMS (ESI-MS) calculated for $C_{34}H_{38}N_2O_5PS$ $[M+H]^+$: 617.2234, found: 617.2236.

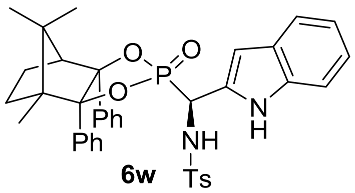

6w

***N*-((1*S*)-(1H-indol-2-yl)((3a*S*,4*S*,7*S*,7a*S*)-4,8,8-trimethyl-2-oxido-3a,7a-diphenylhexahydro-4,7-methanobenzo[*d*][1,3,2]dioxaphosphol-2-yl)methyl)-4-methylbenzenesulfonamide** (**6w**).

$[\alpha]_D^{25}$ = +95.99 °(c = 1.00 in MeOH);

The residue was purified by flash chromatography (PE/EtOAc = 5:1) on silica gel to afford the product (256 mg, 96% yield), white solid. R_f = 0.42 (PE/EA = 5:1).

m.p. = 171.0-172.0 ℃

^{1}H NMR (300 MHz, Chloroform-*d*) δ 8.78 (d, *J* = 2.2 Hz, 1H), 7.72 – 7.09 (m, 13H), 6.93 (d, *J* = 7.9 Hz, 1H), 6.65 (t, *J* = 7.5 Hz, 1H), 6.47 (dd, *J* = 7.8, 3.4 Hz, 3H), 5.97 (d, *J* = 8.2 Hz, 1H), 5.05 (dd, *J* = 22.0, 11.0 Hz, 1H), 4.86 (t, *J* = 2.7 Hz, 1H), 3.08 (d, *J* = 5.2 Hz, 1H), 2.18 – 2.02 (m, 1H), 1.84 (s, 3H), 1.74 (s, 3H), 1.67 – 1.51 (m, 2H), 1.49 – 1.34 (m, 1H), 1.17 (s, 3H), 1.07 (s, 3H).

^{13}C NMR (75 MHz, Chloroform-*d*) δ 142.49, 137.60 (d, *J* = 3.2 Hz), 137.25 (d, *J* = 2.2 Hz), 136.07, 134.91 (d, *J* = 4.1 Hz), 130.58, 128.96, 128.37, 128.13, 127.63 (d, *J* = 4.2 Hz), 126.82 (d, *J* =

2.1 Hz), 126.52, 121.36, 119.41, 118.86, 111.09, 102.91 (d, *J* = 9.7 Hz), 102.03, 100.51, 56.15 (d, *J* = 3.2 Hz), 52.85, 52.27, 50.79, 48.43, 29.71, 26.41, 24.19, 22.28, 20.87, 9.91.

^{31}P NMR (121 MHz, Chloroform-*d*) δ 31.18.

HRMS (ESI-MS) calculated for $C_{38}H_{40}N_2O_5PS$ $[M+H]^+$: 667.2391, found: 667.2392.

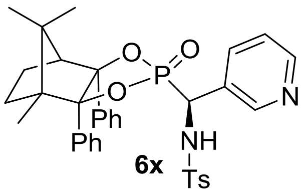

6x

4-Methyl-*N*-((1*S*)-pyridin-3-yl((3a*S*,4*S*,7*S*,7a*S*)-4,8,8-trimethyl-2-oxido-3a,7a-diphenylhexahydro-4,7-methanobenzo[*d*][1,3,2]dioxaphosphol-2-yl)methyl)benzenesulfonamide (**6x**).

$[\alpha]_D^{25}$ = +114.96 °(c = 1.00 in MeOH);

The residue was purified by flash chromatography (PE/EtOAc = 3:1) on silica gel to afford the product (226 mg, 90% yield), white solid. R_f = 0.40 (PE/EA = 3:1).

m.p. = 127.0-128.0 ℃

^{1}H NMR (300 MHz, Chloroform-*d*) δ 8.08-8.00 (m, 1H), 7.91 (dd, *J* = 10.6, 3.2 Hz, 1H), 7.42 (d, *J* = 6.4 Hz, 9H), 7.31-7.15 (m, 5H), 6.80 (d, *J* = 8.1 Hz, 2H), 6.51 (dd, *J* = 7.9, 4.8 Hz, 1H), 4.88 (dd, *J* = 21.9, 10.5 Hz, 1H), 3.06 (d, *J* = 5.2 Hz, 1H), 2.13 (s, 3H), 1.88 (s, 3H), 1.59 (ddq, *J* = 26.3, 13.6, 7.9, 6.9 Hz, 2H), 1.41 (td, *J* = 12.5, 4.5 Hz, 1H), 1.19 (s, 3H), 1.05 (s, 3H).

^{13}C NMR (75 MHz, Chloroform-*d*) δ 148.89 (d, *J* = 7.0 Hz), 148.24, 142.38, 138.23 (d, *J* = 2.2 Hz), 137.50 (d, *J* = 3.2 Hz), 135.71 (d, *J* = 4.3 Hz), 135.15 (d, *J* = 4.1 Hz), 130.45, 129.11, 128.85 (d, *J* = 3.4 Hz), 128.31, 126.66, 122.77, 101.42, 100.52, 56.04 (d, *J* = 3.2 Hz), 55.42, 53.44, 52.25 (d, *J* = 3.4 Hz), 48.45, 29.68, 26.44, 24.16, 22.11, 21.19, 9.82.

^{31}P NMR (121 MHz, Chloroform-*d*) δ 31.26.

HRMS (ESI-MS) calculated for $C_{35}H_{38}N_2O_5PS$ $[M+H]^+$: 629.2234, found: 629.2234.

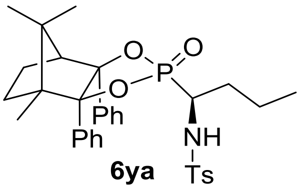

6ya

4-Methyl-*N*-((1*S*)-1-((3a*S*,4*S*,7*S*,7a*S*)-4,8,8-trimethyl-2-oxido-3a,7a-diphenylhexahydro-4,7-met-hanobenzo[*d*][1,3,2]dioxaphosphol-2-yl)butyl)benzenesulfonamide (6ya).

$[\alpha]_D^{25}$ = +47.99 °(c = 1.00 in MeOH);

The residue was purified by flash chromatography (PE/EtOAc = 5:1) on silica gel to afford the product (190 mg, 80% yield), white solid. R_f = 0.40 (PE/EA = 5:1).

m.p. = 147.0-148.0 ℃

^{1}H NMR (300 MHz, Chloroform-*d*) δ 7.88 (d, *J* = 7.9 Hz, 2H), 7.62-7.50 (m, 1H), 7.47-7.22 (m, 6H), 7.19-7.03 (m, 3H), 6.71 (d, *J* = 80.9 Hz, 3H), 5.87 (dd, *J* = 9.8, 4.8 Hz, 1H), 3.50 (p, *J* = 7.5 Hz, 1H), 2.98 (d, *J* = 5.3 Hz, 1H), 2.45 (s, 3H), 2.06 (dd, *J* = 15.0, 7.5 Hz, 1H), 1.53 (dt, *J* = 14.7, 7.2 Hz, 1H), 1.32-1.23 (m, 2H), 1.14 (s, 3H), 1.03 (dt, *J* = 22.8, 10.4 Hz, 4H), 0.75 (s, 3H), 0.44 (t, *J* = 7.0 Hz, 3H).

^{13}C NMR (75 MHz, Chloroform-*d*) δ 143.22, 139.26, 137.55 (d, *J* = 3.4 Hz), 135.51 (d, *J* = 4.3 Hz), 129.55, 128.48, 128.04, 126.89, 101.06, 99.21, 55.54 (d, *J* = 2.9 Hz), 53.98, 52.11, 48.29, 32.73, 29.68, 26.48, 24.40, 21.66 (d, *J* = 6.1 Hz), 18.43 (d, *J* = 10.4 Hz), 13.43, 9.61.

^{31}P NMR (121 MHz, Chloroform-*d*) δ 37.69.

HRMS (ESI-MS) calculated for $C_{33}H_{41}NO_5PS$ $[M+H]^+$: 594.2438, found: 594.2441.

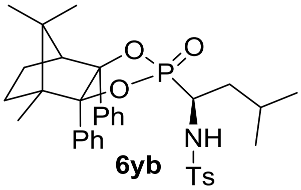

6yb

4-Methyl-*N*-((1*S*)-3-methyl-1-((3a*S*,4*S*,7*S*,7a*S*)-4,8,8-trimethyl-2-oxido-3a,7a-diphenylhex-ahydro-4,7-methanobenzo[*d*][1,3,2]dioxaphosphol-2-yl)butyl)benzenesulfonamide (**6yb**).

$[\alpha]_D^{25}$ = +50.99 °(c = 1.00 in MeOH);

The residue was purified by flash chromatography (PE/EtOAc = 5:1) on silica gel to afford the product (182 mg, 75% yield), white solid. R_f = 0.40 (PE/EA = 5:1).

m.p. = 167.0-169.0 ℃

^{1}H NMR (300 MHz, Chloroform-*d*) δ 7.88 (d, *J* = 8.3 Hz, 2H), 7.64-6.59 (m, 12H), 5.90 (dd, *J* = 9.8, 3.5 Hz, 1H), 3.76-3.48 (m, 1H), 2.98 (d, *J* = 5.2 Hz, 1H), 2.44 (s, 3H), 2.04 (td, *J* = 12.6, 10.6, 5.2 Hz, 1H), 1.77 (s, 3H), 1.51 (dq, *J* = 11.8, 6.1, 4.9 Hz, 2H), 1.26 (t, *J* = 7.4 Hz, 3H), 1.14 (s, 3H), 0.99-0.86 (m, 1H), 0.75 (s, 3H), 0.52 (d, *J* = 6.5 Hz, 3H), 0.46 (d, *J* = 6.6 Hz, 3H).

^{13}C NMR (75 MHz, Chloroform-*d*) δ 143.13, 139.34, 137.64 (d, *J* = 3.3 Hz), 135.58 (d, *J* = 4.5 Hz), 131.71, 129.49, 127.97, 127.61 (d, *J* = 112.0 Hz), 101.29, 99.41 (d, *J* = 1.6 Hz), 55.55 (d, *J* = 2.8 Hz), 52.71, 51.82 (d, *J* = 3.1 Hz), 50.81, 48.25, 39.33 (d, *J* = 2.7 Hz), 29.83, 26.60, 24.46, 23.80, 23.65, 22.87, 21.67 (d, *J* = 7.3 Hz), 21.04, 9.59.

^{31}P NMR (121 MHz, Chloroform-*d*) δ 38.63.

HRMS (ESI-MS) calculated for $C_{34}H_{43}NO_5PS$ $[M+H]^+$: 608.2595, found:608.2595.

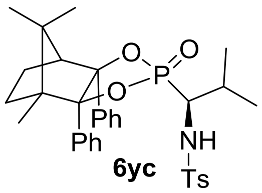

6yc

4-Methyl-*N*-((1*S*)-2-methyl-1-((3a*S*,4*S*,7*S*,7a*S*)-4,8,8-trimethyl-2-oxido-3a,7a-diphenyl-hexahydro-4,7-methanobenzo[*d*][1,3,2]dioxaphosphol-2-yl)propyl)benzenesulfonamide (**6yc**).

$[\alpha]_D^{25}$ = +2.00 °(c = 1.00 in MeOH);

The residue was purified by flash chromatography (PE/EtOAc = 5:1) on silica gel to afford the product (183 mg, 77% yield), white solid. R_f = 0.40 (PE/EA = 5:1).

m.p. = 197.0-198.0 ℃

^{1}H NMR (300 MHz, Chloroform-*d*) δ 7.92 (d, *J* = 8.0 Hz, 2H), 7.54 (d, *J* = 7.8 Hz, 1H), 7.43 (d, *J* = 8.0 Hz, 3H), 7.34-7.17 (m, 3H), 7.00 (t, *J* = 8.0 Hz, 3H), 6.43 (d, *J* = 8.0 Hz, 2H), 5.49 (dd, *J* = 9.6, 7.4 Hz, 1H), 3.36 (ddd, *J* = 15.0, 9.7, 2.2 Hz, 1H), 2.98 (d, *J* = 5.3 Hz, 1H), 2.46 (s, 3H), 2.13-1.98 (m, 1H), 1.74 (s, 3H), 1.52 (ddd, *J* = 14.5, 8.5, 5.4 Hz, 1H), 1.36-1.30 (m, 1H), 1.24 (dt, *J* = 9.1, 4.8 Hz, 2H), 1.14 (s, 3H), 0.71 (t, *J* = 3.5 Hz, 6H), 0.63 (dd, *J* = 6.7, 2.1 Hz, 3H).

^{13}C NMR (75 MHz, Chloroform-*d*) δ 143.44, 138.89, 137.22 (d, *J* = 3.4 Hz), 135.66 (d, *J* = 4.5 Hz), 131.66, 129.60, 128.69, 128.08, 127.05, 126.47, 100.87, 99.42 (d, *J* = 1.6 Hz), 58.57, 56.76, 55.46 (d, *J* = 2.8 Hz), 51.72 (d, *J* = 3.3 Hz), 48.31, 31.68, 29.73, 28.57 (d, *J* = 4.9 Hz), 26.55, 24.46, 22.75, 21.67 (d, *J* = 3.3 Hz), 20.05, 19.83, 16.30 (d, *J* = 1.6 Hz), 14.24, 9.53.

^{31}P NMR (121 MHz, Chloroform-*d*) δ 37.85.

HRMS (ESI-MS) calculated for $C_{33}H_{41}NO_5PS$ $[M+H]^+$: 594.2438, found: 594.2444.

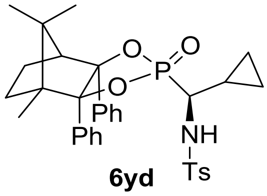

6yd

***N*-((1*S*)-Cyclopropyl((3a*S*,4*S*,7*S*,7a*S*)-4,8,8-trimethyl-2-oxido-3a,7a-diphenylhexahydro-4,7-methan-obenzo[*d*][1,3,2]dioxaphosphol-2-yl)methyl)-4-methylbenzenesulfonamide (6yd)**.

$[\alpha]_D^{25}$ = +61.99 °(c = 1.00 in MeOH);

The residue was purified by flash chromatography (PE/EtOAc = 5:1) on silica gel to afford the product (201 mg, 85% yield), white solid. R_f = 0.40 (PE/EA = 5:1).

m.p. = 108.0-110.0 ℃

^{1}H NMR (300 MHz, Chloroform-*d*) δ 7.88 (d, *J* = 8.0 Hz, 2H), 7.72-6.69 (m, 12H), 6.30 (dd, *J* = 9.7, 3.9 Hz, 1H), 3.30 (dt, *J* = 15.9, 9.0 Hz, 1H), 3.09 (d, *J* = 5.3 Hz, 1H), 2.47 (s, 3H), 2.11 (q, *J* = 10.1, 9.5 Hz, 1H), 1.85 (s, 3H), 1.65-1.47 (m, 1H), 1.46-1.30 (m, 2H), 1.21 (s, 3H), 0.89 (s, 3H), 0.68 (dq, *J* = 8.6, 4.4 Hz, 1H), 0.10-0.27 (m, 3H), -0.68 (dq, *J* = 9.9, 5.1 Hz, 1H).

^{13}C NMR (75 MHz, Chloroform-*d*) δ 142.83, 140.08 (d, *J* = 2.0 Hz), 137.76 (d, *J* = 3.0 Hz), 135.54 (d, *J* = 4.2 Hz), 130.30, 129.36, 128.66, 128.00, 126.93, 100.79, 99.48, 56.95, 55.69 (d, *J* = 2.9 Hz), 54.97, 52.00 (d, *J* = 3.6 Hz), 48.39, 29.86, 26.51, 24.23, 21.87, 21.60, 11.93 (d, *J* = 3.3 Hz), 9.70, 4.78 (d, *J* = 12.1 Hz), 2.03 (d, *J* = 3.2 Hz).

^{31}P NMR (121 MHz, Chloroform-*d*) δ 35.13.

HRMS (ESI-MS) calculated for $C_{33}H_{39}NO_5PS$ $[M+H]^+$: 592.2282, found: 592.2281.

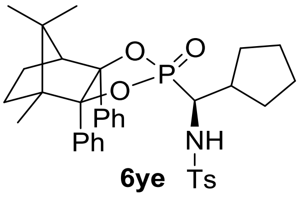

6ye

***N*-((1*S*)-Cyclopentyl((3a*S*,4*S*,7*S*,7a*S*)-4,8,8-trimethyl-2-oxido-3a,7a-diphenylhexahydro-4,7-methan-obenzo[*d*][1,3,2]dioxaphosphol-2-yl)methyl)-4-methylbenzenesulfonamide (6ye)**.

$[\alpha]_D^{25}$ = +60.00 °(c = 1.00 in MeOH);

The residue was purified by flash chromatography (PE/EtOAc = 5:1) on silica gel to afford the product (218 mg, 88% yield), white solid. R_f = 0.40 (PE/EA = 5:1).

m.p. = 121.0-122.0 ℃

^{1}H NMR (300 MHz, Chloroform-*d*) δ 7.92 (d, *J* = 8.0 Hz, 2H), 7.53 (d, *J* = 7.8 Hz, 1H), 7.43 (d, *J* = 8.1 Hz, 3H), 7.29-7.23 (m, 2H), 7.01 (t, *J* = 8.0 Hz, 3H), 6.48 (d, *J* = 7.7 Hz, 3H), 5.58 (t, *J* = 8.2 Hz, 1H), 3.56 (dd, *J* = 14.5, 9.5 Hz, 1H), 2.98 (d, *J* = 5.3 Hz, 1H), 2.47 (s, 3H), 2.14-1.97 (m, 1H), 1.74 (s, 3H), 1.58-1.47 (m, 1H), 1.43-1.29 (m, 5H), 1.26-1.11 (m, 9H), 0.71 (s, 3H).

^{13}C NMR (75 MHz, Chloroform-*d*) δ 143.39, 139.01, 137.22 (d, *J* = 3.3 Hz), 135.67 (d, *J* = 4.5 Hz), 131.70, 129.58, 128.76 (d, *J* = 21.2 Hz), 128.02, 126.97, 126.40, 100.92, 99.29, 55.61-55.05 (m), 53.42, 51.72 (d, *J* = 3.3 Hz), 48.31, 39.98 (d, *J* = 3.8 Hz), 29.75, 28.69 (d, *J* = 14.2 Hz), 26.41 (d, *J* = 16.7 Hz), 25.24, 24.75, 24.40, 21.68 (d, *J* = 4.9 Hz), 9.51.

^{31}P NMR (121 MHz, Chloroform-*d*) δ 37.81.

HRMS (ESI-MS) calculated for $C_{35}H_{43}NO_5PS$ $[M+H]^+$:620.2595, found:620.2601.

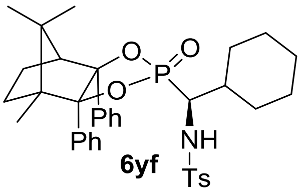

6yf

***N*-((1*S*)-Cyclohexyl((3a*S*,4*S*,7*S*,7a*S*)-4,8,8-trimethyl-2-oxido-3a,7a-diphenylhexahydro-4,7-methan-obenzo[*d*][1,3,2]dioxaphosphol-2-yl)methyl)-4-methylbenzenesulfonamide** (**6yf**).

$[\alpha]_D^{25}$ = +25.99 °(c = 1.00 in MeOH);

The residue was purified by flash chromatography (PE/EtOAc = 5:1) on silica gel to afford the product (215 mg, 85% yield), white solid. R_f = 0.38 (PE/EA = 5:1).

m.p. = 129.0-130.0 ℃

^{1}H NMR (300 MHz, Chloroform-*d*) δ 7.93 (d, *J* = 8.2 Hz, 2H), 7.54 (d, *J* = 7.9 Hz, 1H), 7.45 (d, *J* = 8.0 Hz, 3H), 7.26 (d, *J* = 6.2 Hz, 3H), 7.07 (s, 1H), 7.02-6.92 (m, 2H), 6.64-6.51 (m, 1H), 6.45-6.37 (m, 1H), 5.53 (dd, *J* = 9.7, 7.5 Hz, 1H), 3.27 (ddd, *J* = 15.1, 9.7, 2.0 Hz, 1H), 2.99 (d, *J* = 5.3 Hz, 1H), 2.47 (s, 3H), 2.05 (dt, *J* = 19.9, 5.4 Hz, 1H), 1.72 (s, 3H), 1.49 (td, *J* = 9.7, 8.9, 4.6 Hz, 4H), 1.27 (dtd, *J* = 18.8, 14.8, 13.8, 10.9 Hz, 5H), 1.13 (s, 3H), 1.07 (d, *J* = 11.9 Hz, 2H), 0.89 (dtd, *J* = 18.1, 12.6, 3.2 Hz, 3H), 0.69 (s, 3H).

^{13}C NMR (75 MHz, Chloroform-*d*) δ 143.44, 138.80, 137.09 (d, *J* = 3.3 Hz), 135.72 (d, *J* = 4.4 Hz), 129.58, 128.60, 128.07, 127.09, 100.75, 99.34 (d, *J* = 1.5 Hz), 58.65, 56.87, 55.43 (d, *J* = 2.8 Hz), 51.70 (d, *J* = 3.4 Hz), 48.33, 38.29 (d, *J* = 4.6 Hz), 30.06, 29.85, 29.73, 26.51, 26.33, 25.82, 25.39, 24.33, 21.67 (d, *J* = 3.0 Hz), 9.45.

^{31}P NMR (121 MHz, Chloroform-*d*) δ 37.90.

HRMS (ESI-MS) calculated for $C_{36}H_{45}NO_5PS$ $[M+H]^+$: 634.2751, found: 634.2745.

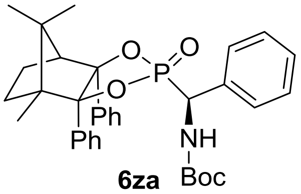

6za

***Tert*-butyl((1*S*)-phenyl((3a*S*,4*S*,7*S*,7a*S*)-4,8,8-trimethyl-2-oxido-3a,7a-diphenylhexahydro-4,7-meth-anobenzo[*d*][1,3,2]dioxaphosphol-2-yl)methyl)carbamate** (**6za**).

$[\alpha]_D^{25}$ = +141.99 °(c = 1.00 in MeOH);

The residue was purified by flash chromatography (PE/EtOAc = 8:1) on silica gel to afford the product (126 mg, 55% yield), white solid. R_f = 0.30 (PE/EA = 8:1).

m.p. = 135.0-136.0 ℃

^{1}H NMR (300 MHz, Chloroform-*d*) δ 7.37-7.31 (m, 2H), 7.31 (s, 1H), 7.27 (s, 3H), 7.22 (d, *J* = 6.4 Hz, 4H), 7.03 (q, *J* = 6.9, 6.4 Hz, 3H), 6.91 (d, *J* = 7.2 Hz, 2H), 5.73 (dd, *J* = 10.1, 4.8 Hz, 1H), 4.96 (dd, *J* = 18.2, 10.1 Hz, 1H), 3.00 (d, *J* = 5.4 Hz, 1H), 2.15-1.98 (m, 1H), 1.75 (s, 3H), 1.48 (s, 9H), 1.31 (ddd, *J* = 19.7, 8.3, 5.1 Hz, 2H), 1.22 (s, 1H), 1.15 (s, 3H), 0.89 (s, 3H).

^{13}C NMR (75 MHz, Chloroform-*d*) δ 154.40 (d, *J* = 11.3 Hz), 137.67 (d, *J* = 3.4 Hz), 135.85 (d, *J* = 4.2 Hz), 135.13, 130.02, 128.54, 128.33 (d, *J* = 2.0 Hz), 127.92 (d, *J* = 6.5 Hz), 126.52, 100.65, 100.16, 80.25, 55.81 (d, *J* = 3.2 Hz), 54.44, 52.51, 52.18 (d, *J* = 3.2 Hz), 48.39, 29.71, 28.44, 26.56, 24.49, 21.63, 10.03.

^{31}P NMR (121 MHz, Chloroform-*d*) δ 34.65.

HRMS (ESI-MS) calculated for $C_{34}H_{41}NO_5P$ $[M+H]^+$: 574.2717; found: 574.2717.

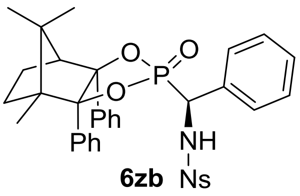

6zb

4-Nitro-*N*-((1*S*)-phenyl((3a*S*,4*S*,7*S*,7a*S*)-4,8,8-trimethyl-2-oxido-3a,7a-diphenylhexahydro-4,7-meth-anobenzo[*d*][1,3,2]dioxaphosphol-2-yl)methyl)benzenesulfonamide (**6zb**).

$[\alpha]_D^{25}$ = +161.97 °(c = 1.00 in MeOH);

The residue was purified by flash chromatography (PE/EtOAc = 5:1) on silica gel to afford the product (250 mg, 95% yield), white solid. R_f = 0.40 (PE/EA = 5:1).

m.p. = 155.0-156.0 ℃

^{1}H NMR (300 MHz, Chloroform-*d*) δ 8.60 (dd, *J* = 10.3, 3.6 Hz, 1H), 7.72 (d, *J* = 8.5 Hz, 2H), 7.57 (s, 1H), 7.47 (d, *J* = 9.4 Hz, 6H), 7.34 (d, *J* = 43.0 Hz, 5H), 6.79 (t, *J* = 7.4 Hz, 1H), 6.54 (t, *J* = 7.6 Hz, 2H), 6.39 (d, *J* = 7.7 Hz, 2H), 4.92 (dd, *J* = 22.1, 10.3 Hz, 1H), 3.12 (d, *J* = 5.2 Hz, 1H), 2.10 (dt, *J* = 16.7, 11.5 Hz, 1H), 1.93 (s, 3H), 1.72-1.60 (m, 1H), 1.52 (ddd, *J* = 30.3, 15.4, 4.9 Hz, 2H), 1.22 (s, 3H), 1.11 (s, 3H).

^{13}C NMR (75 MHz, Chloroform-*d*) δ 148.90, 147.43 (d, *J* = 2.8 Hz), 137.64 (d, *J* = 3.1 Hz), 135.30 (d, *J* = 4.1 Hz), 131.90 (d, *J* = 1.8 Hz), 130.58, 128.98, 128.68, 128.39, 128.22 (d, *J* = 5.4 Hz), 127.97, 127.75 (t, *J* = 3.6 Hz), 126.78, 123.14, 101.41, 100.74, 57.73, 56.18 (d, *J* = 3.1 Hz), 55.77, 52.42 (d, *J* = 3.5 Hz), 48.55, 29.70, 26.50, 24.25, 22.34, 9.92.

^{31}P NMR (121 MHz, Chloroform-*d*) δ 31.58.

HRMS (ESI-MS) calculated for $C_{35}H_{36}N_2O_7PS$ $[M+H]^+$: 659.1976, found: 659.1979.

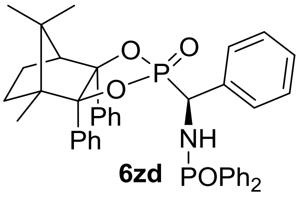

6zd

***P*,*P*-Diphenyl-*N*-((1*S*)-phenyl((3a*S*,4*S*,7*S*,7a*S*)-4,8,8-trimethyl-2-oxido-3a,7a-diphenylhe-xahydro-4,7-methanobenzo[*d*][1,3,2]dioxaphosphol-2-yl)methyl)phosphinic amide** (**6zd**).

$[\alpha]_D^{25}$ = +179.96 °(c = 1.00 in MeOH);

The residue was purified by flash chromatography (PE/EtOAc =3:1) on silica gel to afford the product (188 mg, 70% yield), white solid. R_f = 0.40 (PE/EA = 3:1).

m.p. = 147.0-149.0 ℃

^{1}H NMR (300 MHz, Chloroform-*d*) δ 7.76-7.11 (m, 18H), 7.11-6.85 (m, 5H), 6.75-6.65 (m, 2H), 4.38-4.10 (m, 2H), 2.92 (d, *J* = 5.3 Hz, 1H), 2.00 (t, *J* = 6.5 Hz, 1H), 1.74 (s, 3H), 1.45 (dt, *J* = 14.3, 6.8 Hz, 1H), 1.29 (t, *J* = 7.5 Hz, 2H), 1.13 (s, 3H), 0.92 (s, 3H).

^{13}C NMR (75 MHz, Chloroform-*d*) δ 137.22, 137.18, 136.60, 136.54, 136.06, 136.04, 133.04, 132.59, 132.46, 132.29, 132.22-131.93 (m), 131.33, 130.69, 130.52, 128.72, 128.53 (d, *J* = 3.1 Hz), 128.49-128.38 (m), 128.28, 128.09 (d, *J* = 1.6 Hz), 127.88 (d, *J* = 2.4 Hz), 126.62, 101.06, 99.87 (d, *J* = 1.5 Hz), 55.97, 55.83 (d, *J* = 2.7 Hz), 54.05, 51.93 (d, *J* = 3.5 Hz), 48.27, 30.04, 26.62, 24.26, 21.67, 9.86.

^{31}P NMR (121 MHz, Chloroform-*d*) δ 36.18, 35.87, 24.13 (d, *J* = 2.2 Hz), 23.82 (d, *J* = 2.2 Hz).

HRMS (ESI-MS) calculated for $C_{41}H_{42}NO_4P_2$ $[M+H]^+$:674.2584 found:674.2580.

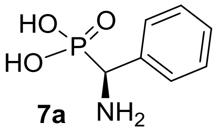

7a

(*S*)-(Amino(phenyl)methyl)phosphonic acid[8] (**7a**).

$[\alpha]_D^{25}$ = -8.00 °(c = 1.00 in MeOH);

72 mg, 95% yield, white solid.

m.p. = 262.0-263.0 ℃.

^{1}H NMR (300 MHz, Deuterium Oxide) δ 7.24 (s, 5H), 4.30 (d, *J* = 16.5 Hz, 1H).

^{13}C NMR (75 MHz, Deuterium Oxide) δ 131.70 (d, *J* = 4.9 Hz), 128.99 (d, *J* = 1.8 Hz), 127.61 (d, *J* = 5.2 Hz), 53.73, 51.84.

^{31}P NMR (121 MHz, Deuterium Oxide) δ 10.33.

HRMS (ESI-MS) calculated for $C_7H_{11}NO_3P$ $[M+H]^+$: 188.0477, found: 188.0479.

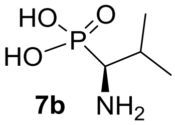

7b

(*S*)-(1-Amino-2-methylpropyl)phosphonic acid[9-13] (**7b**).

$[\alpha]_D^{25}$ = -22.00 °(c = 1.00 in MeOH);

58 mg, 95% yield, white solid.

m.p. = 285.0-286.0 ℃.

^{1}H NMR (300 MHz, Deuterium Oxide) δ 3.05 (dd, *J* = 14.2, 6.0 Hz, 1H), 2.16 (dq, *J* = 13.0, 6.6 Hz, 1H), 1.03 (dd, *J* = 11.9, 6.7 Hz, 6H).

^{13}C NMR (75 MHz, Deuterium Oxide) δ 55.69, 53.81, 27.46, 19.65 (d, *J* = 7.2 Hz), 17.75 (d, *J* = 6.2 Hz).

^{31}P NMR (121 MHz, Deuterium Oxide) δ 11.60.

HRMS (ESI-MS) calculated for $C_4H_{11}O_3P$ $[M-H]^-$: 152.0477, found: 152.0480.

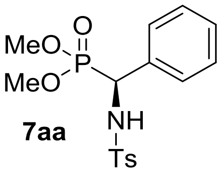

7aa

dimethyl (S)-(((4-methylphenyl)sulfonamido)(phenyl)methyl)phosphonate (7aa)

140 mg, 95% yiedld, white solid.

^{1}H NMR (300 MHz, Chloroform-*d*) δ 7.73 (dd, *J* = 10.2, 3.9 Hz, 1H), 7.47 (d, *J* = 7.9 Hz, 2H), 7.28 – 7.19 (m, 2H), 7.17 – 7.00 (m, 3H), 6.93 (d, *J* = 7.9 Hz, 2H), 4.87 (dd, *J* = 24.4, 10.1 Hz, 1H), 3.91 (d, *J* = 10.8 Hz, 3H), 3.40 (d, *J* = 10.6 Hz, 3H), 2.23 (s, 3H).

^{13}C NMR (75 MHz, Chloroform-*d*) δ 142.70, 138.03 (d, *J* = 1.9 Hz), 133.38, 128.93, 128.50 – 128.11 (m), 127.86 (d, *J* = 3.0 Hz), 127.06, 56.13, 54.76 (d, *J* = 7.1 Hz), 54.21 – 53.80 (m), 21.37.

^{31}P NMR (121 MHz, Chloroform-*d*) δ 21.86.

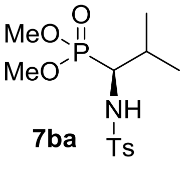

7ba

dimethyl (S)-(2-methyl-1-((4-methylphenyl)sulfonamido)propyl)phosphonate (7ba)

127mg, 95% yiedld, white solid.

^{1}H NMR (300 MHz, Chloroform-*d*) δ 7.82 – 7.73 (m, 2H), 7.28 (d, *J* = 8.2 Hz, 2H), 5.65 (dd, *J* = 9.9, 4.5 Hz, 1H), 3.69 (dd, *J* = 10.0, 3.6 Hz, 1H), 3.60 (dd, *J* = 16.1, 10.7 Hz, 6H), 2.42 (s, 3H), 2.10 (dtt, *J* = 13.8, 6.9, 3.7 Hz, 1H), 0.99 – 0.88 (m, 6H).

^{13}C NMR (75 MHz, Chloroform-*d*) δ 143.19, 138.72, 129.44, 127.12, 56.17, 54.14, 52.97 (dd, *J*

= 17.7, 7.2 Hz), 29.51 (d, J = 5.0 Hz), 21.56, 20.42, 20.25, 17.90 (d, J = 3.1 Hz).

^{31}P NMR (121 MHz, Chloroform-*d*) δ 25.77.

3.4 Characterization data of nitroalkene-based Pudovik reaction

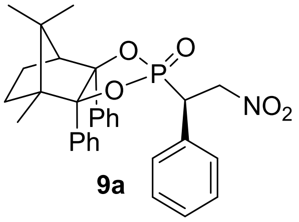

9a

(3a*S*,4*S*,7*S*,7a*S*)-4,8,8-Trimethyl-2-((*S*)-2-nitro-1-phenylethyl)-3a,7a-diphenylhexahydro-4,7-meth-anobenzo[*d*][1,3,2]dioxaphosphole 2-oxide (**9a**).

$[\alpha]_D^{25}$ = +3.00 °(c = 1.00 in MeOH);

The residue was purified by flash chromatography (PE/EtOAc = 10:1) on silica gel to afford the product (98 mg, 95% yield), white solid. R_f = 0.40 (PE/EA = 10:1).

m.p. = 173.1-175.1 ℃

^{1}H NMR (300 MHz, Chloroform-*d*) δ 7.51-7.16 (m, 14H), 7.10-6.96 (m, 1H), 4.74 (ddd, J = 13.7, 12.1, 4.5 Hz, 1H), 4.21 (ddd, J = 13.7, 6.0, 3.4 Hz, 1H), 3.81 (ddd, J = 20.5, 12.1, 3.4 Hz, 1H), 3.09 (d, J = 5.4 Hz, 1H), 2.10 (ddd, J = 19.6, 10.0, 4.9 Hz, 1H), 1.65-1.49 (m, 1H), 1.39-1.30 (m, 2H), 1.16 (s, 3H), 0.76 (s, 3H).

^{13}C NMR (75 MHz, Chloroform-*d*) δ 138.01 (d, J = 3.3 Hz), 135.42 (d, J = 4.1 Hz), 131.24 (d, J = 7.5 Hz), 130.34, 130.05 (d, J = 6.4 Hz), 129.11-128.66 (m), 128.56, 101.87, 99.46, 75.57 (d, J = 6.1 Hz), 55.79 (d, J = 3.4 Hz), 52.19 (d, J = 3.3 Hz), 48.27, 46.48, 44.79, 29.62, 26.45, 24.44, 21.49, 9.85.

^{31}P NMR (121 MHz, Chloroform-*d*) δ 34.78.

HRMS (ESI-MS) calculated for $C_{30}H_{33}NO_5P$ $[M+H]^+$: 518.2091, found: 518.2091.

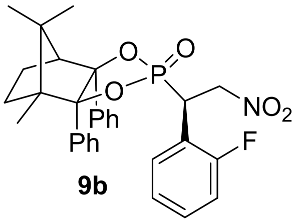

9b

(3a*S*,4*S*,7*S*,7a*S*)-2-((*R*)-1-(2-Fluorophenyl)-2-nitroethyl)-4,8,8-trimethyl-3a,7a-diphenyl-hexahydro-4,7-methanobenzo[*d*][1,3,2]dioxaphosphole 2-oxide (9b).

$[\alpha]_D^{25}$ = +75.99 °(c = 1.00 in MeOH);

The residue was purified by flash chromatography (PE/EtOAc = 10:1) on silica gel to afford the product (77 mg, 72% yield), white solid. R_f = 0.42 (PE/EA = 10:1).

m.p. = 75.0-77.0 ℃

^{1}H NMR (300 MHz, Chloroform-*d*) δ 7.70 (tt, *J* = 7.4, 2.0 Hz, 1H), 7.40-6.83 (m, 13H), 4.87-4.64 (m, 1H), 4.45-4.25 (m, 2H), 3.09 (d, *J* = 5.4 Hz, 1H), 2.09 (ddt, *J* = 15.1, 9.7, 5.9 Hz, 1H), 1.74 (s, 3H), 1.61-1.49 (m, 1H), 1.30 (dd, *J* = 11.2, 5.0 Hz, 2H), 1.15 (s, 3H), 0.77 (s, 3H).

^{13}C NMR (75 MHz, Chloroform-*d*) δ 137.58 (d, *J* = 3.2 Hz), 135.39 (d, *J* = 4.4 Hz), 130.32 (d, *J* = 5.4 Hz), 128.93, 128.14, 125.04, 119.76-118.74 (m), 116.20 (d, *J* = 1.9 Hz), 115.90, 102.01, 100.08 (d, *J* = 1.5 Hz), 75.39 (d, *J* = 4.3 Hz), 55.69 (d, *J* = 3.2 Hz), 52.05 (d, *J* = 3.5 Hz), 48.24, 38.24, 36.51, 29.82, 26.49, 24.27, 21.53, 9.71.

^{19}F NMR (282 MHz, Chloroform-*d*) δ -115.28 (dq, *J* = 10.0, 4.6 Hz).

^{31}P NMR (121 MHz, Chloroform-*d*) δ 34.26 (d, *J* = 3.9 Hz).

HRMS (ESI-MS) calculated for $C_{30}H_{32}FNO_5P$ $[M+H]^+$: 536.1997, found: 536.1997.

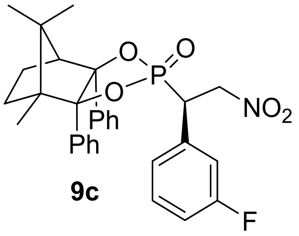

9c

(3a*S*,4*S*,7*S*,7a*S*)-2-((*R*)-1-(3-Fluorophenyl)-2-nitroethyl)-4,8,8-trimethyl-3a,7a-diphenylh-exahydro-4,7-methanobenzo[*d*][1,3,2]dioxaphosphole 2-oxide (**9c**).

$[\alpha]_D^{25}$ = +39.99 °(c = 1.00 in MeOH);

The residue was purified by flash chromatography (PE/EtOAc = 8:1) on silica gel to afford the product (102 mg, 95% yield), white solid. R_f = 0.42 (PE/EA = 8:1).

m.p. = 105.0-106.0 ℃

^{1}H NMR (300 MHz, Chloroform-*d*) δ 7.47-7.18 (m, 9H), 7.17-6.98 (m, 5H), 4.71 (ddd, *J* = 13.8, 12.1, 4.2 Hz, 1H), 4.20 (ddd, *J* = 13.9, 6.0, 3.3 Hz, 1H), 3.81 (ddd, *J* = 20.6, 12.1, 3.4 Hz, 1H), 3.10 (d, *J* = 5.3 Hz, 1H), 2.11 (ddd, *J* = 22.5, 11.4, 6.4 Hz, 1H), 1.72 (s, 3H), 1.65-1.49 (m, 1H), 1.40-1.30 (m, 2H), 1.16 (s, 3H), 0.78 (s, 3H).

^{13}C NMR (75 MHz, Chloroform-*d*) δ 164.40 (d, *J* = 2.4 Hz), 161.12 (d, *J* = 2.4 Hz), 137.88 (d, *J* = 3.4 Hz), 135.30 (d, *J* = 4.1 Hz), 133.66 (t, *J* = 7.6 Hz), 130.48 (dd, *J* = 8.3, 2.3 Hz), 128.94, 128.73, 128.33, 126.54, 125.63 (dd, *J* = 6.4, 3.0 Hz), 117.51 (d, *J* = 6.4 Hz), 117.21 (d, *J* = 6.4 Hz), 116.02 (d, *J* = 2.8 Hz), 115.74 (d, *J* = 2.8 Hz), 102.05, 99.69 (d, *J* = 1.3 Hz), 75.25 (d, *J* = 5.6 Hz), 55.81 (d, *J* = 3.4 Hz), 52.17 (d, *J* = 3.3 Hz), 48.25, 46.16 (d, *J* = 1.8 Hz), 44.48 (d, *J* = 1.9 Hz), 29.59, 26.41, 24.40, 21.46, 9.82.

^{19}F NMR (282 MHz, Chloroform-*d*) δ -111.88 (td, *J* = 9.0, 6.1 Hz).

^{31}P NMR (121 MHz, Chloroform-*d*) δ 33.89.

HRMS (ESI-MS) calculated for $C_{30}H_{32}FNO_5P$ $[M+H]^+$: 536.1997, found: 536.1998.

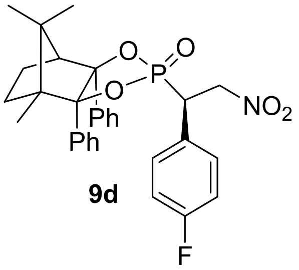

9d

(3a*S*,4*S*,7*S*,7a*S*)-2-((*R*)-1-(4-Fluorophenyl)-2-nitroethyl)-4,8,8-trimethyl-3a,7a-diphenyl-hexahydro-4,7-methanobenzo[*d*][1,3,2]dioxaphosphole 2-oxide (**9d**).

$[\alpha]_D^{25}$ = +29.99 °(c = 1.00 in MeOH);

The residue was purified by flash chromatography (PE/EtOAc = 10:1) on silica gel to afford the product (98 mg, 92% yield), white solid. R_f = 0.42 (PE/EA = 10:1).

m.p. = 179.0-180.0 ℃

^{1}H NMR (300 MHz, Chloroform-*d*) δ 7.44-7.12 (m, 11H), 7.04-6.92 (m, 3H), 4.60 (td, *J* = 13.0, 4.3 Hz, 1H), 4.11 (dt, *J* = 13.9, 4.4 Hz, 1H), 3.72 (ddd, *J* = 20.8, 12.3, 3.4 Hz, 1H), 3.02 (d, *J* = 5.4 Hz, 1H), 2.03 (ddt, *J* = 15.5, 10.8, 5.8 Hz, 1H), 1.65 (s, 3H), 1.50 (dt, *J* = 14.7, 7.4 Hz, 1H), 1.27 (p, *J* = 7.0, 5.3 Hz, 2H), 1.09 (s, 3H), 0.70 (s, 3H).

^{13}C NMR (75 MHz, Chloroform-*d*) δ 137.98 (d, *J* = 3.1 Hz), 135.45 (d, *J* = 4.2 Hz), 131.87 (t, *J* = 7.4 Hz), 129.90, 128.83 (d, *J* = 17.5 Hz), 116.03 (d, *J* = 20.9 Hz), 101.96, 99.61, 75.58 (d, *J* = 6.3 Hz), 55.85 (d, *J* = 3.5 Hz), 52.22 (d, *J* = 3.3 Hz), 48.30, 45.74, 44.05, 29.61, 26.44, 24.45, 21.49, 9.86.

^{19}F NMR (282 MHz, Chloroform-*d*) δ -112.92 (tq, *J* = 9.2, 4.9 Hz).

^{31}P NMR (121 MHz, Chloroform-*d*) δ 34.37 (d, *J* = 4.4 Hz).

HRMS (ESI-MS) calculated for $C_{30}H_{32}FNO_5P$ $[M+H]^+$: 536.1997, found: 536.1996.

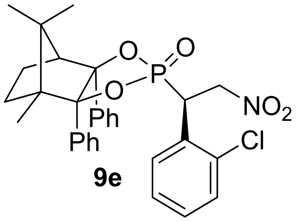

9e

(3a*S*,4*S*,7*S*,7a*S*)-2-((*R*)-1-(2-Chlorophenyl)-2-nitroethyl)-4,8,8-trimethyl-3a,7a-diphenyl-hexahydro-4,7-methanobenzo[*d*][1,3,2]dioxaphosphole 2-oxide (**9e**).

$[\alpha]_D^{25}$ = +119.98 °(c = 1.00 in MeOH);

The residue was purified by flash chromatography (PE/EtOAc = 10:1) on silica gel to afford the product (75 mg, 68% yield), white solid. R_f = 0.40 (PE/EA = 10:1).

m.p. = 102.2-04.2 ℃

^1H NMR (300 MHz, Chloroform-*d*) δ 7.94 (d, *J* = 7.6 Hz, 1H), 7.48 (s, 1H), 7.29 (h, *J* = 12.4, 9.8 Hz, 9H), 7.06 (t, *J* = 7.6 Hz, 2H), 6.79 (s, 1H), 4.74-4.40 (m, 3H), 3.10 (d, *J* = 5.4 Hz, 1H), 2.08 (dq, *J* = 14.3, 7.1 Hz, 1H), 1.74 (s, 3H), 1.55 (dt, *J* = 14.9, 7.2 Hz, 1H), 1.28 (t, *J* = 7.4 Hz, 2H), 1.15 (s, 3H), 0.76 (s, 3H).

^{13}C NMR (75 MHz, Chloroform-*d*) δ 137.56 (d, J = 3.3 Hz), 135.39 (d, *J* = 4.4 Hz), 135.14 (d, *J* = 10.5 Hz), 130.74 (d, *J* = 4.2 Hz), 130.13 (d, *J* = 4.3 Hz), 129.85 (d, *J* = 2.3 Hz), 128.90, 128.09, 127.83 (d, *J* = 2.5 Hz), 102.32, 100.35 (d, *J* = 1.8 Hz), 76.29 (d, *J* = 2.8 Hz), 55.72 (d, *J* = 3.1 Hz), 51.99 (d, *J* = 3.5 Hz), 48.15, 42.08, 40.35, 29.84, 26.55, 24.34, 21.47, 9.73.

^{31}P NMR (121 MHz, Chloroform-*d*) δ 34.80.

HRMS (ESI-MS) calculated for $C_{30}H_{32}ClNO_5P$ $[M+H]^+$: 552.1702, found: 552.1703.

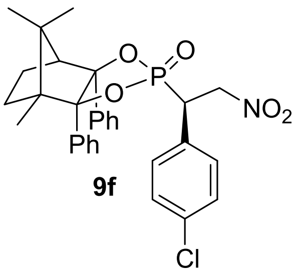

9f

(3a*S*,4*S*,7*S*,7a*S*)-2-((*R*)-1-(4-chlorophenyl)-2-nitroethyl)-4,8,8-trimethyl-3a,7a-diphenylh-exahydro-4,7-methanobenzo[*d*][1,3,2]dioxaphosphole 2-oxide (**9f**).

$[\alpha]_D^{25}$ = +52.99 °(c = 1.00 in MeOH);

The residue was purified by flash chromatography (PE/EtOAc = 10:1) on silica gel to afford the product (99 mg, 90% yield), white solid. R_f = 0.40 (PE/EA = 10:1).

m.p. = 172.0-173.0 ℃

^{1}H NMR (300 MHz, Chloroform-*d*) δ 7.46-7.19 (m, 12H), 7.01 (d, *J* = 24.3 Hz, 2H), 4.68 (ddd, *J* = 13.9, 12.2, 4.2 Hz, 1H), 4.19 (ddd, *J* = 13.8, 5.9, 3.4 Hz, 1H), 3.78 (ddd, *J* = 20.5, 12.2, 3.4 Hz, 1H), 3.09 (d, *J* = 5.3 Hz, 1H), 2.11 (ddt, *J* = 15.3, 10.7, 5.7 Hz, 1H), 1.72 (s, 3H), 1.57 (dt, *J* = 14.5, 7.3 Hz, 1H), 1.38-1.29 (m, 2H), 1.16 (s, 3H), 0.77 (s, 3H).

^{13}C NMR (75 MHz, Chloroform-*d*) δ 137.93 (d, *J* = 3.4 Hz), 135.40 (d, *J* = 4.1 Hz), 134.95 (d, *J* = 3.2 Hz), 131.43 (d, *J* = 6.4 Hz), 130.25, 129.88 (d, *J* = 7.4 Hz), 129.20 (d, *J* = 2.2 Hz), 128.84 (d, *J* = 17.9 Hz), 128.35, 127.16-125.90 (m), 102.01, 99.66 (d, *J* = 1.3 Hz), 75.38 (d, *J* = 5.8 Hz), 55.84 (d, *J* = 3.4 Hz), 52.20 (d, *J* = 3.3 Hz), 48.28, 45.90, 44.21, 29.60, 26.43, 24.43, 21.47, 9.85.

^{31}P NMR (121 MHz, Chloroform-*d*) δ 34.01.

HRMS (ESI-MS) calculated for $C_{30}H_{32}ClNO_5P$ $[M+H]^+$: 552.1702, found 552.1703.

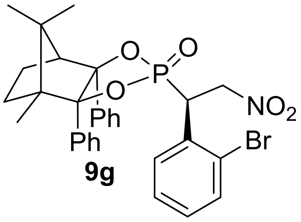

9g

(3a*S*,4*S*,7*S*,7a*S*)-2-((*R*)-1-(2-Bromophenyl)-2-nitroethyl)-4,8,8-trimethyl-3a,7a-diphenylh-exahydro-4,7-methanobenzo[*d*][1,3,2]dioxaphosphole 2-oxide (**9g**).

$[\alpha]_D^{25}$ = +135.88 °(c = 1.00 in MeOH);

The residue was purified by flash chromatography (PE/EtOAc = 10:1) on silica gel to afford the product (89 mg, 75% yield), white solid. R_f = 0.44 (PE/EA = 10:1).

m.p. = 169.0-170.0 ℃

^{1}H NMR (300 MHz, Chloroform-*d*) δ 8.03 (d, *J* = 7.9 Hz, 1H), 7.57-7.20 (m, 9H), 7.06 (t, *J* = 7.7 Hz, 2H), 6.78 (s, 2H), 4.53 (dtd, *J* = 15.9, 11.8, 10.7, 4.3 Hz, 3H), 3.10 (d, *J* = 5.4 Hz, 1H), 2.17-1.99 (m, 1H), 1.76 (s, 3H), 1.56 (dt, *J* = 14.6, 7.3 Hz, 1H), 1.27 (t, *J* = 7.4 Hz, 2H), 1.15 (s, 3H), 0.77 (s, 3H).

^{13}C NMR (75 MHz, Chloroform-*d*) δ 137.57 (d, *J* = 3.3 Hz), 135.43 (d, *J* = 4.4 Hz), 133.44, 132.00 (d, *J* = 4.8 Hz), 130.96 (d, *J* = 4.1 Hz), 130.06 (d, *J* = 2.2 Hz), 128.78, 128.36 (d, *J* = 2.2 Hz), 128.02, 126.02, 125.87, 102.20, 100.20 (d, *J* = 1.7 Hz), 76.45, 55.67 (d, *J* = 3.1 Hz), 51.93 (d, *J* = 3.5 Hz), 48.10, 44.69, 42.97, 29.79, 26.54, 24.33, 21.45, 9.71.

^{31}P NMR (121 MHz, Chloroform-*d*) δ 34.83.

HRMS (ESI-MS) calculated for $C_{30}H_{32}BrNO_5P$ $[M+H]^+$: 596.1196, found: 596.1196.

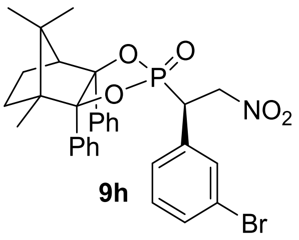

9h

(3a*S*,4*S*,7*S*,7a*S*)-2-((*R*)-1-(3-Bromophenyl)-2-nitroethyl)-4,8,8-trimethyl-3a,7a-diphenyl-hexahydro-4,7-methanobenzo[*d*][1,3,2]dioxaphosphole 2-oxide (**9h**).

$[\alpha]_D^{25}$ = +56.99 °(c = 1.00 in MeOH);

The residue was purified by flash chromatography (PE/EtOAc = 10:1) on silica gel to afford the product (103 mg, 87% yield), white solid. R_f = 0.42 (PE/EA = 10:1).

m.p. = 106.0-108.0 ℃

^{1}H NMR (300 MHz, Chloroform-*d*) δ 7.67-6.90 (m, 14H), 4.71 (ddd, *J* = 14.0, 12.1, 4.2 Hz, 1H), 4.18 (ddd, *J* = 14.0, 6.0, 3.3 Hz, 1H), 3.77 (ddd, *J* = 20.6, 12.1, 3.3 Hz, 1H), 3.10 (d, *J* = 5.3 Hz, 1H), 2.11 (tt, *J* = 10.3, 5.4 Hz, 1H), 1.71 (s, 3H), 1.56 (tt, *J* = 12.9, 5.9 Hz, 1H), 1.45-1.23 (m, 2H), 1.16 (s, 3H), 0.78 (s, 3H).

^{13}C NMR (75 MHz, Chloroform-*d*) δ 137.88 (d, *J* = 3.3 Hz), 135.23 (d, *J* = 4.1 Hz), 133.48 (dd, *J* = 9.3, 7.2 Hz), 131.95 (d, *J* = 2.9 Hz), 131.64-128.84 (m), 129.80, 128.76, 128.19 (d, *J* = 5.5 Hz),

122.71 (d, *J* = 2.3 Hz), 102.08, 99.67 (d, *J* = 1.3 Hz), 74.99 (d, J = 5.7 Hz), 55.81 (d, J = 3.4 Hz), 52.17 (d, *J* = 3.3 Hz), 48.23, 46.00, 44.32, 29.56, 26.39, 24.39, 21.45, 9.83.

^{31}P NMR (121 MHz, Chloroform-*d*) δ 33.77.

HRMS (ESI-MS) calculated for $C_{30}H_{32}BrNO_5P$ $[M+H]^+$: 596.1196, found: 596.1194.

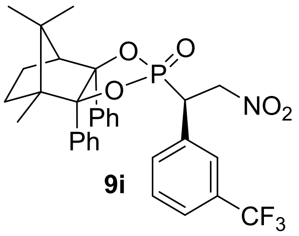

9i

(3a*S*,4*S*,7*S*,7a*S*)-4,8,8-Trimethyl-2-((*R*)-2-nitro-1-(3-(trifluoromethyl)phenyl)ethyl)-3a,7a-diphenyl-hexahydro-4,7-methanobenzo[*d*][1,3,2]dioxaphosphole 2-oxide (**9i**).

$[\alpha]_D^{25}$ = +23.00 °(c = 1.00 in MeOH);

The residue was purified by flash chromatography (PE/EtOAc = 10:1) on silica gel to afford the product (101 mg, 86% yield), white solid. R_f = 0.35 (PE/EA = 10:1).

m.p. = 152.0-153.0 ℃

^{1}H NMR (300 MHz, Chloroform-d) δ 7.94 – 6.72 (m, 15H), 4.82 – 4.35 (m, 1H), 4.24 – 3.97 (m, 1H), 3.94 – 3.54 (m, 1H), 3.21 – 2.85 (m, 1H), 2.22 – 1.88 (m, 1H), 1.78 – 1.37 (m, 4H), 1.35 – 1.17 (m, 2H), 1.07 (s, 3H), 0.69 (s, 3H).

^{13}C NMR (75 MHz, Chloroform-*d*) δ 137.86 (d, *J* = 3.5 Hz), 135.30 (d, *J* = 4.1 Hz),132.92 (d, *J* = 5.3 Hz), 132.48 (d, *J* = 7.4 Hz), 131.35, 130.93, 129.63 (d, *J* = 2.5 Hz), 128.93 (d, *J* = 9.4 Hz), 127.37, 125.74, 122.09, 102.20, 99.77, 74.81 (d, *J* = 5.5 Hz), 55.84 (d, *J* = 3.3 Hz), 52.16 (d, *J* = 3.4 Hz), 48.23, 46.30, 44.63, 29.55, 26.36, 24.38, 21.44, 9.81. ^{19}F NMR (282 MHz, Chloroform-d) δ -62.31.

^{31}P NMR (121 MHz, Chloroform-*d*) δ 34.43.

HRMS (ESI-MS) calculated for $C_{31}H_{32}F_3NO_5P$ $[M+H]^+$: 586.1965, found: 586.1966.

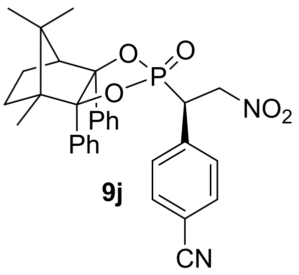

9j

4-((1*R*)-2-Nitro-1-((3a*S*,4*S*,7*S*,7a*S*)-4,8,8-trimethyl-2-oxido-3a,7a-diphenylhexahydro-4,7-methano-benzo[*d*][1,3,2]dioxaphosphol-2-yl)ethyl)benzonitrile (**9j**).

$[\alpha]_D^{25}$ = +10.00 °(c = 1.00 in MeOH);

The residue was purified by flash chromatography (PE/EtOAc = 10:1) on silica gel to afford the product (95 mg, 88% yield), white solid. R_f = 0.30 (PE/EA = 10:1).

m.p. = 169.0-170.0 ℃

^{1}H NMR (300 MHz, Chloroform-*d*) δ 7.67 (d, *J* = 7.9 Hz, 2H), 7.50-7.22 (m, 10H), 7.01 (s, 2H), 4.72 (td, *J* = 13.2, 4.0 Hz, 1H), 4.21 (dt, *J* = 14.9, 4.4 Hz, 1H), 3.87 (ddd, *J* = 20.6, 12.3, 3.3 Hz, 1H), 3.11 (d, *J* = 5.4 Hz, 1H), 2.10 (dt, *J* = 17.9, 8.2 Hz, 1H), 1.70 (s, 3H), 1.57 (dt, *J* = 14.7, 7.4 Hz, 1H), 1.34 (t, *J* = 7.5 Hz, 2H), 1.17 (s, 3H), 0.78 (s, 3H).

^{13}C NMR (75 MHz, Chloroform-*d*) δ 137.76 (d, *J* = 3.3 Hz), 137.01 (d, *J* = 7.4 Hz), 135.35 (d, J = 4.1 Hz), 132.64 (d, *J* = 2.3 Hz), 130.95 (d, *J* = 6.2 Hz), 129.80, 129.03 (d, *J* = 15.0 Hz), 126.60, 118.40, 112.89 (d, *J* = 3.0 Hz), 102.30, 99.99, 74.92 (d, *J* = 5.0 Hz), 55.90 (d, *J* = 3.3 Hz), 52.20 (d, *J* = 3.5 Hz), 48.28, 46.55, 44.88, 29.60, 26.43, 24.40, 21.46, 9.83.

^{31}P NMR (121 MHz, Chloroform-*d*) δ 32.82.

HRMS (ESI-MS) calculated for $C_{31}H_{32}N_2O_5P$ $[M+H]^+$: 543.2044, found: 543.2043.

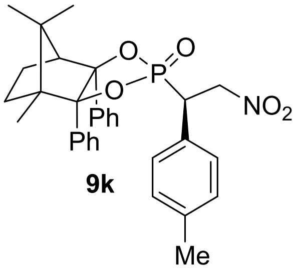

9k

(3a*S*,4*S*,7*S*,7a*S*)-4,8,8-Trimethyl-2-((*R*)-2-nitro-1-(p-tolyl)ethyl)-3a,7a-diphenylhexahydro-4,7-meth-anobenzo[*d*][1,3,2]dioxaphosphole 2-oxide (**9k**).

$[\alpha]_D^{25}$ = +9.00 °(c = 1.00 in MeOH);

The residue was purified by flash chromatography (PE/EtOAc = 10:1) on silica gel to afford the product (91 mg, 86% yield), white solid. R_f = 0.45 (PE/EA = 10:1).

m.p. = 180.2-182.2 ℃

^{1}H NMR (300 MHz, Chloroform-*d*) δ 7.53-6.98 (m, 14H), 4.71 (td, *J* = 12.9, 4.5 Hz, 1H), 4.18 (d, *J* = 13.0 Hz, 1H), 3.77 (dd, *J* = 20.7, 12.1 Hz, 1H), 3.08 (d, *J* = 5.4 Hz, 1H), 2.36 (s, 3H), 2.10 (ddd, *J* = 16.7, 11.1, 5.9 Hz, 1H), 1.73 (s, 3H), 1.57 (dt, *J* = 14.8, 7.6 Hz, 1H), 1.43-1.23 (m, 2H), 1.15 (s, 3H), 0.76 (s, 3H).

^{13}C NMR (75 MHz, Chloroform-*d*) δ 138.57 (d, *J* = 2.8 Hz), 138.03 (d, *J* = 3.3 Hz), 135.42 (d, *J* = 4.1 Hz), 130.33, 129.86 (d, *J* = 6.4 Hz), 129.63 (d, *J* = 2.2 Hz), 128.78, 128.50, 128.01 (d, *J* = 7.6 Hz), 101.74, 99.32, 75.57 (d, *J* = 6.3 Hz), 55.74 (d, *J* = 3.4 Hz), 52.15 (d, *J* = 3.3 Hz), 48.23, 46.12, 44.43, 29.58, 26.41, 24.42, 21.46, 21.27, 9.83.

^{31}P NMR (121 MHz, Chloroform-*d*) δ 34.96.

HRMS (ESI-MS) calculated for $C_{31}H_{35}NO_5P$ $[M+H]^+$: 532.2248, found: 532.2249.

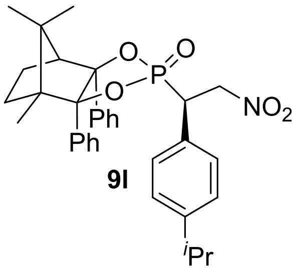

9l

(3a*S*,4*S*,7*S*,7a*S*)-2-((*R*)-1-(4-Isopropylphenyl)-2-nitroethyl)-4,8,8-trimethyl-3a,7a-diphenylhexahy-dro-4,7-methanobenzo[*d*][1,3,2]dioxaphosphole 2-oxide (**9l**).

$[\alpha]_D^{25}$ = +34 °(c = 1.00 in MeOH);

The residue was purified by flash chromatography (PE/EtOAc = 10:1) on silica gel to afford the product (101 mg, 90% yield), white solid. R_f = 0.42 (PE/EA = 10:1).

m.p. = 190.0-191.0 ℃

^{1}H NMR (300 MHz, Chloroform-*d*) δ 7.42-7.18 (m, 12H), 7.04 (s, 2H), 4.80-4.64 (m, 1H), 4.18 (ddd, *J* = 13.8, 6.0, 3.5 Hz, 1H), 3.78 (ddd, *J* = 20.6, 12.1, 3.5 Hz, 1H), 3.08 (d, *J* = 5.3 Hz, 1H), 2.90 (h, *J* = 7.0 Hz, 1H), 2.17-1.98 (m, 1H), 1.73 (s, 3H), 1.57 (dt, *J* = 14.6, 7.4 Hz, 1H), 1.43-1.30 (m, 2H), 1.27 (d, *J* = 6.9 Hz, 6H), 1.16 (s, 3H), 0.77 (s, 3H).

^{13}C NMR (75 MHz, Chloroform-*d*) δ 149.40, 138.06, 135.52 (d, *J* = 4.0 Hz), 130.41, 129.89 (d,

J = 6.3 Hz), 128.84, 128.54, 128.31 (d, *J* = 7.7 Hz), 127.08 (d, *J* = 2.2 Hz), 101.79, 99.35, 75.55 (d, *J* = 6.3 Hz), 55.82 (d, *J* = 3.4 Hz), 52.21 (d, *J* = 3.2 Hz), 48.31, 46.15, 44.47, 33.95, 29.66, 26.46, 24.48, 24.03 (d, *J* = 3.7 Hz), 21.52, 9.91.

^{31}P NMR (121 MHz, Chloroform-*d*) δ 35.08.

HRMS (ESI-MS) calculated for $C_{33}H_{39}NO_5P$ [M+H]$^+$: 560.2561, found 560.2550.

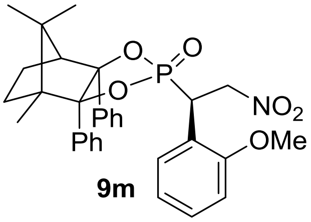

9m

(3a*S*,4*S*,7*S*,7a*S*)-2-((*R*)-1-(2-Methoxyphenyl)-2-nitroethyl)-4,8,8-trimethyl-3a,7a-diphenyl-hexahy-dro-4,7-methanobenzo[*d*][1,3,2]dioxaphosphole 2-oxide (**9m**).

$[\alpha]_D^{25}$ = +87.99 °(c = 1.00 in MeOH);

The residue was purified by flash chromatography (PE/EtOAc = 10:1) on silica gel to afford the product (84 mg, 77% yield), white solid. R_f = 0.40 (PE/EA = 10:1).

m.p. = 202.0-203.0 ℃

^{1}H NMR (300 MHz, Chloroform-*d*) δ 7.79-7.13 (m, 9H), 7.01 (td, *J* = 7.6, 3.2 Hz, 3H), 6.75 (d, *J* = 8.3 Hz, 2H), 4.75-4.48 (m, 3H), 3.38 (s, 3H), 3.07 (d, *J* = 5.3 Hz, 1H), 2.06 (ddt, *J* = 15.4, 10.6, 5.2 Hz, 1H), 1.76 (s, 3H), 1.61-1.46 (m, 1H), 1.25 (tt, *J* = 9.1, 5.0 Hz, 2H), 1.14 (s, 3H), 0.73 (s, 3H).

^{13}C NMR (75 MHz, Chloroform-*d*) δ 156.84 (d, *J* = 7.9 Hz), 137.63 (d, *J* = 3.2 Hz), 135.97 (d, *J* = 4.4 Hz), 129.53 (dd, *J* = 6.2, 3.5 Hz), 128.66, 127.43, 121.20 (d, *J* = 2.5 Hz), 120.39 (d, *J* = 6.3 Hz), 110.68 (d, *J* = 1.7 Hz), 101.38, 99.73 (d, *J* = 1.9 Hz), 76.55 (d, *J* = 3.2 Hz), 55.53 (d, *J* = 3.0 Hz), 55.12, 51.91 (d, *J* = 3.6 Hz), 48.14, 38.04, 36.30, 29.87, 26.52, 24.23, 21.55, 9.63.

^{31}P NMR (121 MHz, Chloroform-*d*) δ 36.81.

HRMS (ESI-MS) calculated for $C_{31}H_{35}NO_6P$ [M+H]$^+$: 548.2197, found: 548.2197.

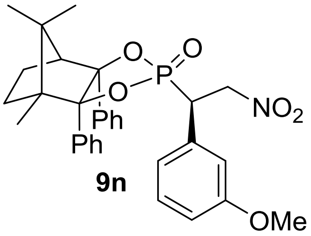

9n

(3a*S*,4*S*,7*S*,7a*S*)-2-((*R*)-1-(3-Methoxyphenyl)-2-nitroethyl)-4,8,8-trimethyl-3a,7a-diphenyl-hexahy-dro-4,7-methanobenzo[*d*][1,3,2]dioxaphosphole 2-oxide (**9n**).

$[\alpha]_D^{25}$ = +16.00 °(c = 1.00 in MeOH);

The residue was purified by flash chromatography (PE/EtOAc = 10:1) on silica gel to afford the product (94 mg, 86% yield), white solid. R_f = 0.42 (PE/EA = 10:1).

m.p. = 166.0-168.0 ℃

^{1}H NMR (300 MHz, Chloroform-*d*) δ 7.68 (dt, *J* = 7.7, 2.0 Hz, 1H), 7.47-7.16 (m, 8H), 7.03 (dt, *J* = 8.1, 3.9 Hz, 3H), 6.75 (d, *J* = 8.3 Hz, 2H), 4.75-4.47 (m, 3H), 3.38 (s, 3H), 3.07 (d, *J* = 5.4 Hz, 1H), 2.15-1.98 (m, 1H), 1.75 (s, 3H), 1.54 (ddd, *J* = 14.6, 8.6, 6.1 Hz, 1H), 1.23 (dt, *J* = 9.3, 5.1 Hz, 2H), 1.14 (s, 3H), 0.73 (s, 3H).

^{13}C NMR (75 MHz, Chloroform-*d*) δ 159.91 (d, *J* = 2.0 Hz), 138.01 (d, *J* = 3.3 Hz), 135.46 (d, *J* = 4.1 Hz), 132.70 (d, *J* = 7.3 Hz), 130.36, 129.93 (d, *J* = 2.2 Hz), 128.87, 128.52, 126.47, 122.30 (d, *J* = 6.5 Hz), 115.43 (d, *J* = 6.4 Hz), 114.55 (d, *J* = 2.7 Hz), 101.89, 99.46 (d, *J* = 1.4 Hz), 75.58 (d, *J* = 5.7 Hz), 55.82 (d, *J* = 3.4 Hz), 55.37, 52.21 (d, J = 3.4 Hz), 48.29, 46.53, 44.84, 29.67, 26.46, 24.45, 21.53, 9.87.

^{31}P NMR (121 MHz, Chloroform-*d*) δ 36.77.

HRMS (ESI-MS) calculated for $C_{31}H_{35}NO_6P$ $[M+H]^+$: 548.2197, found: 548.2200.

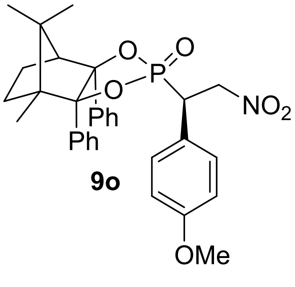

9o

(3a*S*,4*S*,7*S*,7a*S*)-2-((*R*)-1-(4-Methoxyphenyl)-2-nitroethyl)-4,8,8-trimethyl-3a,7a-diphenyl-hexahy-dro-4,7-methanobenzo[*d*][1,3,2]dioxaphosphole 2-oxide (**9o**).

$[\alpha]_D^{25}$ = +34.99 °(c = 1.00 in MeOH);

The residue was purified by flash chromatography (PE/EtOAc = 10:1) on silica gel to afford the product (92 mg, 84% yield), white solid. R_f = 0.40 (PE/EA = 10:1).

m.p. = 208.0-210.0 ℃

^{1}H NMR (300 MHz, Chloroform-*d*) δ 7.48-7.14 (m, 11H), 7.04 (s, 1H), 6.88 (d, *J* = 8.2 Hz, 2H), 4.68 (td, *J* = 12.8, 4.4 Hz, 1H), 4.18 (dt, *J* = 14.4, 4.1 Hz, 1H), 3.82 (s, 3H), 3.80-3.67 (m, 1H), 3.08 (d, *J* = 5.3 Hz, 1H), 2.10 (ddt, *J* = 15.3, 10.7, 5.0 Hz, 1H), 1.73 (s, 3H), 1.57 (dt, *J* = 14.7, 7.5 Hz, 1H), 1.33 (dt, *J* = 11.0, 5.0 Hz, 2H), 1.15 (s, 3H), 0.77 (s, 3H).

^{13}C NMR (75 MHz, Chloroform-*d*) δ 159.89 (d, *J* = 2.6 Hz), 138.04 (d, *J* = 3.3 Hz), 135.43 (d, *J* = 4.0 Hz), 131.18 (d, *J* = 6.4 Hz), 130.33, 128.65 (d, *J* = 18.9 Hz), 128.23, 126.41, 122.82 (d, *J* = 7.6 Hz), 114.33 (d, *J* = 2.0 Hz), 101.70, 99.31 (d, *J* = 1.3 Hz), 75.66 (d, *J* = 6.9 Hz), 55.74 (d, *J* = 3.4 Hz), 55.37, 52.15 (d, *J* = 3.3 Hz), 48.23, 45.69, 43.99, 29.55, 26.39, 24.41, 21.45, 9.83.

^{31}P NMR (121 MHz, Chloroform-*d*) δ 35.04.

HRMS (ESI-MS) calculated for $C_{31}H_{35}NO_6P$ $[M+H]^+$: 548.2197, found: 548.2198.

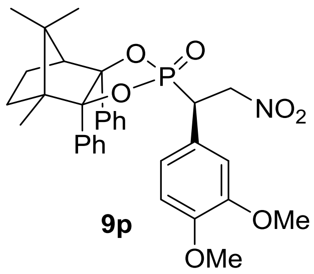

9p

(3a*S*,4*S*,7*S*,7a*S*)-2-((*R*)-1-(3,4-Dimethoxyphenyl)-2-nitroethyl)-4,8,8-trimethyl-3a,7a-diphenylhexahydro-4,7-methanobenzo[*d*][1,3,2]dioxaphosphole 2-oxide (**9p**).

$[\alpha]_D^{25}$ = +8.00 °(c = 1.00 in MeOH);

The residue was purified by flash chromatography (PE/EtOAc = 10:1) on silica gel to afford the product (106 mg, 92% yield), white solid. R_f = 0.40 (PE/EA = 10:1).

m.p. = 115.0-157.0 ℃

^{1}H NMR (300 MHz, Chloroform-*d*) δ 7.46-7.16 (m, 9H), 7.05 (s, 2H), 6.84 (d, *J* = 4.1 Hz, 3H), 4.69 (td, *J* = 12.9, 4.5 Hz, 1H), 4.18 (dt, *J* = 14.0, 4.3 Hz, 1H), 3.89 (d, *J* = 7.9 Hz, 6H), 3.74 (ddd, *J* = 20.6, 12.2, 3.4 Hz, 1H), 3.09 (d, *J* = 5.3 Hz, 1H), 2.11 (ddd, *J* = 21.4, 10.3, 5.8 Hz, 1H), 1.74 (s, 3H), 1.57 (dt, *J* = 14.6, 7.3 Hz, 1H), 1.33 (t, *J* = 8.0 Hz, 2H), 1.16 (s, 3H), 0.78 (s, 3H).

^{13}C NMR (75 MHz, Chloroform-*d*) δ 149.37 (d, *J* = 2.7 Hz), 149.14 (d, *J* = 1.9 Hz), 138.02 (d, *J* = 3.4 Hz), 135.55 (d, *J* = 4.1 Hz), 130.30, 128.83, 128.47, 126.40, 123.30 (d, *J* = 7.5 Hz), 122.70 (d, *J* = 7.4 Hz), 112.53 (d, *J* = 5.9 Hz), 111.22 (d, *J* = 2.2 Hz), 101.76, 99.33 (d, *J* = 1.3 Hz), 75.79 (d, *J* = 6.8 Hz), 56.00 (d, *J* = 1.5 Hz), 55.80 (d, *J* = 3.4 Hz), 52.16 (d, *J* = 3.3 Hz), 48.24, 46.11, 44.41, 29.58, 26.42, 24.43, 21.48, 9.85.

^{31}P NMR (121 MHz, Chloroform-*d*) δ 34.88.

HRMS (ESI-MS) calculated for $C_{32}H_{37}NO_7P$ $[M+H]^+$: 578.2303, found: 578.2303.

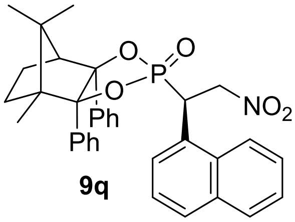

9q

(3a*S*,4*S*,7*S*,7a*S*)-4,8,8-trimethyl-2-((*R*)-1-(naphthalen-1-yl)-2-nitroethyl)-3a,7a-diphenylhexahydro-4,7-methanobenzo[*d*][1,3,2]dioxaphosphole 2-oxide (**9q**).

$[\alpha]_D^{25}$ = +96.98 °(c = 1.00 in MeOH);

The residue was purified by flash chromatography (PE/EtOAc = 10:1) on silica gel to afford the product (102 mg, 90% yield), white solid. R_f = 0.35 (PE/EA = 10:1).

m.p. = 165.0-166.0 ℃

^1H NMR (300 MHz, Chloroform-*d*) δ 7.98-6.96 (m, 15H), 6.73 (t, *J* = 7.4 Hz, 2H), 5.33-5.14 (m, 1H), 4.87-4.53 (m, 2H), 2.95 (d, *J* = 5.4 Hz, 1H), 2.14-1.96 (m, 1H), 1.82 (s, 3H), 1.62-1.45 (m, 1H), 1.32 (t, *J* = 7.6 Hz, 2H), 1.16 (s, 3H), 0.95 (s, 3H).

^{13}C NMR (75 MHz, Chloroform-*d*) δ 136.78 (d, *J* = 3.4 Hz), 135.96 (d, *J* = 4.2 Hz), 133.98, 131.18, 131.04, 130.06, 129.39, 128.88, 128.41, 127.88 (dd, *J* = 10.7, 5.4 Hz), 127.65, 127.22, 127.08-126.63 (m), 125.76, 125.37 (d, *J* = 1.9 Hz), 121.31, 101.49, 99.83 (d, *J* = 1.6 Hz), 55.80 (d, *J* = 3.3 Hz), 51.95 (d, *J* = 3.5 Hz), 48.32, 42.66, 40.90, 29.82, 29.56, 26.49, 24.66, 21.61, 10.12.

^{31}P NMR (121 MHz, Chloroform-*d*) δ 36.62.

HRMS (ESI-MS) calculated for $C_{34}H_{35}NO_5P$ $[M+H]^+$: 568.2248, found: 568.2251.

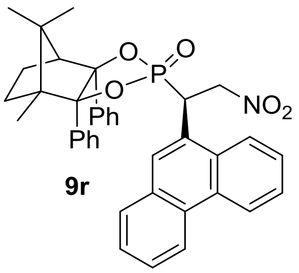

9r

(3a*S*,4*S*,7*S*,7a*S*)-4,8,8-Trimethyl-2-((*R*)-2-nitro-1-(phenanthren-9-yl)ethyl)-3a,7a-diphenyl-hexahyd-ro-4,7-methanobenzo[*d*][1,3,2]dioxaphosphole 2-oxide (**9r**).

$[\alpha]_D^{25}$ = +77.99 °(c = 1.00 in MeOH);

The residue was purified by flash chromatography (PE/EtOAc = 5:1) on silica gel to afford the product (106 mg, 86% yield), white solid. R_f = 0.42 (PE/EA = 5:1).

m.p. = 174.0-175.0 ℃

^{1}H NMR (300 MHz, Chloroform-*d*) δ 8.66 (d, *J* = 9.1 Hz, 2H), 8.20 (s, 2H), 7.92 (d, *J* = 8.4 Hz, 2H), 7.73 (d, *J* = 8.5 Hz, 2H), 7.34 (dddd, *J* = 54.1, 20.8, 15.1, 7.7 Hz, 10H), 6.56 (t, *J* = 7.3 Hz, 1H), 5.72-5.47 (m, 2H), 4.81-4.60 (m, 1H), 2.85 (d, *J* = 5.3 Hz, 1H), 1.99 (dq, *J* = 16.6, 8.9 Hz, 1H), 1.86 (s, 3H), 1.41-1.24 (m, 3H), 1.13 (s, 3H), 0.97 (s, 3H).

^{13}C NMR (75 MHz, Chloroform-*d*) δ 136.37 (d, *J* = 3.9 Hz), 135.84 (d, *J* = 4.1 Hz), 131.66, 131.37 (d, *J* = 2.5 Hz), 130.86, 130.71, 130.37 (d, *J* = 4.4 Hz), 129.78 (d, *J* = 1.5 Hz), 129.49 (d, *J* = 2.7 Hz), 129.29, 128.19, 127.51, 127.10 (d, *J* = 5.0 Hz), 126.73, 125.08, 124.84 (d, *J* = 1.8 Hz), 124.73, 124.43 (d, *J* = 5.3 Hz), 121.86, 100.78 (d, *J* = 2.2 Hz), 55.78 (d, *J* = 3.6 Hz), 51.90 (d, *J* = 3.0 Hz), 48.22, 41.38, 39.63, 29.21, 26.60, 25.00, 21.49, 10.31.

^{31}P NMR (121 MHz, Chloroform-*d*) δ 39.91.

HRMS (ESI-MS) calculated for $C_{38}H_{37}NO_5P$ $[M+H]^+$: 618.2404, found: 618.2402.

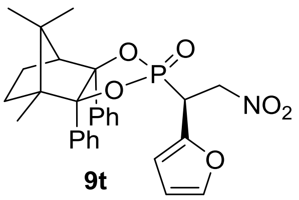

9t

(3a*S*,4*S*,7*S*,7a*S*)-2-((*R*)-1-(Furan-2-yl)-2-nitroethyl)-4,8,8-trimethyl-3a,7a-diphenylhexa-hydro-4,7-methanobenzo[*d*][1,3,2]dioxaphosphole 2-oxide (**9t**).

$[\alpha]_D^{25}$ = +44.99 °(c = 1.00 in MeOH);

The residue was purified by flash chromatography (PE/EtOAc = 10:1) on silica gel to afford the product (89 mg, 88% yield), white solid. R_f = 0.44 (PE/EA = 10:1).

m.p. = 157.1-159.1 ℃

^{1}H NMR (300 MHz, Chloroform-*d*) δ 7.31 (ddd, *J* = 28.1, 21.0, 12.7 Hz, 11H), 6.37 (dd, *J* = 10.0, 3.5 Hz, 2H), 4.84-4.69 (m, 1H), 4.17 (ddd, *J* = 19.2, 8.7, 4.5 Hz, 1H), 4.05 (ddd, *J* = 15.3, 11.6, 5.8 Hz, 1H), 3.08 (d, *J* = 5.3 Hz, 1H), 2.10 (dtd, *J* = 13.8, 9.4, 8.5, 4.4 Hz, 1H), 1.74 (s, 3H), 1.55 (dd, *J* = 14.5, 7.3 Hz, 1H), 1.34 (th, *J* = 11.8, 6.1, 5.1 Hz, 2H), 1.16 (s, 3H), 0.83 (s, 3H).

^{13}C NMR (75 MHz, Chloroform-*d*) δ 144.99, 144.86, 143.41 (d, *J* = 3.0 Hz), 137.69 (d, *J* = 3.3 Hz), 135.30 (d, *J* = 4.1 Hz), 129.99, 128.84, 128.41, 126.41, 111.04 (d, *J* = 2.7 Hz), 109.99 (d, *J* = 7.0 Hz), 101.82, 99.70 (d, *J* = 1.4 Hz), 73.17 (d, *J* = 5.8 Hz), 55.75 (d, *J* = 3.4 Hz), 52.01 (d, *J* = 3.4 Hz), 48.24, 40.70, 38.96, 29.54, 26.37, 24.31, 21.51, 9.82.

^{31}P NMR (121 MHz, Chloroform-*d*) δ 30.86.

HRMS (ESI-MS) calculated for $C_{28}H_{31}NO_6P$ $[M+H]^+$: 508.1884, found: 508.1884.

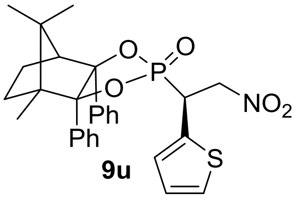

9u

(3a*S*,4*S*,7*S*,7a*S*)-4,8,8-Trimethyl-2-((*R*)-2-nitro-1-(thiophen-2-yl)ethyl)-3a,7a-diphenylhexahydro-4,7-methanobenzo[*d*][1,3,2]dioxaphosphole 2-oxide (**9u**).

$[\alpha]_D^{25}$ = +16.00 °(c = 1.00 in MeOH);

The residue was purified by flash chromatography (PE/EtOAc = 10:1) on silica gel to afford the product (92 mg, 88% yield), white solid. R_f = 0.44 (PE/EA = 10:1).

m.p. = 157.0-158.0 ℃

^{1}H NMR (300 MHz, Chloroform-*d*) δ 7.65-6.82 (m, 13H), 4.64 (td, *J* = 12.9, 4.0 Hz, 1H), 4.14 (ddd, *J* = 21.0, 10.2, 6.7 Hz, 2H), 3.09 (d, *J* = 5.3 Hz, 1H), 2.11 (ddt, *J* = 20.5, 15.3, 7.0 Hz, 1H), 1.74 (s, 3H), 1.58 (dt, *J* = 14.6, 7.6 Hz, 1H), 1.36 (dt, *J* = 11.0, 6.3 Hz, 2H), 1.17 (s, 3H), 0.80 (s, 3H).

^{13}C NMR (75 MHz, Chloroform-*d*) δ 137.89 (d, *J* = 3.3 Hz), 135.20 (d, *J* = 3.9 Hz), 132.32 (d, *J* = 8.7 Hz), 130.09, 129.95-129.47 (m), 129.26, 129.16, 128.84, 128.57, 128.29, 127.04 (dd, *J* = 11.6,

2.9 Hz), 126.59, 101.91, 99.53 (d, *J* = 1.2 Hz), 76.24 (d, *J* = 6.8 Hz), 55.82 (d, *J* = 3.5 Hz), 52.11 (d, *J* = 3.3 Hz), 48.27, 42.00, 40.26, 29.45, 26.36, 24.42, 21.51, 9.87.

^{31}P NMR (121 MHz, Chloroform-*d*) δ 32.78.

HRMS (ESI-MS) calculated for $C_{28}H_{31}NO_5PS$ $[M+H]^+$: 524.1656, found: 524.1656.

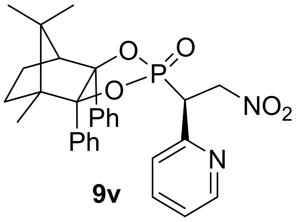

9v

(3a*S*,4*S*,7*S*,7a*S*)-4,8,8-Trimethyl-2-((*R*)-2-nitro-1-(pyridin-2-yl)ethyl)-3a,7a-diphenylhexahydro-4,7-methanobenzo[*d*][1,3,2]dioxaphosphole 2-oxide (**9v**).

$[\alpha]_D^{25}$ = +25.00 °(c = 1.00 in MeOH);

The residue was purified by flash chromatography (PE/EtOAc = 5:1) on silica gel to afford the product (90 mg, 87% yield), white solid. R_f = 0.36 (PE/EA = 5:1).

m.p. = 184.0-186.0 ℃

^{1}H NMR (300 MHz, Chloroform-*d*) δ 8.63 (d, *J* = 4.8 Hz, 1H), 8.34 (s, 1H), 7.94-7.85 (m, 1H), 7.37 (ddd, *J* = 24.0, 17.5, 11.4 Hz, 9H), 7.02 (s, 2H), 4.70 (td, *J* = 13.1, 4.0 Hz, 1H), 4.20 (ddd, *J* = 14.0, 5.9, 3.4 Hz, 1H), 3.82 (ddd, *J* = 20.5, 12.3, 3.3 Hz, 1H), 3.11 (d, *J* = 5.4 Hz, 1H), 2.14 (dq, *J* = 9.6, 5.0 Hz, 1H), 1.71 (s, 3H), 1.58 (dt, *J* = 14.5, 7.4 Hz, 1H), 1.46-1.23 (m, 2H), 1.17 (s, 3H), 0.78 (s, 3H).

^{13}C NMR (75 MHz, Chloroform-*d*) δ 151.24 (d, *J* = 7.9 Hz), 150.17 (d, *J* = 2.9 Hz), 137.84 (d, *J* = 3.3 Hz), 137.01 (d, *J* = 4.7 Hz), 135.23 (d, *J* = 4.1 Hz), 129.80, 128.97 (d, *J* = 8.0 Hz), 127.70 (d, J = 7.3 Hz), 123.92 (d, *J* = 2.3 Hz), 102.18, 99.83, 74.85 (d, *J* = 5.6 Hz), 55.85 (d, *J* = 3.4 Hz), 52.17 (d, *J* = 3.4 Hz), 48.27, 43.85, 42.16, 29.53, 26.38, 24.41, 21.45, 9.83.

^{31}P NMR (121 MHz, Chloroform-*d*) δ 33.49.

HRMS (ESI-MS) calculated for $C_{29}H_{32}N_2O_5P$ $[M+H]^+$: 519.2044, found 519.2045.

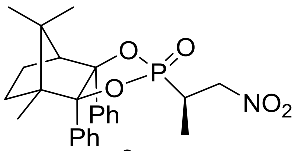

9wa

(3a*S*,4*S*,7*S*,7a*S*)-4,8,8-Trimethyl-2-((*R*)-1-nitropropan-2-yl)-3a,7a-diphenylhexahydro-4,7-methano-benzo[*d*][1,3,2]dioxaphosphole 2-oxide (**9wa**).

$[\alpha]_D^{25}$ = +123.98 °(c = 1.00 in MeOH);

The residue was purified by flash chromatography (PE/EtOAc = 15:1) on silica gel to afford the product (64 mg, 70% yield), white solid. R_f = 0.35 (PE/EA = 15:1).

m.p. = 105.0-106.0 ℃

^{1}H NMR (300 MHz, Chloroform-*d*) δ 7.26 (s, 10H), 4.21 (td, *J* = 12.6, 3.8 Hz, 1H), 3.92 (ddd, *J* = 13.2, 7.0, 3.1 Hz, 1H), 3.06 (d, *J* = 5.4 Hz, 1H), 2.56 (dddt, *J* = 15.0, 11.9, 6.6, 3.1 Hz, 1H), 2.19-2.01 (m, 1H), 1.74 (s, 3H), 1.57 (dt, *J* = 14.5, 7.3 Hz, 1H), 1.45-1.30 (m, 5H), 1.18 (s, 3H), 0.88 (s, 3H).

^{13}C NMR (75 MHz, Chloroform-*d*) δ 138.05 (d, *J* = 3.3 Hz), 135.89 (d, *J* = 3.9 Hz), 128.78, 128.58, 101.41, 99.36, 75.47 (d, *J* = 5.2 Hz), 55.84 (d, *J* = 3.3 Hz), 52.19 (d, *J* = 3.3 Hz), 48.35, 34.45, 32.72, 29.57, 26.42, 24.51, 21.59, 12.53 (d, *J* = 6.1 Hz), 10.05.

^{31}P NMR (121 MHz, Chloroform-*d*) δ 39.79.

HRMS (ESI-MS) calculated for $C_{25}H_{31}NO_5P$ $[M+H]^+$: 456.1935, found: 456.1935.

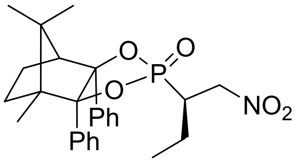

9wb

(3a*S*,4*S*,7*S*,7a*S*)-4,8,8-Trimethyl-2-((*R*)-1-nitrobutan-2-yl)-3a,7a-diphenylhexahydro-4,7-methano-benzo[*d*][1,3,2]dioxaphosphole 2-oxide (**9wb**).

$[\alpha]_D^{25}$ = +148.98 °(c = 1.00 in MeOH);

The residue was purified by flash chromatography (PE/EtOAc = 10:1) on silica gel to afford the product (68 mg, 73% yield), white solid. R_f = 0.35 (PE/EA = 15:1).

m.p. = 158.0-159.0 ℃

^{1}H NMR (300 MHz, Chloroform-*d*) δ 7.75-6.87 (m, 10H), 4.21 (td, *J* = 12.8, 12.3, 3.5 Hz, 1H), 3.82 (ddd, *J* = 13.5, 8.0, 2.9 Hz, 1H), 3.04 (d, *J* = 5.4 Hz, 1H), 2.48 (dtd, *J* = 19.2, 9.9, 8.7, 3.8 Hz, 1H), 2.19-2.02 (m, 1H), 1.92 (tt, *J* = 14.9, 7.6 Hz, 1H), 1.76 (s, 3H), 1.68-1.41 (m, 2H), 1.32 (t, *J* = 7.6 Hz, 2H), 1.28-1.08 (m, 6H), 0.90 (s, 3H).

^{13}C NMR (75 MHz, Chloroform-*d*) δ 137.86 (d, *J* = 3.4 Hz), 135.97 (d, *J* = 4.1 Hz), 128.77, 128.51, 101.76, 99.00, 74.69 (d, *J* = 5.6 Hz), 55.74 (d, *J* = 3.2 Hz), 52.01 (d, *J* = 3.3 Hz), 48.28, 41.42, 39.73, 29.68, 26.45, 24.49, 22.15 (d, *J* = 5.3 Hz), 21.55, 12.41 (d, *J* = 4.3 Hz), 9.98.

^{31}P NMR (121 MHz, Chloroform-*d*) δ 39.52.

HRMS (ESI-MS) calculated for $C_{26}H_{33}NO_5P$ $[M+H]^+$: 470.2091, found: 470.2090.

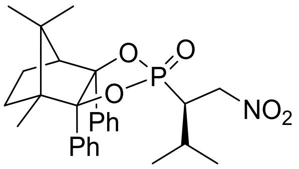

9wc

(3a*S*,4*S*,7*S*,7a*S*)-4,8,8-Trimethyl-2-((*R*)-3-methyl-1-nitrobutan-2-yl)-3a,7a-diphenylhexa-hydro-4,7-methanobenzo[*d*][1,3,2]dioxaphosphole 2-oxide (**9wc**).

$[\alpha]_D^{25}$ = +145.98 °(c = 1.00 in MeOH);

The residue was purified by flash chromatography (PE/EtOAc = 15:1) on silica gel to afford the product (73 mg, 75% yield), white solid. R_f = 0.35 (PE/EA = 15:1).

m.p. = 174.0-175.0 ℃

^{1}H NMR (300 MHz, Chloroform-*d*) δ 7.31 (d, *J* = 28.9 Hz, 10H), 4.36 (ddd, *J* = 14.0, 11.1, 4.6 Hz, 1H), 3.90 (ddd, *J* = 13.8, 10.4, 3.0 Hz, 1H), 3.04 (d, *J* = 5.4 Hz, 1H), 2.54 (dddd, *J* = 20.0, 11.2, 5.2, 3.1 Hz, 1H), 2.37-2.01 (m, 2H), 1.76 (s, 3H), 1.65-1.49 (m, 1H), 1.31 (td, *J* = 7.5, 6.7, 4.0 Hz, 2H), 1.21-1.06 (m, 9H), 0.90 (s, 3H).

^{13}C NMR (75 MHz, Chloroform-*d*) δ 137.81 (d, *J* = 3.4 Hz), 136.05 (d, *J* = 4.3 Hz), 128.70, 128.48, 101.90, 99.06 (d, *J* = 1.6 Hz), 72.59 (d, *J* = 5.0 Hz), 55.76 (d, *J* = 3.1 Hz), 52.05 (d, *J* = 3.2 Hz), 48.20, 45.37, 43.70, 29.74, 28.38 (d, *J* = 3.9 Hz), 26.53, 24.55, 21.46, 20.96 (d, *J* = 7.0 Hz), 19.79 (d, *J* = 6.5 Hz), 9.99.

^{31}P NMR (121 MHz, Chloroform-*d*) δ 39.08.

HRMS (ESI-MS) calculated for $C_{27}H_{35}NO_3P$ $[M+H]^+$: 484.2248, found: 484.2249.

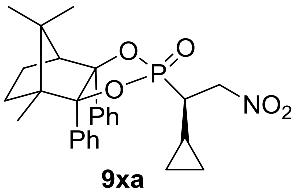

9xa

(3a*S*,4*S*,7*S*,7a*S*)-2-((*R*)-1-Cyclopropyl-2-nitroethyl)-4,8,8-trimethyl-3a,7a-diphenylhexahydro-4,7-methanobenzo[*d*][1,3,2]dioxaphosphole 2-oxide (**9xa**).

$[\alpha]_D^{25}$ = +102.98 °(c = 1.00 in MeOH);

The residue was purified by flash chromatography (PE/EtOAc = 15:1) on silica gel to afford the product (83 mg, 86% yield), white solid. R_f = 0.33 (PE/EA = 15:1).

m.p. = 140.0-141.0 ℃

^{1}H NMR (300 MHz, Chloroform-*d*) δ 7.64-6.66 (m, 10H), 4.25 (td, *J* = 12.3, 3.4 Hz, 1H), 3.79-3.66 (m, 1H), 2.91 (d, *J* = 5.3 Hz, 1H), 1.97 (tt, *J* = 10.4, 5.2 Hz, 1H), 1.77 (dt, *J* = 18.7, 10.4 Hz, 1H), 1.65 (s, 3H), 1.52-1.36 (m, 1H), 1.18 (qd, *J* = 11.8, 9.3, 4.7 Hz, 3H), 1.05 (s, 3H), 0.80 (s, 3H), 0.64 (q, *J* = 10.2, 7.5 Hz, 1H), 0.42 (h, *J* = 6.7, 5.4 Hz, 2H), 0.16--0.07 (m, 1H).

^{13}C NMR (75 MHz, Chloroform-*d*) δ 137.85 (d, *J* = 3.3 Hz), 135.96 (d, *J* = 4.3 Hz), 128.73, 128.54, 101.83, 99.12 (d, *J* = 1.4 Hz), 75.27 (d, *J* = 7.4 Hz), 55.73 (d, *J* = 3.1 Hz), 51.94 (d, *J* = 3.2 Hz), 48.17, 44.74, 43.02, 29.78, 26.45, 24.45, 21.51, 9.95, 9.88, 4.04 (d, *J* = 14.0 Hz), 2.69.

^{31}P NMR (121 MHz, Chloroform- *d*) δ 37.19.

HRMS (ESI-MS) calculated for $C_{27}H_{33}NO_5P$ $[M+H]^+$: 482.2091, found: 482.2093.

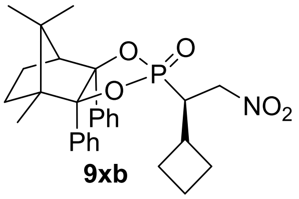

9xb

(3a*S*,4*S*,7*S*,7a*S*)-2-((*R*)-1-Cyclobutyl-2-nitroethyl)-4,8,8-trimethyl-3a,7a-diphenylhexahydro-4,7-methanobenzo[*d*][1,3,2]dioxaphosphole 2-oxide (**9xb**).

$[\alpha]_D^{25}$ = +219.98 °(c = 1.00 in MeOH);

The residue was purified by flash chromatography (PE/EtOAc = 15:1) on silica gel to afford the product (86 mg, 87% yield), white solid. R_f = 0.35 (PE/EA = 15:1).

m.p. = 173.0-174.0 ℃

^{1}H NMR (300 MHz, Chloroform-*d*) δ 7.51-7.00 (m, 10H), 4.64 (td, *J* = 12.6, 11.5, 5.0 Hz, 1H), 4.38 (td, *J* = 13.1, 8.2 Hz, 1H), 3.19 (p, *J* = 8.6, 7.8 Hz, 1H), 2.97 (d, *J* = 5.4 Hz, 1H), 2.85 (q, *J* = 8.6 Hz, 1H), 2.47 (dq, *J* = 13.3, 6.9 Hz, 1H), 2.32-2.16 (m, 1H), 2.16-1.94 (m, 3H), 1.88 (t, *J* = 7.1 Hz, 2H), 1.75 (dt, *J* = 18.5, 9.4 Hz, 1H), 1.58 (s, 3H), 1.40-1.26 (m, 2H), 1.17 (s, 3H), 0.88 (s, 3H).

^{13}C NMR (75 MHz, Chloroform-*d*) δ 137.31, 136.48, 128.10, 127.73, 127.17, 100.97 (d, *J* = 2.3 Hz), 98.51 (d, *J* = 3.4 Hz), 73.93 (d, *J* = 1.4 Hz), 55.45 (d, *J* = 5.5 Hz), 51.61 (d, *J* = 5.5 Hz), 47.98, 43.98, 42.27, 35.47 (d, *J* = 5.2 Hz), 29.48, 28.78 (d, *J* = 4.2 Hz), 27.95, 27.74, 25.92, 24.70, 21.86, 18.87, 10.05.

^{31}P NMR (121 MHz, Chloroform-*d*) δ 40.02.

HRMS (ESI-MS) calculated for $C_{28}H_{35}NO_5P$ $[M+H]^+$: 496.2248, found: 496.2249.

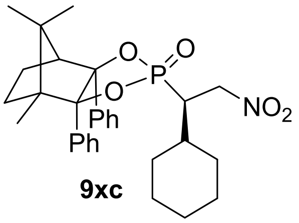

9xc

(3a*S*,4*S*,7*S*,7a*S*)-2-((*R*)-1-Cyclohexyl-2-nitroethyl)-4,8,8-trimethyl-3a,7a-diphenylhexahydro-4,7-methanobenzo[*d*][1,3,2]dioxaphosphole 2-oxide (**9xc**).

$[\alpha]_D^{25}$ = +231.99 °(c = 1.00 in MeOH);

The residue was purified by flash chromatography (PE/EtOAc = 15:1) on silica gel to afford the product (91 mg, 87% yield), white solid. R_f = 0.38 (PE/EA = 15:1).

m.p. = 192.0-193.0 ℃

^{1}H NMR (300 MHz, Chloroform-*d*) δ 7.54-6.99 (m, 10H), 4.38 (ddd, *J* = 14.2, 10.8, 5.4 Hz, 1H), 3.93 (ddd, *J* = 14.2, 10.9, 3.2 Hz, 1H), 3.04 (d, *J* = 5.3 Hz, 1H), 2.64-2.46 (m, 1H), 2.09 (ddt, *J* = 15.1, 10.3, 5.4 Hz, 1H), 1.97-1.43 (m, 11H), 1.43-1.20 (m, 6H), 1.17 (s, 3H), 0.91 (s, 3H).

^{13}C NMR (75 MHz, Chloroform-*d*) δ 137.80 (d, *J* = 3.3 Hz), 136.11 (d, *J* = 4.2 Hz), 128.69, 128.47, 101.86, 99.02 (d, *J* = 1.6 Hz), 72.42 (d, *J* = 4.7 Hz), 55.77 (d, *J* = 3.2 Hz), 52.04 (d, *J* = 3.5 Hz), 48.23, 45.20, 43.54, 38.25 (d, *J* = 3.8 Hz), 31.59 (d, *J* = 7.3 Hz), 30.10-29.63 (m), 26.82, 26.55 (d, *J* = 3.6 Hz), 25.96, 24.54, 21.49, 10.02.

^{31}P NMR (121 MHz, Chloroform-*d*) δ 39.72.

HRMS (ESI-MS) calculated for $C_{30}H_{39}NO_5P$ $[M+H]^+$: 524.2561, found: 524.2558.

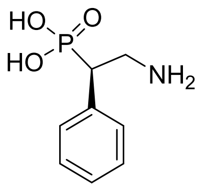

10a

(*R*)-(2-Amino-1-phenylethyl)phosphonic acid [14] (**10a**).

$[\alpha]_D^{25}$ = +32 °(c = 1.00 in MeOH);

38 mg, 95% yield, white solid,

m.p. = 120.0-121.0 ℃

^{1}H NMR (300 MHz, Deuterium Oxide) δ 7.28 (s, 5H), 3.77-3.00 (m, 3H).

^{13}C NMR (75 MHz, Deuterium Oxide) δ 129.18, 128.92, 128.32, 39.77, 37.72.

^{31}P NMR (121 MHz, Deuterium Oxide) δ 19.86.

HRMS (ESI-MS) calculated for $C_8H_{13}NO_3P$ $[M+H]^+$: 202.0628, found: 202.0627.

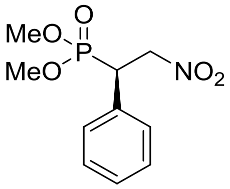

10aa

dimethyl (R)-(2-nitro-1-phenylethyl)phosphonate (10aa)

47 mg, 90% yield, white solid,

^{1}H NMR (300 MHz, Chloroform-*d*) δ 7.37 (d, *J* = 4.2 Hz, 5H), 4.95 (t, *J* = 7.5 Hz, 2H), 4.06 (dt, *J* = 24.0, 7.8 Hz, 1H), 3.73 (d, *J* = 10.9 Hz, 3H), 3.51 (d, *J* = 10.7 Hz, 3H).

^{13}C NMR (75 MHz, Chloroform-*d*) δ 131.39 (d, *J* = 7.4 Hz), 129.20 (d, *J* = 2.5 Hz), 128.95 (d, *J* = 6.1 Hz), 128.67 (d, *J* = 3.1 Hz), 75.11 (d, *J* = 4.0 Hz), 54.23 (d, *J* = 7.0 Hz), 53.31 (d, *J* = 7.4 Hz), 43.64, 41.80.

^{31}P NMR (121 MHz, Chloroform-*d*) δ 24.54.

3.5 Characterization data of Menthyl-PHO, TADDOL-PHO adducts

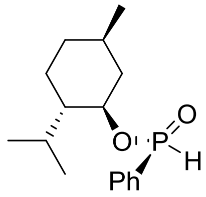

Menthyl-PHO

^{1}H NMR (300 MHz, Chloroform-*d*) δ 7.66 (d, *J* = 553.3 Hz, 1H), 7.78 (ddt, *J* = 14.0, 6.8, 1.5 Hz, 2H), 7.66 (d, *J* = 552 Hz, 1H), 7.65 – 7.55 (m, 1H), 7.51 (ddd, *J* = 8.5, 6.4, 3.5 Hz, 2H), 4.29 (qd, *J* = 10.4, 4.5 Hz, 1H), 2.30 – 2.12 (m, 2H), 1.68 (tq, *J* = 9.5, 3.4 Hz, 2H), 1.56 – 1.36 (m, 2H), 1.24 (td, *J* = 12.2, 10.8 Hz, 1H), 1.15 – 1.01 (m, 1H), 0.96 (d, *J* = 7.0 Hz, 3H), 0.88 (dd, *J* = 9.4, 6.7 Hz, 7H).

^{31}P NMR (121 MHz, Chloroform-*d*) δ 24.68.

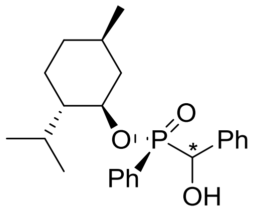

(1*R*,2*S*,5*R*)-2-isopropyl-5-methylcyclohexyl-(*S*)-((*S*)-hydroxy(phenyl)methyl)(phenyl)-phosphinate[15].

The residue was purified by flash chromatography (PE/EtOAc = 3:1) on silica gel to afford the product (69 mg, 45% yield), white solid. R_f = 0.38 (PE/EA = 3:1).

^{1}H NMR (300 MHz, Methanol-*d*$_4$) δ 7.95 – 7.07 (m, 10H), 5.10 (d, *J* = 10.2 Hz, 1H), 4.65 (s, 1H), 4.46 – 3.96 (m, 1H), 1.85 (td, *J* = 15.0, 6.3 Hz, 1H), 1.76 – 1.56 (m, 3H), 1.37 – 1.23 (m, 2H), 1.09 – 0.93 (m, 2H), 0.93 – 0.66 (m, 8H), 0.62 (d, *J* = 6.9 Hz, 2H).

^{13}C NMR (75 MHz, Methanol-*d*$_4$) δ 138.55 (d, *J* = 1.5 Hz), 138.41, 134.00, 133.93 – 133.54 (m), 132.11, 130.47, 129.32, 129.26 – 128.69 (m), 128.53 (d, *J* = 5.0 Hz), 79.00 (d, *J* = 8.4 Hz), 78.71 (d, *J* = 8.3 Hz), 75.27, 74.34, 73.69, 72.73, 44.46, 35.08, 32.76 (d, *J* = 1.3 Hz), 26.18, 26.03, 23.69 (d, *J* = 4.1 Hz), 22.30 (d, *J* = 1.4 Hz), 21.52, 15.79 (d, *J* = 4.5 Hz).

^{31}P NMR (121 MHz, Methanol-*d*$_4$) δ 36.21, 36.11.

HRMS (ESI-MS) calculated for $C_{23}H_{32}O_3P$ $[M+H]^+$: 387.2089, found:387.2090.

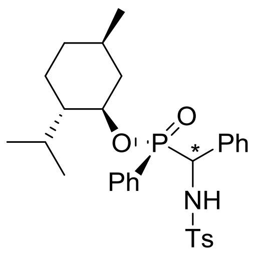

(1*R*,2*S*,5*R*)-2-isopropyl-5-methylcyclohexyl-(*S*)-((*S*)-((4-methylphenyl)sulfonamido)-(phenyl)methyl)(phenyl)phosphinate[16].

The residue was purified by flash chromatography (PE/EtOAc = 5:1) on silica gel to afford the product (75 mg, 35% yield), white solid. R_f = 0.38 (PE/EA = 5:1).

^{1}H NMR (300 MHz, Chloroform-*d*) δ 7.53 – 7.36 (m, 3H), 7.34 (d, *J* = 8.2 Hz, 1H), 7.33 – 7.18 (m, 3H), 6.92 (tt, *J* = 4.2, 2.4 Hz, 1H), 6.83 (q, *J* = 6.7, 5.4 Hz, 5H), 6.51 (t, *J* = 7.7 Hz, 1H), 4.81 (dd, *J* = 17.7, 9.3 Hz, 1H), 4.52 (tdd, *J* = 10.8, 6.5, 4.4 Hz, 1H), 2.53 – 2.37 (m, 1H), 2.21 (s, 3H), 1.94 – 1.84 (m, 1H), 1.77 – 1.57 (m, 2H), 1.56 – 1.34 (m, 2H), 1.31 – 1.21 (m, 1H), 1.17 – 1.03 (m, 2H), 0.98 (s, 3H), 0.93 (d, *J* = 6.8 Hz, 3H), 0.86 (dd, *J* = 12.2, 2.9 Hz, 1H), 0.75 (d, *J* = 6.5 Hz, 3H).

^{13}C NMR (75 MHz, Chloroform-*d*) δ 142.63, 137.85, 133.03, 132.42, 132.12 (d, *J* = 9.4 Hz), 130.78, 128.95, 128.40 – 128.07 (m), 127.98, 127.80 (d, *J* = 2.5 Hz), 127.18 (d, *J* = 6.3 Hz), 78.95 (d, *J* = 8.2 Hz), 59.28, 57.91, 49.05 (d, *J* = 6.4 Hz), 43.48, 34.17, 31.53, 25.49, 22.87, 22.06, 21.35 (d, *J* = 13.9 Hz), 15.90.

^{31}P NMR (121 MHz, Chloroform-*d*) δ 35.00, 33.55.

HRMS (ESI-MS) calculated for $C_{30}H_{39}NO_4PS$ $[M+H]^+$:540.2337, found: .540.2341.

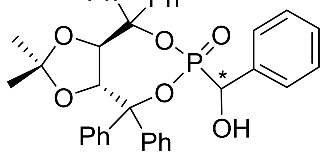

(3a*R*,8a*R*)-6-((*R*)-Hydroxy(phenyl)methyl)-2,2-dimethyl-4,4,8,8-tetraphenyltetrahydro-[1,3]dioxolo-[4,5-e][1,3,2]dioxaphosphepine 6-oxide[17].

The residue was purified by flash chromatography (PE/EtOAc = 3:1) on silica gel to afford the product (185 mg, 75% yield), white solid. R_f = 0.30 (PE/EA = 3:1).

^{1}H NMR (300 MHz, Chloroform-*d*) δ 7.56-7.47 (m, 2H), 7.46-7.39 (m, 2H), 7.32-7.13 (m, 12H), 7.09-6.95 (m, 7H), 6.61-6.52 (m, 2H), 5.26 (d, *J* = 7.9 Hz, 1H), 4.99 (d, *J* = 8.6 Hz, 1H), 4.77 (d, *J* =

7.9 Hz, 1H), 4.20 (s, 1H), 0.65 (s, 3H), 0.35 (s, 3H).

^{13}C NMR (75 MHz, Chloroform-*d*) δ 144.32 (d, *J* = 6.9 Hz), 143.45, 139.58, 139.45, 136.33 (d, *J* = 4.8 Hz), 130.03, 128.65, 128.37-128.20 (m), 128.15-127.98 (m), 127.83, 127.81, 127.55, 127.51, 127.28, 127.14, 126.88 (d, *J* = 5.3 Hz), 126.56, 113.83, 91.28 (d, *J* = 14.7 Hz), 86.36 (d, *J* = 9.7 Hz), 79.18 (dd, *J* = 12.4, 2.6 Hz), 72.09, 69.90, 27.05, 26.47.

^{31}P NMR (121 MHz, Chloroform-*d*) δ 16.36.

HRMS (ESI-MS) calculated for $C_{38}H_{36}O_6P$ $[M+H]^+$: 619.2244, found: .619.2241.

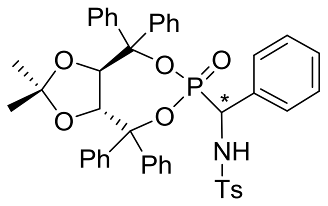

***N*-((*R*)-((3a*R*,8a*R*)-2,2-Dimethyl-6-oxido-4,4,8,8-tetraphenyltetrahydro-[1,3]dioxolo[4,5-e][1,3,2]dio-xaphosphepin-6-yl)(phenyl)methyl)-4-methylbenzenesulfonamide**[18-19].

The residue was purified by flash chromatography (PE/EtOAc = 5:1) on silica gel to afford the product (154 mg, 50% yield), white solid. R_f = 0.35 (PE/EA = 5:1).

^1H NMR (300 MHz, Chloroform-*d*) δ 7.51-7.28 (m, 13H), 7.24-6.90 (m, 16H), 5.65 (t, *J* = 7.6 Hz, 1H), 5.41 (d, *J* = 7.9 Hz, 1H), 5.08 (d, *J* = 7.9 Hz, 1H), 4.90 (dd, *J* = 24.6, 8.4 Hz, 1H), 2.24 (s, 3H), 0.75 (s, 3H), 0.54 (s, 3H).

^{13}C NMR (75 MHz, Chloroform-*d*) δ 144.23, 143.49, 143.33, 139.11, 133.60, 129.75, 129.42, 128.77, 128.63, 128.55, 128.45, 128.40, 128.31, 127.87, 127.41, 127.38, 127.32, 127.07, 126.77, 114.07, 92.09, 91.91, 87.38, 79.67, 79.05, 57.18, 27.01, 26.60, 21.51.

^{31}P NMR (121 MHz, Chloroform-*d*) δ 13.17, 13.11.

HRMS (ESI-MS) calculated for $C_{45}H_{43}NO_7PS$ $[M+H]^+$: 772.2492, found: 772.2489.

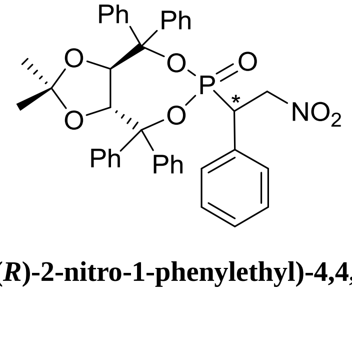

(3a*R*,8a*R*)-2,2-Dimethyl-6-((*R*)-2-nitro-1-phenylethyl)-4,4,8,8-tetraphenyltetrahydro-

[1,3]dioxolo-[4,5-e][1,3,2]dioxaphosphepine 6-oxide[20].

The residue was purified by flash chromatography (PE/EtOAc = 8:1) on silica gel to afford the product (106 mg, 40% yield), white solid. R_f = 0.35 (PE/EA = 8:1).

^{1}H NMR (300 MHz, Chloroform-*d*) δ 7.54-7.45 (m, 2H), 7.29 (s, 5H), 7.24-7.07 (m, 16H), 6.90-6.80 (m, 2H), 5.39 (d, *J* = 7.9 Hz, 1H), 4.94 (dd, *J* = 8.0, 6.6 Hz, 3H), 4.05 (dt, *J* = 26.0, 7.9 Hz, 1H), 0.73 (s, 3H), 0.35 (s, 3H).

^{13}C NMR (75 MHz, Chloroform-*d*) δ 143.98 (d, *J* = 7.1 Hz), 143.32, 139.29 (dd, *J* = 5.9, 4.1 Hz), 131.62 (d, *J* = 8.9 Hz), 129.74, 129.07-128.79 (m), 128.62, 128.60, 128.45, 128.05, 127.88, 127.49-127.25 (m), 126.49, 114.06, 91.35 (d, *J* = 12.5 Hz), 87.53 (d, *J* = 7.9 Hz), 79.53 (d, *J* = 2.4 Hz), 79.01 (d, *J* = 2.5 Hz), 74.24 (d, *J* = 6.9 Hz), 45.12, 43.19, 27.06, 26.33.

^{31}P NMR (121 MHz, Chloroform-*d*) δ 15.90, 15.65.

HRMS (ESI-MS) calculated for $C_{39}H_{37}NO_7P$ $[M+H]^+$: 662.2302, found: 662.2300.

3.6 Characterization data of the recovered epoxide-CAMDOL

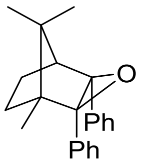

epoxide-CAMDOL

Epoxide-CAMDOL[1]

^{1}H NMR (300 MHz, Chloroform-d) δ 7.26 (dd, *J* = 4.9, 2.0 Hz, 3H), 7.13 (dq, *J* = 6.5, 4.2, 3.3 Hz, 5H), 7.04-6.93 (m, 2H), 2.57 (d, *J* = 4.0 Hz, 1H), 1.99 (ddt, *J* = 16.7, 10.0, 3.8 Hz, 1H), 1.82 (ddd, *J* = 12.8, 9.0, 4.4 Hz, 1H), 1.68 (ddd, *J* = 12.8, 9.0, 3.6 Hz, 1H), 1.46-1.31 (m, 4H), 0.92 (d, *J* = 2.0 Hz, 6H).

^{13}C NMR (75 MHz, Chloroform-*d*) δ 135.61, 132.90, 130.32, 128.10, 127.77, 127.66, 127.59, 127.16, 74.77, 67.77, 52.16, 49.05, 43.11, 30.77, 26.03, 23.64, 23.22, 11.62.

4. Copies of NMR spectra

^{1}H NMR (300 MHz, $CDCl_3$)

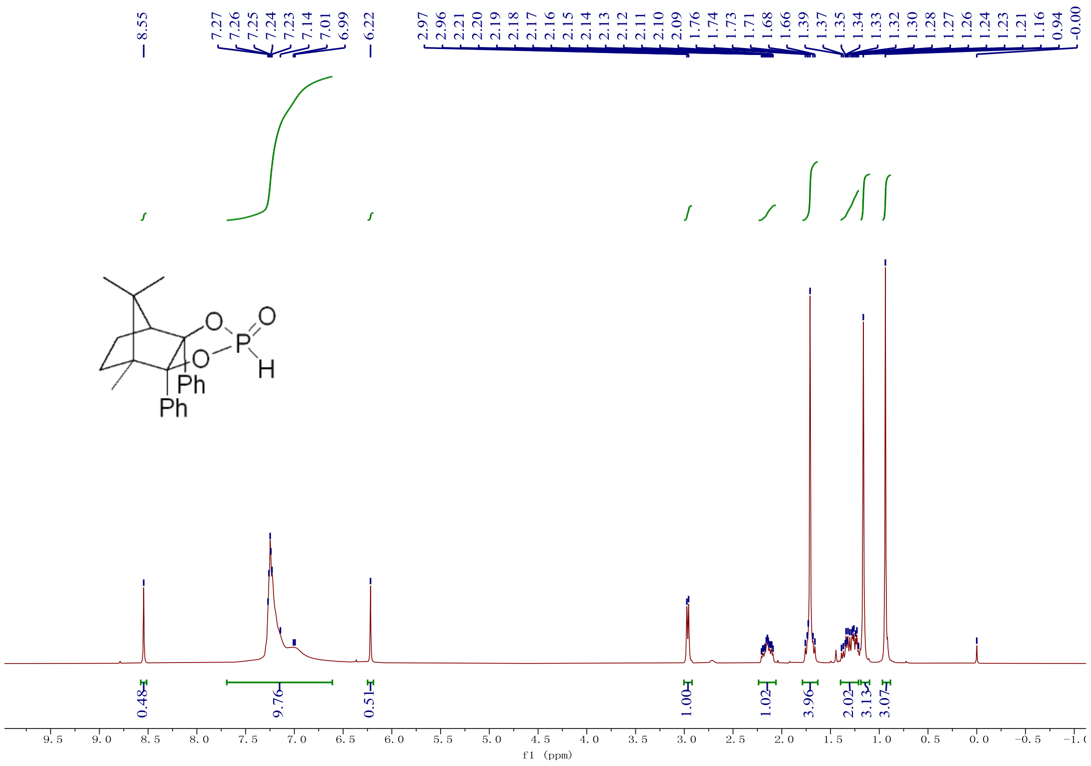

Fig. S1 ^{1}H NMR of compound **1**

^{13}C NMR (75 MHz, $CDCl_3$)

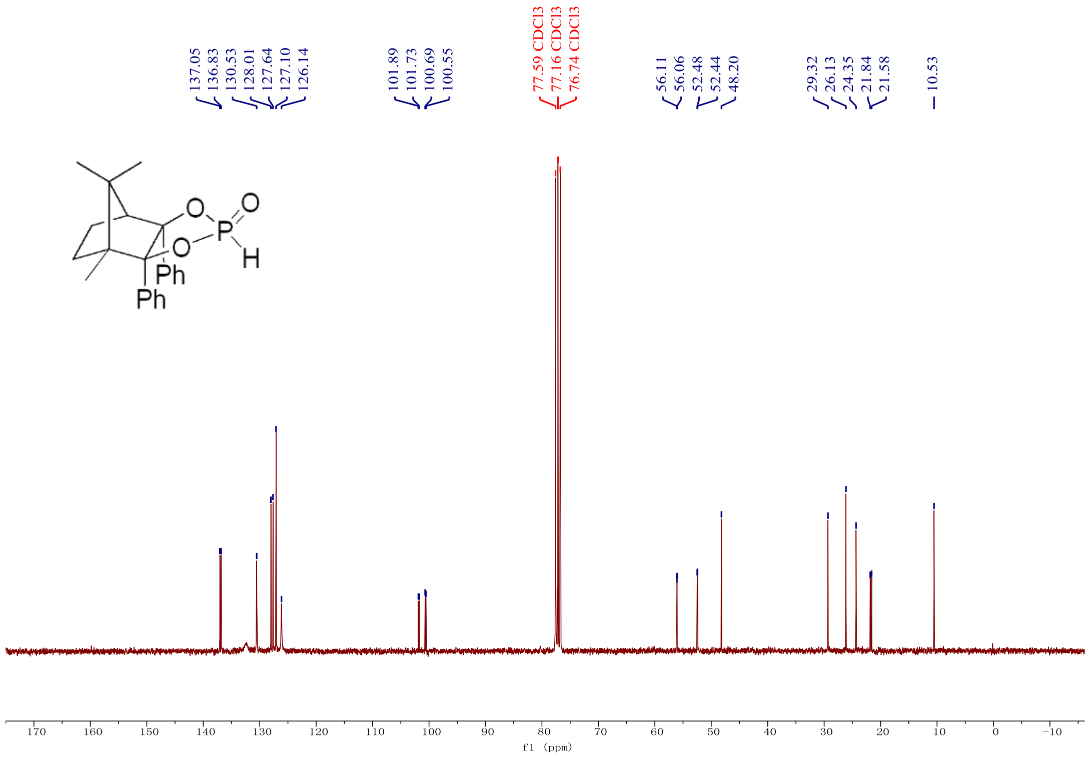

Fig. S2 ^{13}C NMR of compound **1**

^{31}P NMR (121 MHz, $CDCl_3$)

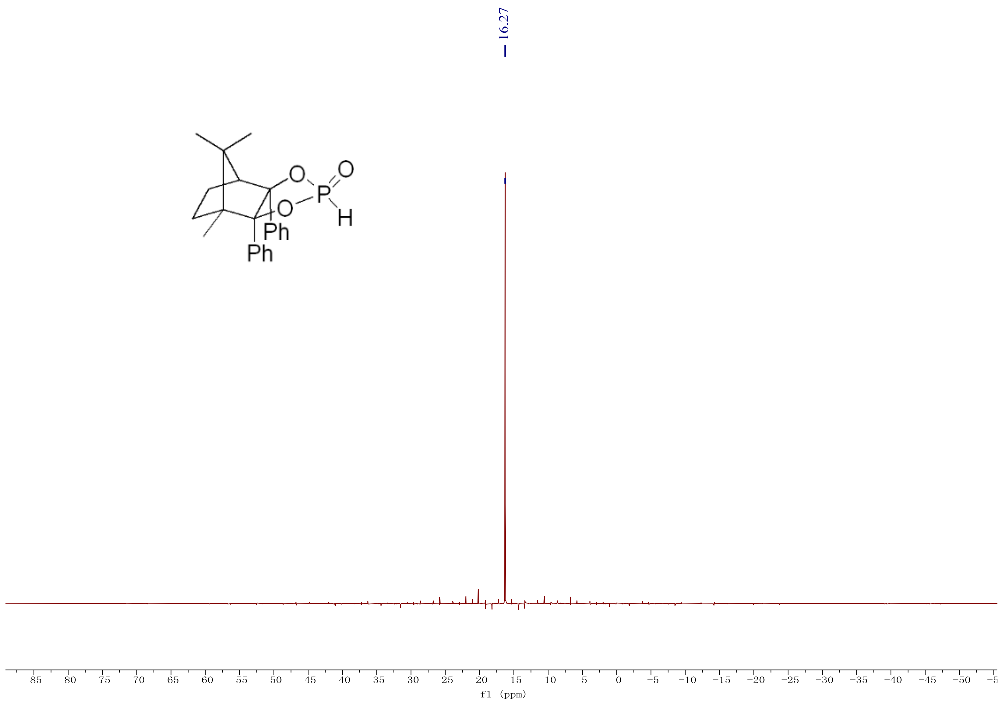

Fig. S3 ^{13}P NMR of compound **1**

^{1}H NMR (300 MHz, $CDCl_3$)

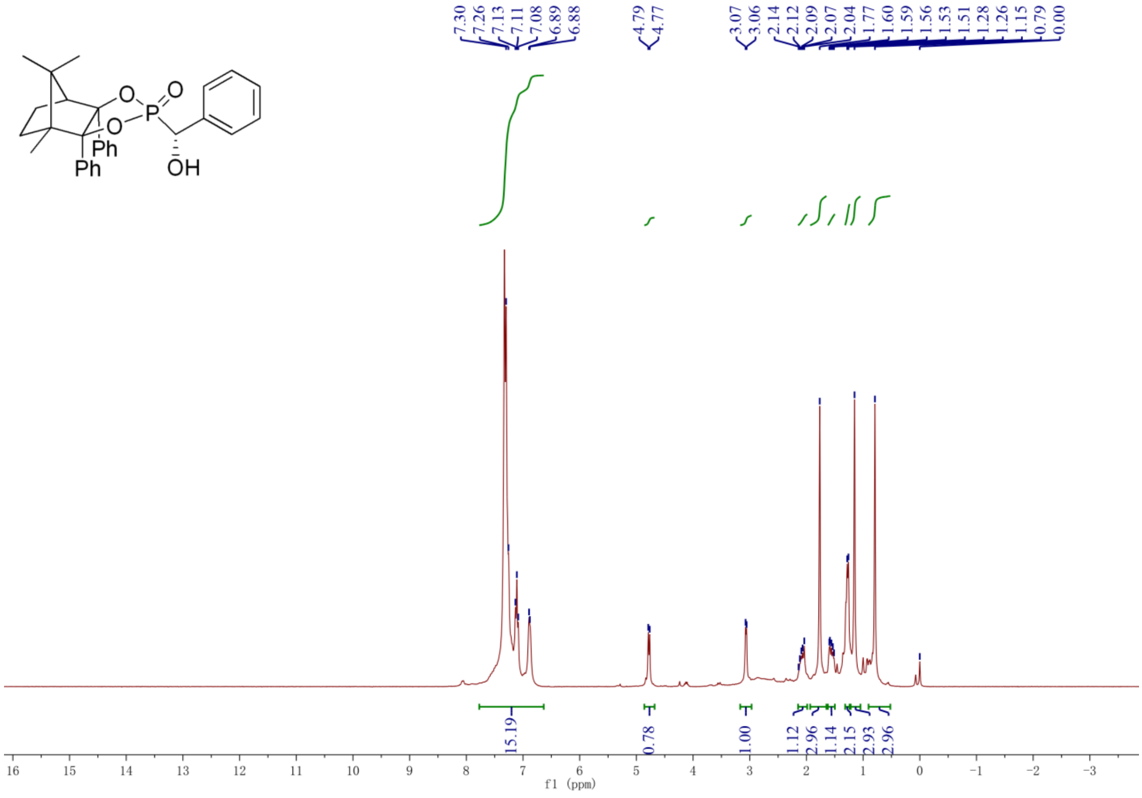

Fig. S4 ^{1}H NMR of compound **3a**

^{13}C NMR (75 MHz, $CDCl_3$)

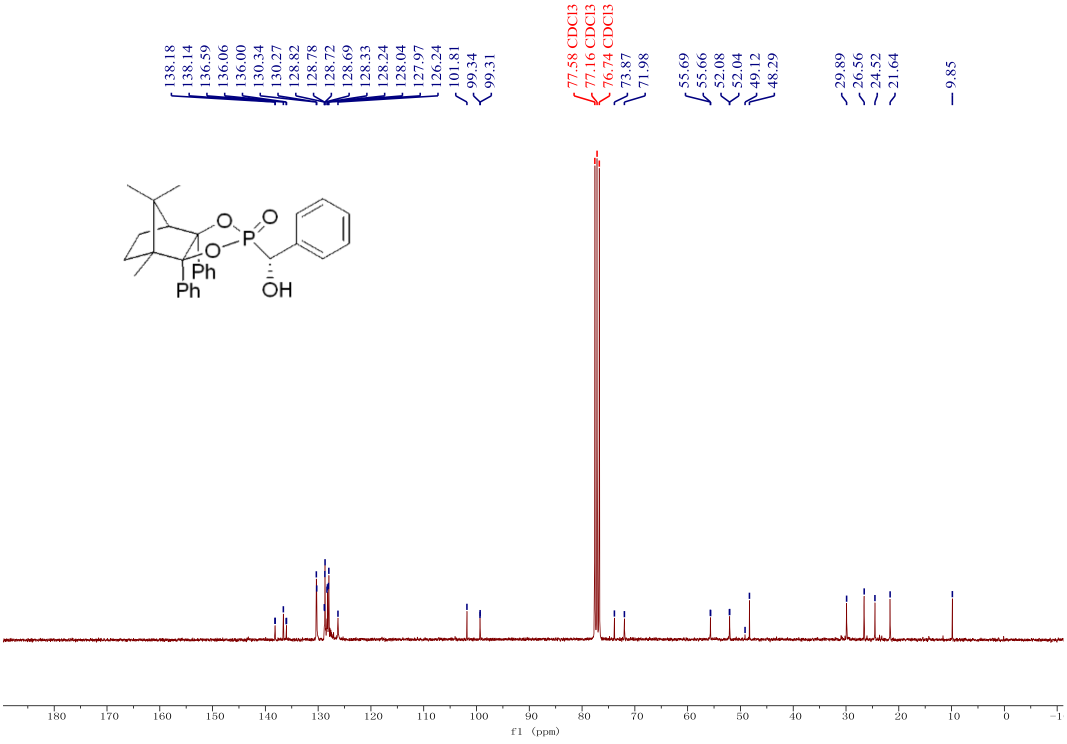

Fig. S5 ^{13}C NMR of compound **3a**

^{31}P NMR (121 MHz, $CDCl_3$)

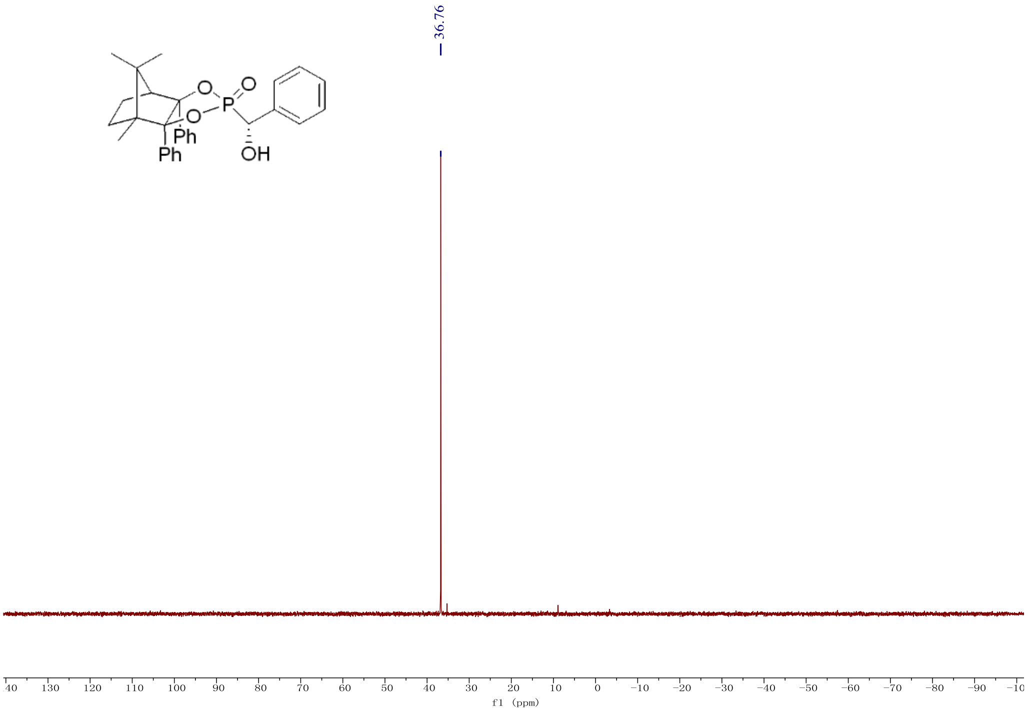

Fig. S6 ^{31}P NMR of compound **3a**

^{1}H NMR (300 MHz, $CDCl_3$)

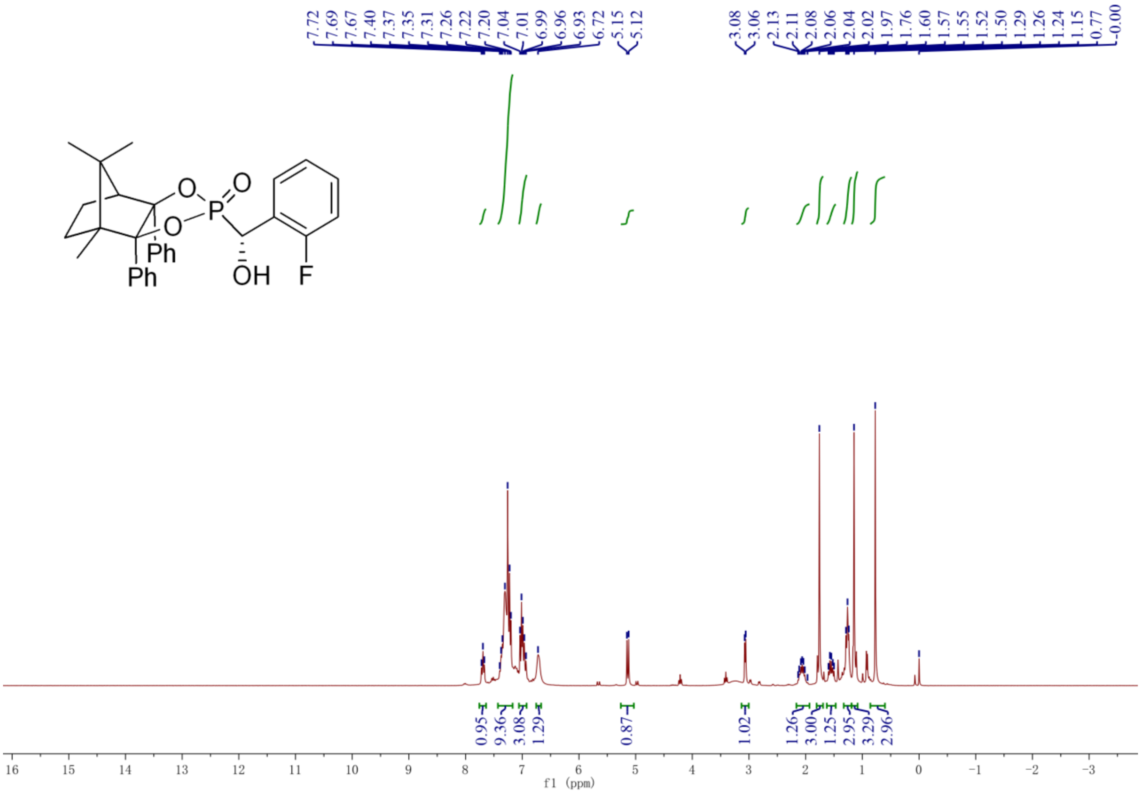

Fig. S7 ^{1}H NMR of compound **3b**

^{13}C NMR (75 MHz, $CDCl_3$)

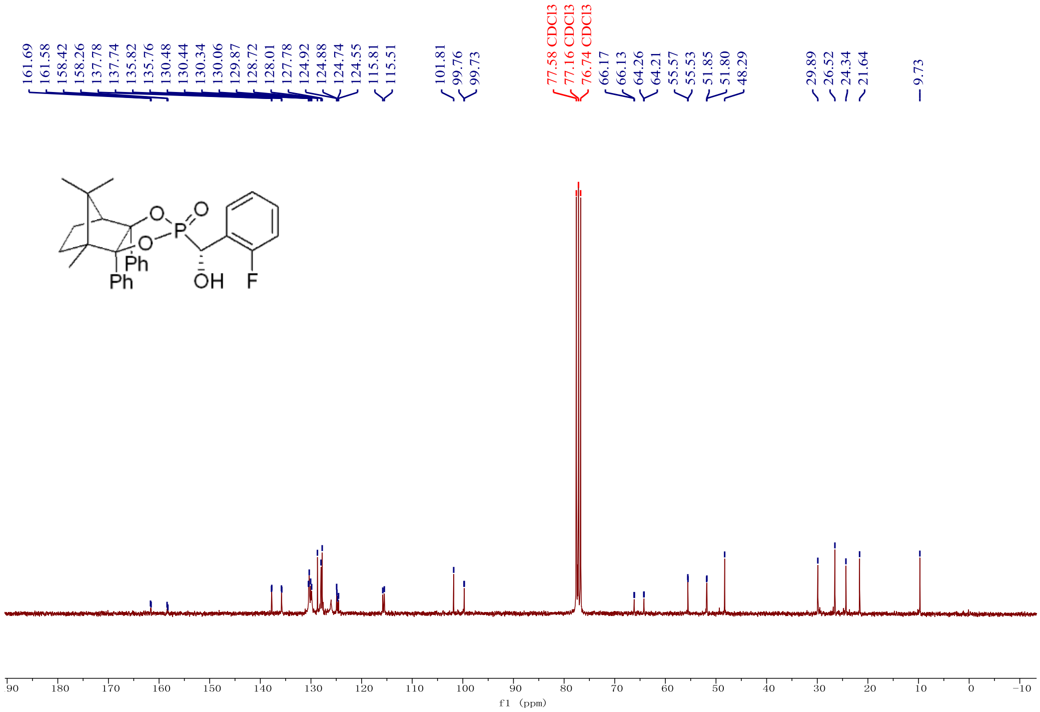

Fig. S8 ^{13}C NMR of compound **3b**

^{19}F NMR (282 MHz, $CDCl_3$)

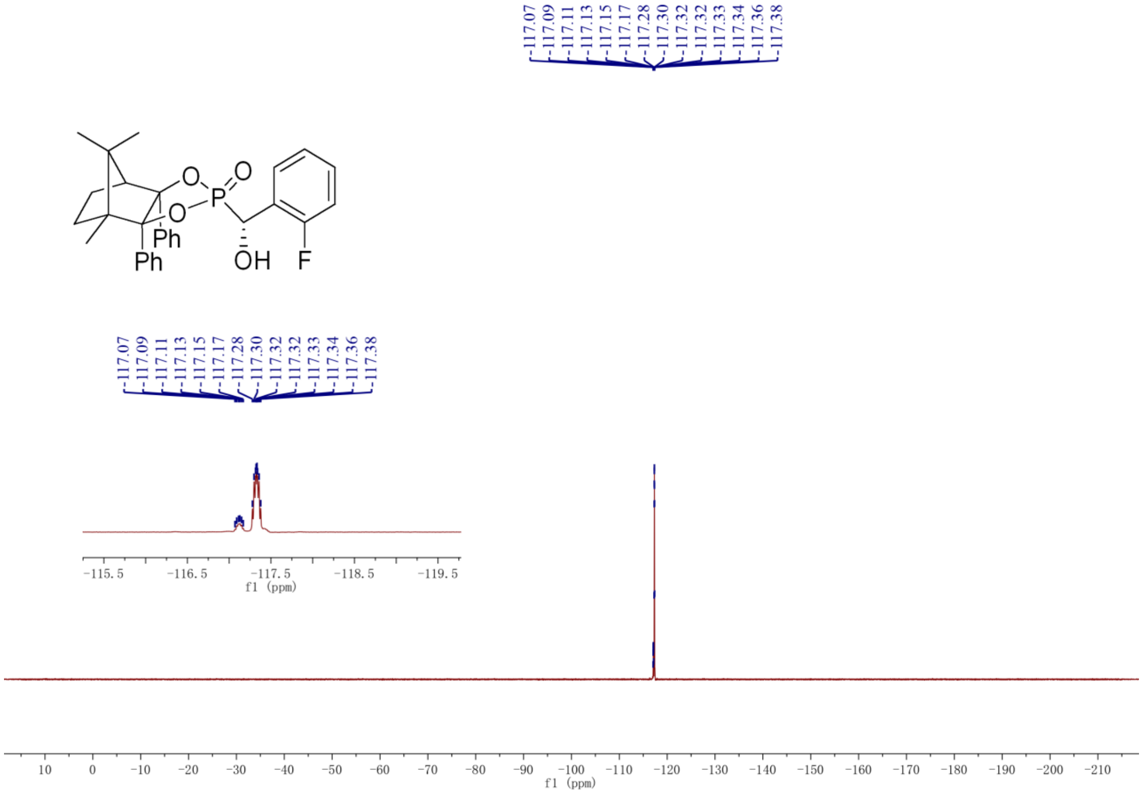

Fig. S9 ^{19}F NMR of compound **3b**

^{31}P NMR (121 MHz, $CDCl_3$)

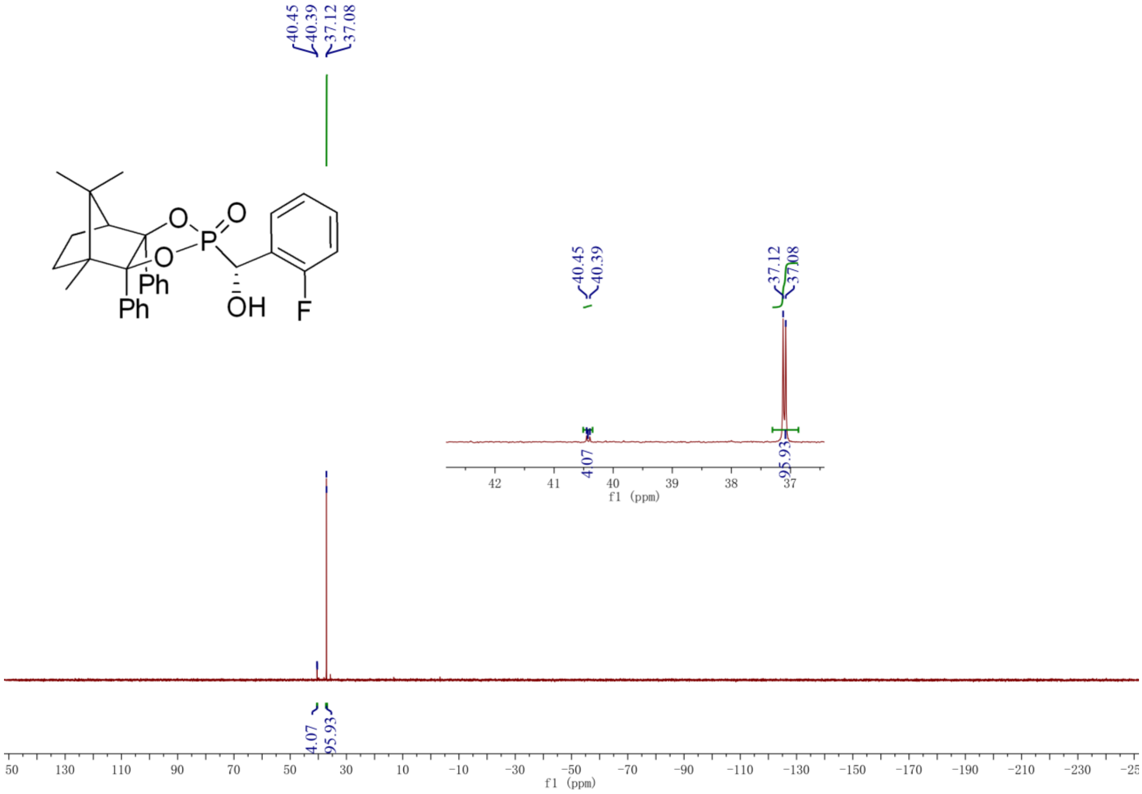

Fig. S10 ^{31}P NMR of compound **3b**

^{1}H NMR (300 MHz, $CDCl_3$)

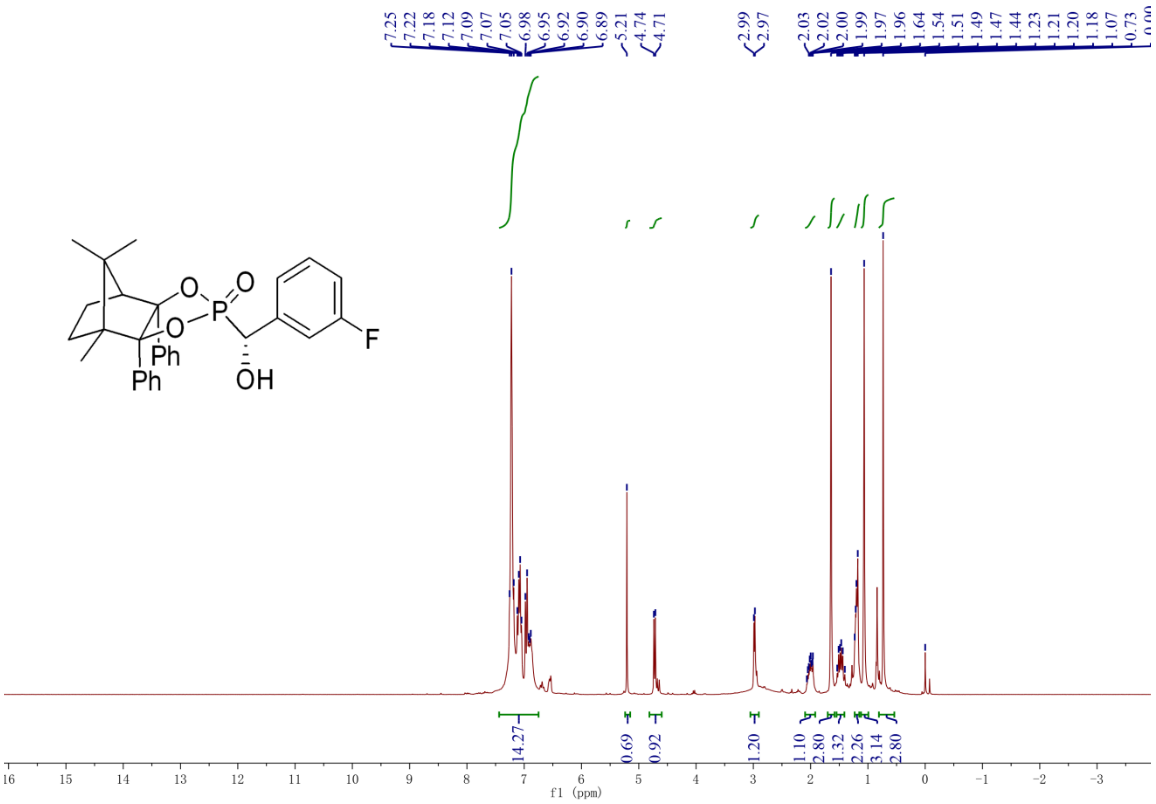

Fig. S11 ^{1}H NMR of compound **3c**

^{13}C NMR (75 MHz, $CDCl_3$)

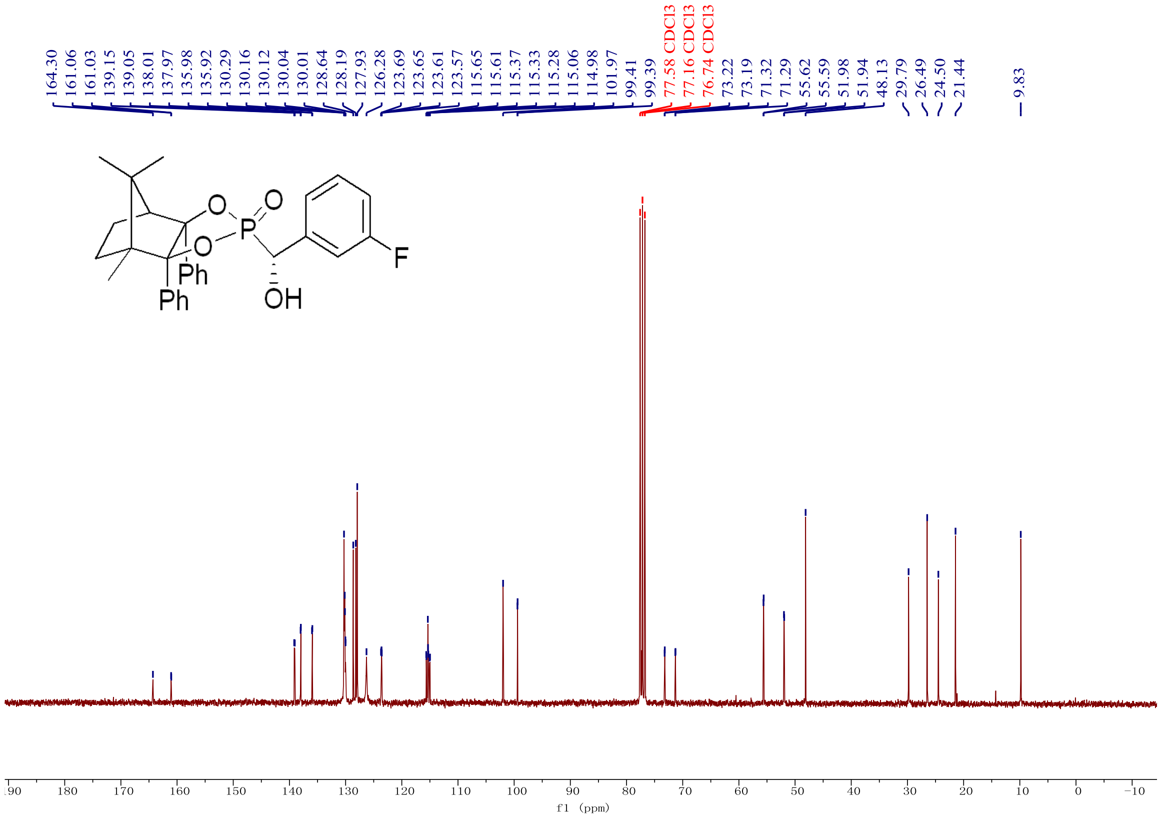

Fig. S12 ^{13}C NMR of compound **3c**

^{19}F NMR (282 MHz, $CDCl_3$)

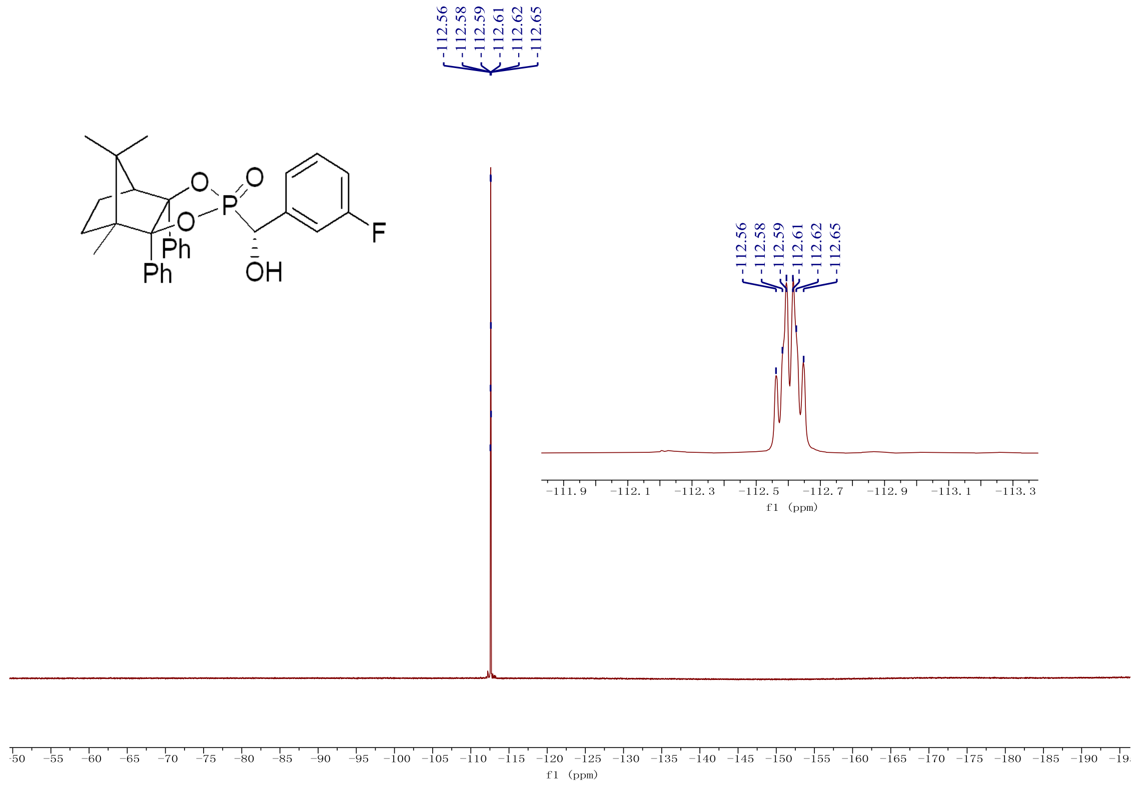

Fig. S13 ^{19}F NMR of compound **3c**

^{31}P NMR (121 MHz, $CDCl_3$)

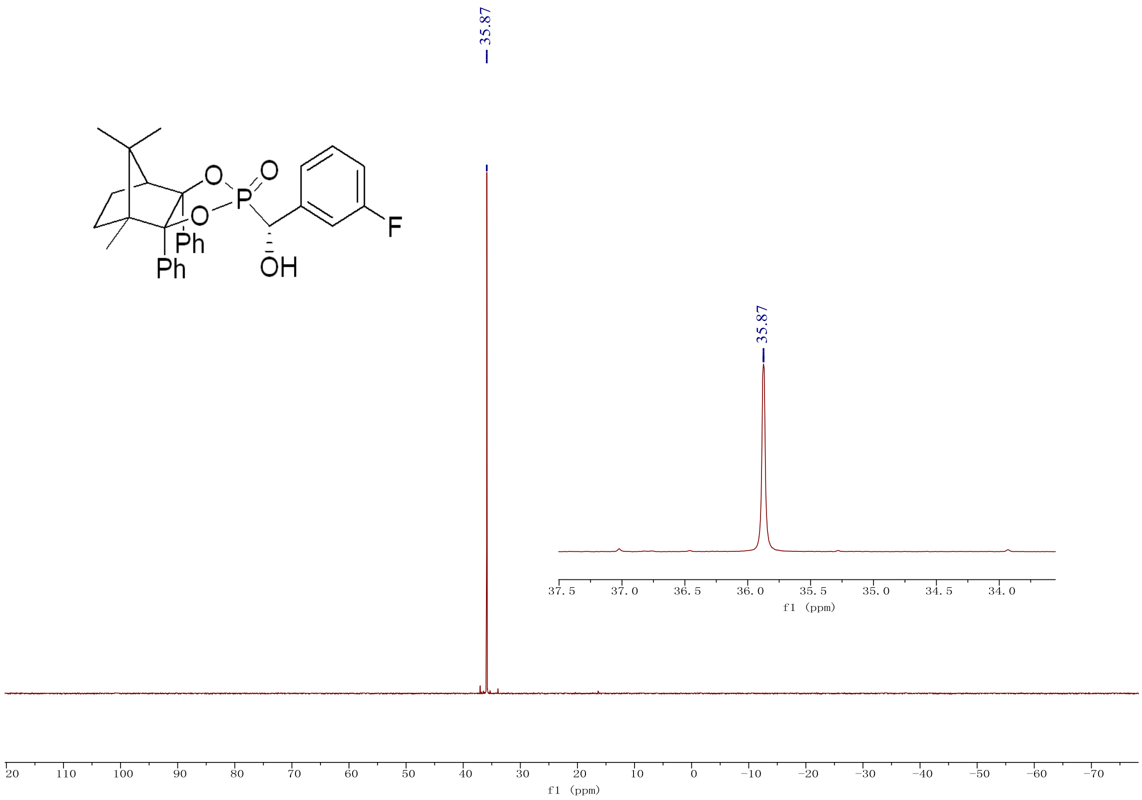

Fig. S14 ^{31}P NMR of compound **3c**

^{1}H NMR (300 MHz, $CDCl_3$)

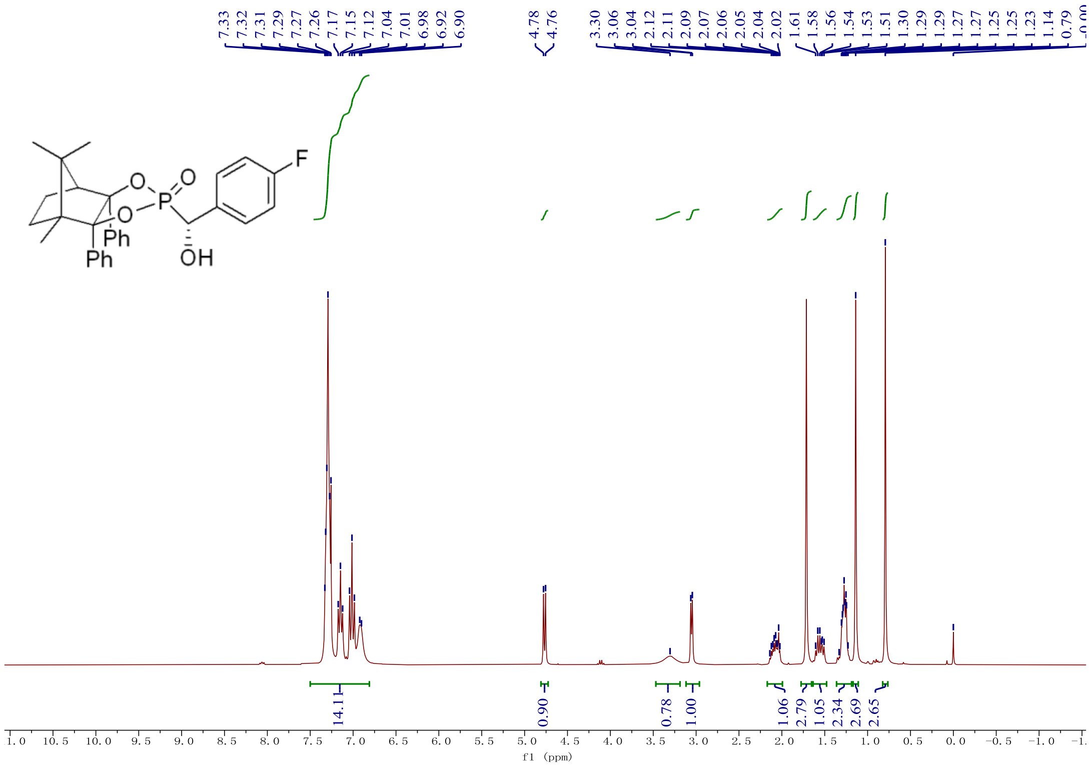

Fig. S15 ^{1}H NMR of compound **3d**

^{13}C NMR (75 MHz, $CDCl_3$)

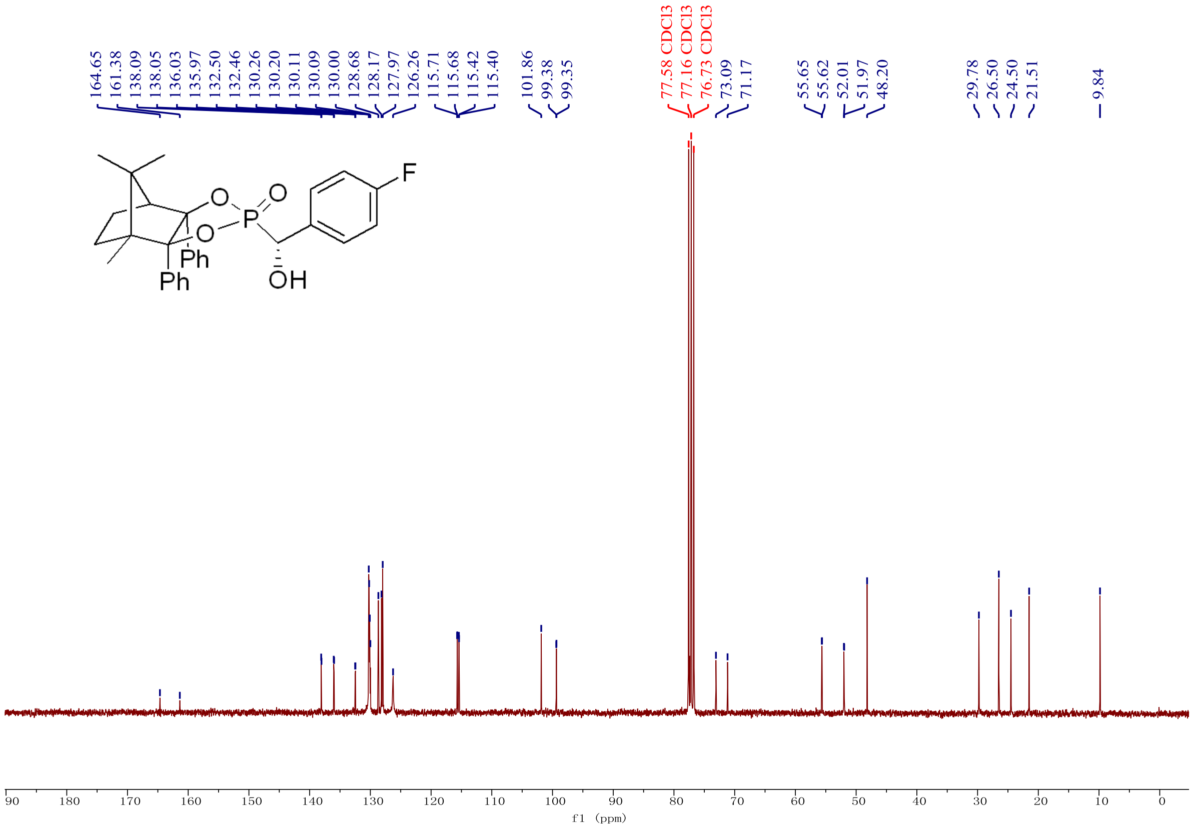

Fig. S16 ^{13}C NMR of compound **3d**

^{19}F NMR (282 MHz, $CDCl_3$)

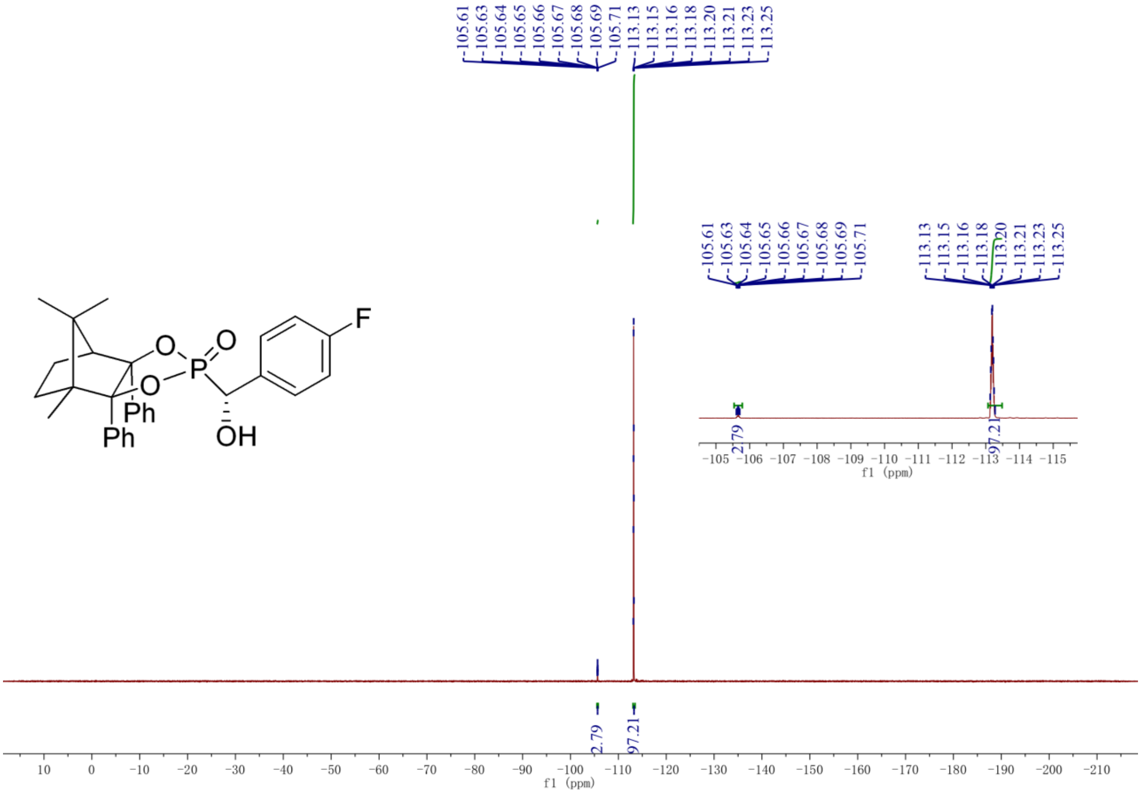

Fig. S17 ^{19}F NMR of compound **3d**

^{31}P NMR (121 MHz, $CDCl_3$)

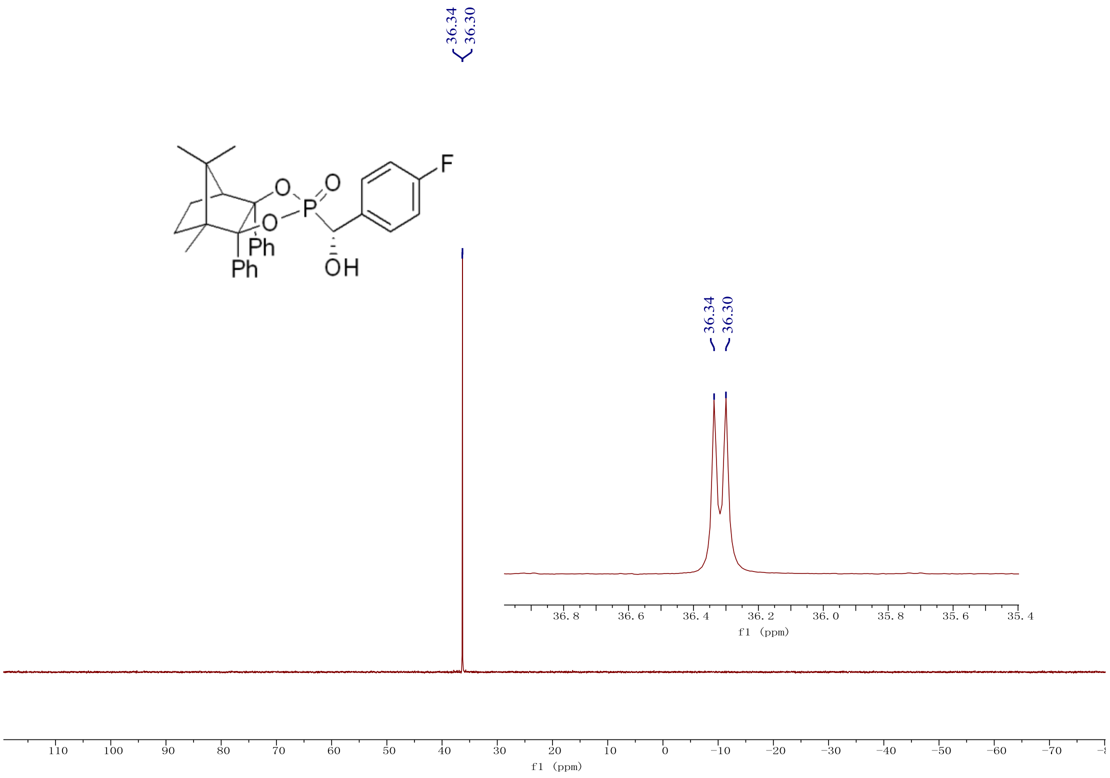

Fig. S18 ^{31}P NMR of compound **3d**

^{1}H NMR (300 MHz, $CDCl_3$)

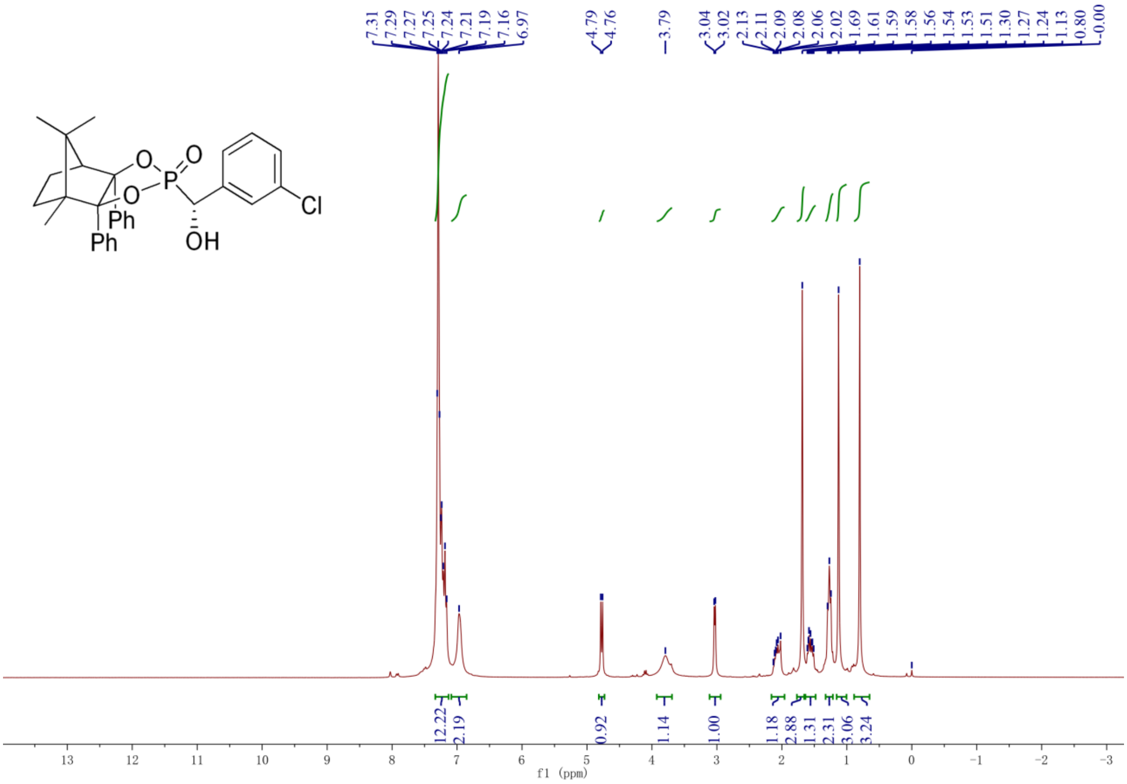

Fig. S19 ^{1}H NMR of compound **3e**

^{13}C NMR (75 MHz, $CDCl_3$)

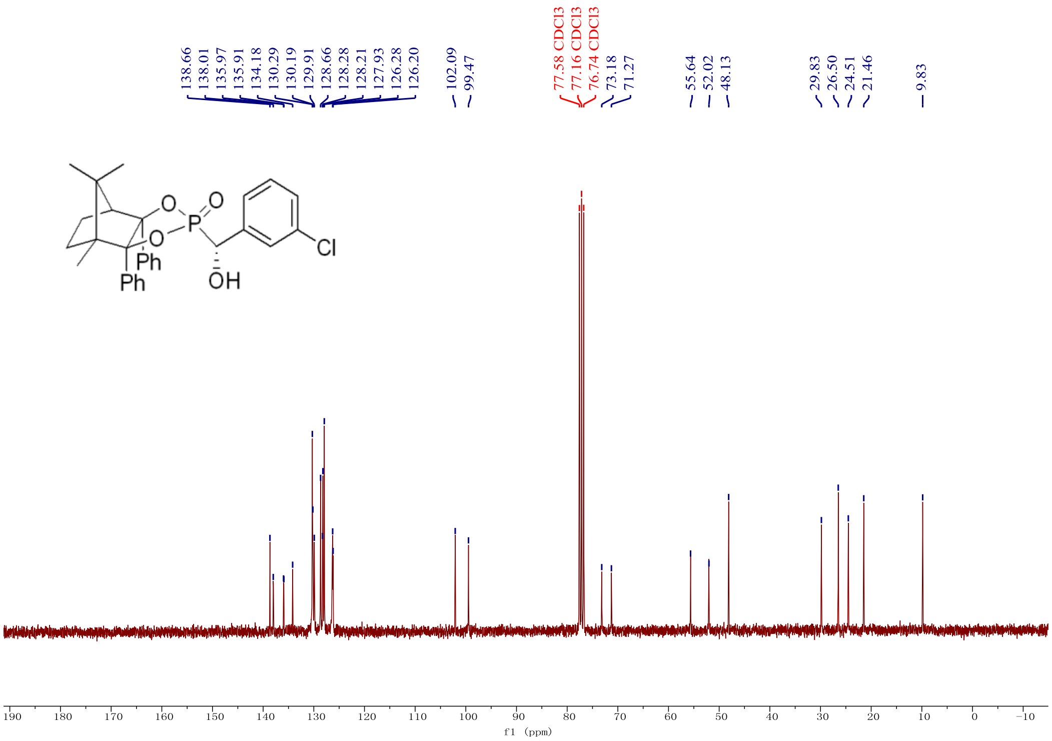

Fig. S20 ^{13}C NMR of compound **3e**

^{31}P NMR (121 MHz, $CDCl_3$)

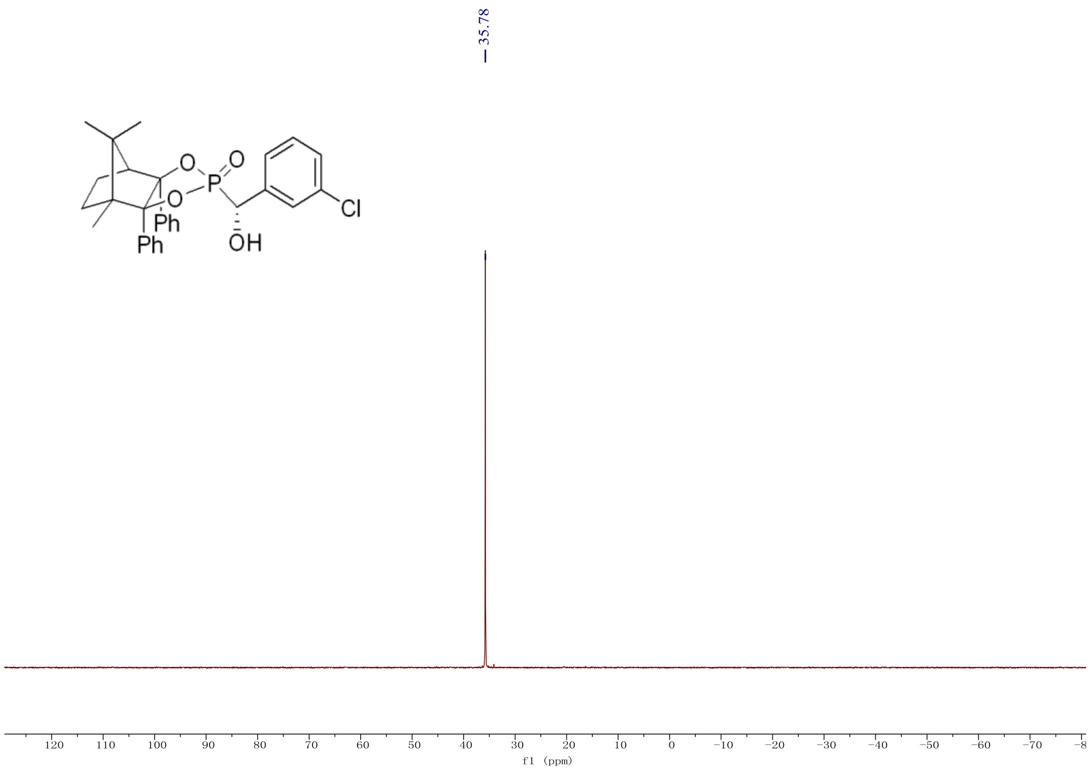

Fig. S21 ^{31}P NMR of compound **3e**

^{1}H NMR (300 MHz, $CDCl_3$)

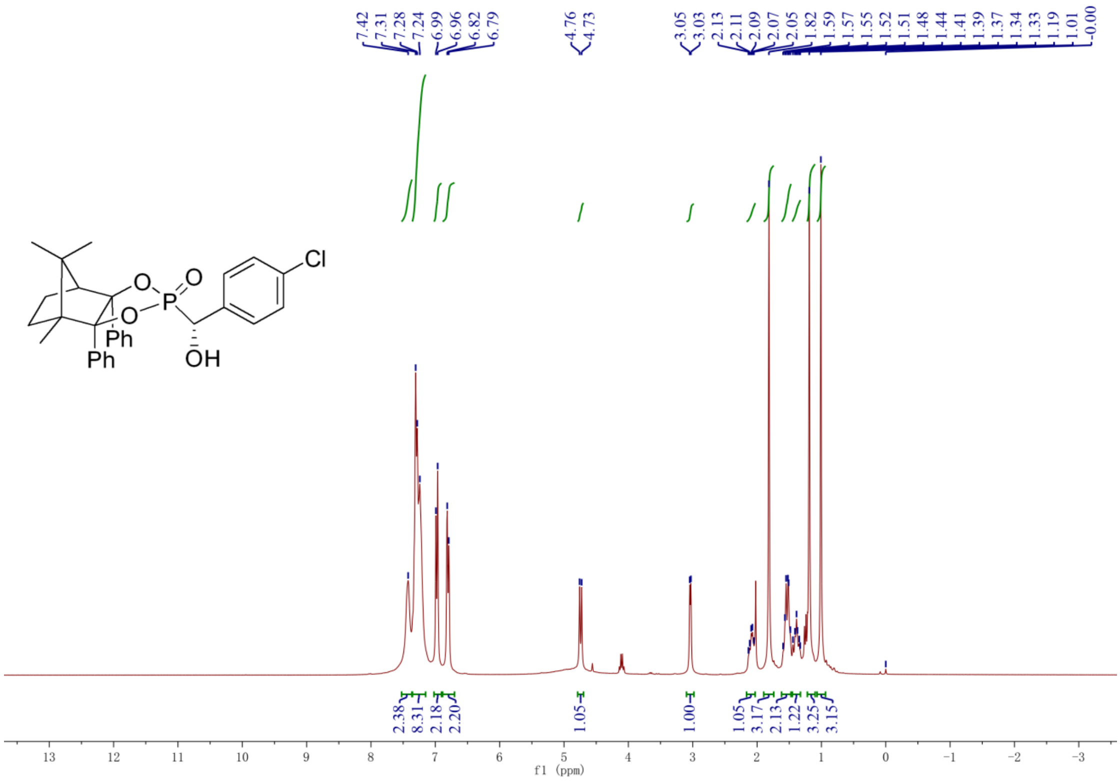

Fig. S22 ^{1}H NMR of compound **3f**

^{13}C NMR (75 MHz, $CDCl_3$)

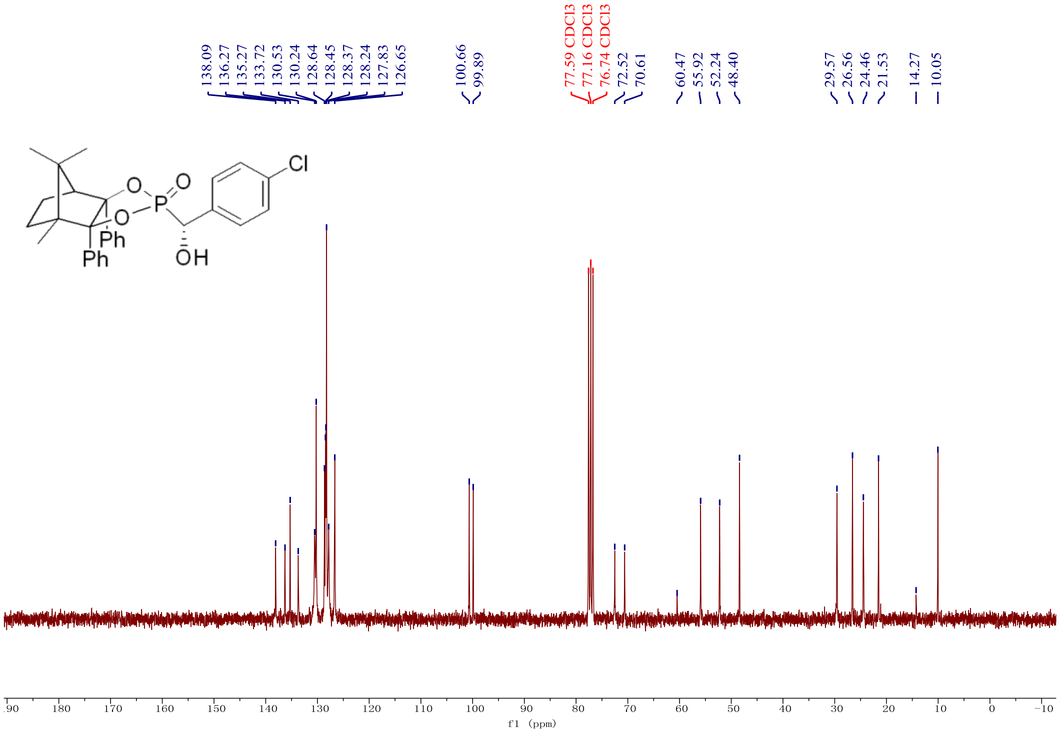

Fig. S23 ^{13}C NMR of compound **3f**

^{31}P NMR (121 MHz, $CDCl_3$)

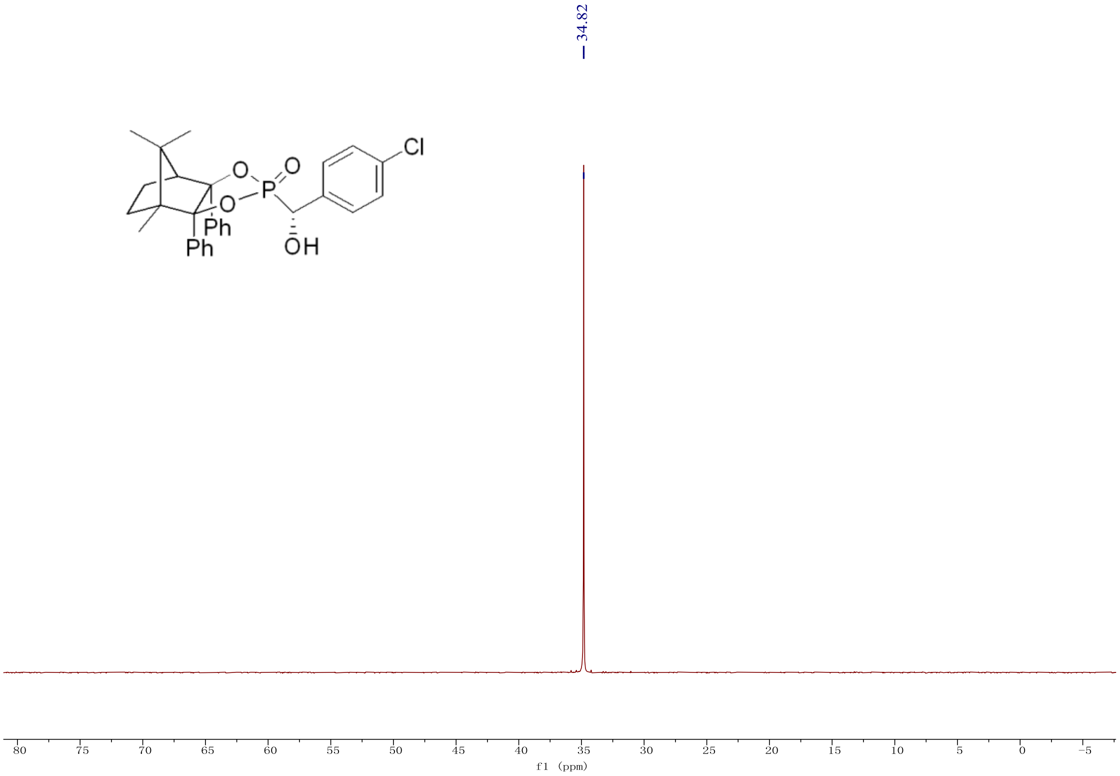

Fig. S24 ^{31}P NMR of compound **3f**

^{1}H NMR (300 MHz, $CDCl_3$)

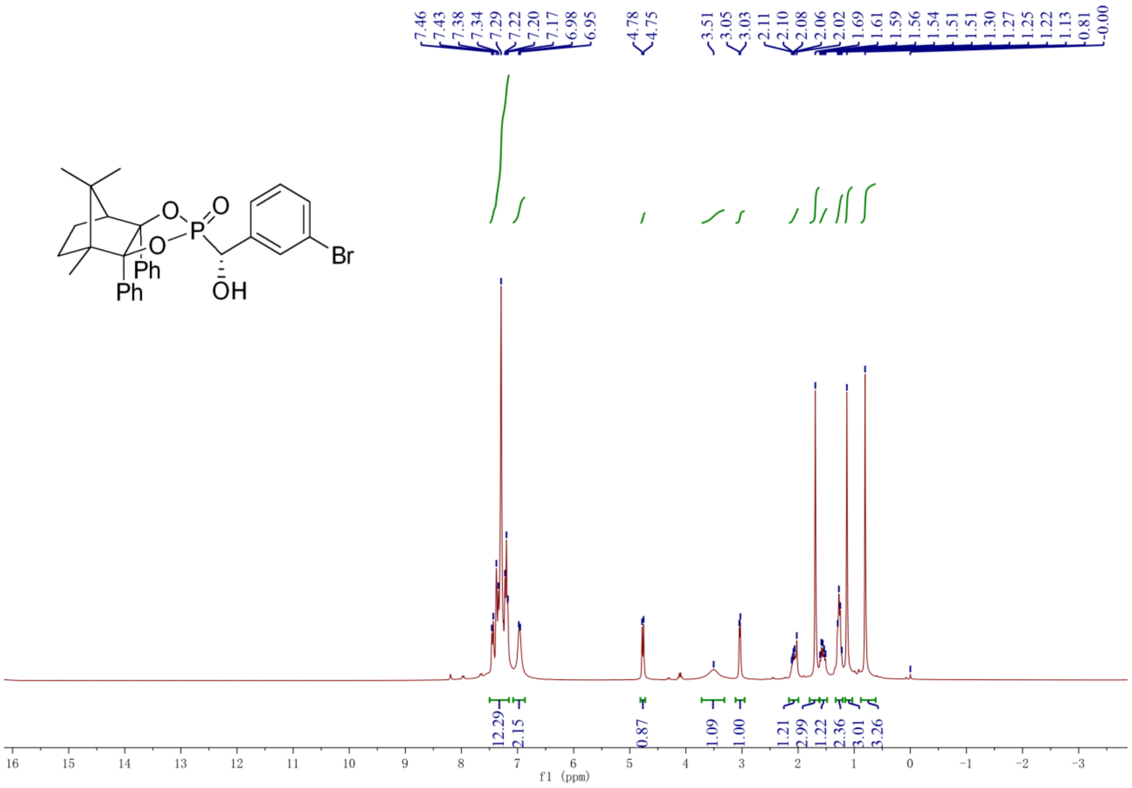

Fig. S25 ^{1}H NMR of compound **3g**

^{13}C NMR (75 MHz, $CDCl_3$)

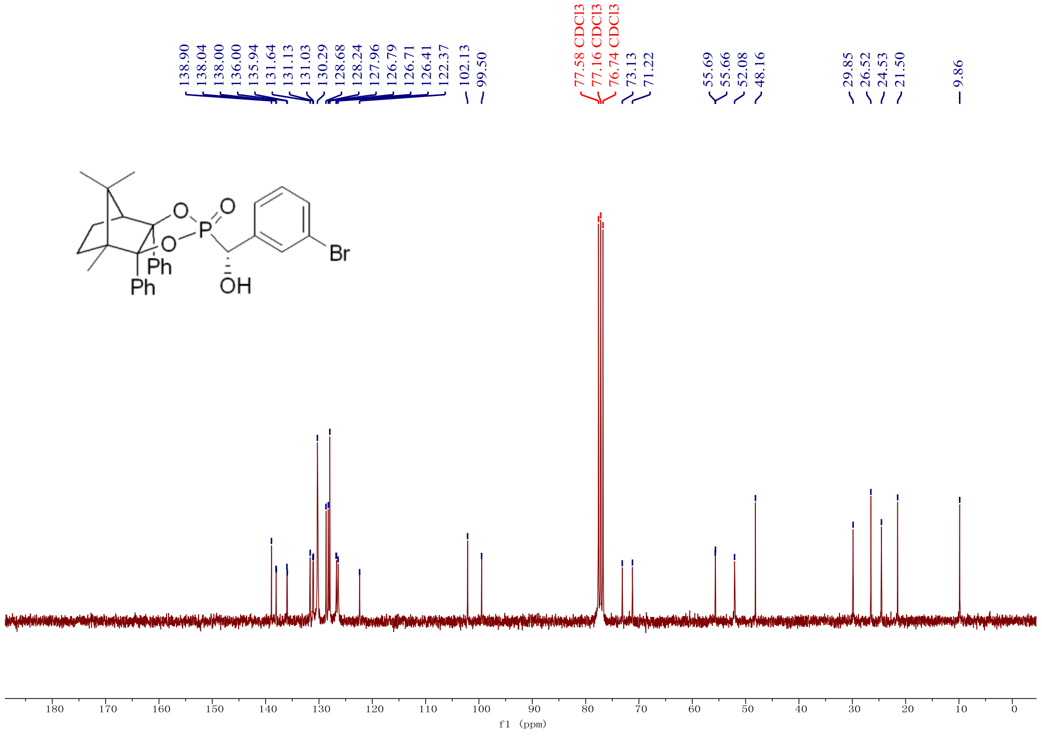

Fig. S26 ^{13}C NMR of compound **3g**

^{31}P NMR (121 MHz, $CDCl_3$)

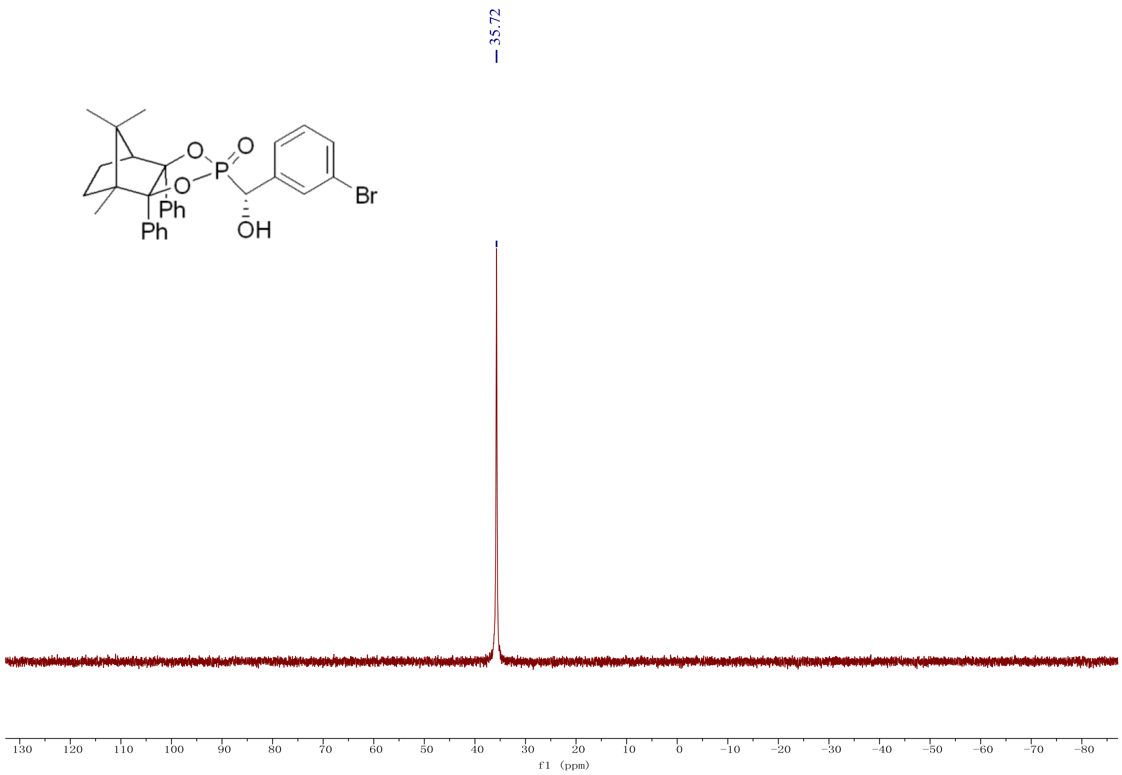

Fig. S27 ^{31}P NMR of compound **3g**

^{1}H NMR (300 MHz, $CDCl_3$)

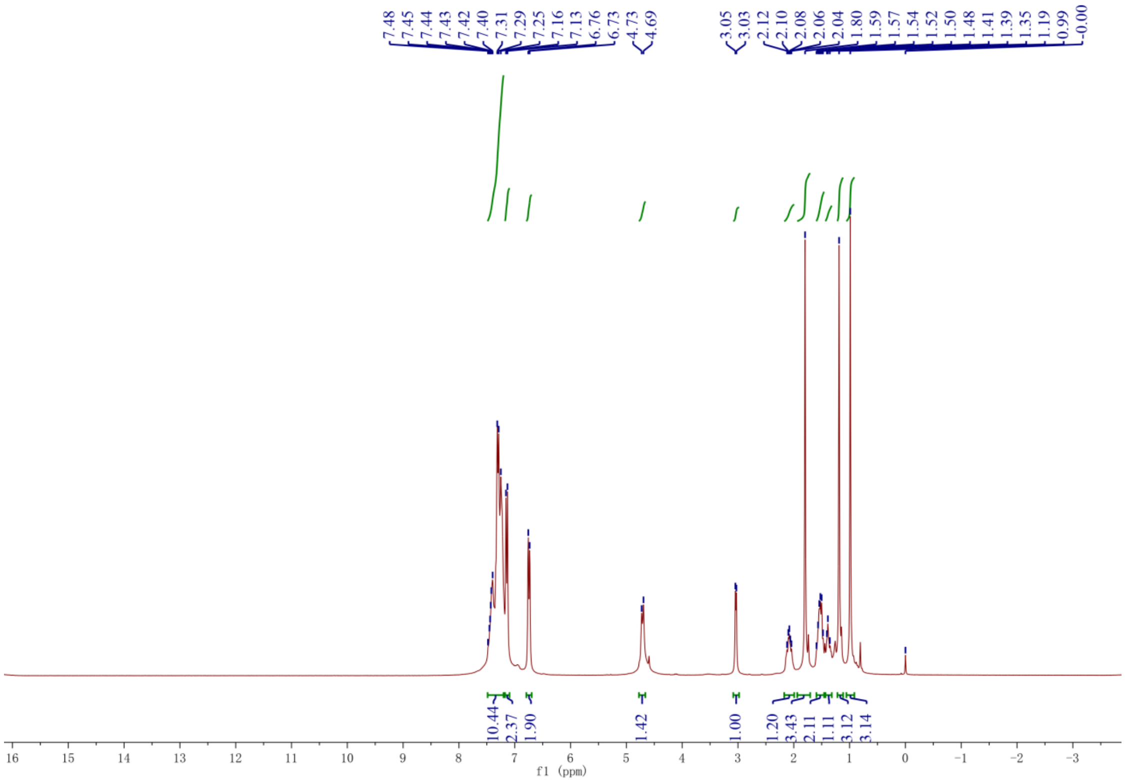

Fig. S28 ^{1}H NMR of compound **3h**

^{13}C NMR (75 MHz, $CDCl_3$)

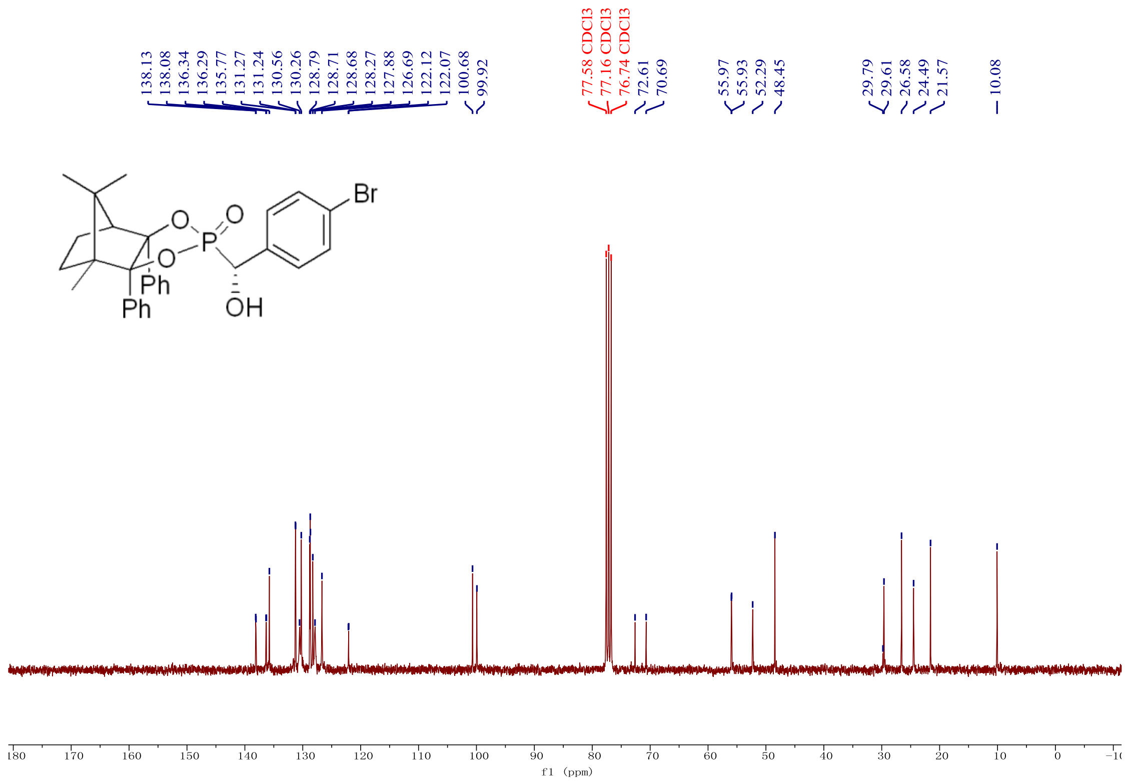

Fig. S29 ^{13}C NMR of compound **3h**

^{31}P NMR (121 MHz, $CDCl_3$)

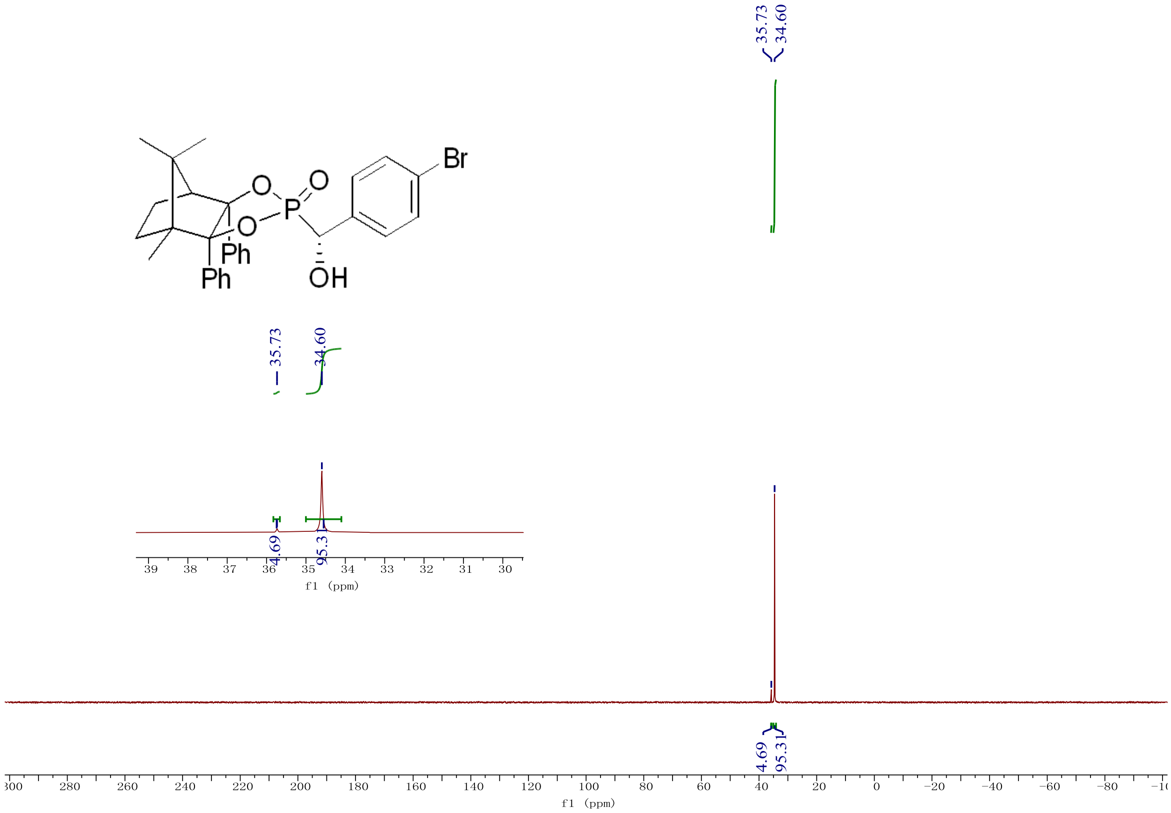

Fig. S30 ^{31}P NMR of compound **3h**

^{1}H NMR (300 MHz, $CDCl_3$)

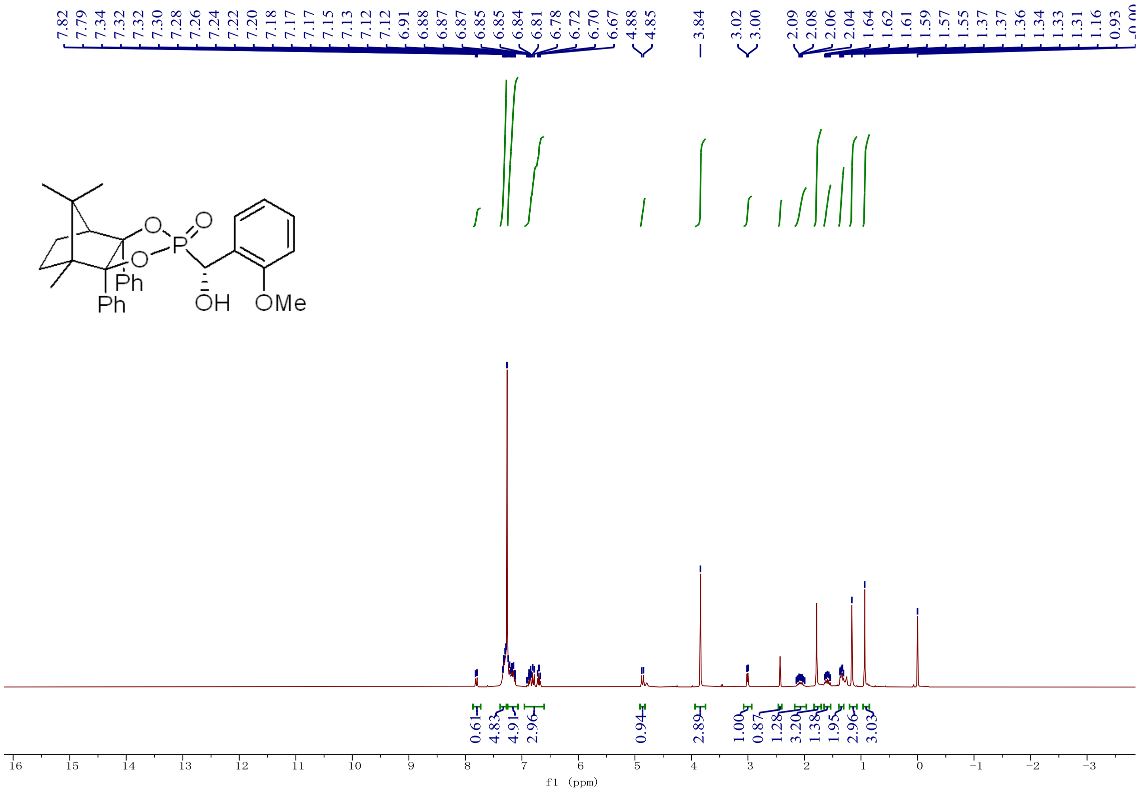

Fig. S31 ^{1}H NMR of compound **3i**

^{13}C NMR (75 MHz, $CDCl_3$)

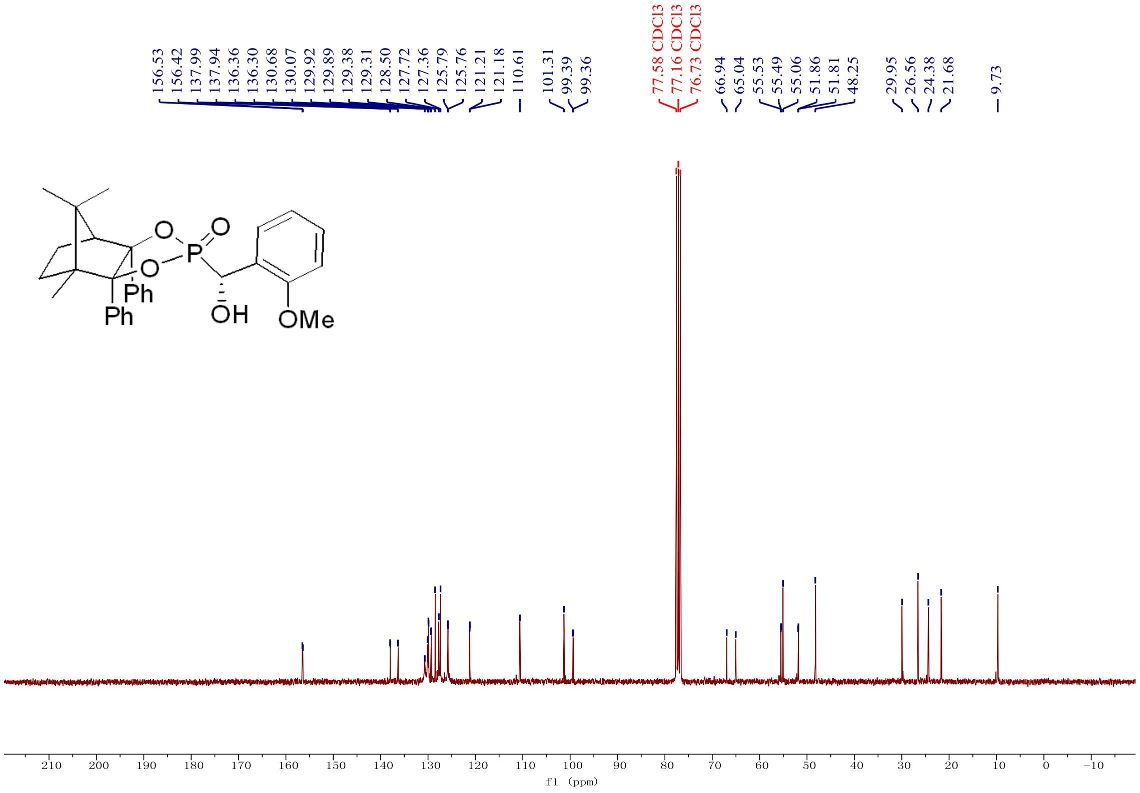

Fig. S32 ^{13}C NMR of compound **3i**

^{31}P NMR (121 MHz, $CDCl_3$)

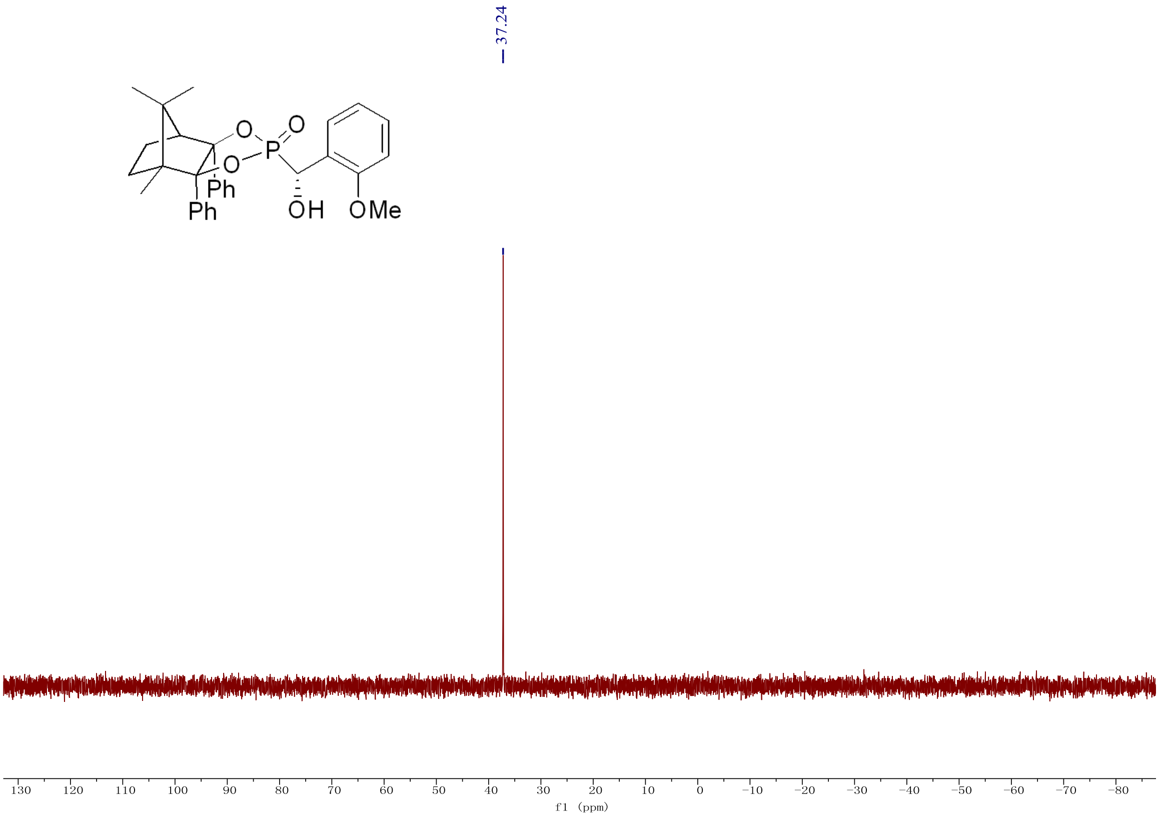

Fig. S33 ^{31}P NMR of compound **3i**

^{1}H NMR (300 MHz, $CDCl_3$)

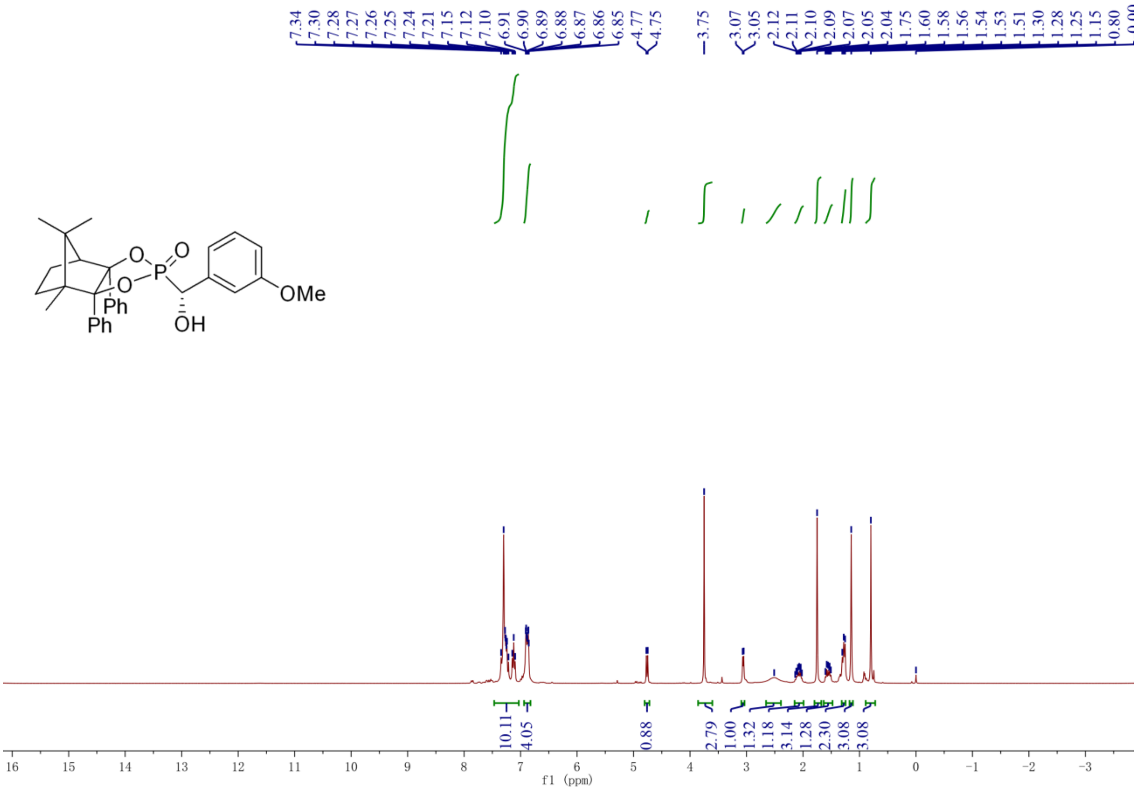

Fig. S34 ^{1}H NMR of compound **3j**

^{13}C NMR (75 MHz, $CDCl_3$)

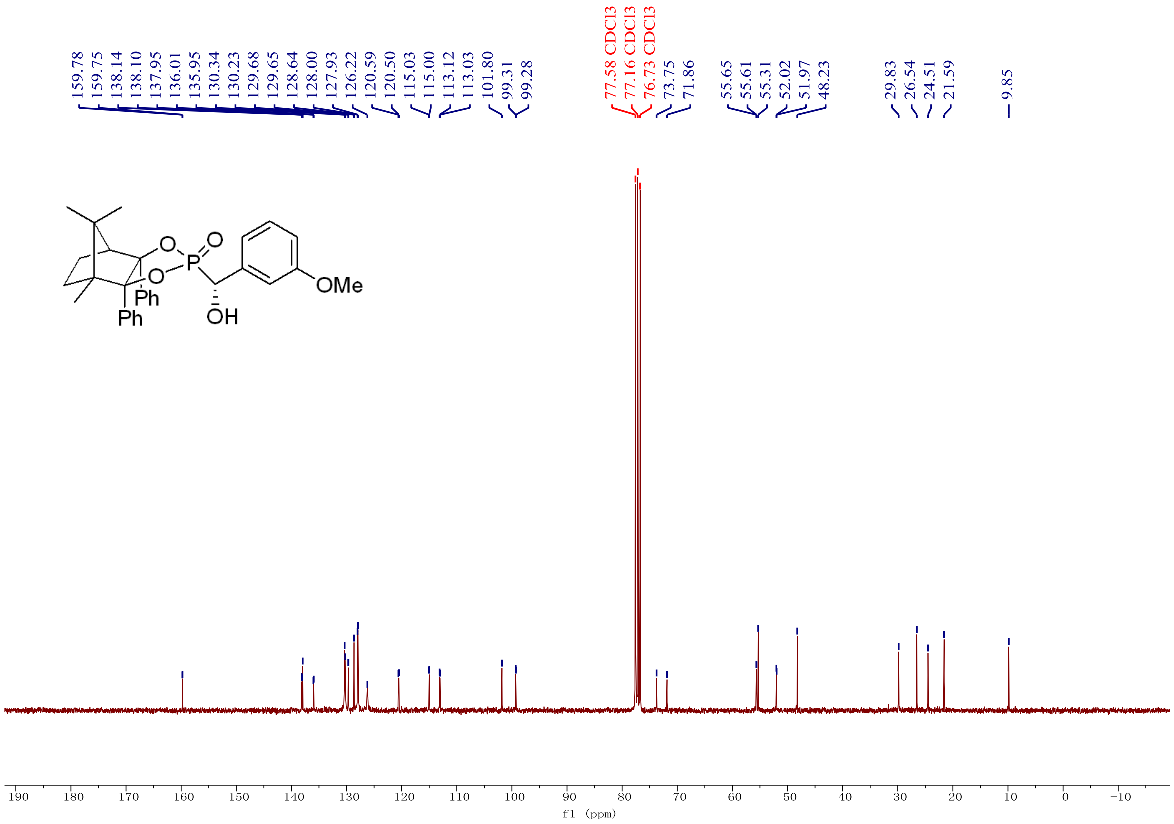

Fig. S35 ^{13}C NMR of compound **3j**

^{31}P NMR (121 MHz, $CDCl_3$)

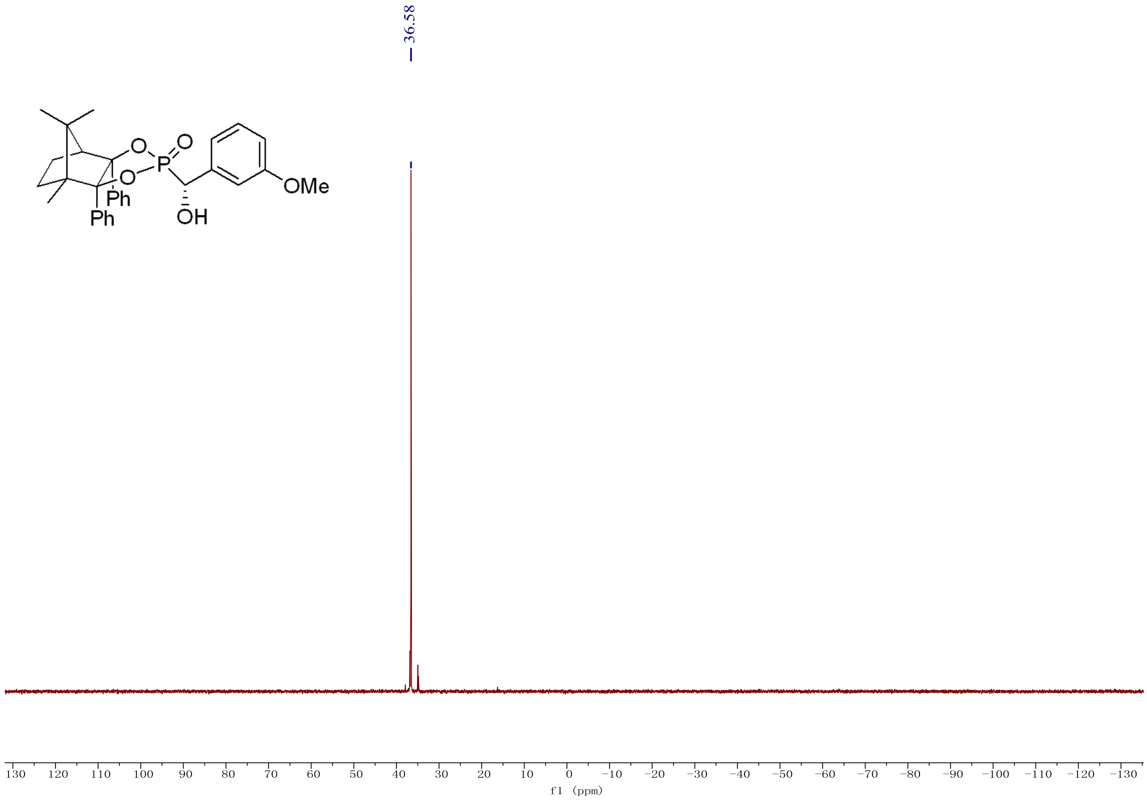

Fig. S36 ^{31}P NMR of compound **3j**

^{1}H NMR (300 MHz, $CDCl_3$)

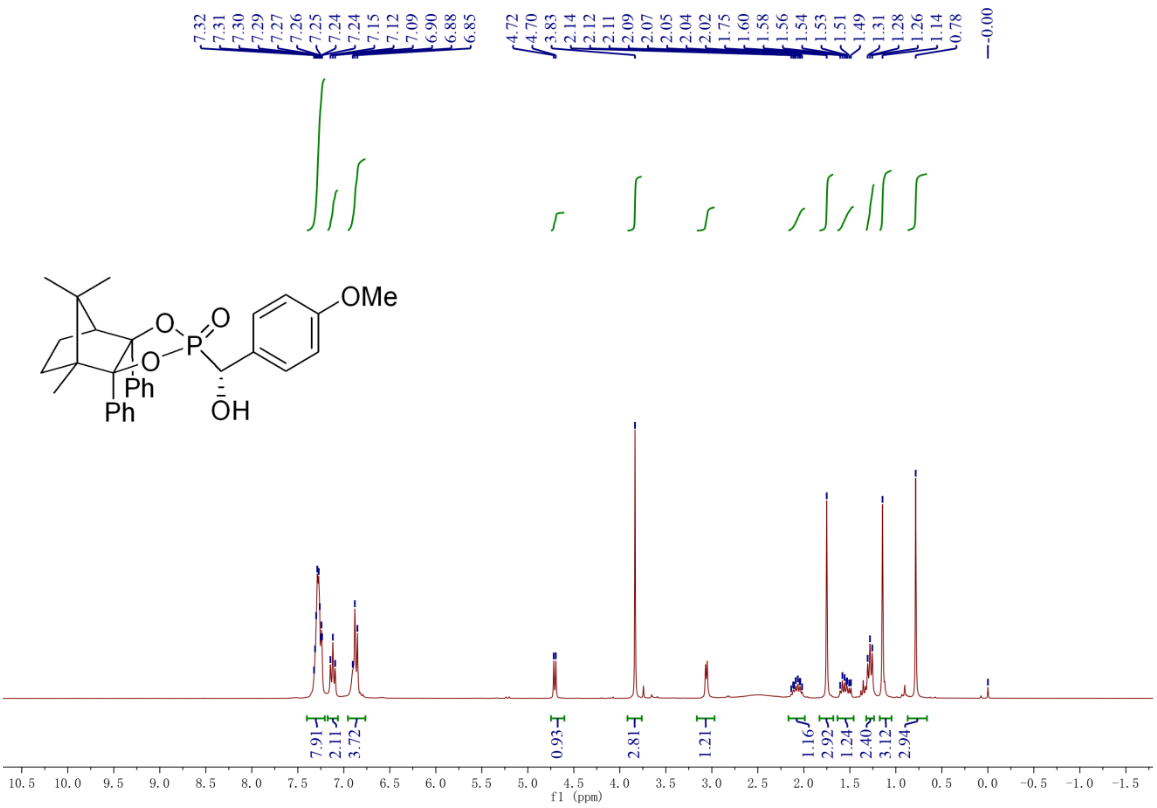

Fig. S37 ^{1}H NMR of compound **3k**

^{13}C NMR (75 MHz, $CDCl_3$)

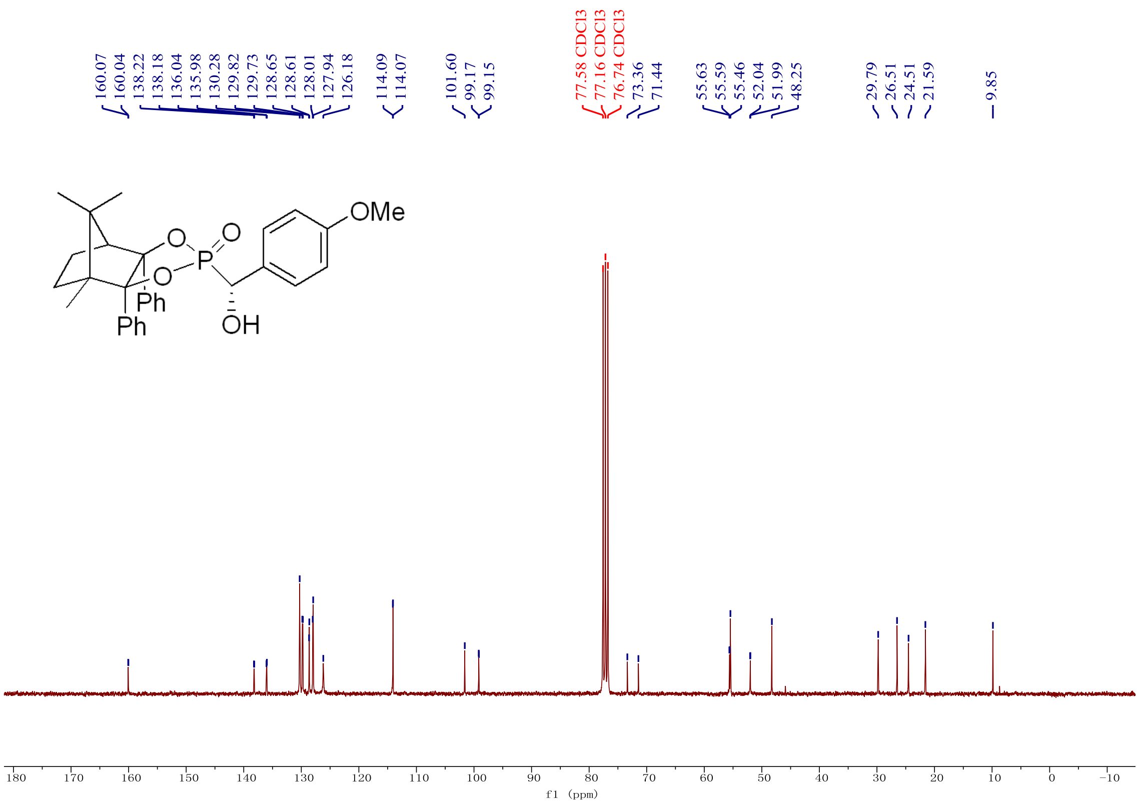

Fig. S38 ^{13}C NMR of compound **3k**

^{31}P NMR (121 MHz, $CDCl_3$)

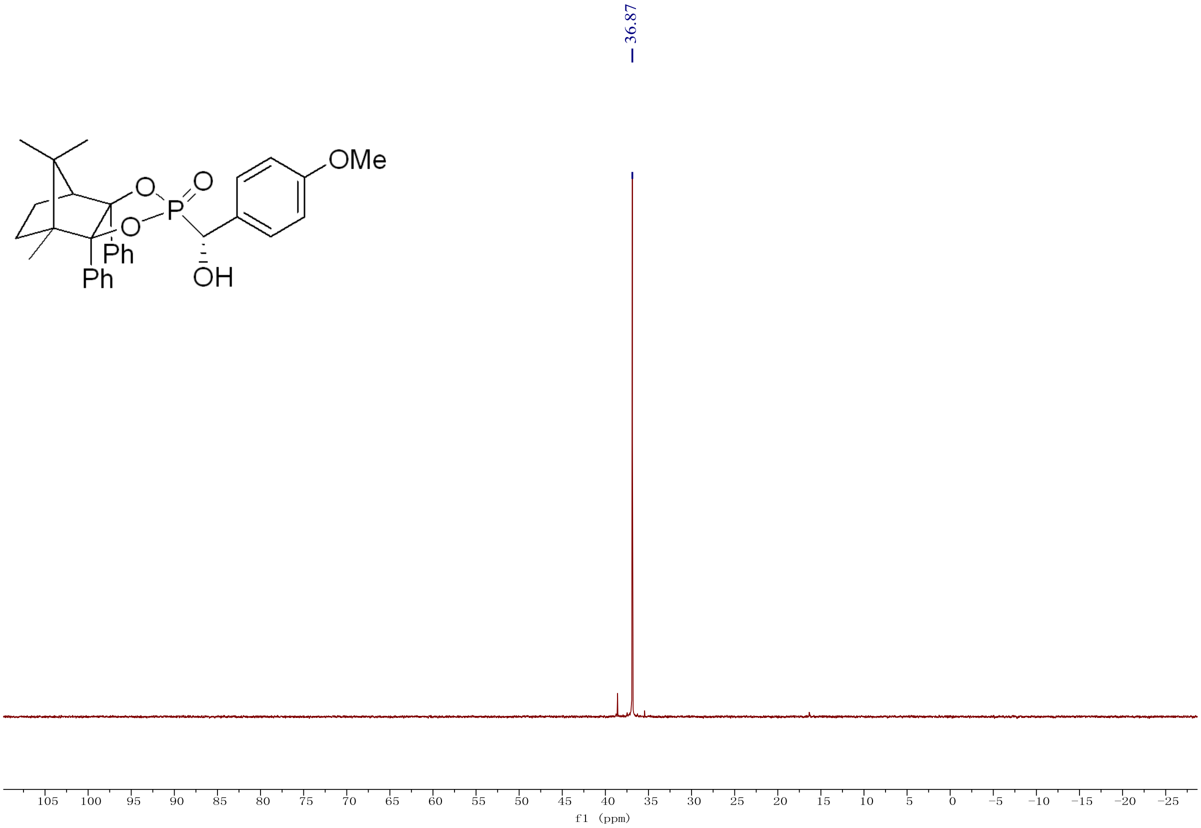

Fig. S39 ^{31}P NMR of compound **3k**

^{1}H NMR (300 MHz, $CDCl_3$)

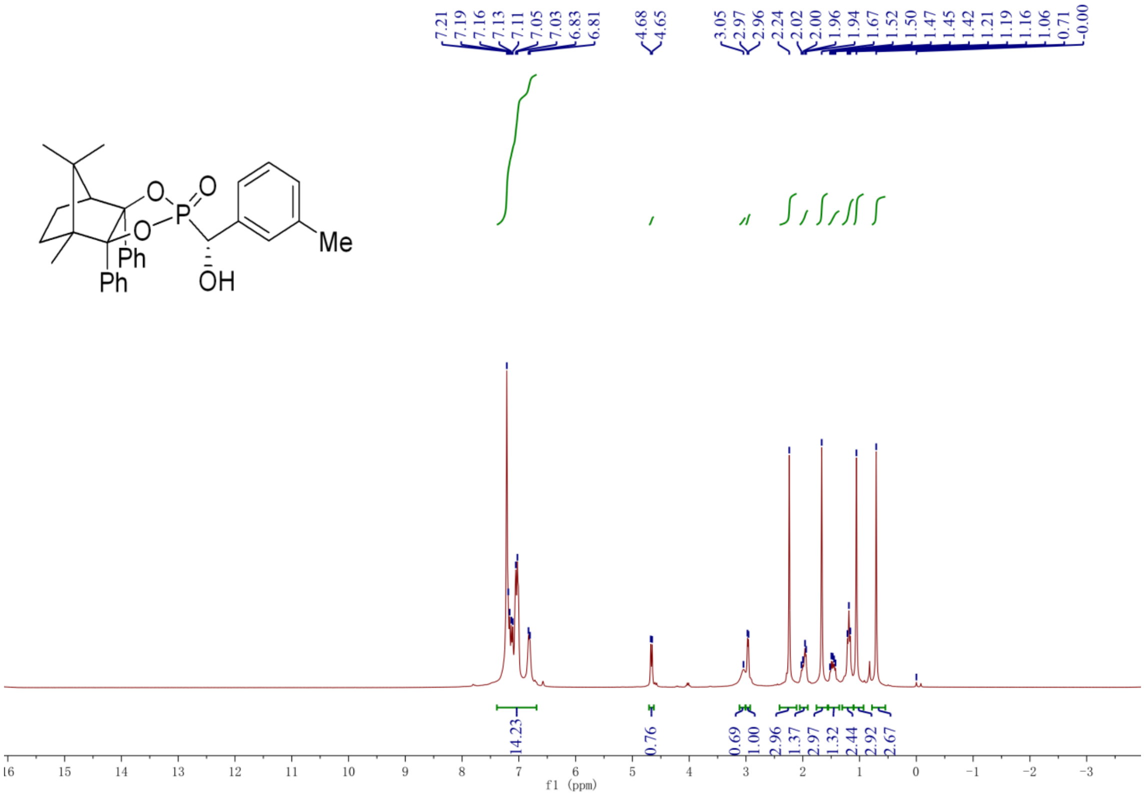

Fig. S40 ^{1}H NMR of compound **3l**

^{13}C NMR (75 MHz, $CDCl_3$)

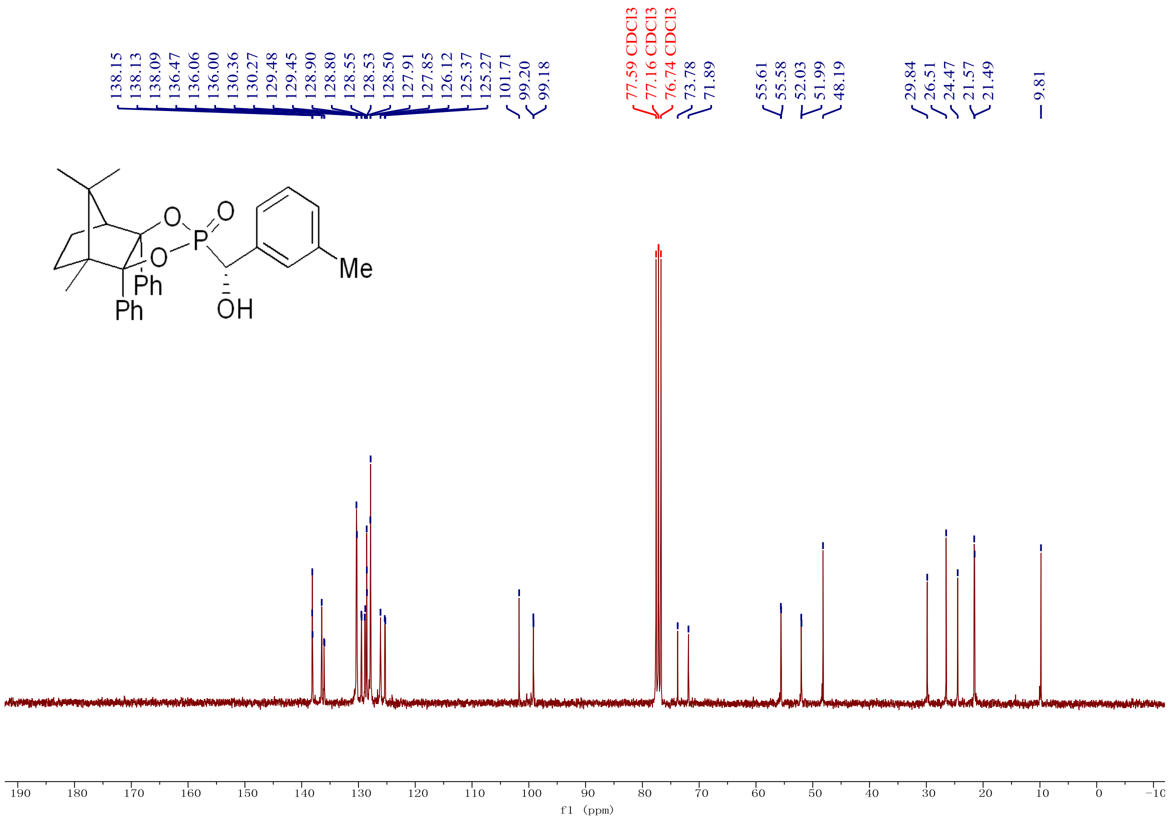

Fig. S41 ^{13}C NMR of compound **3l**

^{31}P NMR (121 MHz, $CDCl_3$)

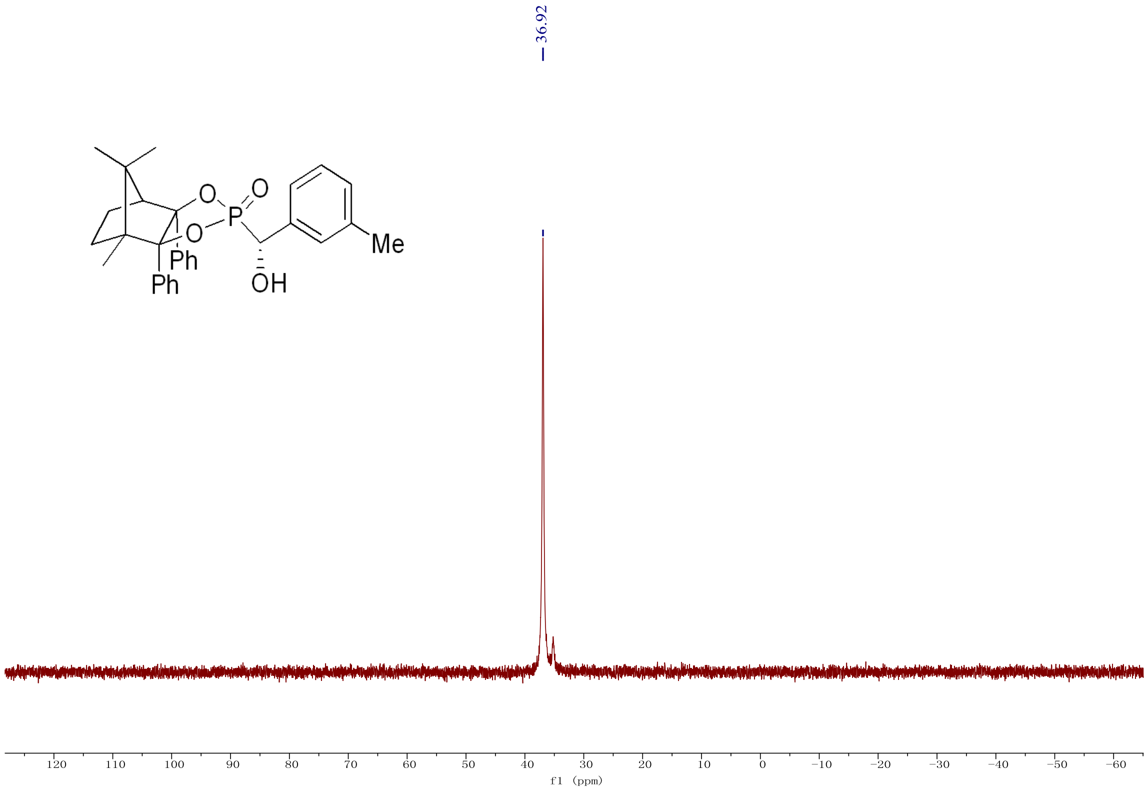

Fig. S42 ^{31}P NMR of compound **3l**

^{1}H NMR (300 MHz, $CDCl_3$)

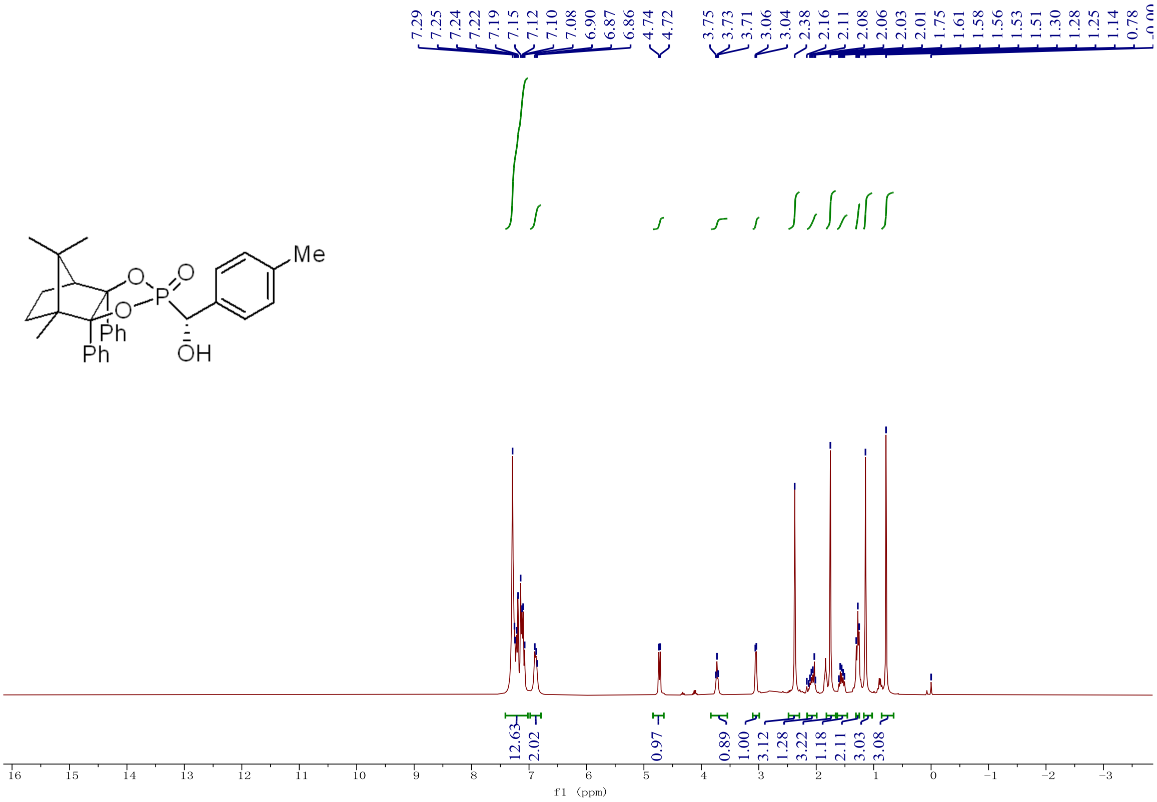

Fig. S43 ^{1}H NMR of compound **3m**

^{13}C NMR (75 MHz, $CDCl_3$)

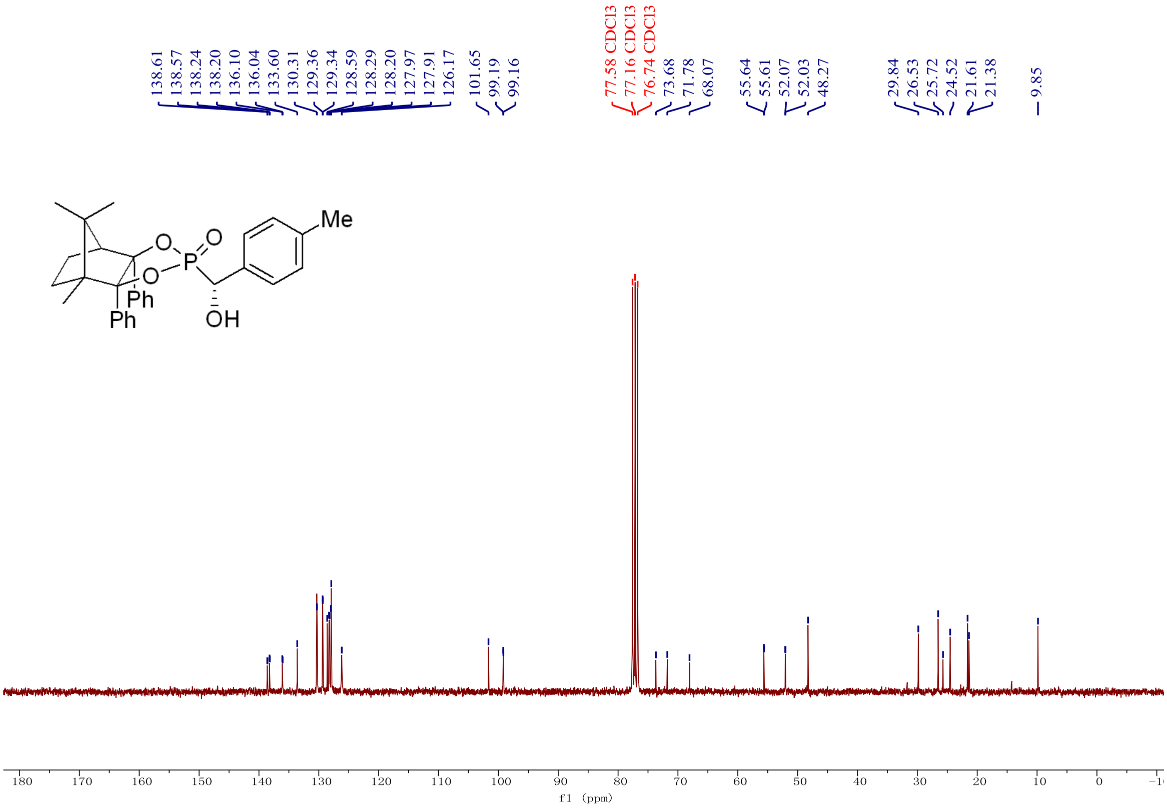

Fig. S44 ^{13}C NMR of compound **3m**

^{31}P NMR (121 MHz, $CDCl_3$)

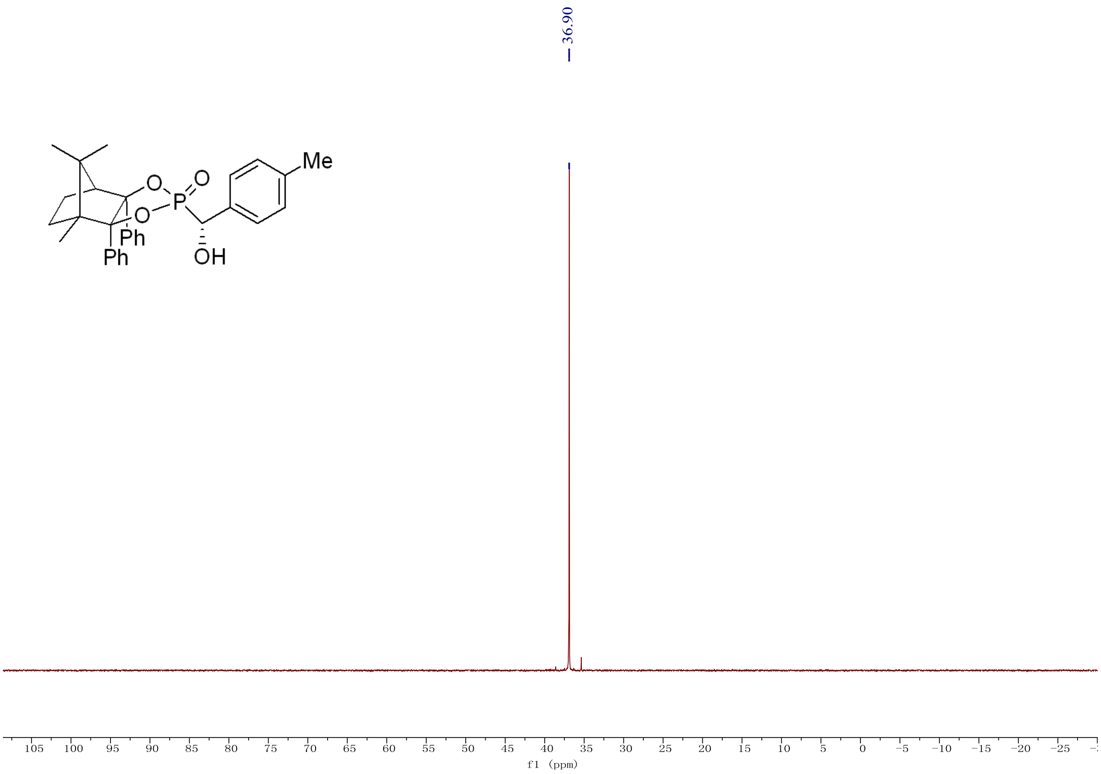

Fig. S45 ^{31}P NMR of compound **3m**

^{1}H NMR (300 MHz, $CDCl_3$)

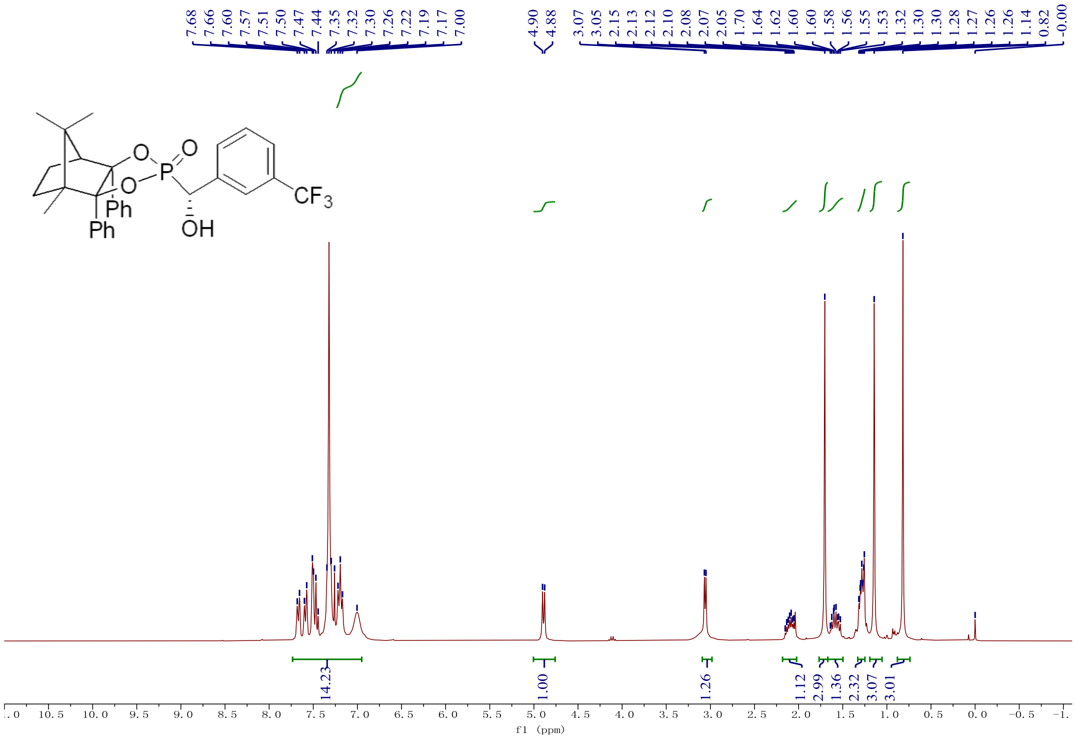

Fig. S46 ^{1}H NMR of compound **3n**

^{13}C NMR (75 MHz, $CDCl_3$)

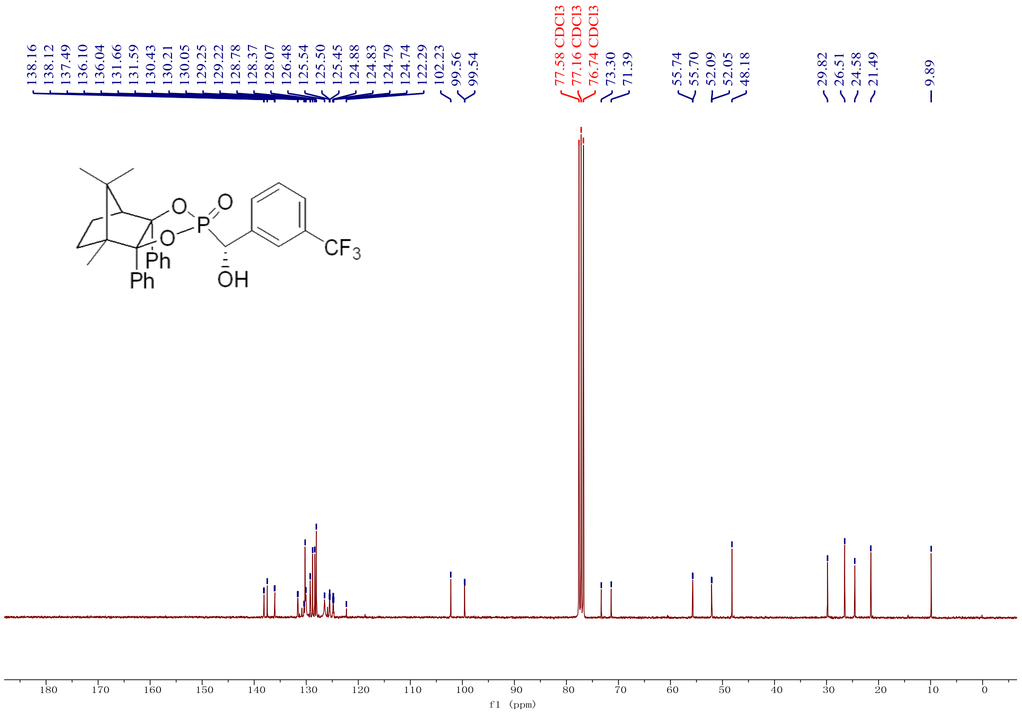

Fig. S47 ^{13}C NMR of compound **3n**

^{19}F NMR (282 MHz, $CDCl_3$)

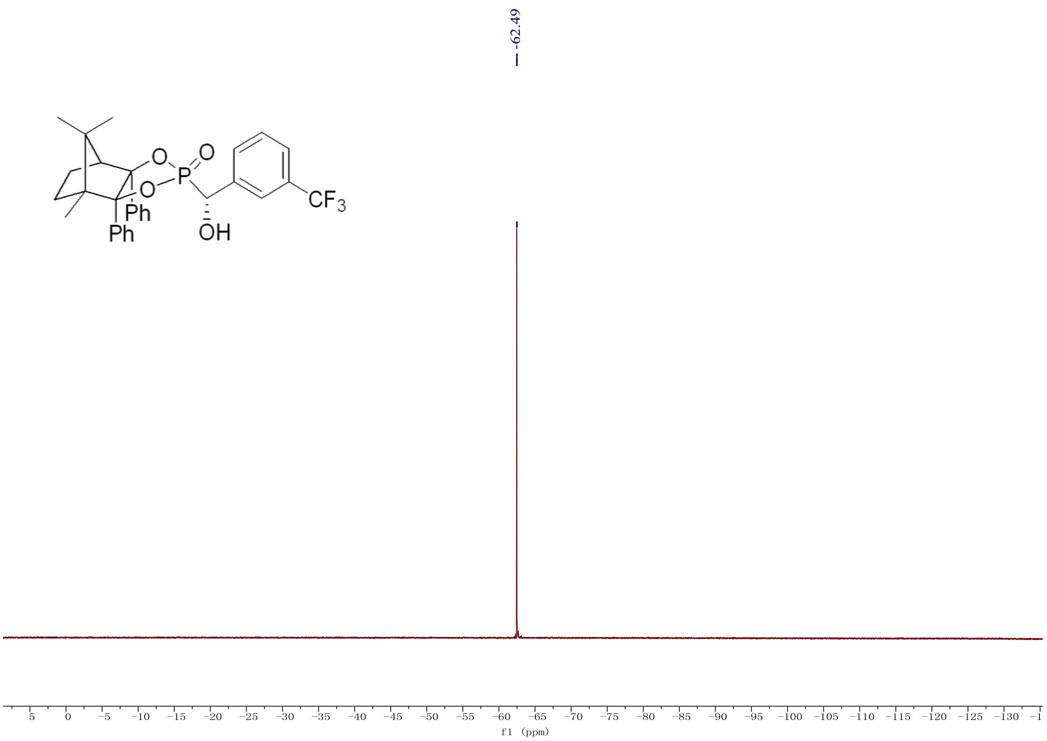

Fig. S48 ^{19}F NMR of compound **3n**

^{31}P NMR (121 MHz, $CDCl_3$)

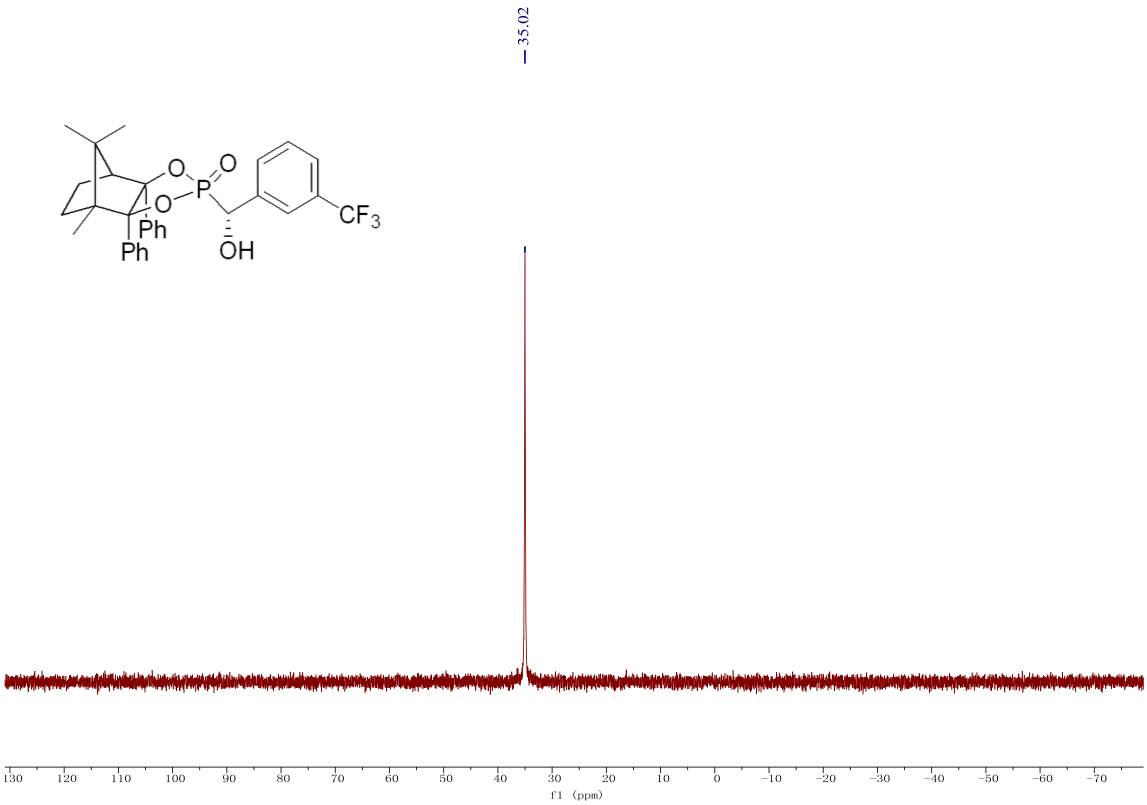

Fig. S49 ^{31}P NMR of compound **3n**

^{1}H NMR (300 MHz, $CDCl_3$)

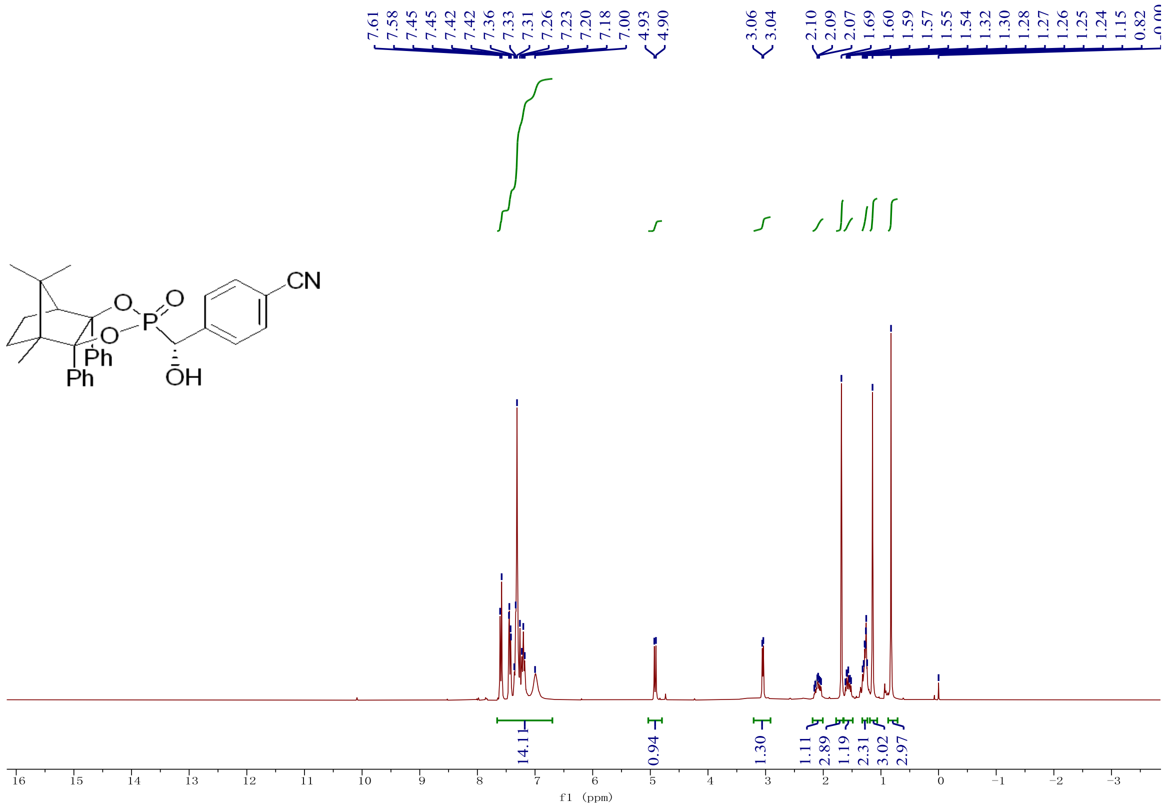

Fig. S50 ^{1}H NMR of compound **3o**

^{13}C NMR (75 MHz, $CDCl_3$)

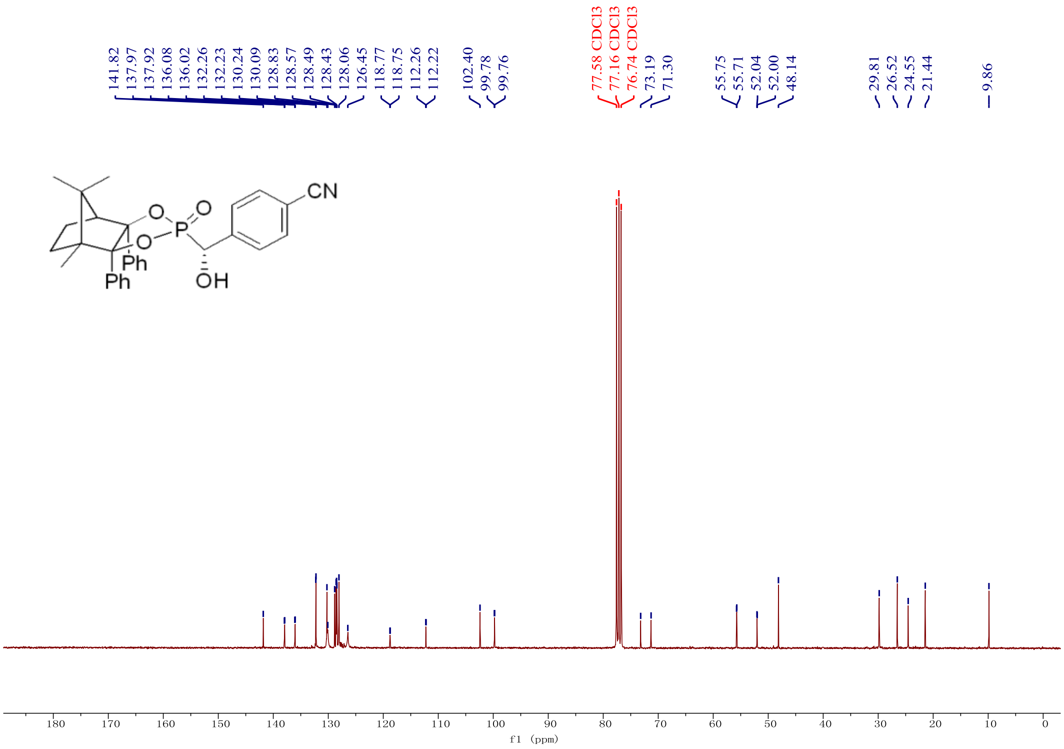

Fig. S51 ^{13}C NMR of compound **3o**

^{31}P NMR (121 MHz, $CDCl_3$)

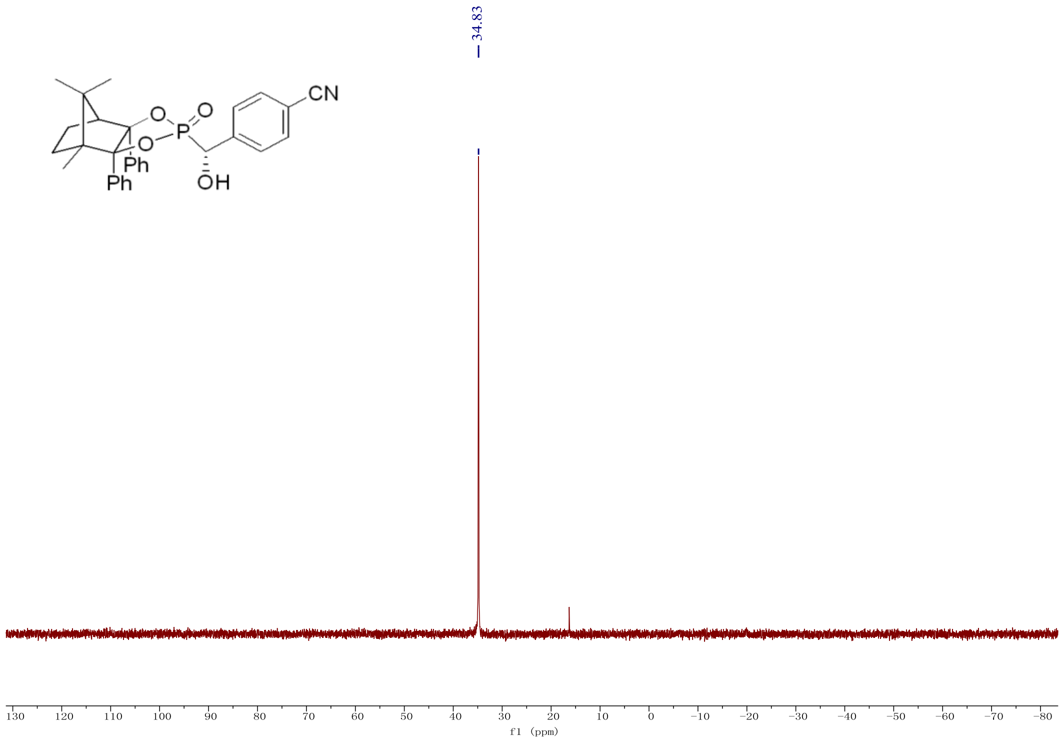

Fig. S52 ^{31}P NMR of compound **3o**

^{1}H NMR (300 MHz, $CDCl_3$)

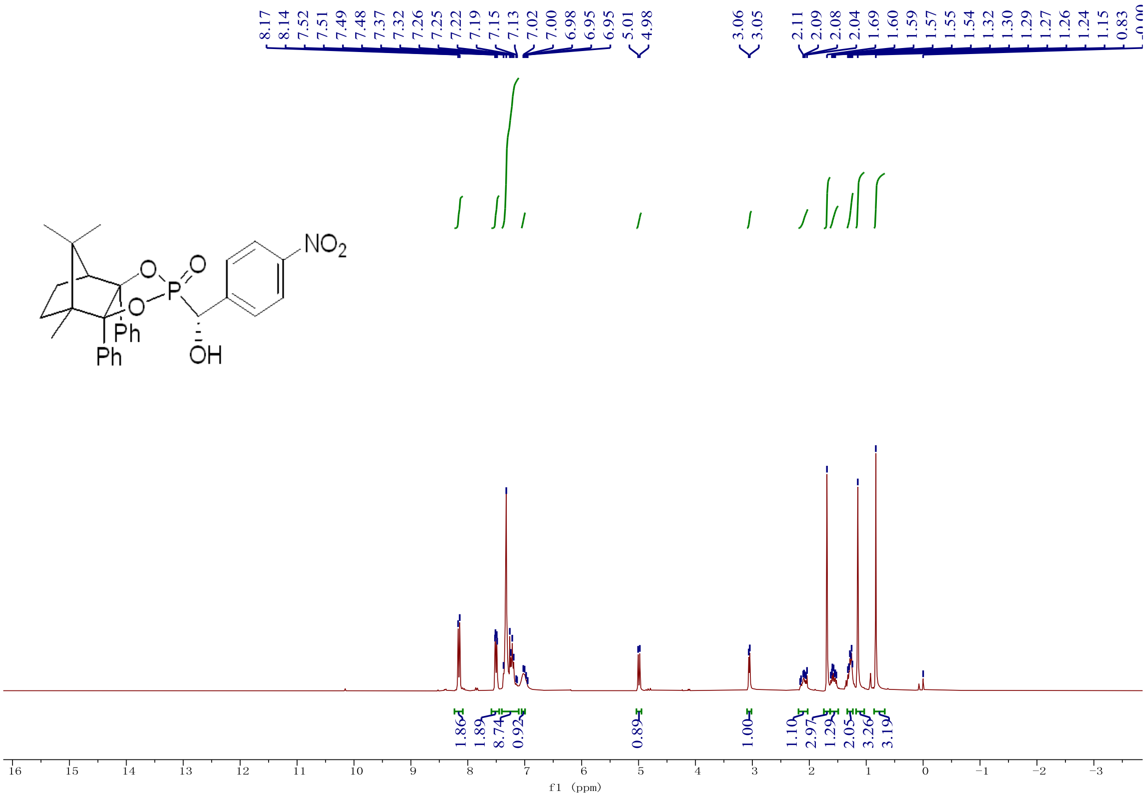

Fig. S53 ^{1}H NMR of compound **3p**

^{13}C NMR (75 MHz, $CDCl_3$)

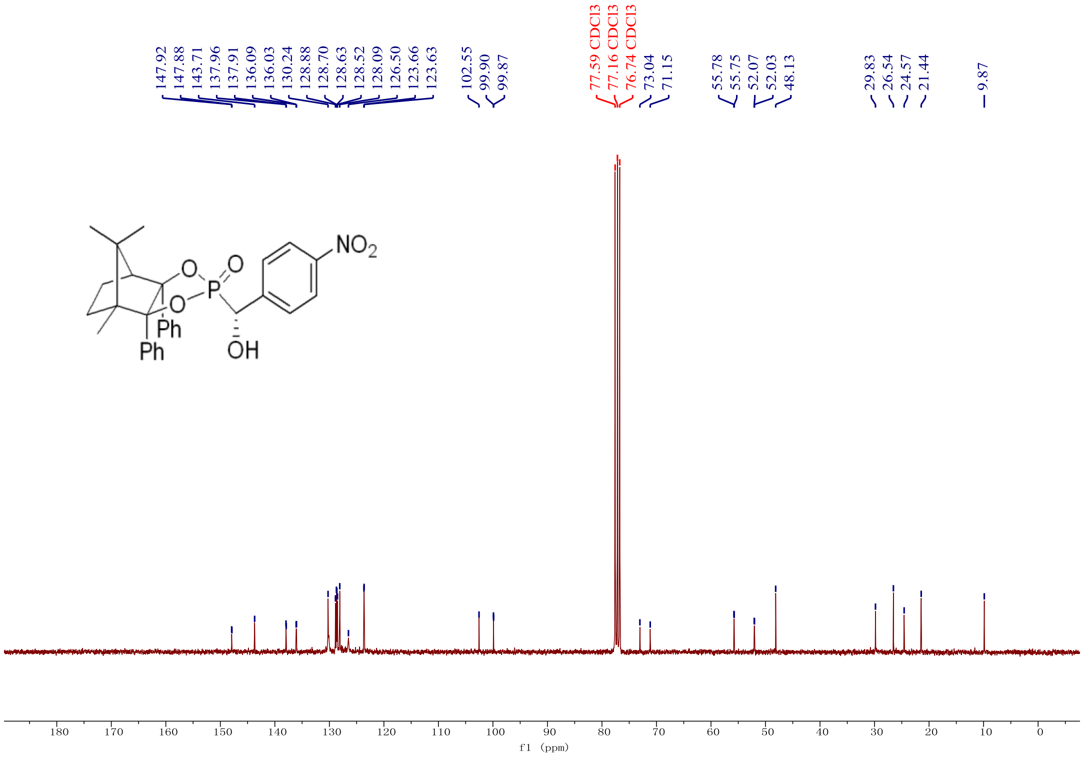

Fig. S54 ^{13}C NMR of compound **3p**

^{31}P NMR (121 MHz, $CDCl_3$)

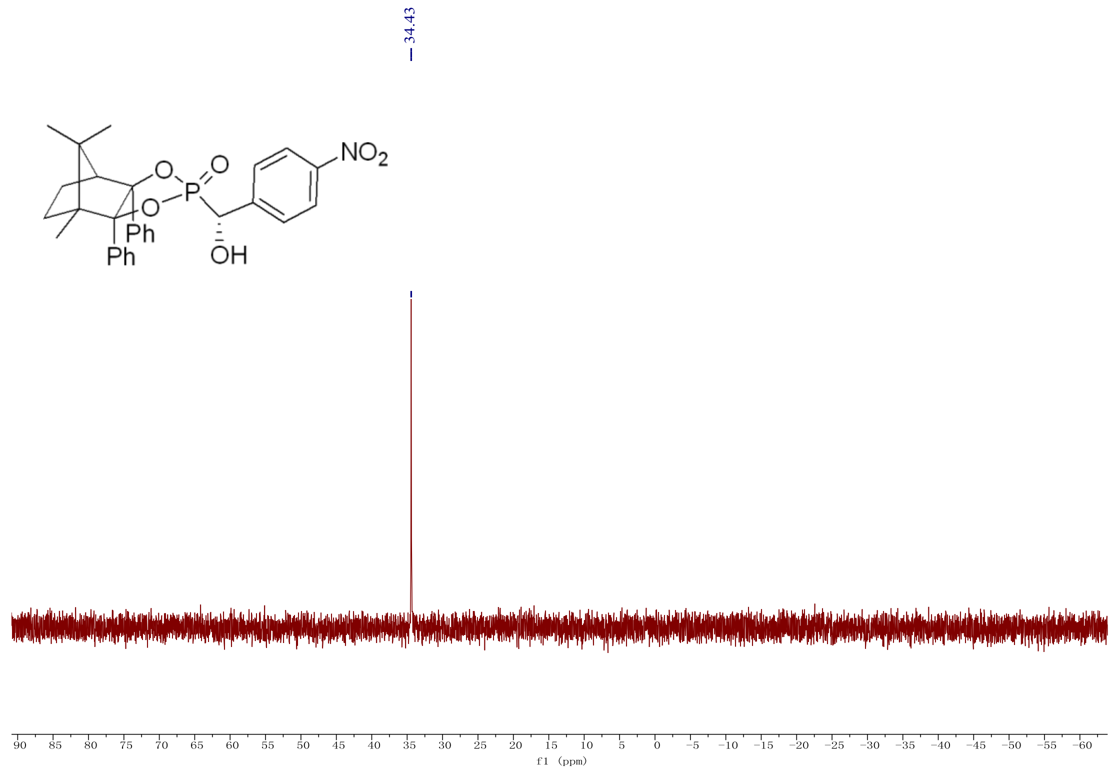

Fig. S55 ^{31}P NMR of compound **3p**

^{1}H NMR (300 MHz, $CDCl_3$)

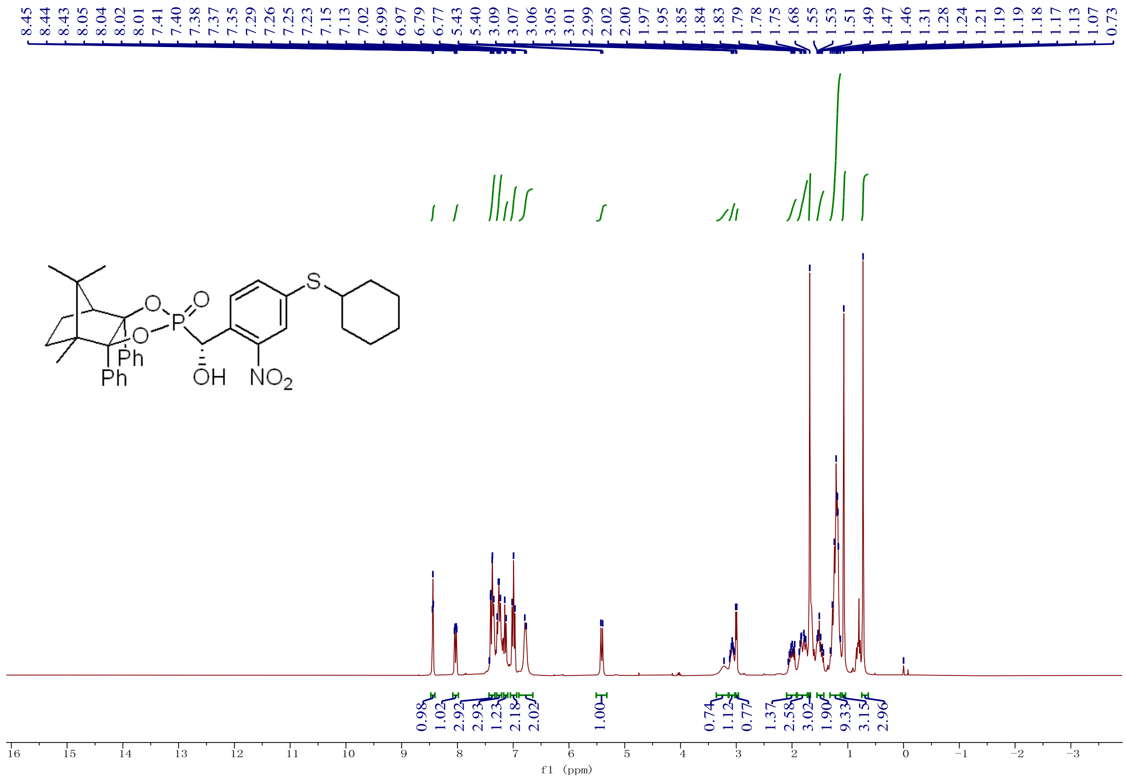

Fig. S56 ^{1}H NMR of compound **3q**

^{13}C NMR (75 MHz, CDCl$_3$)

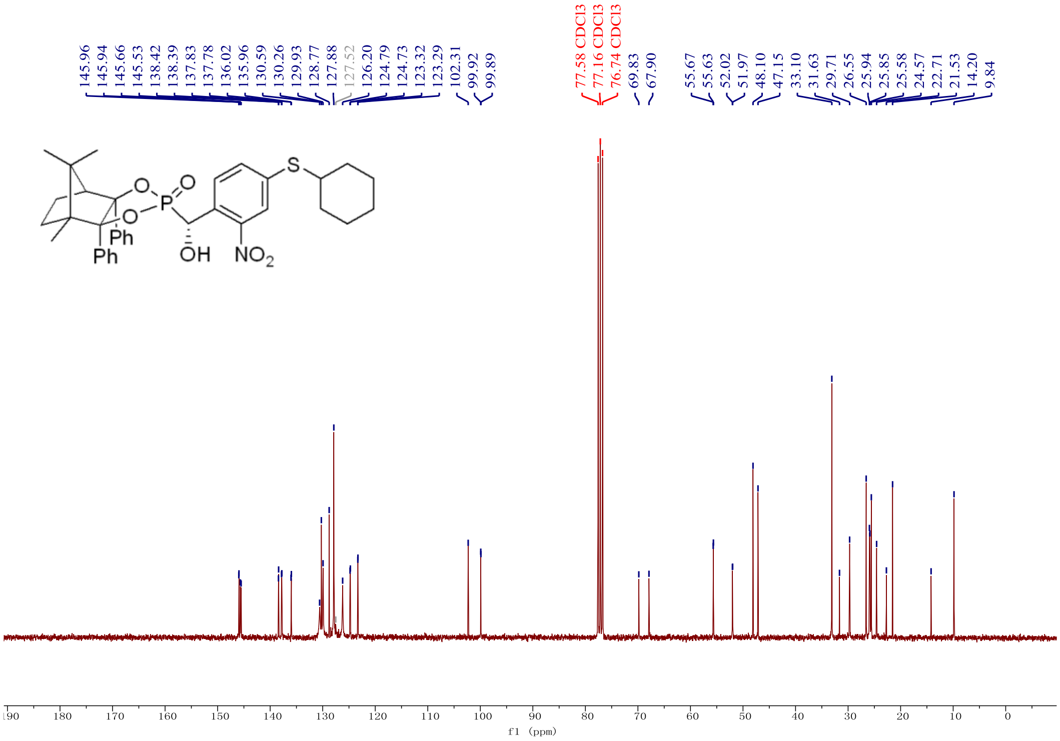

Fig. S57 ^{13}C NMR of compound **3q**

^{31}P NMR (121 MHz, CDCl$_3$)

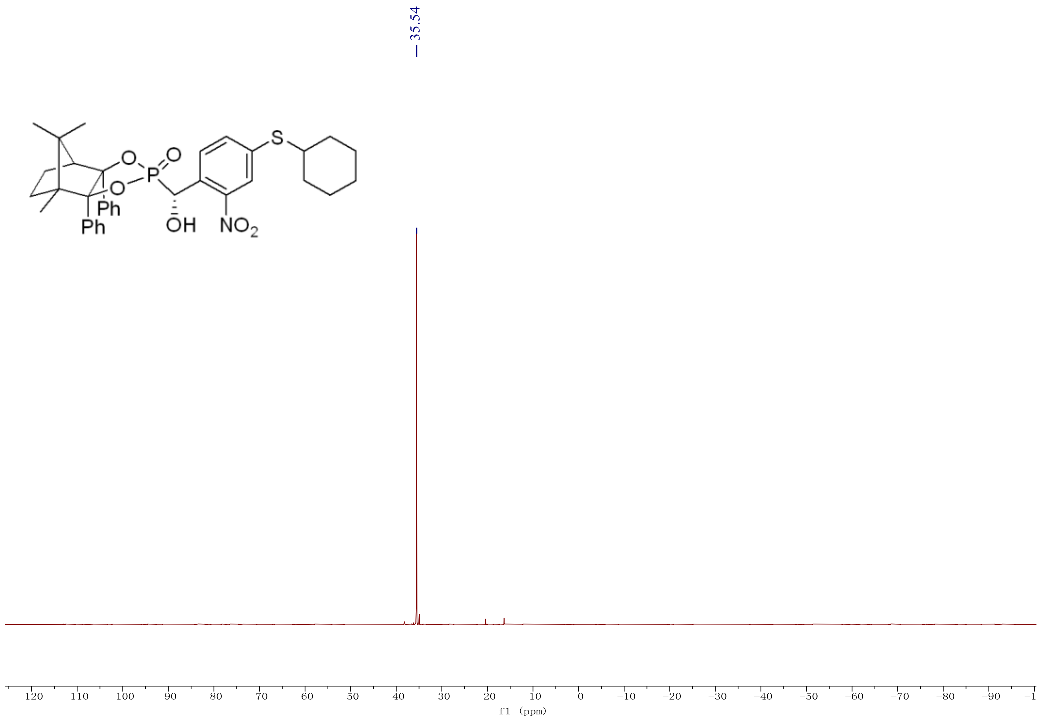

Fig. S58 ^{31}P NMR of compound **3q**

^1H NMR (300 MHz, $CDCl_3$)

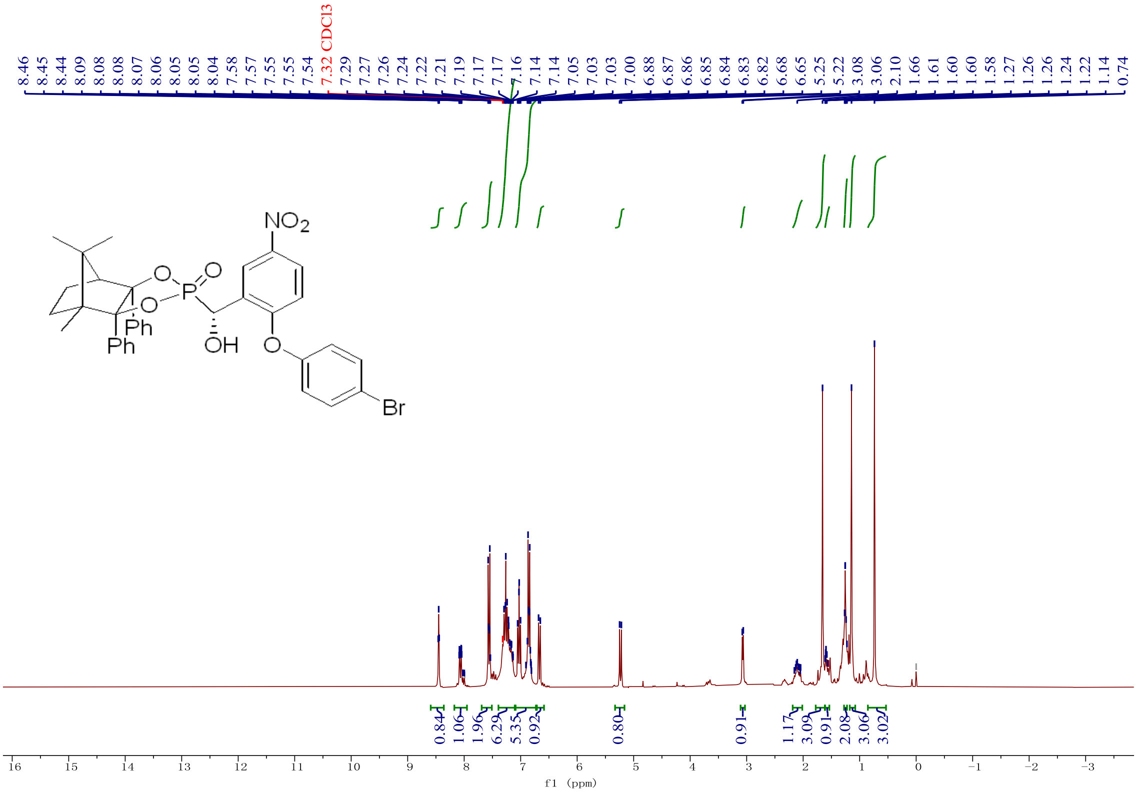

Fig. S59 ^1H NMR of compound **3r**

^{13}C NMR (75 MHz, $CDCl_3$)

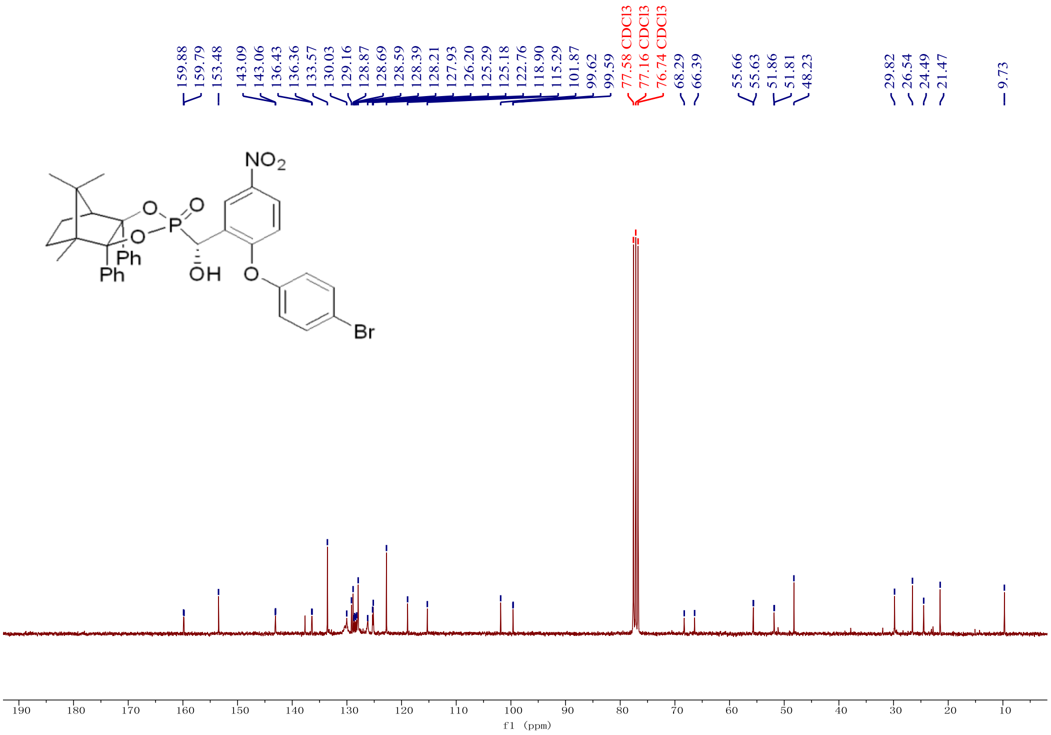

Fig. S60 ^{13}C NMR of compound **3r**

^{31}P NMR (121 MHz, $CDCl_3$)

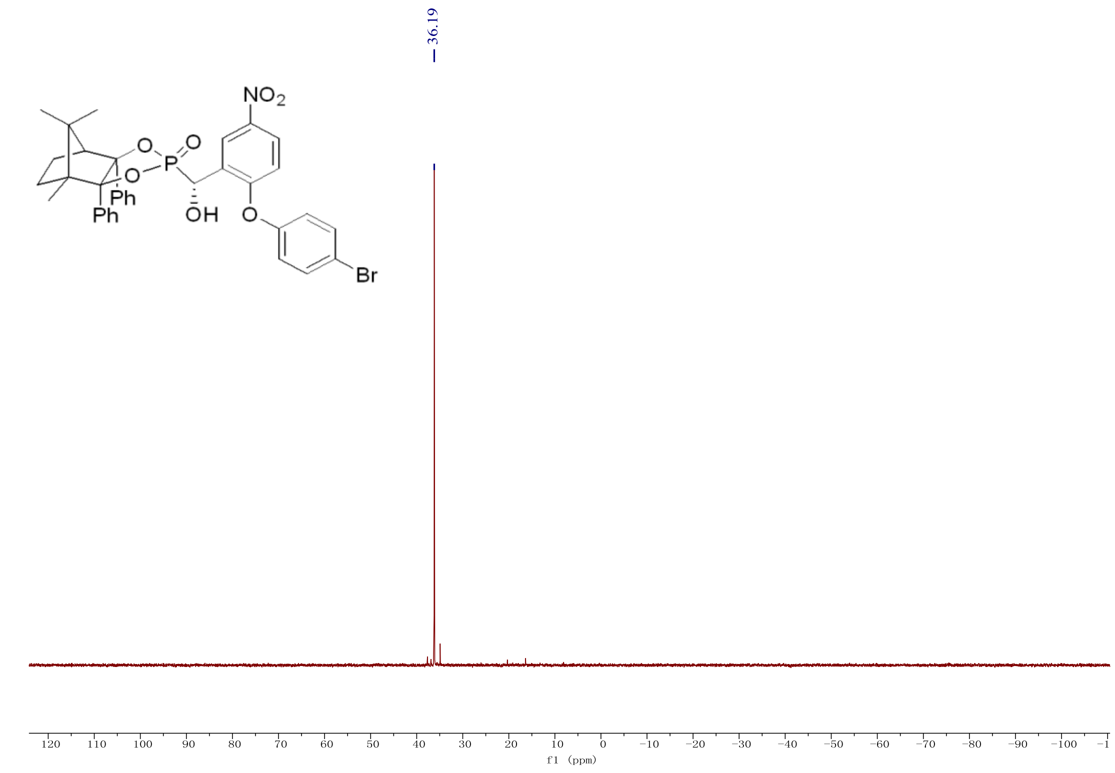

Fig. S61 ^{31}P NMR of compound **3r**

^{1}H NMR (300 MHz, $CDCl_3$)

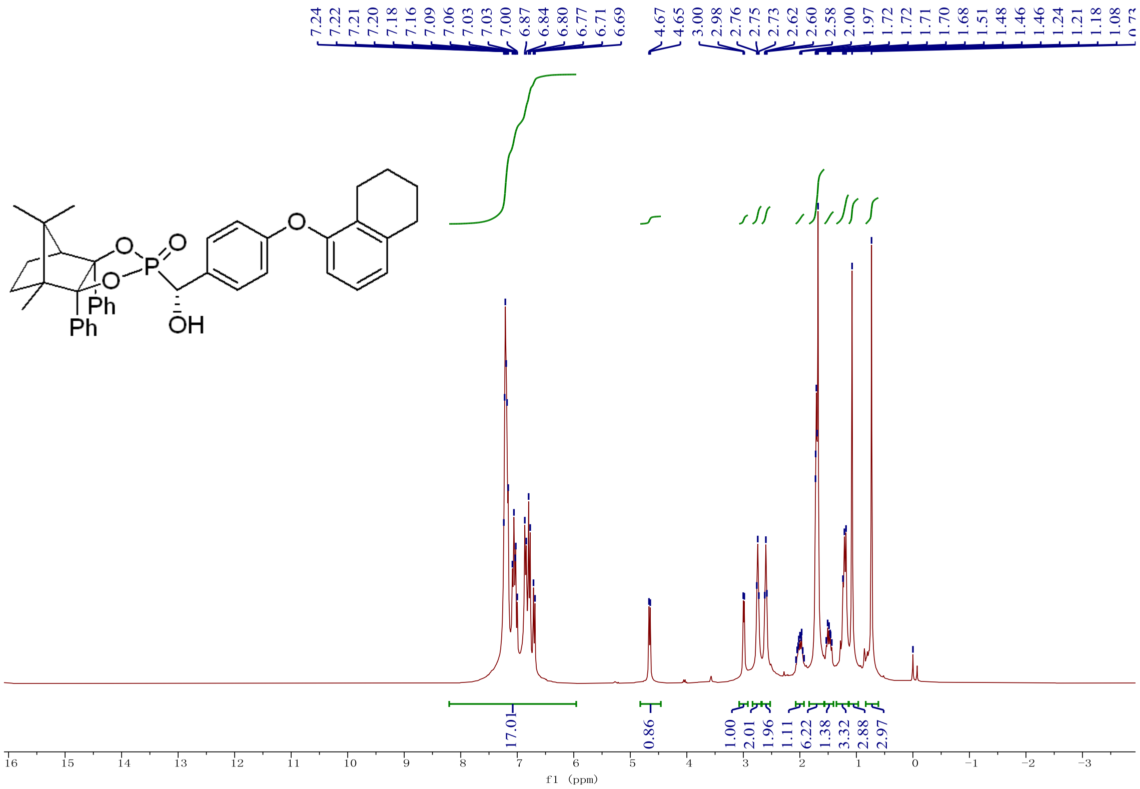

Fig. S62 ^{1}H NMR of compound **3s**

^{13}C NMR (75 MHz, $CDCl_3$)

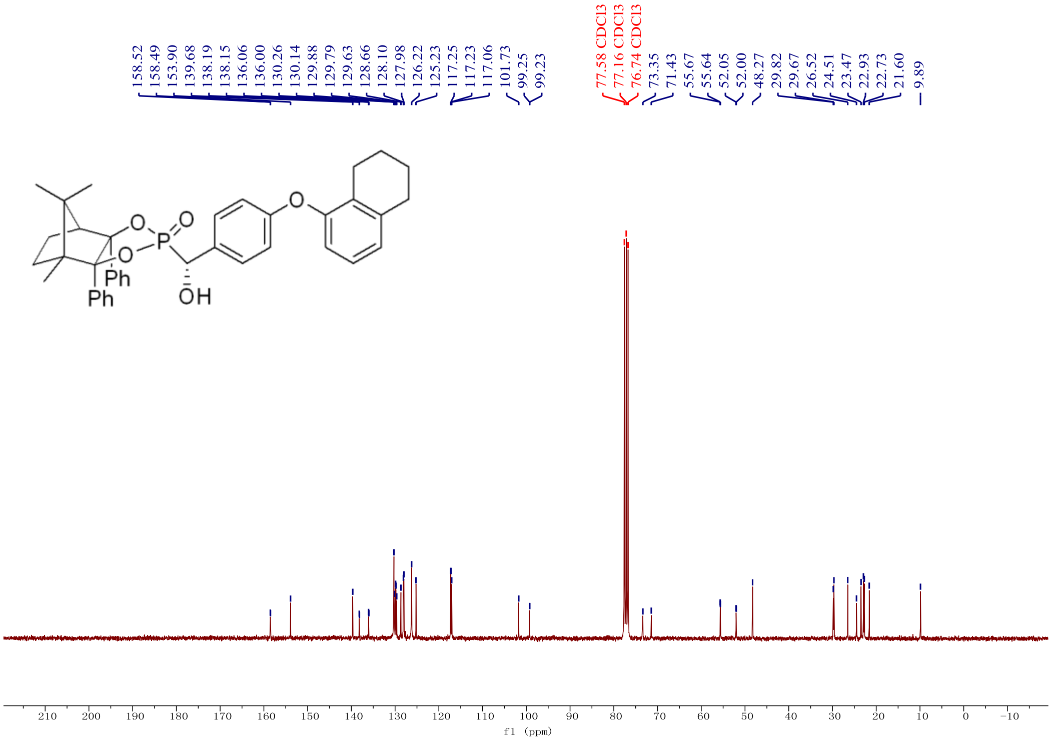

Fig. S63 ^{13}C NMR of compound **3s**

^{31}P NMR (121 MHz, $CDCl_3$)

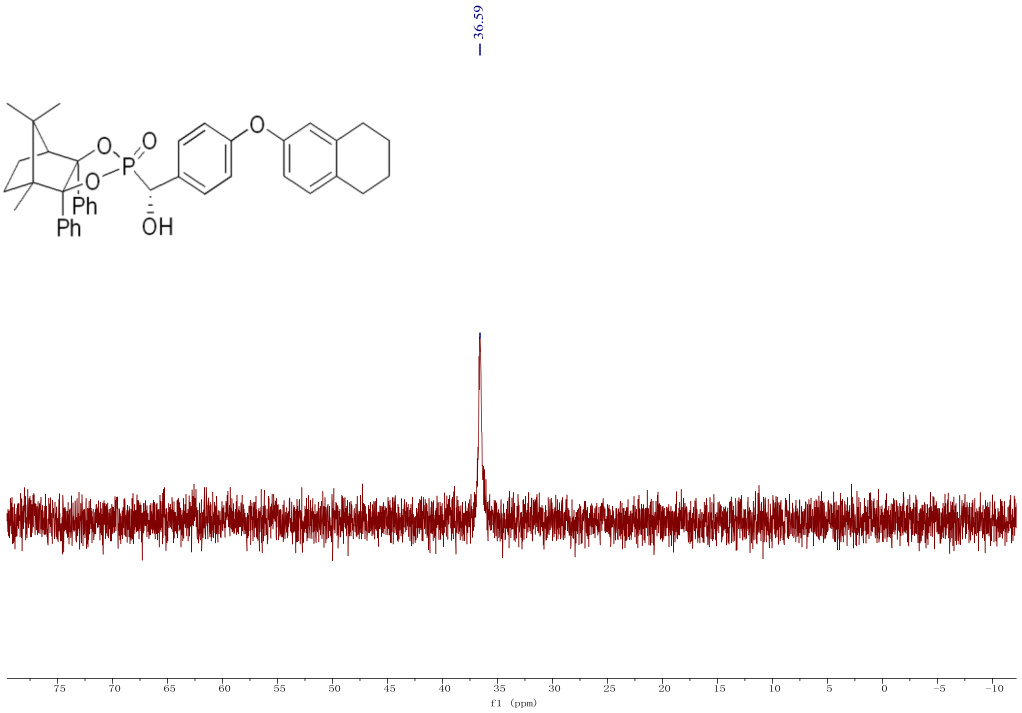

Fig. S64 ^{31}P NMR of compound **3s**

^{1}H NMR (300 MHz, $CDCl_3$)

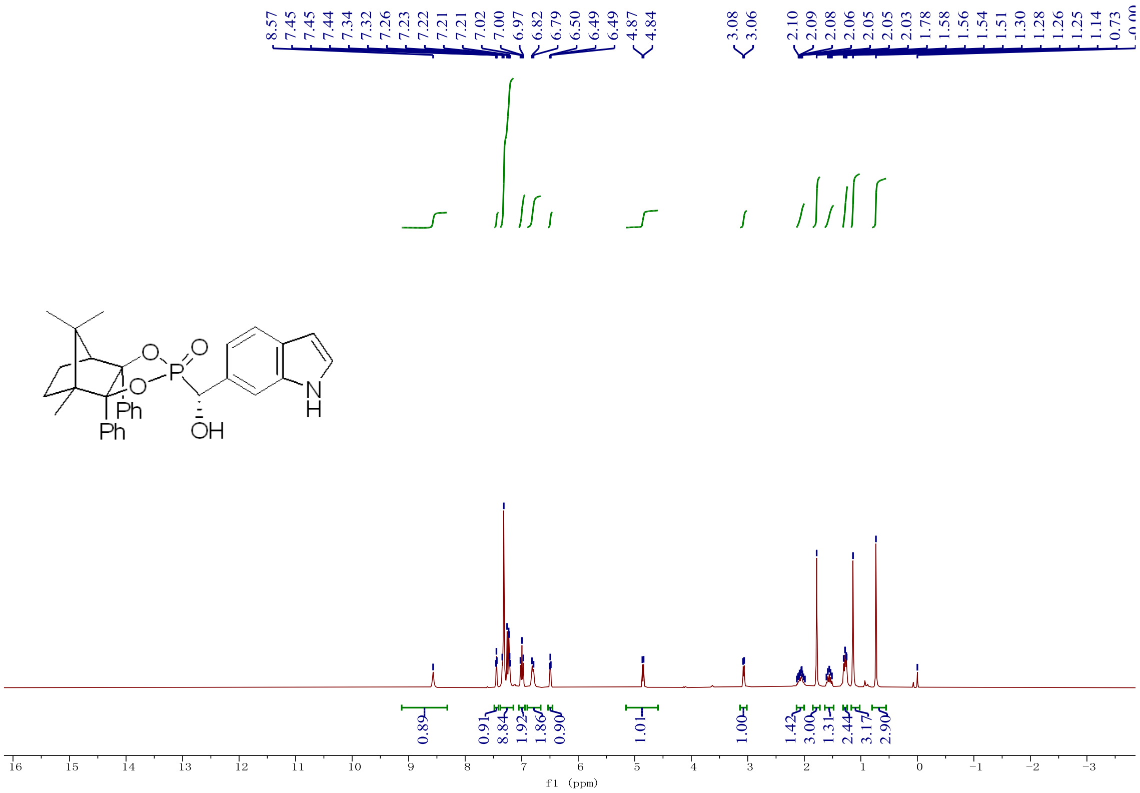

Fig. S65 ^{1}H NMR of compound **3t**

^{13}C NMR (75 MHz, $CDCl_3$)

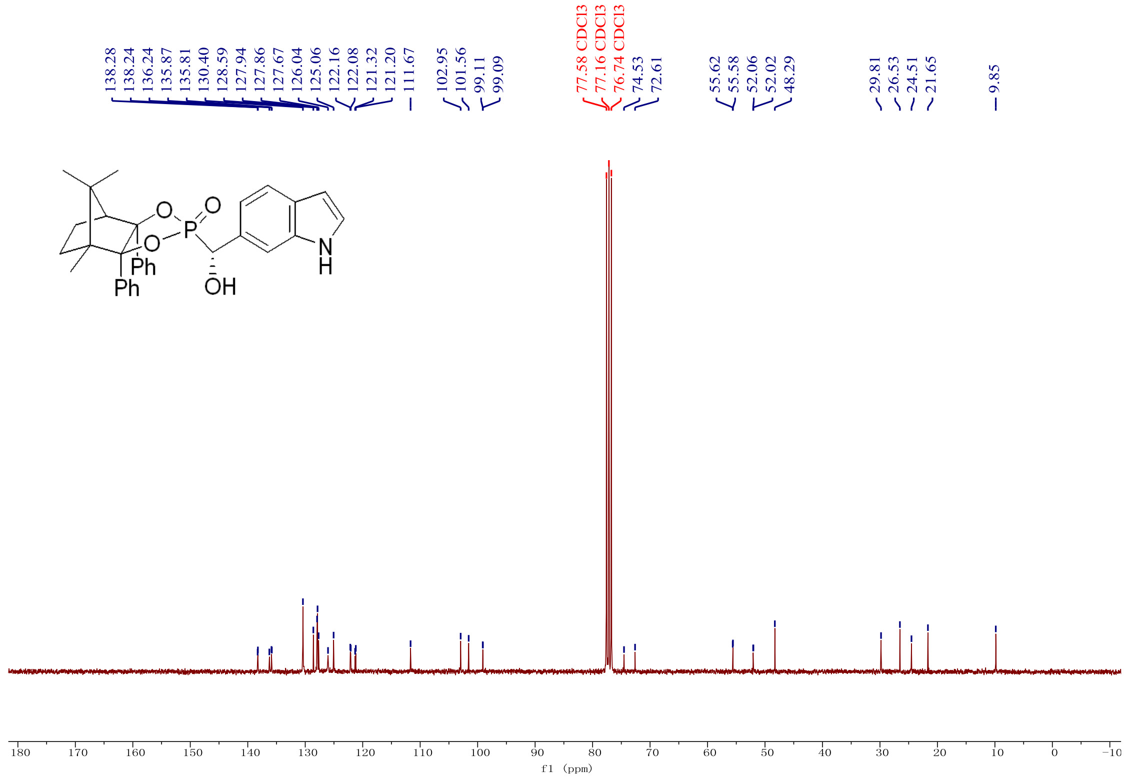

Fig. S66 ^{13}C NMR of compound **3t**

^{31}P NMR (121 MHz, $CDCl_3$)

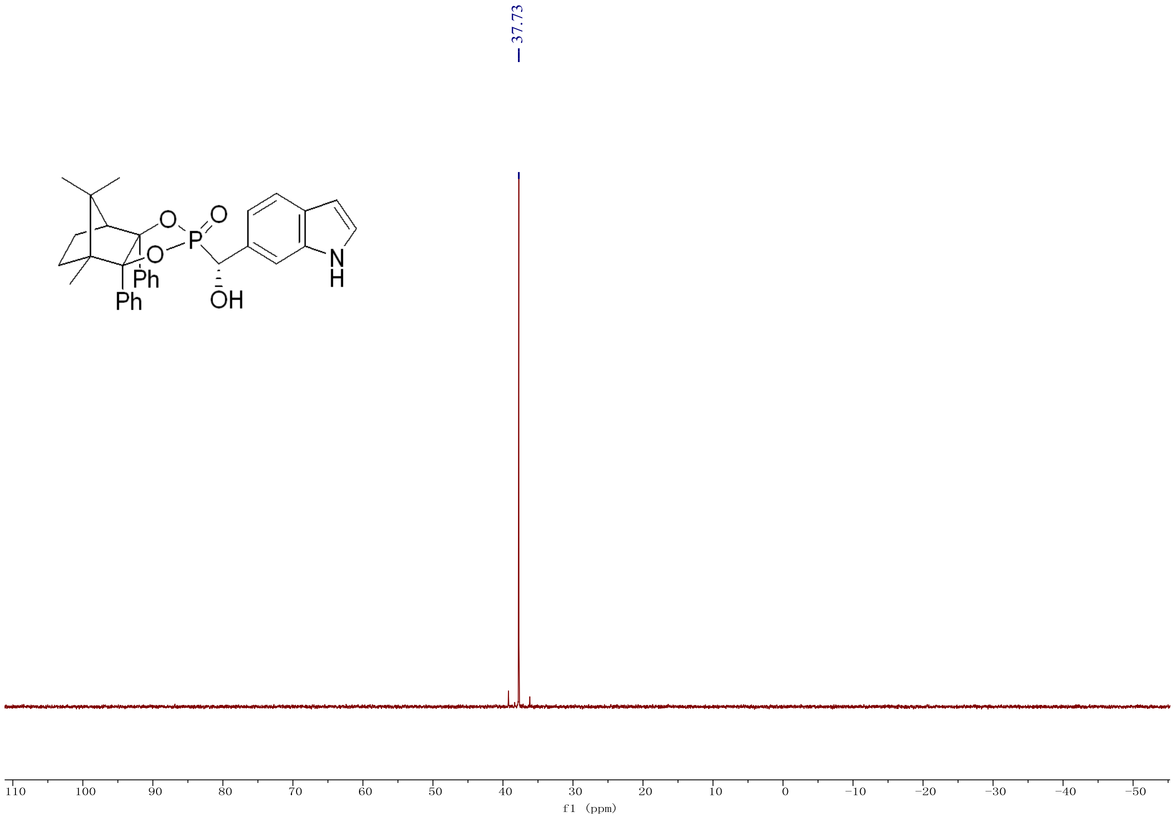

Fig. S67 ^{31}P NMR of compound **3t**

^{1}H NMR (300 MHz, $CDCl_3$)

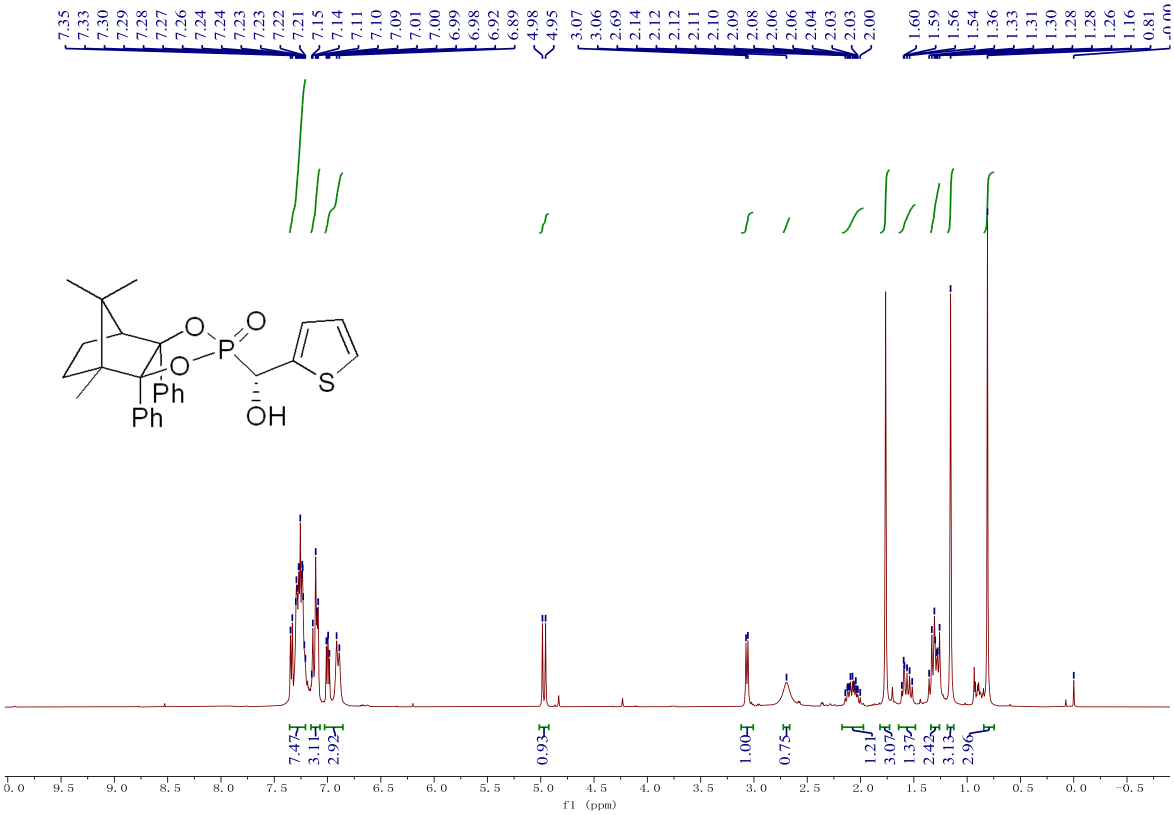

Fig. S68 ^{1}H NMR of compound **3u**

^{13}C NMR (75 MHz, $CDCl_3$)

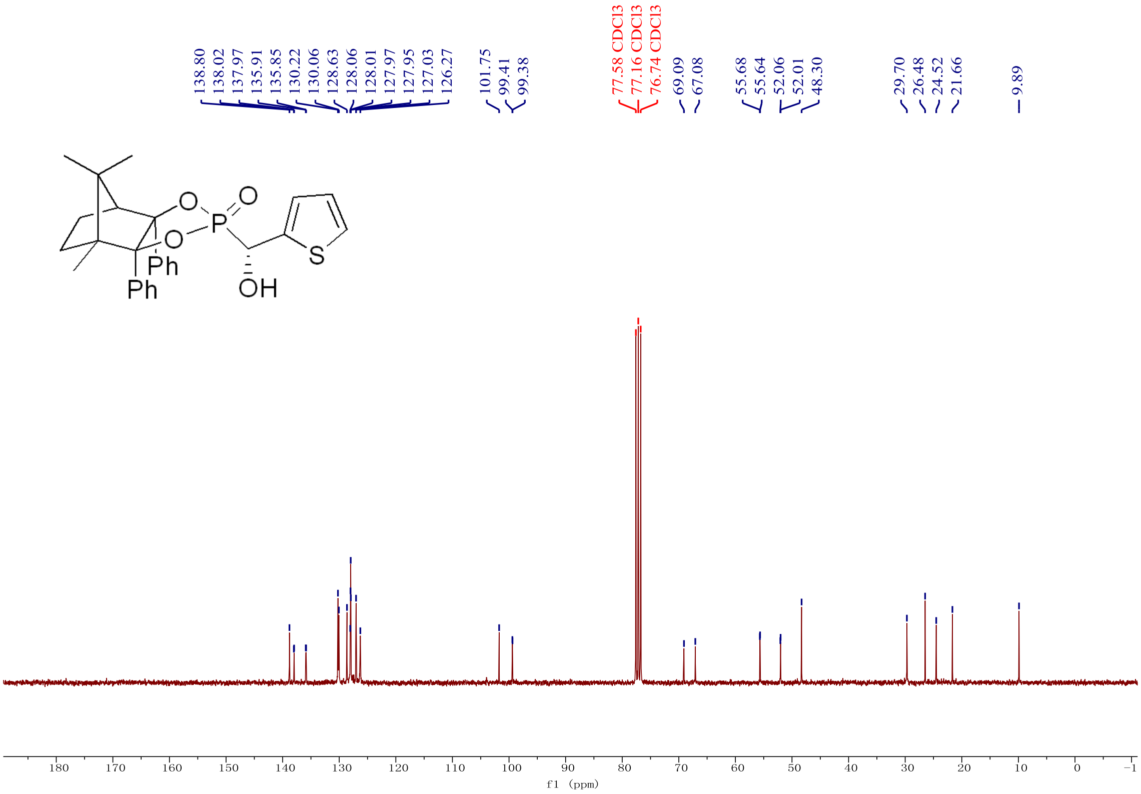

Fig. S69 ^{13}C NMR of compound **3u**

^{31}P NMR (121 MHz, $CDCl_3$)

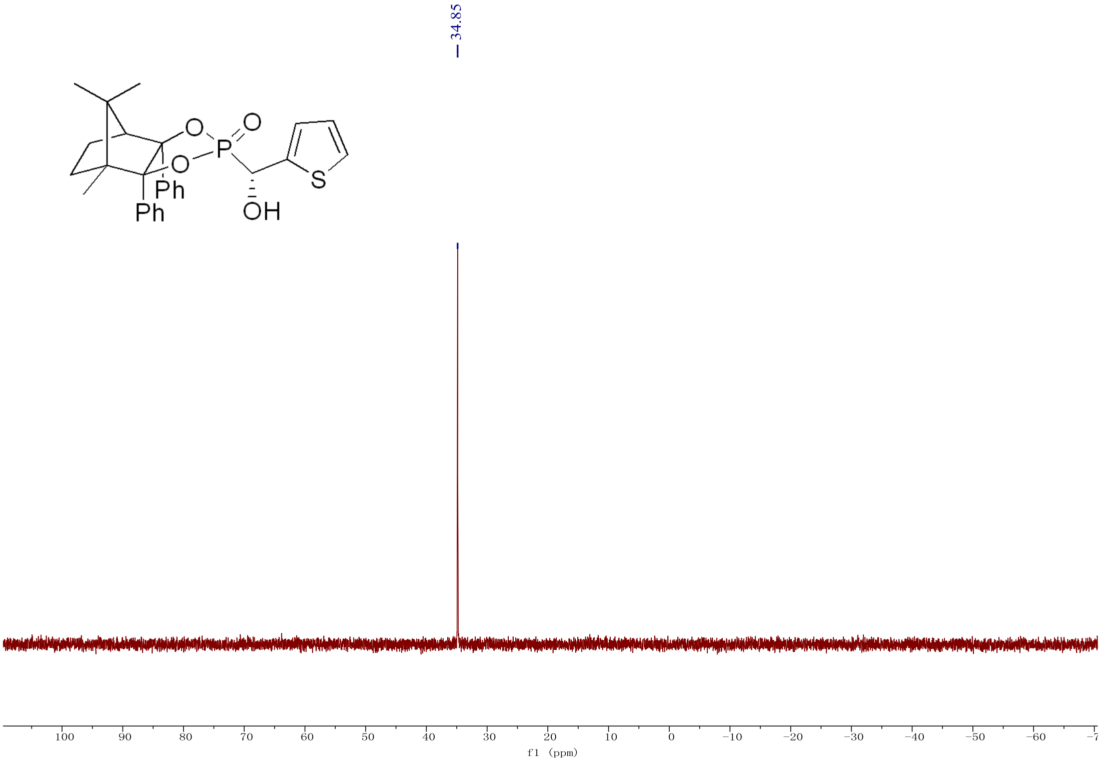

Fig. S70 ^{31}P NMR of compound **3u**

^{1}H NMR (300 MHz, $CDCl_3$)

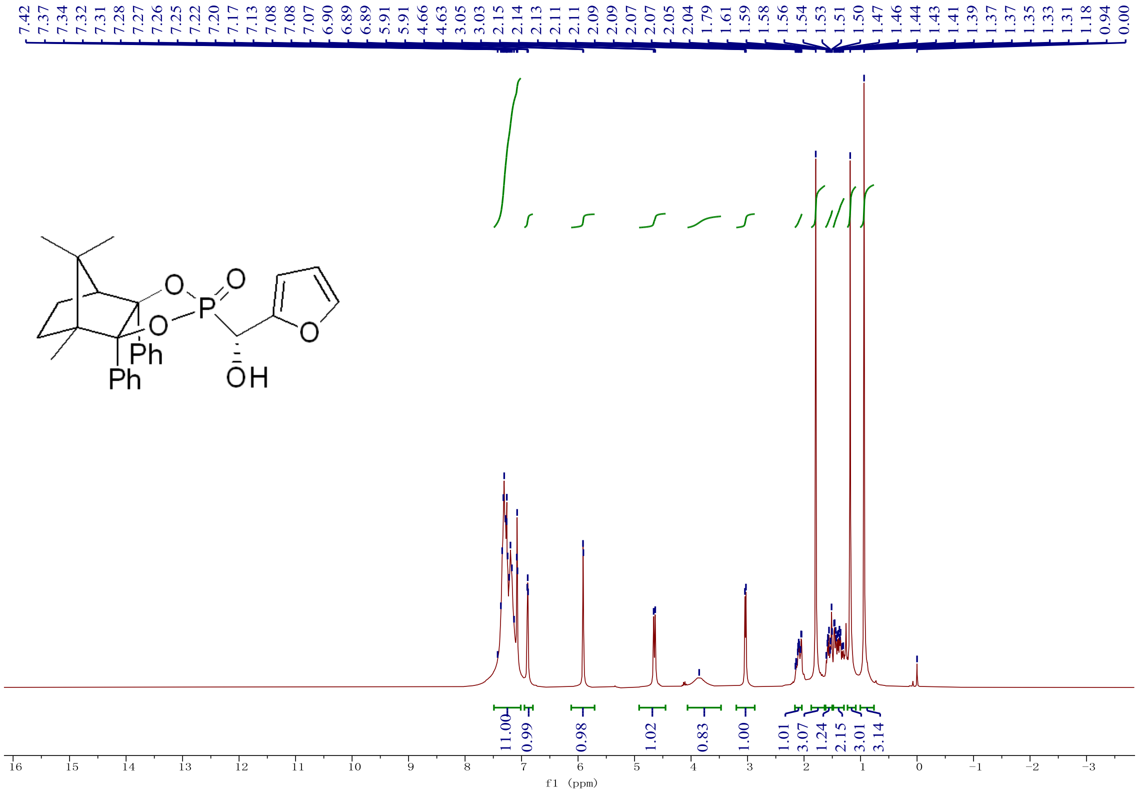

Fig. S71 ^{1}H NMR of compound **3v**

^{13}C NMR (75 MHz, $CDCl_3$)

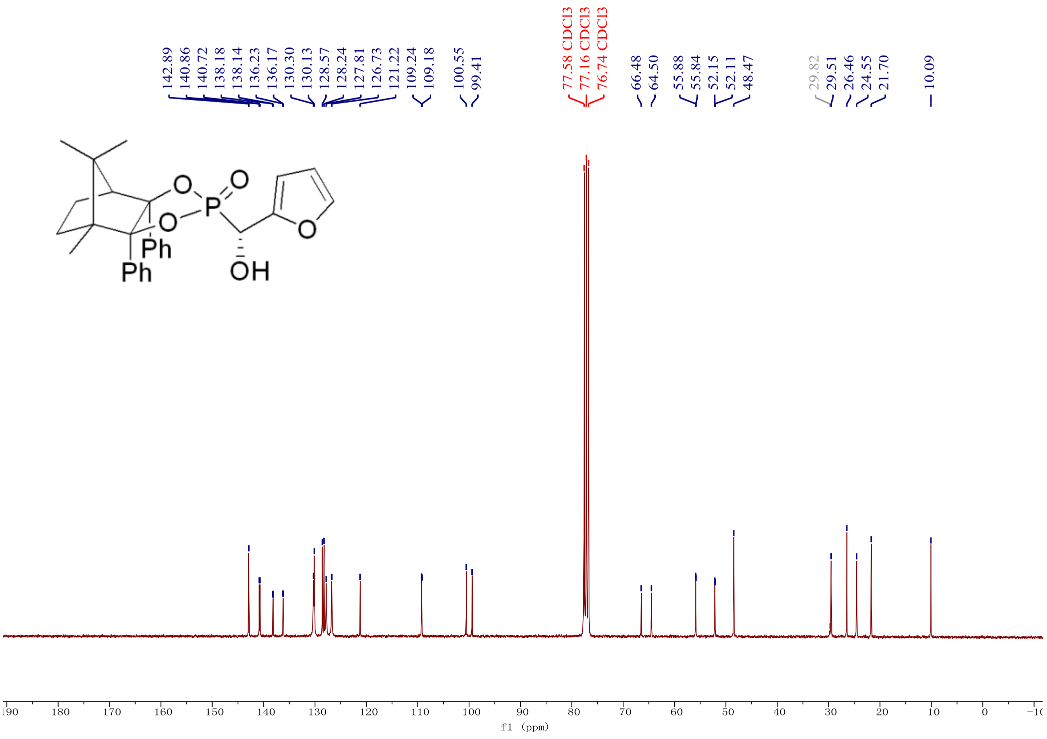

Fig. S72 ^{13}C NMR of compound **3v**

^{31}P NMR (121 MHz, $CDCl_3$)

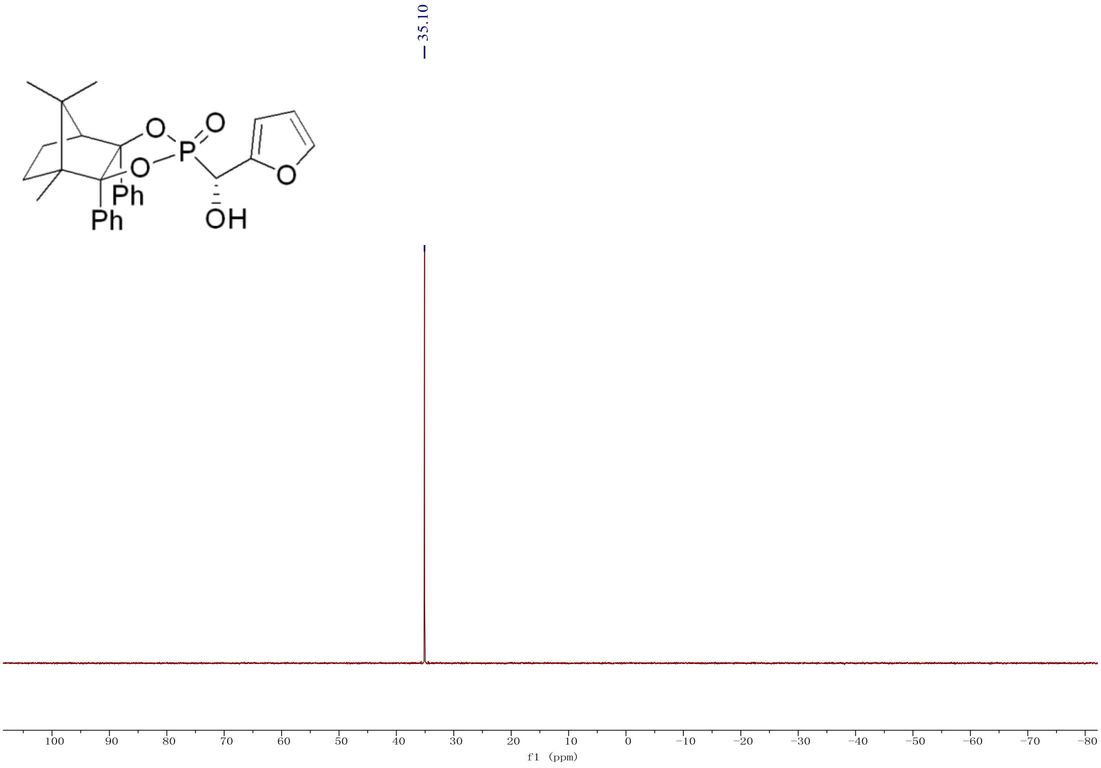

Fig. S73 ^{31}P NMR of compound **3v**

^{1}H NMR (300 MHz, $CDCl_3$)

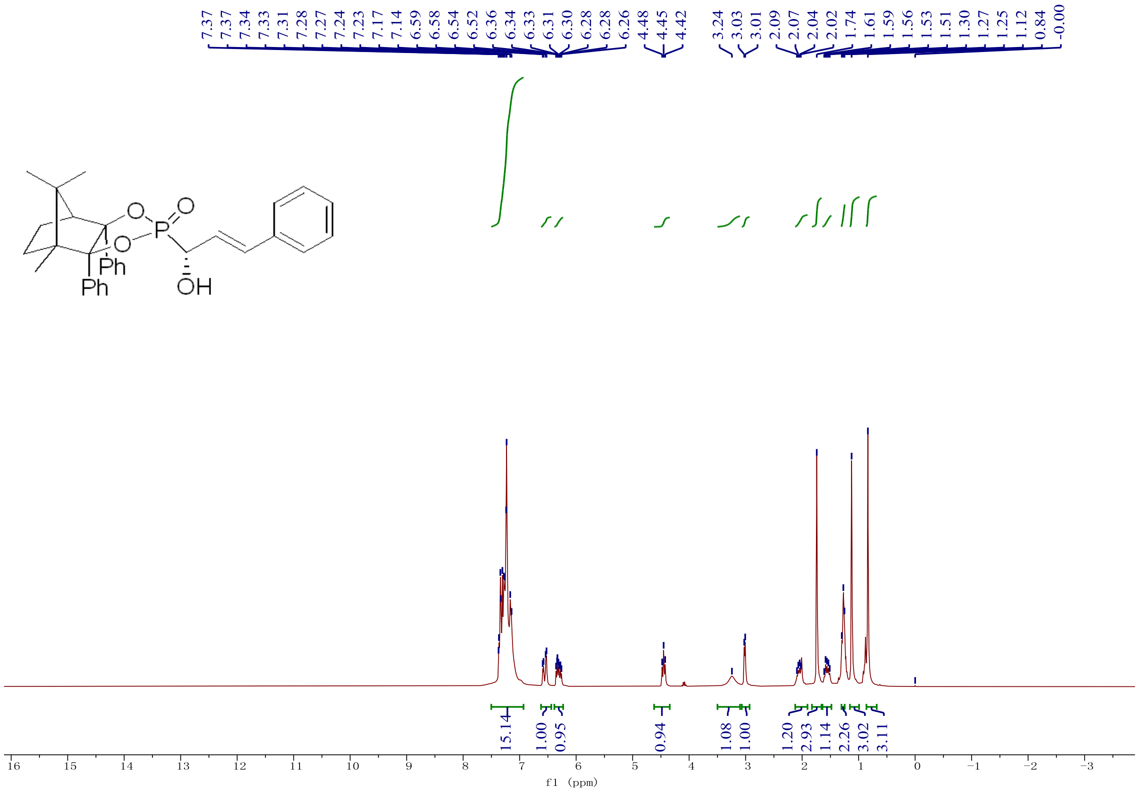

Fig. S74 ^{1}H NMR of compound **3w**

^{13}C NMR (75 MHz, $CDCl_3$)

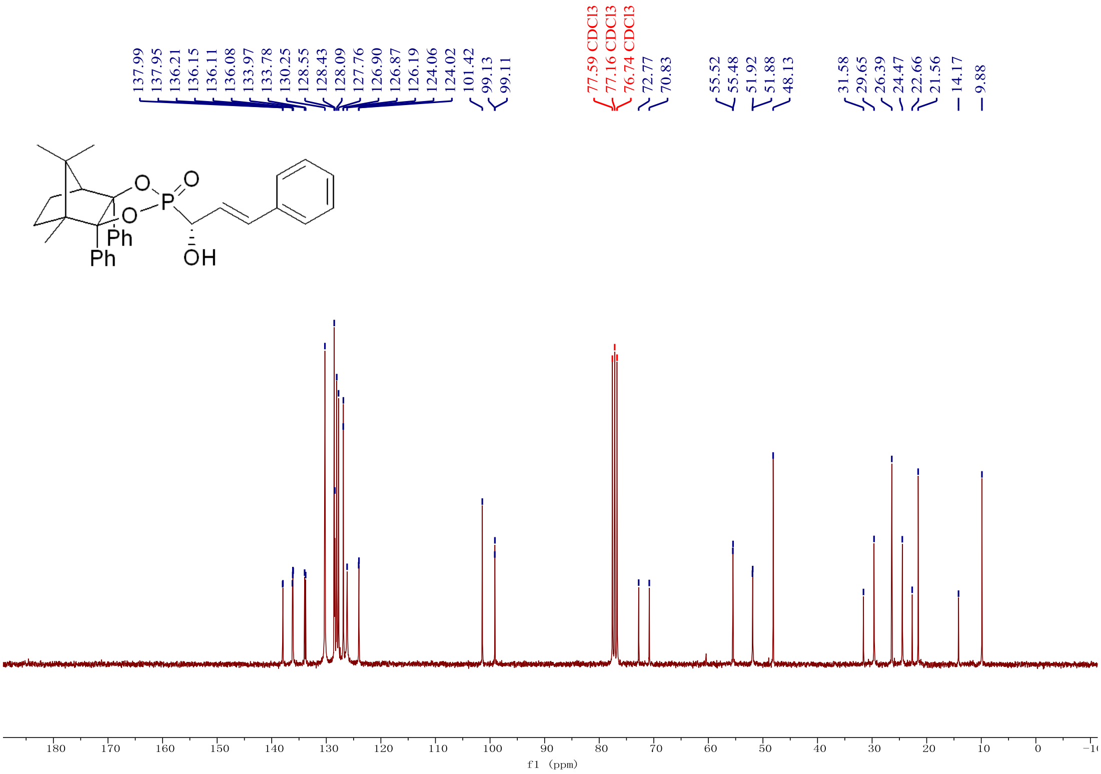

Fig. S75 ^{13}C NMR of compound **3w**

^{31}P NMR (121 MHz, $CDCl_3$)

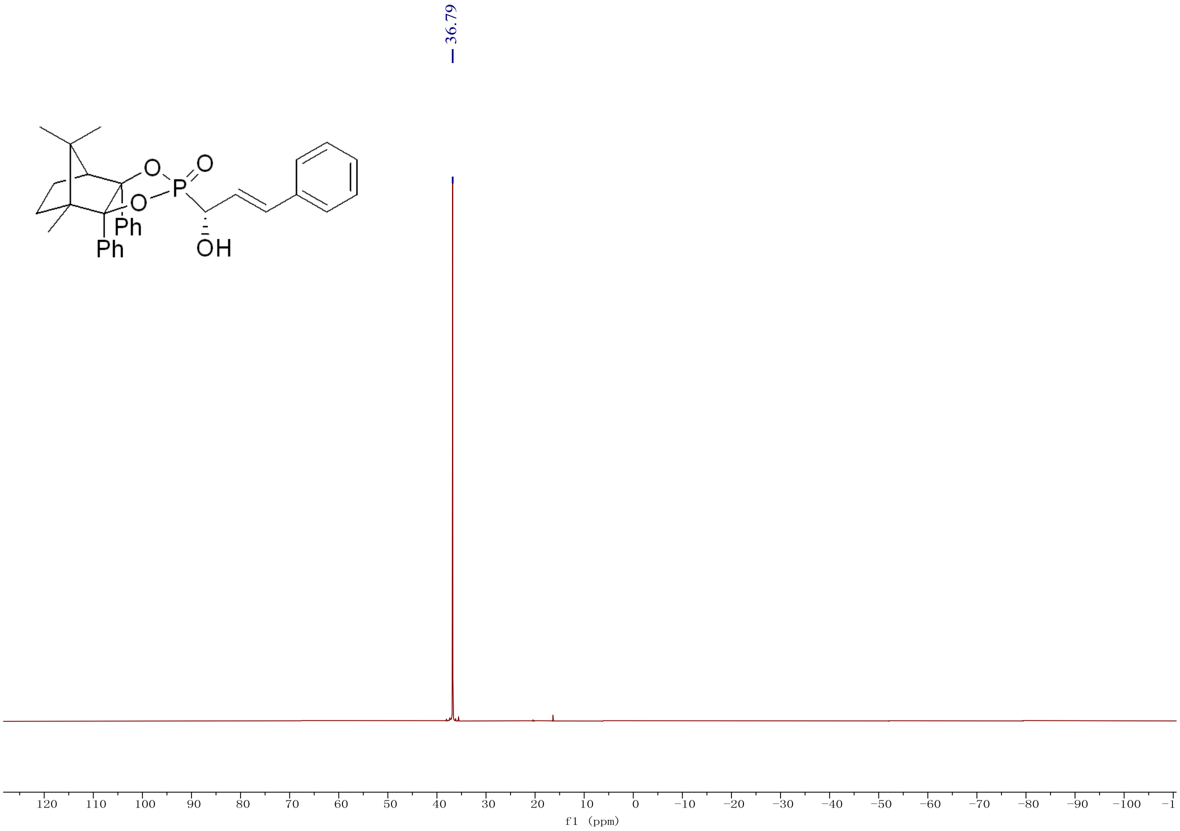

Fig. S76 ^{31}P NMR of compound **3w**

^{1}H NMR (300 MHz, $CDCl_3$)

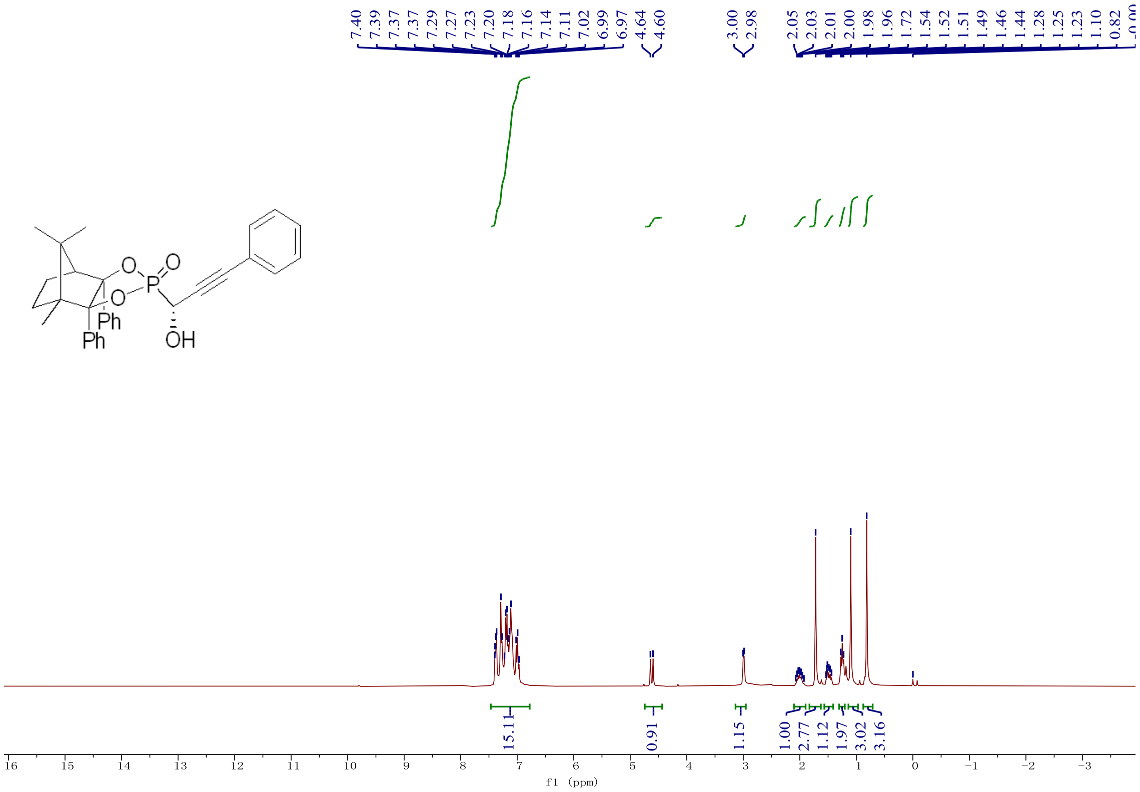

Fig. S77 ^{1}H NMR of compound **3x**

^{13}C NMR (75 MHz, $CDCl_3$)

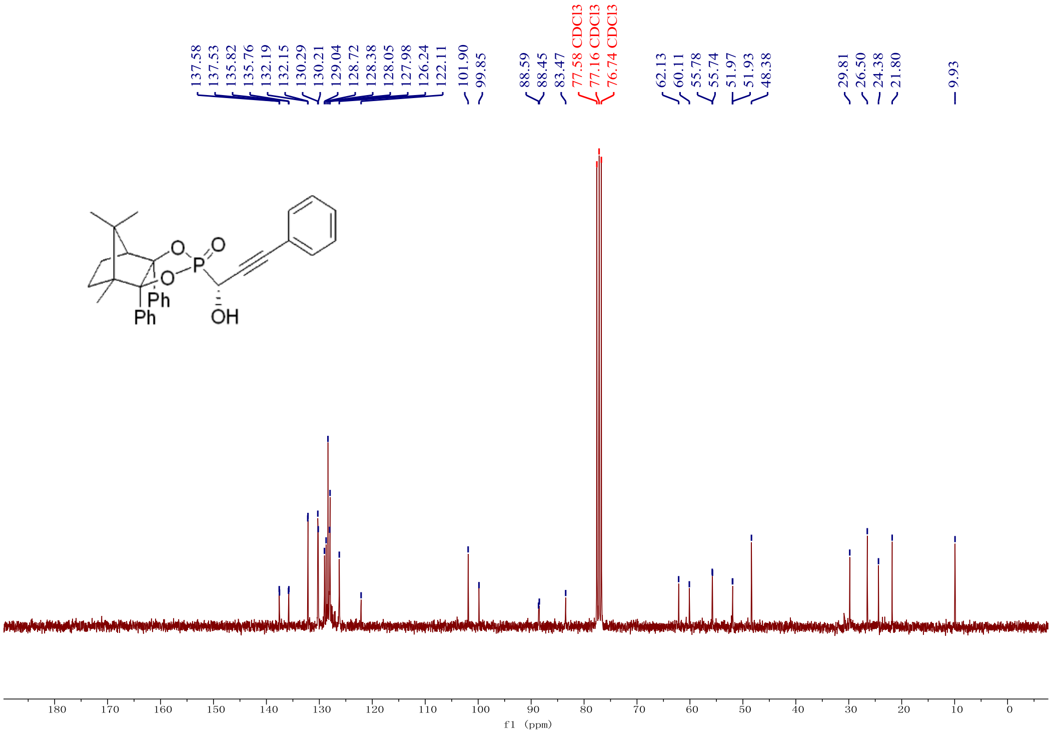

Fig. S78 ^{13}C NMR of compound **3x**

^{31}P NMR (121 MHz, $CDCl_3$)

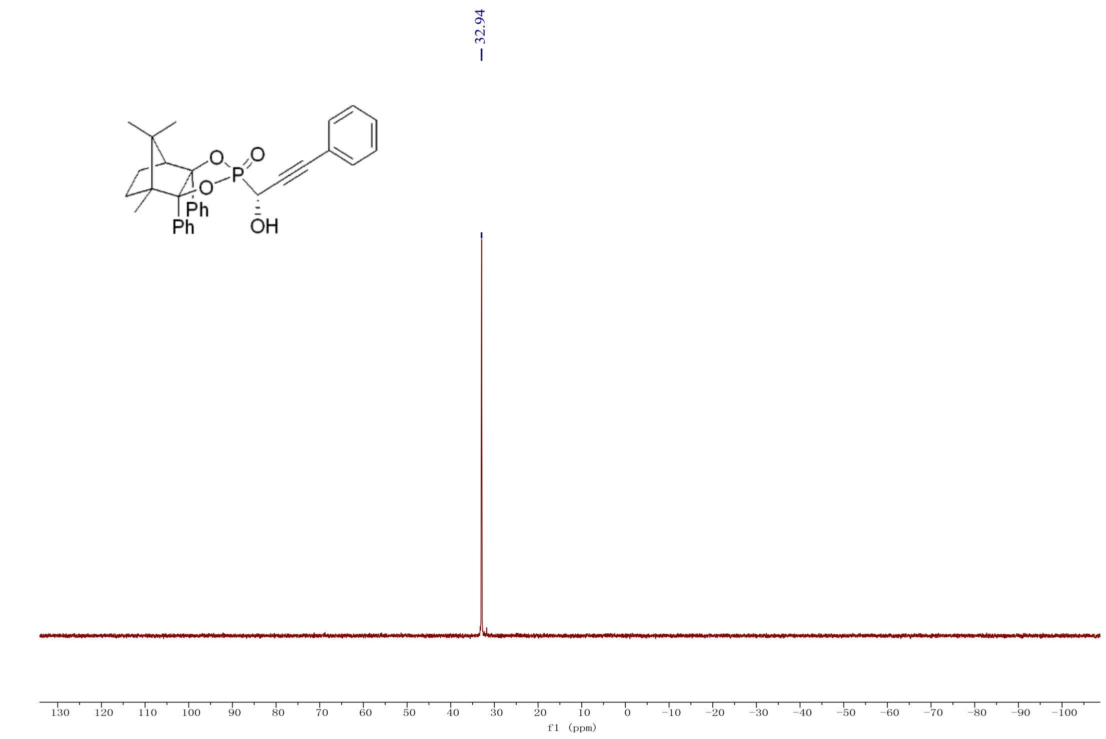

Fig. S79 ^{31}P NMR of compound **3x**

^{1}H NMR (300 MHz, $CDCl_3$)

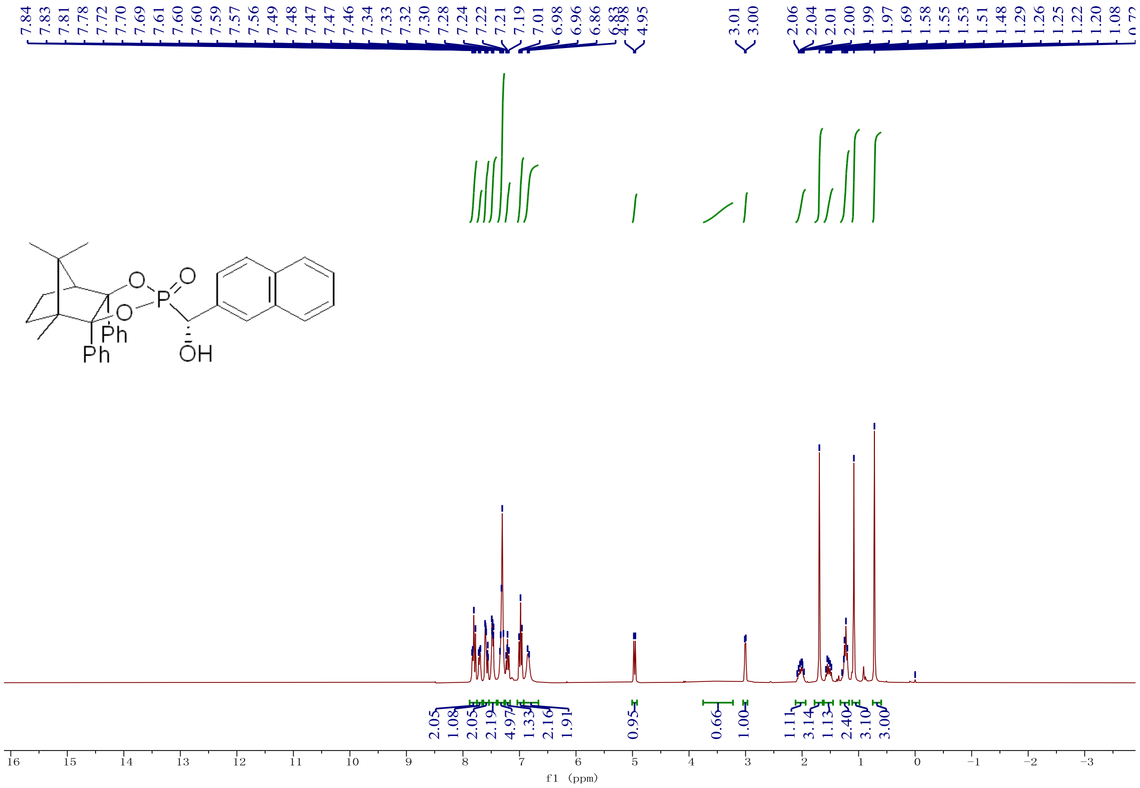

Fig. S80 ^{1}H NMR of compound **3y**

^{13}C NMR (75 MHz, $CDCl_3$)

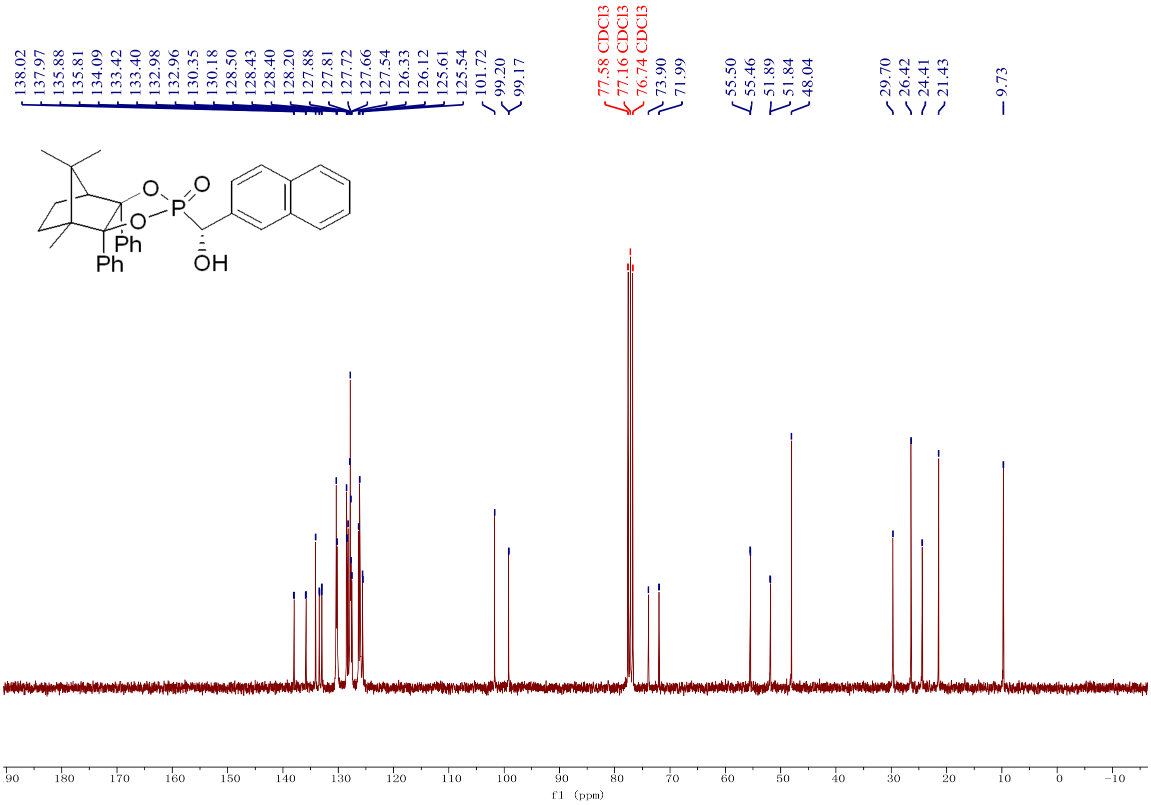

Fig. S81 ^{13}C NMR of compound **3y**

^{31}P NMR (121 MHz, $CDCl_3$)

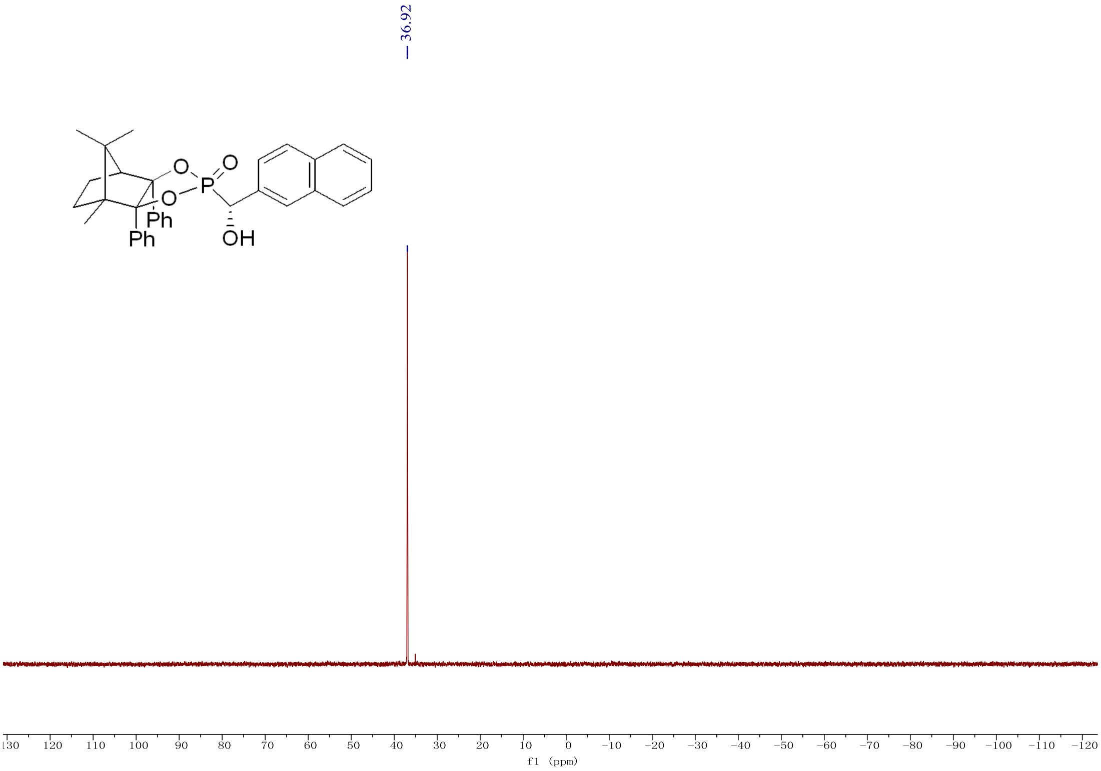

Fig. S82 ^{31}P NMR of compound **3y**

^{1}H NMR (300 MHz, $CDCl_3$)

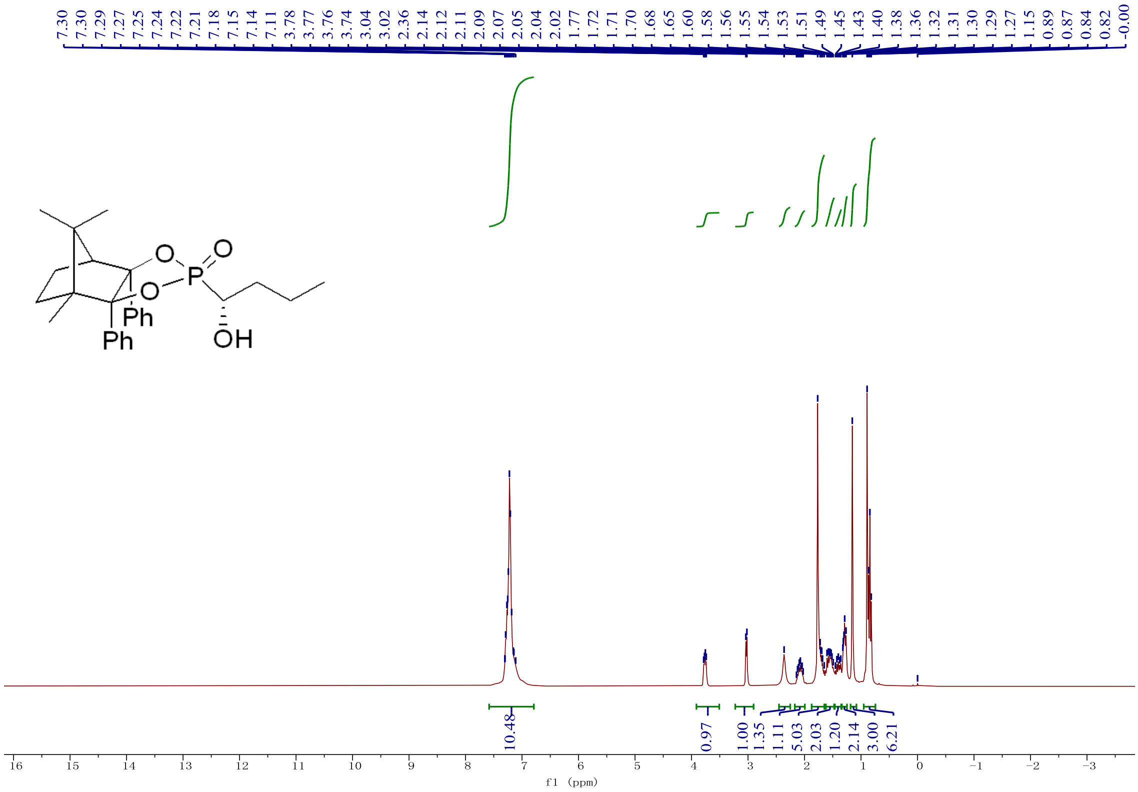

Fig. S83 ^{1}H NMR of compound **3za**

^{13}C NMR (75 MHz, $CDCl_3$)

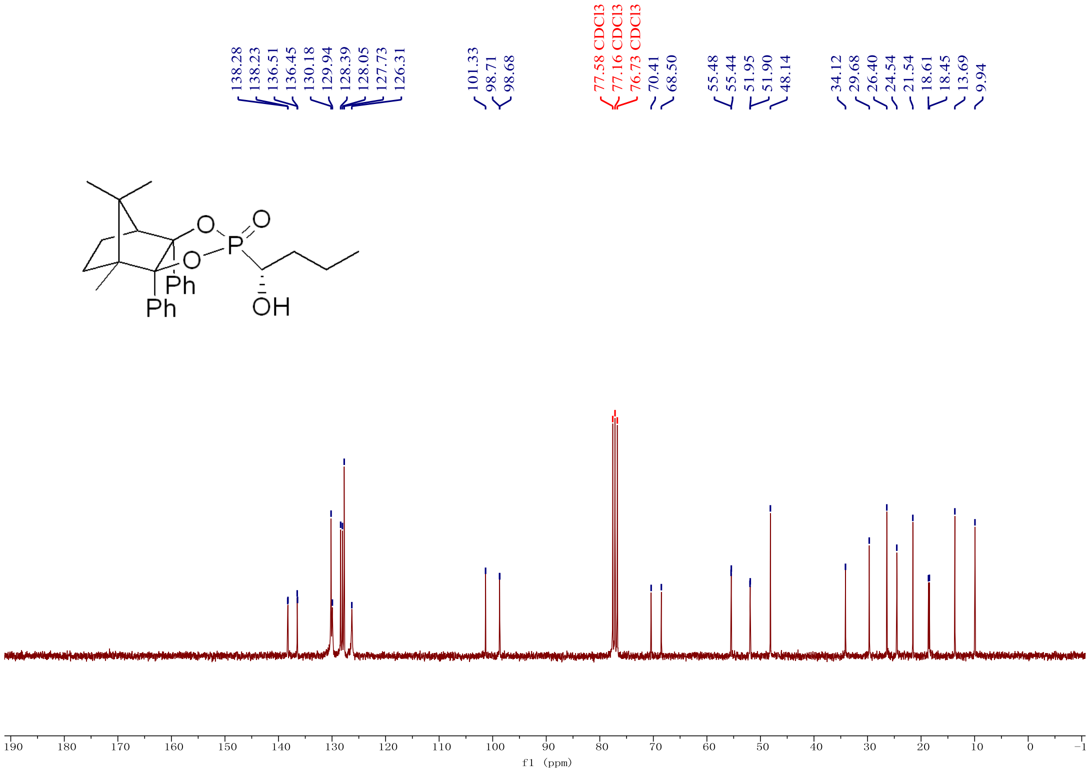

Fig. S84 ^{13}C NMR of compound **3za**

^{31}P NMR (121 MHz, $CDCl_3$)

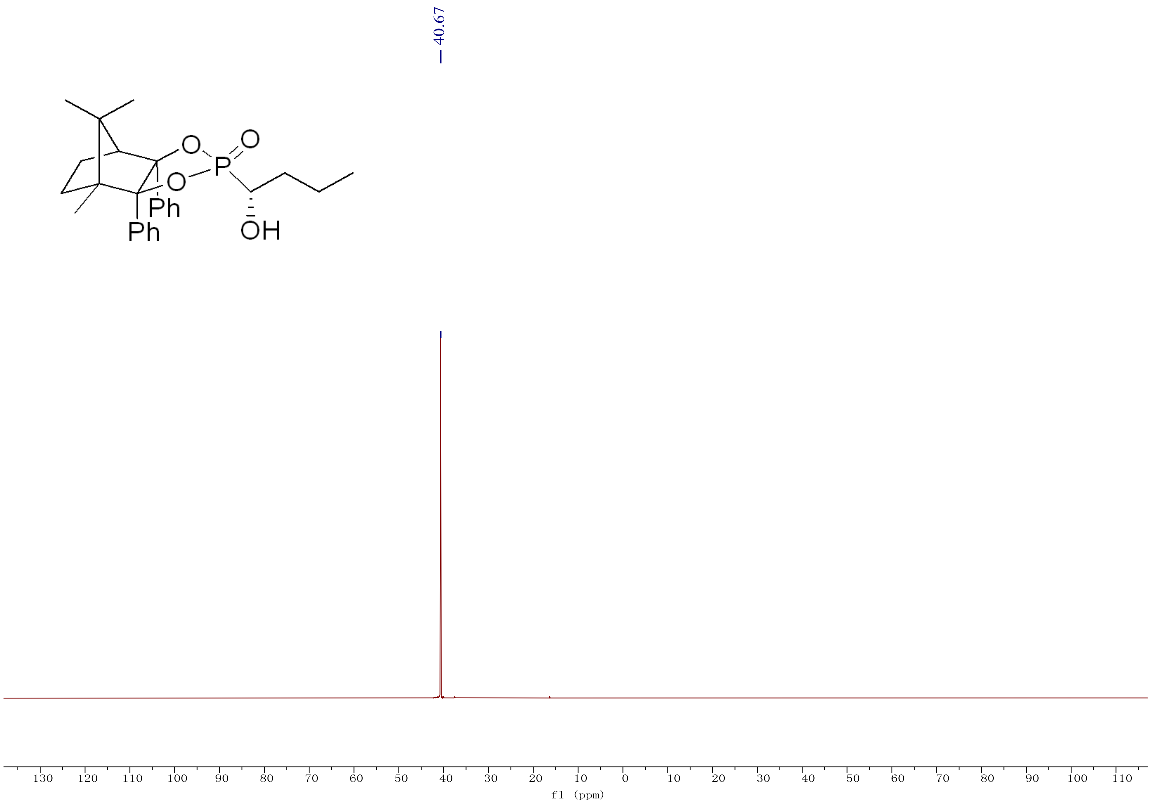

Fig. S85 ^{31}P NMR of compound **3za**

^{1}H NMR (300 MHz, $CDCl_3$)

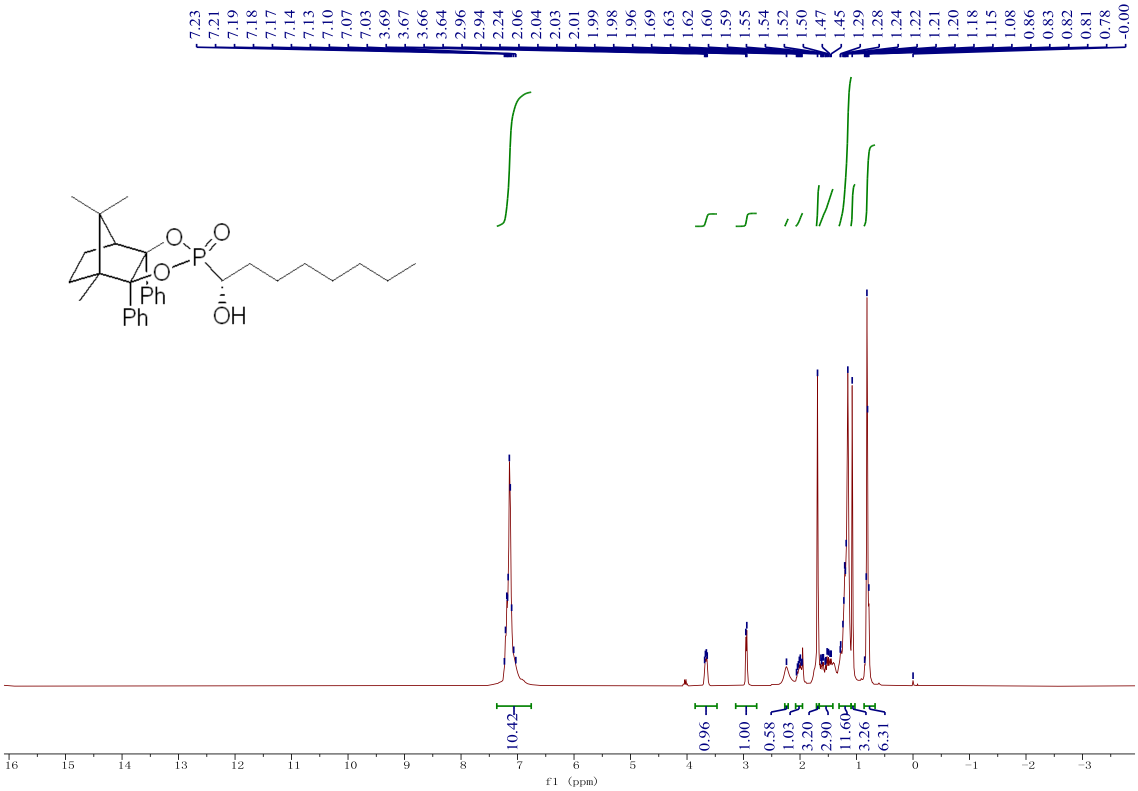

Fig. S86 ^{1}H NMR of compound **3zb**

^{13}C NMR (75 MHz, $CDCl_3$)

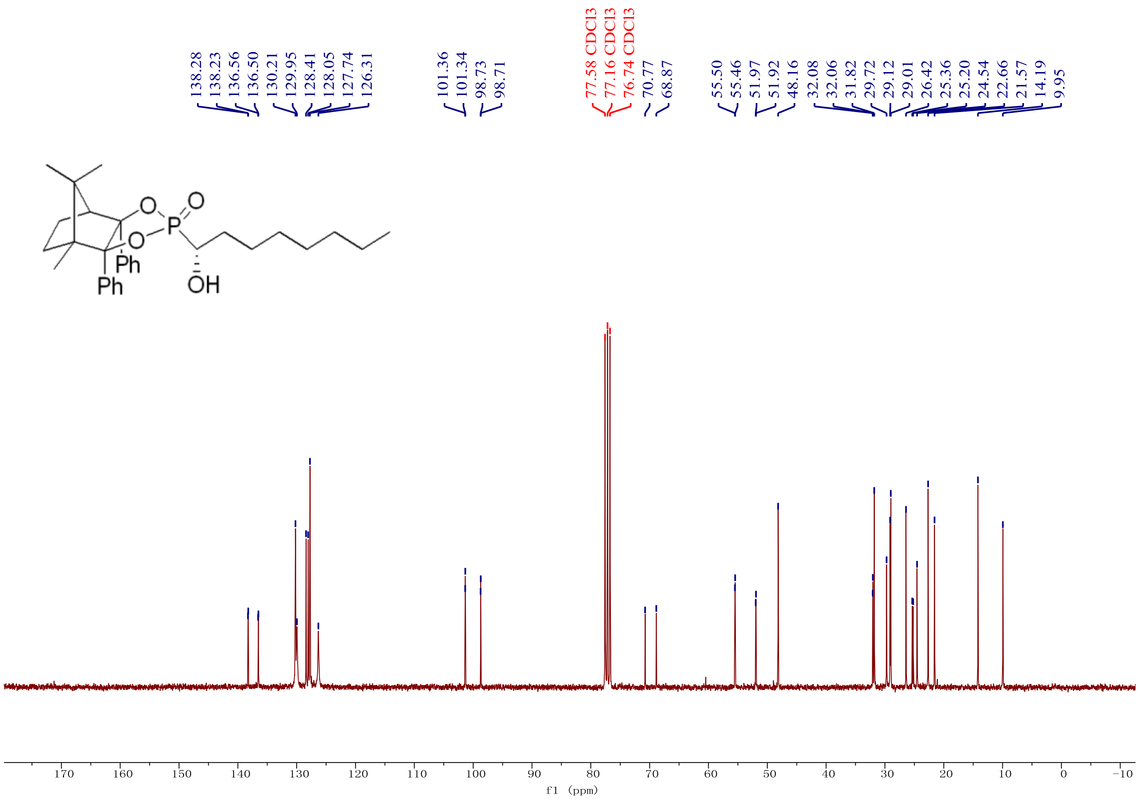

Fig. S87 ^{13}C NMR of compound **3zb**

^{31}P NMR (121 MHz, $CDCl_3$)

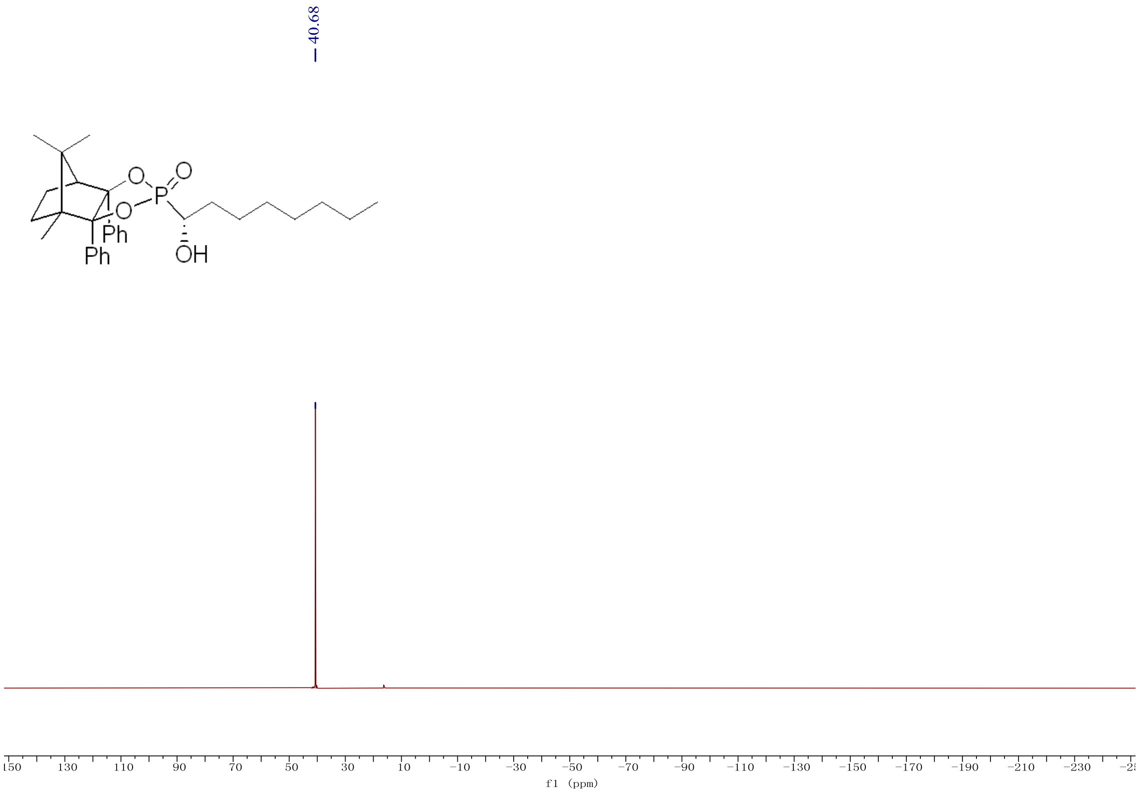

Fig. S88 ^{31}P NMR of compound **3zb**

H NMR (300 MHz, $CDCl_3$)

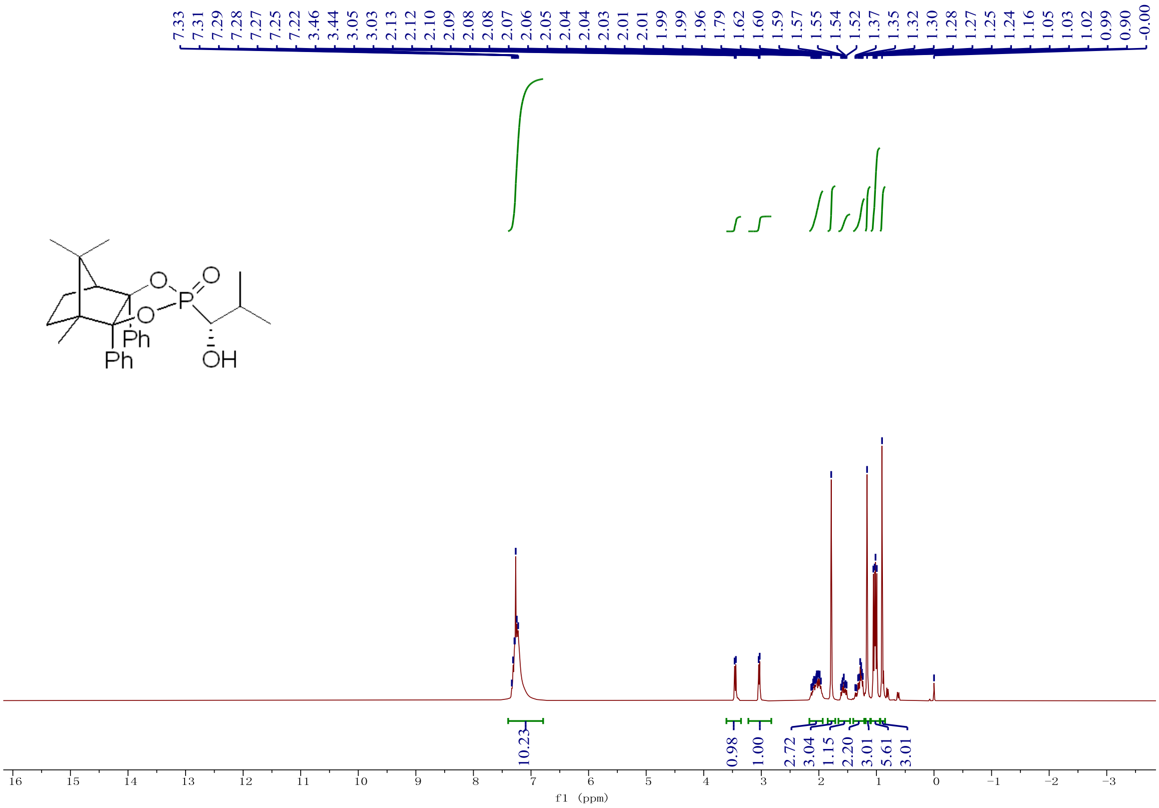

Fig. S89 ^{1}H NMR of compound **3zc**

^{13}C NMR (75 MHz, $CDCl_3$)

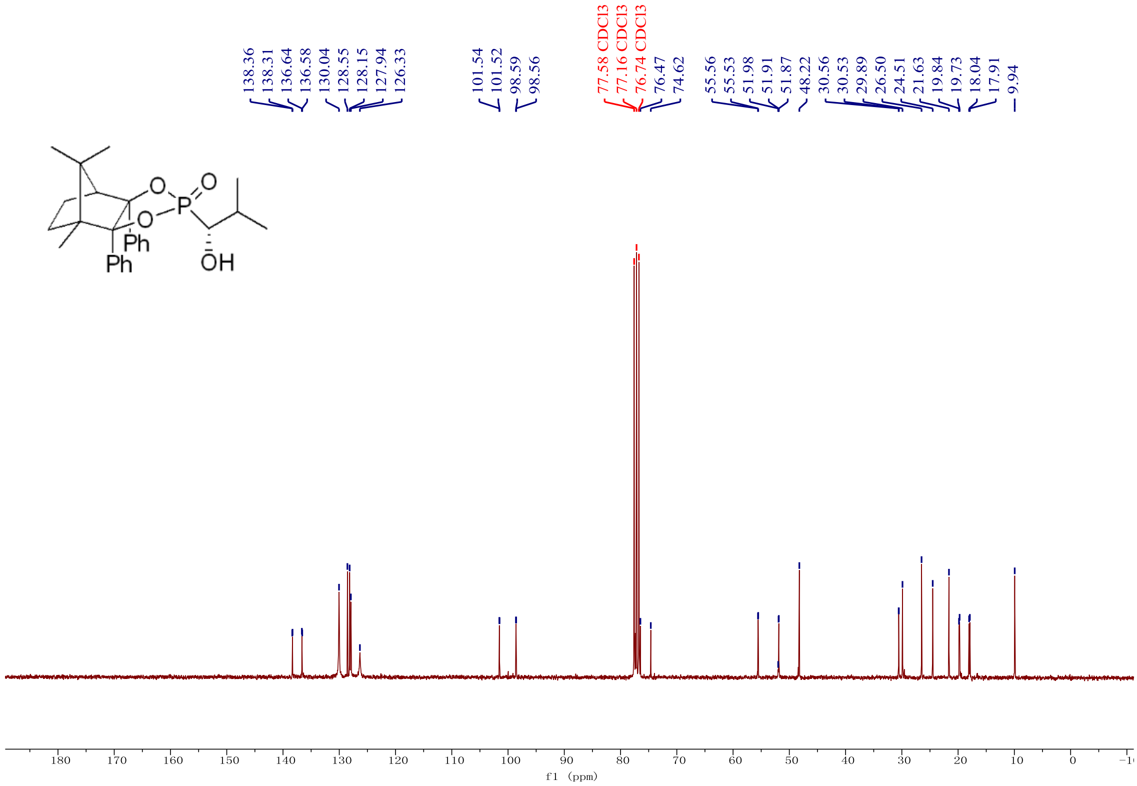

Fig. S90 ^{13}C NMR of compound **3zc**

^{31}P NMR (121 MHz, $CDCl_3$)

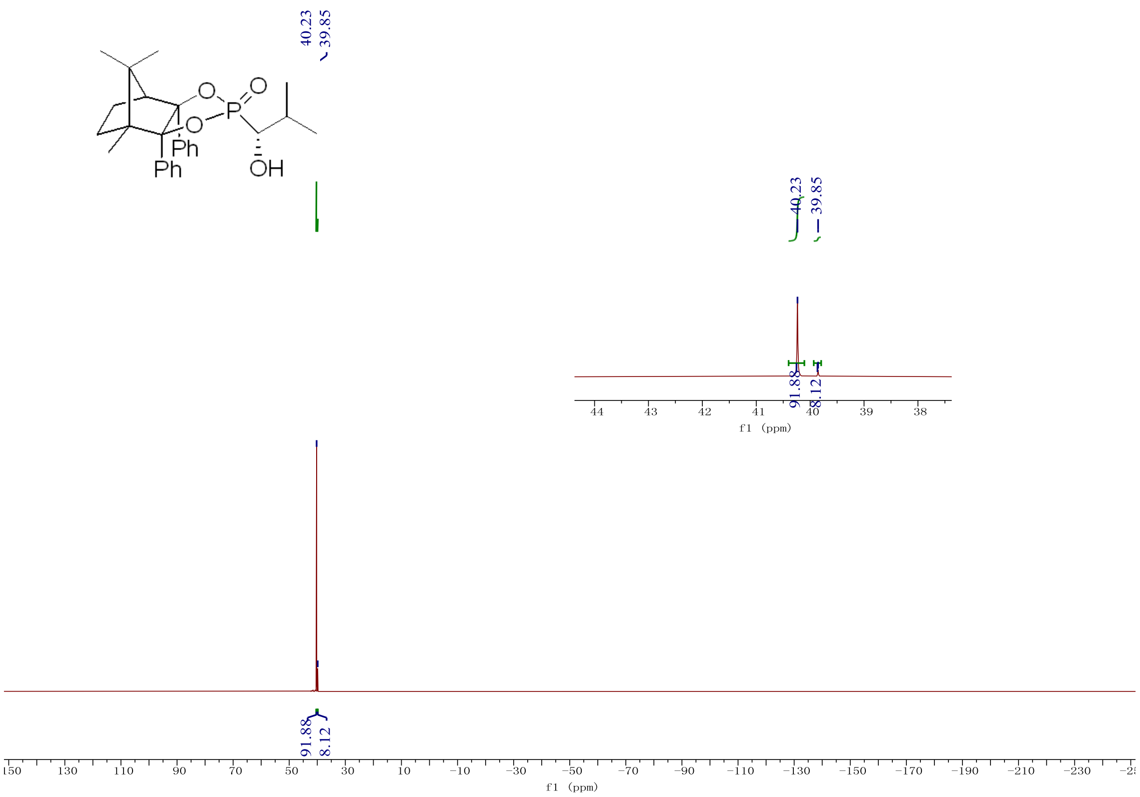

Fig. S91 ^{31}P NMR of compound **3zc**

^{1}H NMR (300 MHz, $CDCl_3$)

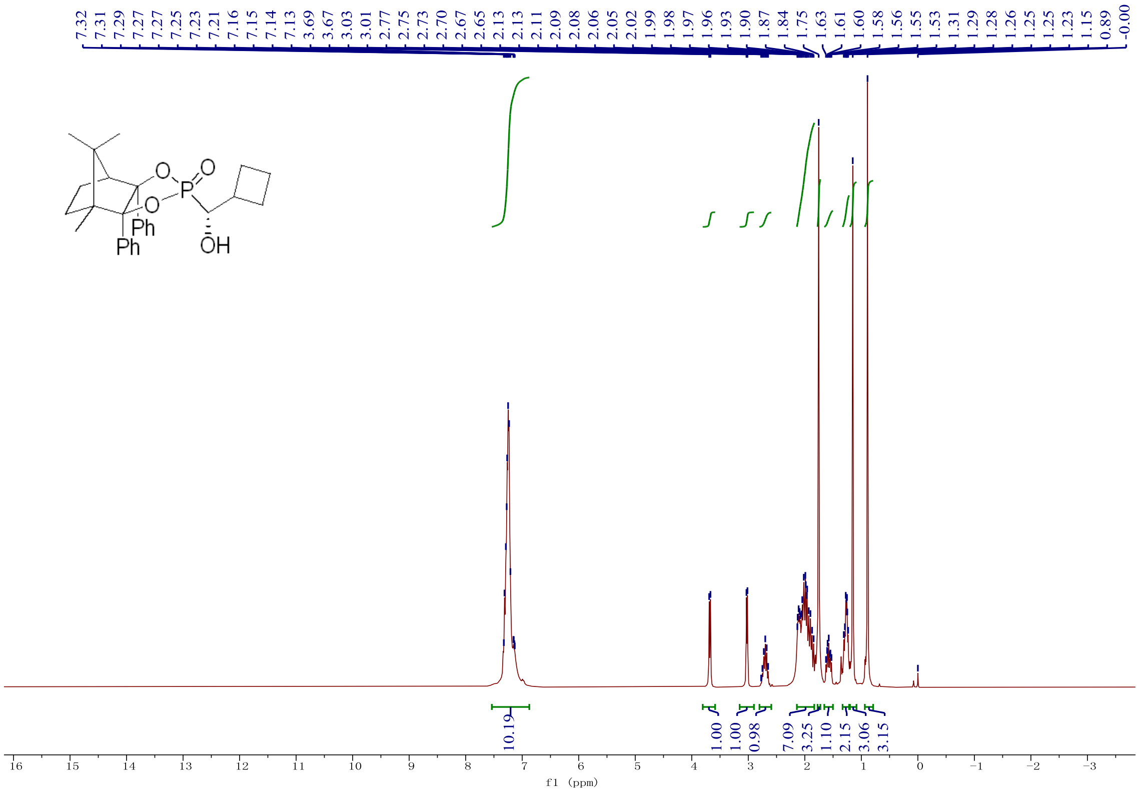

Fig. S92 ^{1}H NMR of compound **3zd**

^{13}C NMR (75 MHz, $CDCl_3$)

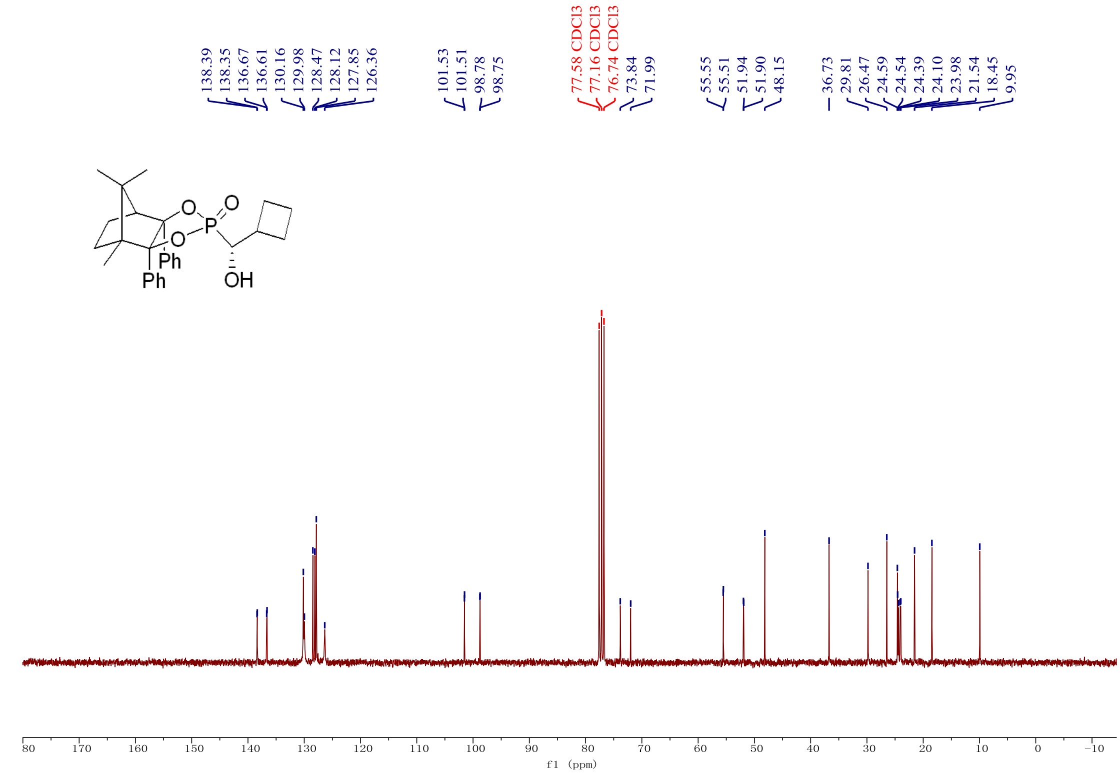

Fig. S93 ^{13}C NMR of compound **3zd**

^{31}P NMR (121 MHz, $CDCl_3$)

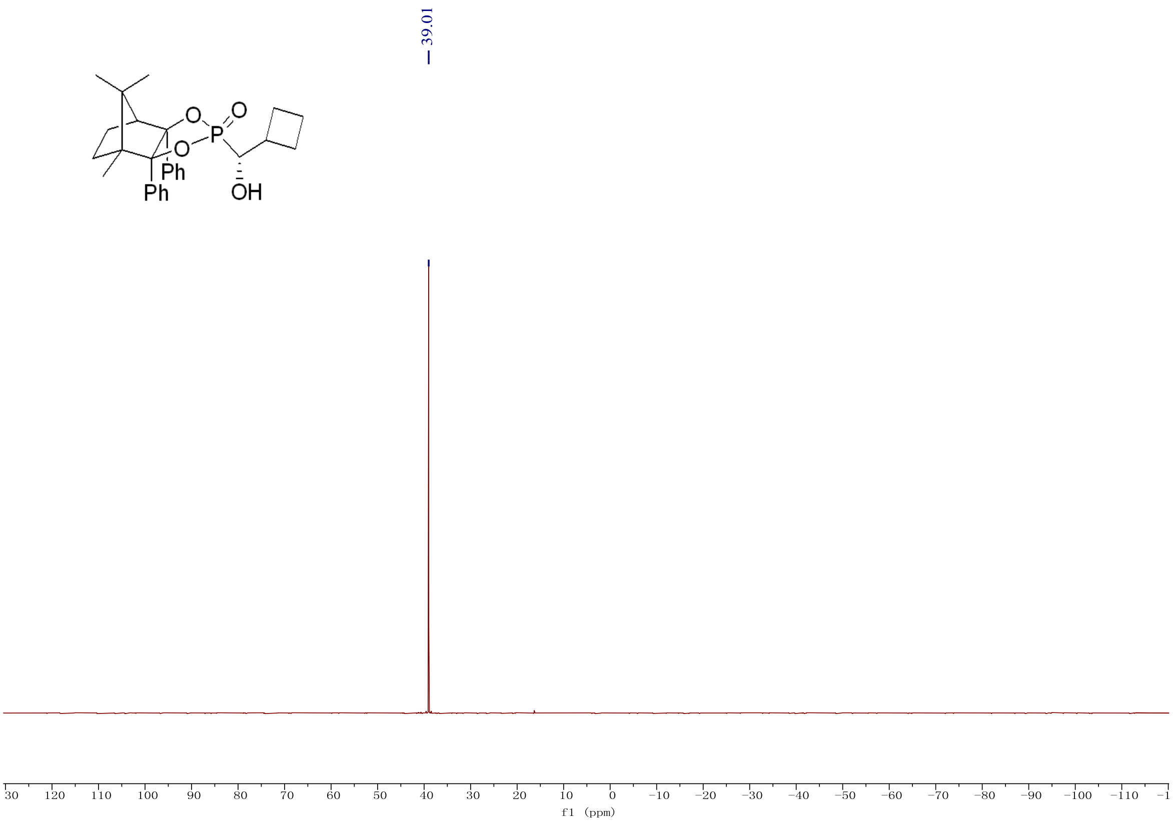

Fig. S94 ^{31}P NMR of compound **3zd**

^{1}H NMR (300 MHz, $CDCl_3$)

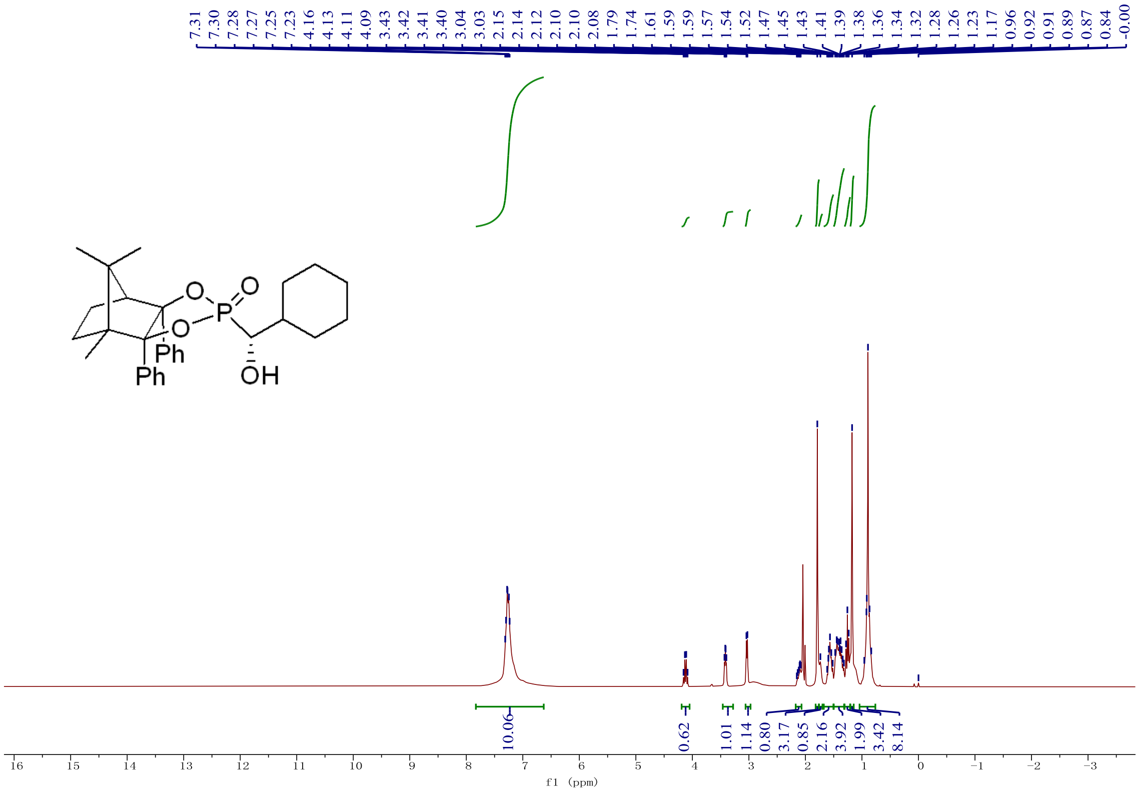

Fig. S95 ^{1}H NMR of compound **3ze**

^{13}C NMR (75 MHz, $CDCl_3$)

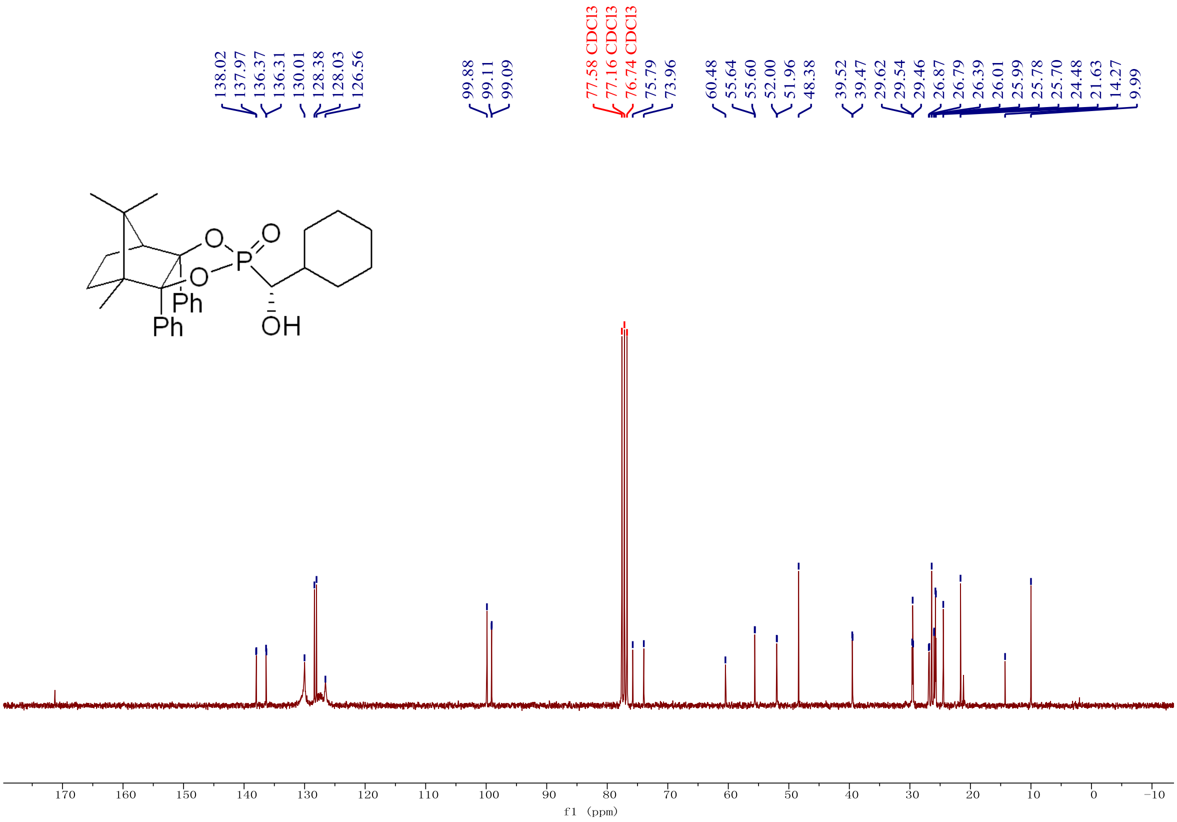

Fig. S96 ^{13}C NMR of compound **3ze**

^{31}P NMR (121 MHz, $CDCl_3$)

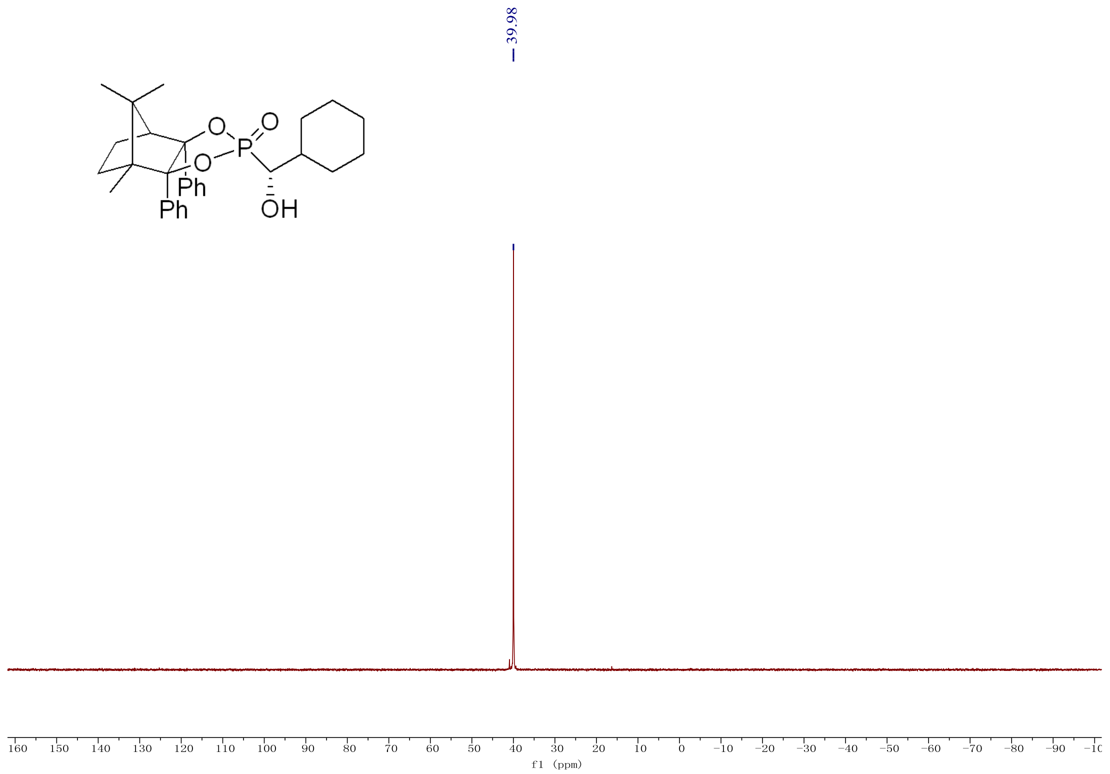

Fig. S97 ^{31}P NMR of compound **3ze**

^{1}H NMR (300 MHz, $CDCl_3$)

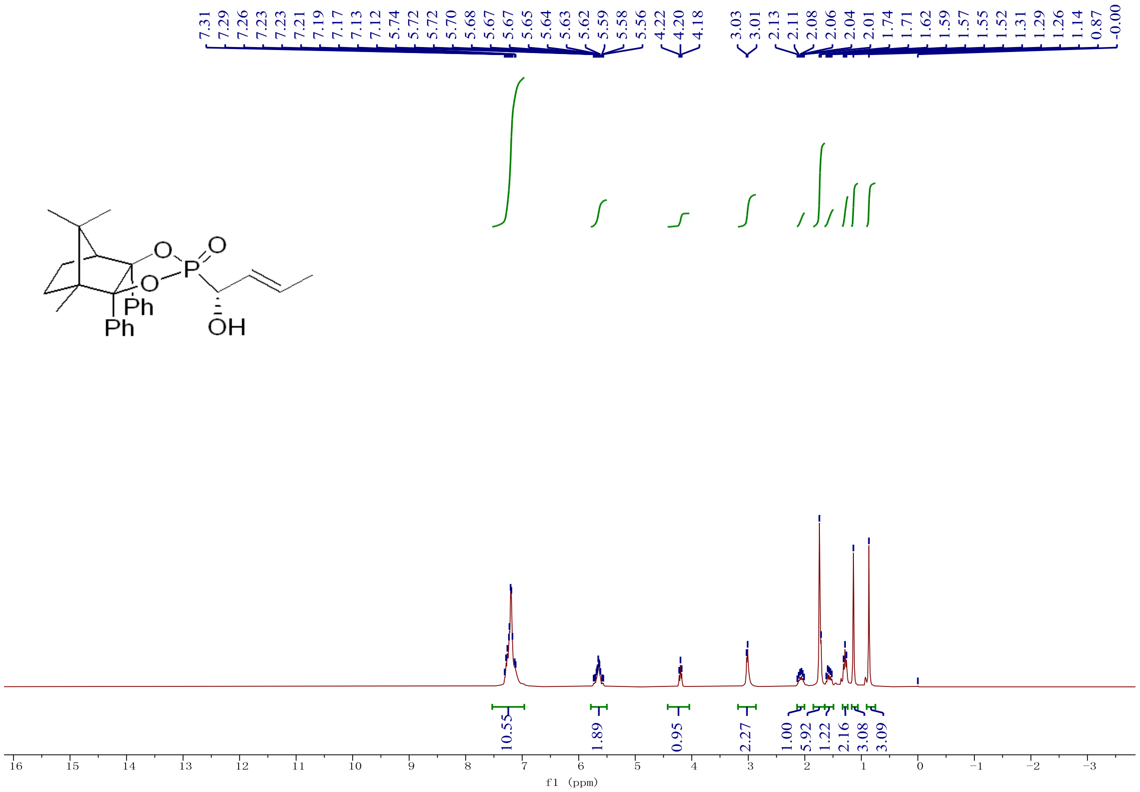

Fig. S98 ^{1}H NMR of compound **3zf**

^{13}C NMR (75 MHz, $CDCl_3$)

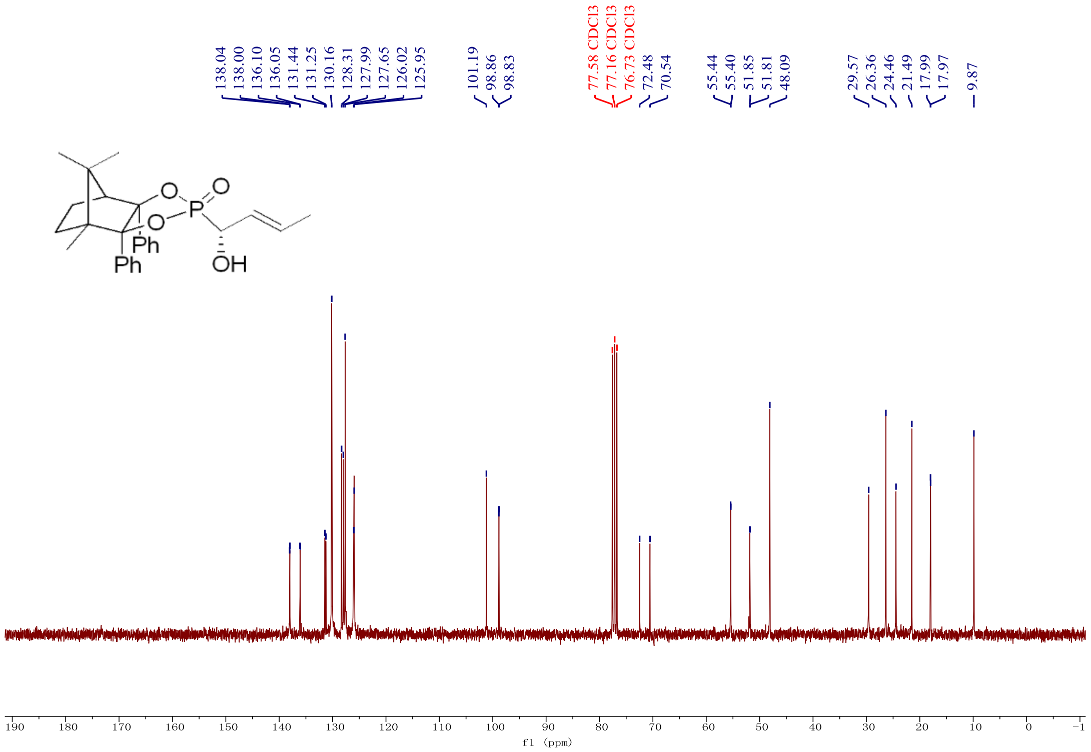

Fig. S99 ^{13}C NMR of compound **3zf**

^{31}P NMR (121 MHz, $CDCl_3$)

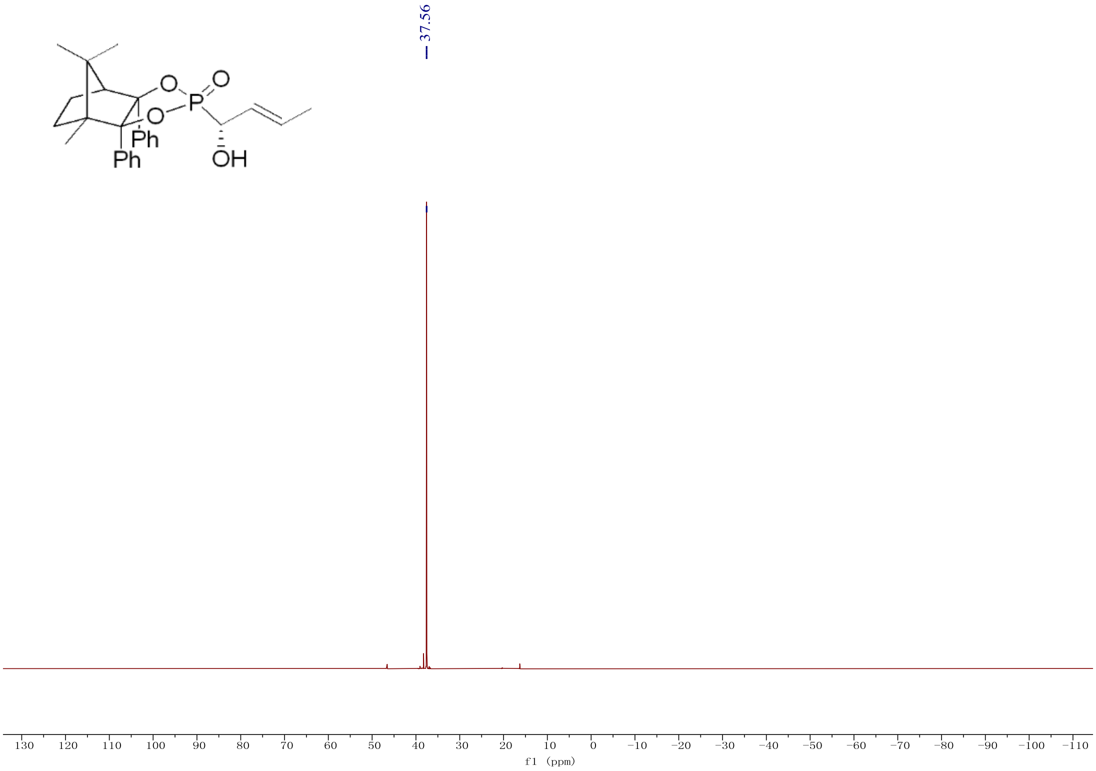

Fig. S100 ^{31}P NMR of compound **3zf**

^{1}H NMR (300 MHz, $CDCl_3$)

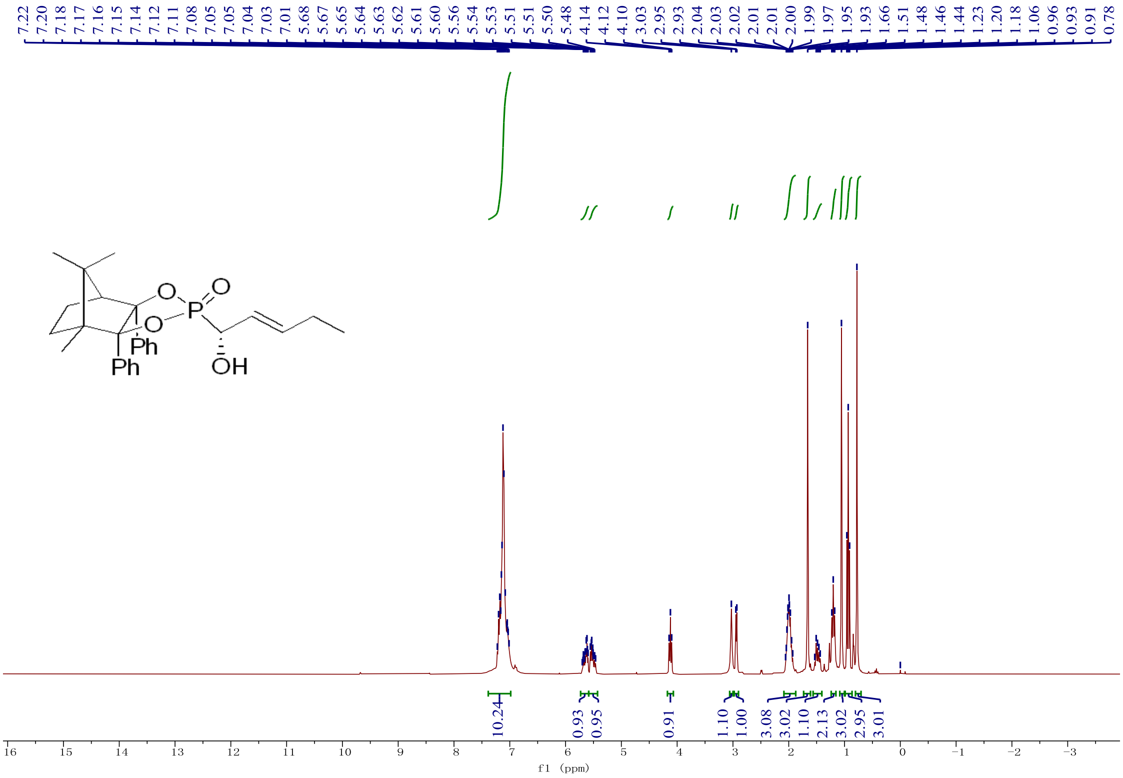

Fig. S101 ^{1}H NMR of compound **3zg**

^{13}C NMR (75 MHz, $CDCl_3$)

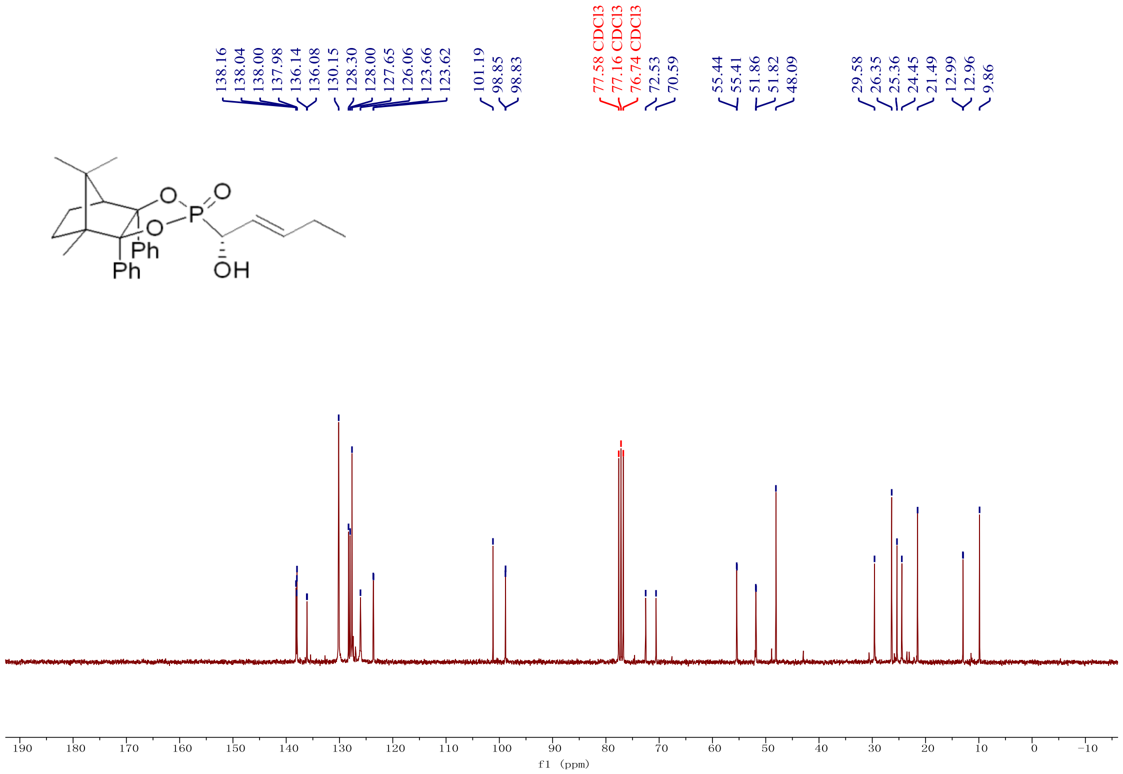

Fig. S102 ^{13}C NMR of compound **3zg**

^{31}P NMR (121 MHz, $CDCl_3$)

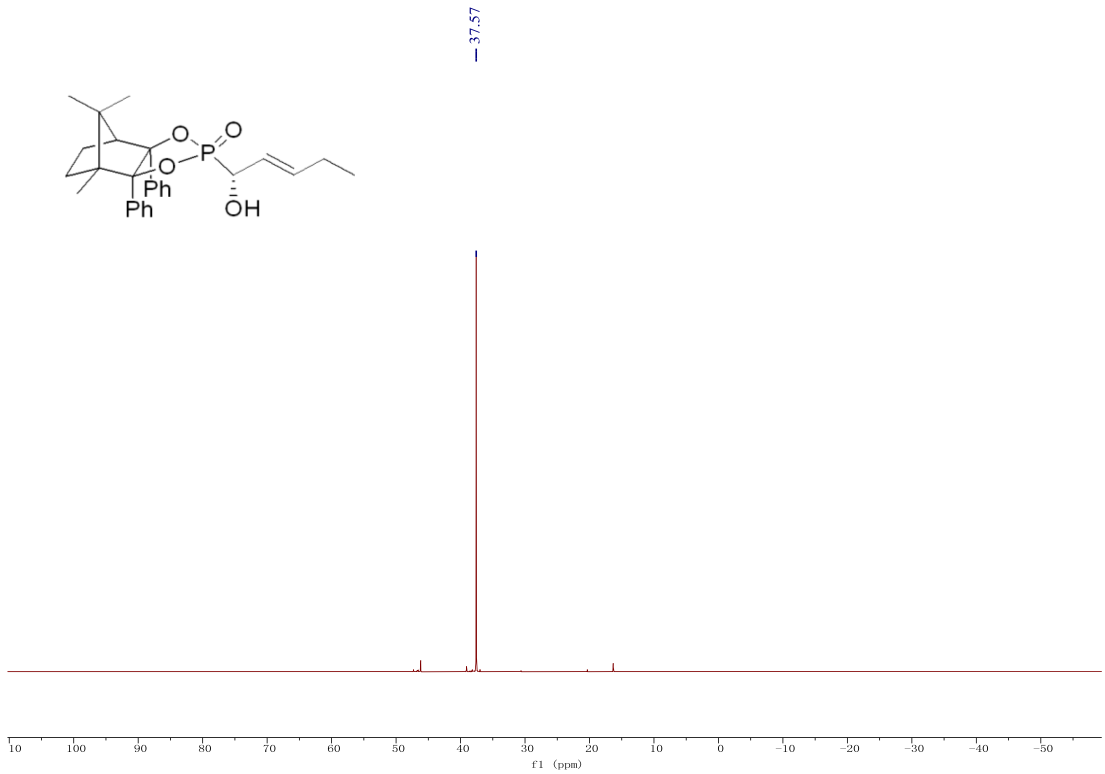

Fig. S103 ^{31}P NMR of compound **3zg**

^{1}H NMR (300 MHz, D_2O)

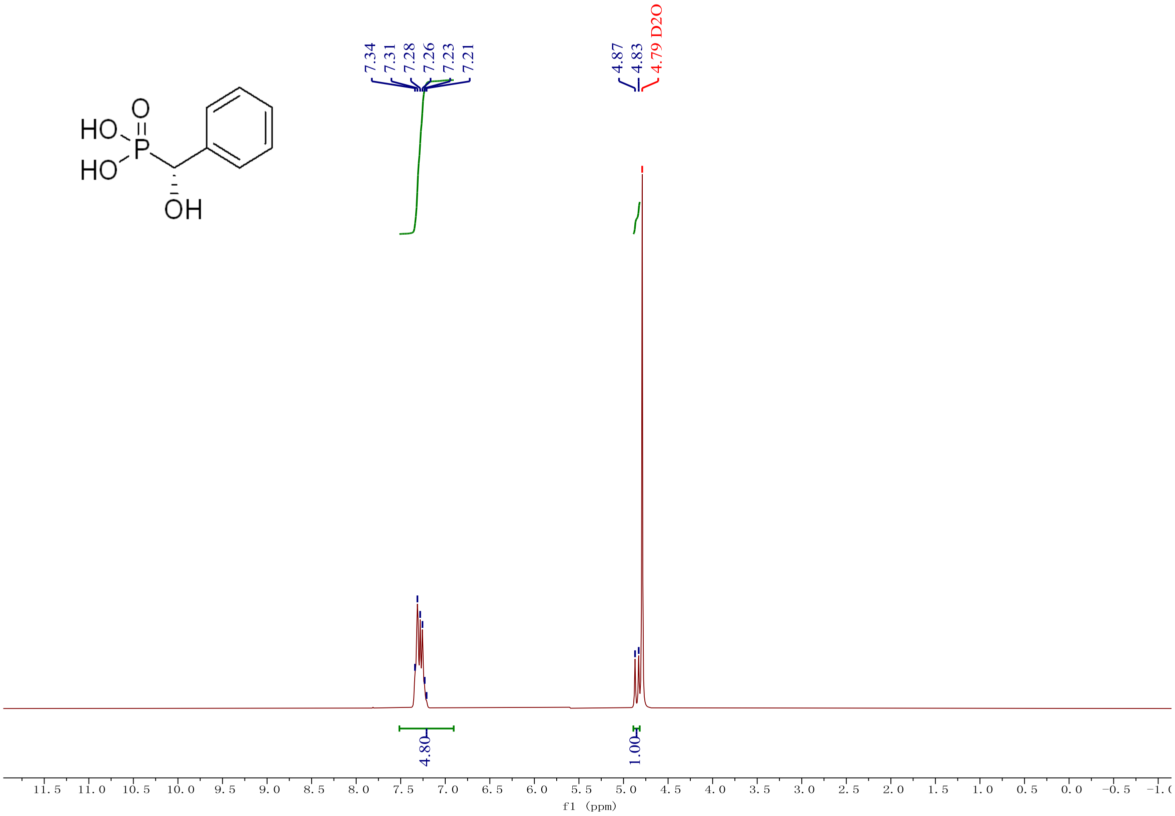

Fig. S104 ^{1}H NMR of compound **4a**

^{13}C NMR (75 MHz, D_2O)

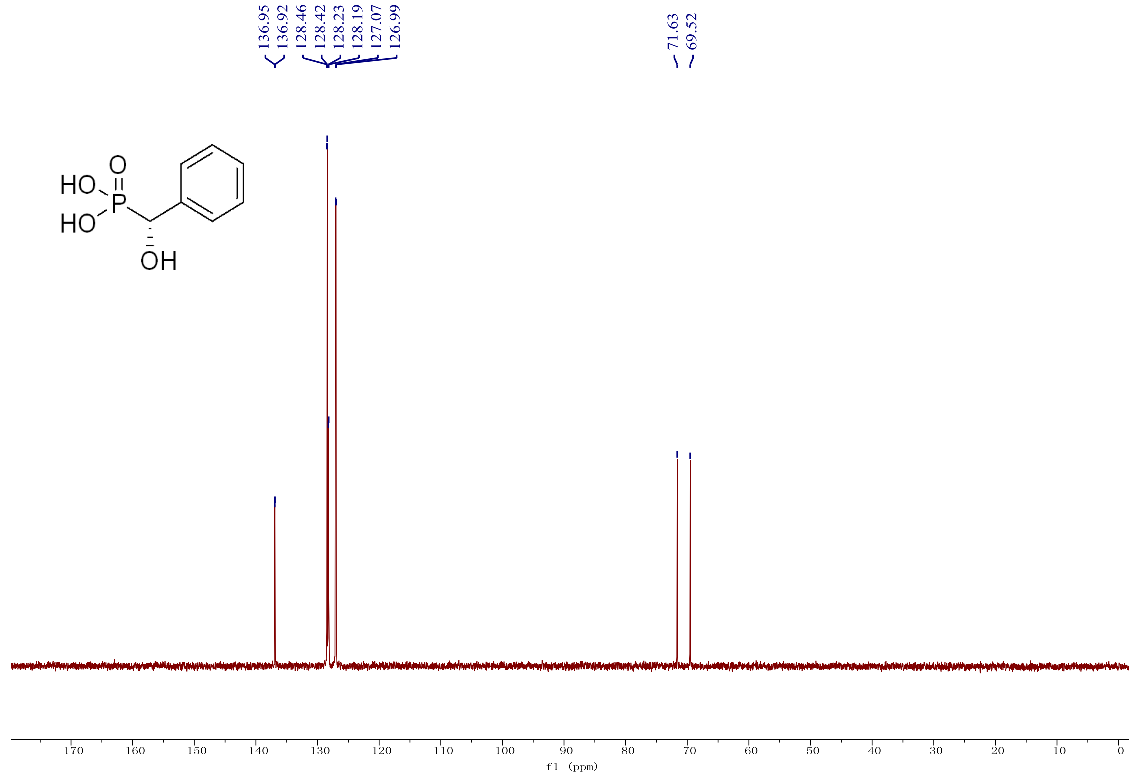

Fig. S105 ^{13}C NMR of compound **4a**

^{31}P NMR (121 MHz, D_2O)

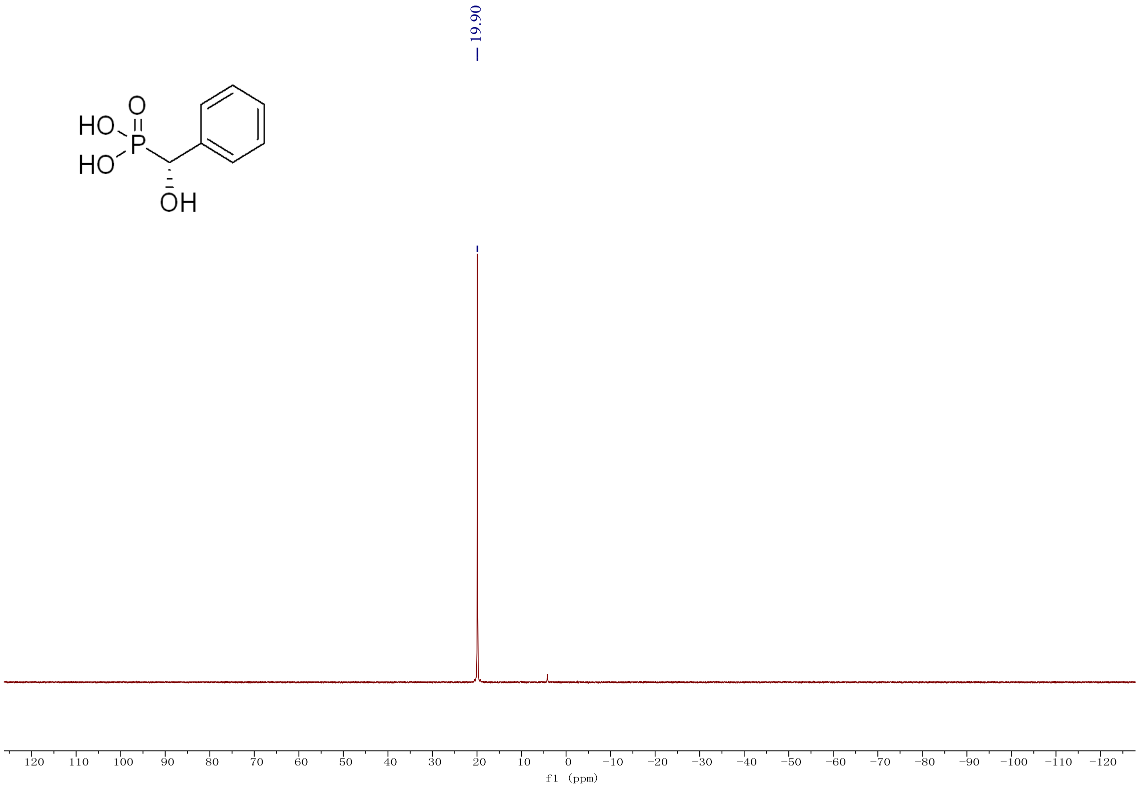

Fig. S106 ^{31}P NMR of compound **4a**

^{1}H NMR (300 MHz, $CDCl_3$)

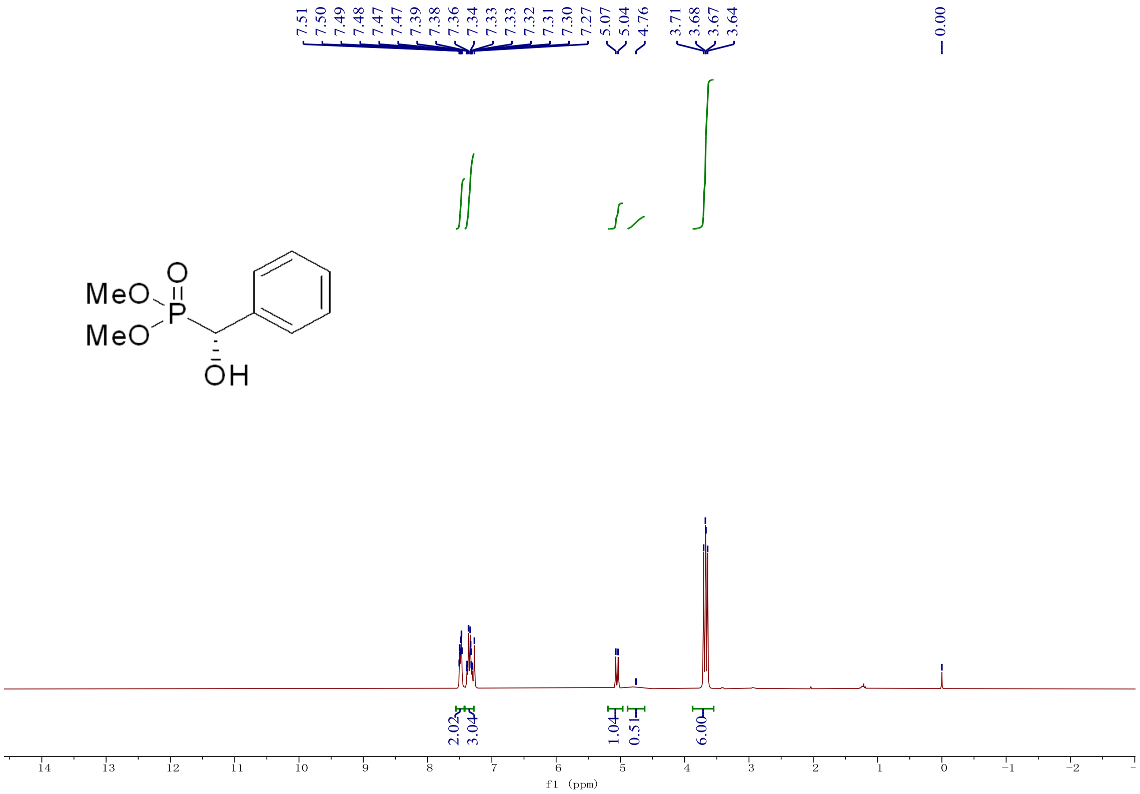

Fig. S107 ^{1}H NMR of compound **4b**

^{13}C NMR (75 MHz, $CDCl_3$)

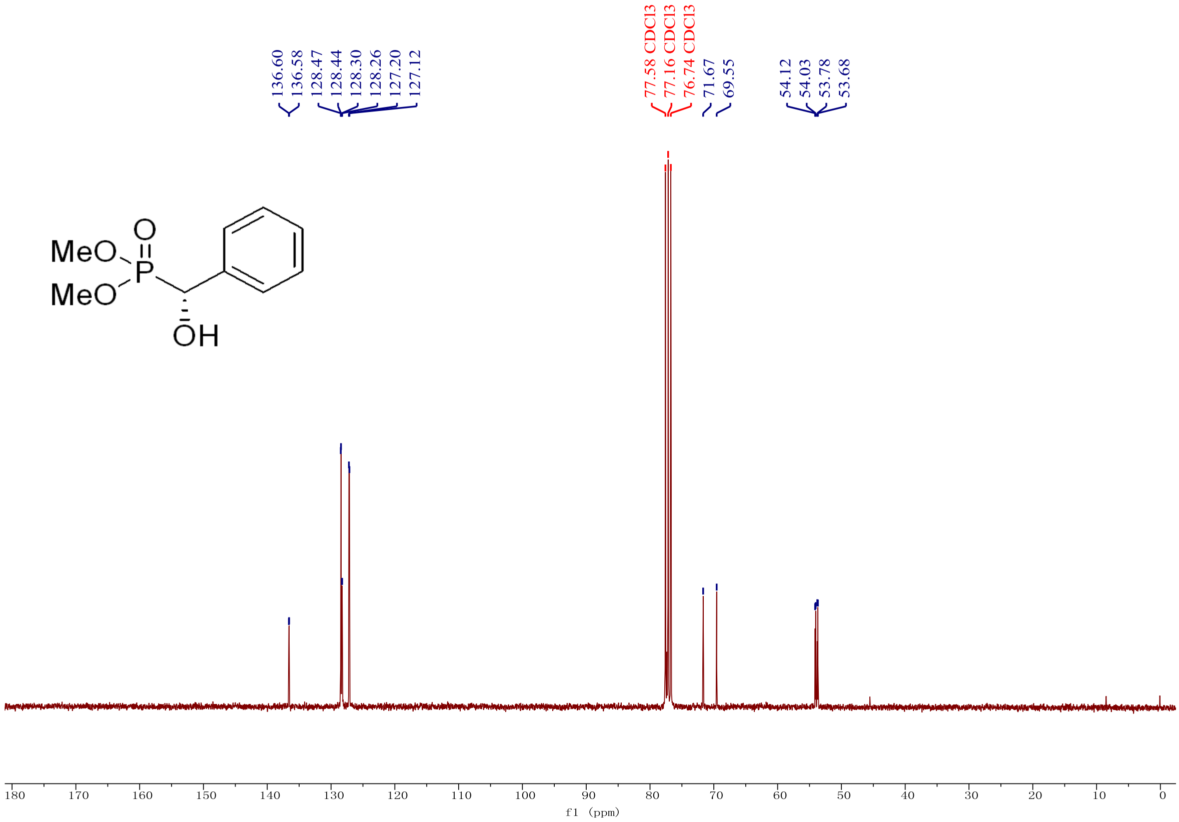

Fig. S108 ^{13}C NMR of compound **4b**

^{31}P NMR (121 MHz, $CDCl_3$)

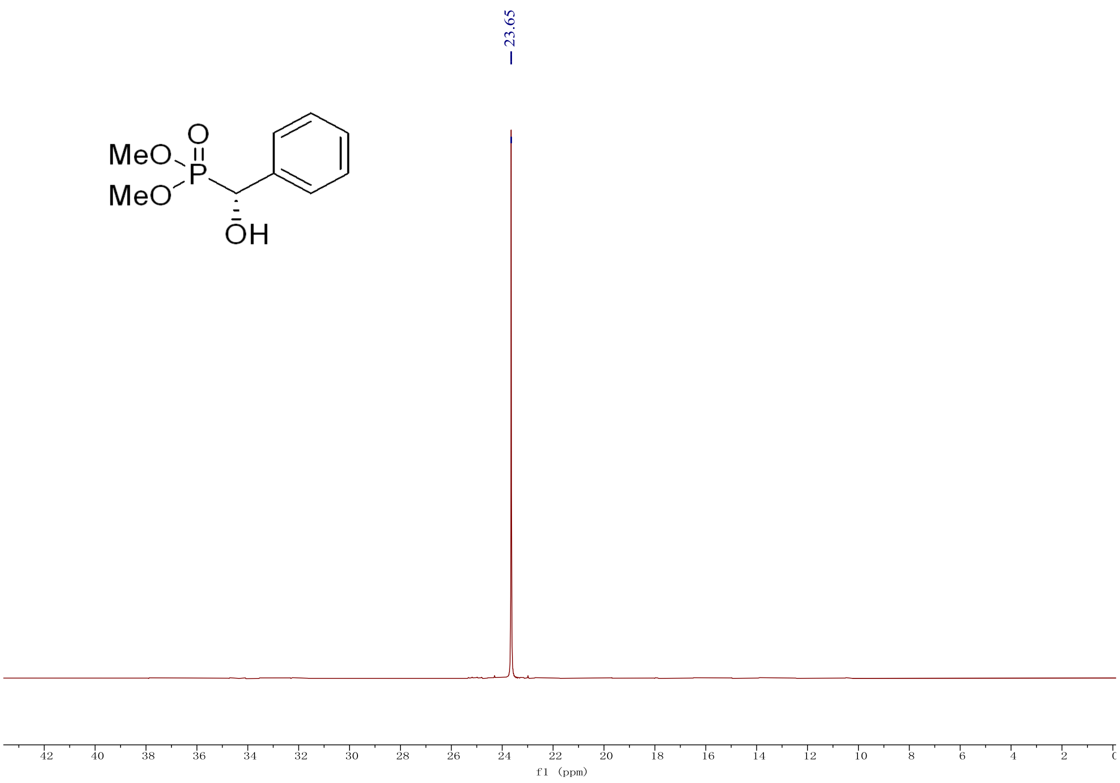

Fig. S109 ^{31}P NMR of compound **4b**

^{1}H NMR (300 MHz, MeOD-d_4)

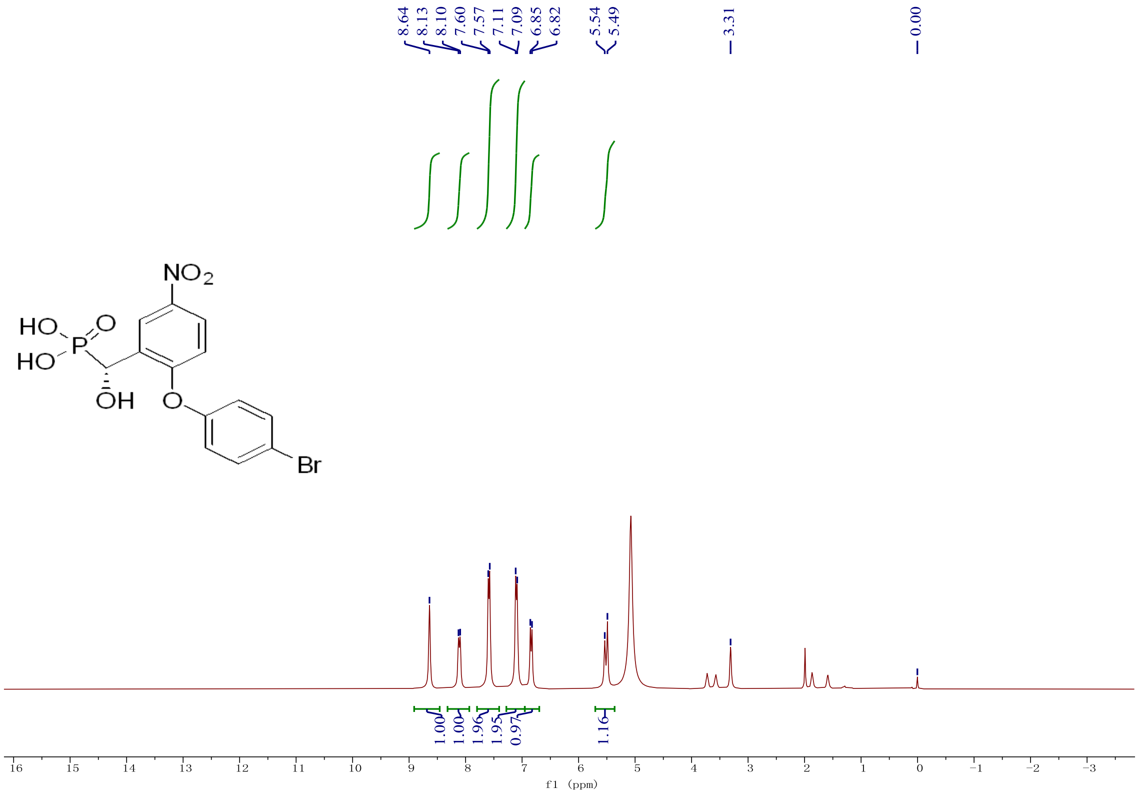

Fig. S110 ^{1}H NMR of compound **4c**

^{13}C NMR (75 MHz, MeOD-d_4)

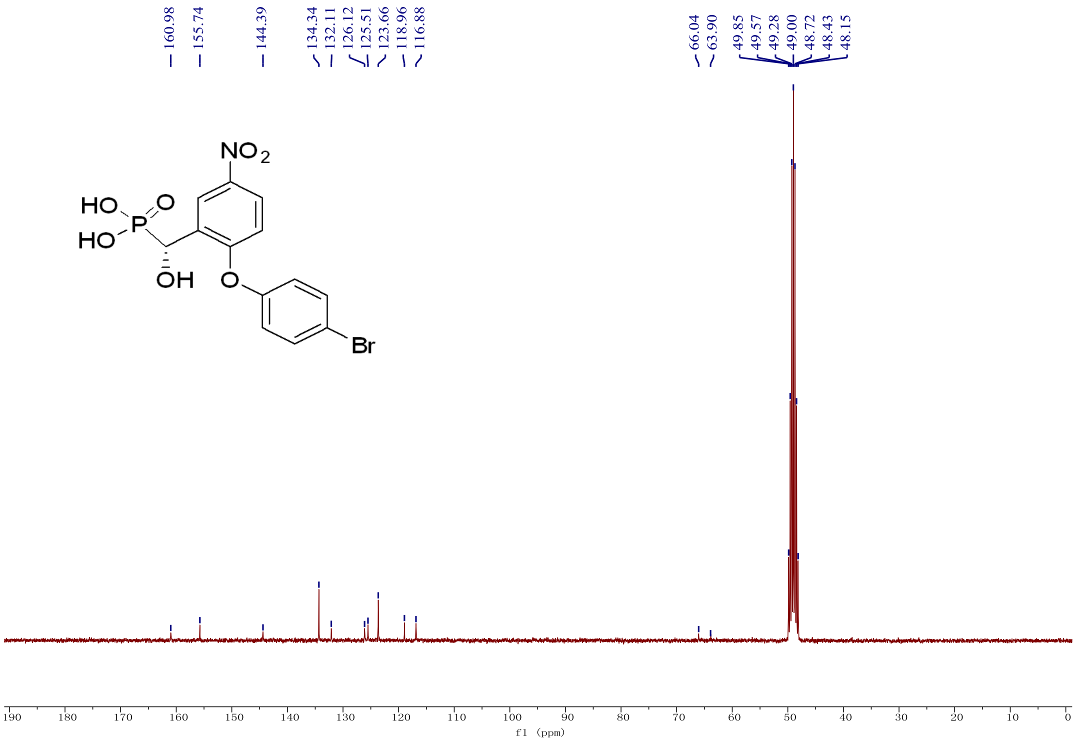

Fig. S111 ^{13}C NMR of compound **4c**

^{31}P NMR (121 MHz, MeOD-d_4)

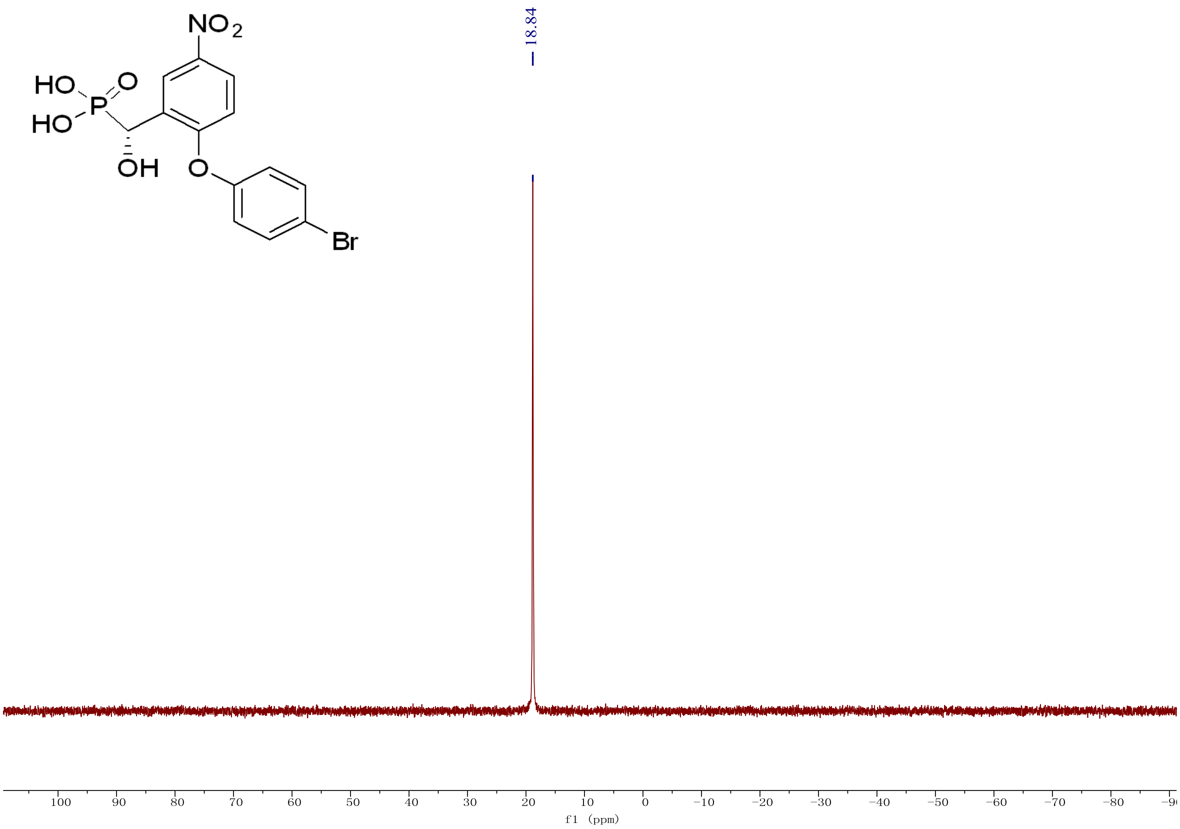

Fig. S112 ^{31}P NMR of compound **4c**

^{1}H NMR (300 MHz, MeOD-d_4)

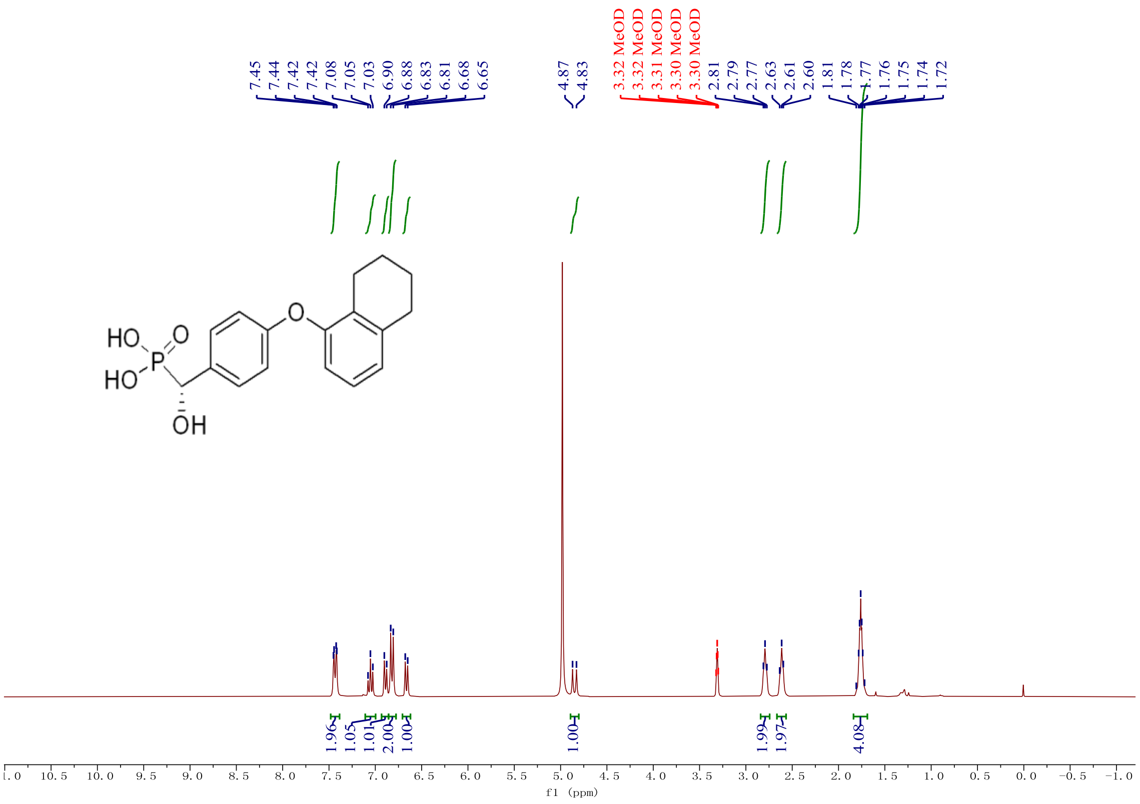

Fig. S113 ^{1}H NMR of compound **4d**

^{13}C NMR (75 MHz, MeOD-d_4)

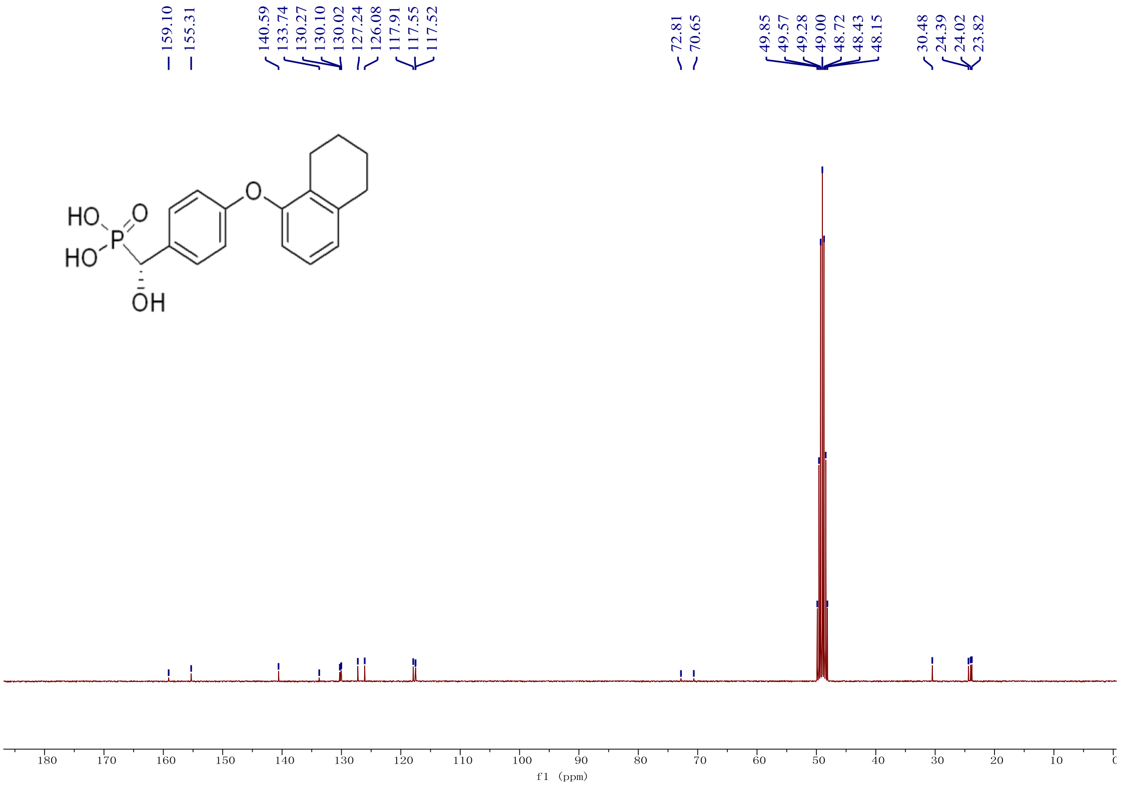

Fig. S114 ^{13}C NMR of compound **4d**

^{31}P NMR (121 MHz, MeOD-d_4)

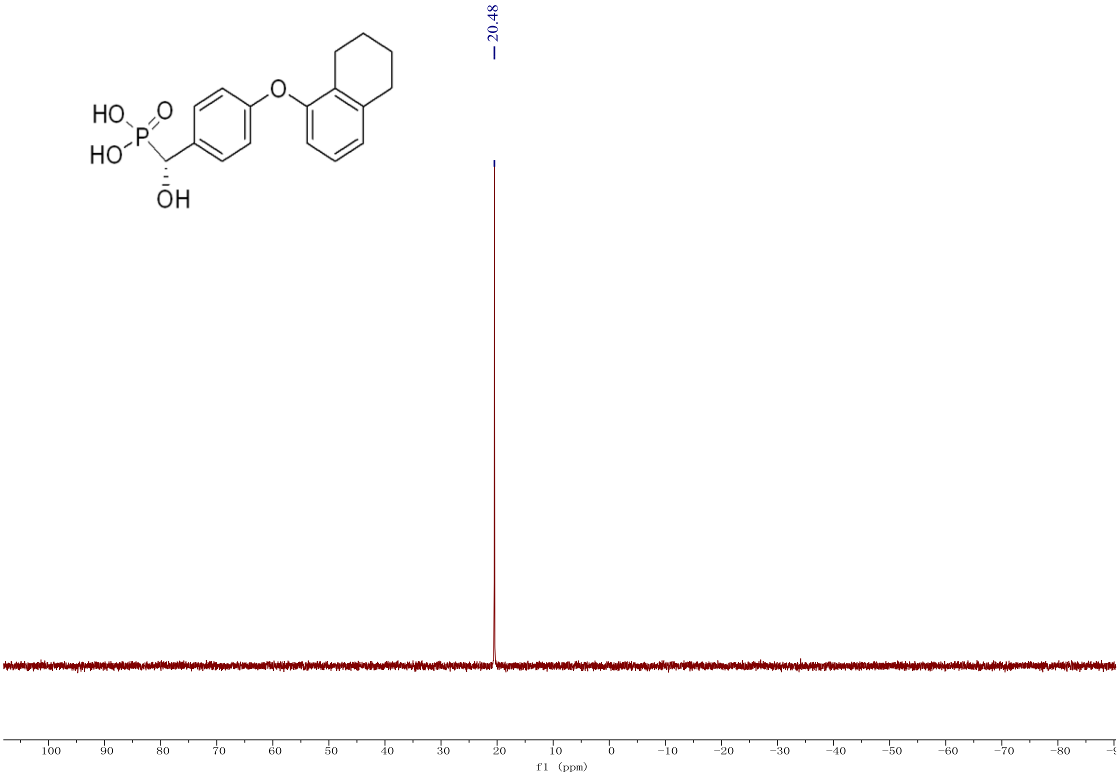

Fig. S115 ^{31}P NMR of compound **4d**

^{1}H NMR (300 MHz, $CDCl_3$)

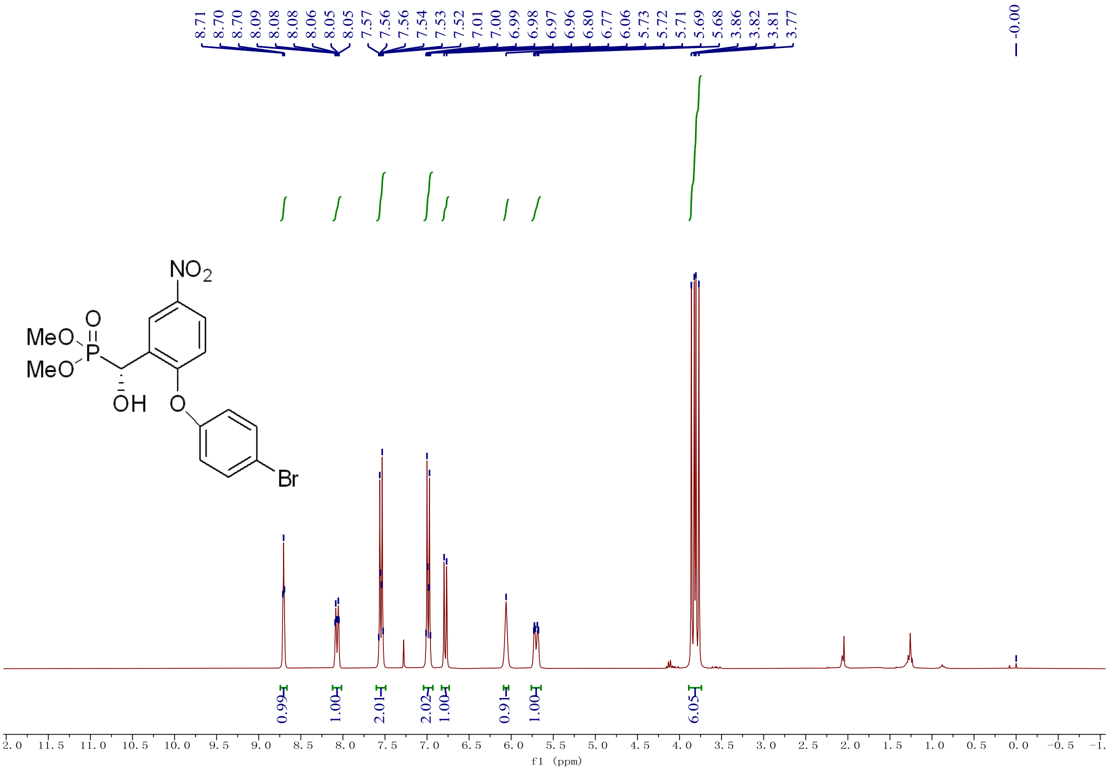

Fig. S116 ^{1}H NMR of compound **4ca**

^{13}C NMR (75 MHz, $CDCl_3$)

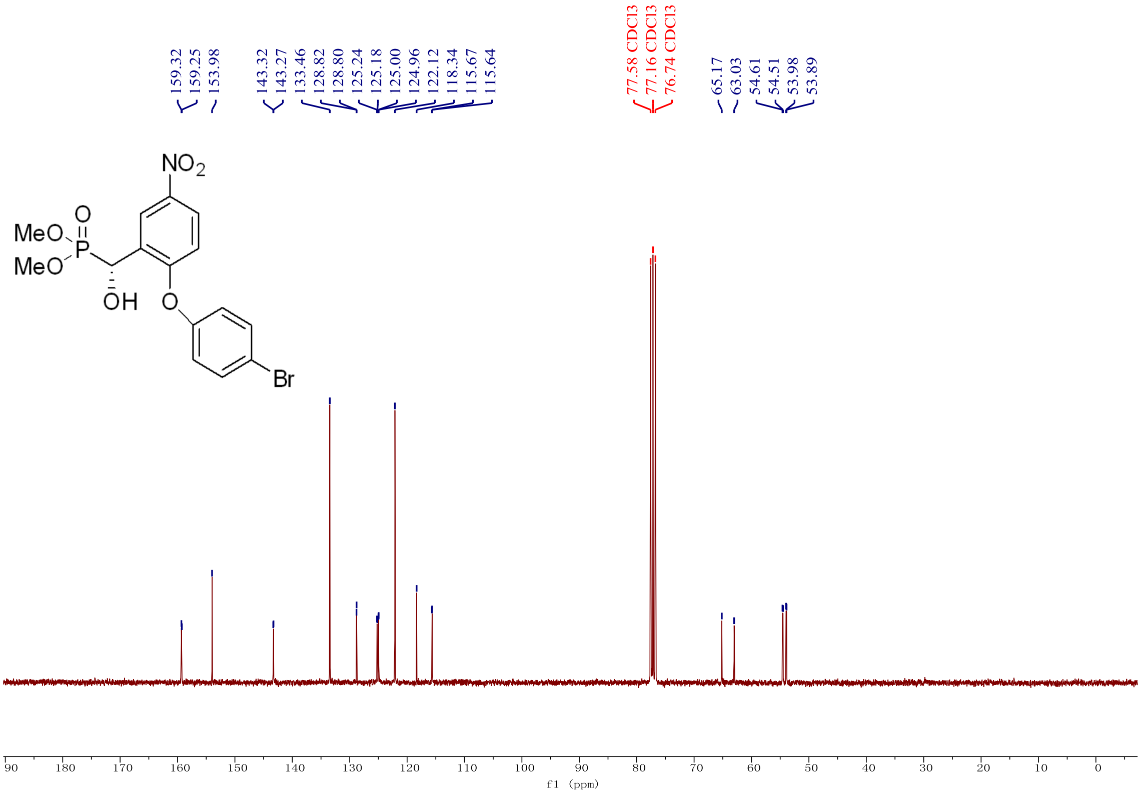

Fig. S117 ^{13}C NMR of compound **4ca**

^{31}P NMR (121 MHz, $CDCl_3$)

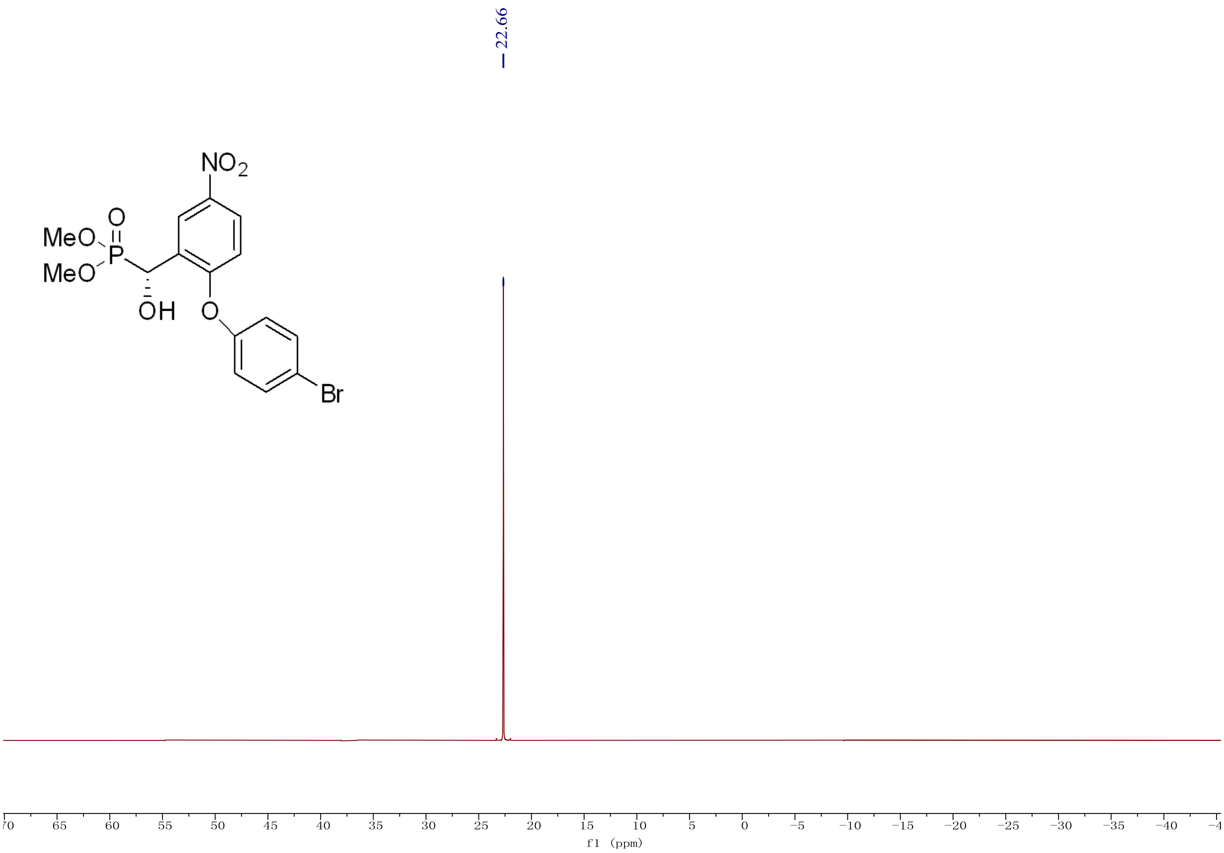

Fig. S118 ^{31}P NMR of compound **4ca**

^{1}H NMR (300 MHz, $CDCl_3$)

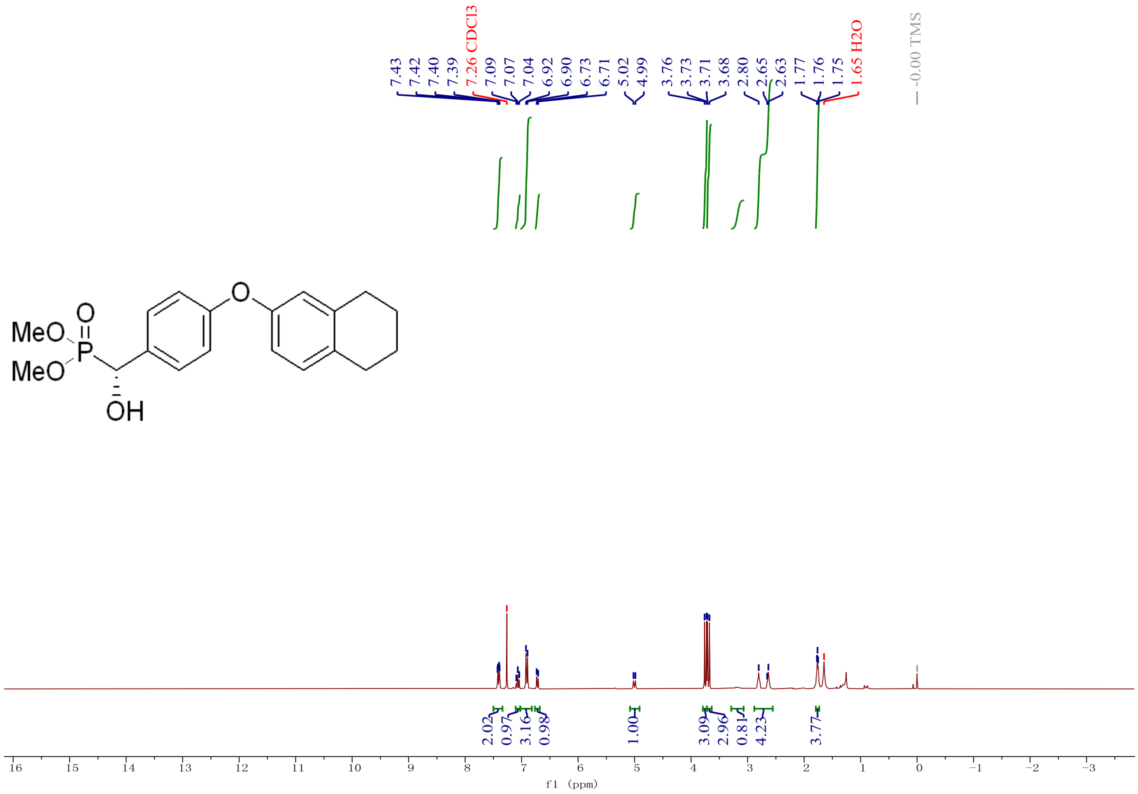

Fig. S119 ^{1}H NMR of compound **4da**

^{13}C NMR (75 MHz, $CDCl_3$)

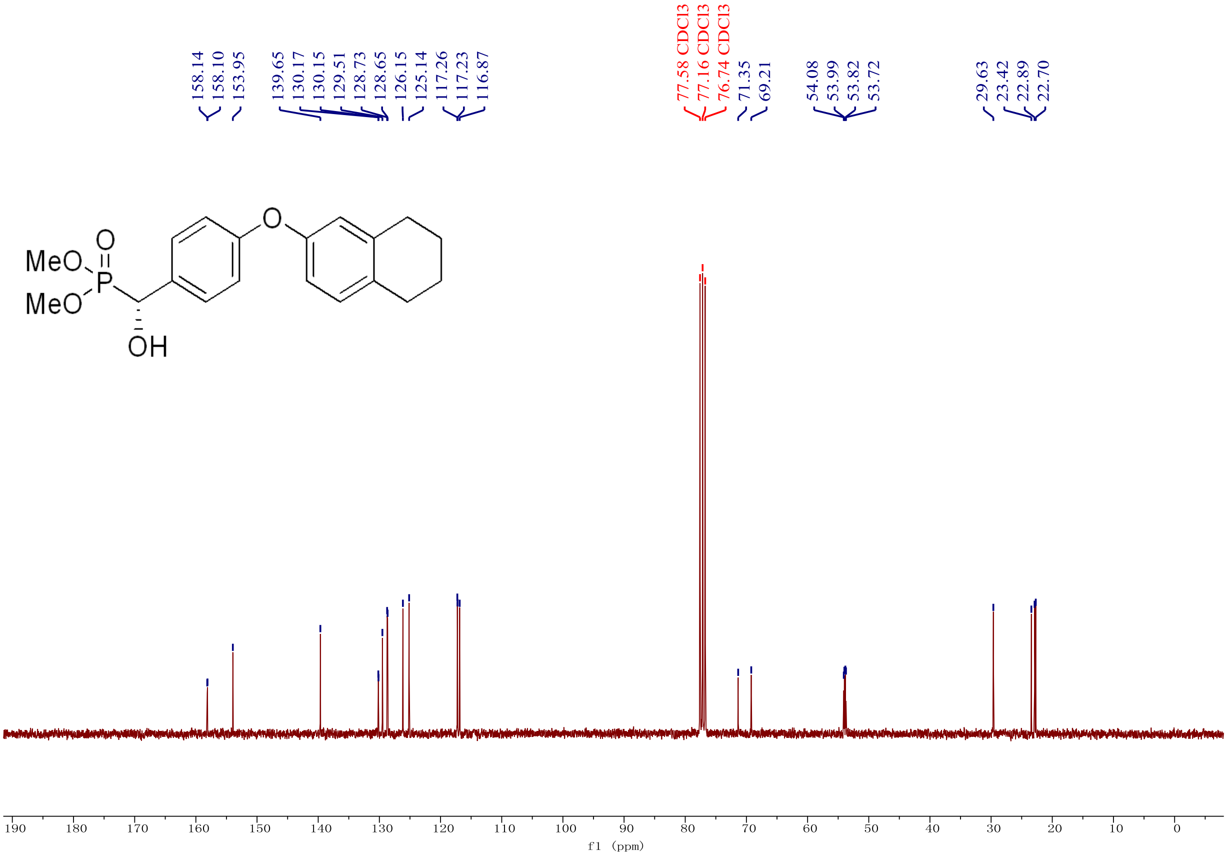

Fig. S120 ^{13}C NMR of compound **4da**

^{31}P NMR (121 MHz, $CDCl_3$)

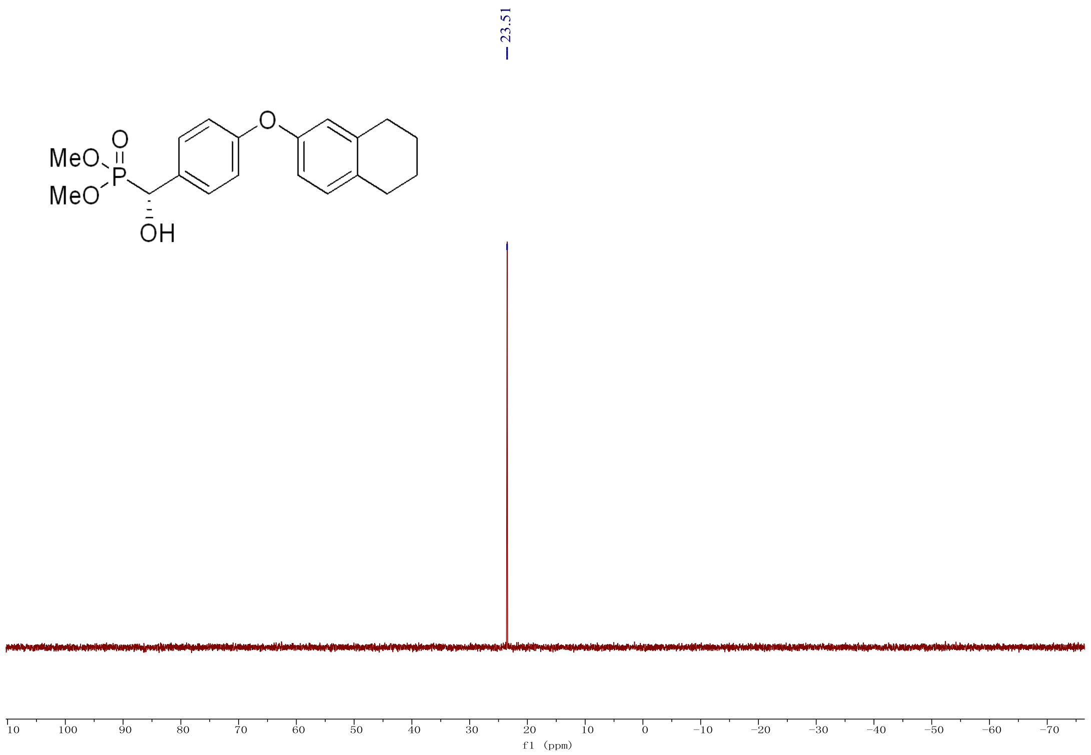

Fig. S121 ^{31}P NMR of compound **4da**

^{1}H NMR (300 MHz, $CDCl_3$)

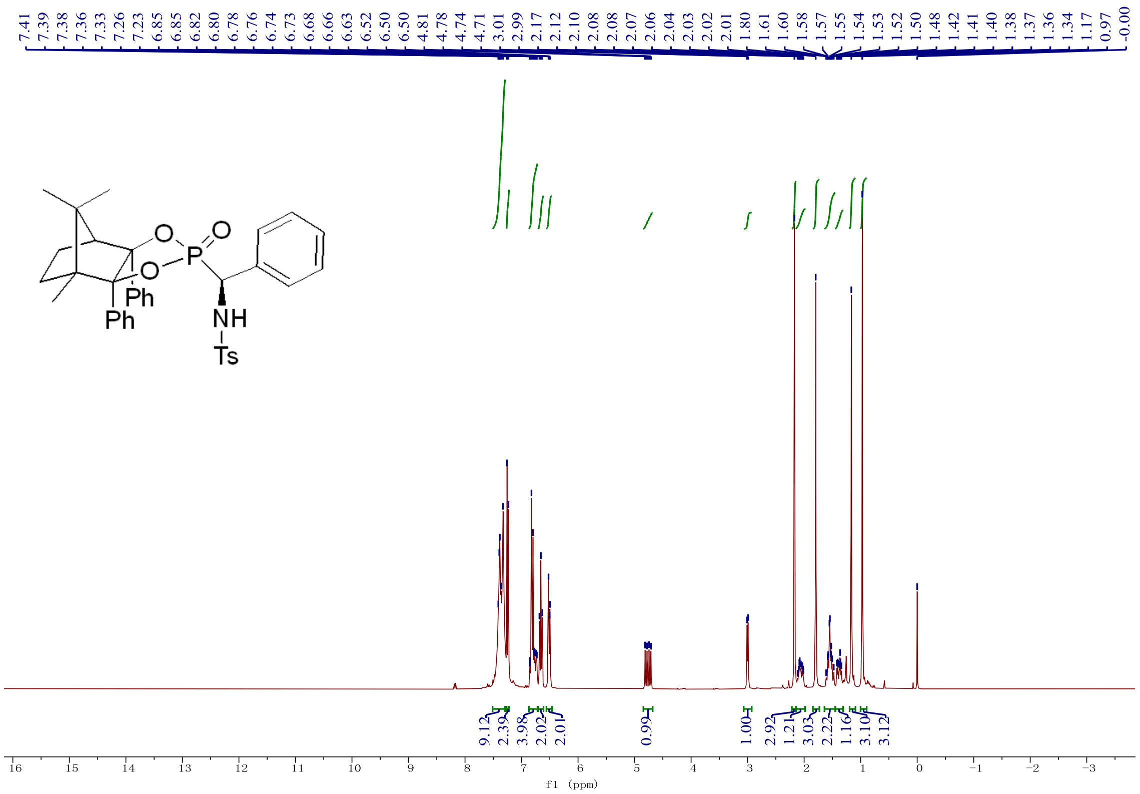

Fig. S122 ^{1}H NMR of compound **6a**

^{13}C NMR (75 MHz, $CDCl_3$)

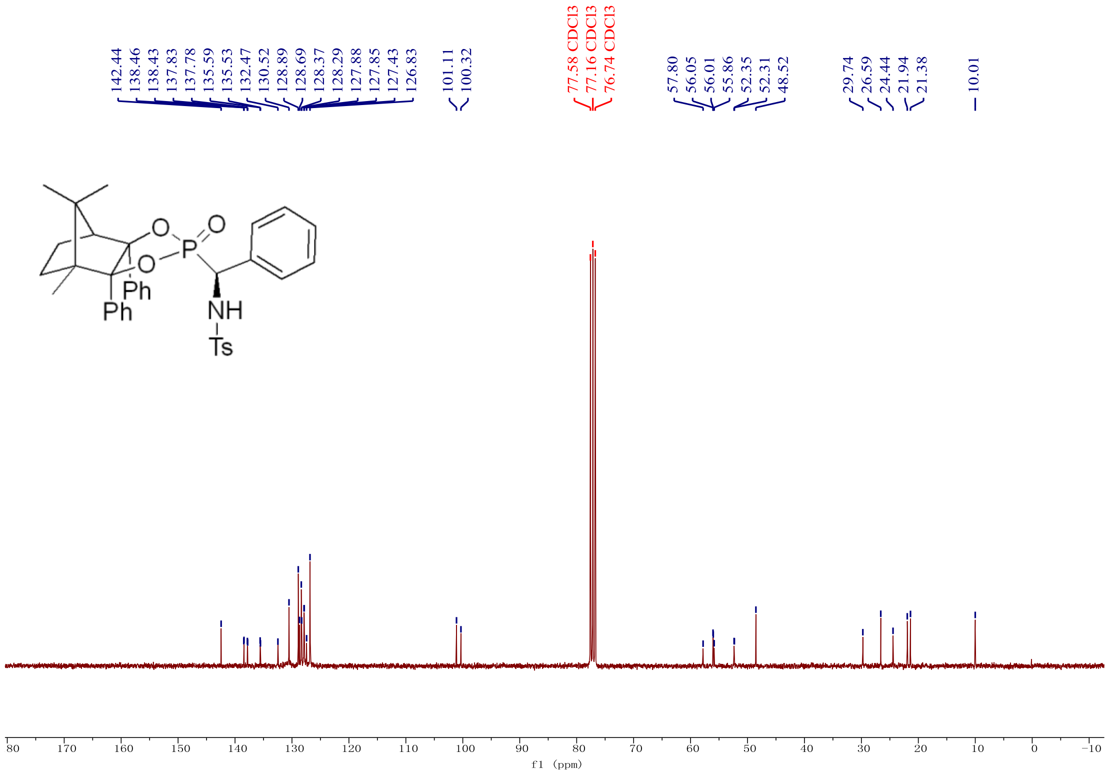

Fig. S123 ^{13}C NMR of compound **6a**

^{31}P NMR (121 MHz, $CDCl_3$)

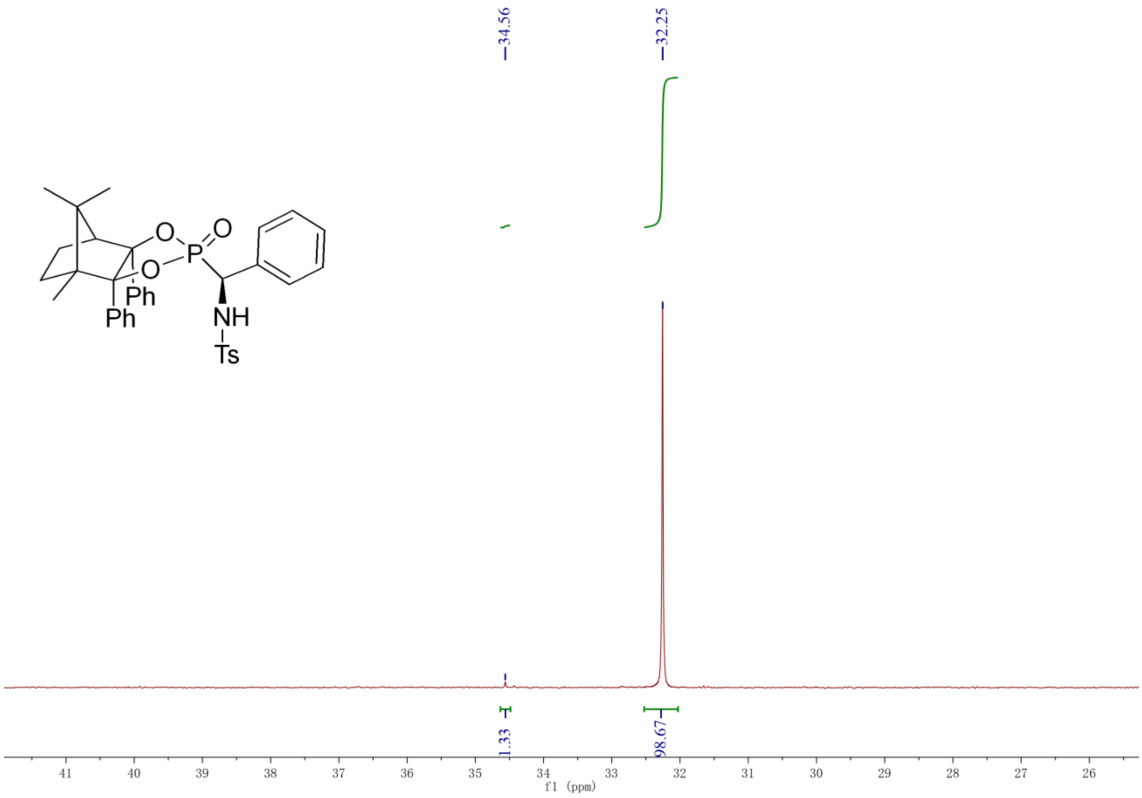

Fig. S124 ^{31}P NMR of compound **6a**

^{1}H NMR (300 MHz, $CDCl_3$)

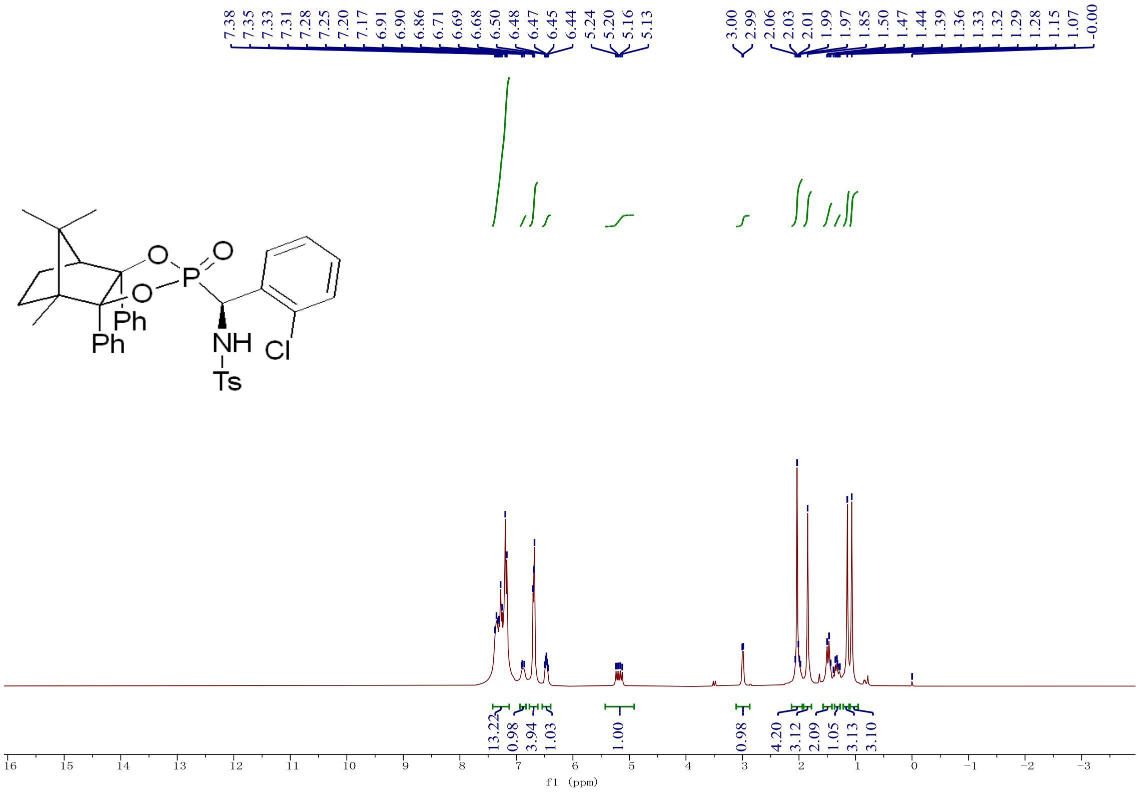

Fig. S125 ^{1}H NMR of compound **6b**

^{13}C NMR (75 MHz, $CDCl_3$)

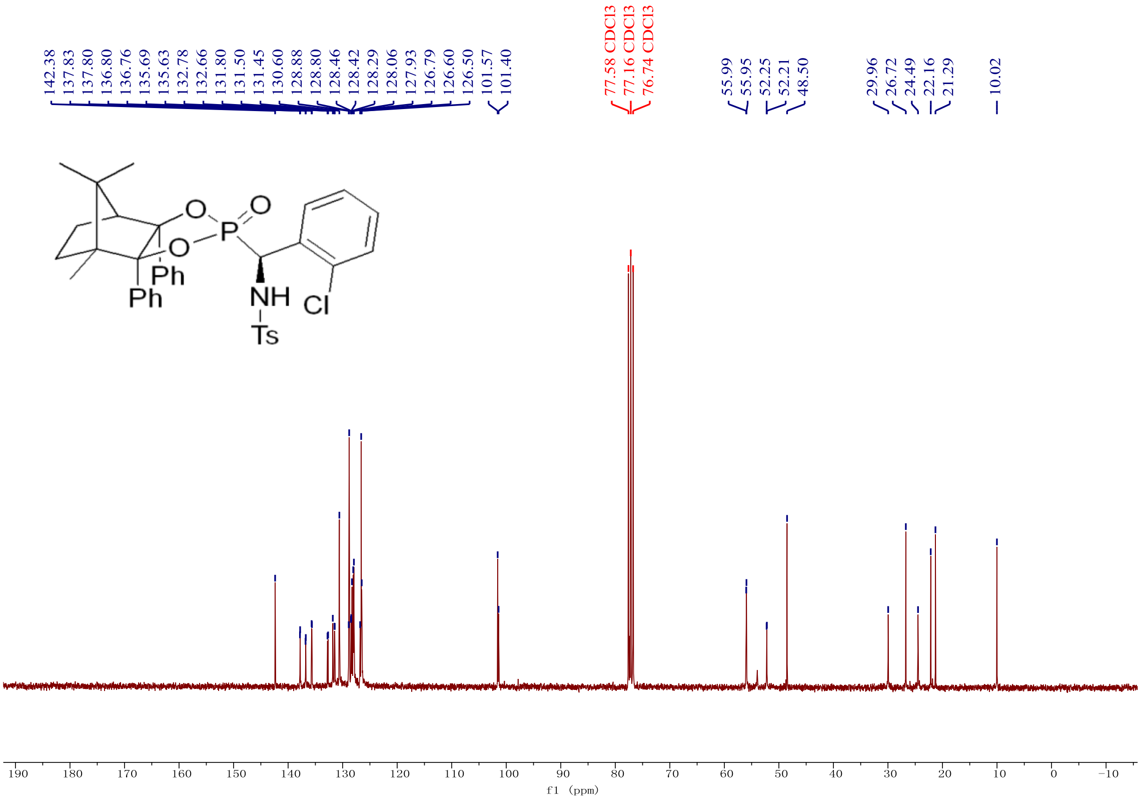

Fig. S126 ^{13}C NMR of compound **6b**

^{31}P NMR (121 MHz, $CDCl_3$)

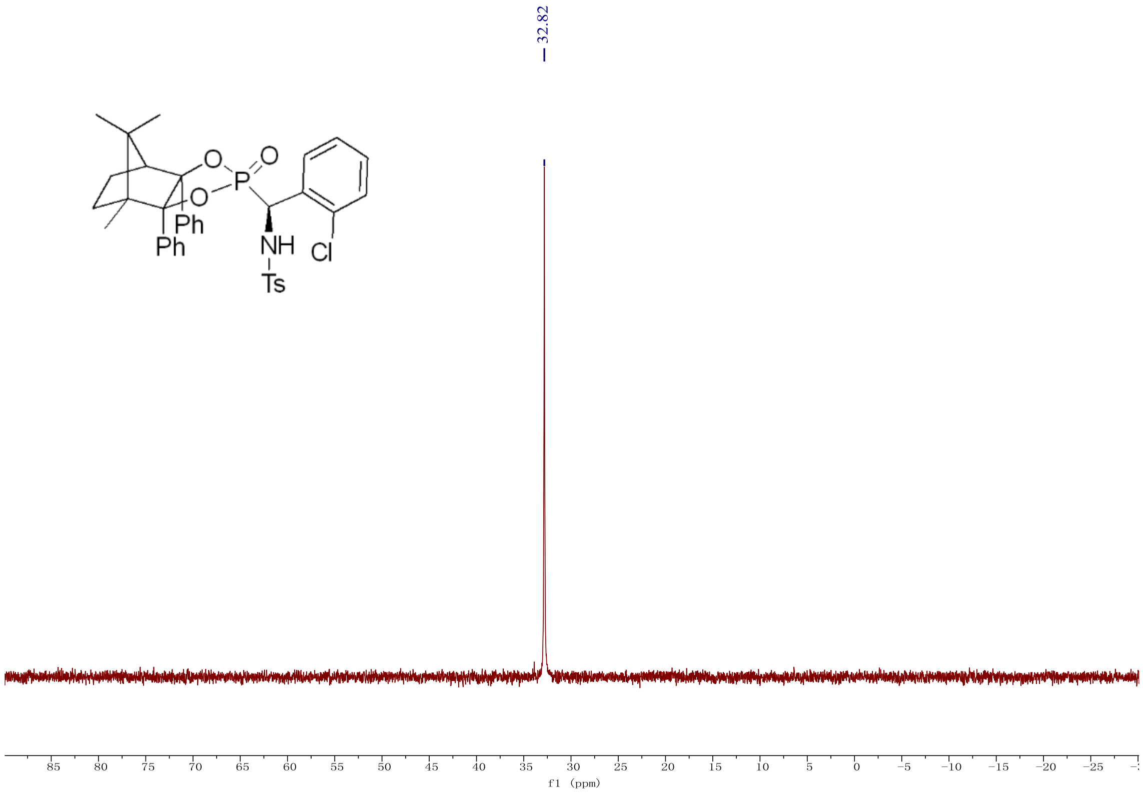

Fig. S127 ^{31}P NMR of compound **6b**

^{1}H NMR (300 MHz, $CDCl_3$)

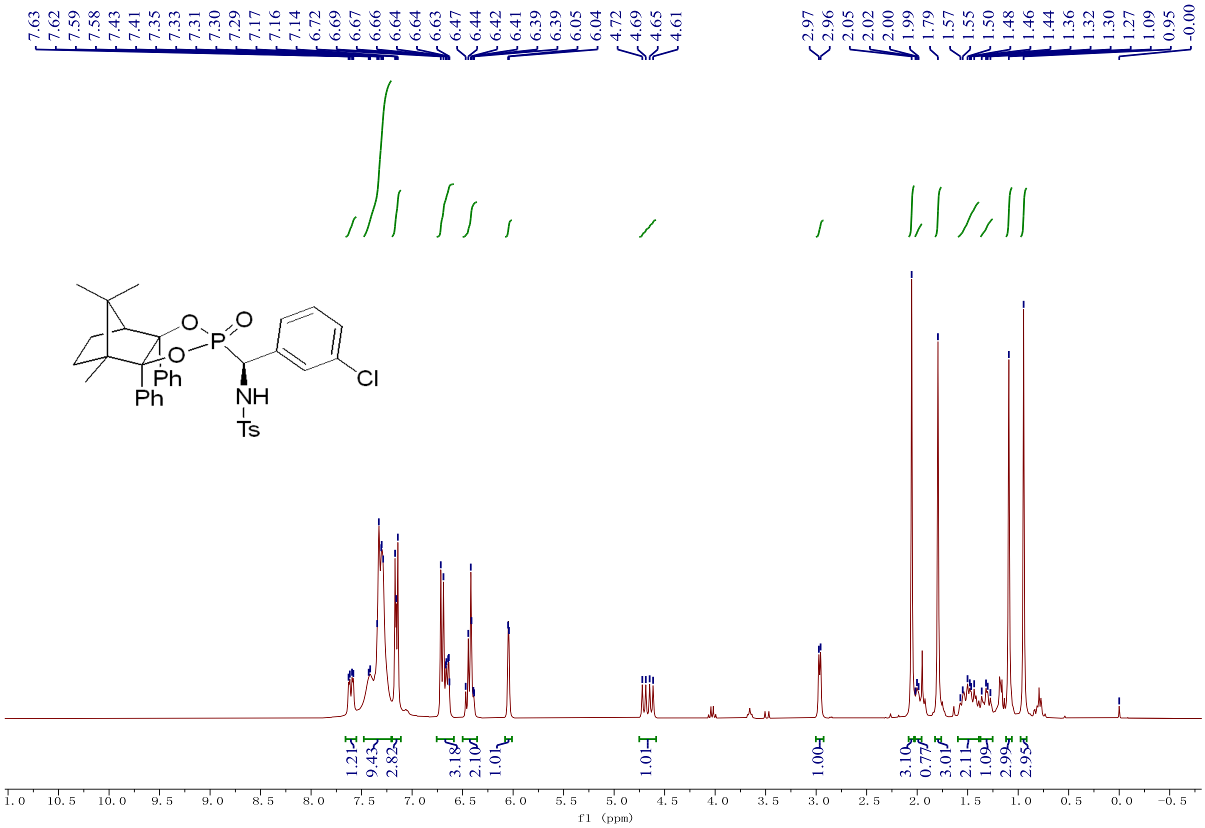

Fig. S128 ^{1}H NMR of compound **6c**

^{13}C NMR (75 MHz, $CDCl_3$)

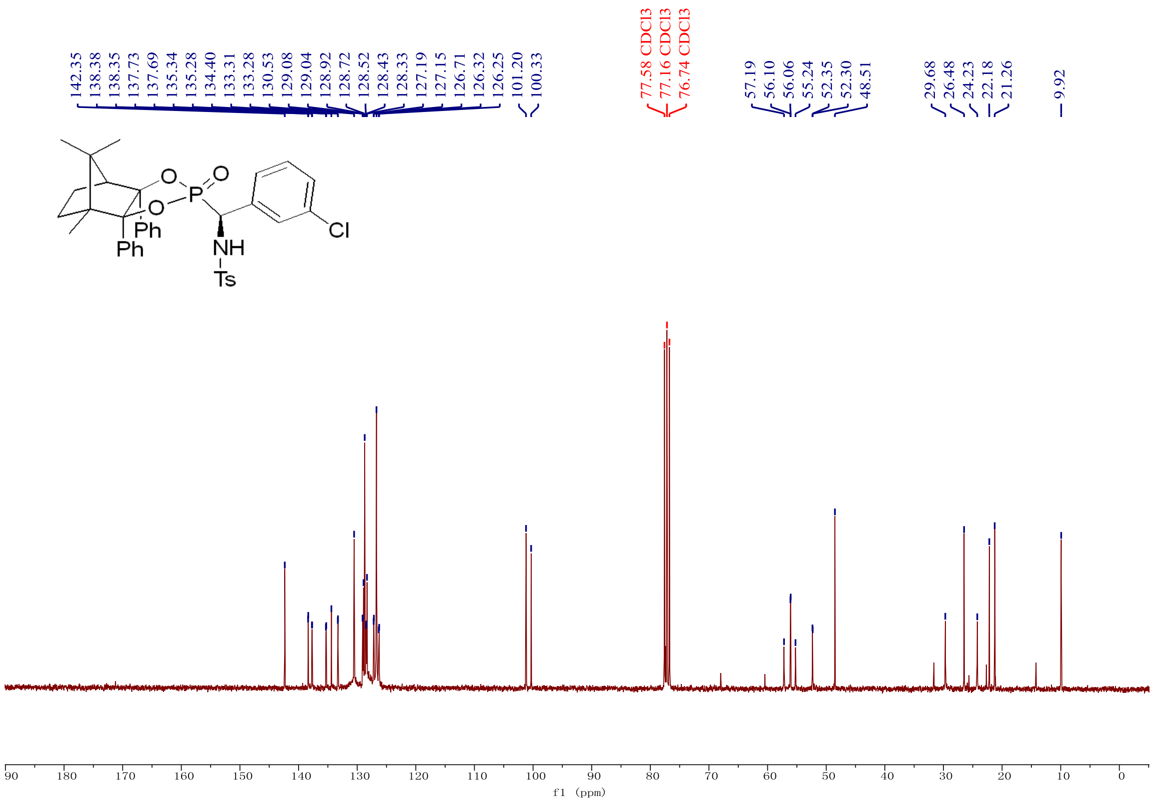

Fig. S129 ^{13}C NMR of compound **6c**

^{31}P NMR (121 MHz, $CDCl_3$)

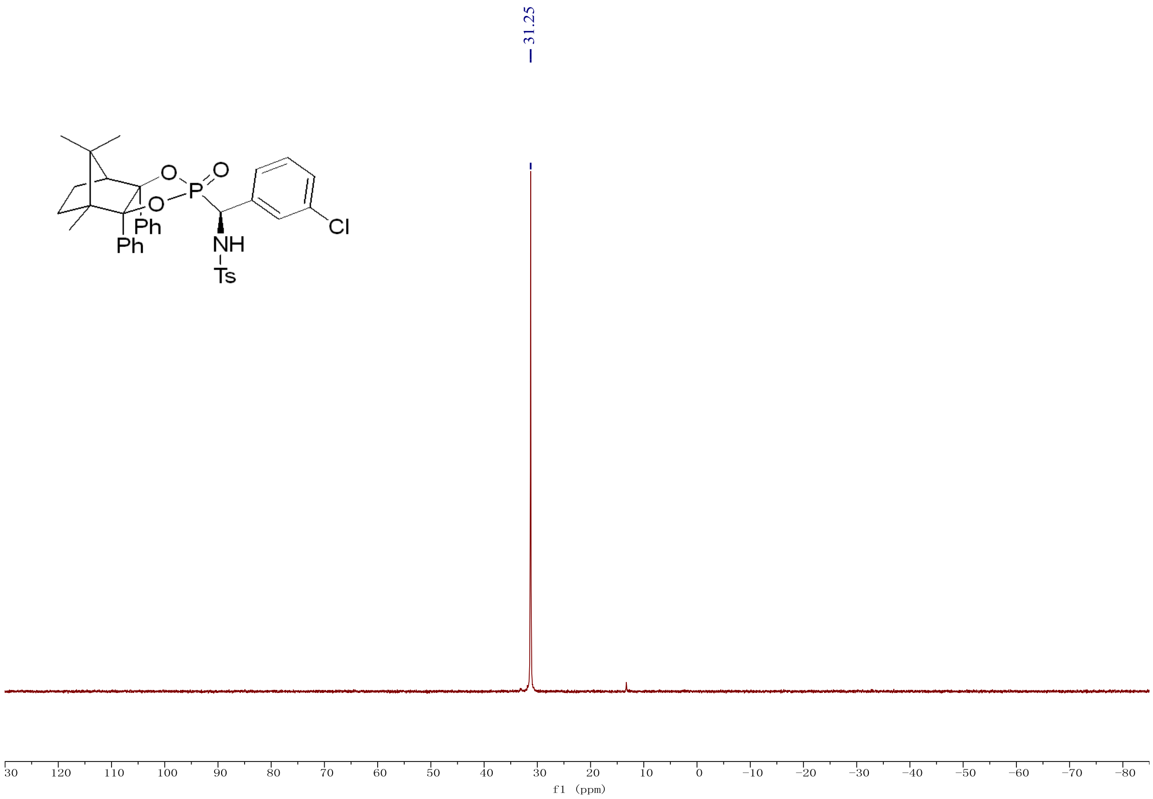

Fig. S130 ^{31}P NMR of compound **6c**

^{1}H NMR (300 MHz, $CDCl_3$)

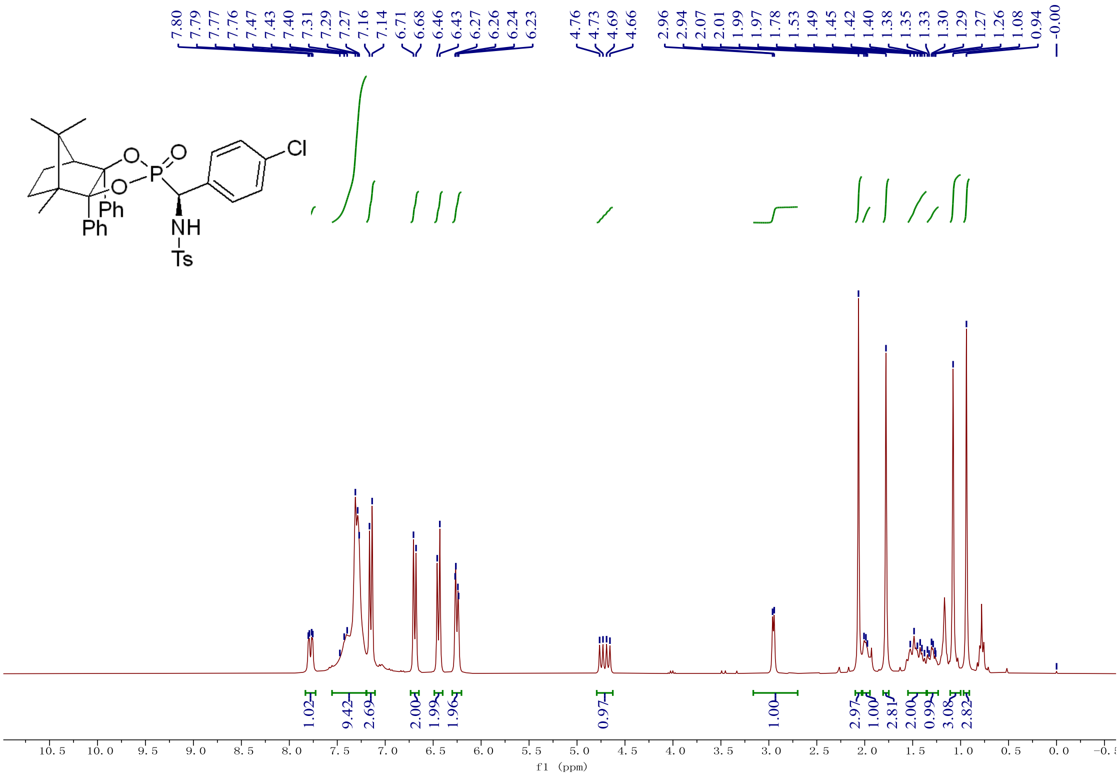

Fig. S131 ^{1}H NMR of compound **6d**

^{13}C NMR (75 MHz, $CDCl_3$)

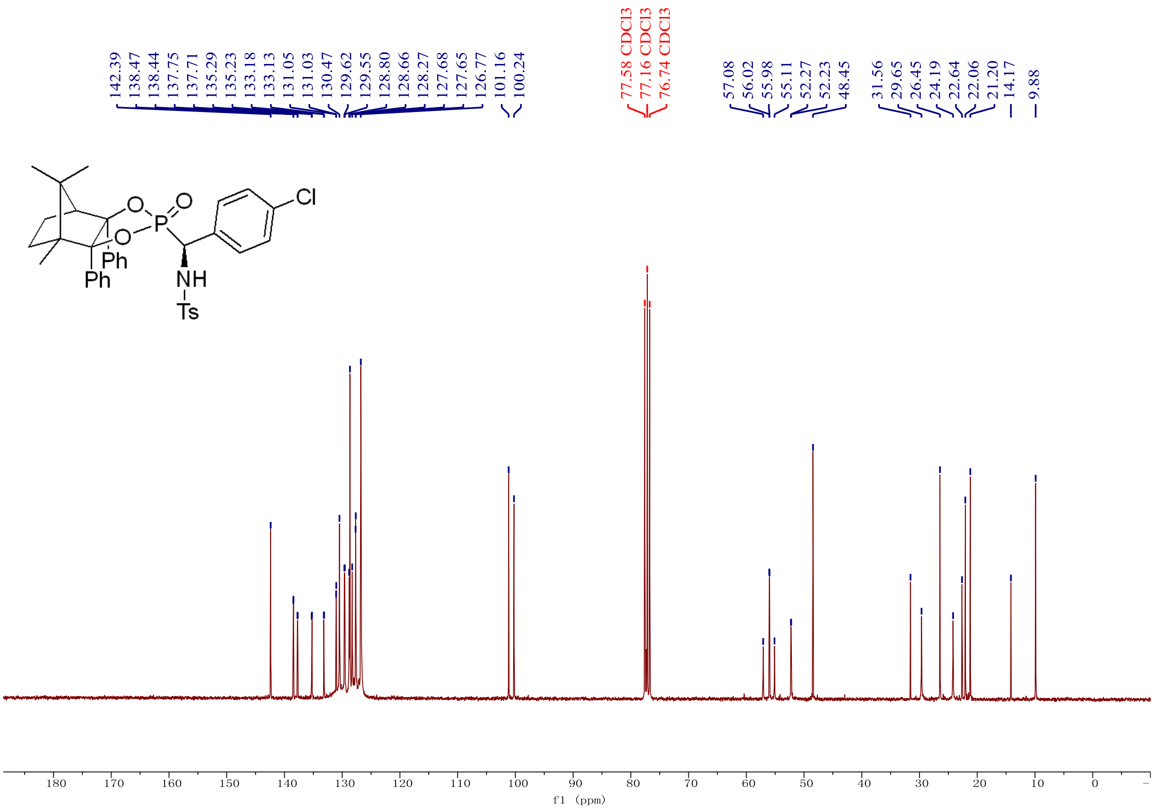

Fig. S132 ^{13}C NMR of compound **6d**

^{31}P NMR (121 MHz, $CDCl_3$)

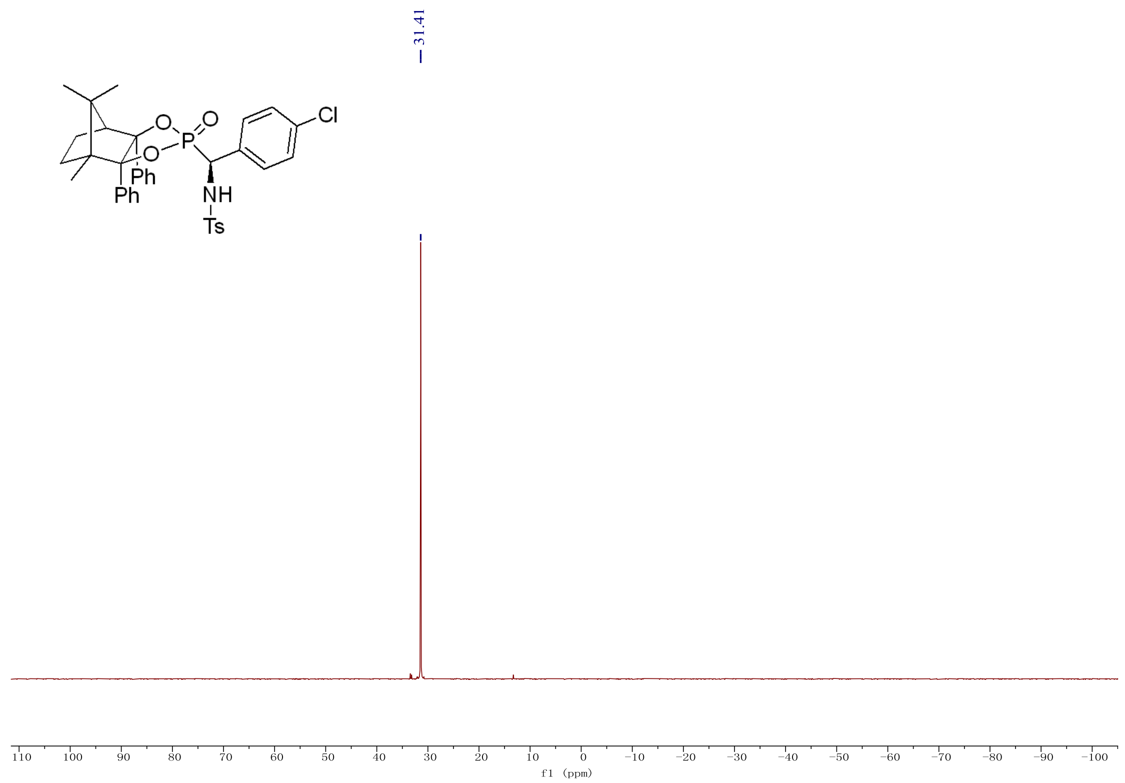

Fig. S133 ^{31}P NMR of compound **6d**

^1H NMR (300 MHz, $CDCl_3$)

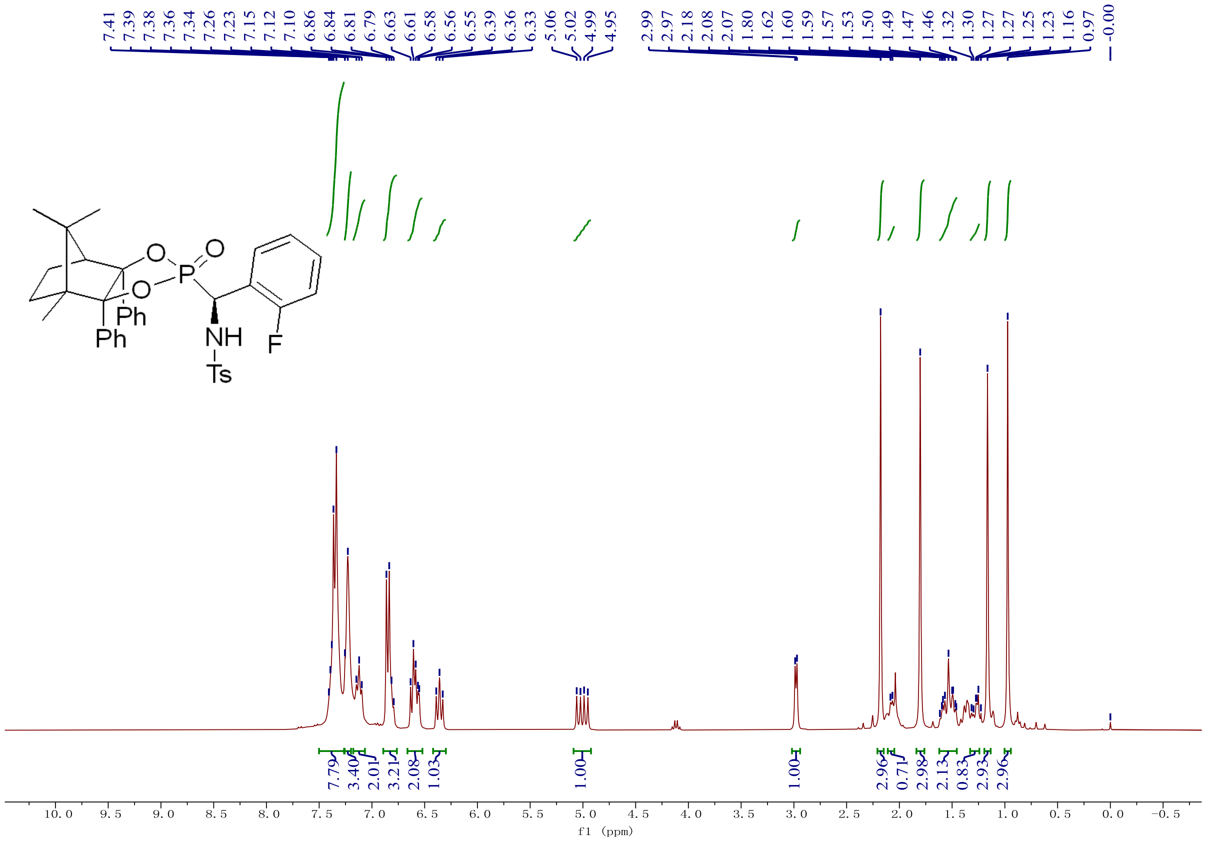

Fig. S134 ^1H NMR of compound **6e**

^{13}C NMR (75 MHz, $CDCl_3$)

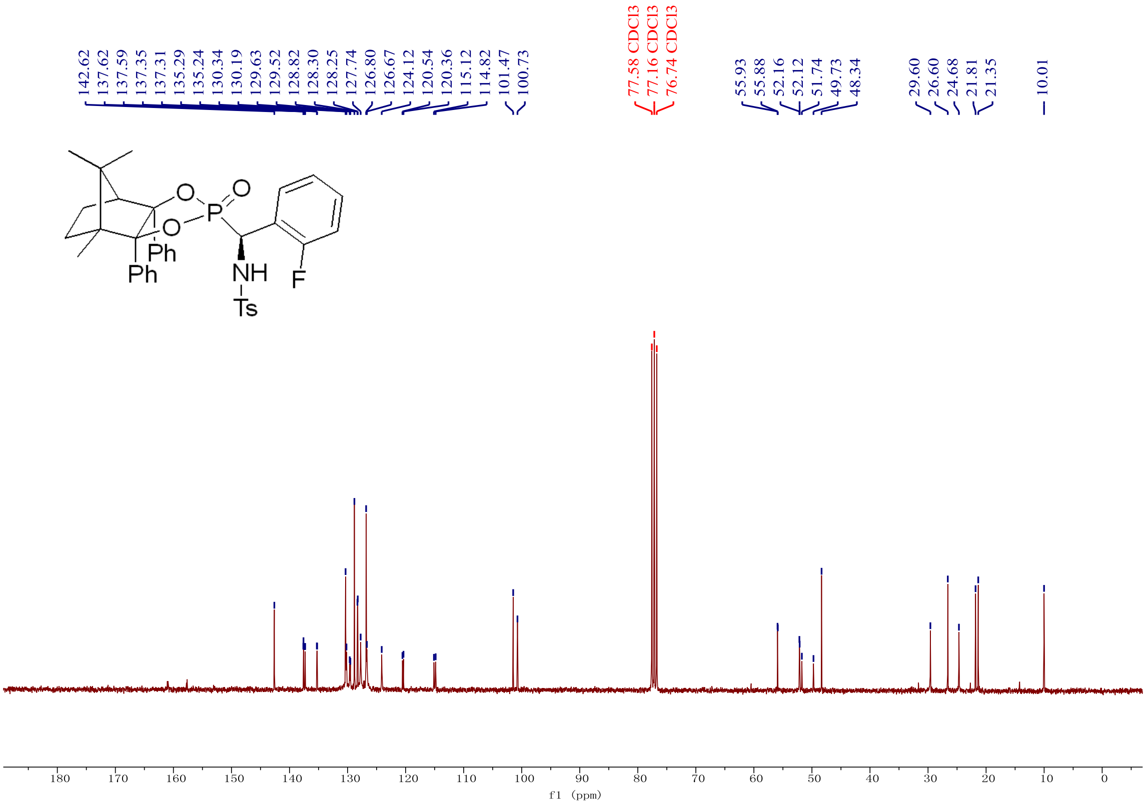

Fig. S135 ^{13}C NMR of compound **6e**

^{19}F NMR (282 MHz, $CDCl_3$)

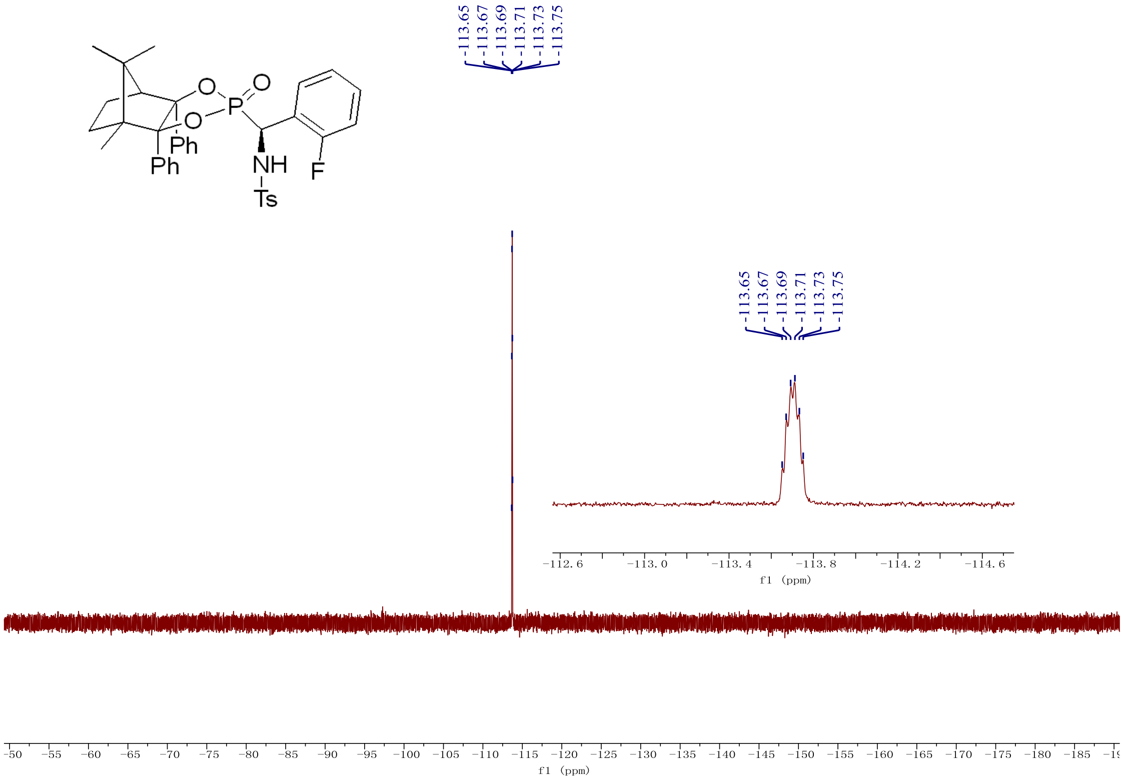

Fig. S136 ^{19}F NMR of compound **6e**

^{31}P NMR (121 MHz, $CDCl_3$)

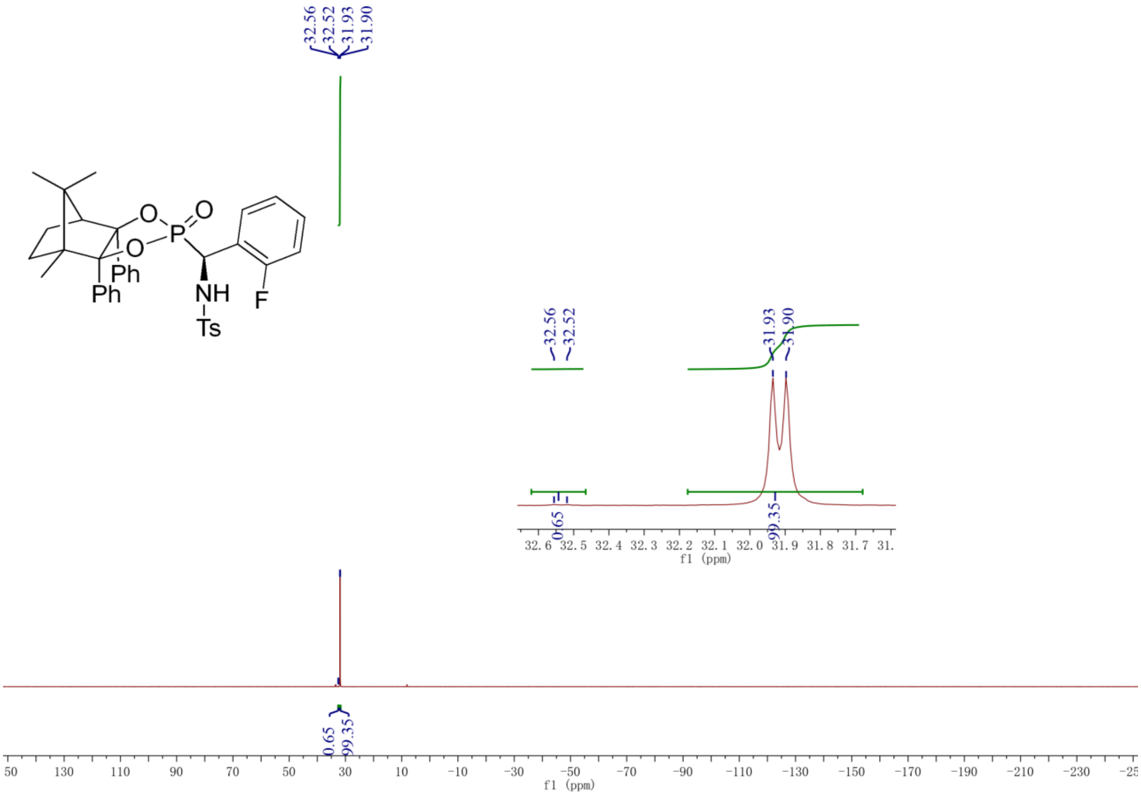

Fig. S137 ^{31}P NMR of compound **6e**

^{1}H NMR (300 MHz, $CDCl_3$)

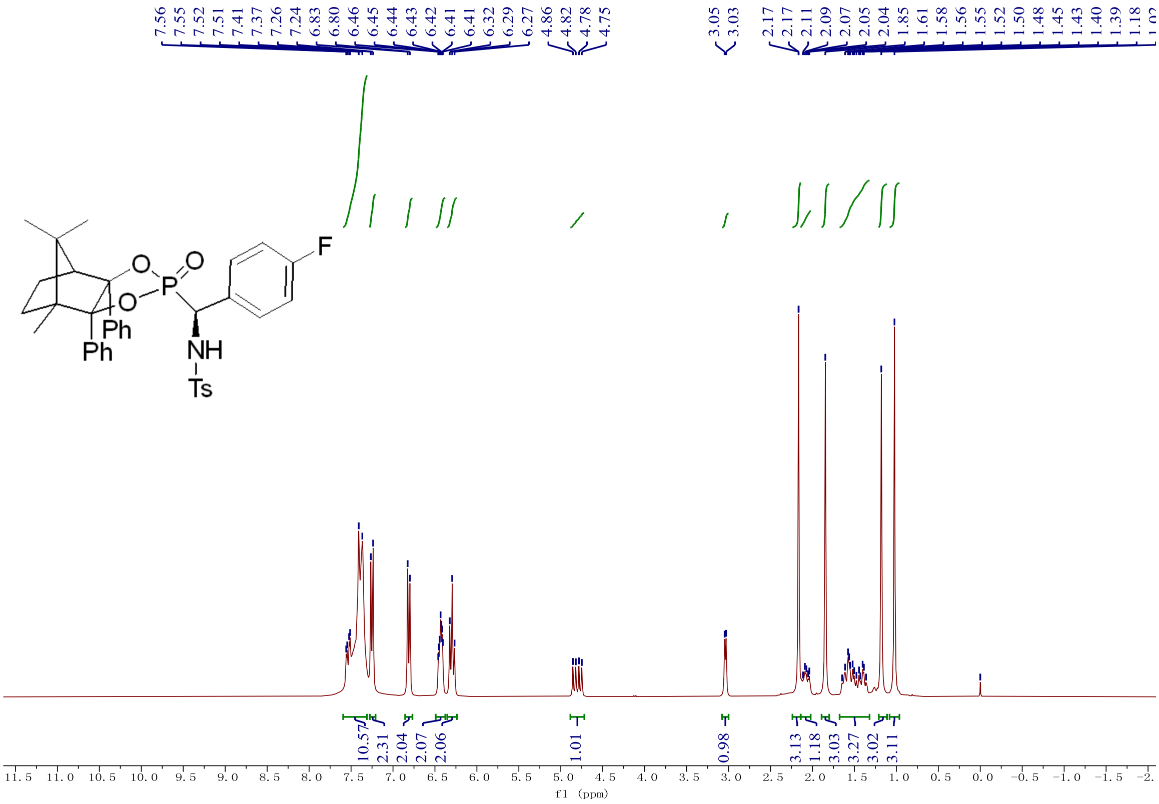

Fig. S138 ^{1}H NMR of compound **6f**

^{13}C NMR (75 MHz, $CDCl_3$)

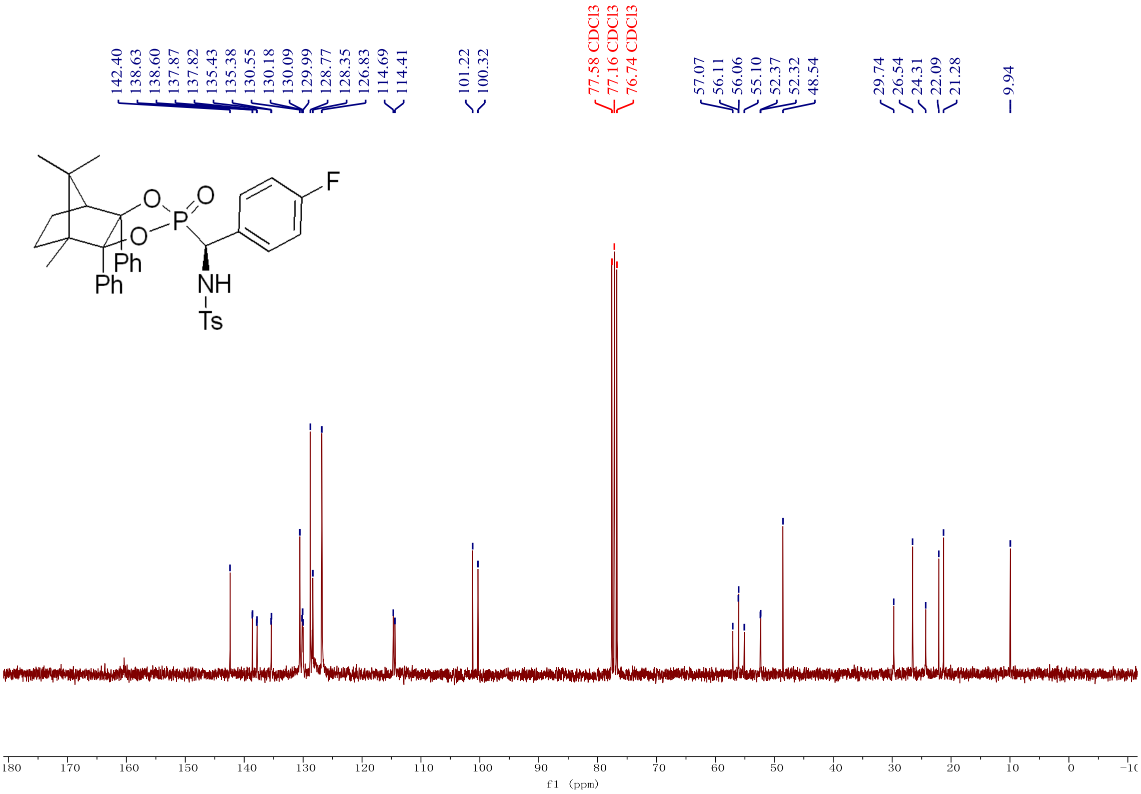

Fig. S139 ^{13}C NMR of compound **6f**

^{19}F NMR (282 MHz, $CDCl_3$)

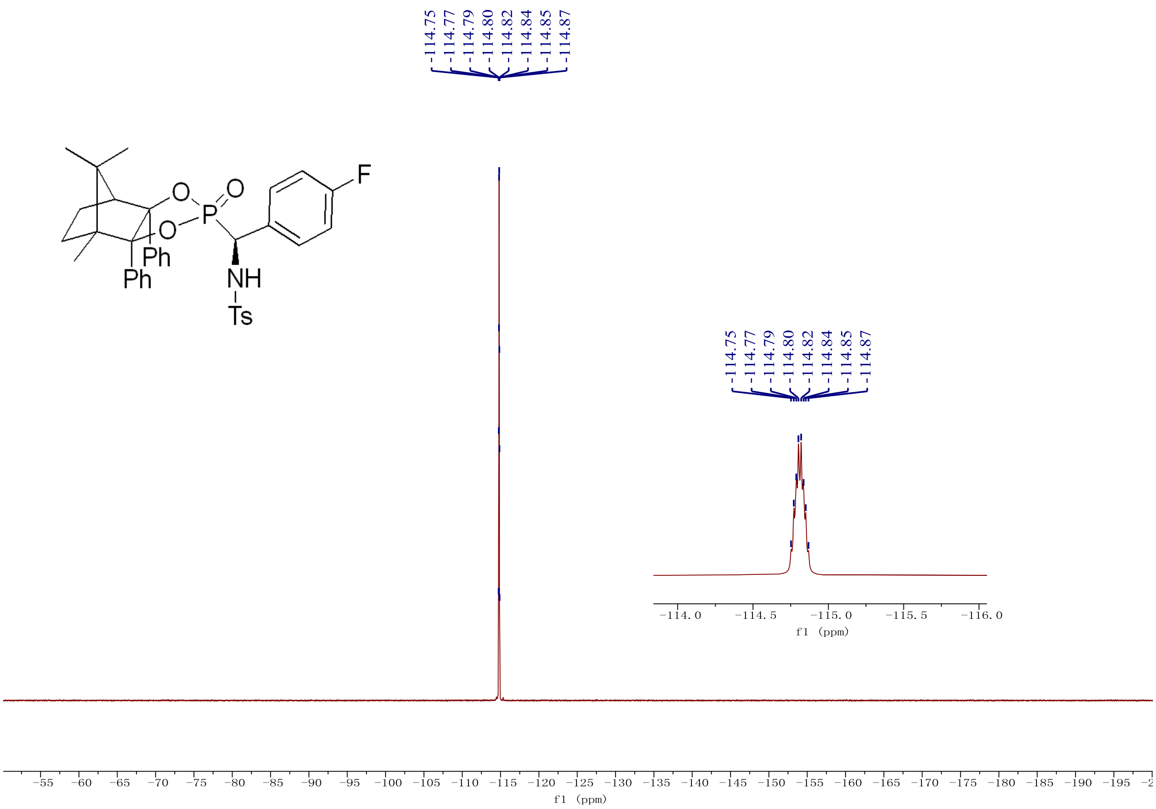

Fig. S140 ^{19}F NMR of compound **6f**

^{31}P NMR (121 MHz, $CDCl_3$)

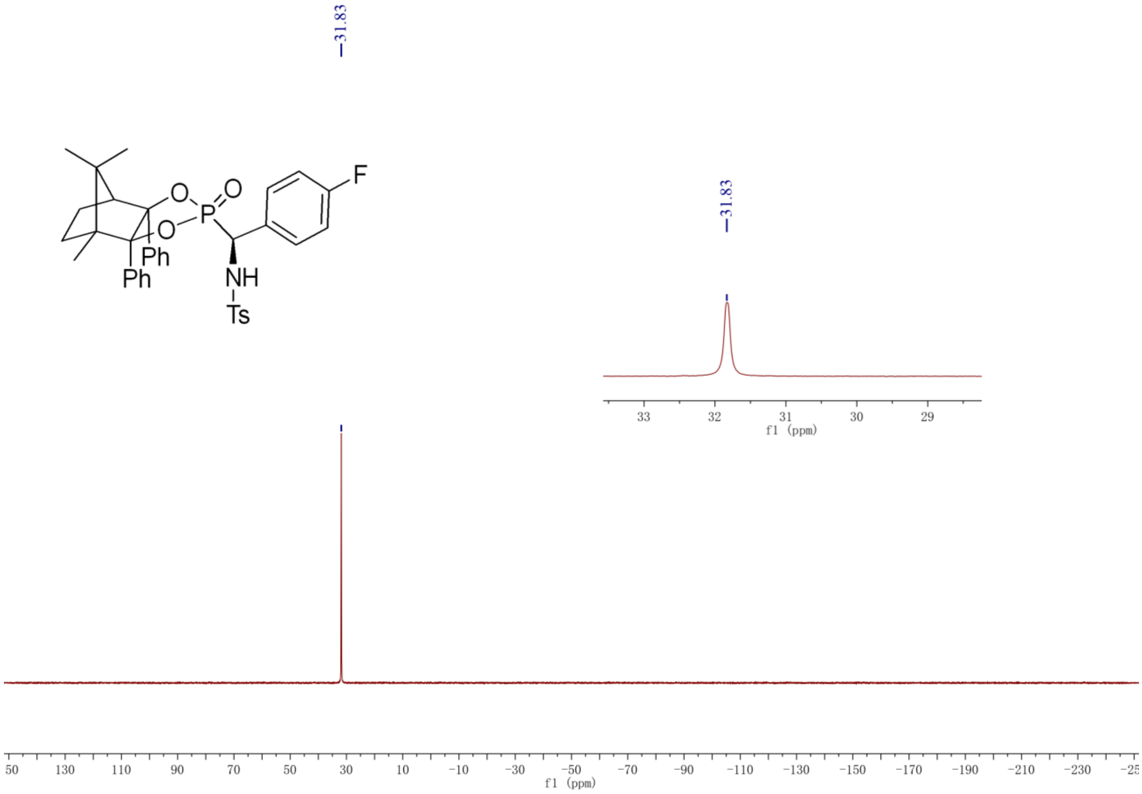

Fig. S141 ^{31}P NMR of compound **6f**

^{1}H NMR (300 MHz, $CDCl_3$)

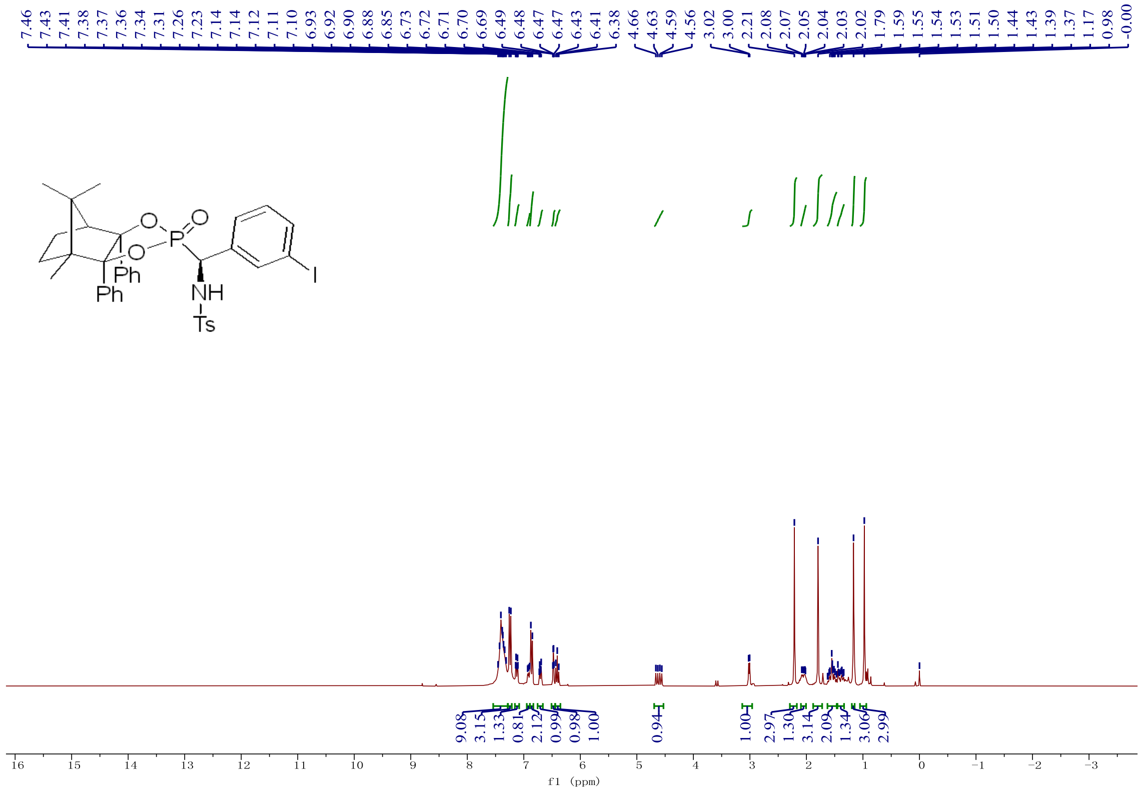

Fig. S142 ^{1}H NMR of compound **6g**

^{13}C NMR (75 MHz, $CDCl_3$)

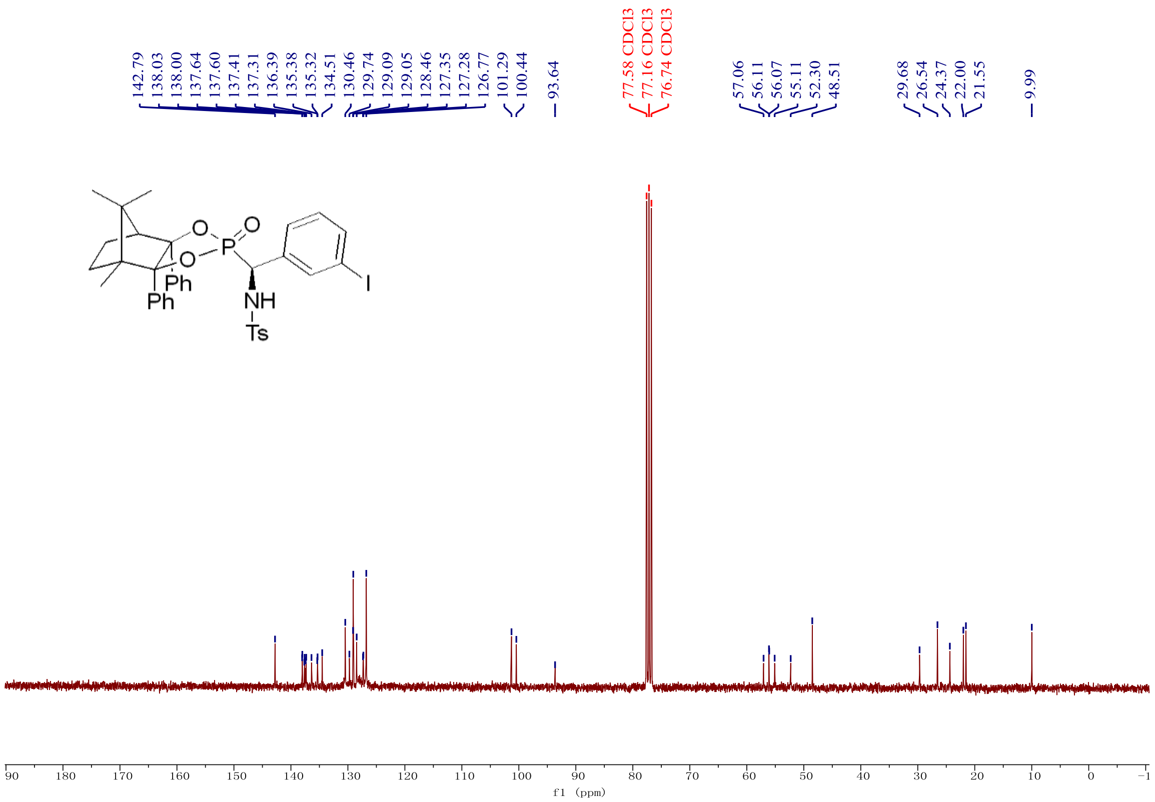

Fig. S143 ^{13}C NMR of compound **6g**

^{31}P NMR (121 MHz, $CDCl_3$)

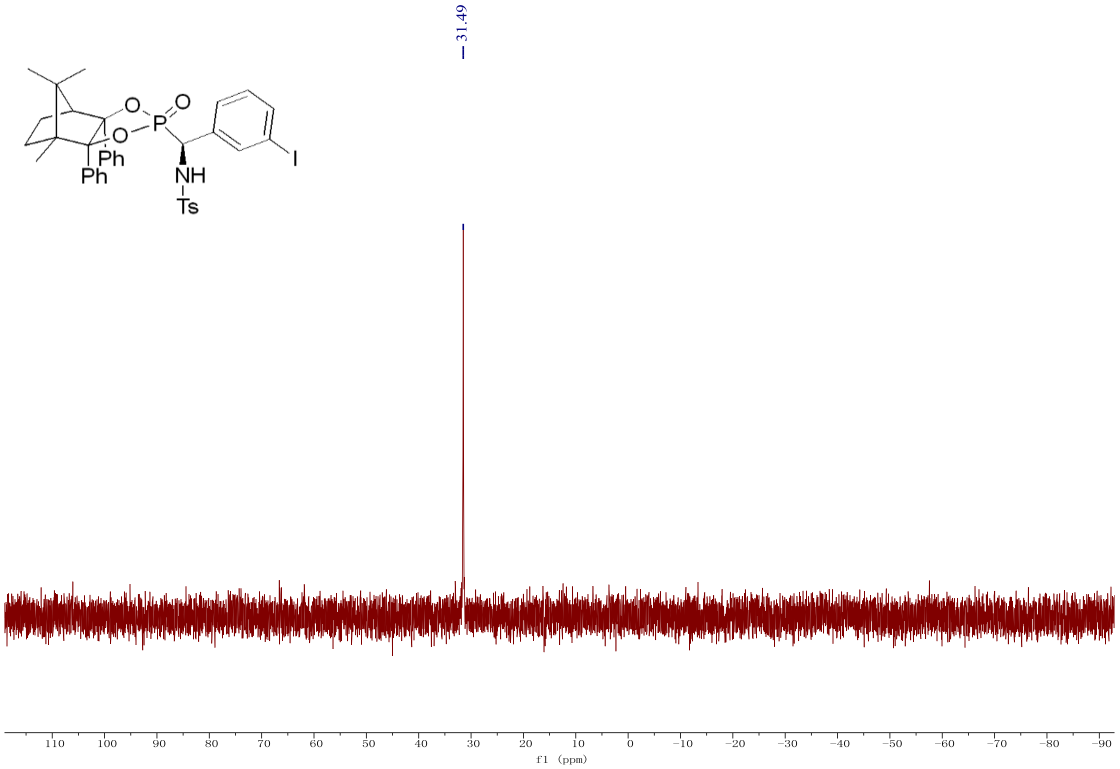

Fig. S144 ^{31}P NMR of compound **6g**

^{1}H NMR (300 MHz, $CDCl_3$)

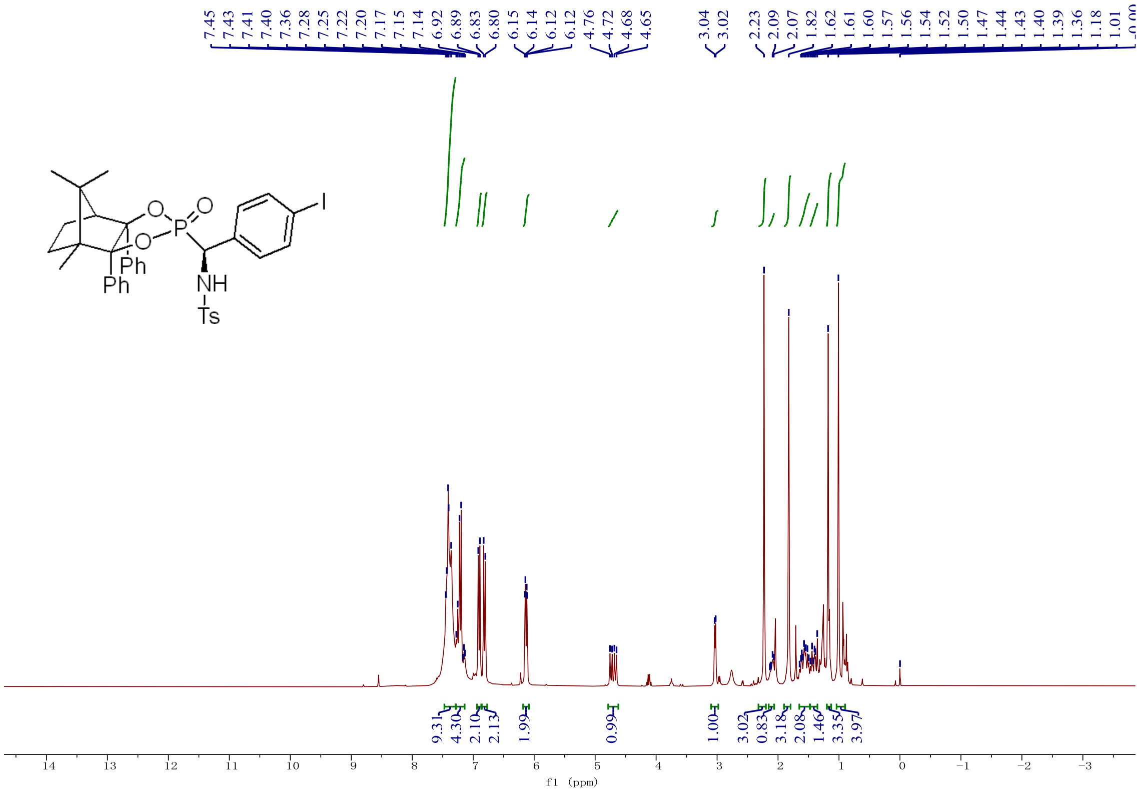

Fig. S145 ^{1}H NMR of compound **6h**

^{13}C NMR (75 MHz, $CDCl_3$)

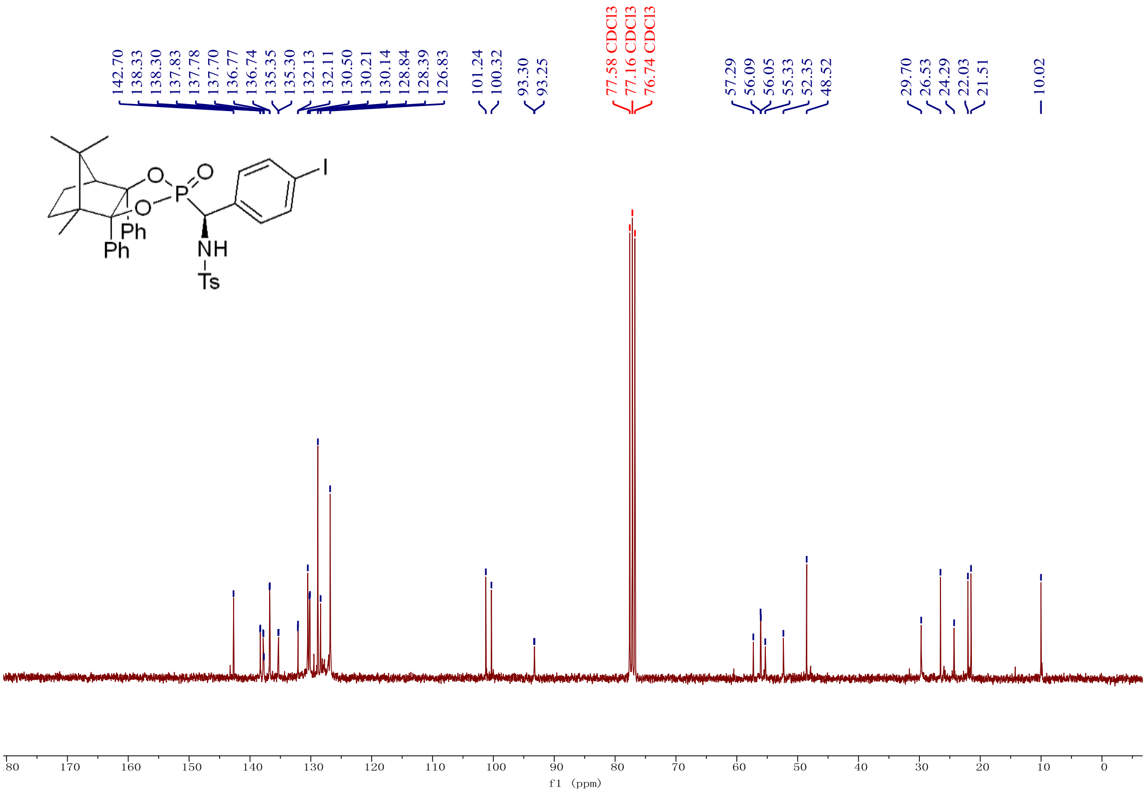

Fig. S146 ^{13}C NMR of compound **6h**

^{31}P NMR (121 MHz, $CDCl_3$)

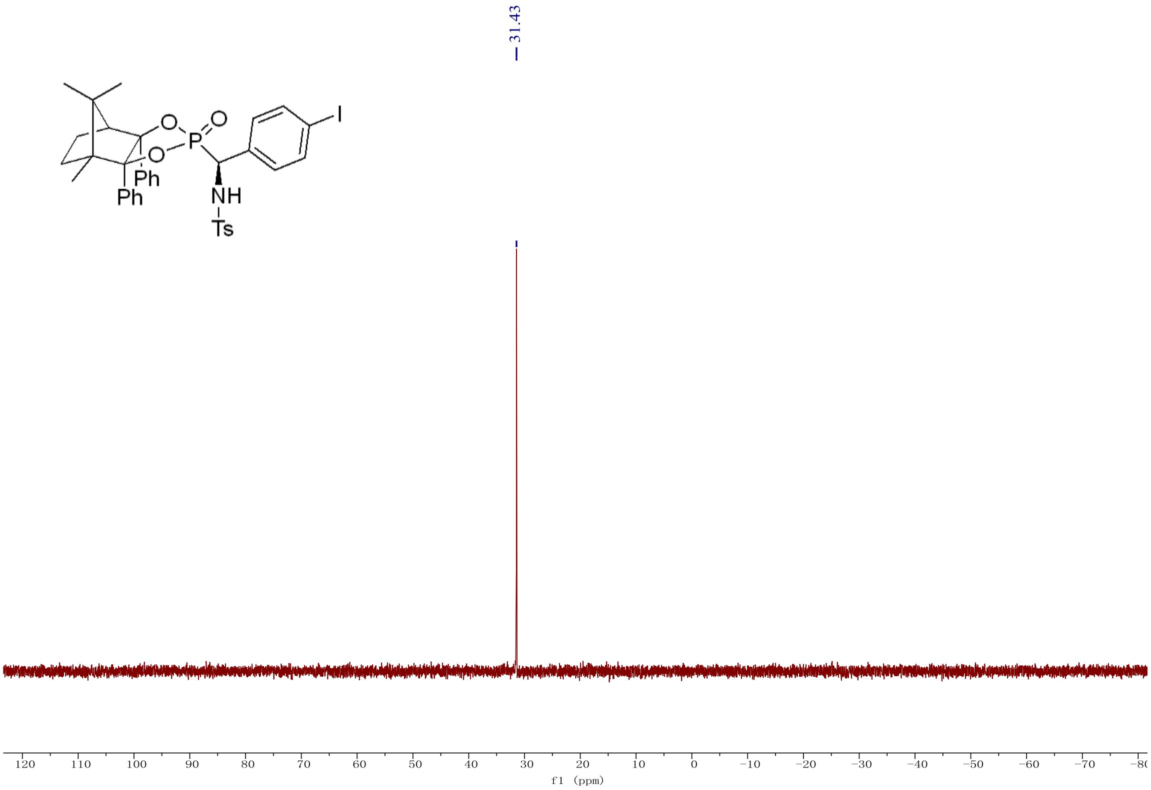

Fig. S147 ^{31}P NMR of compound **6h**

^{1}H NMR (300 MHz, $CDCl_3$)

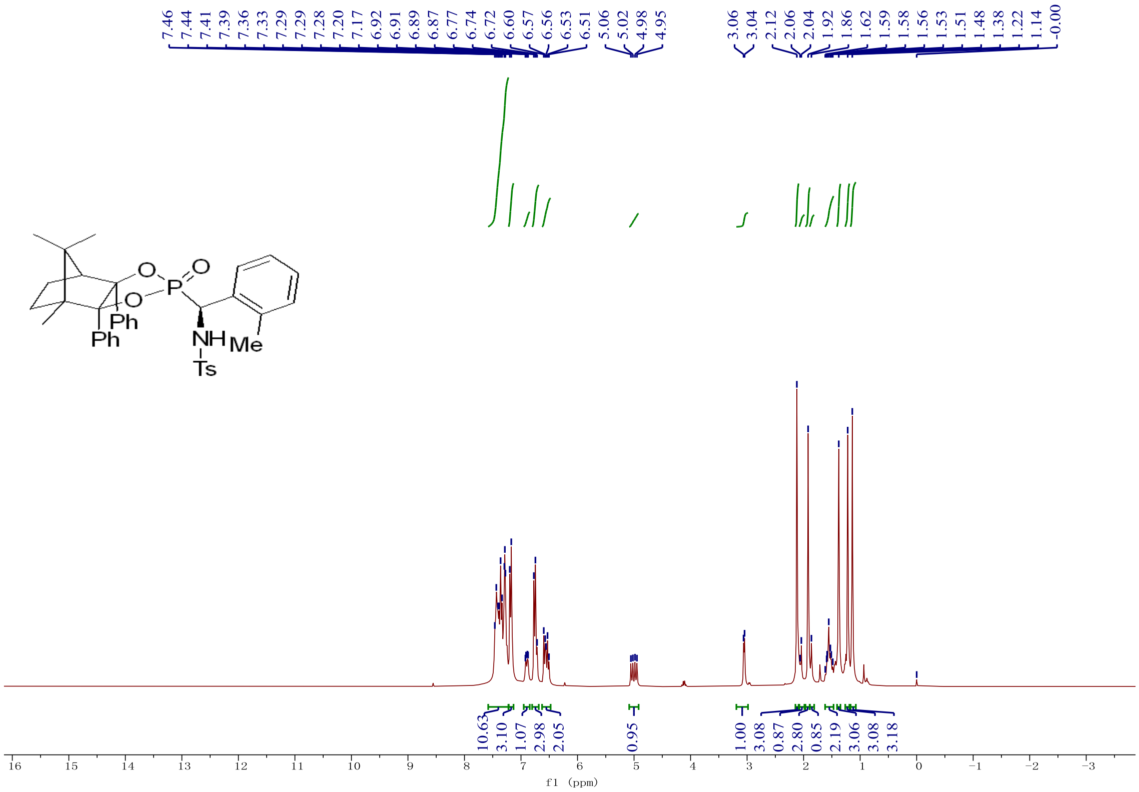

Fig. S148 ^{1}H NMR of compound **6i**

^{13}C NMR (75 MHz, $CDCl_3$)

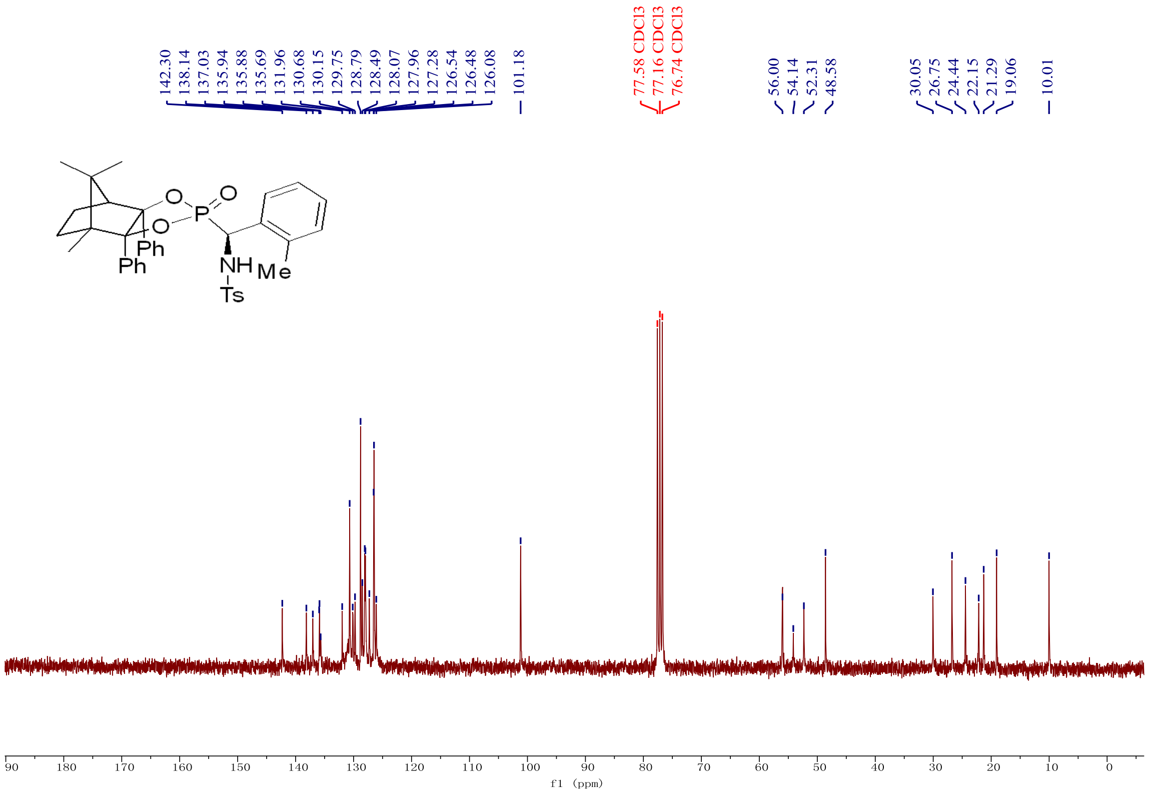

Fig. S149 ^{13}C NMR of compound **6i**

^{31}P NMR (121 MHz, $CDCl_3$)

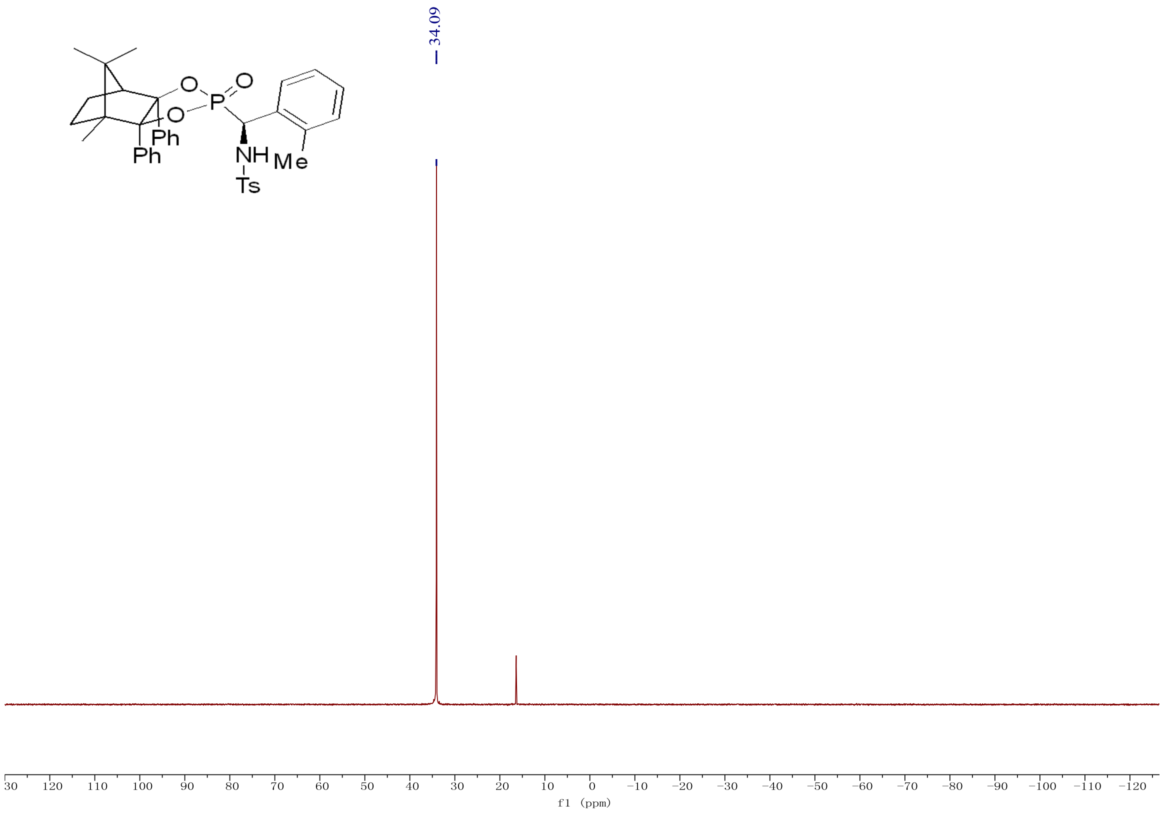

Fig. S150 ^{31}P NMR of compound **6i**

¹H NMR (300 MHz, $CDCl_3$)

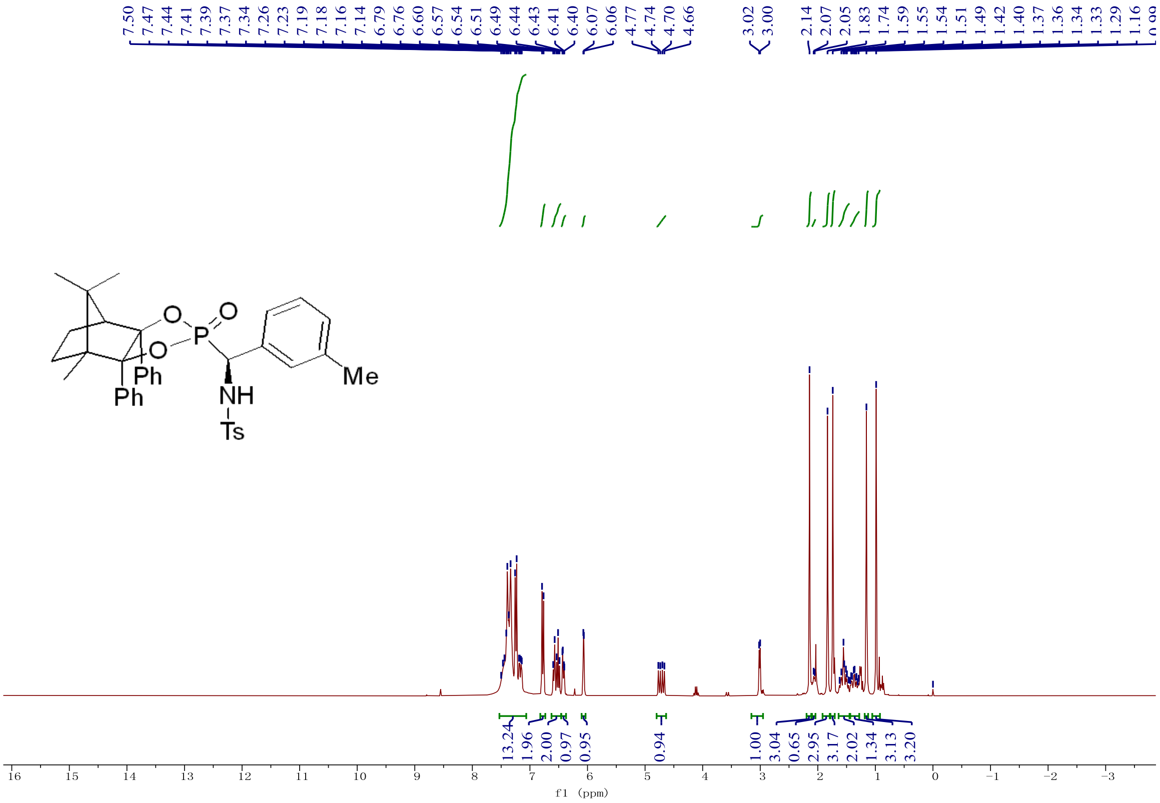

Fig. S151 ¹H NMR of compound **6j**

¹³C NMR (75 MHz, $CDCl_3$)

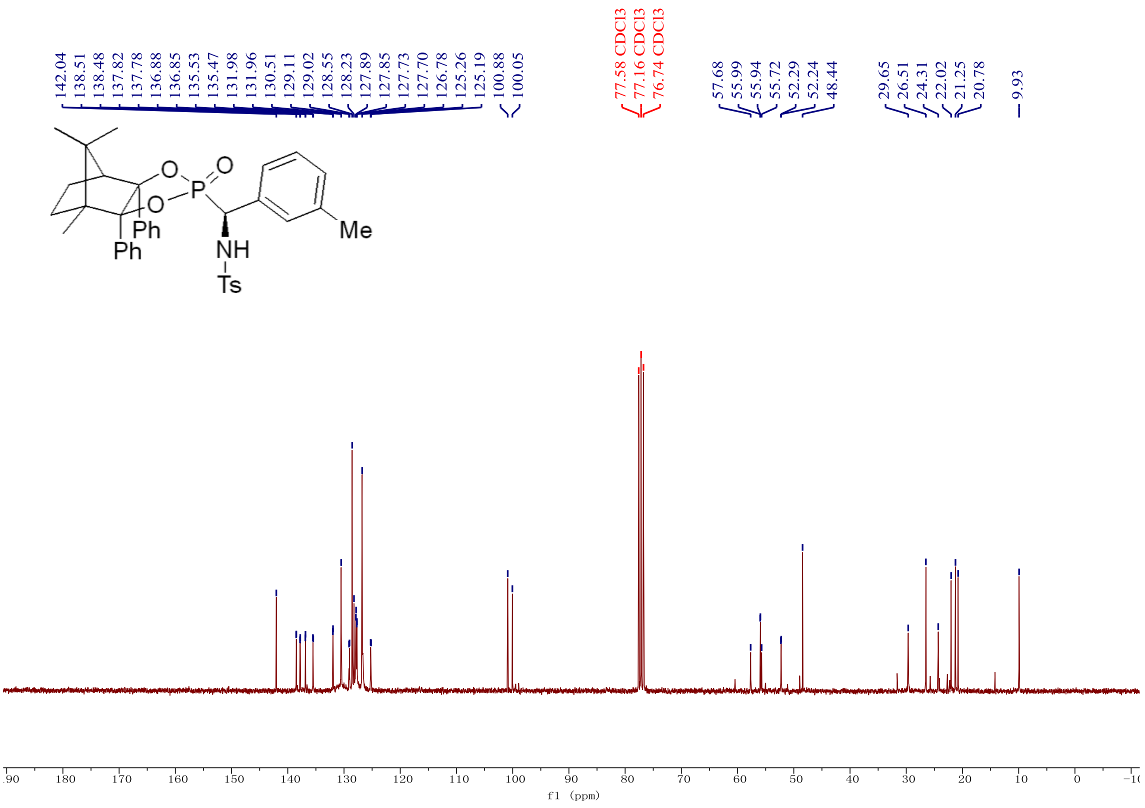

Fig. S152 ¹³C NMR of compound **6j**

^{31}P NMR (121 MHz, $CDCl_3$)

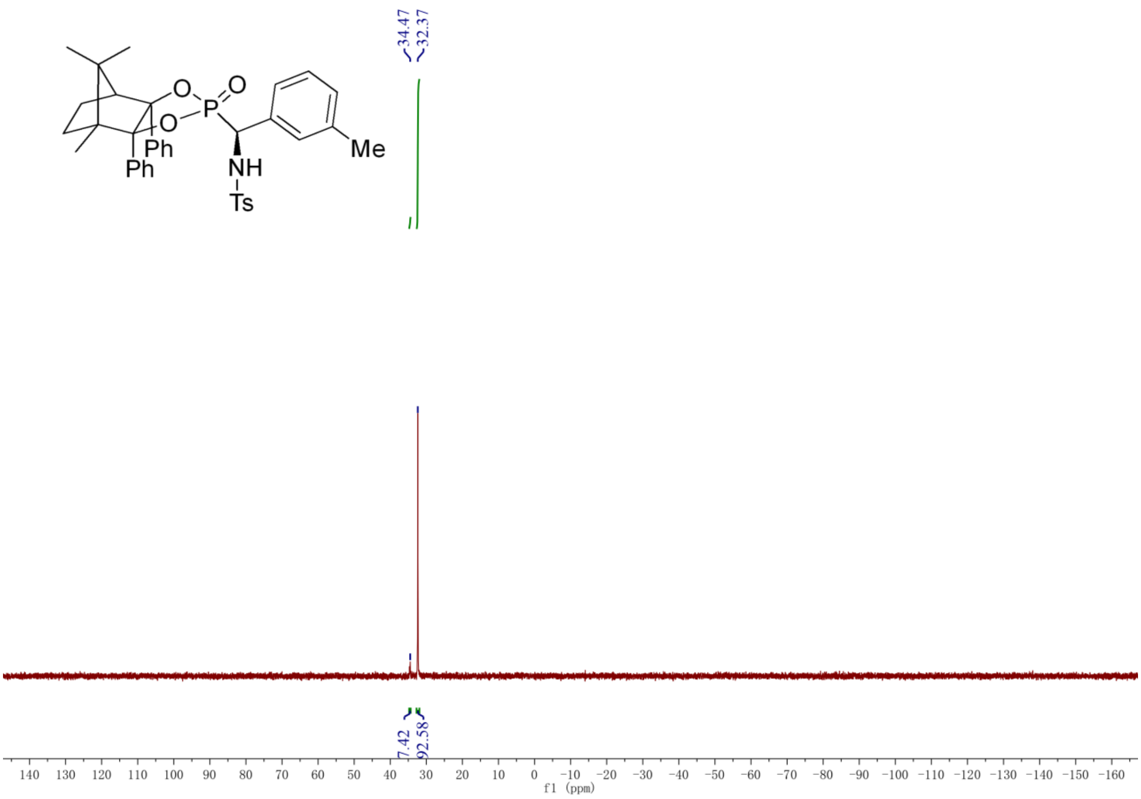

Fig. S153 ^{31}P NMR of compound **6j**

^{1}H NMR (300 MHz, $CDCl_3$)

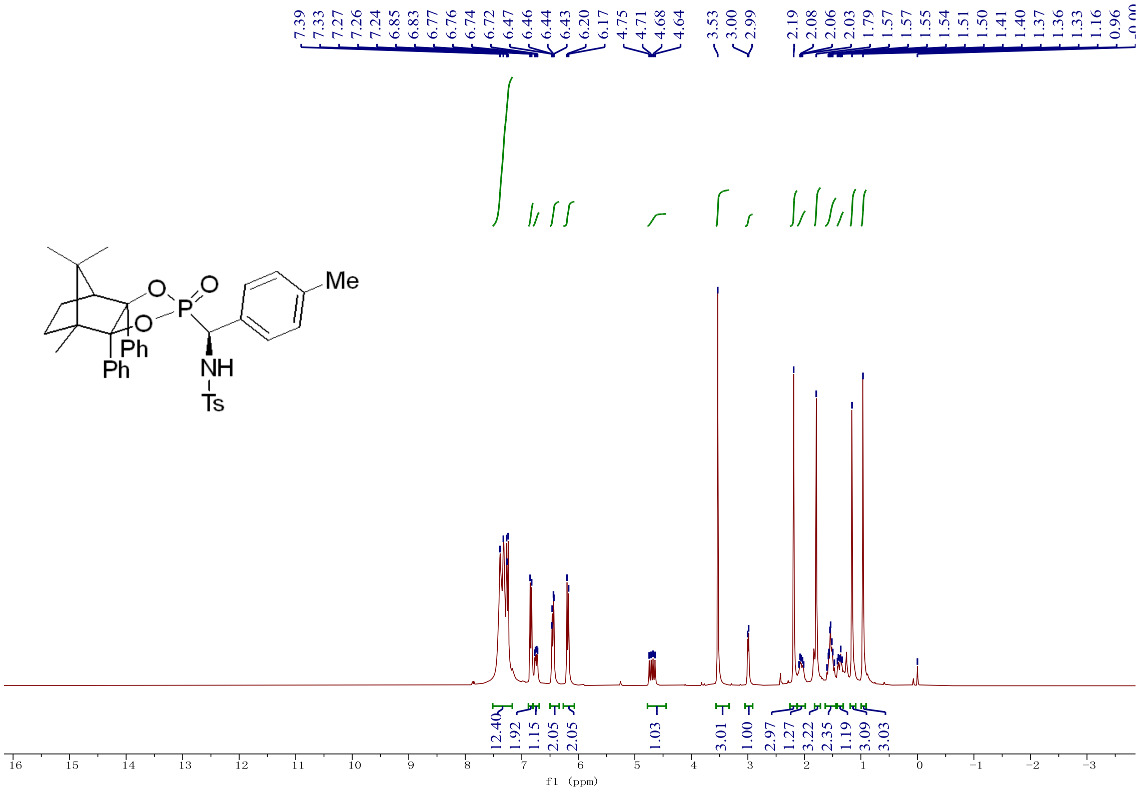

Fig. S154 ^{1}H NMR of compound **6k**

^{13}C NMR (75 MHz, $CDCl_3$)

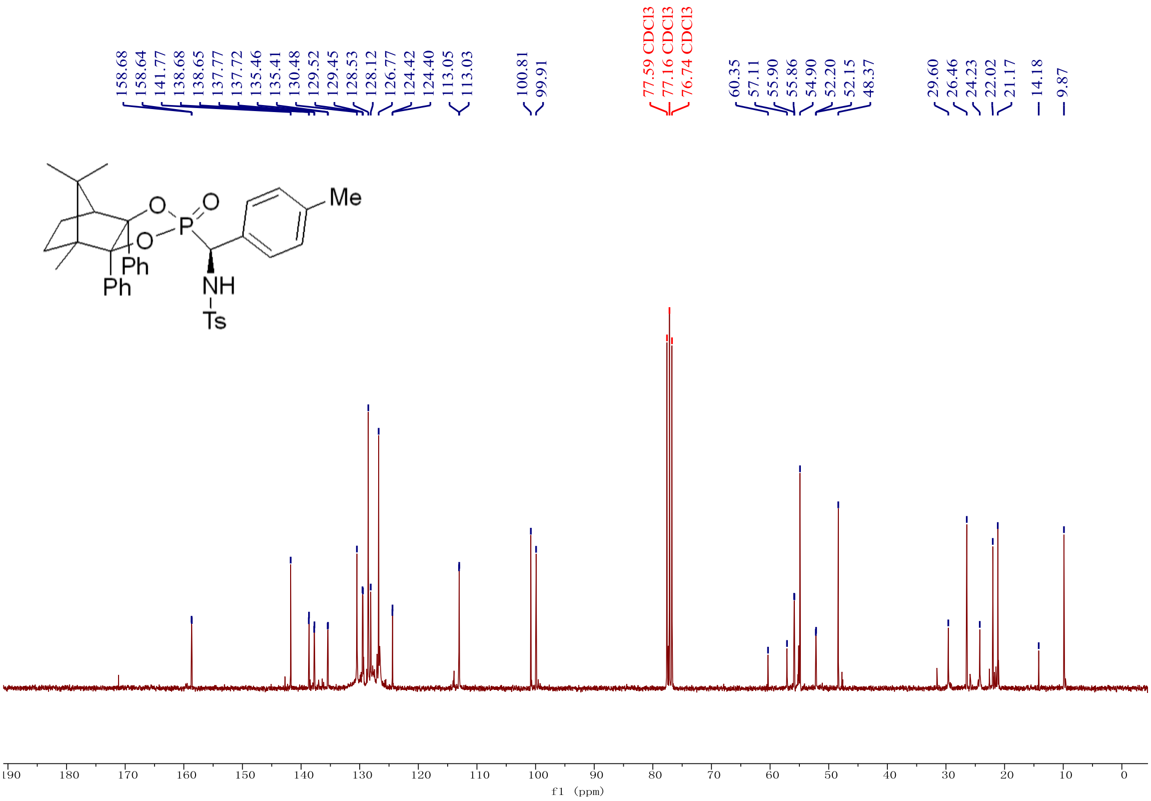

Fig. S155 ^{13}C NMR of compound **6k**

^{31}P NMR (121 MHz, $CDCl_3$)

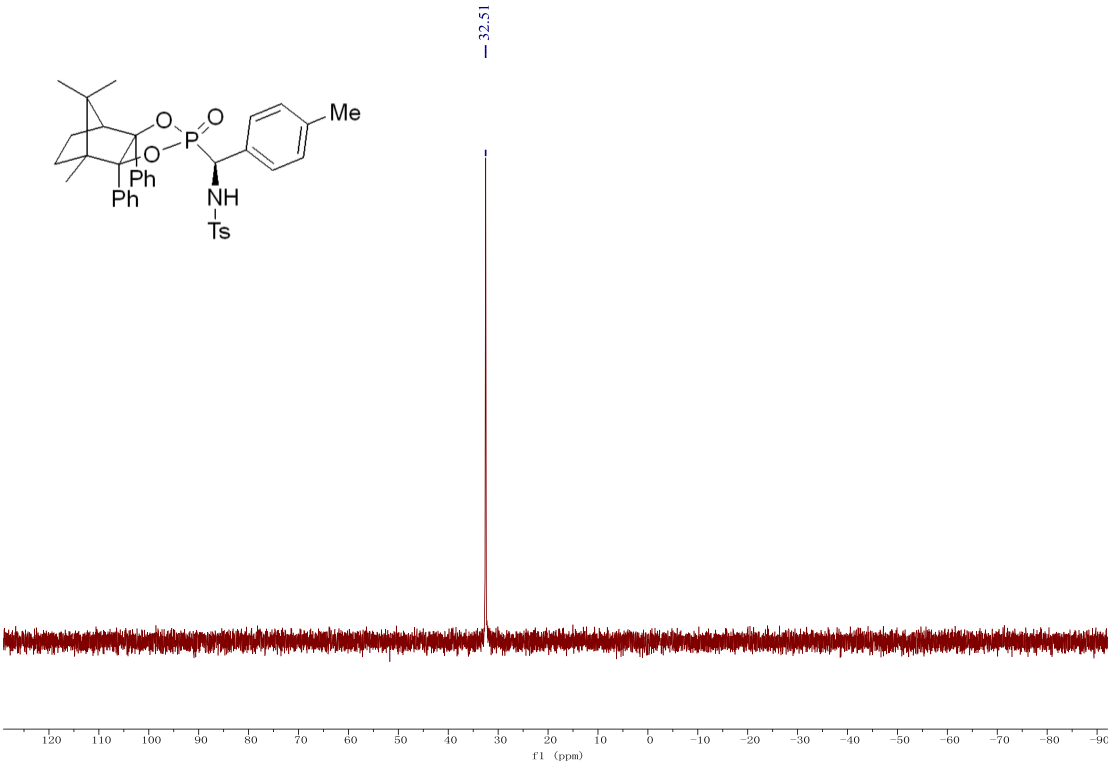

Fig. S156 ^{31}P NMR of compound **6k**

^{1}H NMR (300 MHz, $CDCl_3$)

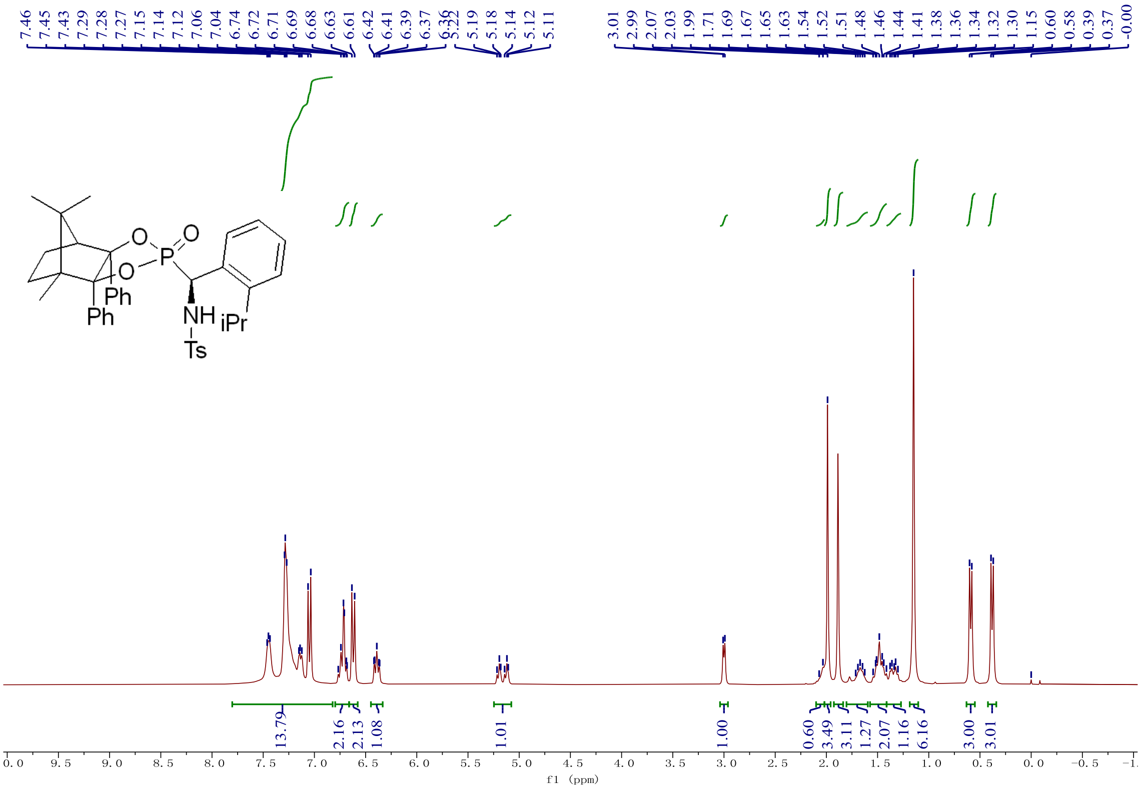

Fig. S157 ^{1}H NMR of compound **6l**

^{13}C NMR (75 MHz, $CDCl_3$)

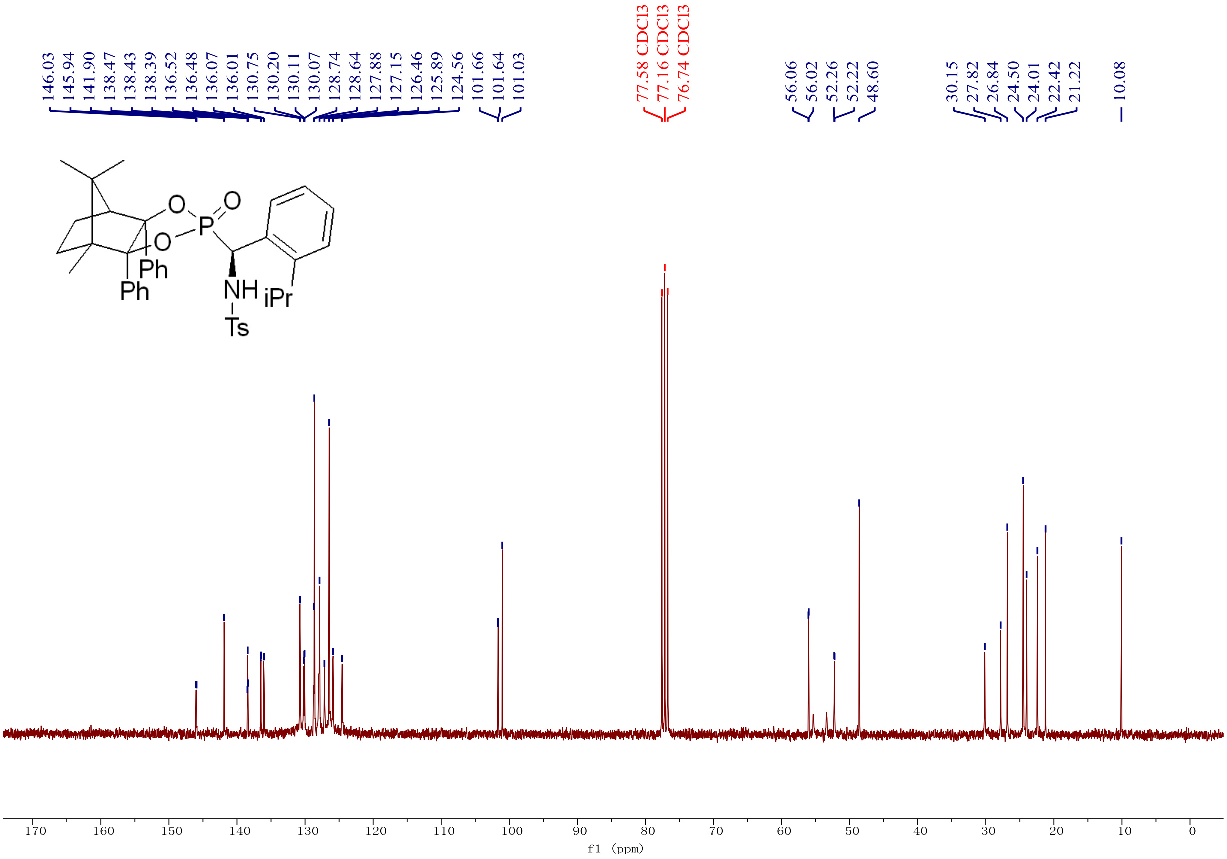

Fig. S158 ^{13}C NMR of compound **6l**

^{31}P NMR (121 MHz, $CDCl_3$)

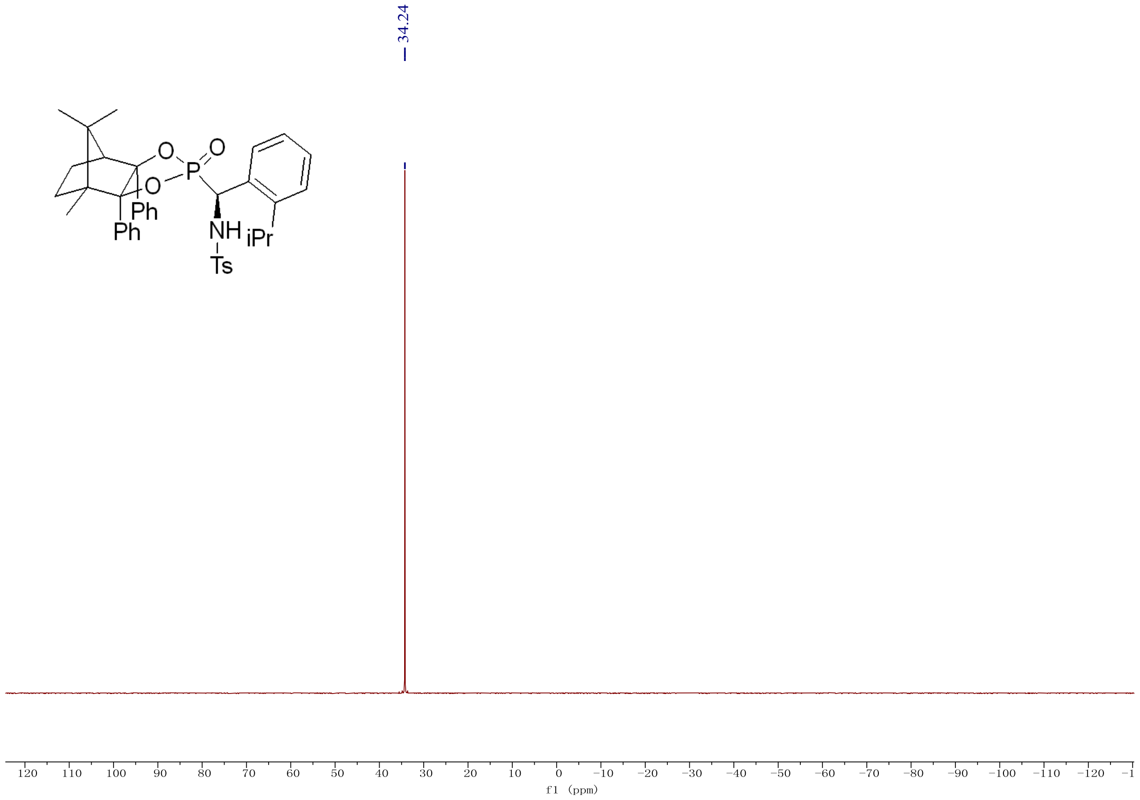

Fig. S159 ^{31}P NMR of compound **6l**

^{1}H NMR (300 MHz, $CDCl_3$)

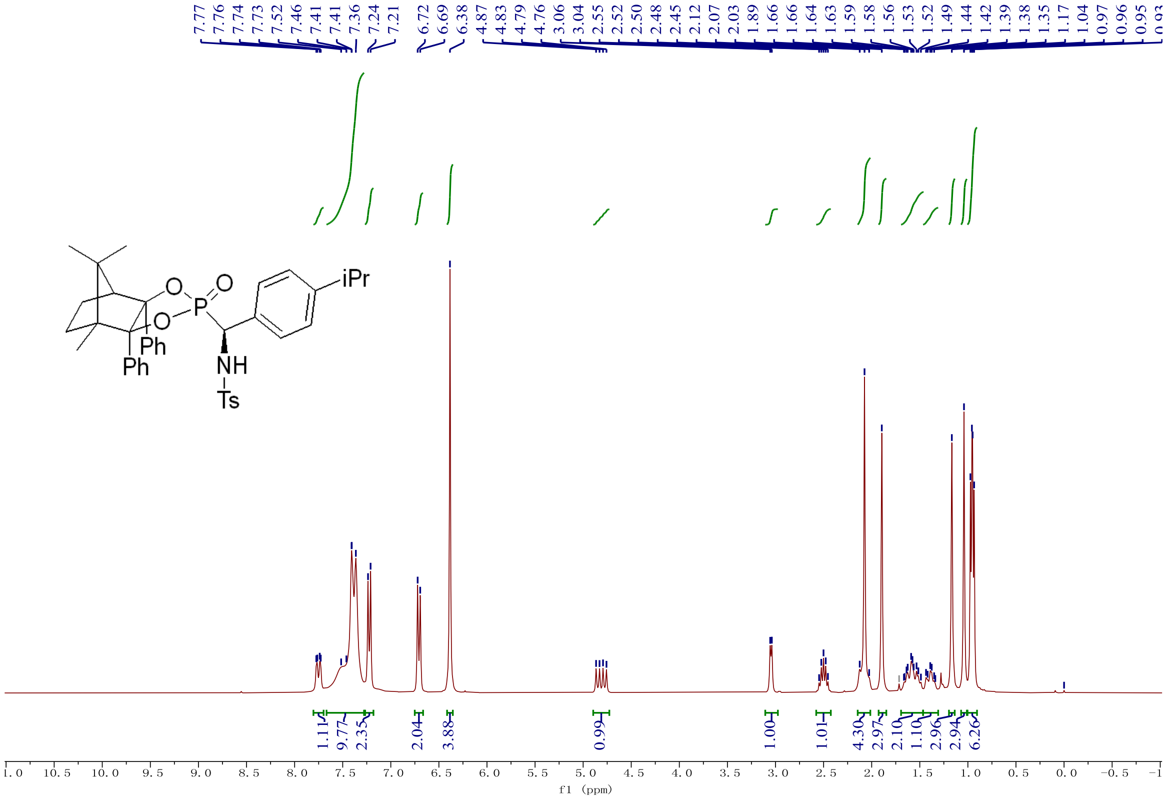

Fig. S160 ^{1}H NMR of compound **6m**

^{13}C NMR (75 MHz, $CDCl_3$)

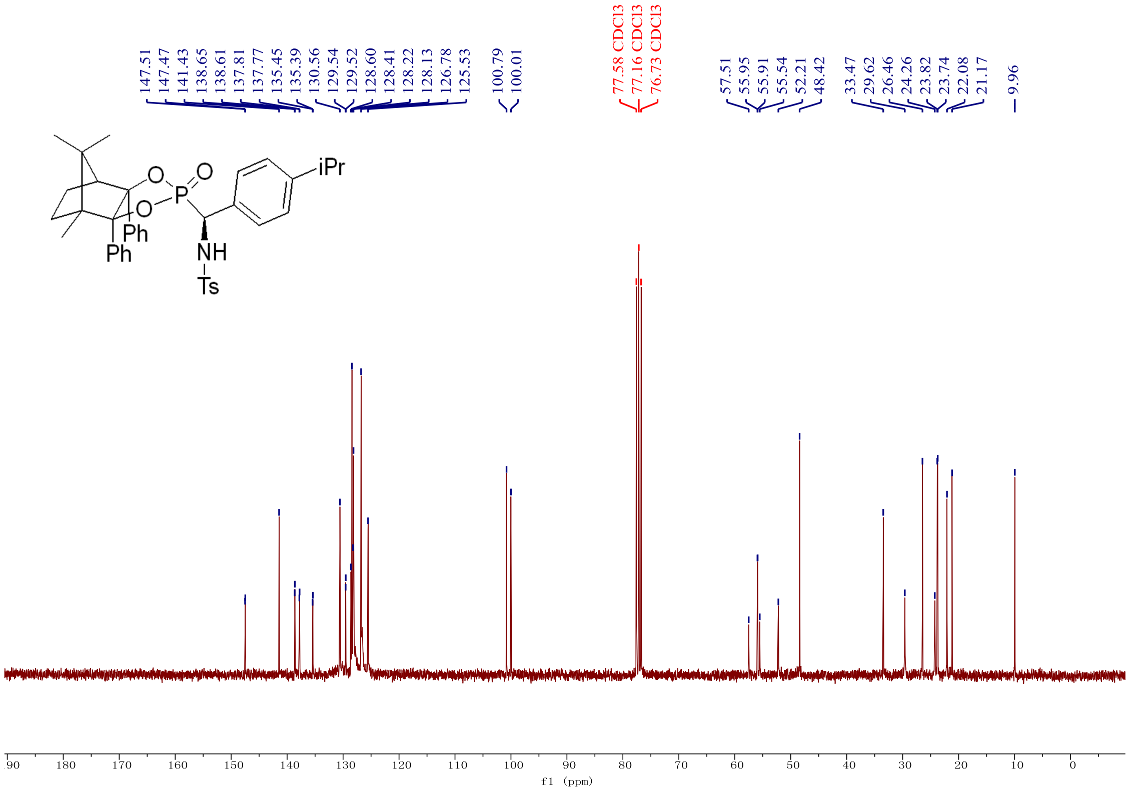

Fig. S161 ^{13}C NMR of compound **6m**

^{31}P NMR (121 MHz, $CDCl_3$)

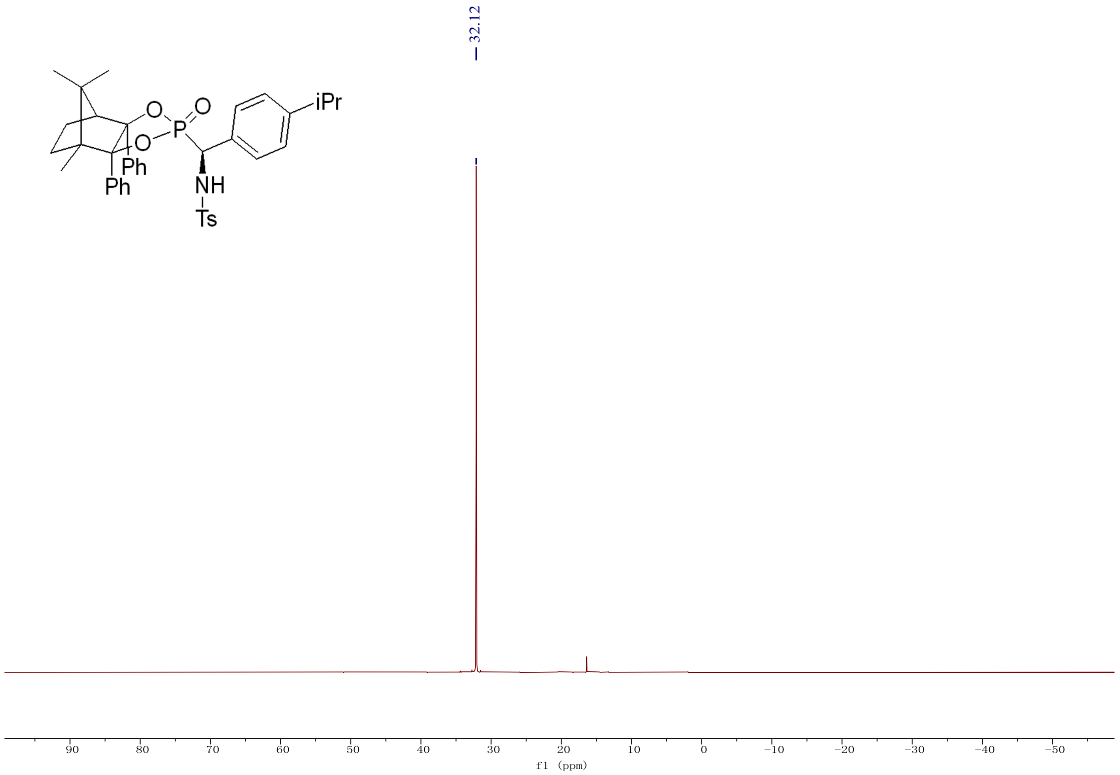

Fig. S162 ^{31}P NMR of compound **6m**

^{1}H NMR (300 MHz, $CDCl_3$)

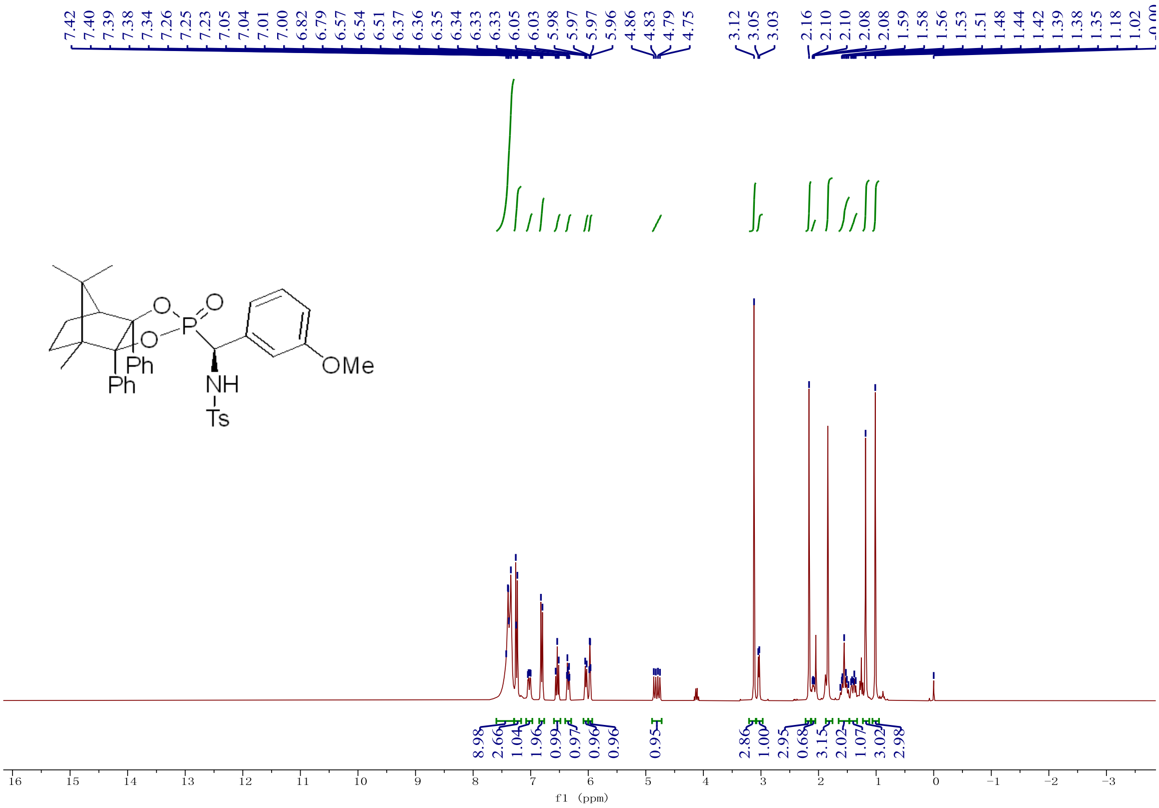

Fig. S163 ^{1}H NMR of compound **6n**

^{13}C NMR (75 MHz, $CDCl_3$)

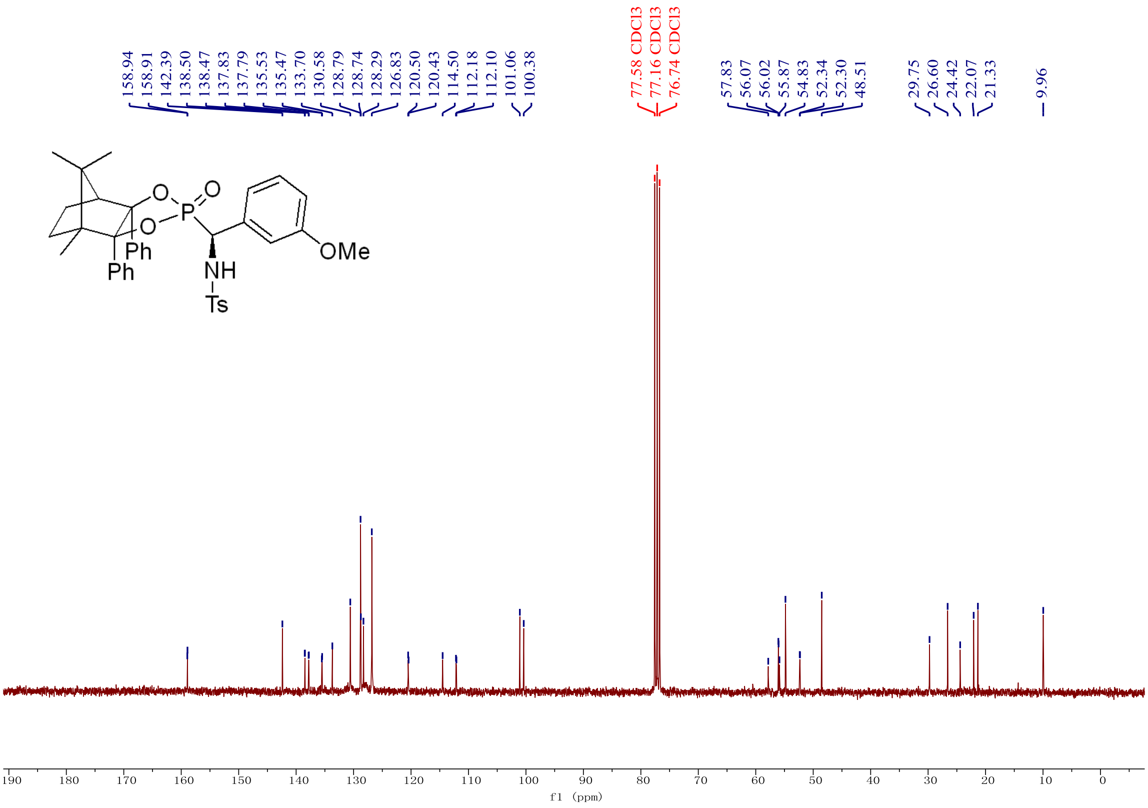

Fig. S164 ^{13}C NMR of compound **6n**

^{31}P NMR (121 MHz, $CDCl_3$)

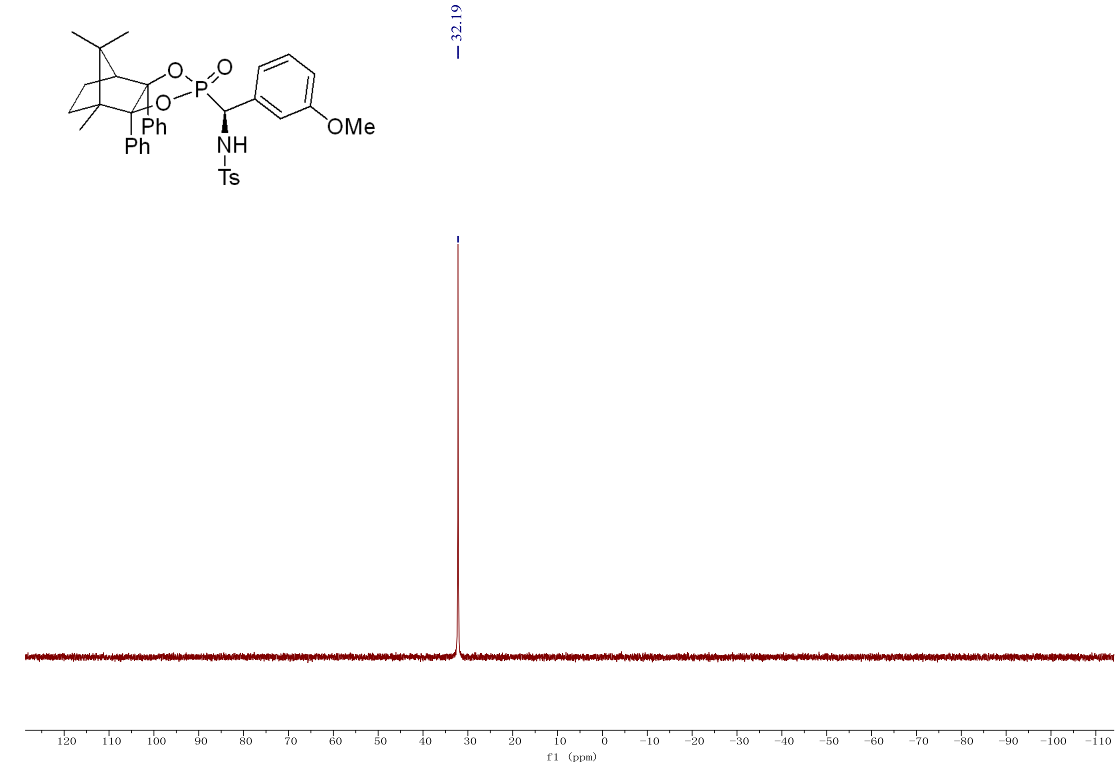

Fig. S165 ^{31}P NMR of compound **6n**

^{1}H NMR (300 MHz, $CDCl_3$)

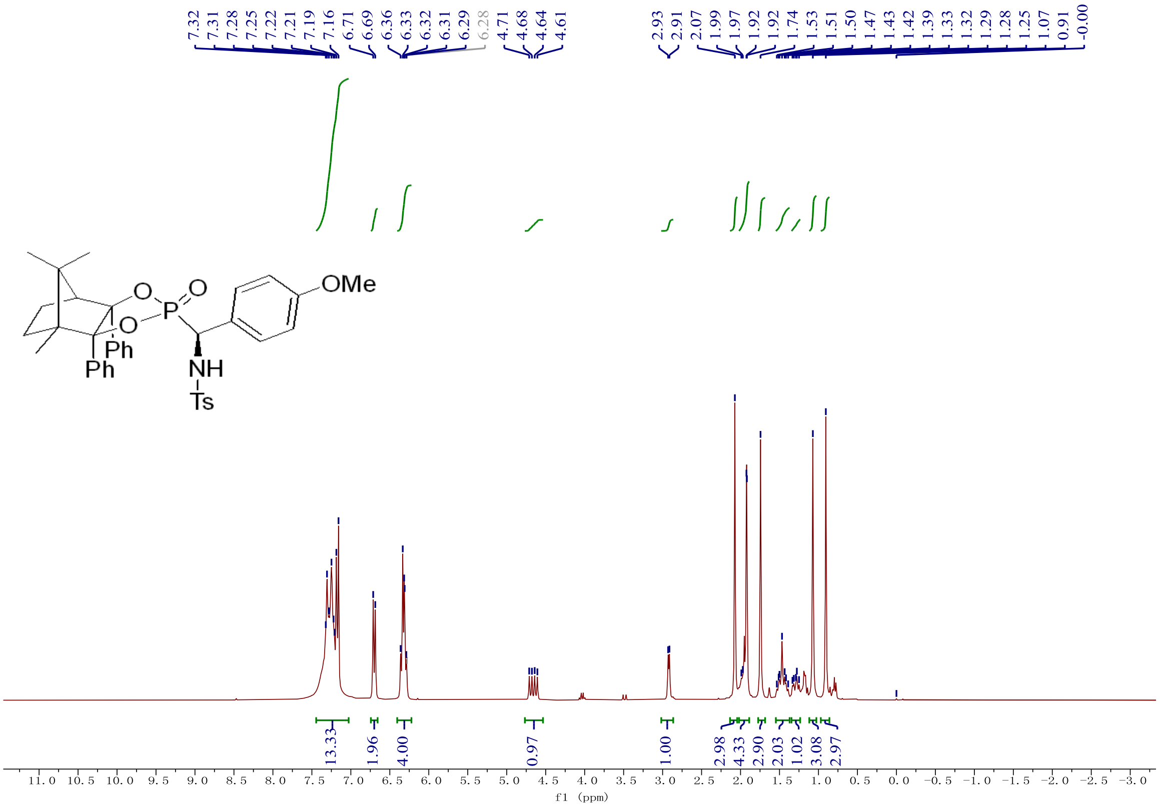

Fig. S166 ^{1}H NMR of compound **6o**

^{13}C NMR (75 MHz, $CDCl_3$)

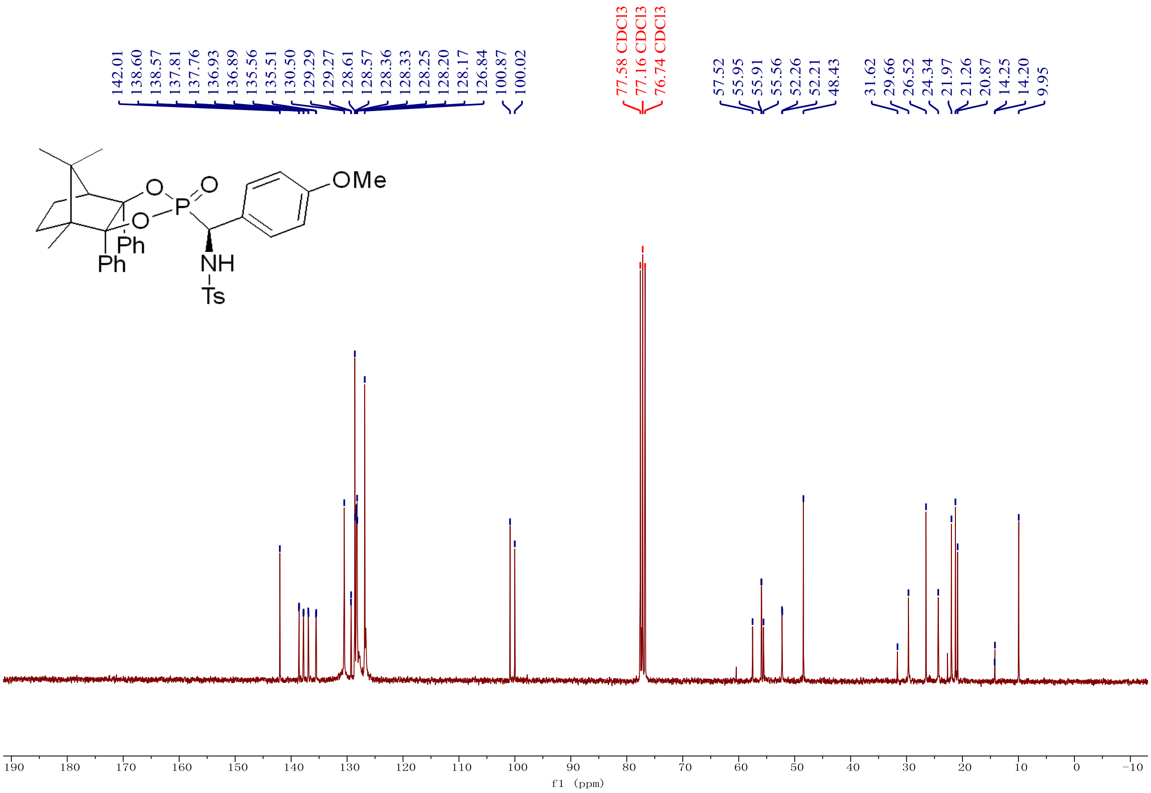

Fig. S167 ^{13}C NMR of compound **6o**

^{31}P NMR (121 MHz, $CDCl_3$)

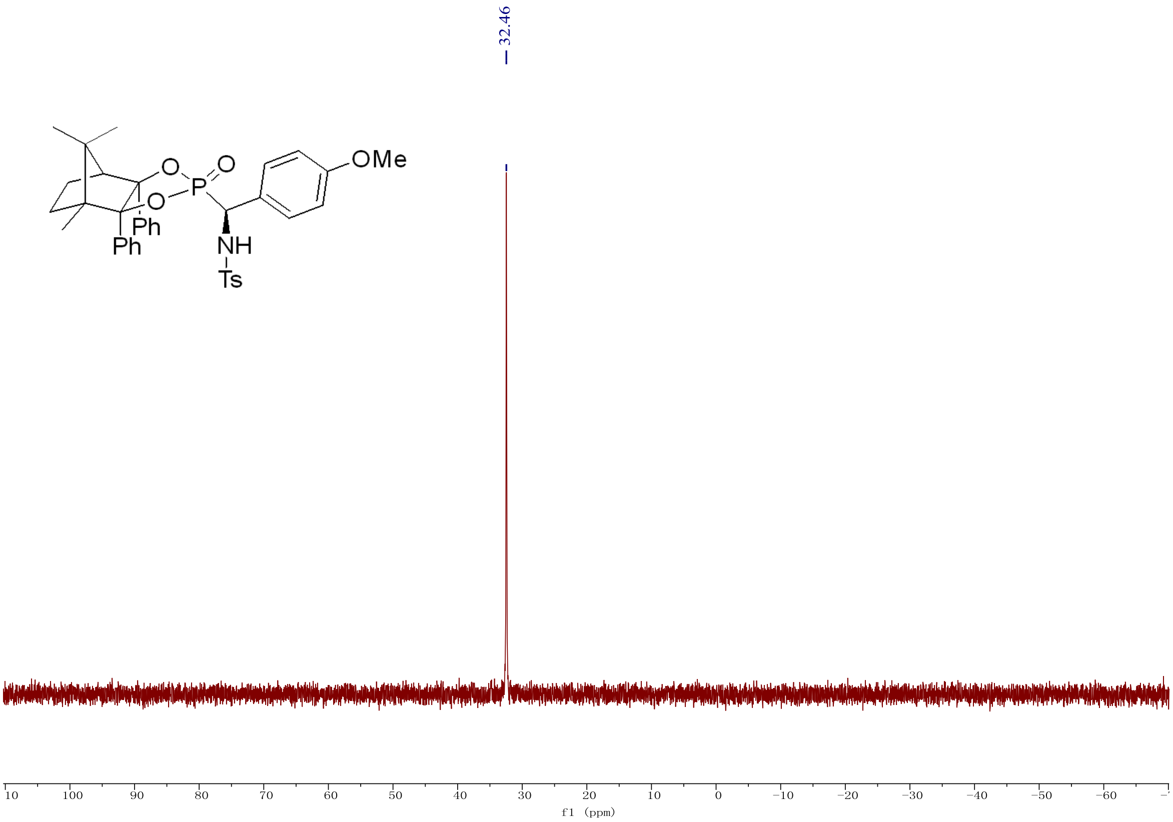

Fig. S168 ^{31}P NMR of compound **6o**

^{1}H NMR (300 MHz, $CDCl_3$)

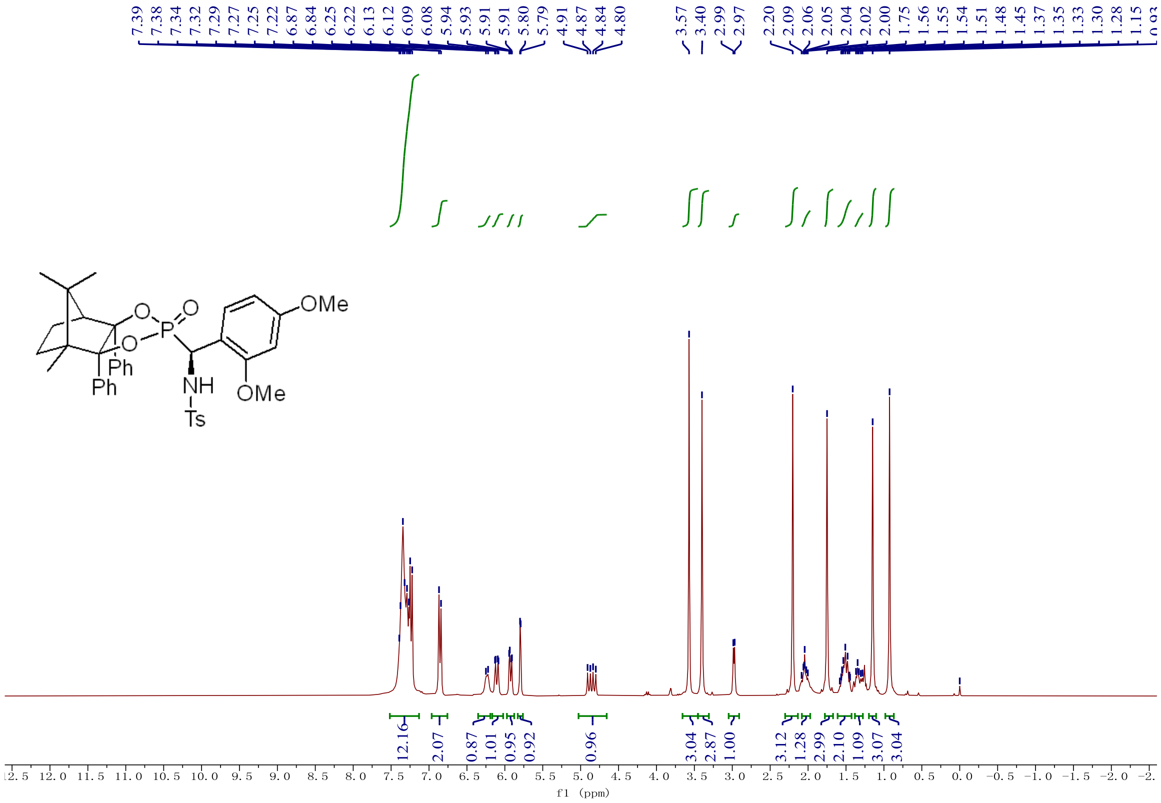

Fig. S169 ^{1}H NMR of compound **6p**

^{13}C NMR (75 MHz, $CDCl_3$)

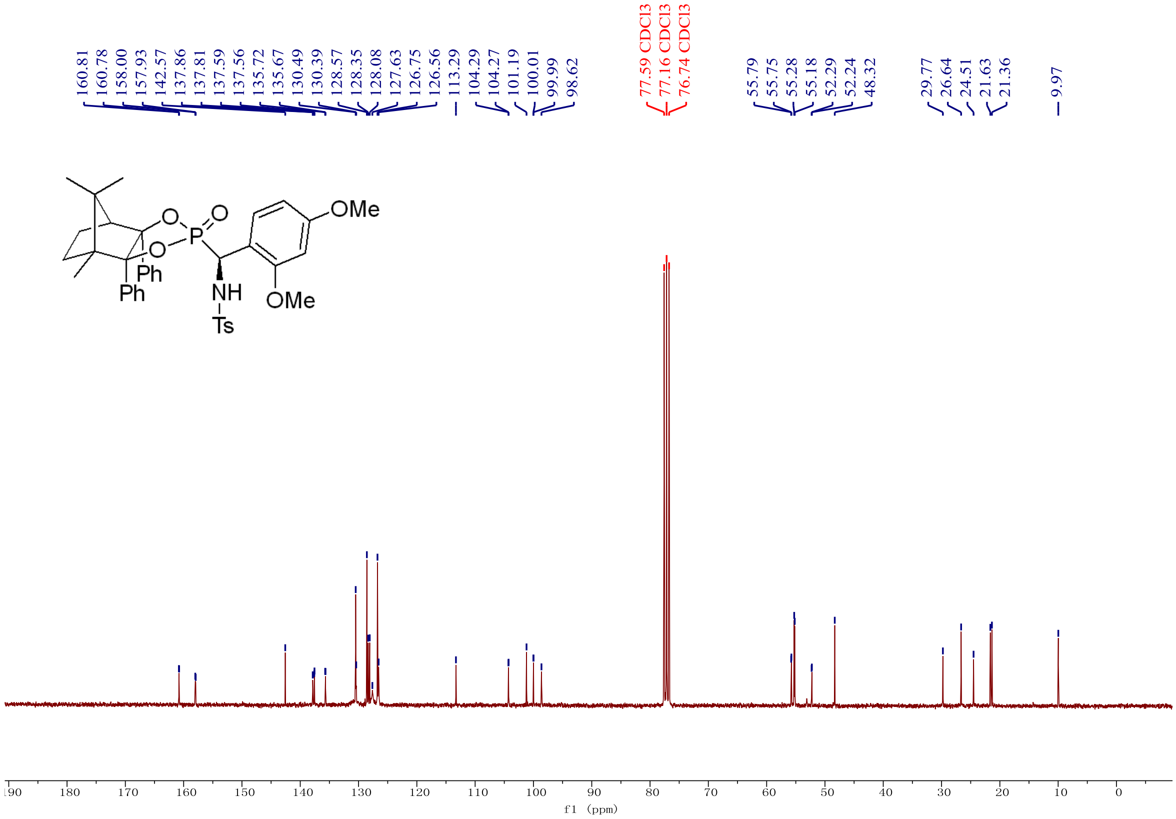

Fig. S170 ^{13}C NMR of compound **6p**

^{31}P NMR (121 MHz, $CDCl_3$)

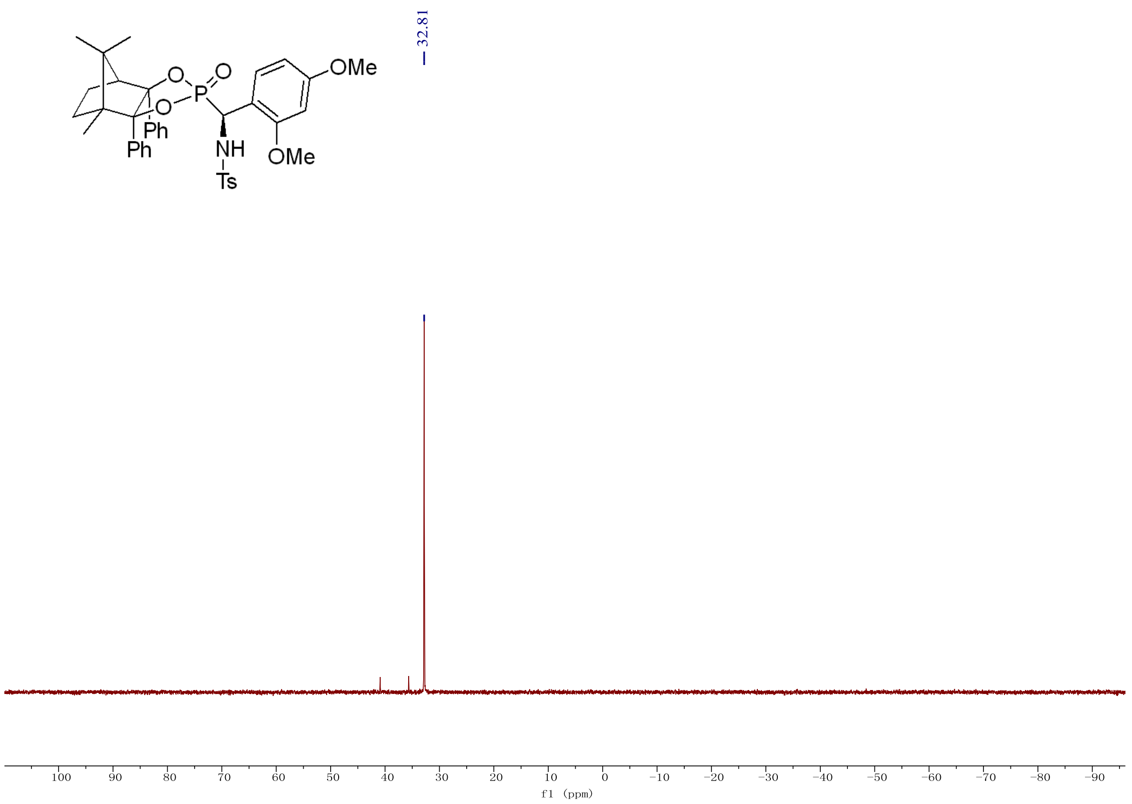

Fig. S171 ^{31}P NMR of compound **6p**

^{1}H NMR (300 MHz, $CDCl_3$)

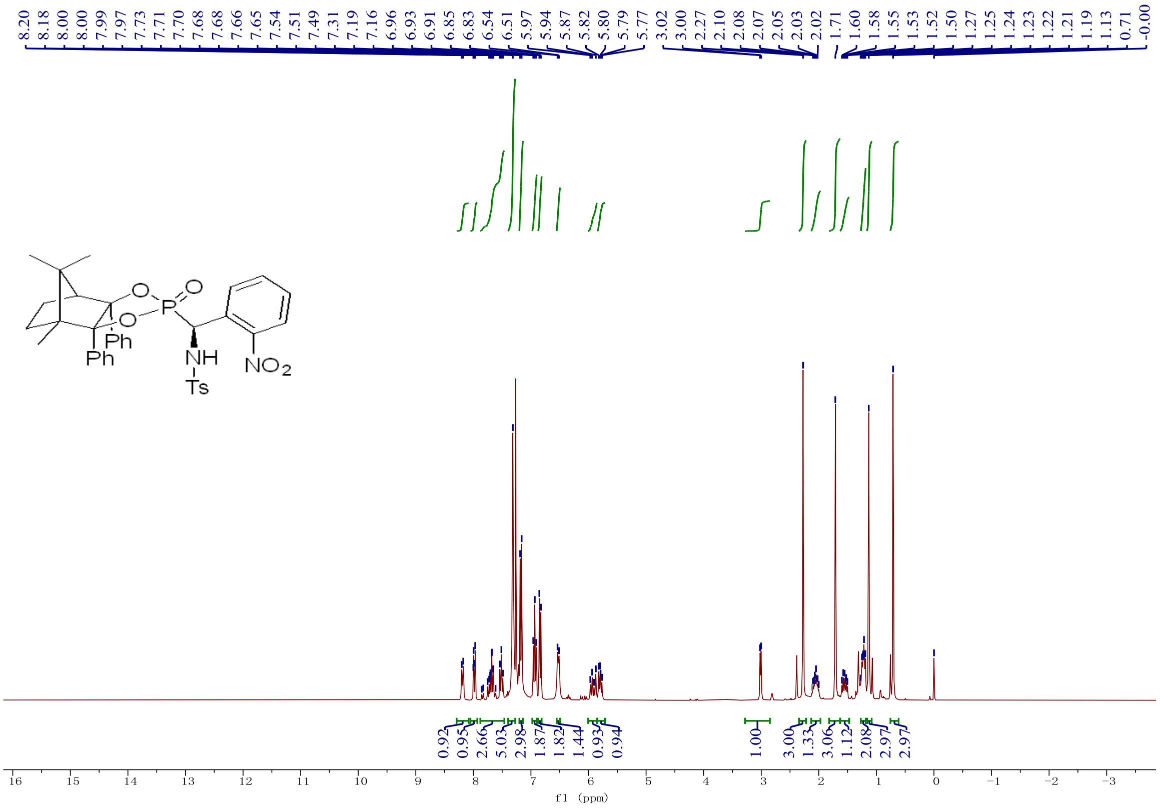

Fig. S172 ^{1}H NMR of compound **6q**

^{13}C NMR (75 MHz, $CDCl_3$)

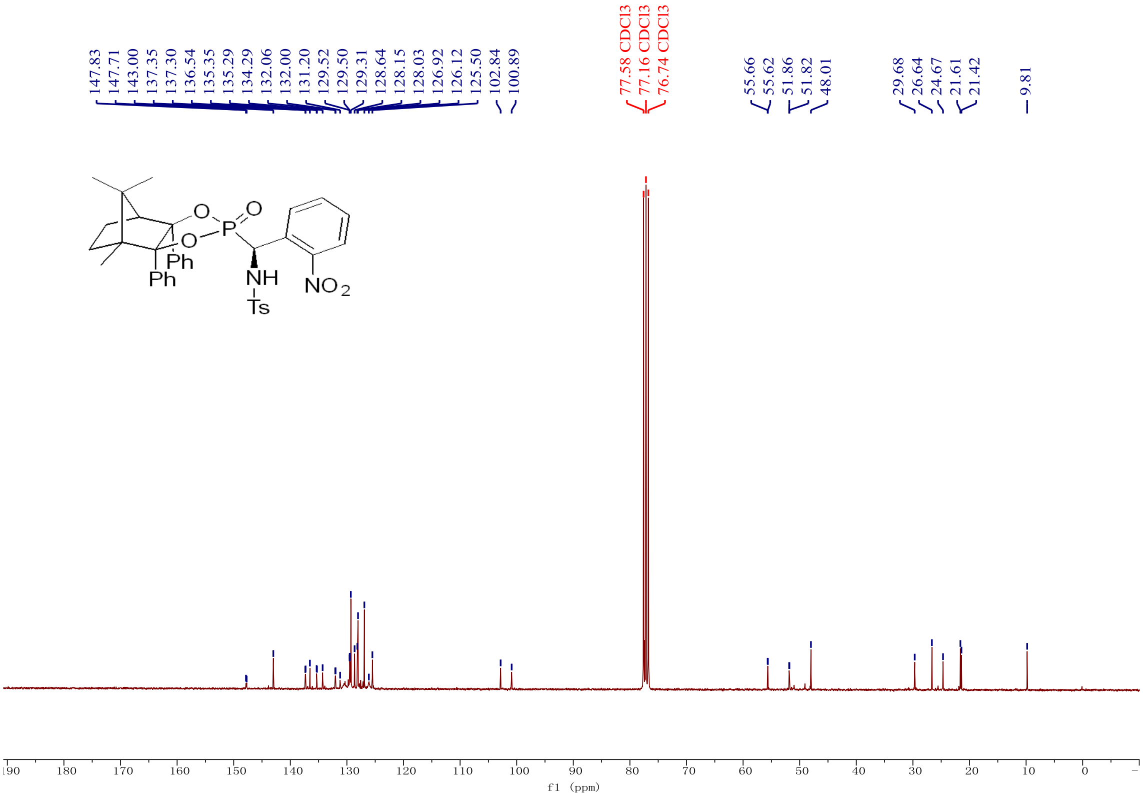

Fig. S173 ^{13}C NMR of compound **6q**

^{31}P NMR (121 MHz, $CDCl_3$)

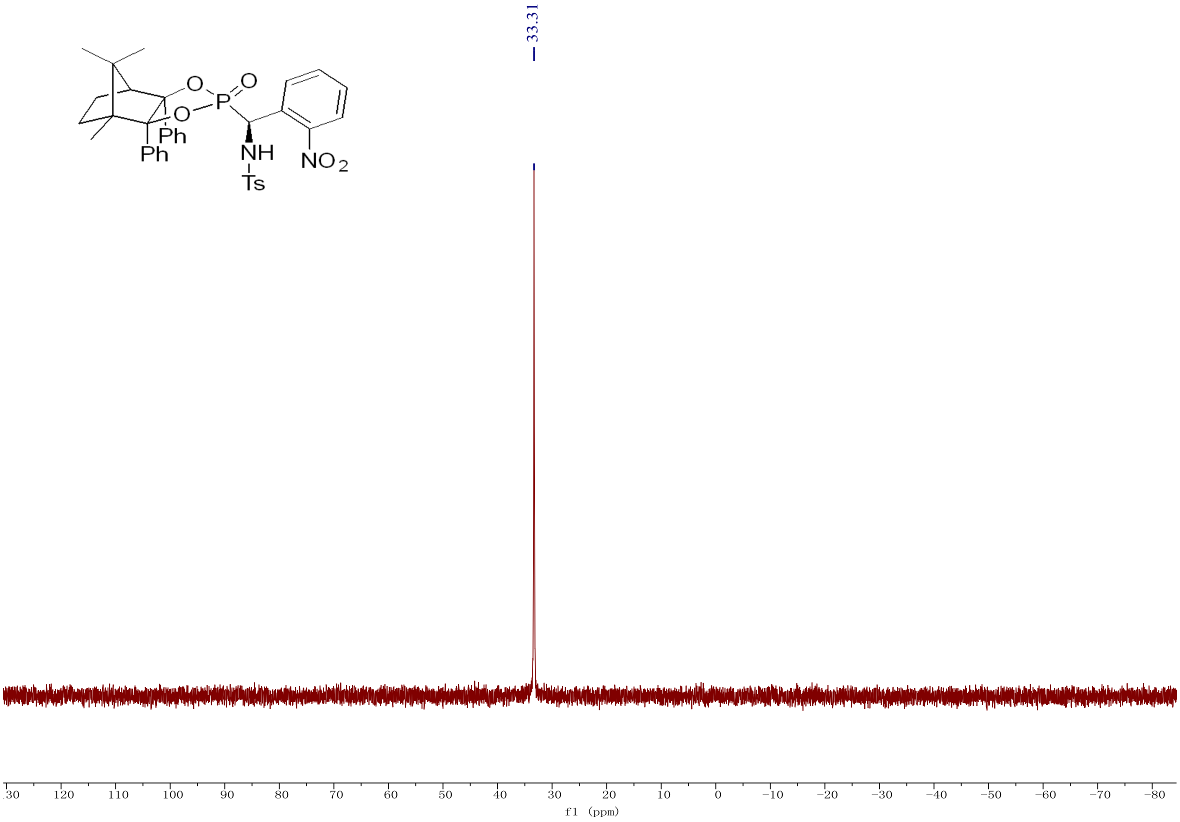

Fig. S174 ^{31}P NMR of compound **6q**

^{1}H NMR (300 MHz, $CDCl_3$)

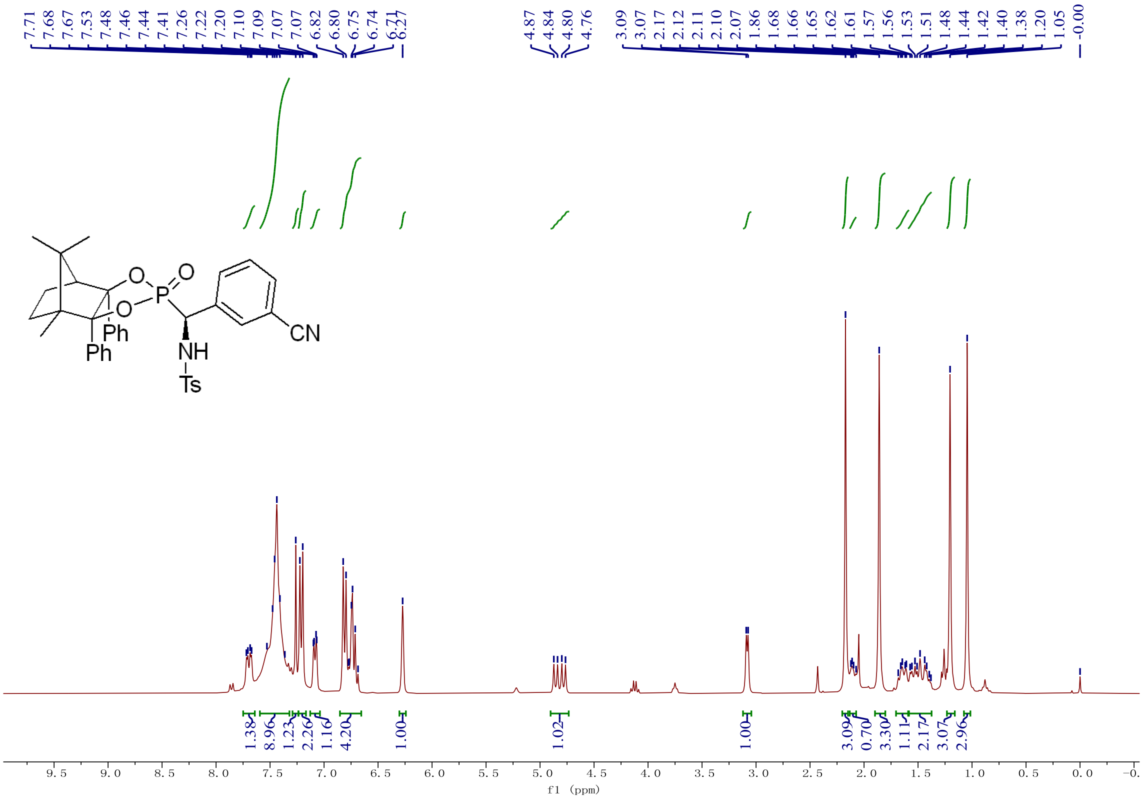

Fig. S175 ^{1}H NMR of compound **6r**

^{13}C NMR (75 MHz, $CDCl_3$)

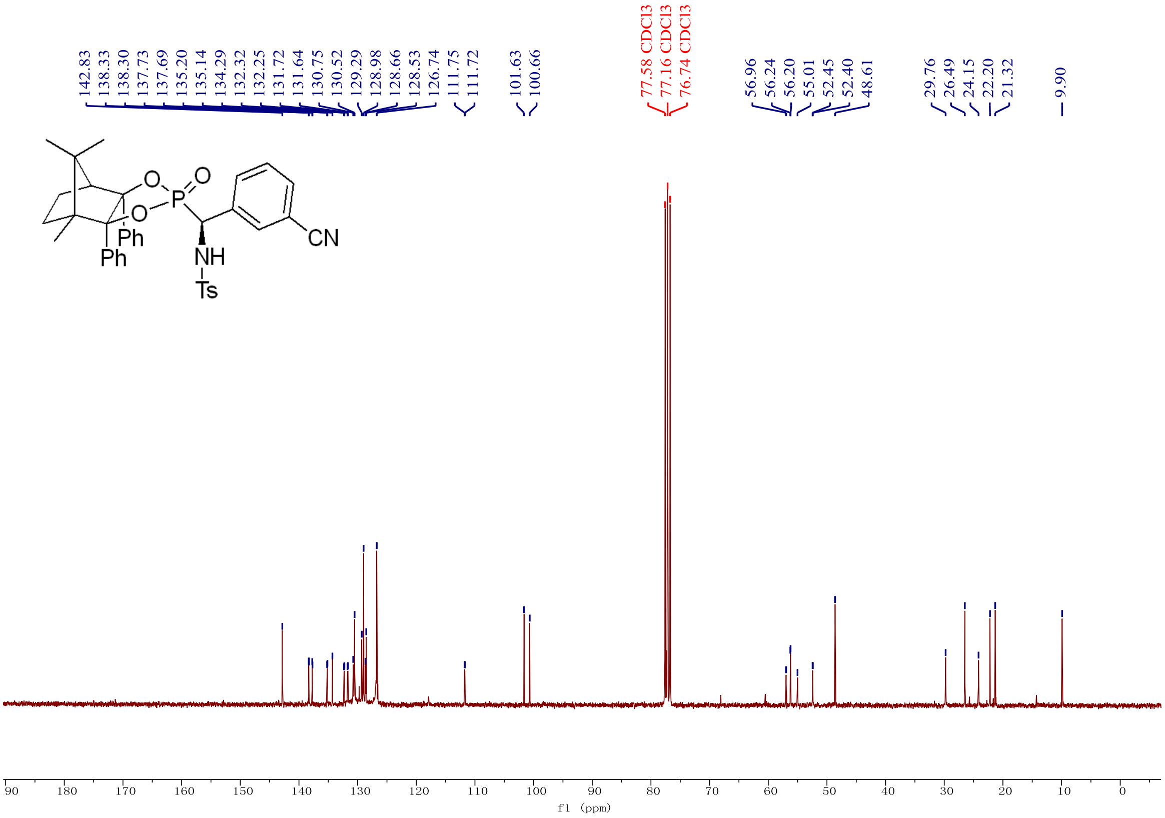

Fig. S176 ^{13}C NMR of compound **6r**

^{31}P NMR (121 MHz, $CDCl_3$)

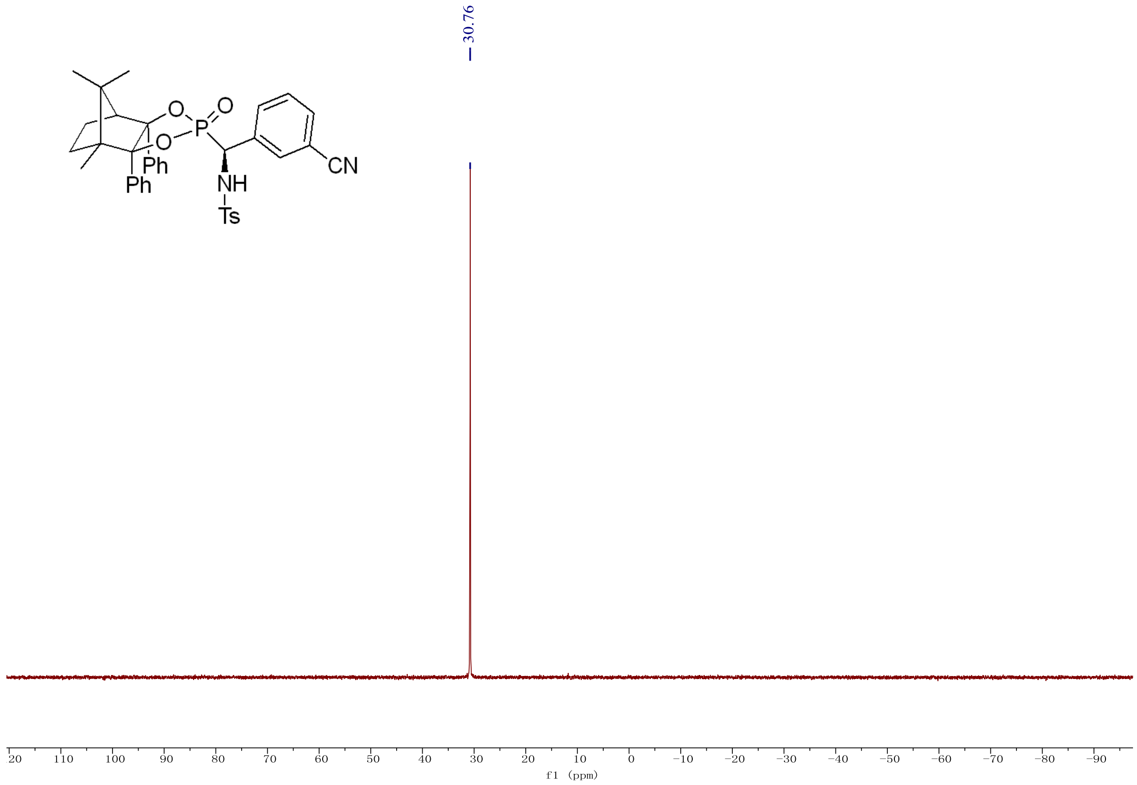

Fig. S177 ^{31}P NMR of compound **6r**

^{1}H NMR (300 MHz, $CDCl_3$)

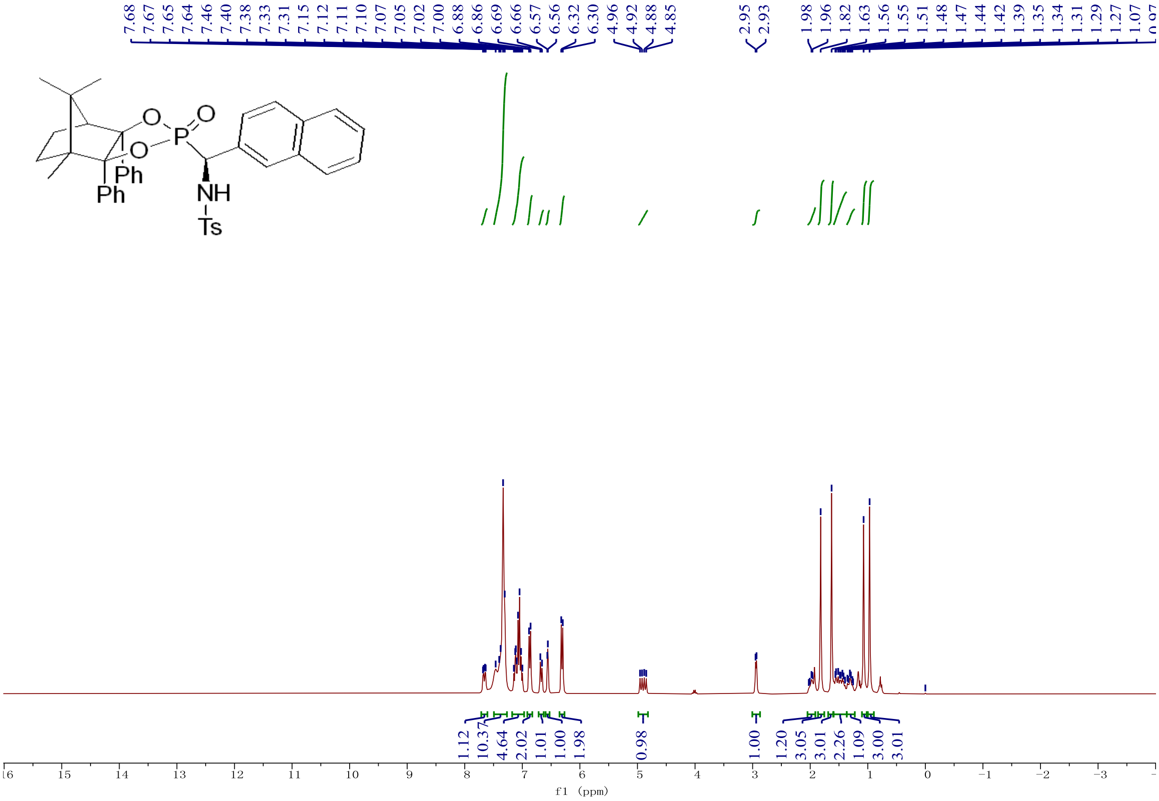

` Fig. S178 ^{1}H NMR of compound **6s**

^{13}C NMR (75 MHz, $CDCl_3$)

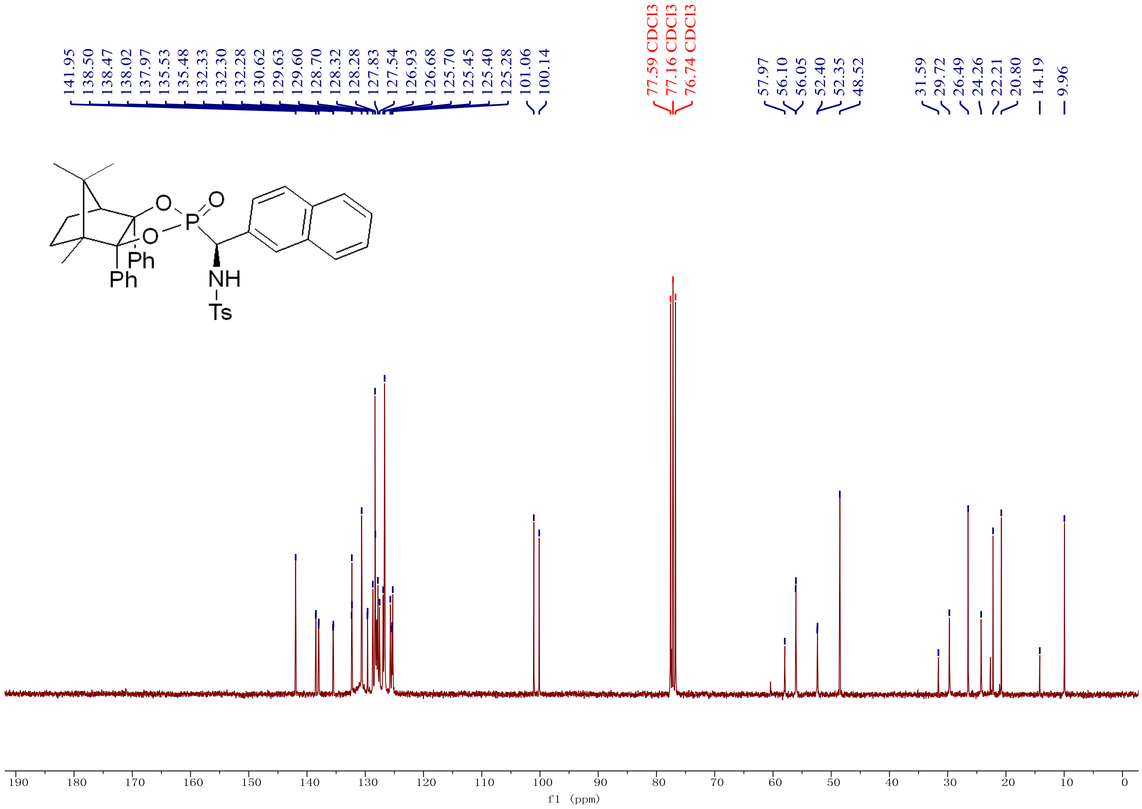

Fig. S179 ^{13}C NMR of compound **6s**

^{31}P NMR (121 MHz, $CDCl_3$)

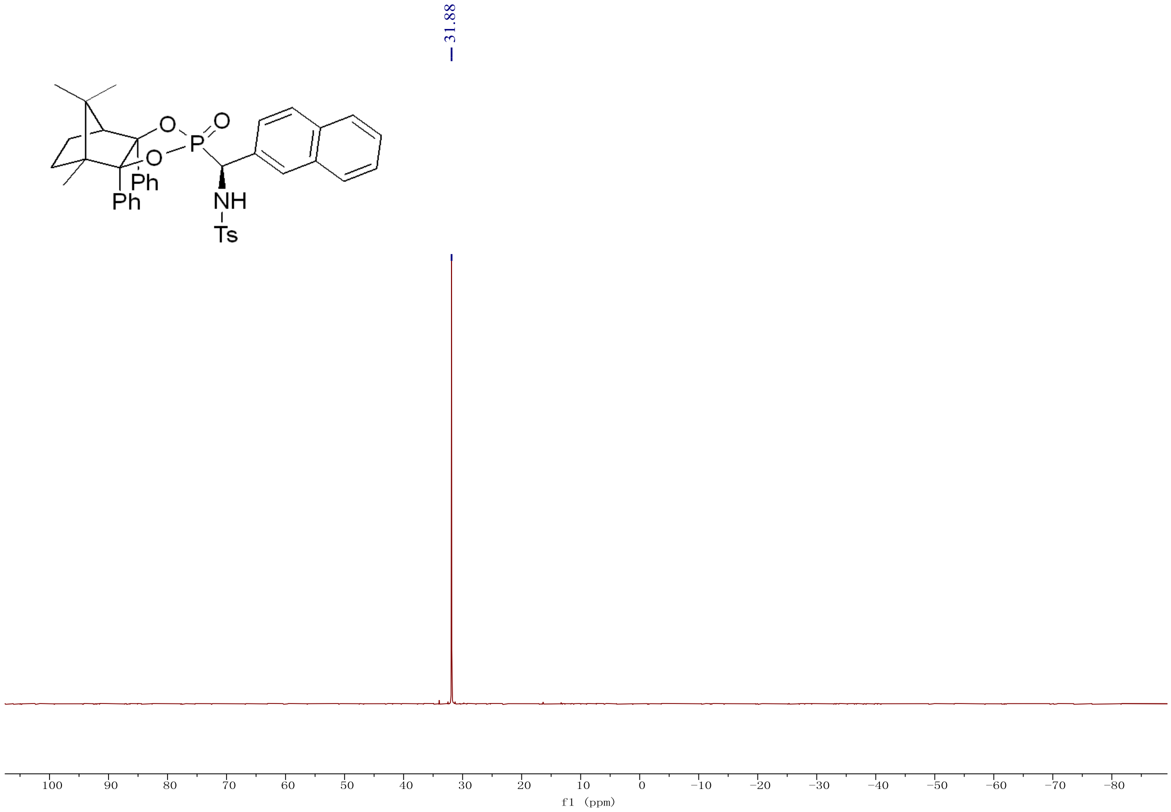

Fig. S180 ^{31}P NMR of compound **6s**

^{1}H NMR (300 MHz, $CDCl_3$)

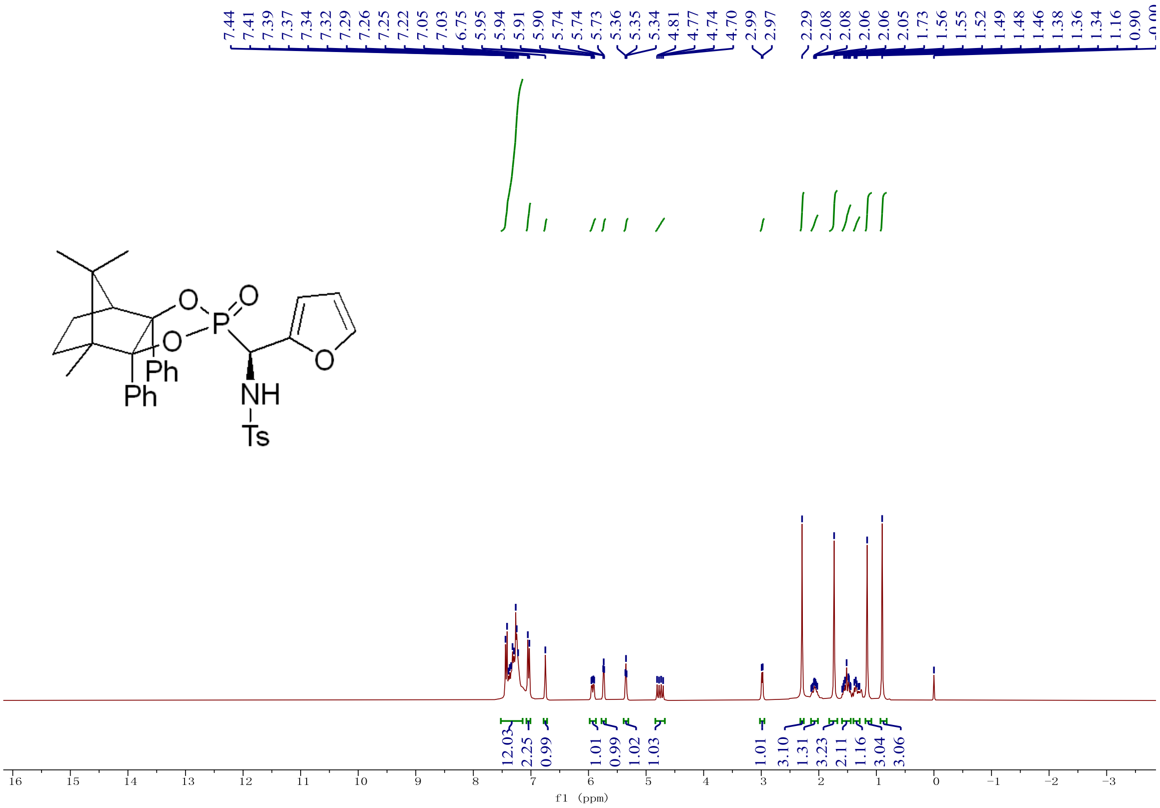

Fig. S181 ^{1}H NMR of compound **6t**

^{13}C NMR (75 MHz, $CDCl_3$)

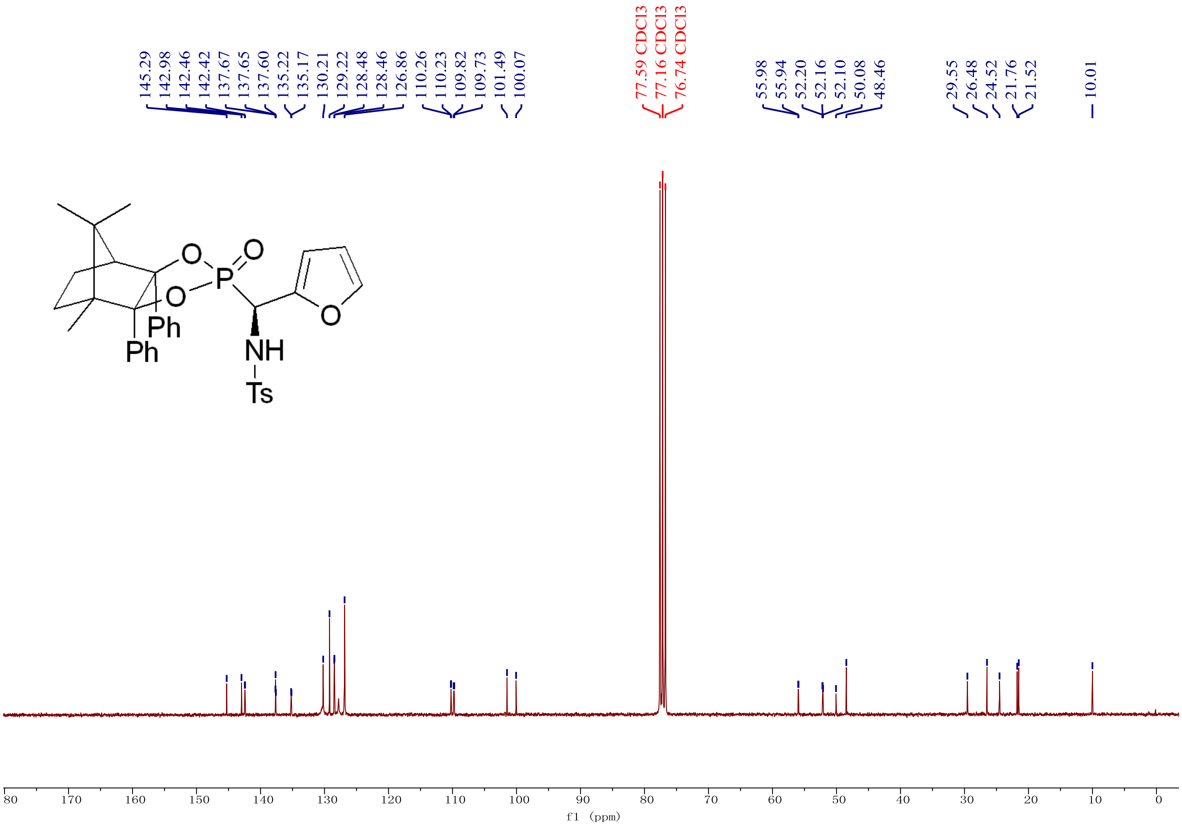

Fig. S182 ^{13}C NMR of compound **6t**

^{31}P NMR (121 MHz, $CDCl_3$)

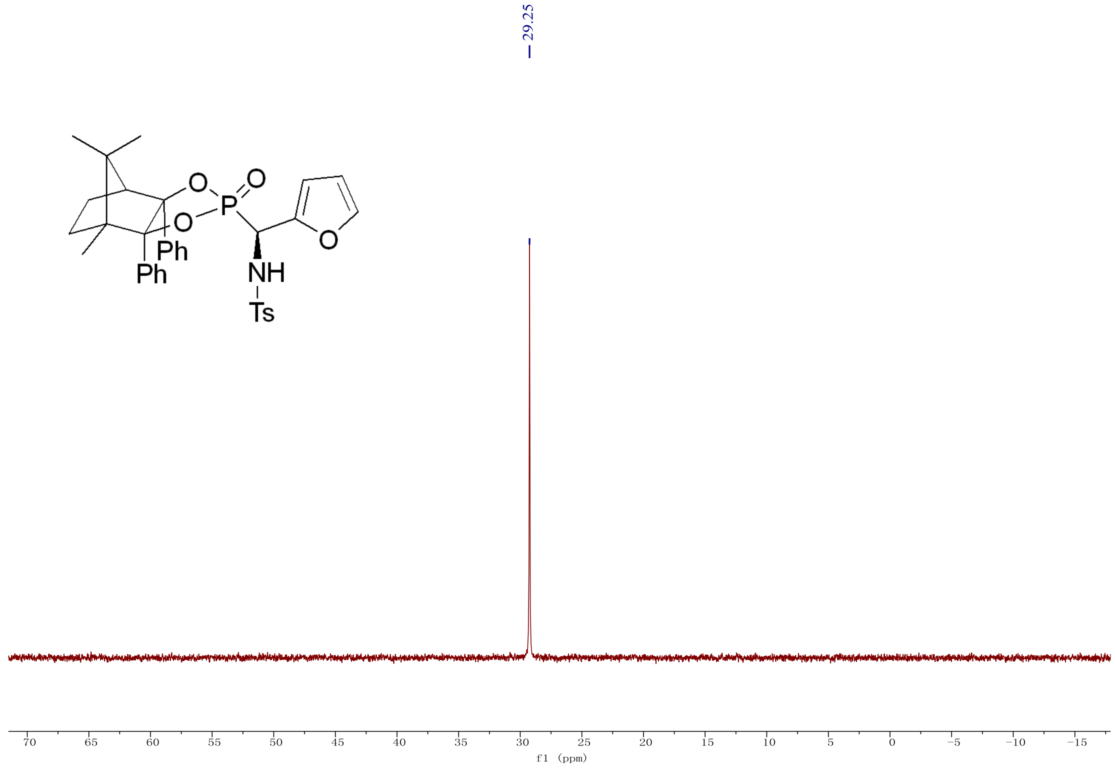

Fig. S183 ^{31}P NMR of compound **6t**

^{1}H NMR (300 MHz, $CDCl_3$)

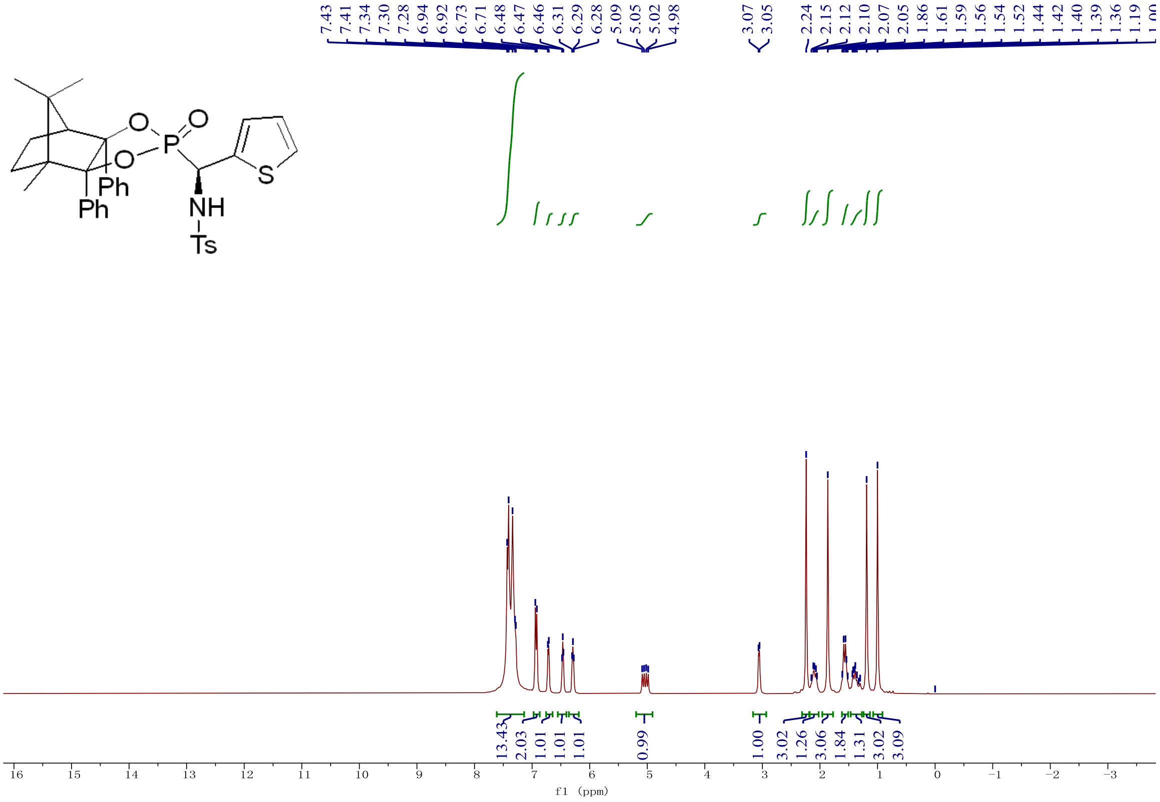

Fig. S184 ^{1}H NMR of compound **6u**

^{13}C NMR (75 MHz, $CDCl_3$)

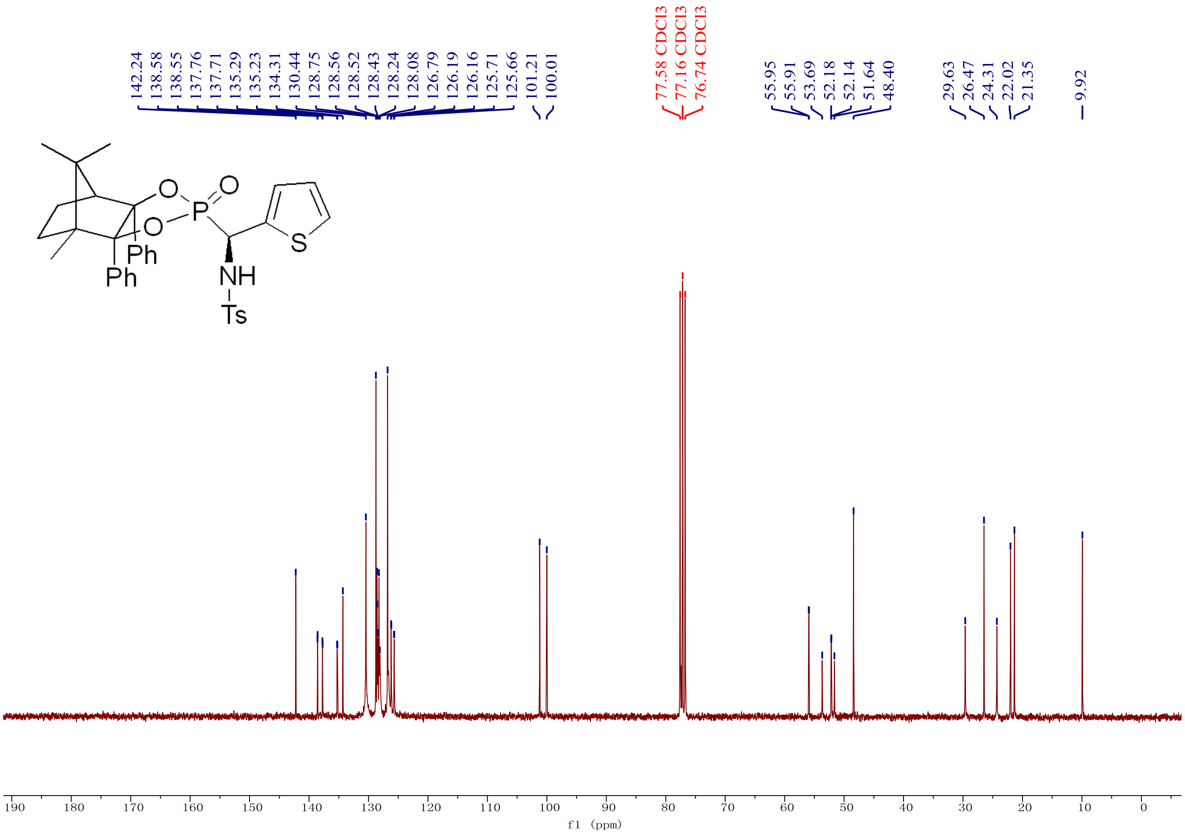

Fig. S185 ^{13}C NMR of compound **6u**

^{31}P NMR (121 MHz, $CDCl_3$)

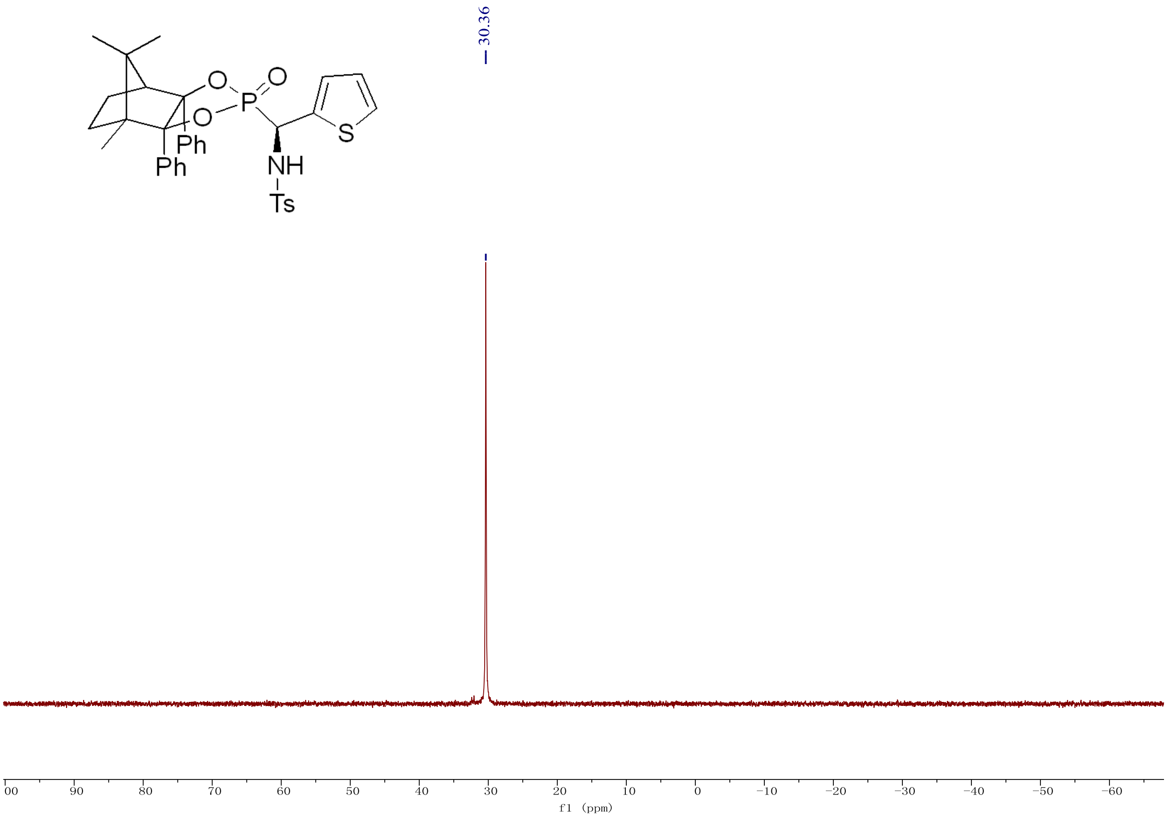

Fig. S186 ^{31}P NMR of compound **6u**

^{1}H NMR (300 MHz, $CDCl_3$)

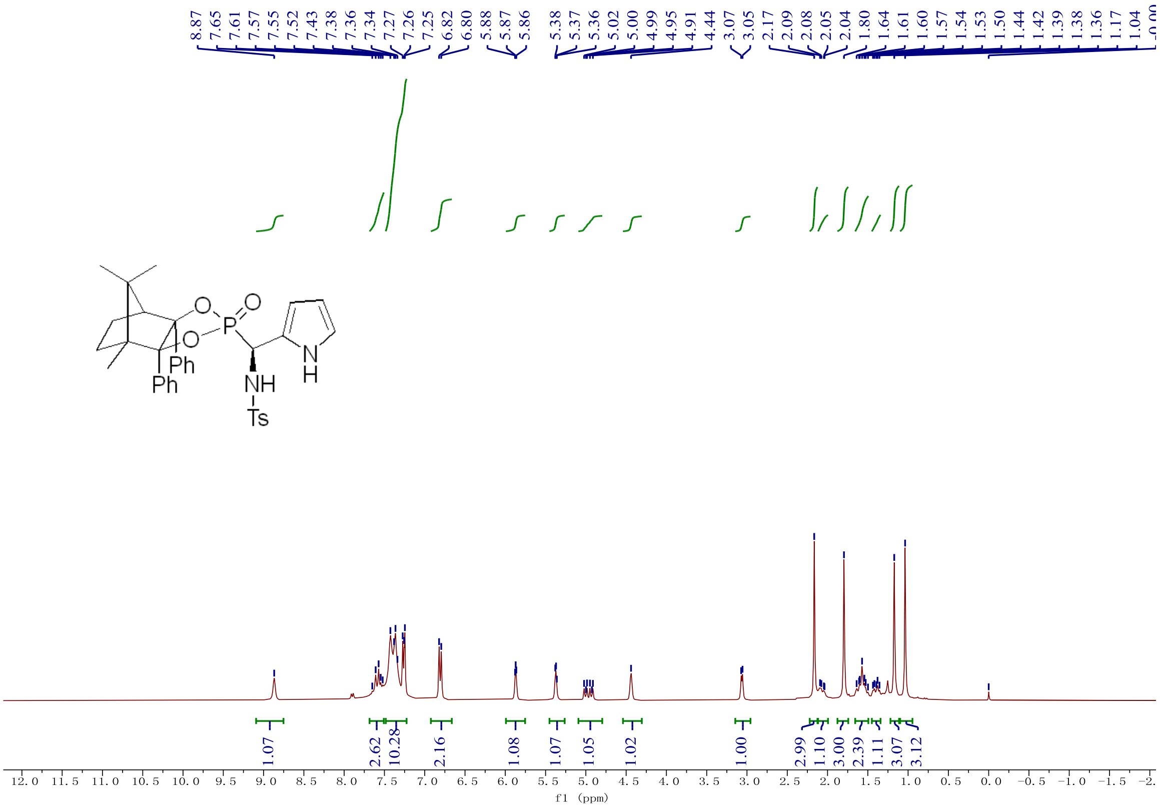

Fig. S187 ^{1}H NMR of compound **6v**

^{13}C NMR (75 MHz, $CDCl_3$)

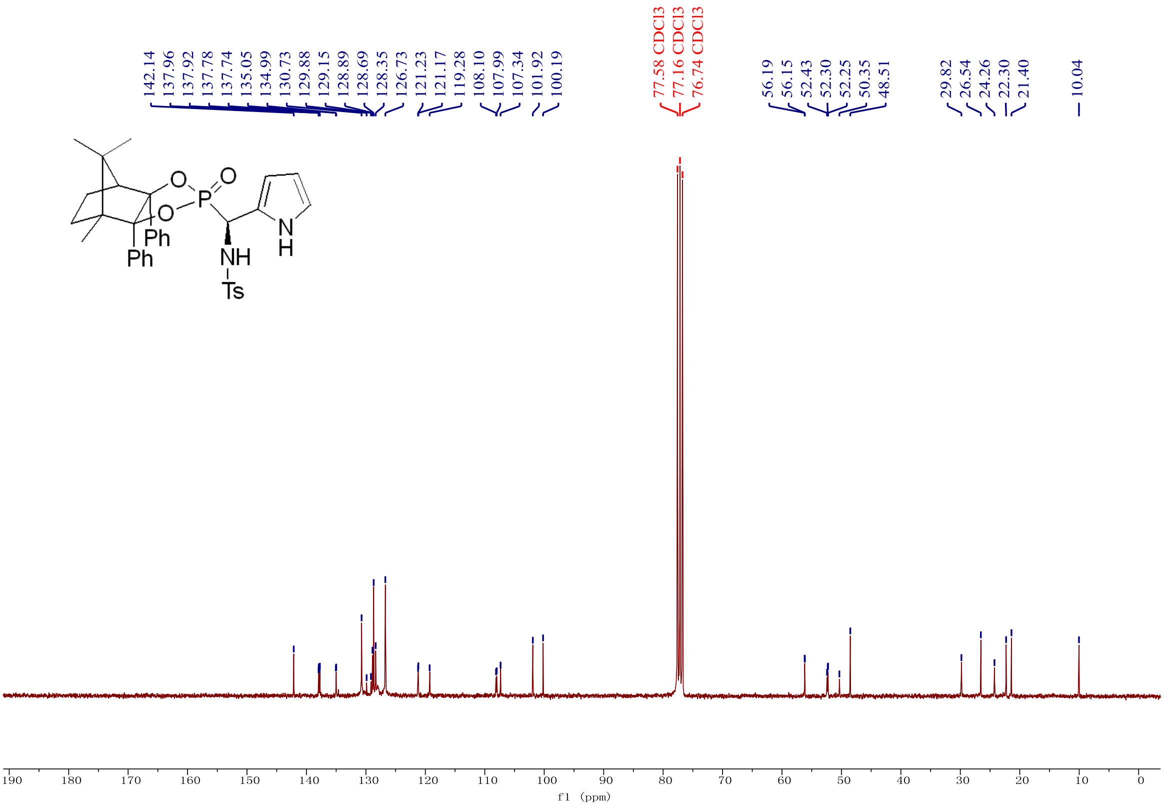

Fig. S188 ^{13}C NMR of compound **6v**

^{31}P NMR (121 MHz, $CDCl_3$)

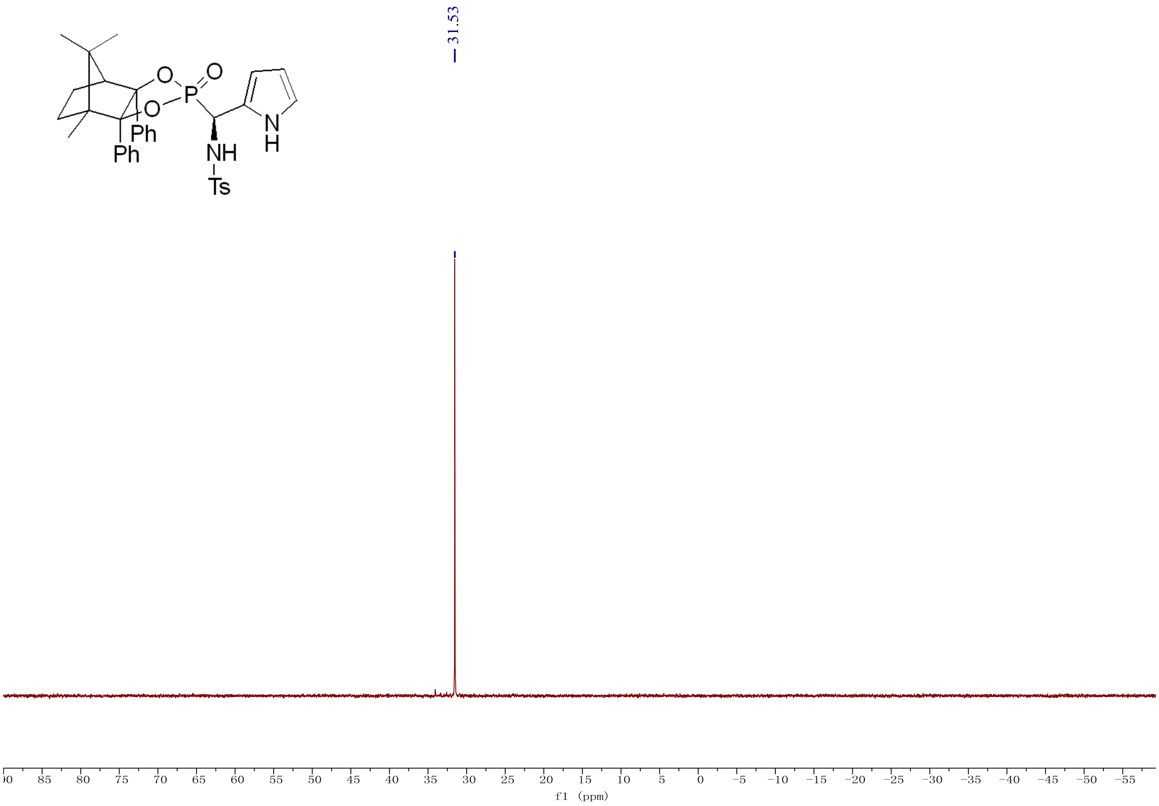

Fig. S189 ^{31}P NMR of compound **6v**

^{1}H NMR (300 MHz, $CDCl_3$)

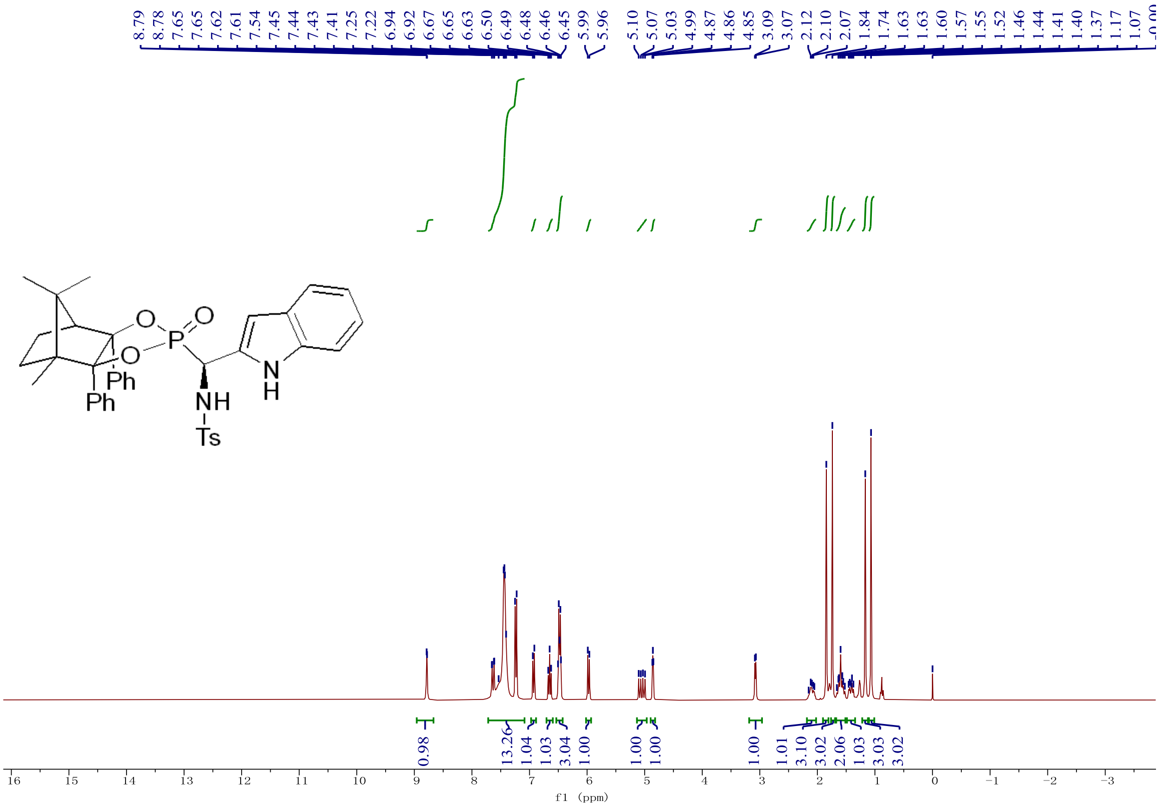

Fig. S190 ^{1}H NMR of compound **6w**

^{13}C NMR (75 MHz, $CDCl_3$)

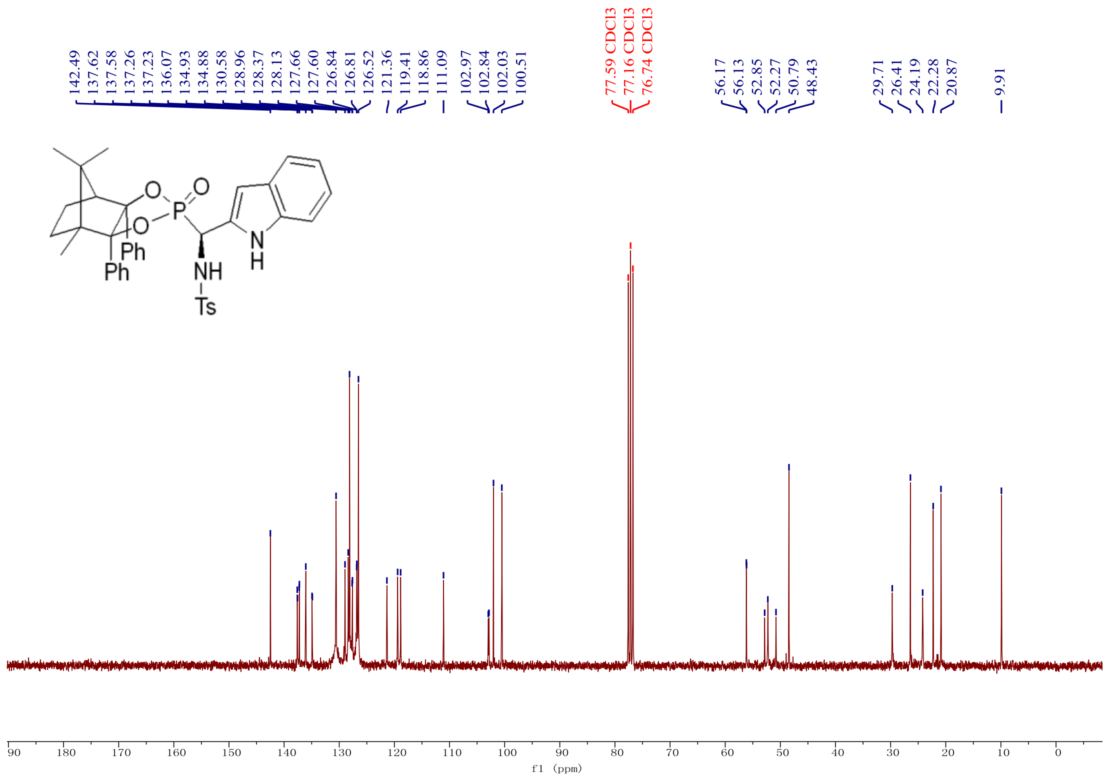

Fig. S191 ^{13}C NMR of compound **6w**

^{31}P NMR (121 MHz, $CDCl_3$)

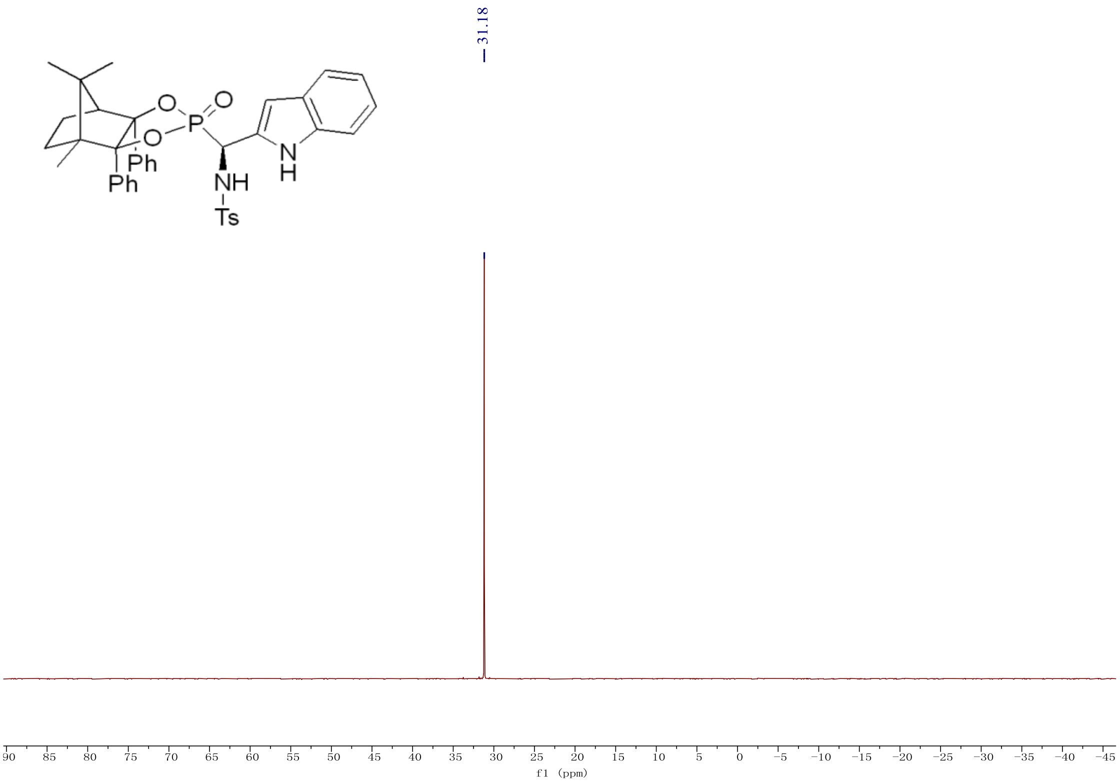

Fig. S192 ^{31}P NMR of compound **6w**

^{1}H NMR (300 MHz, $CDCl_3$)

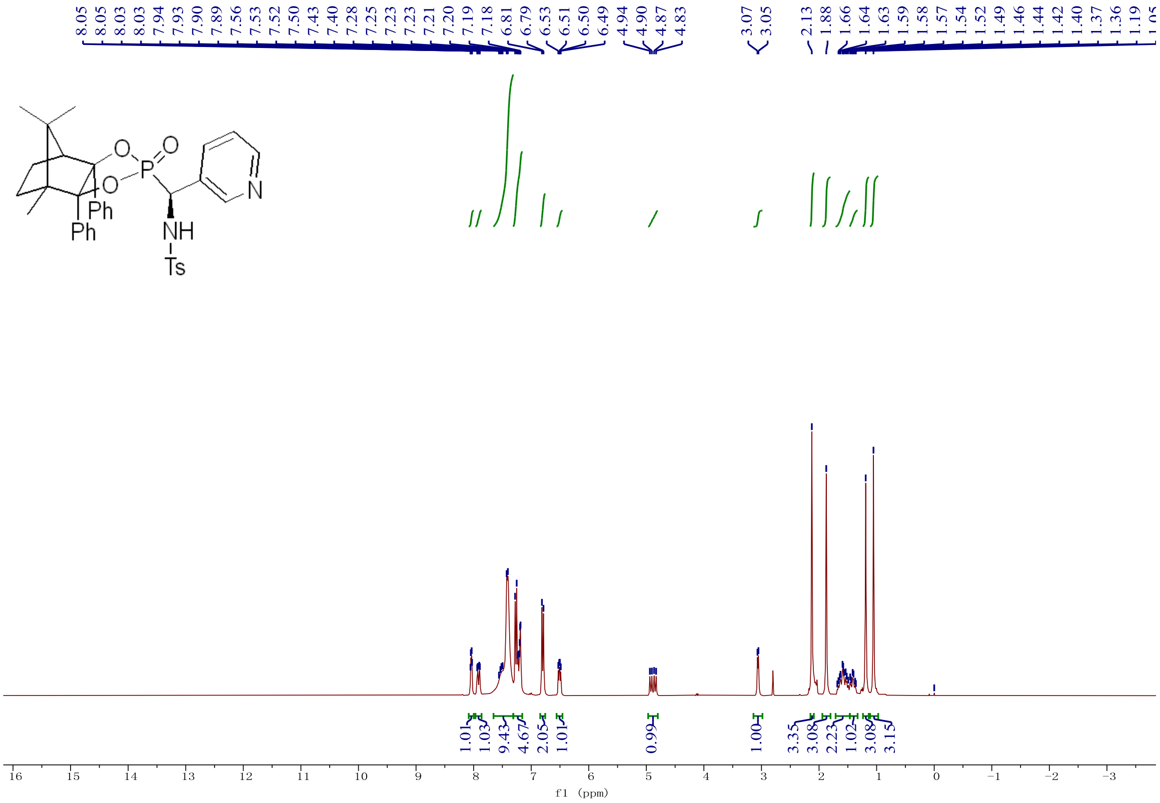

Fig. S193 ^{1}H NMR of compound **6x**

^{13}C NMR (75 MHz, $CDCl_3$)

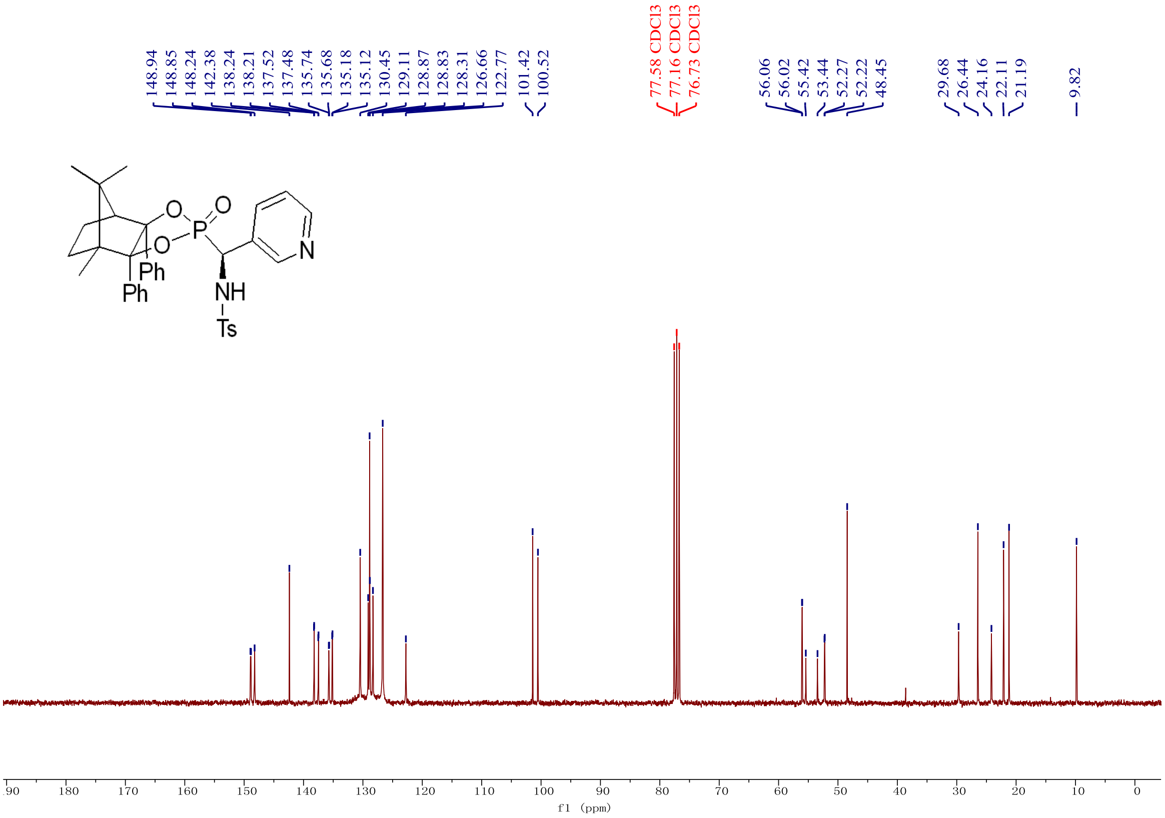

Fig. S194 ^{13}C NMR of compound **6x**

^{31}P NMR (121 MHz, $CDCl_3$)

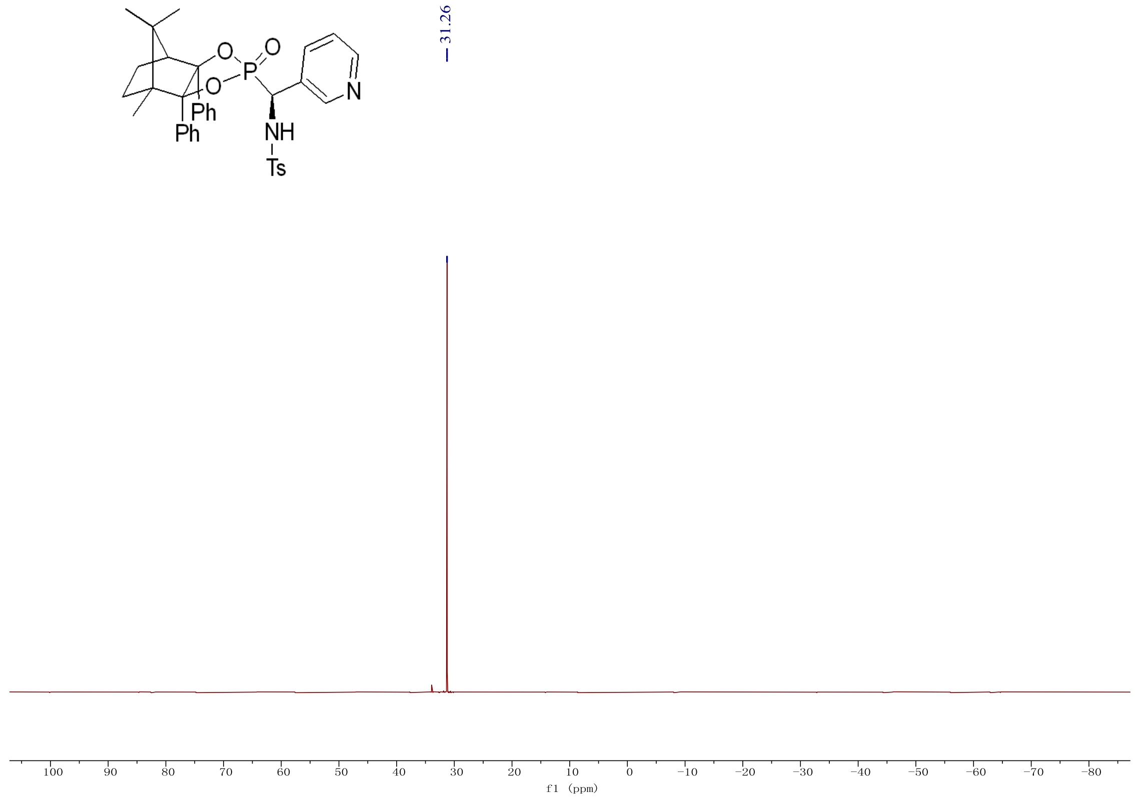

Fig. S195 ^{31}P NMR of compound **6x**

^{1}H NMR (300 MHz, $CDCl_3$)

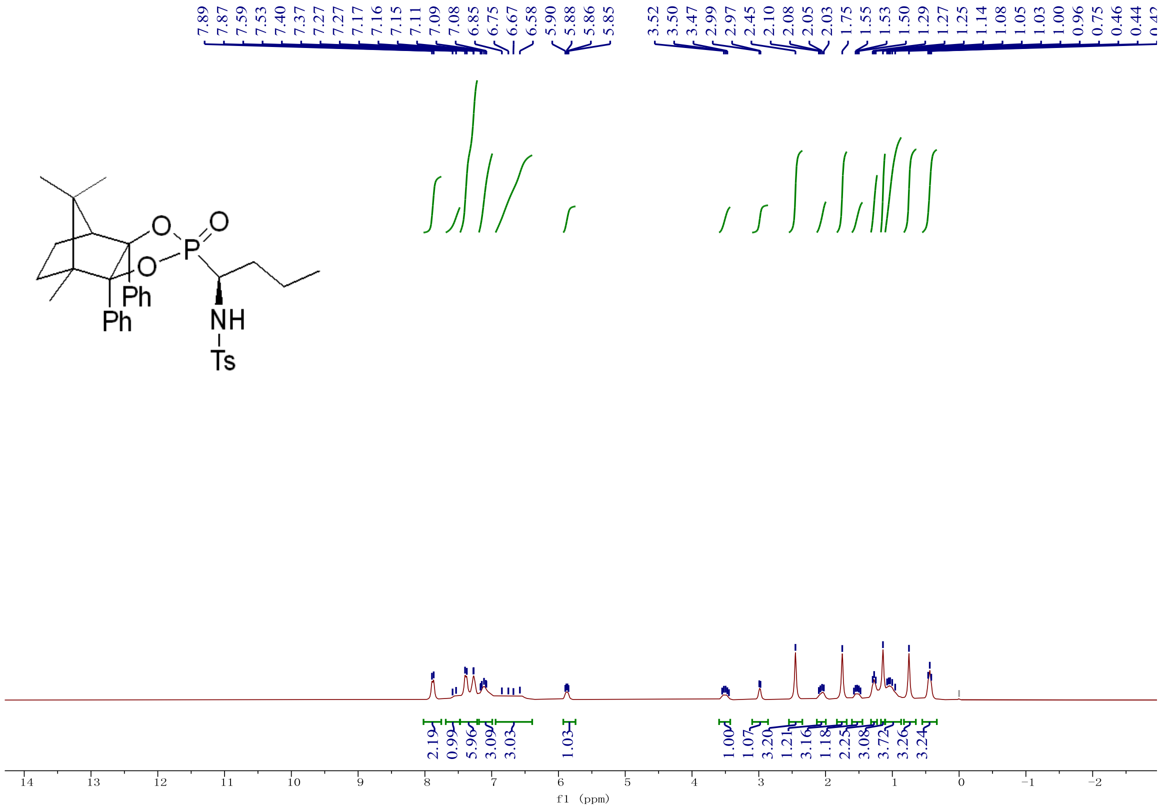

Fig. S196 ^{1}H NMR of compound **6ya**

^{13}C NMR (75 MHz, $CDCl_3$)

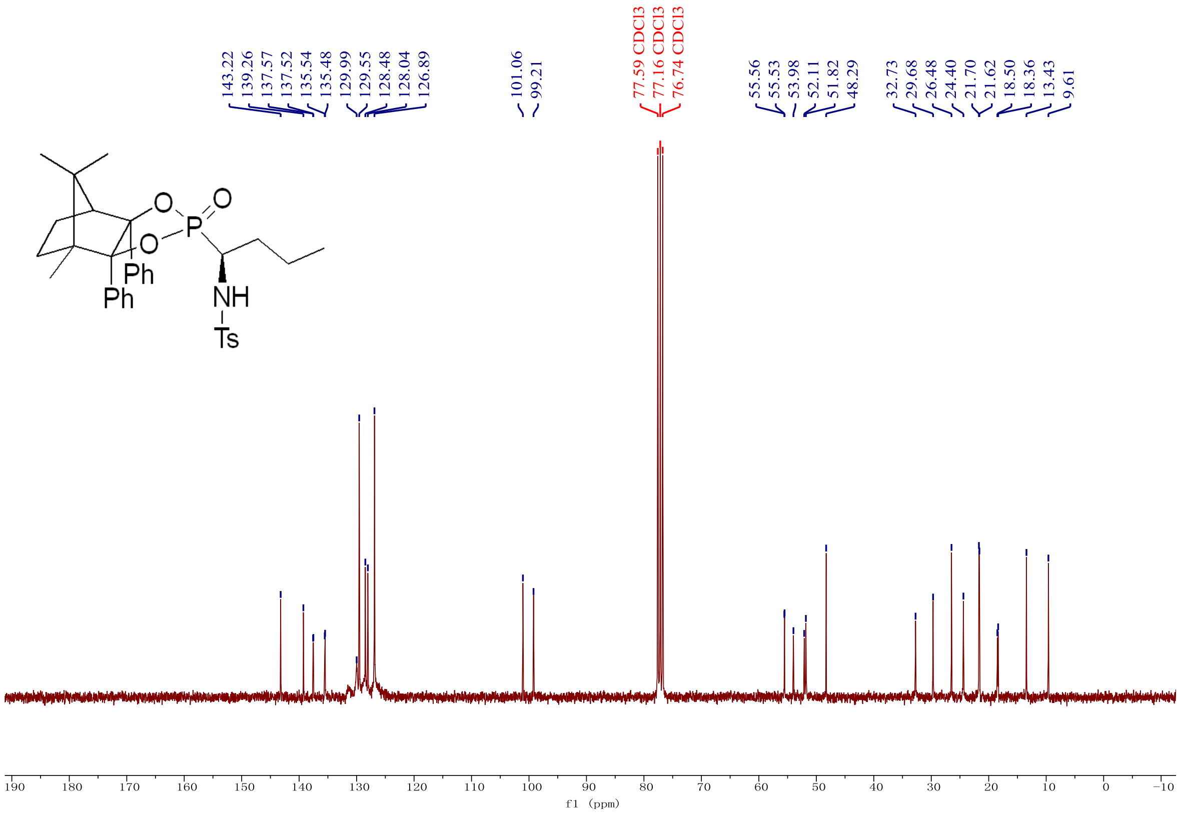

Fig. S197 ^{13}C NMR of compound **6ya**

^{31}P NMR (121 MHz, $CDCl_3$)

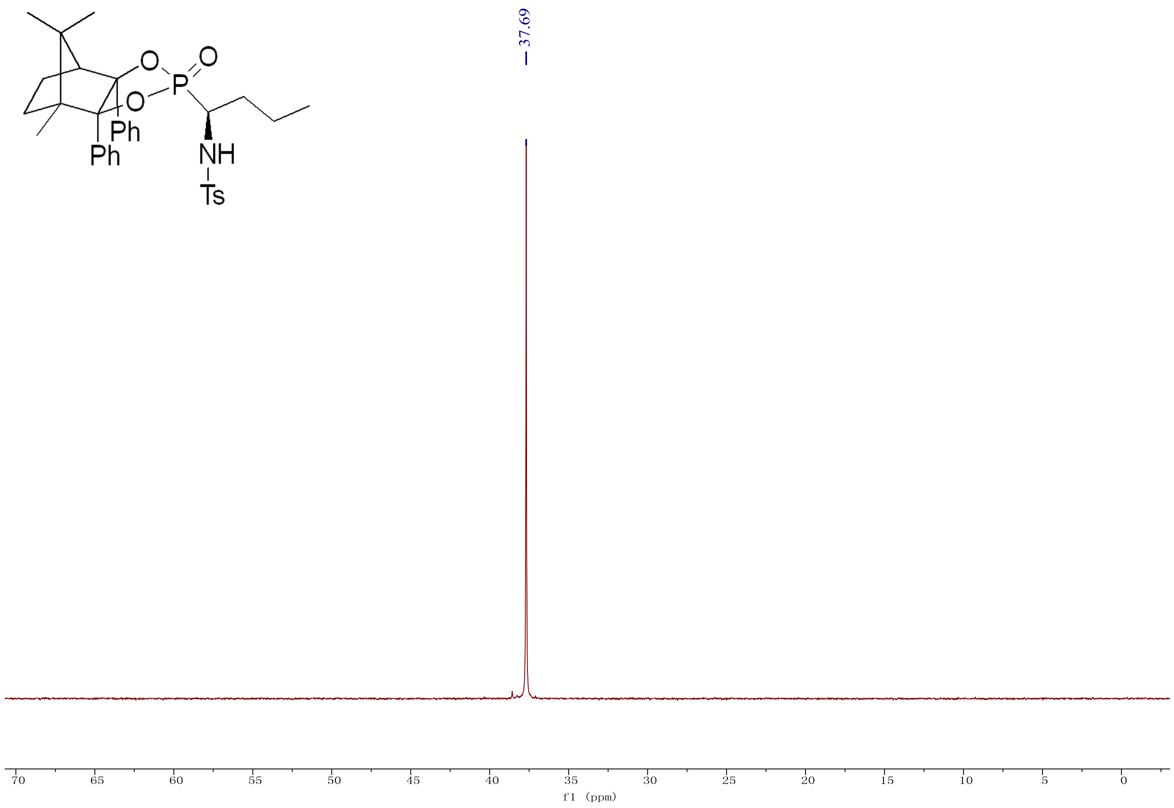

Fig. S198 ^{31}P NMR of compound **6ya**

^{1}H NMR (300 MHz, $CDCl_3$)

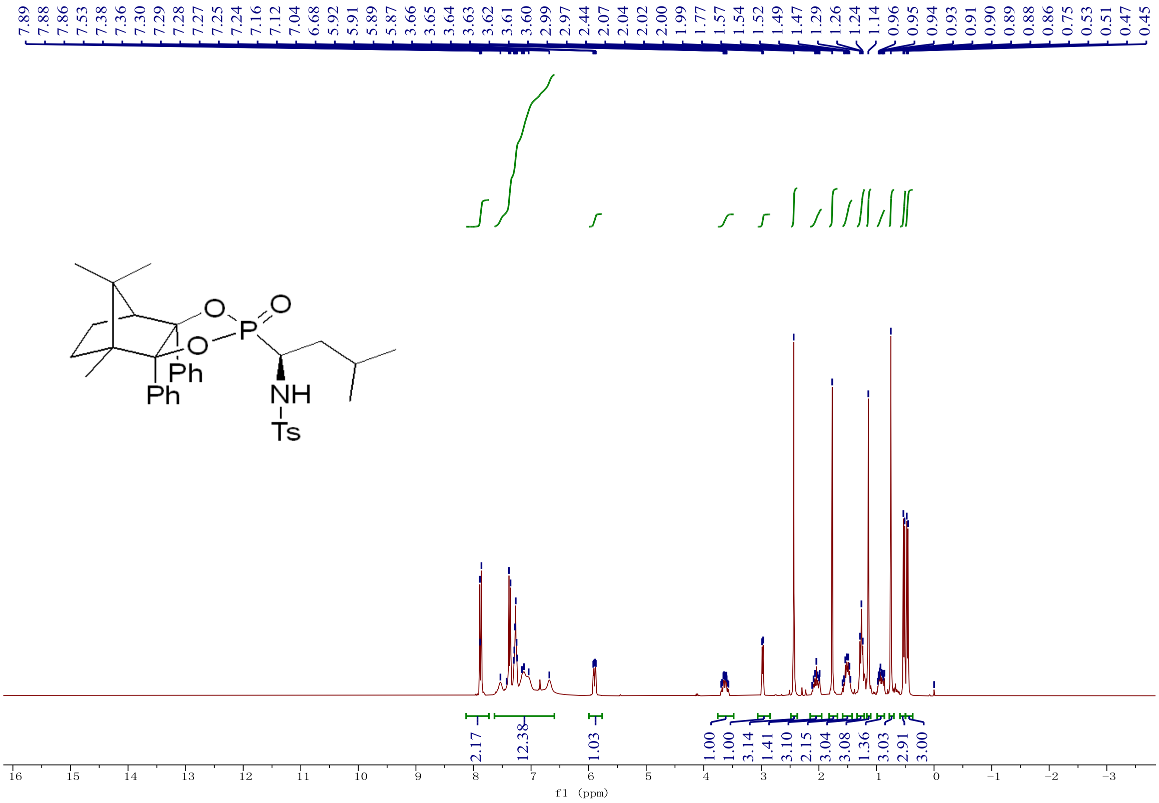

Fig. S199 ^{1}H NMR of compound **6yb**

^{13}C NMR (75 MHz, $CDCl_3$)

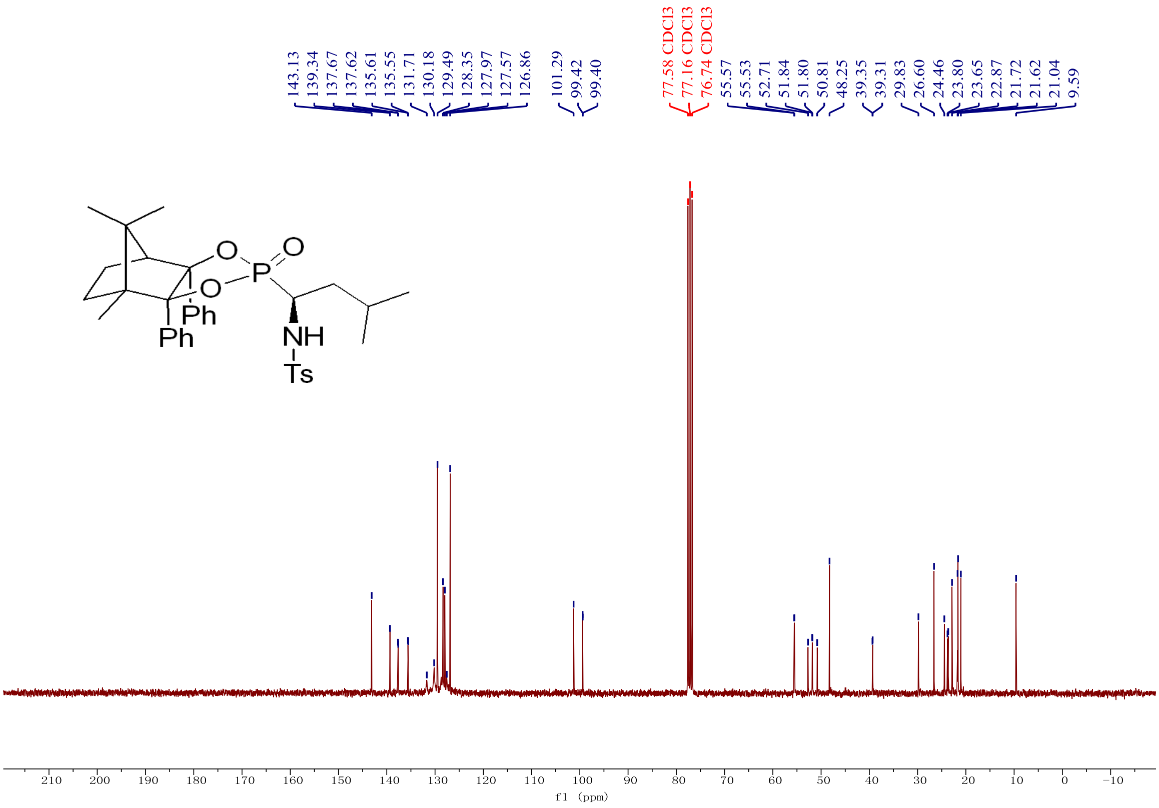

Fig. S200 ^{13}C NMR of compound **6yb**

^{31}P NMR (121 MHz, $CDCl_3$)

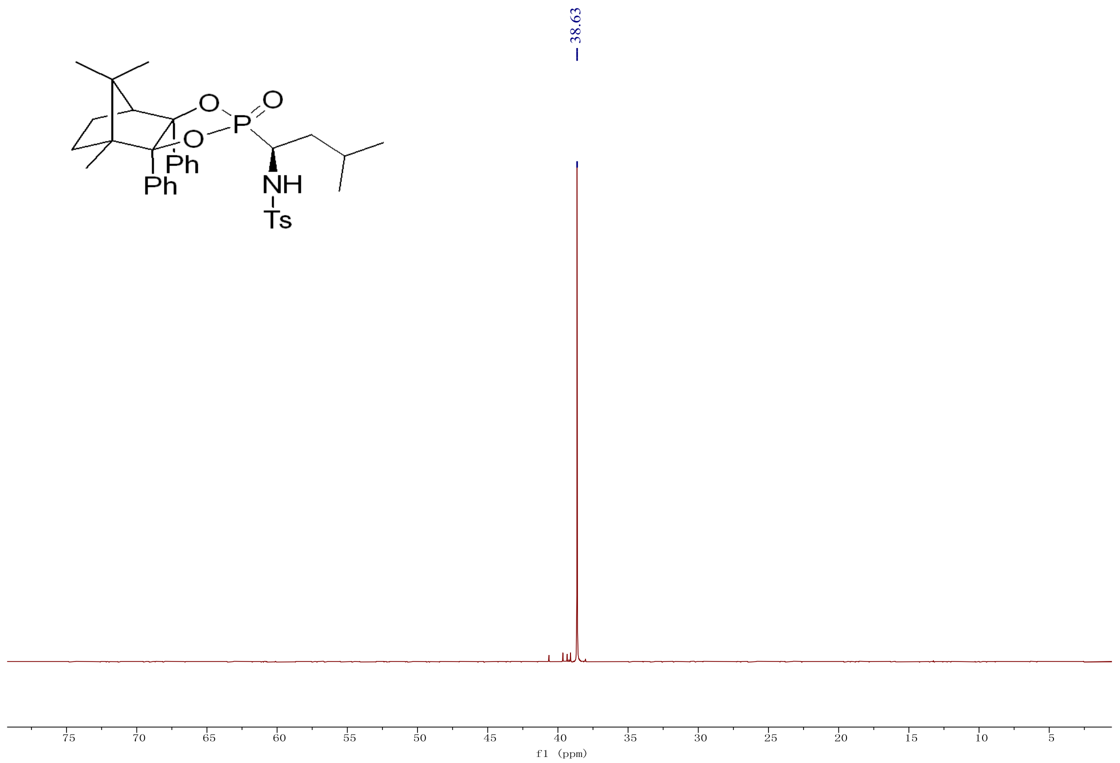

Fig. S201 ^{31}P NMR of compound **6yb**

^{1}H NMR (300 MHz, $CDCl_3$)

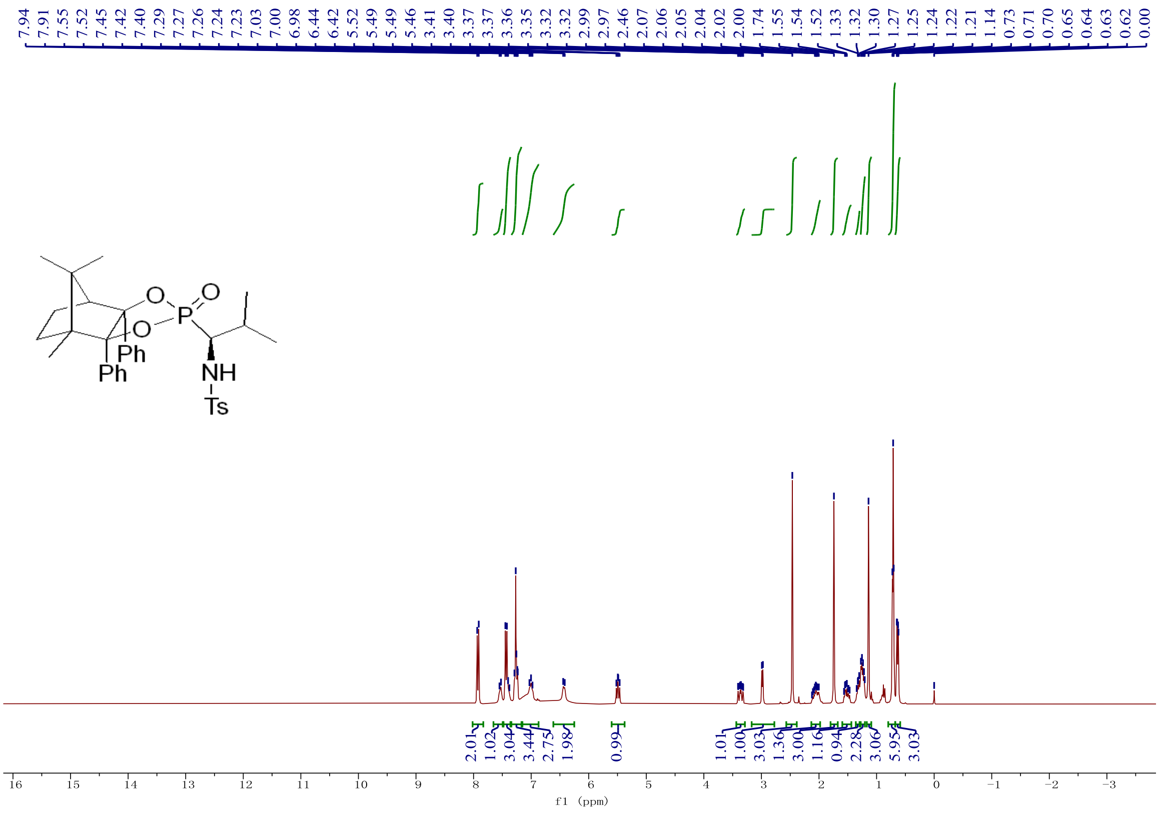

Fig. S202 ^{1}H NMR of compound **6yc**

^{13}C NMR (75 MHz, $CDCl_3$)

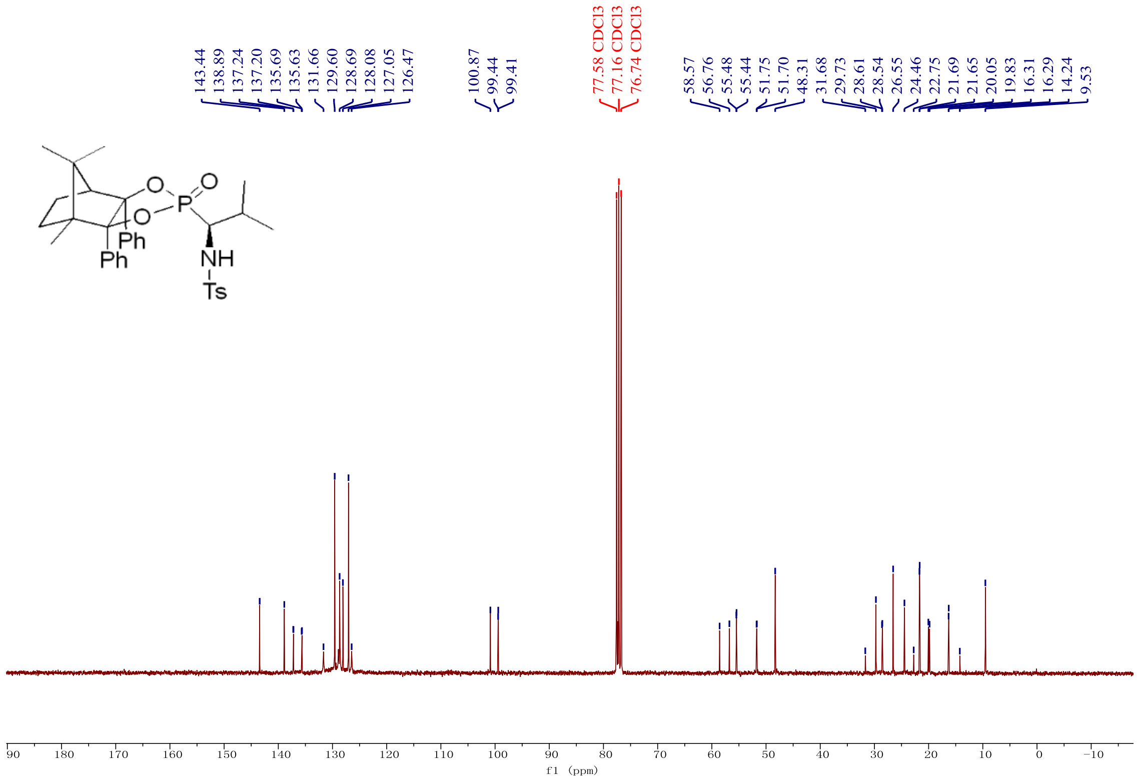

Fig. S203 ^{13}C NMR of compound **6yc**

^{31}P NMR (121 MHz, $CDCl_3$)

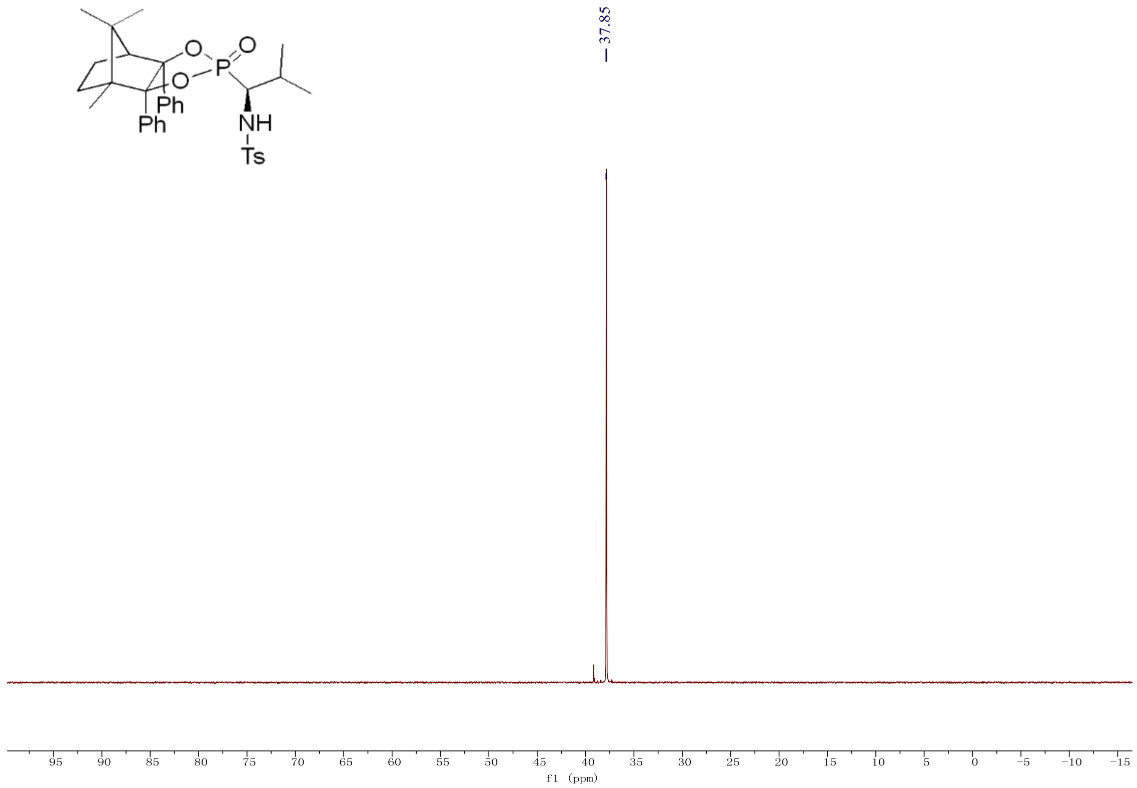

Fig. S204 ^{31}P NMR of compound **6yc**

^{1}H NMR (300 MHz, $CDCl_3$)

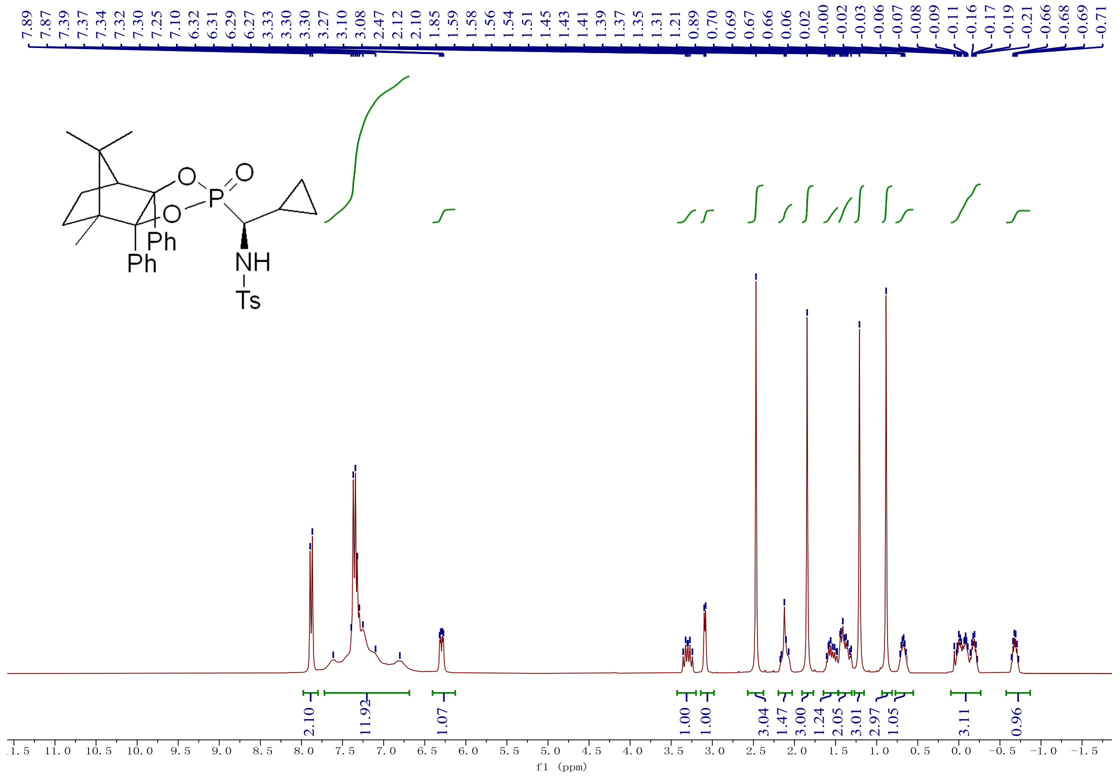

Fig. S205 ^{1}H NMR of compound **6yd**

^{13}C NMR (75 MHz, $CDCl_3$)

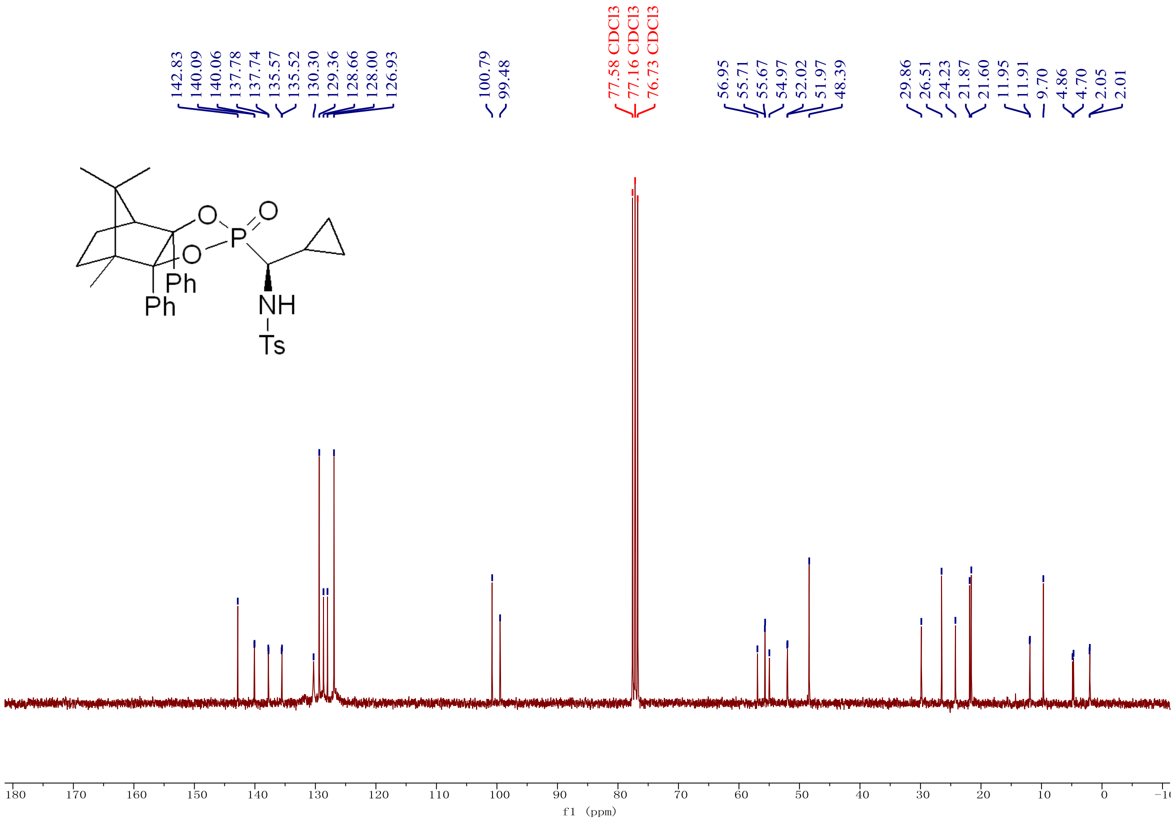

Fig. S206 ^{13}C NMR of compound **6yd**

^{31}P NMR (121 MHz, $CDCl_3$)

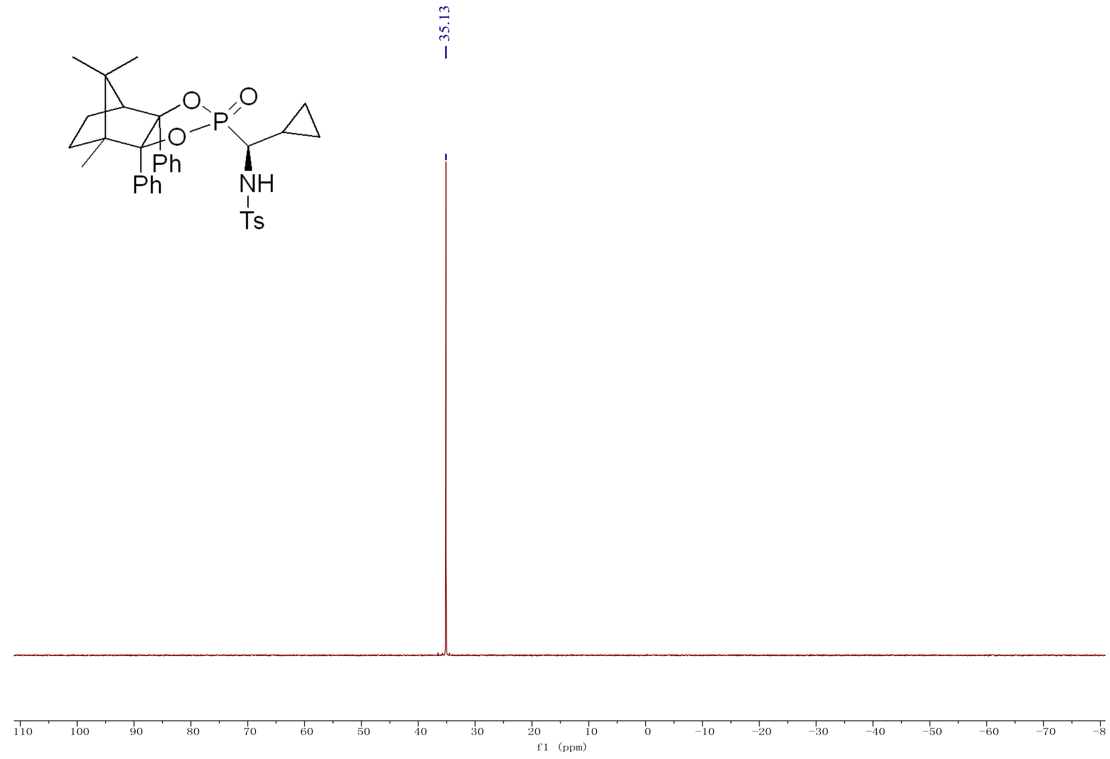

Fig. S207 ^{31}P NMR of compound **6yd**

^{1}H NMR (300 MHz, $CDCl_3$)

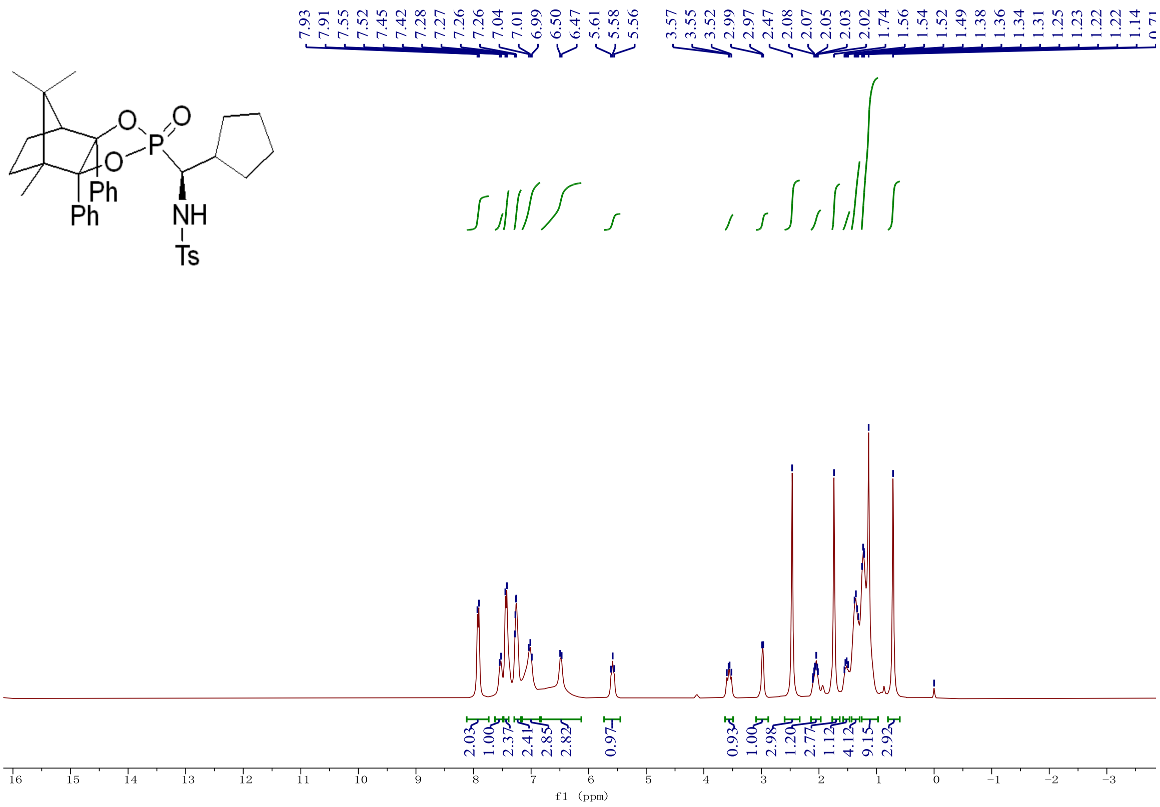

Fig. S208 ^{1}H NMR of compound **6ye**

^{13}C NMR (75 MHz, $CDCl_3$)

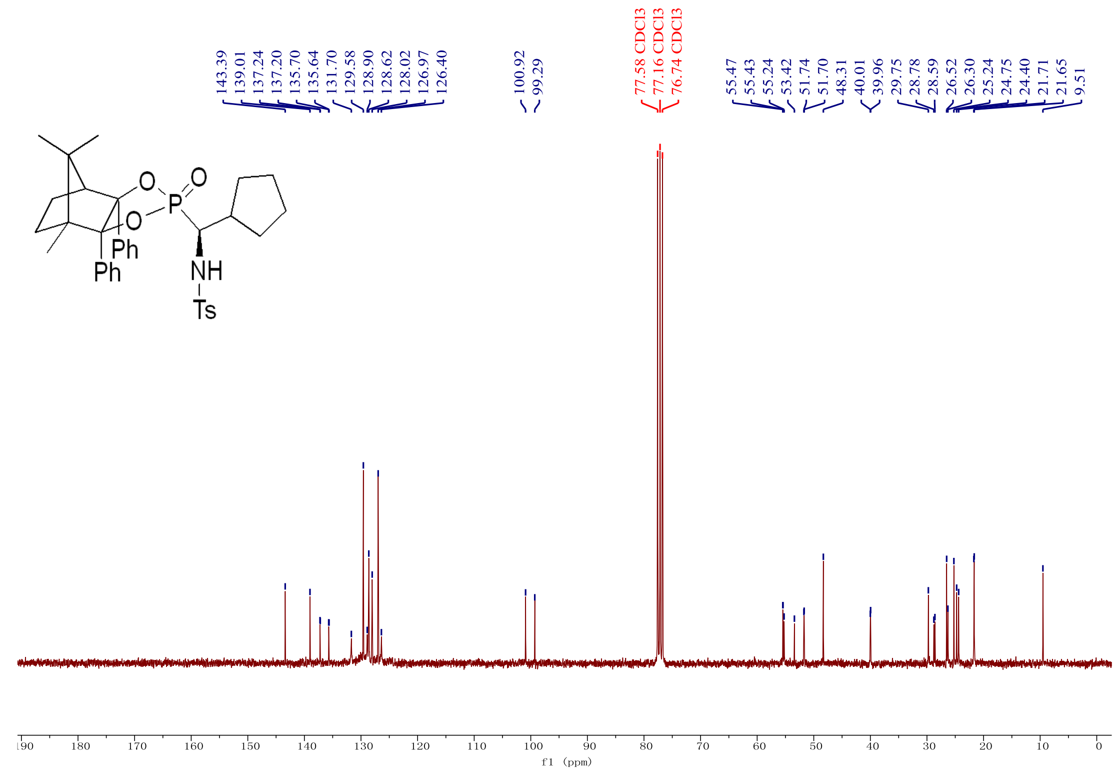

Fig. S209 ^{13}C NMR of compound **6ye**

^{31}P NMR (121 MHz, $CDCl_3$)

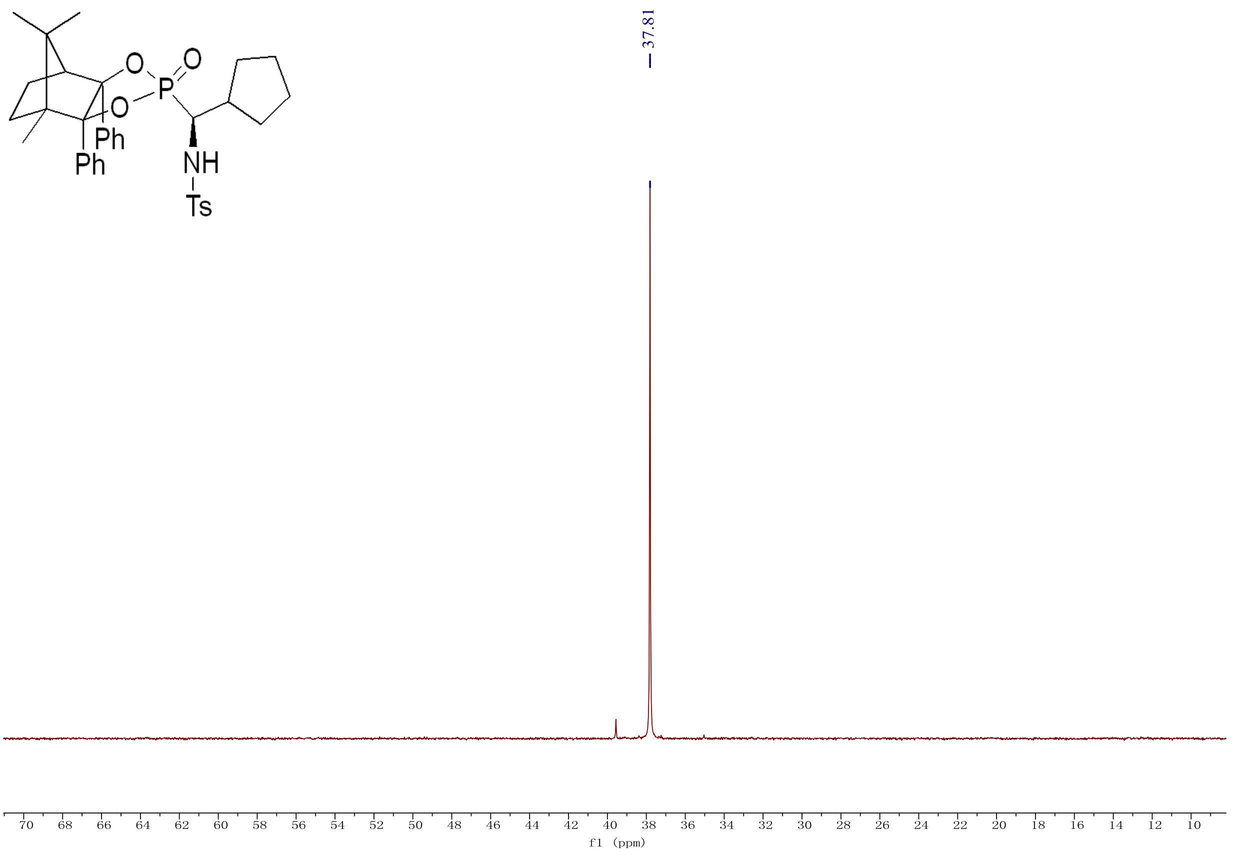

Fig. S210 ^{31}P NMR of compound **6ye**

^{1}H NMR (300 MHz, $CDCl_3$)

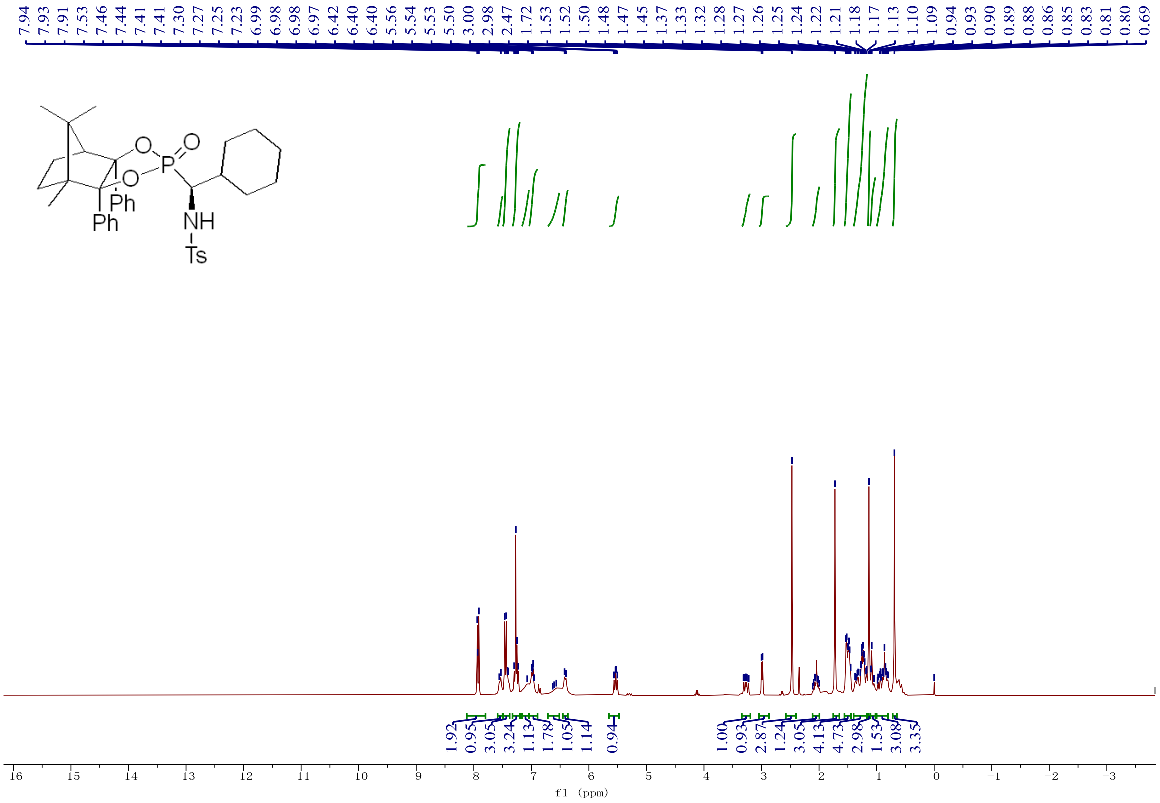

Fig. S211 ^{1}H NMR of compound 6**yf**

^{13}C NMR (75 MHz, $CDCl_3$)

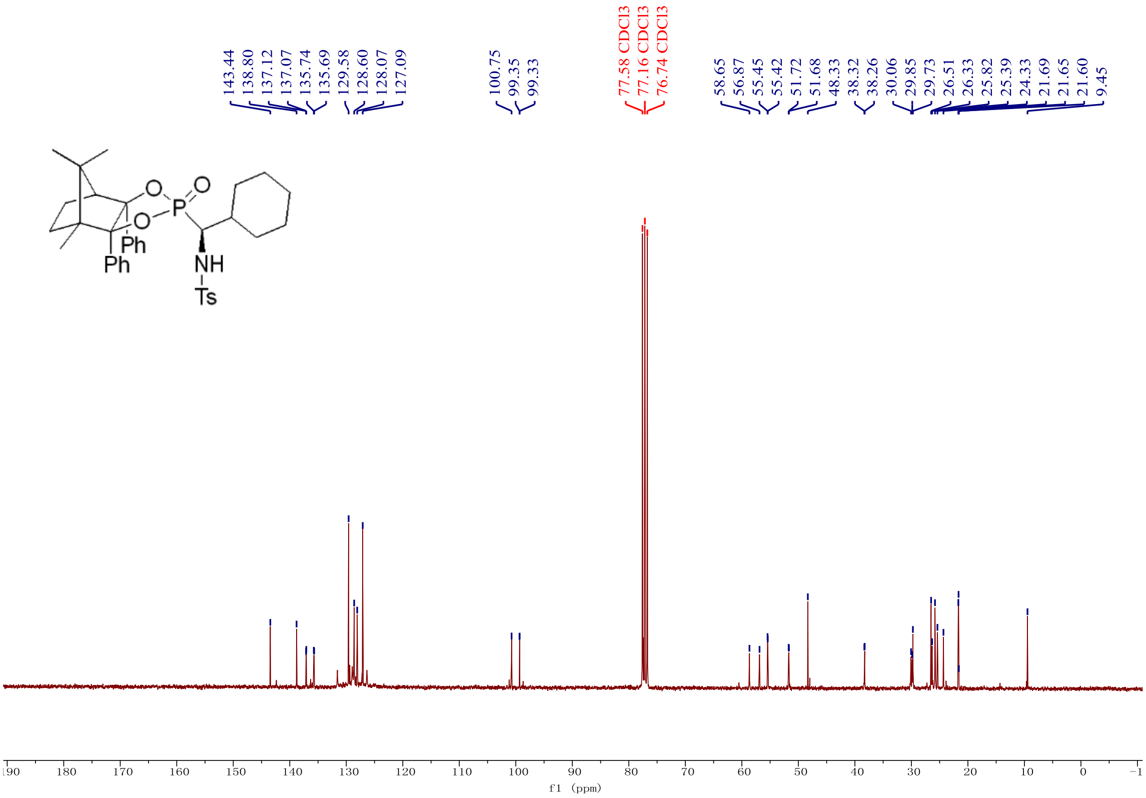

Fig. S212 ^{13}C NMR of compound **6yf**

^{31}P NMR (121 MHz, $CDCl_3$)

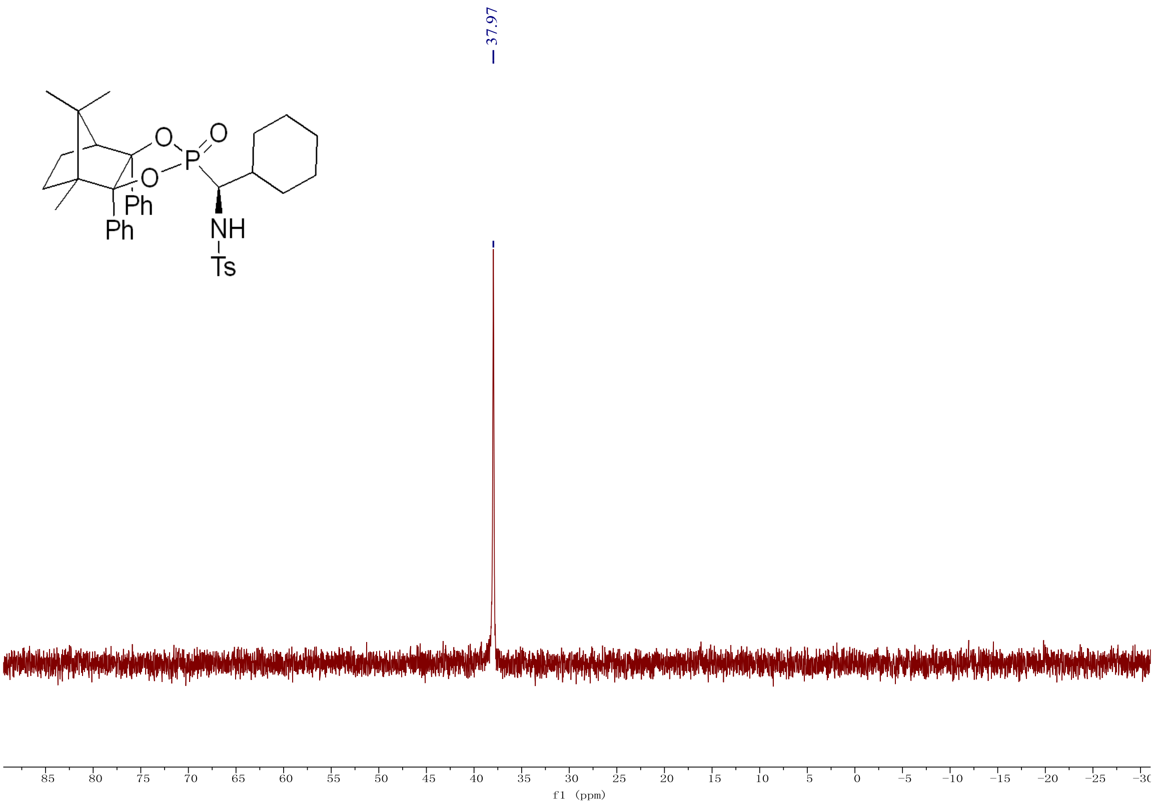

Fig. S213 ^{31}P NMR of compound **6yf**

^{1}H NMR (300 MHz, $CDCl_3$)

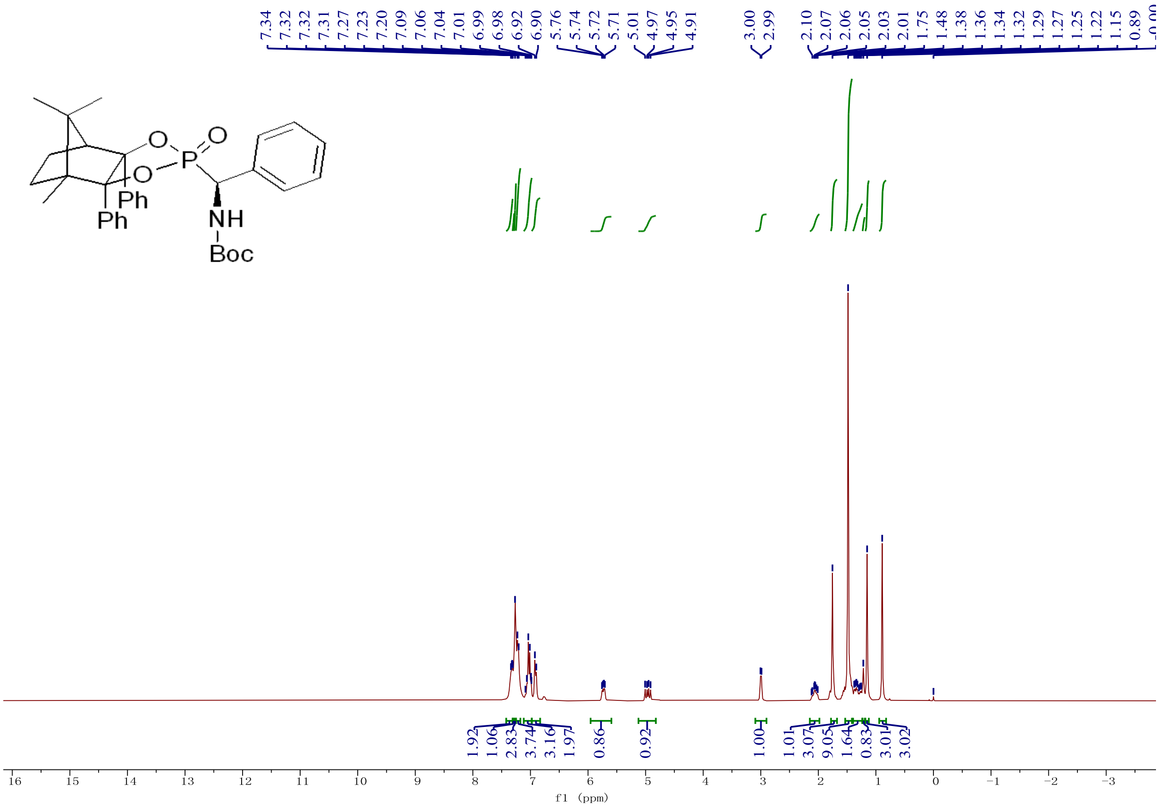

Fig. S214 ^{1}H NMR of compound **6za**

^{13}C NMR (75 MHz, $CDCl_3$)

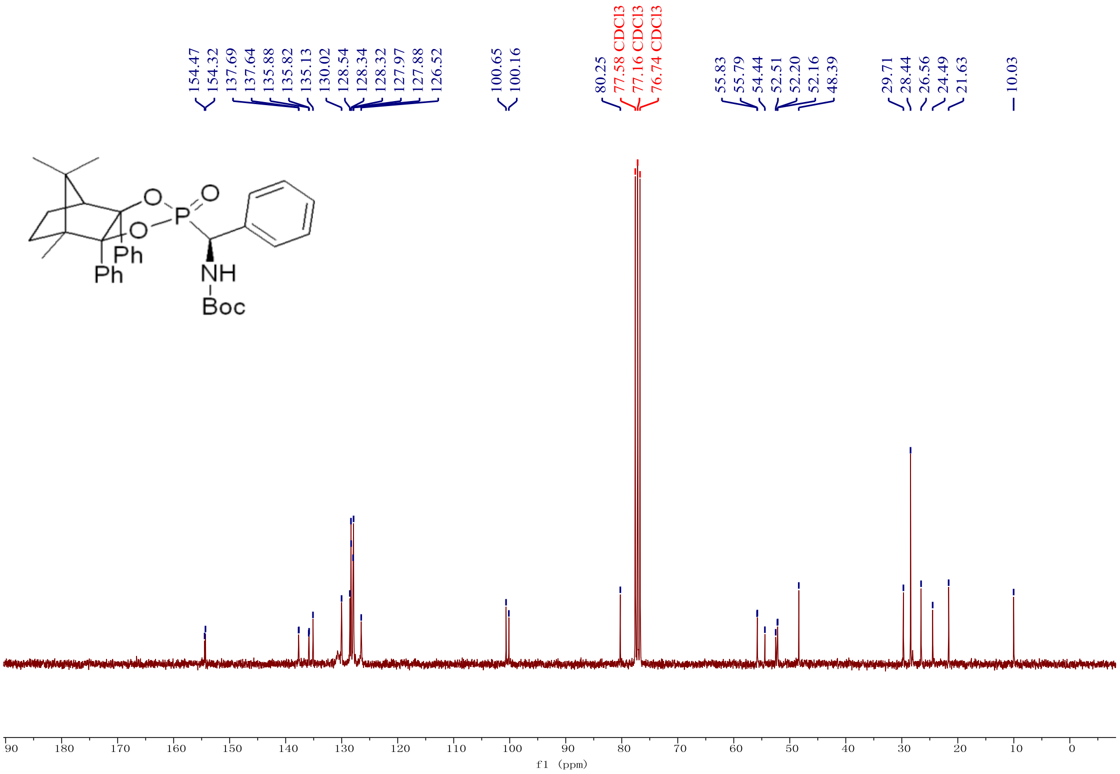

Fig. S215 ^{13}C NMR of compound **6za**

^{31}P NMR (121 MHz, $CDCl_3$)

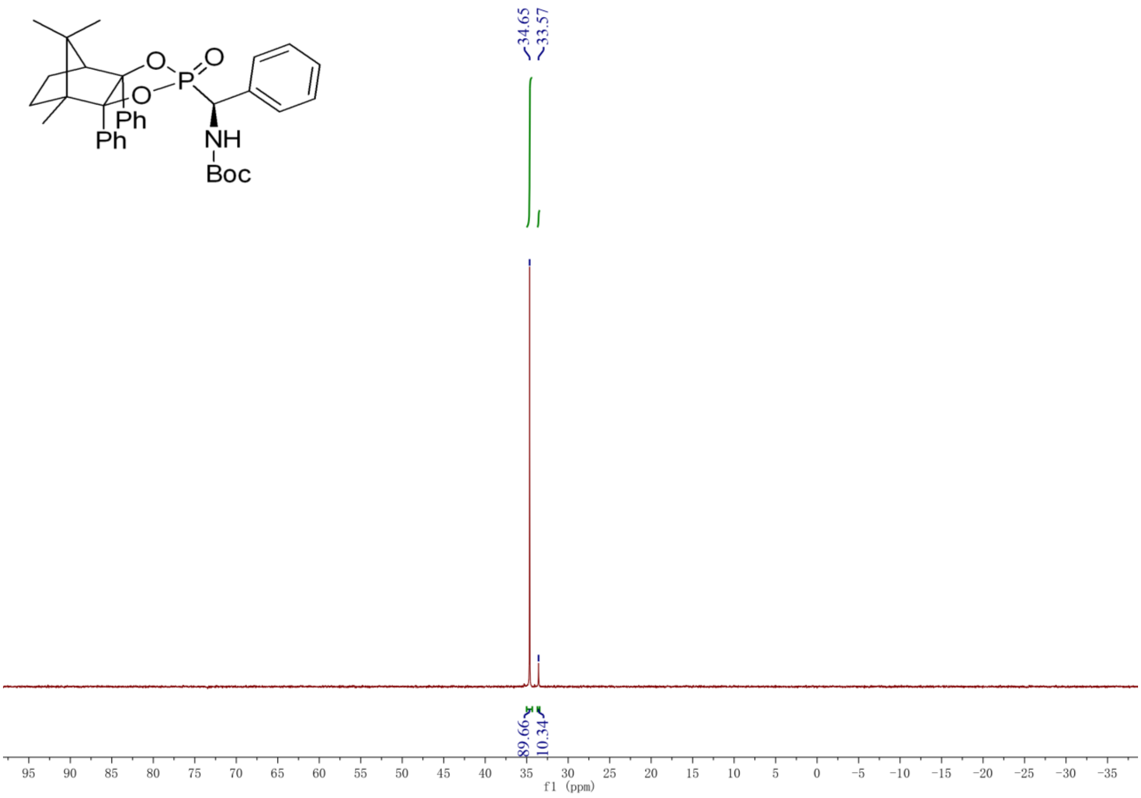

Fig. S216 ^{31}P NMR of compound **6za**

^{1}H NMR (300 MHz, $CDCl_3$)

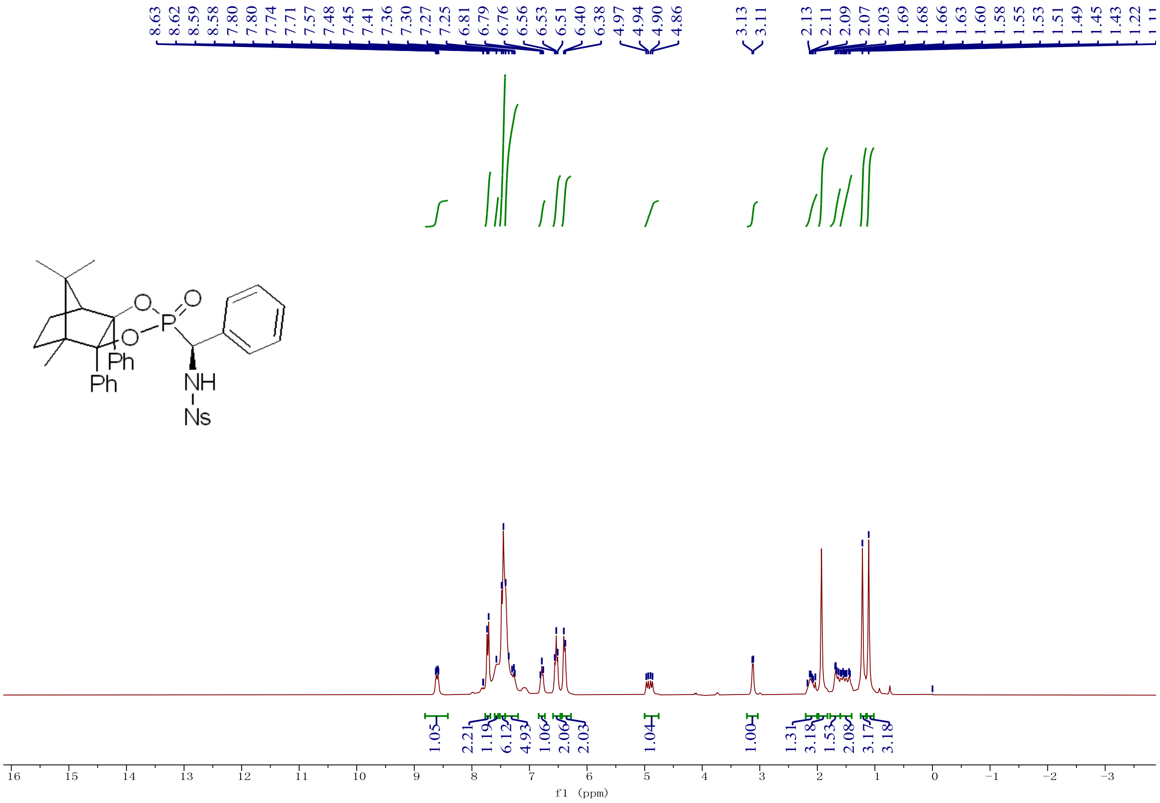

Fig. S217 ^{1}H NMR of compound **6zb**

^{13}C NMR (75 MHz, $CDCl_3$)

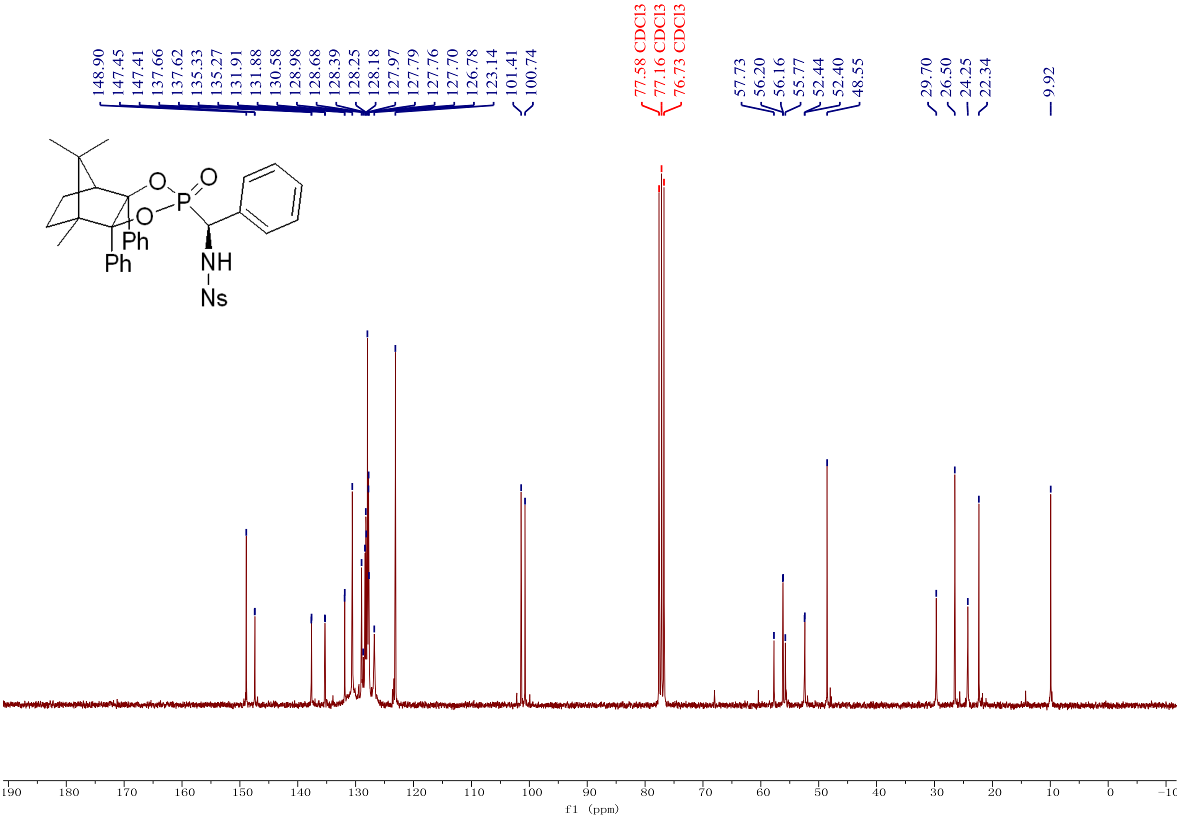

Fig. S218 ^{13}C NMR of compound **6zb**

^{31}P NMR (121 MHz, $CDCl_3$)

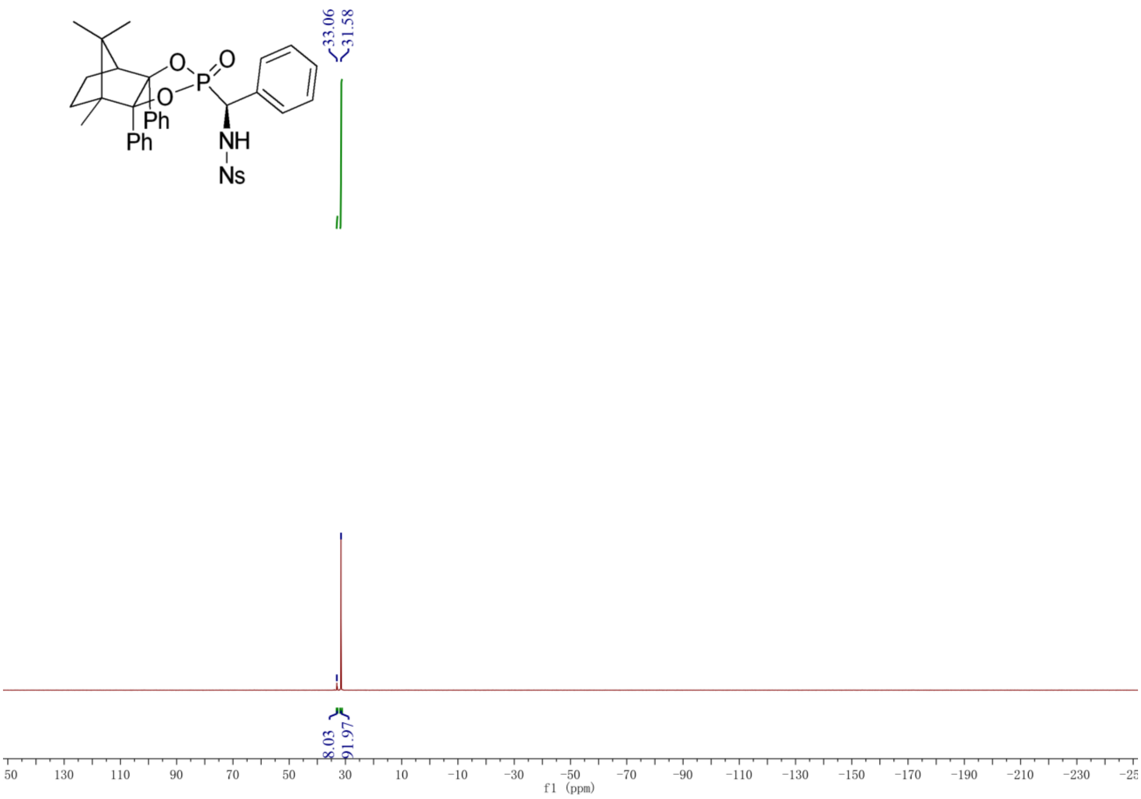

Fig. S219 ^{31}P NMR of compound **6zb**

^{1}H NMR (300 MHz, $CDCl_3$)

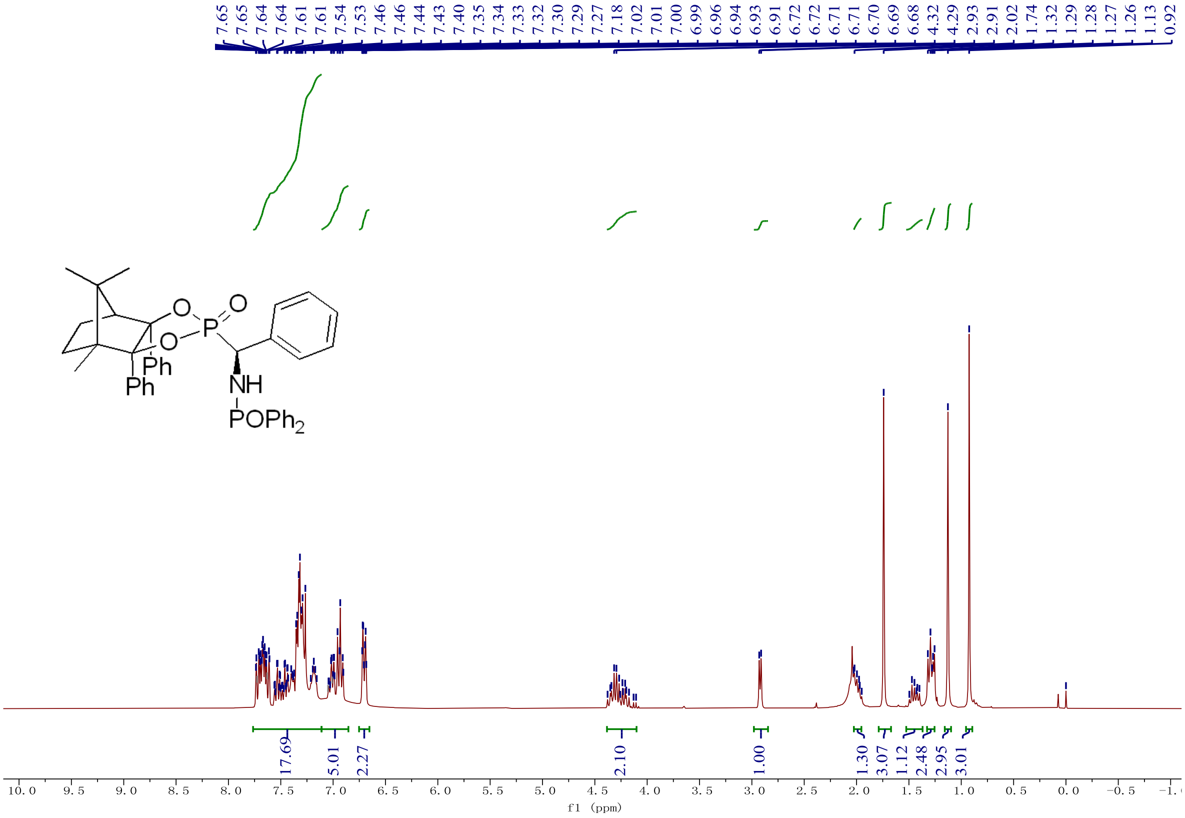

Fig. S220 ^{1}H NMR of compound **6zd**

^{13}C NMR (75 MHz, $CDCl_3$)

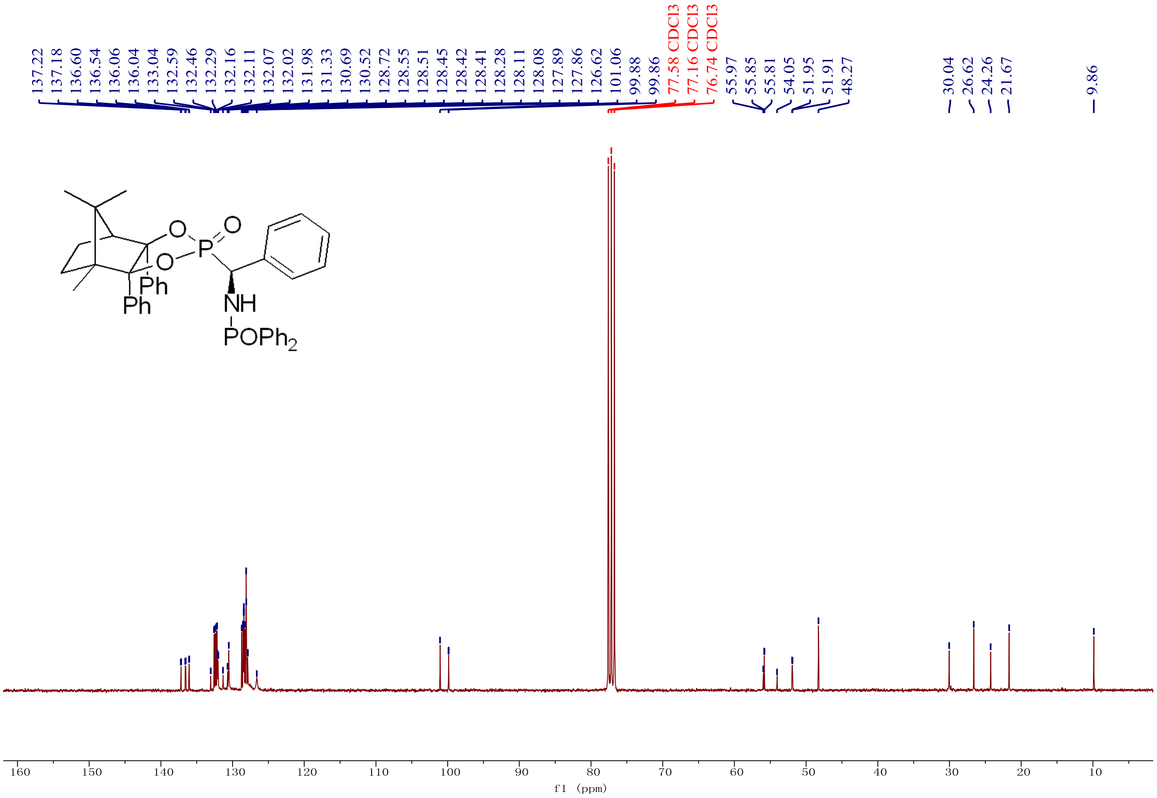

Fig. S221 ^{13}C NMR of compound **6zd**

^{31}P NMR (121 MHz, $CDCl_3$)

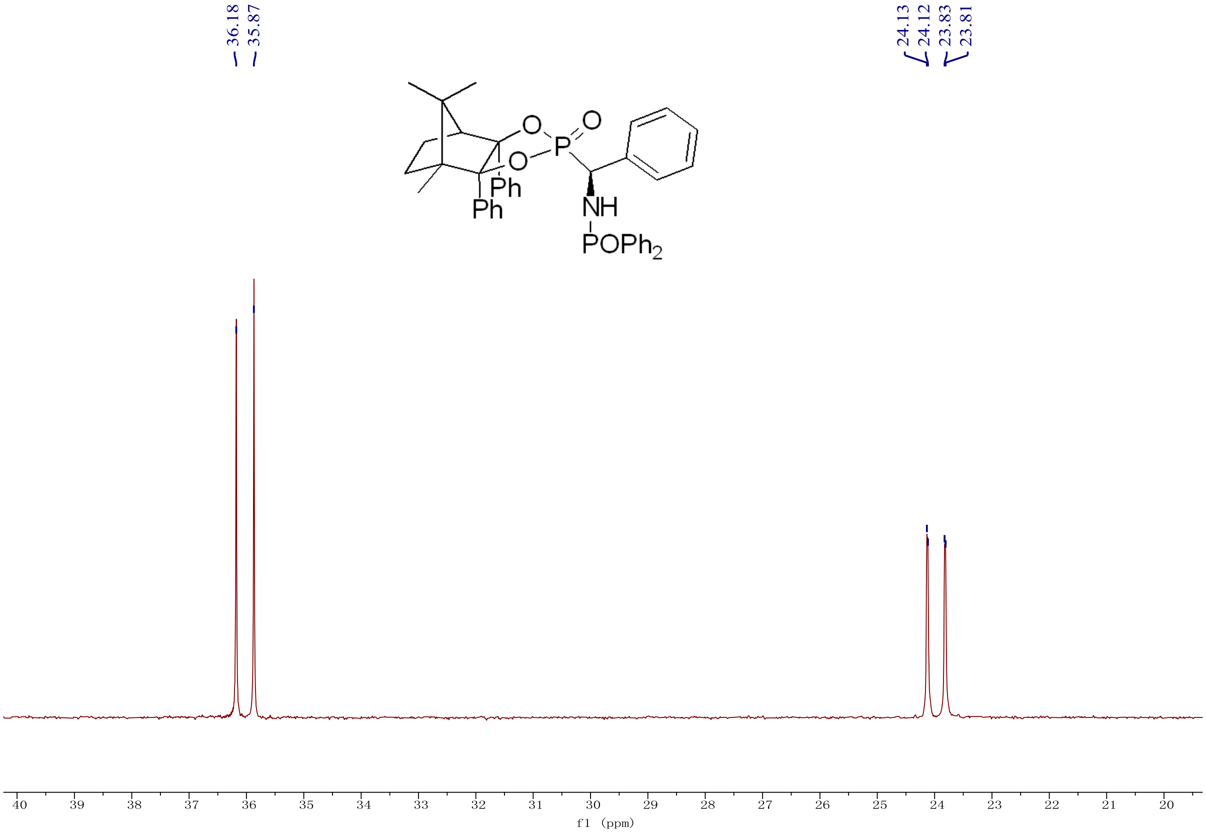

Fig. S222 ^{31}P NMR of compound **6zd**

^{1}H NMR (300 MHz, D_2O)

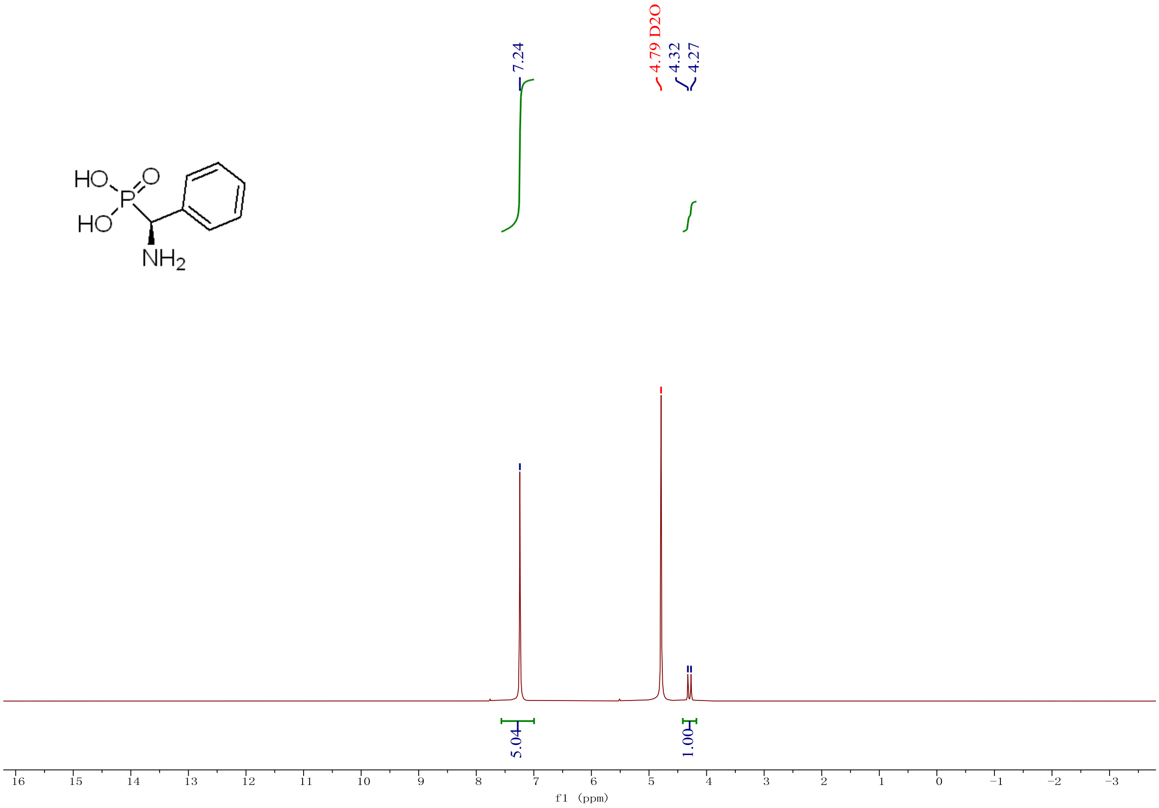

Fig. S223 ^{1}H NMR of compound **7a**

^{13}C NMR (75 MHz, D_2O)

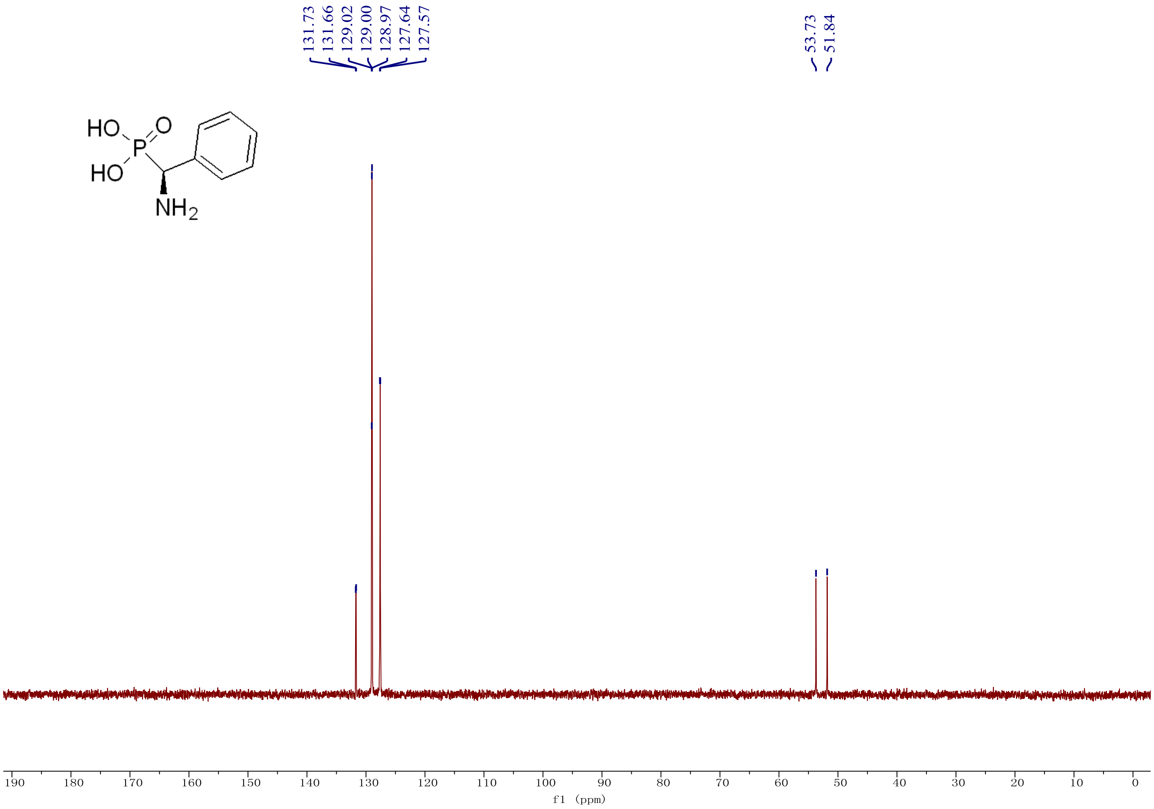

Fig. S224 ^{13}C NMR of compound **7a**

^{31}P NMR (121 MHz, D_2O)

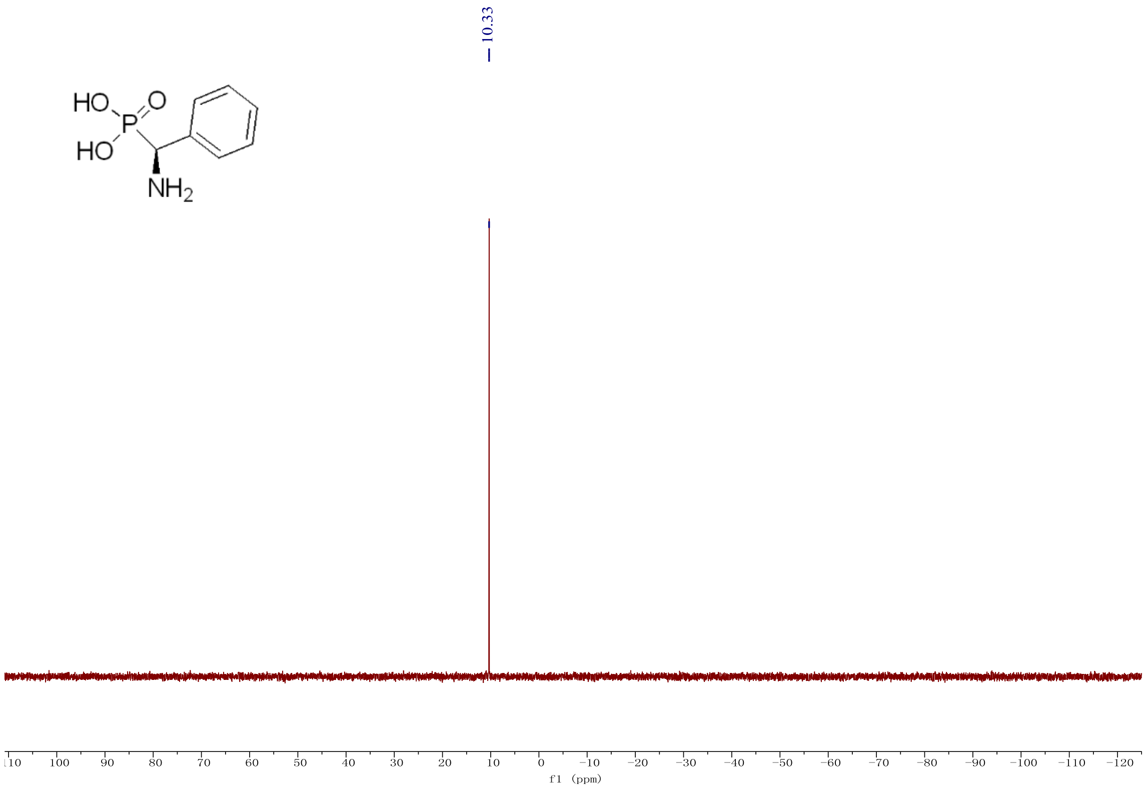

Fig. S225 ^{31}P NMR of compound **7a**

^{1}H NMR (300 MHz, D_2O)

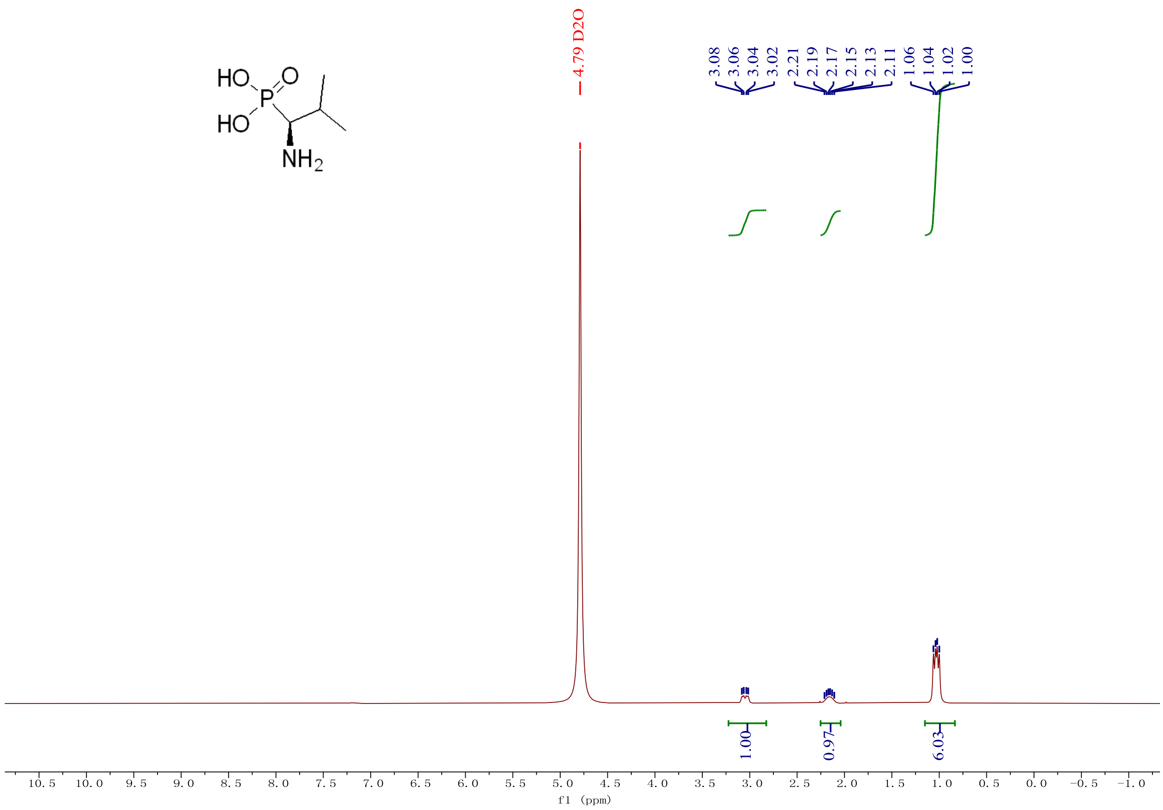

Fig. S226 ^{1}H NMR of compound **7b**

^{13}C NMR (75 MHz, D_2O)

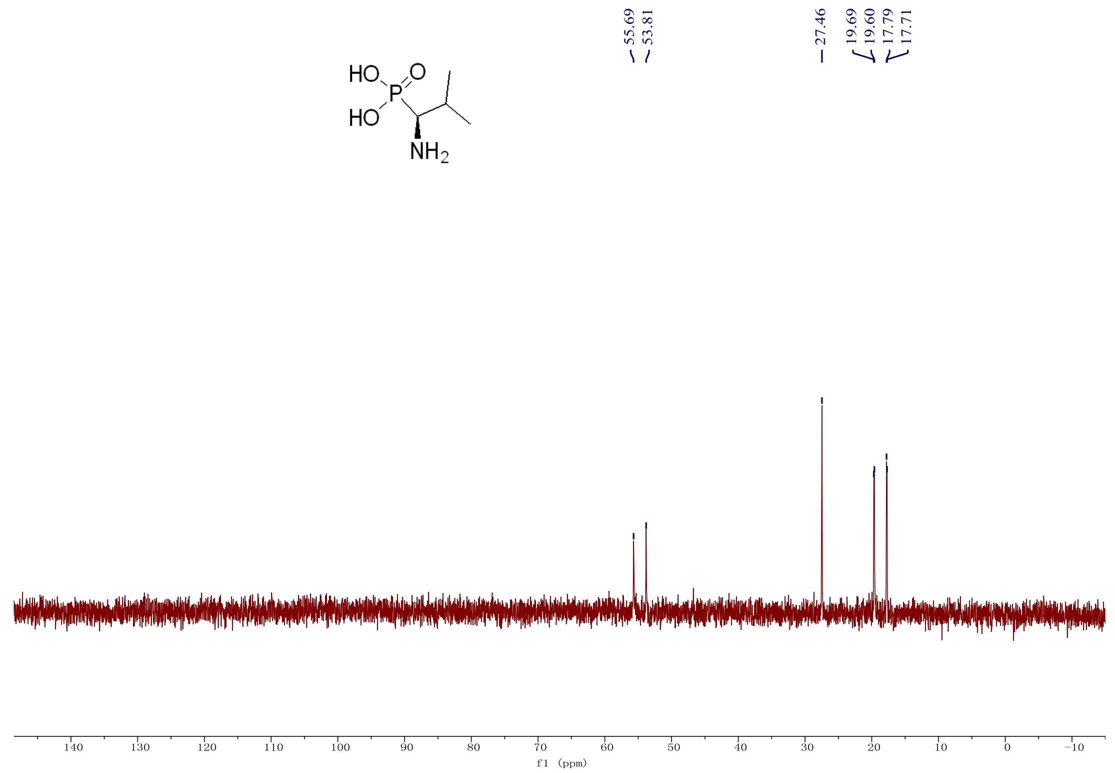

Fig. S227 ^{13}C NMR of compound **7b**

^{31}P NMR (121 MHz, D_2O)

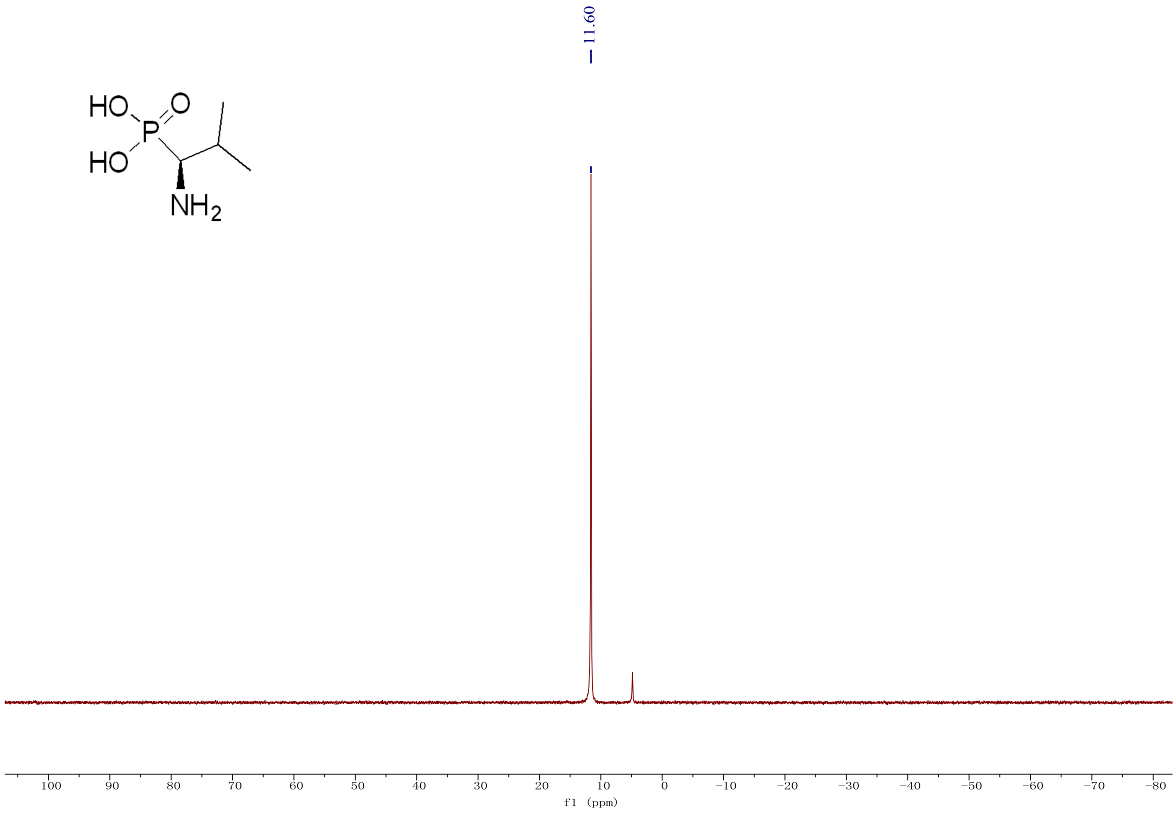

Fig. S228 ^{31}P NMR of compound **7b**

^{1}H NMR (300 MHz, $CDCl_3$)

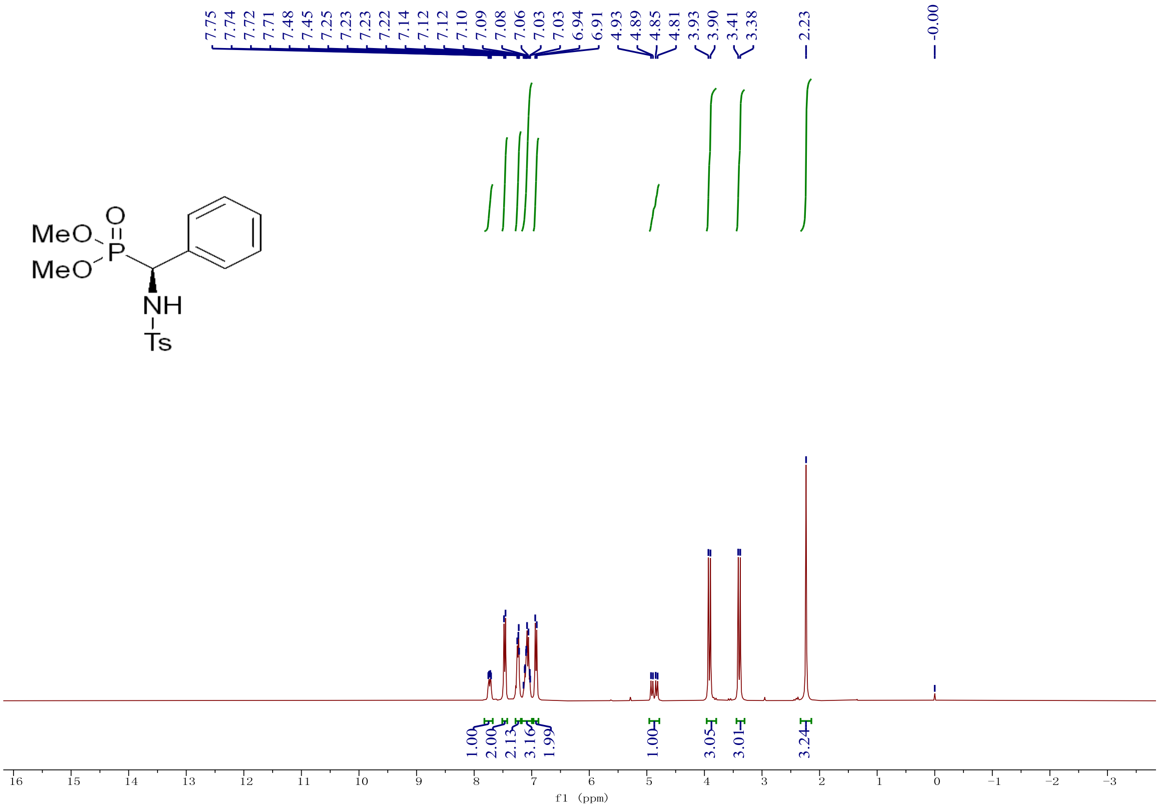

Fig. S229 ^{1}H NMR of compound **7aa**

^{13}C NMR (75 MHz, $CDCl_3$)

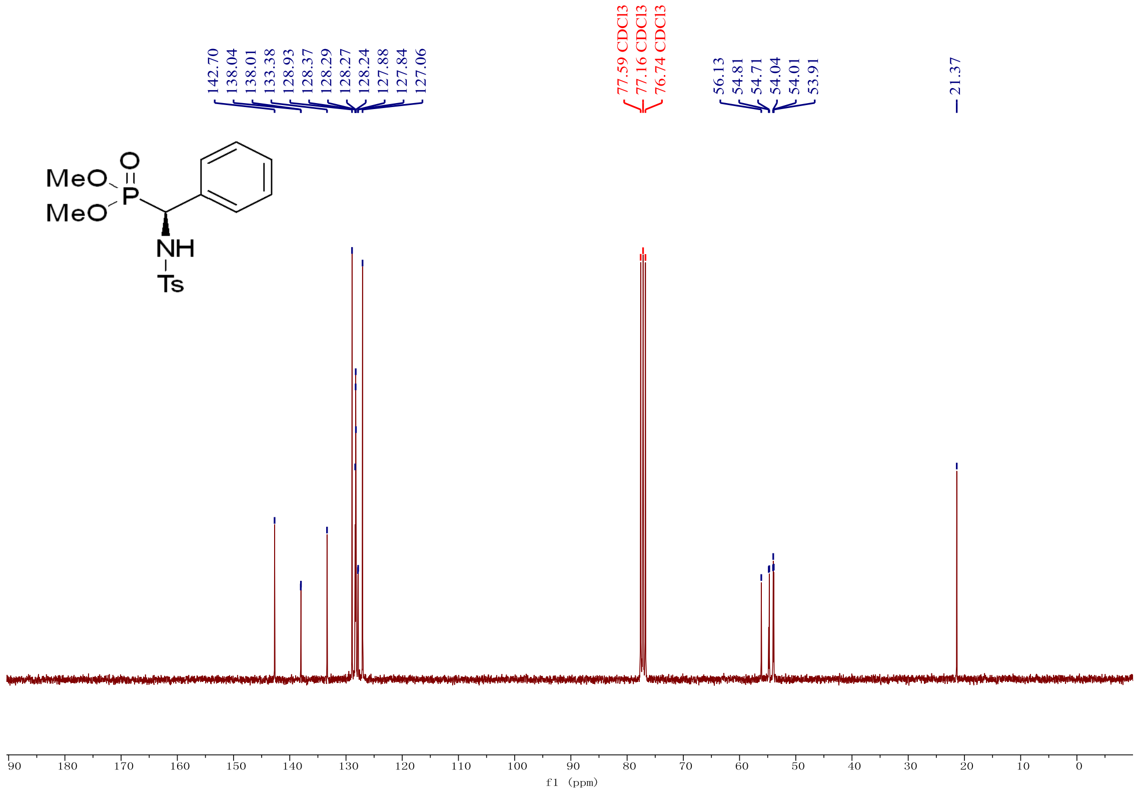

Fig. S230 13C NMR of compound 7aa

^{31}P NMR (121 MHz, $CDCl_3$)

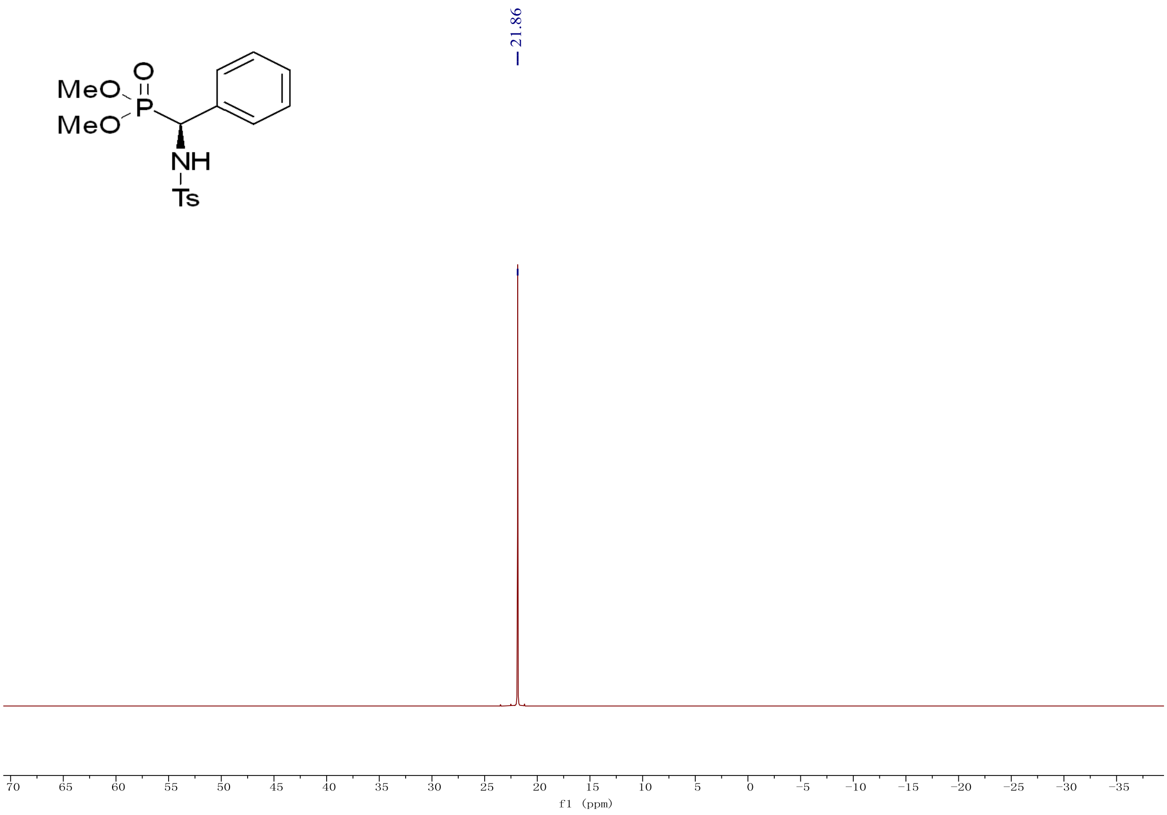

Fig. S231 ^{31}P NMR of compound **7aa**

^{1}H NMR (300 MHz, $CDCl_3$)

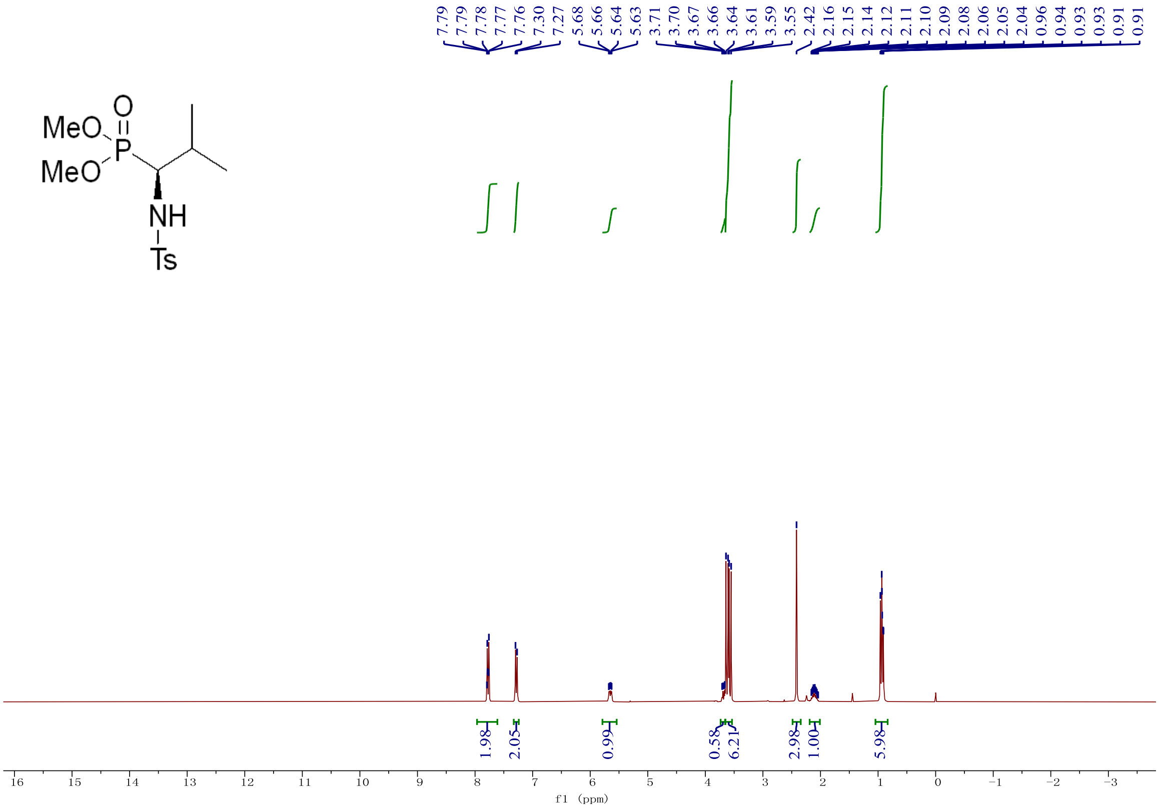

Fig. S232 ^{1}H NMR of compound **7ba**

^{13}C NMR (75 MHz, $CDCl_3$)

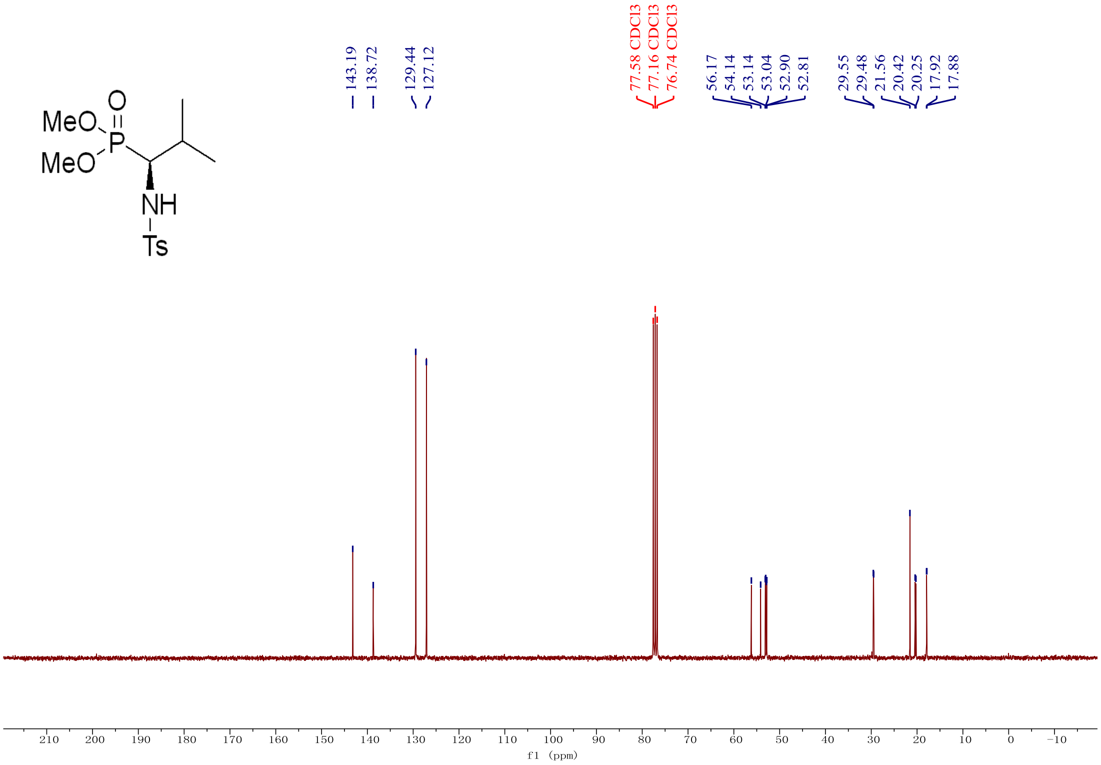

Fig. S233 ^{13}C NMR of compound **7ba**

^{31}P NMR (121 MHz, $CDCl_3$)

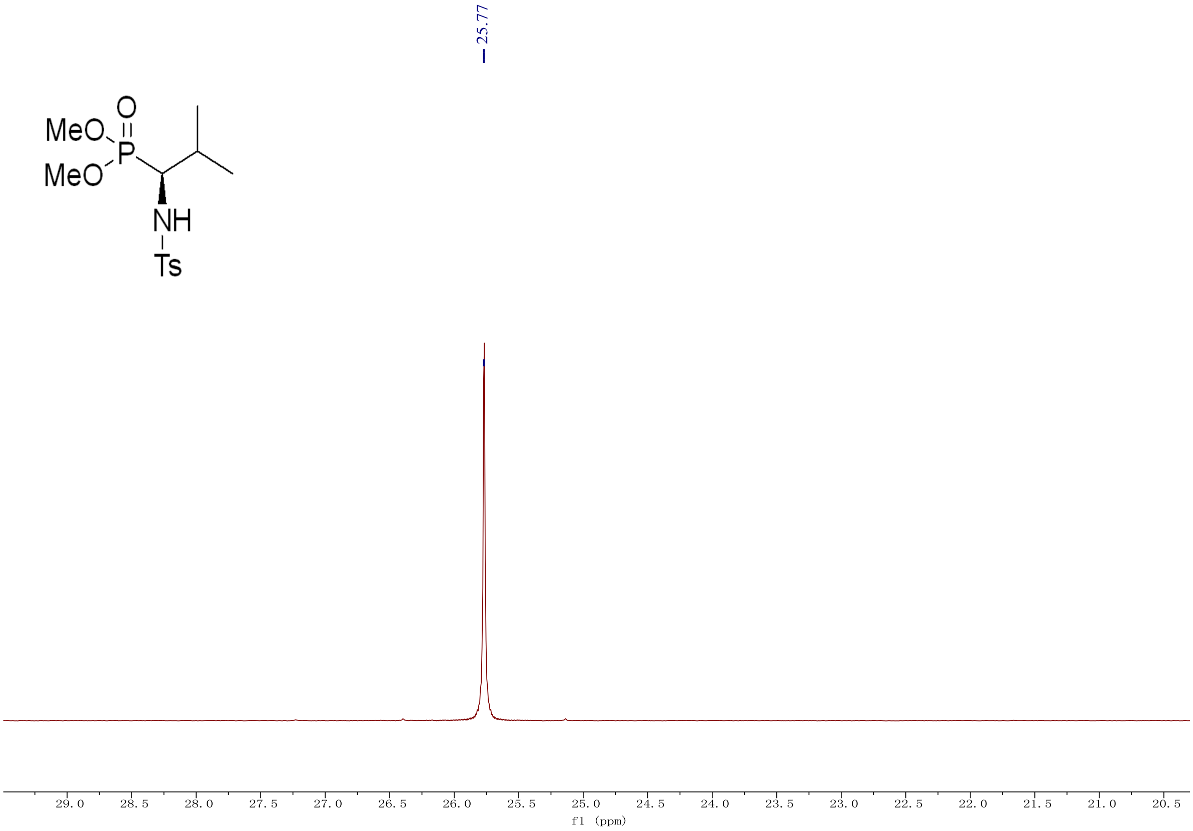

Fig. S234 ^{31}P NMR of compound **7ba**

^{1}H NMR (300 MHz, $CDCl_3$)

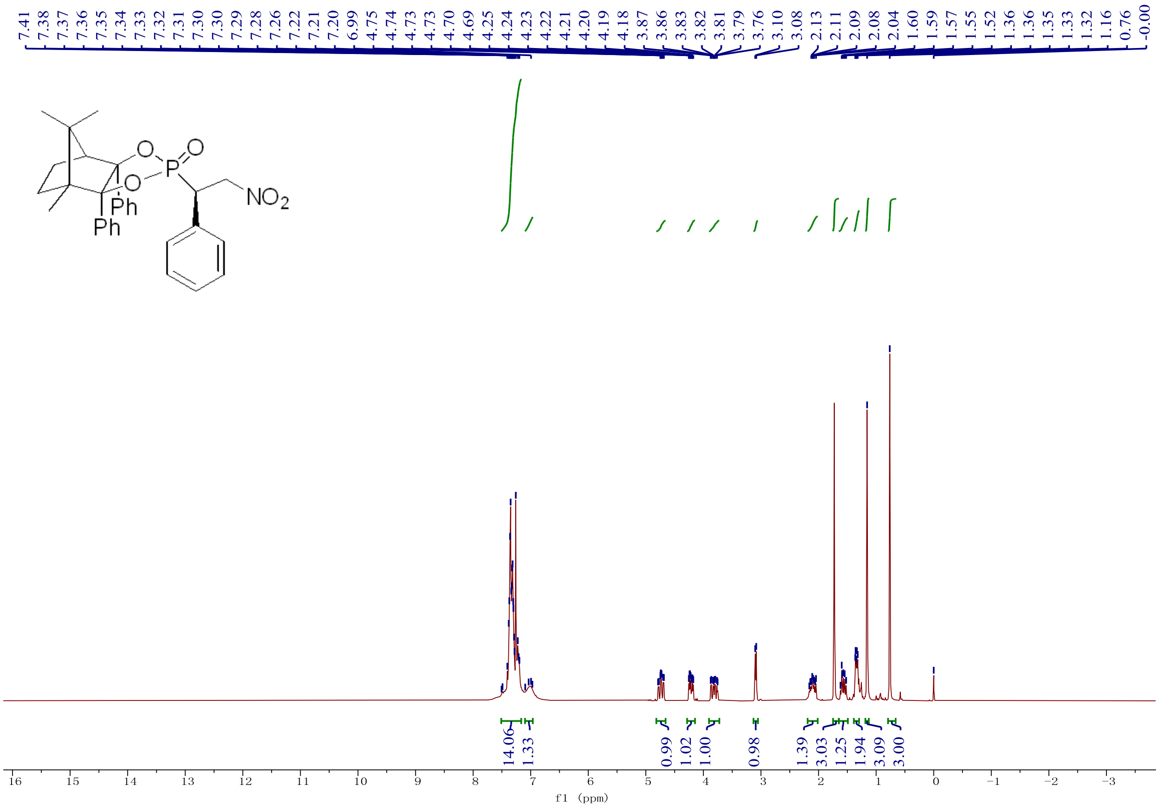

Fig. S235 ^{1}H NMR of compound **9a**

^{13}C NMR (75 MHz, $CDCl_3$)

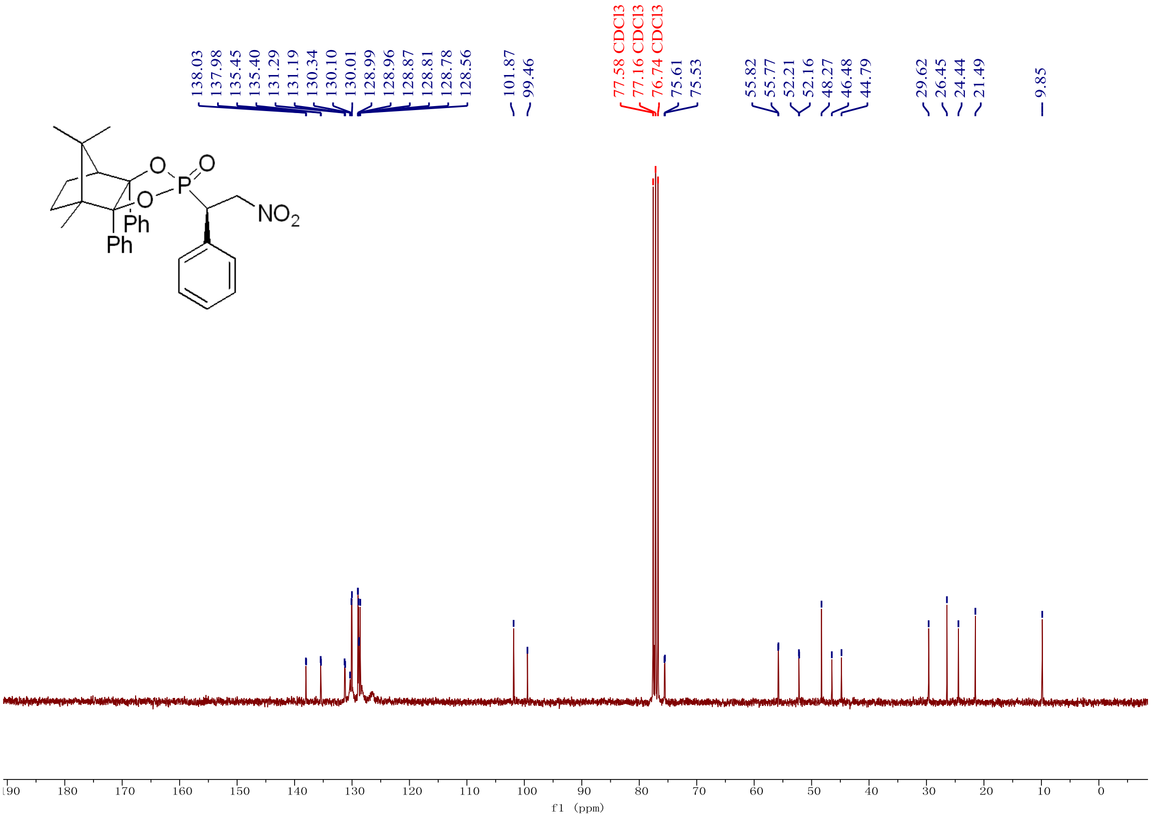

Fig. S236 ^{13}C NMR of compound **9a**

^{31}P NMR (121 MHz, $CDCl_3$)

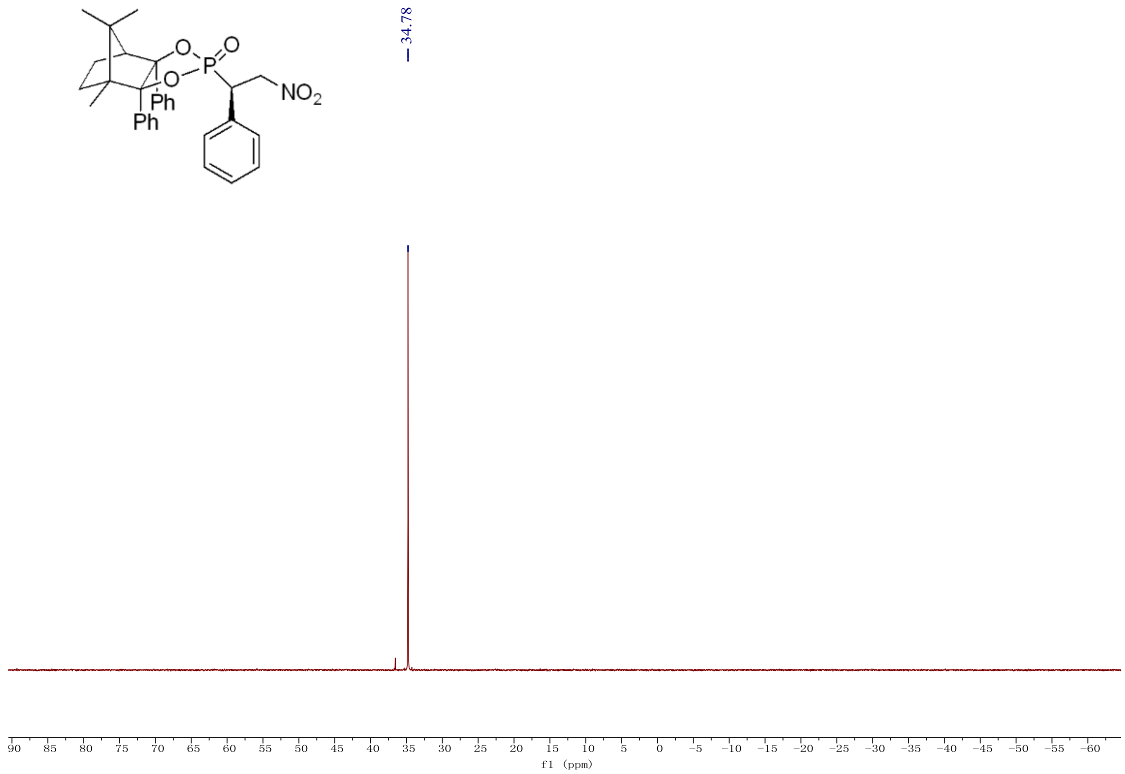

Fig. S237 ^{31}P NMR of compound **9a**

^{1}H NMR (300 MHz, $CDCl_3$)

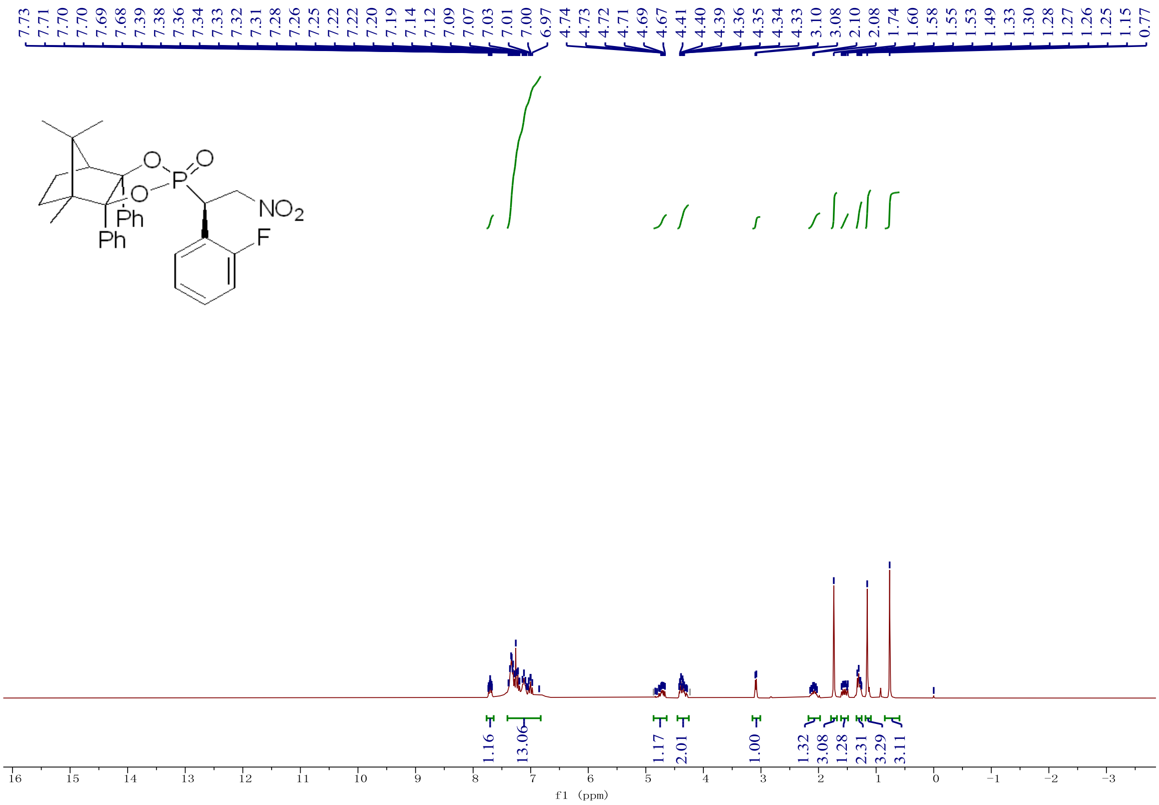

Fig. S238 ^{1}H NMR of compound **9b**

^{13}C NMR (75 MHz, $CDCl_3$)

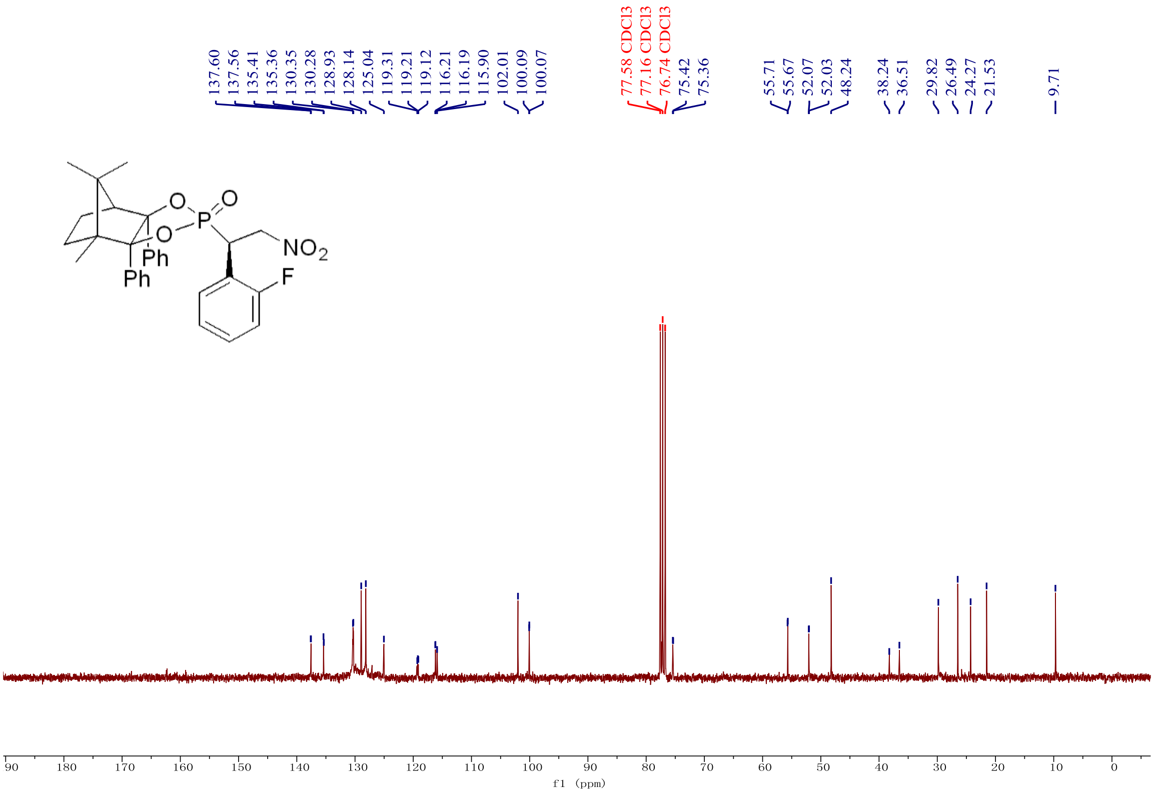

Fig. S239 ^{13}C NMR of compound **9b**

^{19}F NMR (282 MHz, $CDCl_3$)

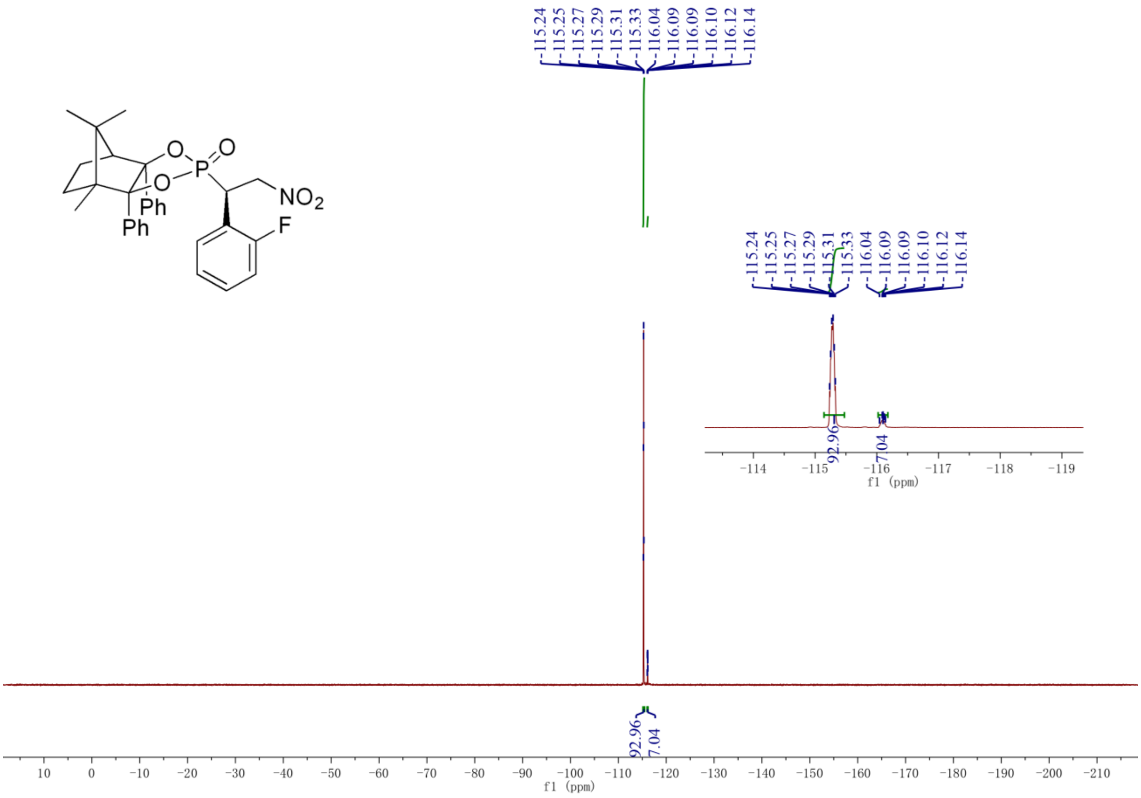

Fig. S240 ^{19}F NMR of compound **9b**

^{31}P NMR (121 MHz, $CDCl_3$)

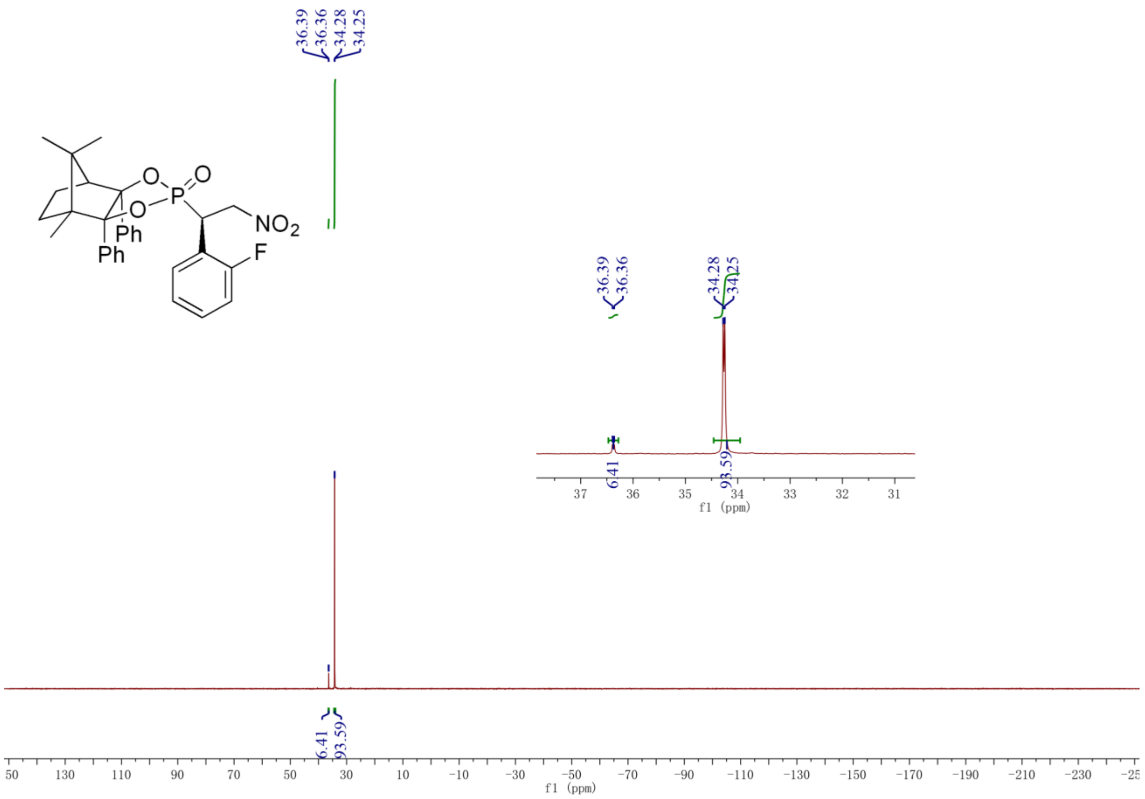

Fig. S241 ^{31}P NMR of compound **9b**

^{1}H NMR (300 MHz, $CDCl_3$)

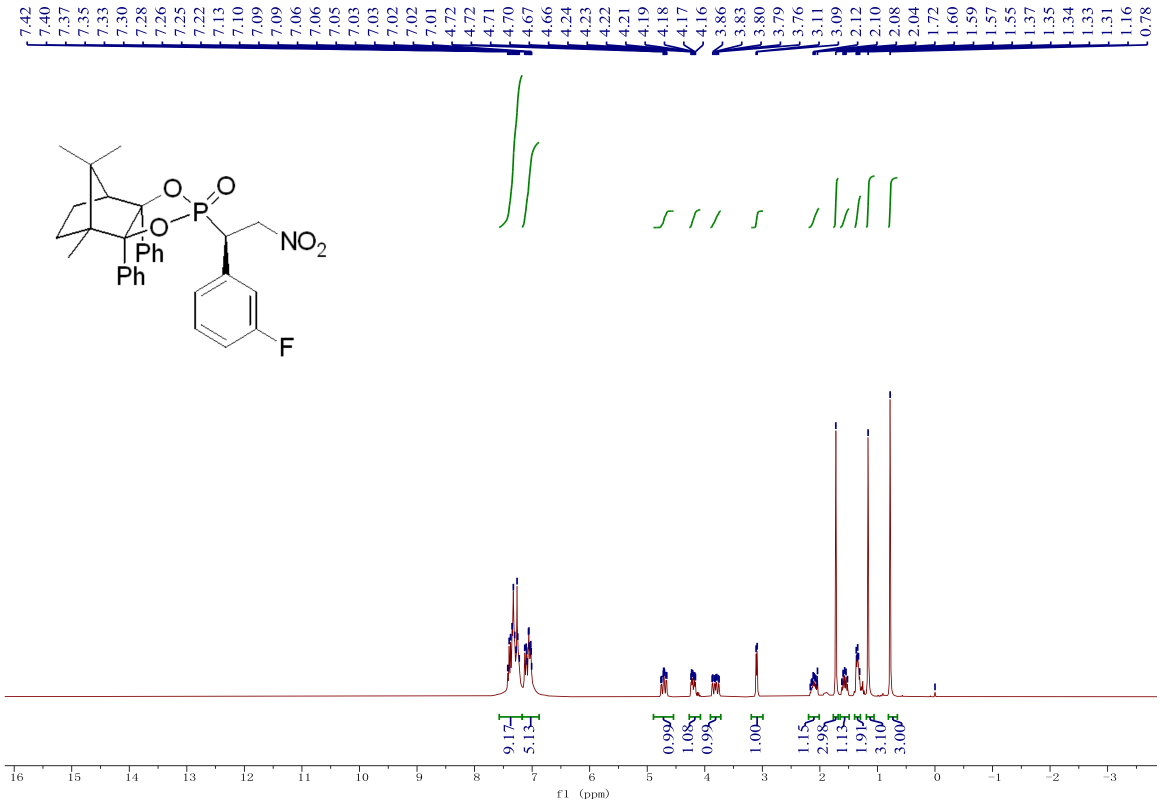

Fig. S242 ^{1}H NMR of compound **9c**

^{13}C NMR (75 MHz, $CDCl_3$)

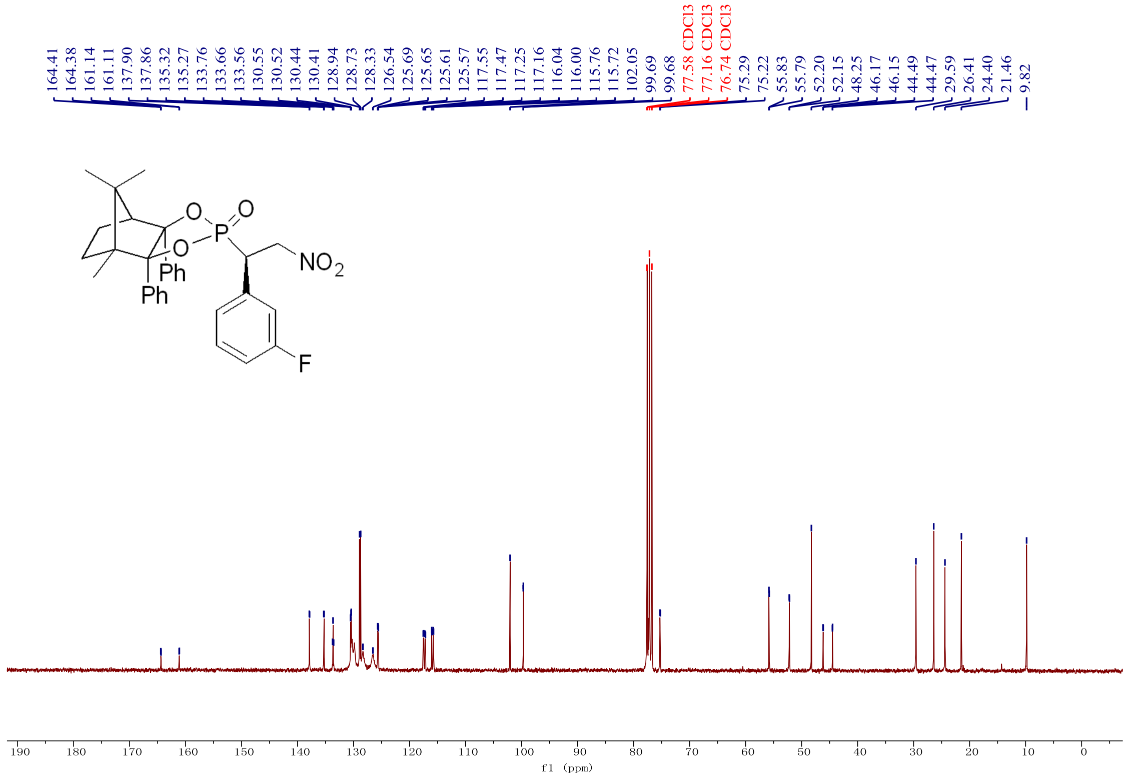

Fig. S243 ^{13}C NMR of compound **9c**

^{19}F NMR (282 MHz, $CDCl_3$)

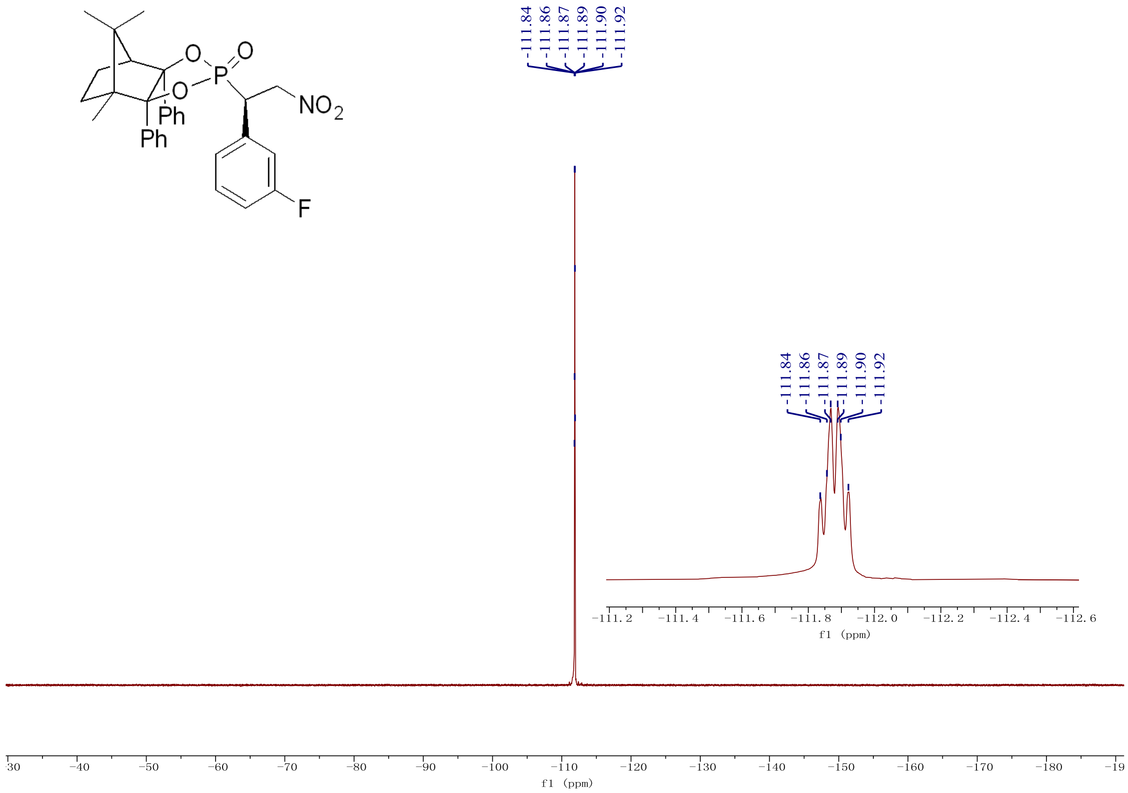

Fig. S244 ^{19}F NMR of compound **9c**

^{31}P NMR (121 MHz, $CDCl_3$)

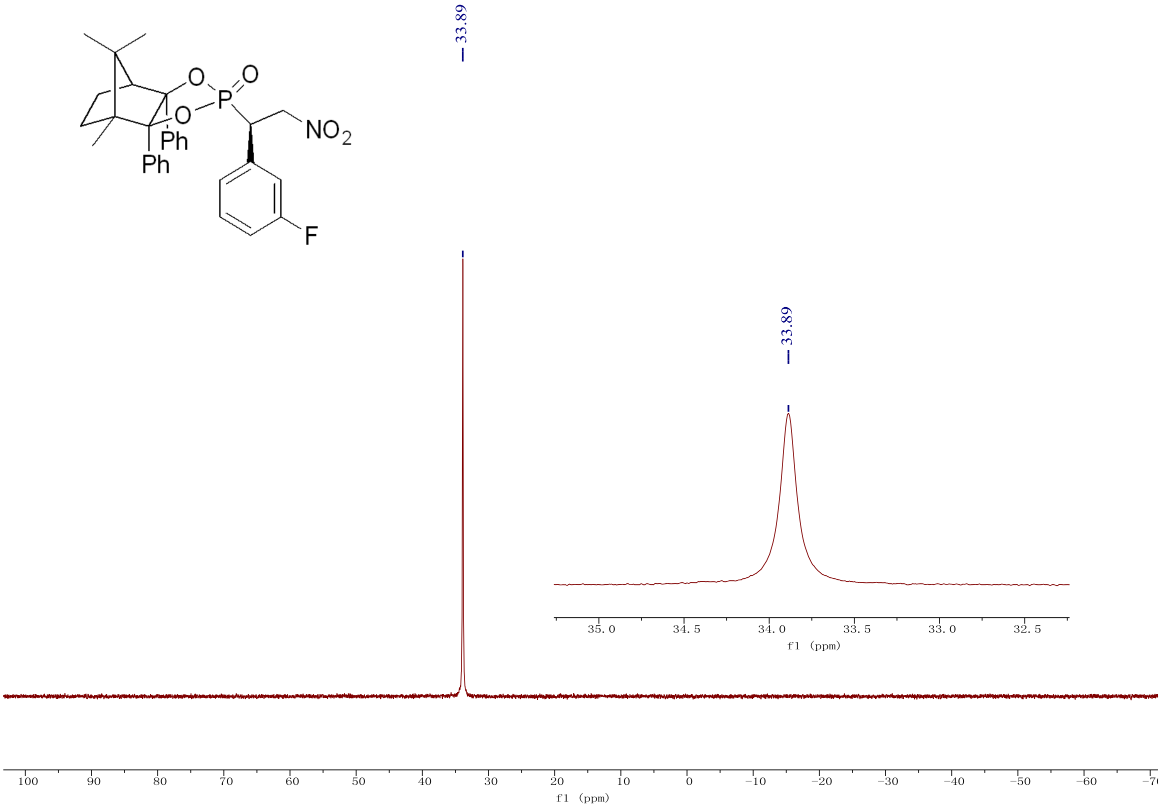

Fig. S245 ^{31}P NMR of compound **9c**

^{1}H NMR (300 MHz, $CDCl_3$)

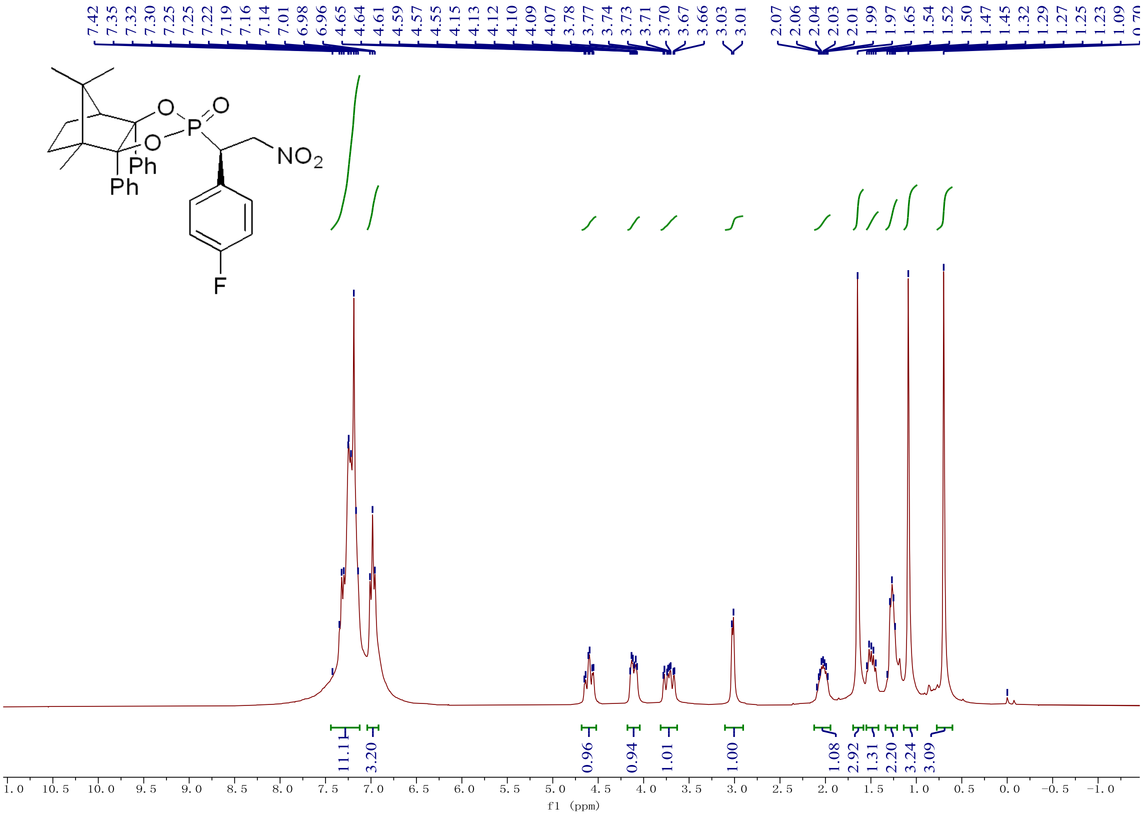

Fig. S246 ^{1}H NMR of compound **9d**

^{13}C NMR (75 MHz, $CDCl_3$)

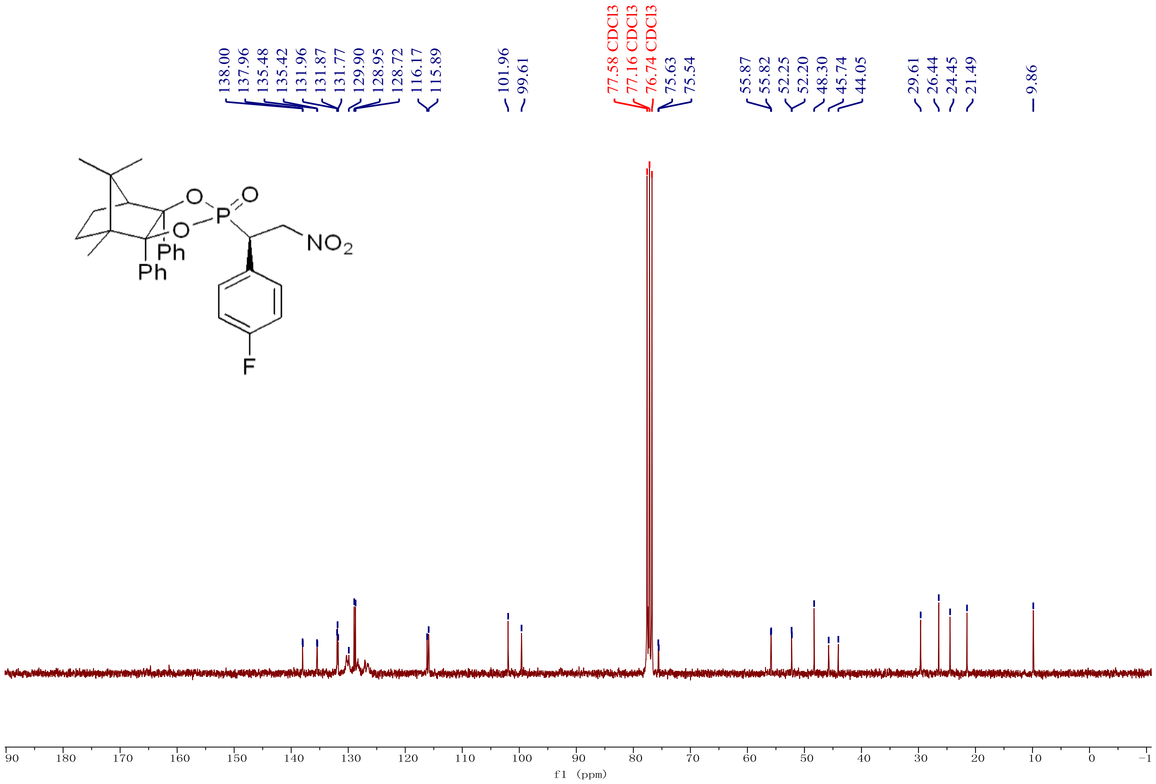

Fig. S247 ^{13}C NMR of compound **9d**

^{19}F NMR (282 MHz, $CDCl_3$)

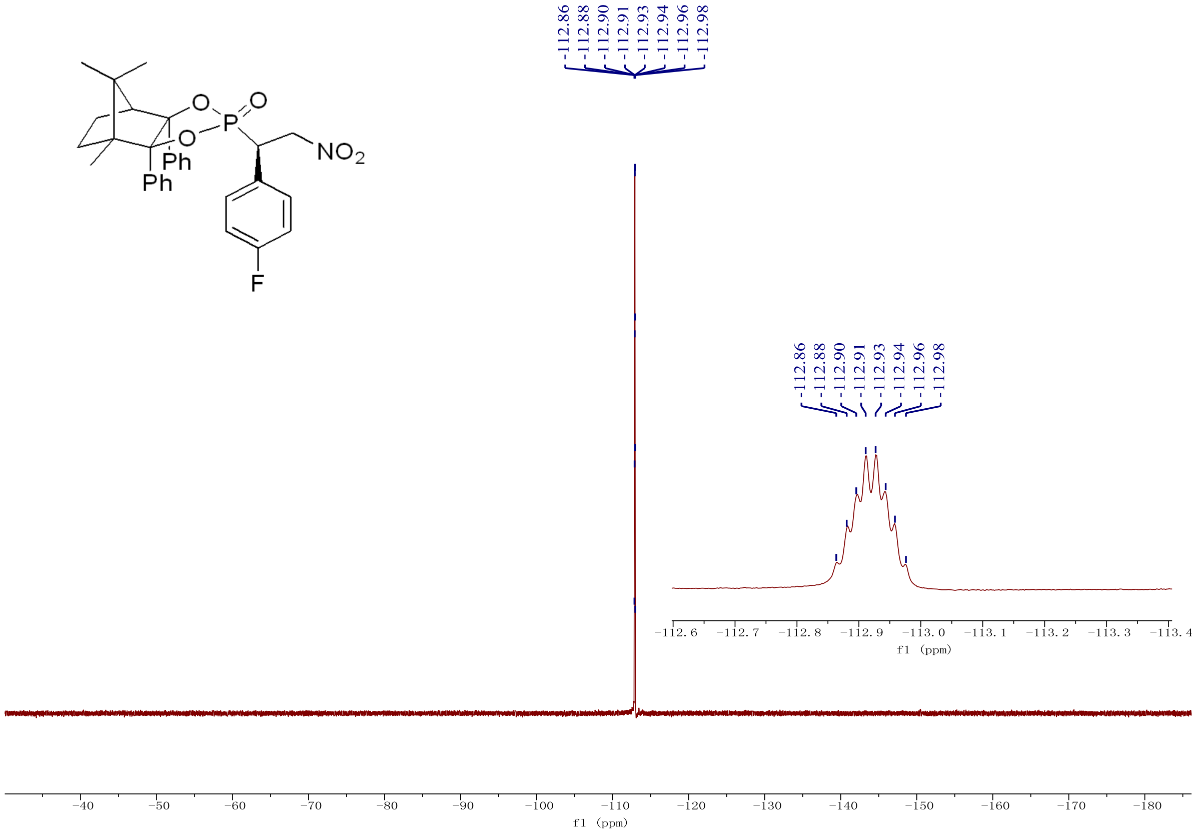

Fig. S248 ^{19}F NMR of compound **9d**

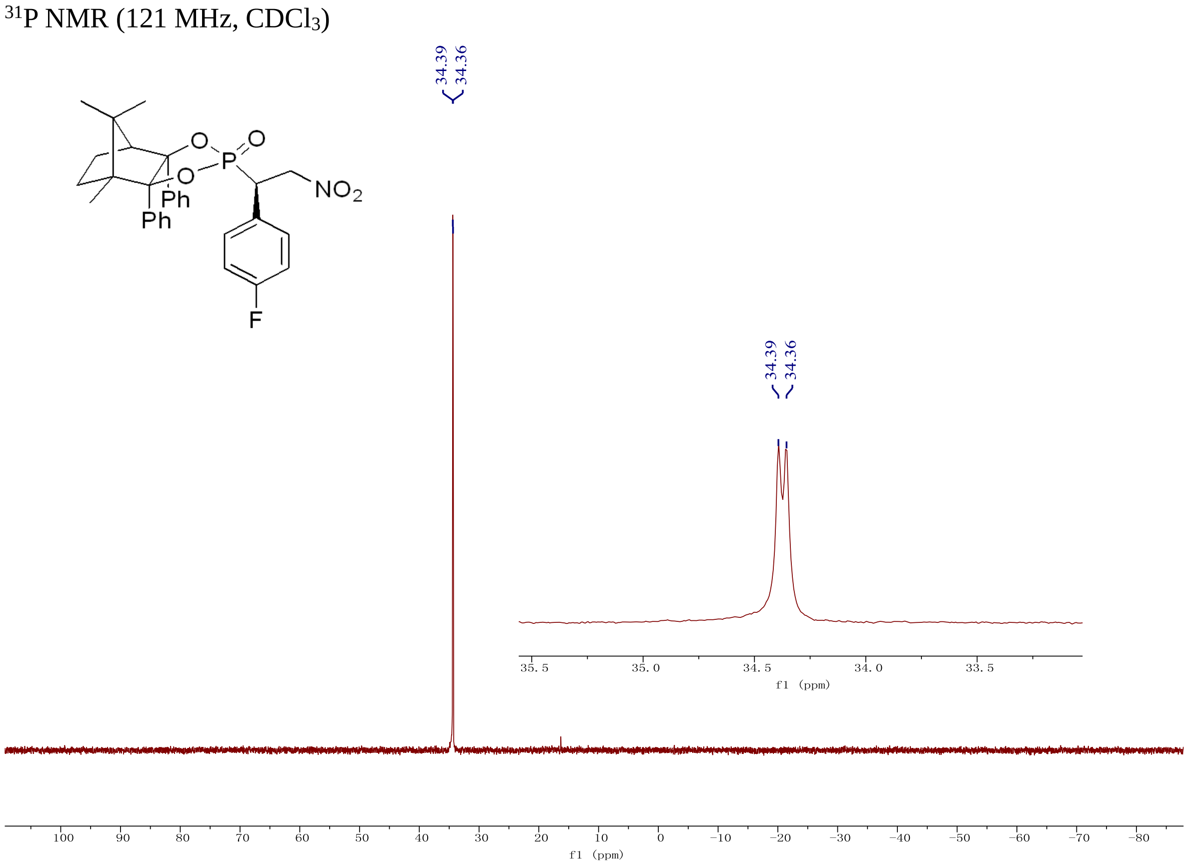

Fig. S249 ^{31}P NMR of compound **9d**

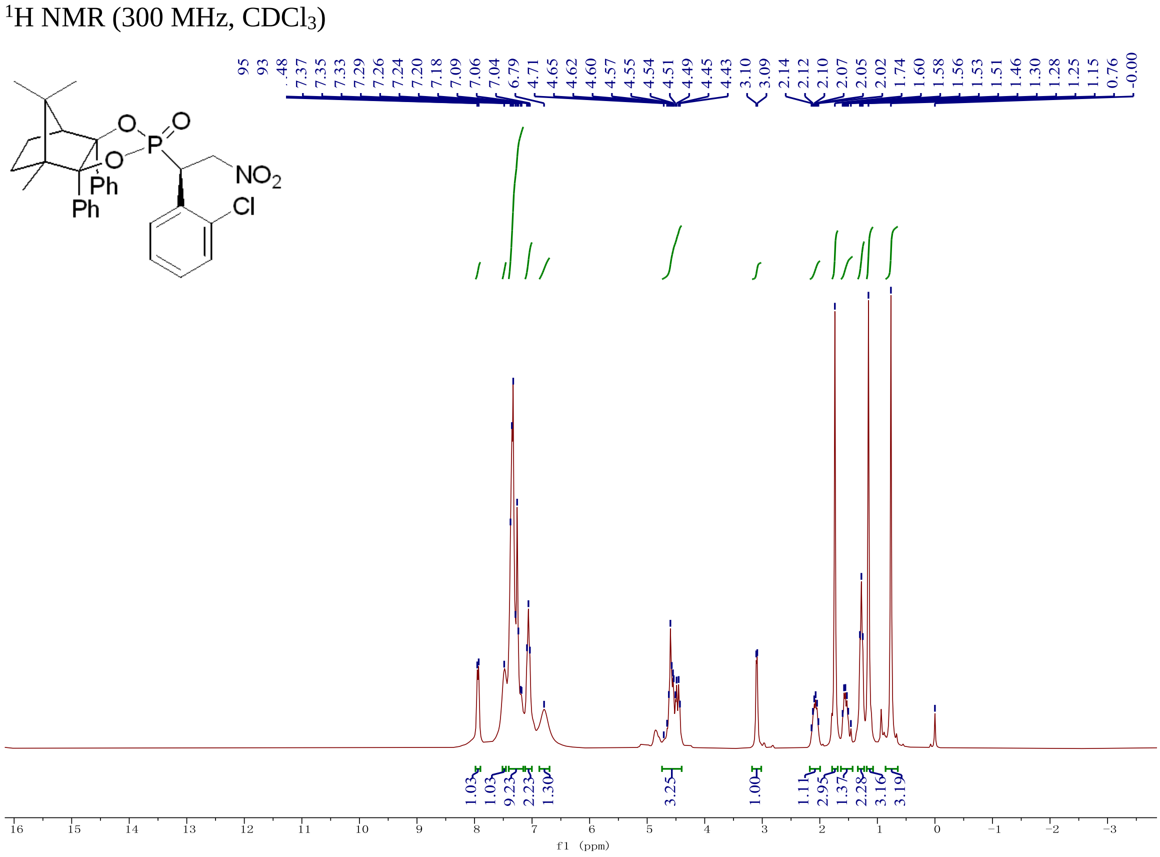

Fig. S250 ^{1}H NMR of compound **9e**

^{13}C NMR (75 MHz, $CDCl_3$)

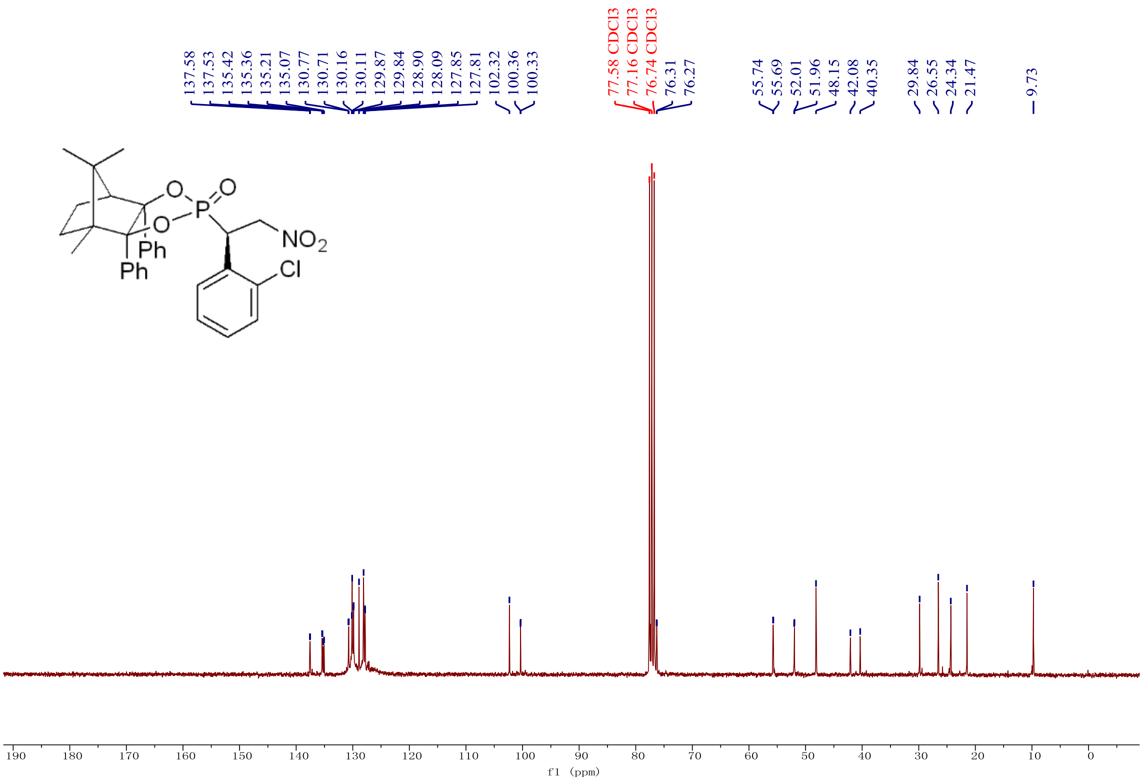

Fig. S251 ^{13}C NMR of compound **9e**

^{31}P NMR (121 MHz, $CDCl_3$)

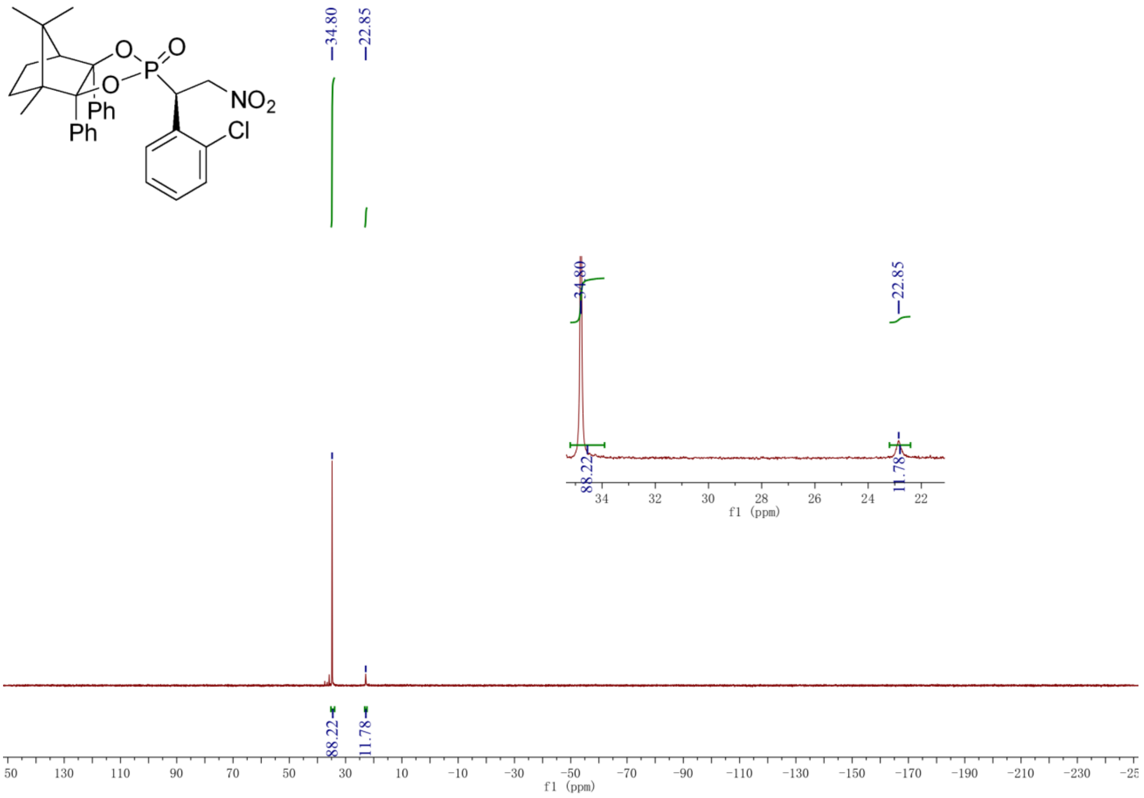

Fig. S252 ^{31}P NMR of compound **9e**

^1H NMR (300 MHz, $CDCl_3$)

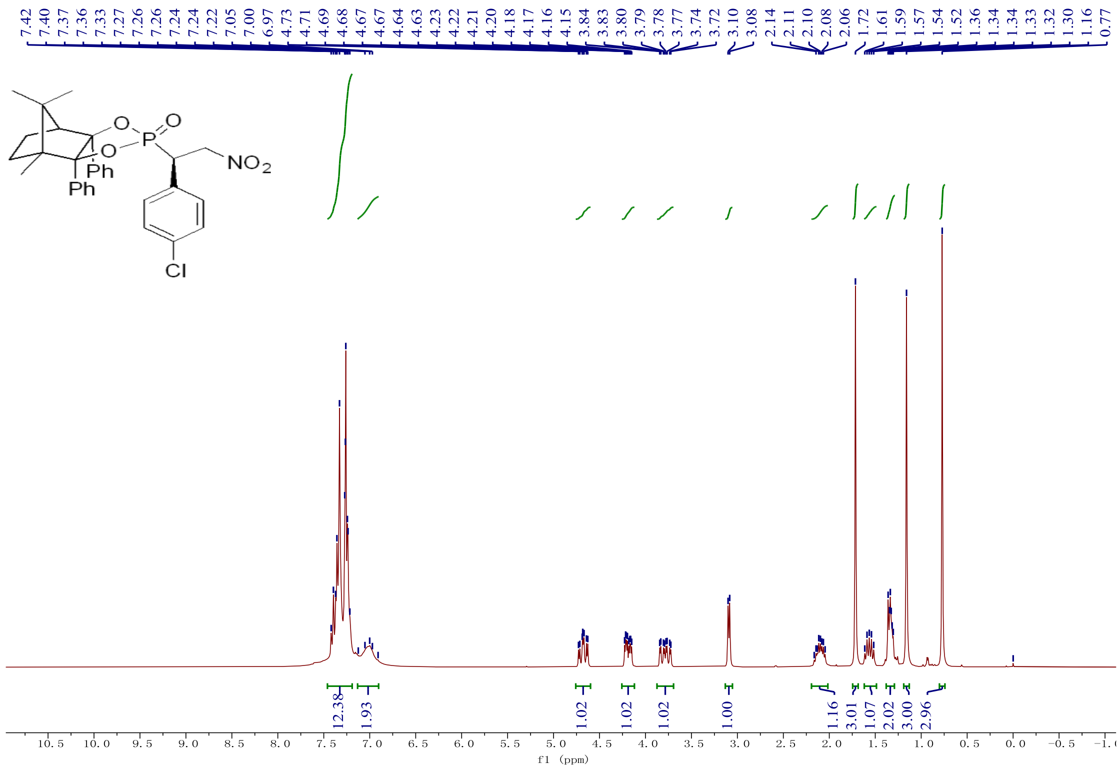

Fig. S253 ^1H NMR of compound **9f**

^{13}C NMR (75 MHz, $CDCl_3$)

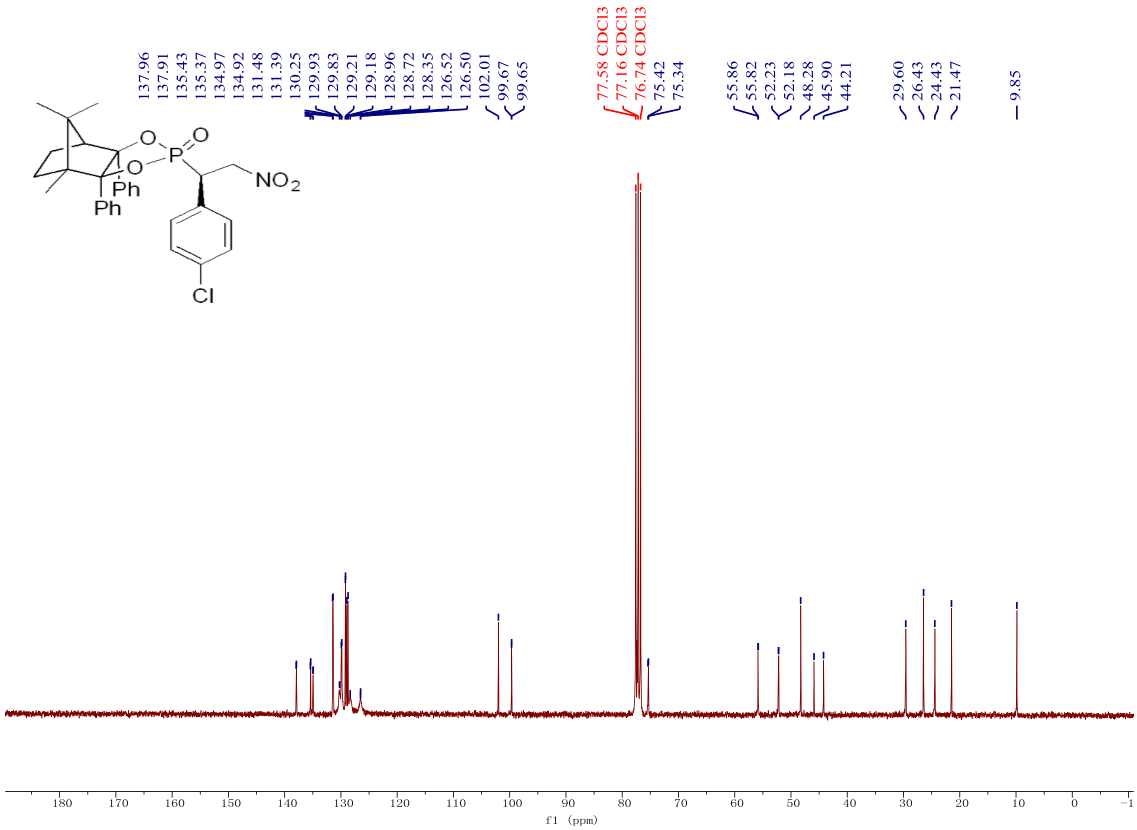

Fig. S254 ^{13}C NMR of compound **9f**

^{31}P NMR (121 MHz, $CDCl_3$)

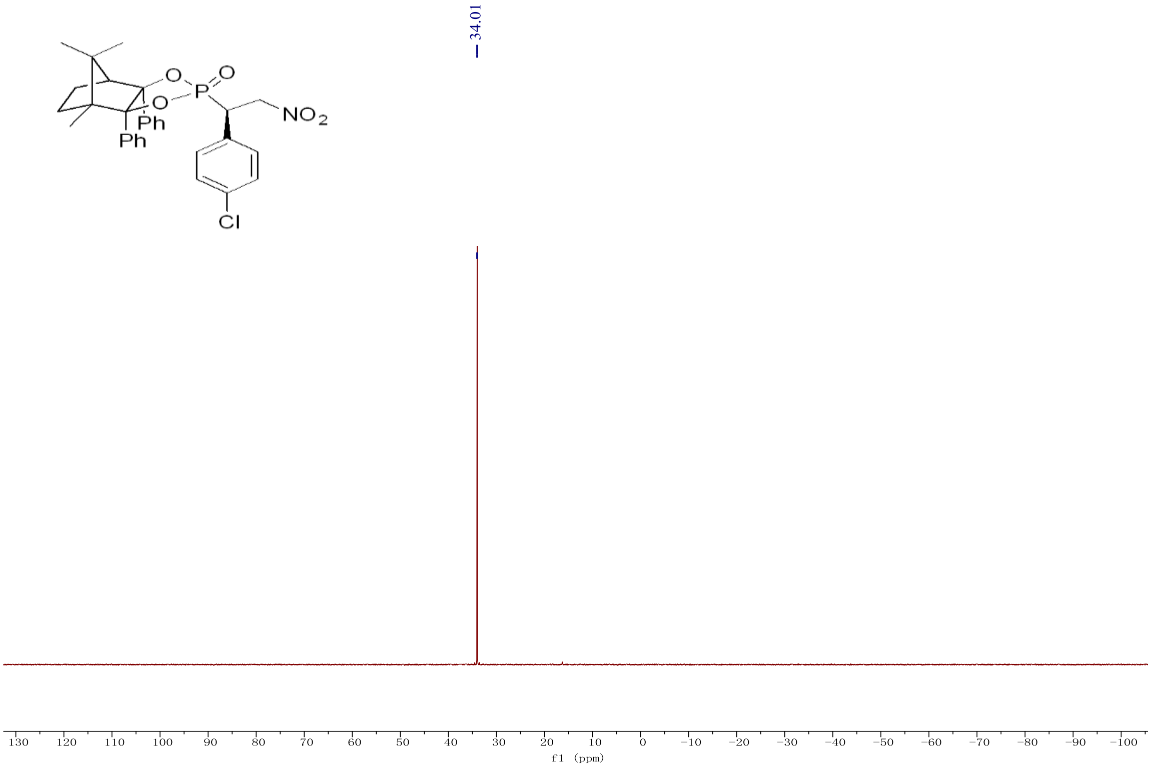

Fig. S255 ^{31}P NMR of compound **9f**

^{1}H NMR (300 MHz, $CDCl_3$)

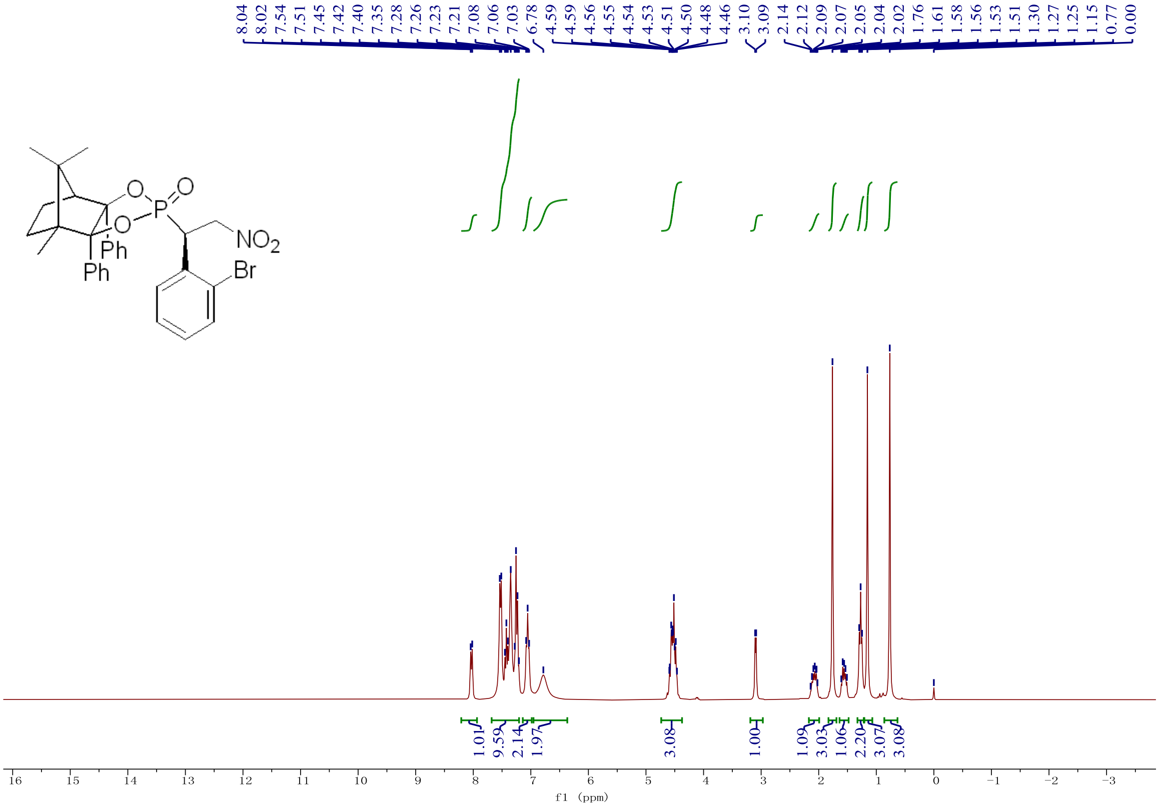

Fig. S256 ^{1}H NMR of compound **9g**

^{13}C NMR (75 MHz, $CDCl_3$)

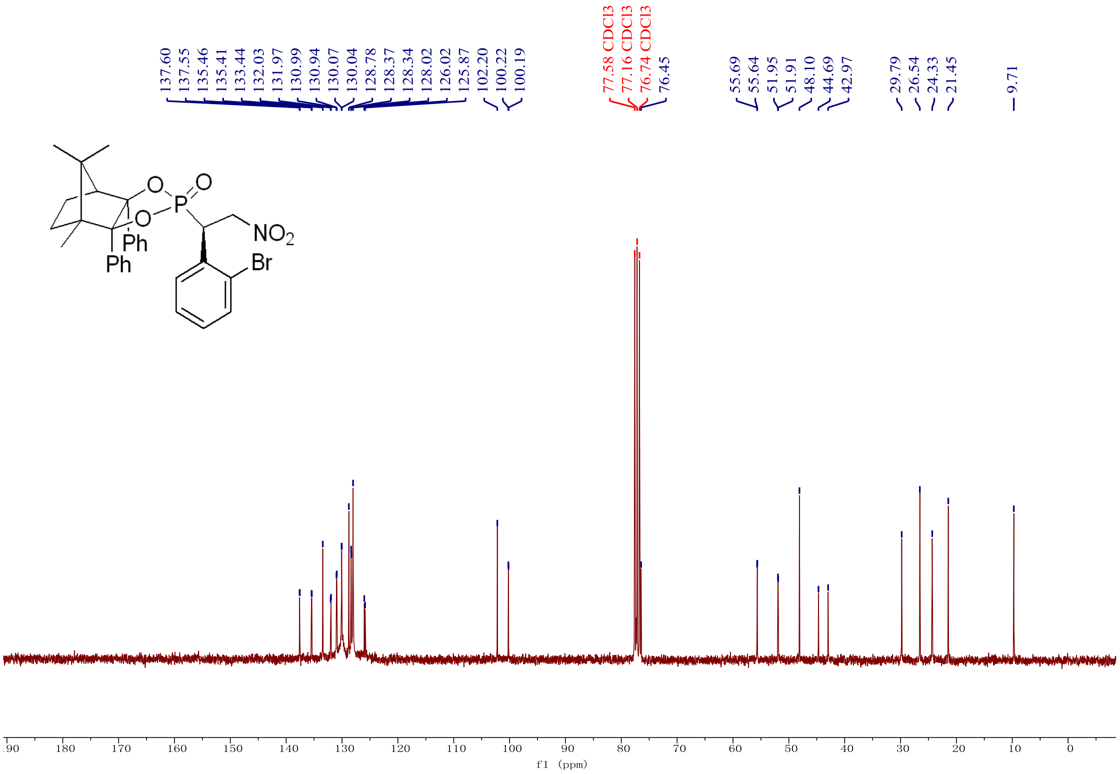

Fig. S257 ^{13}C NMR of compound **9g**

^{31}P NMR (121 MHz, $CDCl_3$)

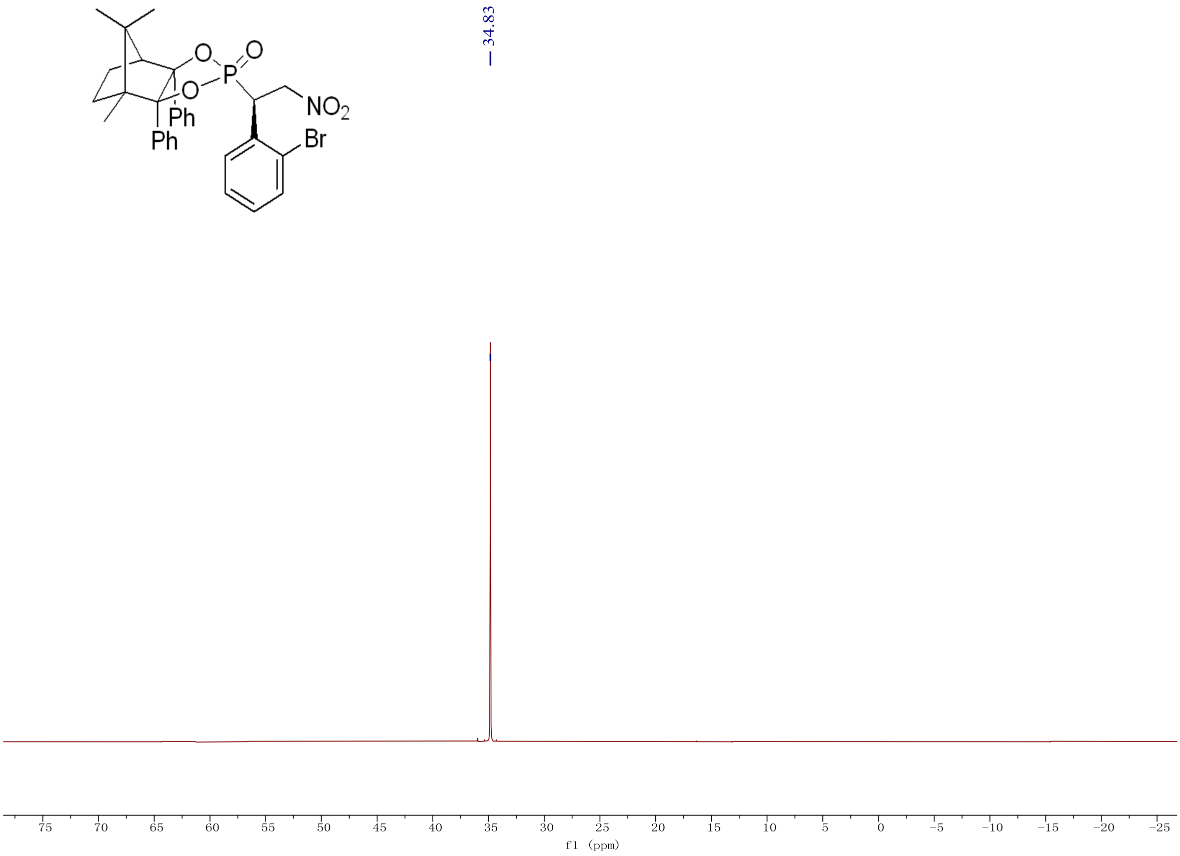

Fig. S258 ^{31}P NMR of compound **9g**

^{1}H NMR (300 MHz, $CDCl_3$)

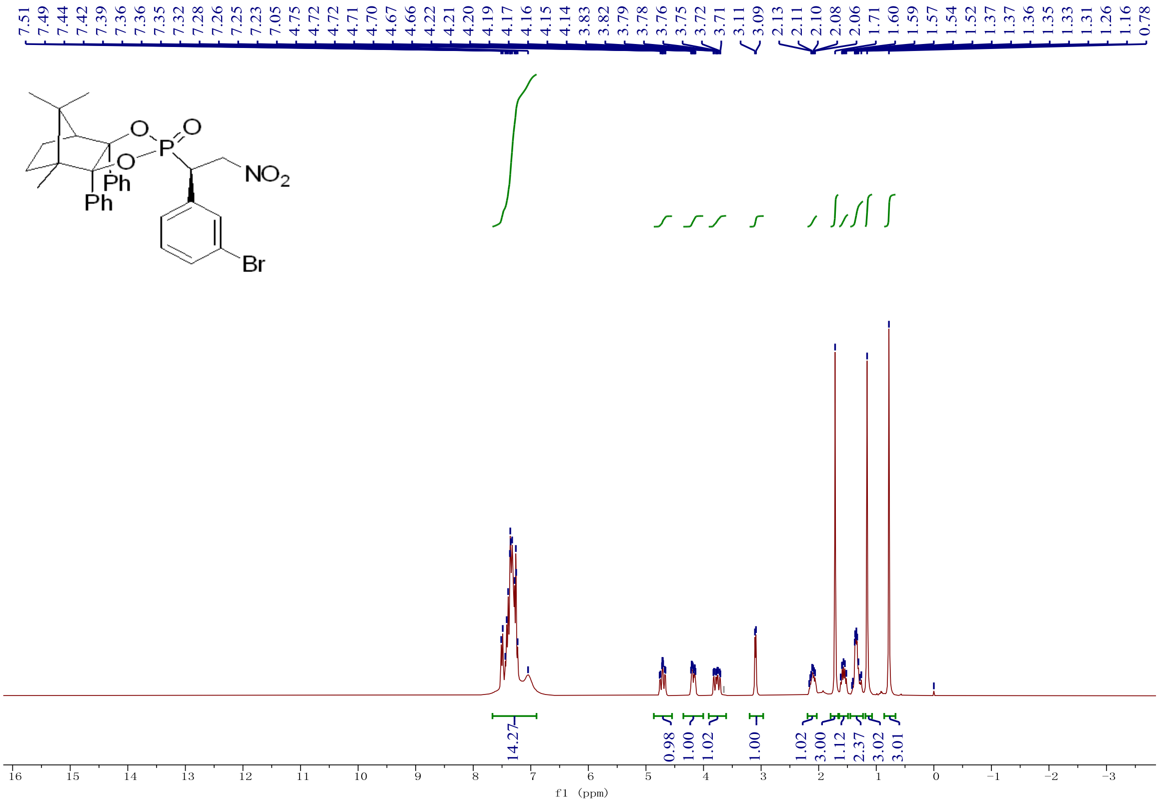

Fig. S259 ^{1}H NMR of compound **9h**

^{13}C NMR (75 MHz, $CDCl_3$)

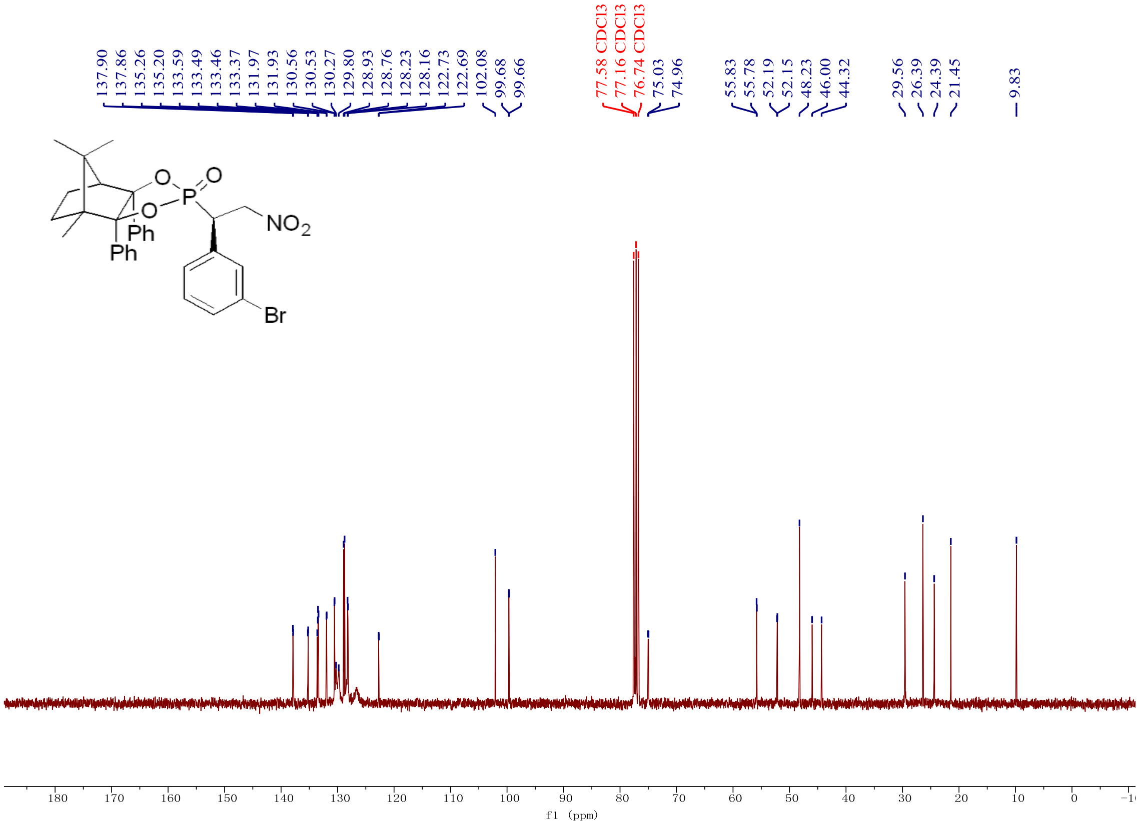

Fig. S260 ^{13}C NMR of compound **9h**

^{31}P NMR (121 MHz, $CDCl_3$)

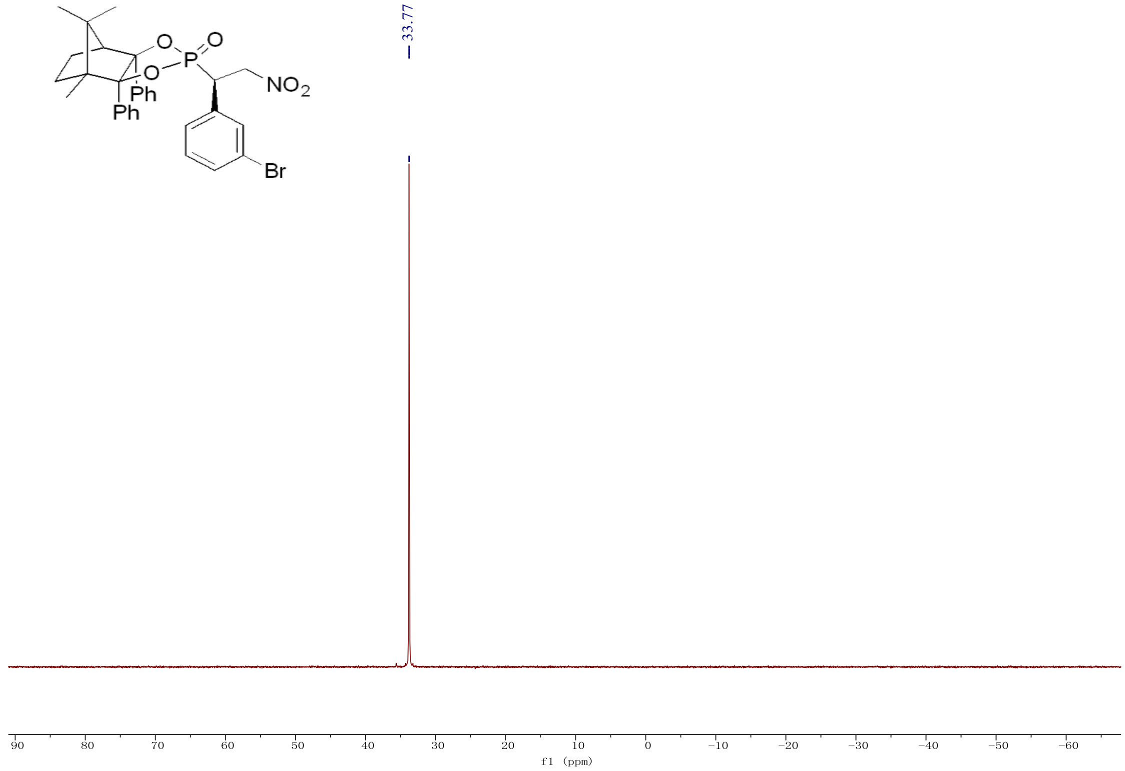

Fig. S261 ^{31}P NMR of compound **9h**

^{1}H NMR (300 MHz, $CDCl_3$)

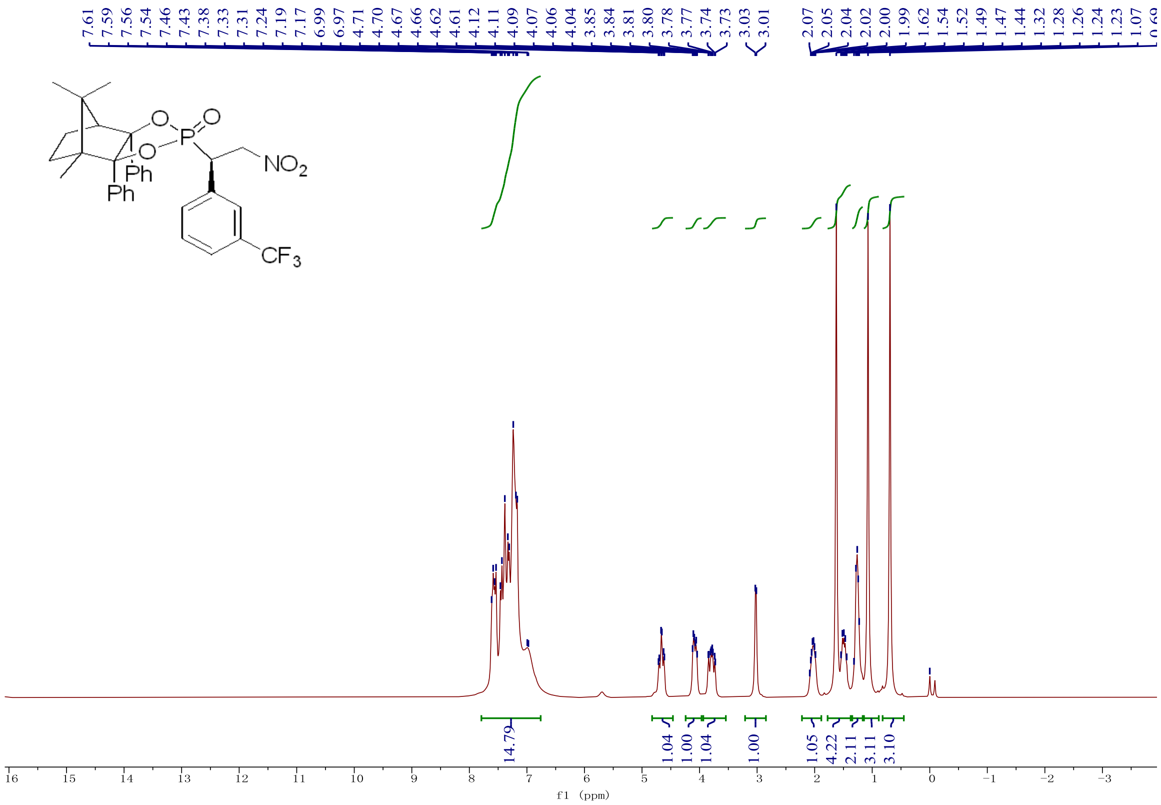

Fig. S262 ^{1}H NMR of compound **9i**

^{13}C NMR (75 MHz, $CDCl_3$)

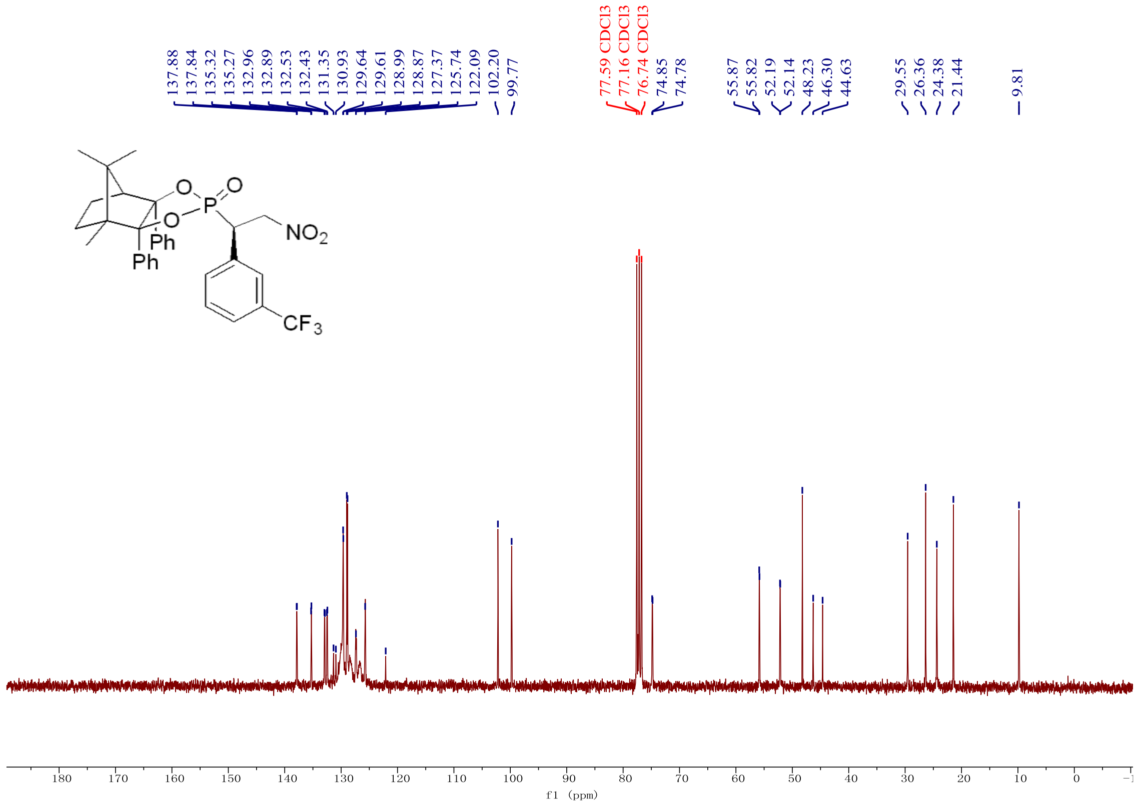

Fig. S263 ^{13}C NMR of compound **9i**

^{19}F NMR (282 MHz, $CDCl_3$)

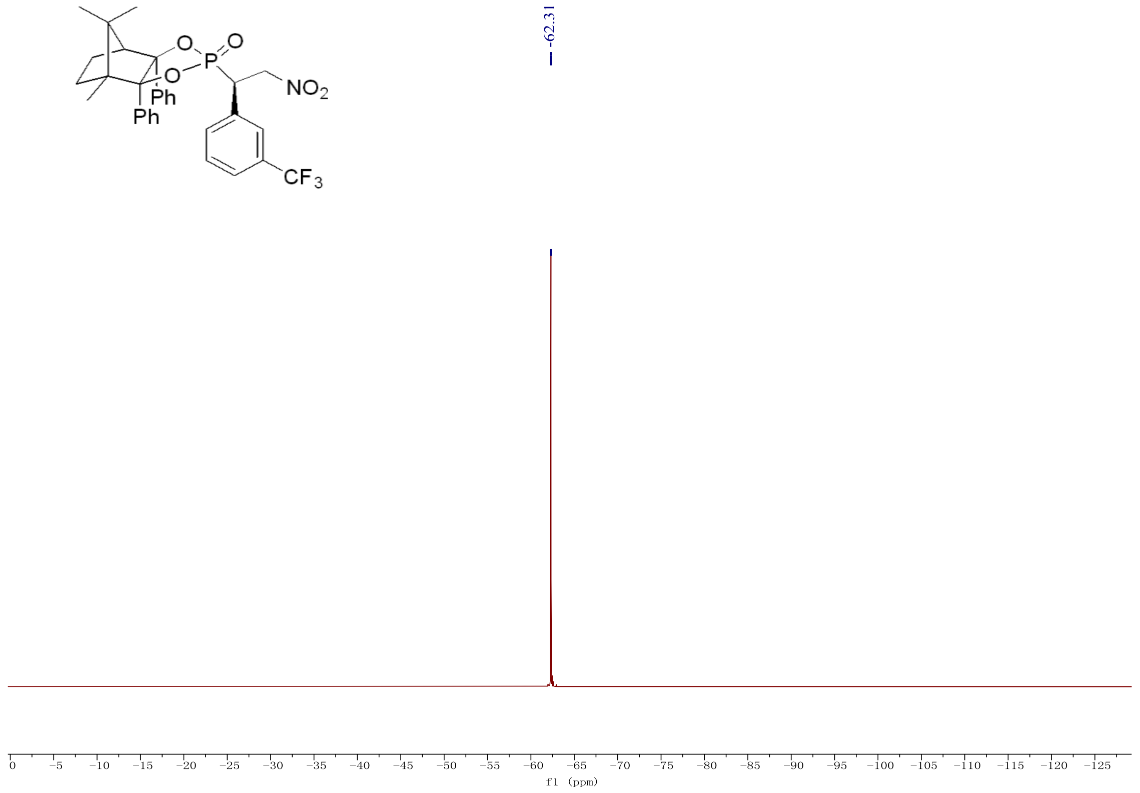

Fig. S264 ^{19}F NMR of compound **9i**

^{31}P NMR (121 MHz, $CDCl_3$)

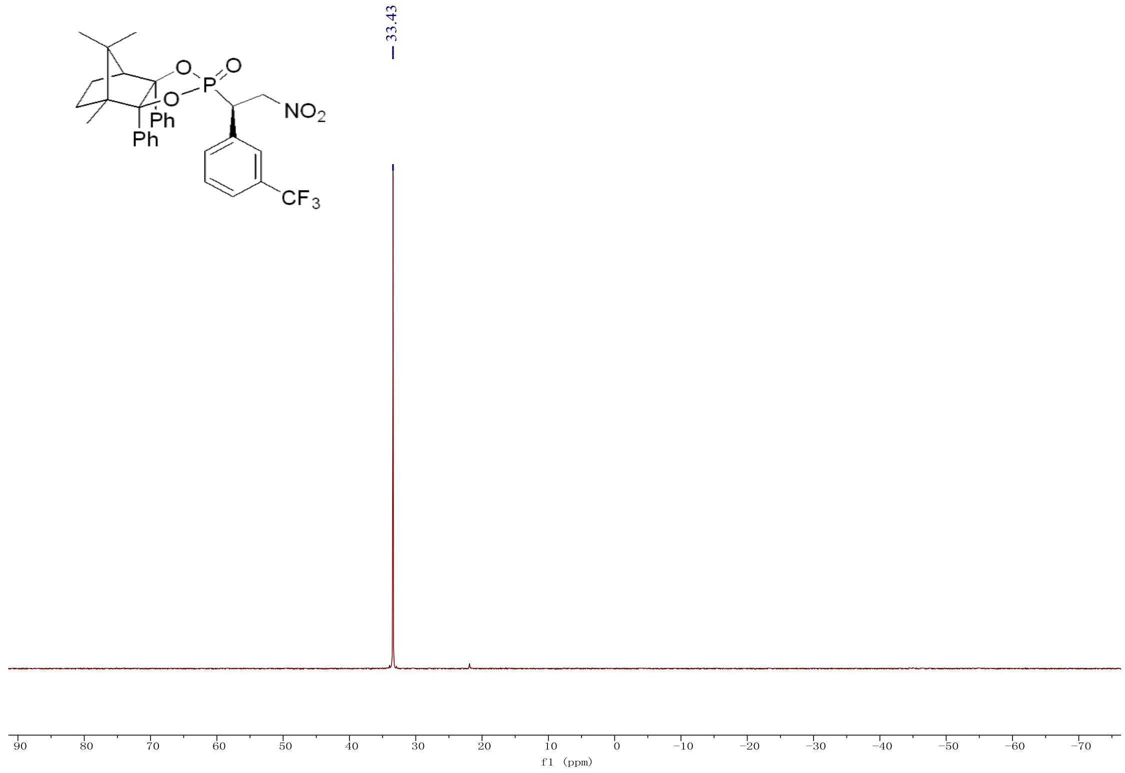

Fig. S265 ^{31}P NMR of compound **9i**

^{1}H NMR (300 MHz, $CDCl_3$)

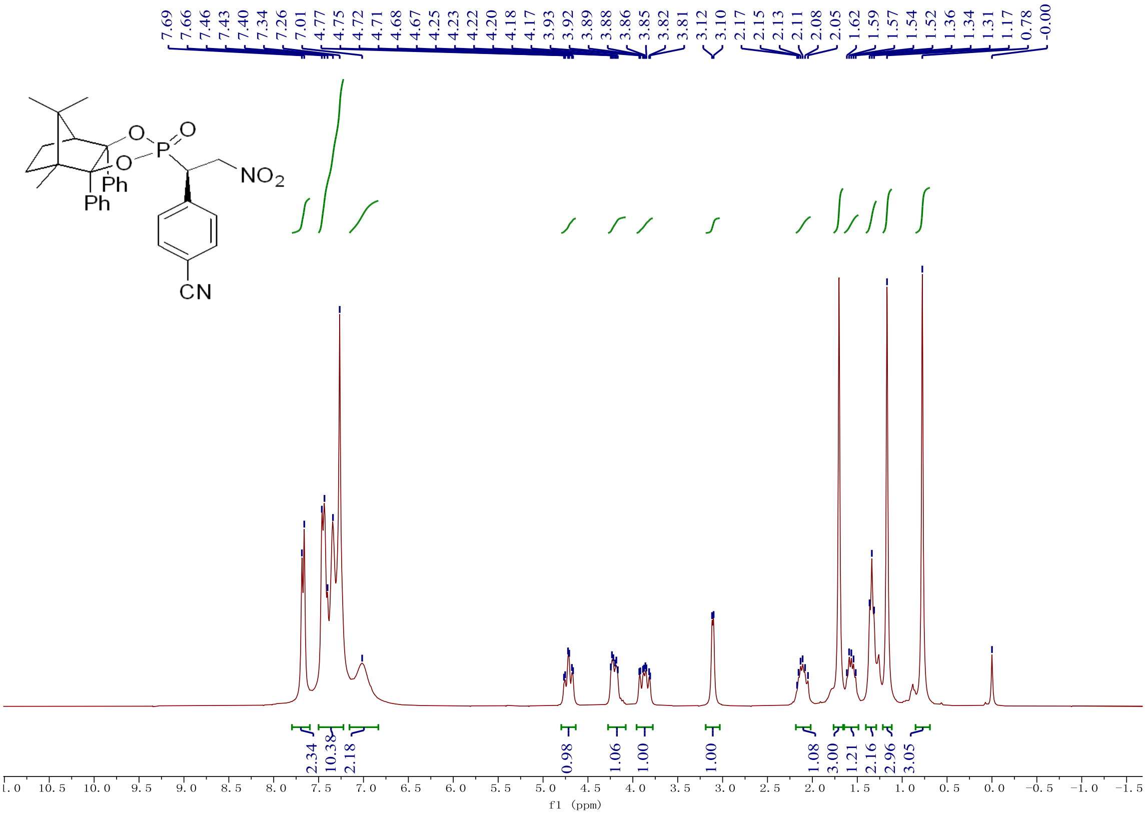

Fig. S266 ^{1}H NMR of compound **9j**

^{13}C NMR (75 MHz, $CDCl_3$)

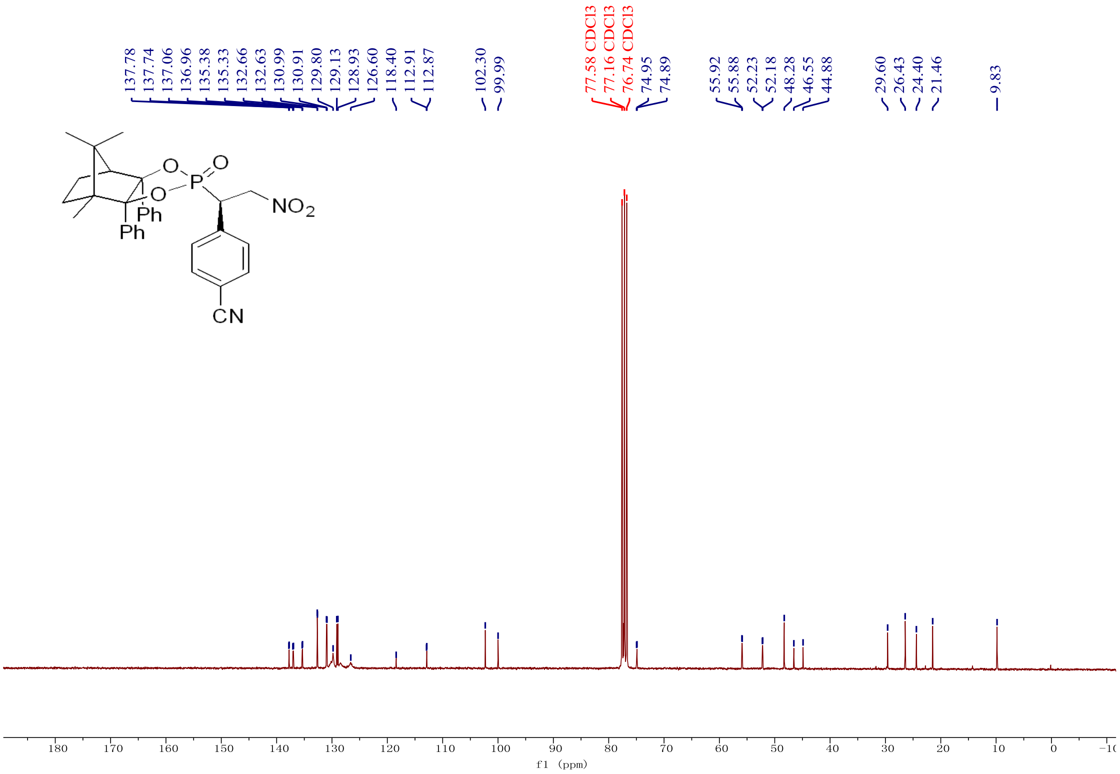

Fig. S267 ^{13}C NMR of compound **9j**

^{31}P NMR (121 MHz, $CDCl_3$)

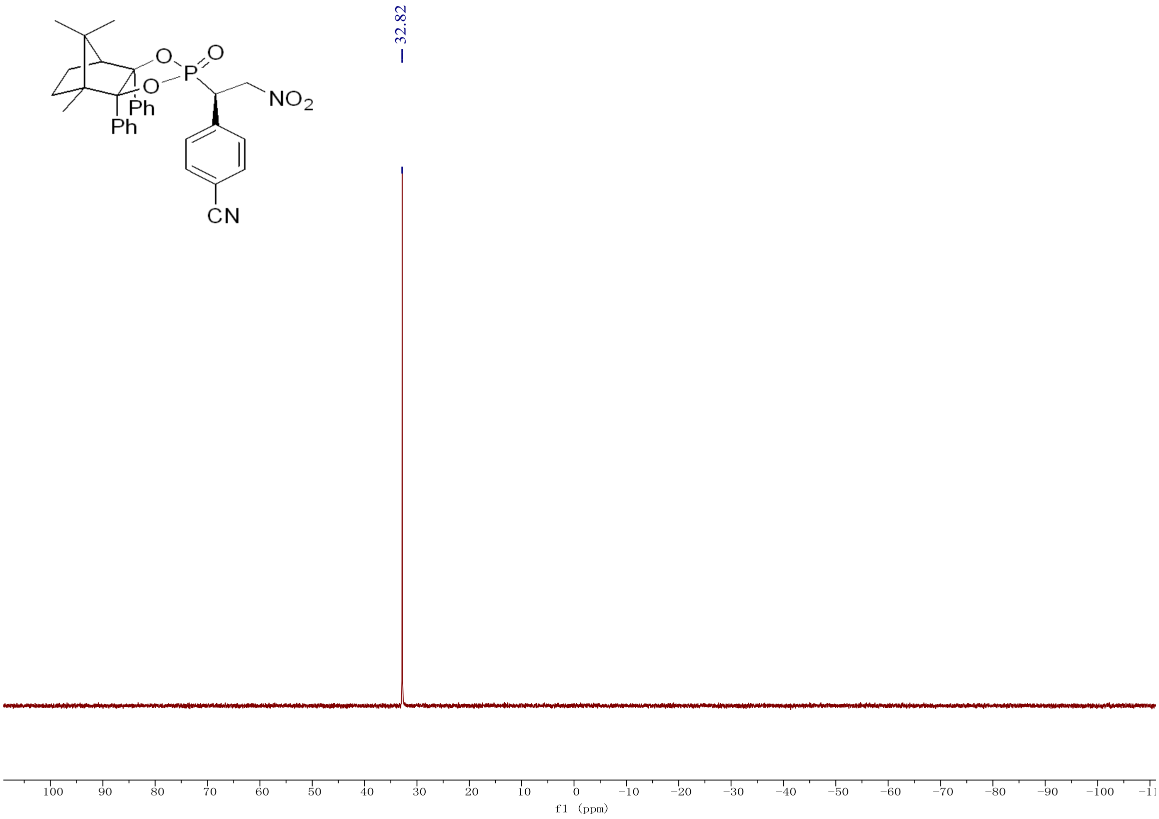

Fig. S268 ^{31}P NMR of compound **9j**

^{1}H NMR (300 MHz, $CDCl_3$)

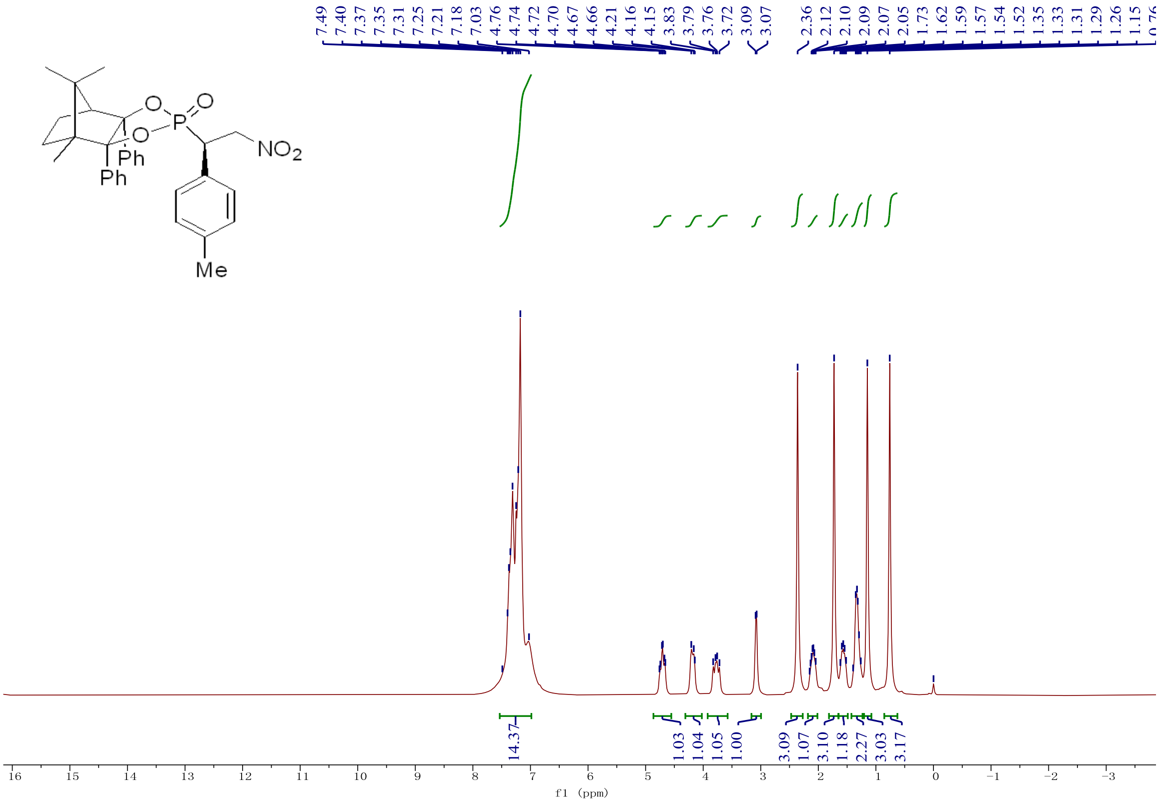

Fig. S269 ^{1}H NMR of compound **9k**

^{13}C NMR (75 MHz, $CDCl_3$)

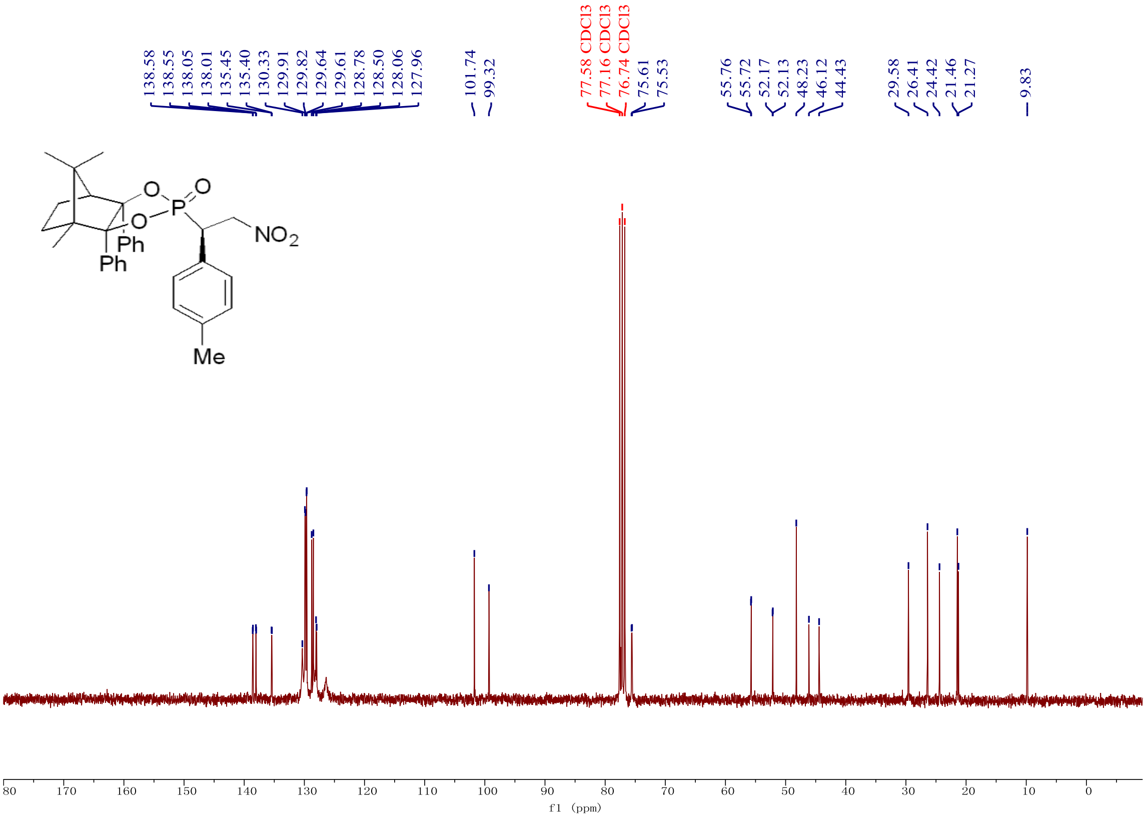

Fig. S270 ^{13}C NMR of compound **9k**

^{31}P NMR (121 MHz, $CDCl_3$)

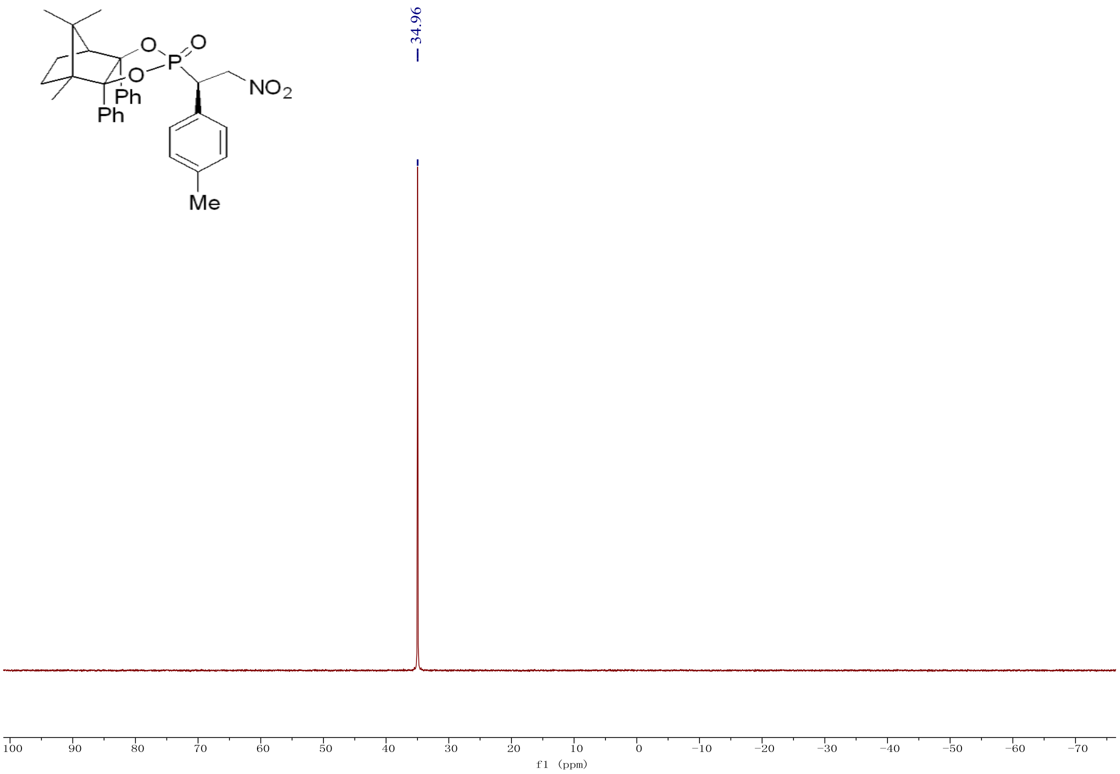

Fig. S271 ^{31}P NMR of compound **9k**

^{1}H NMR (300 MHz, $CDCl_3$)

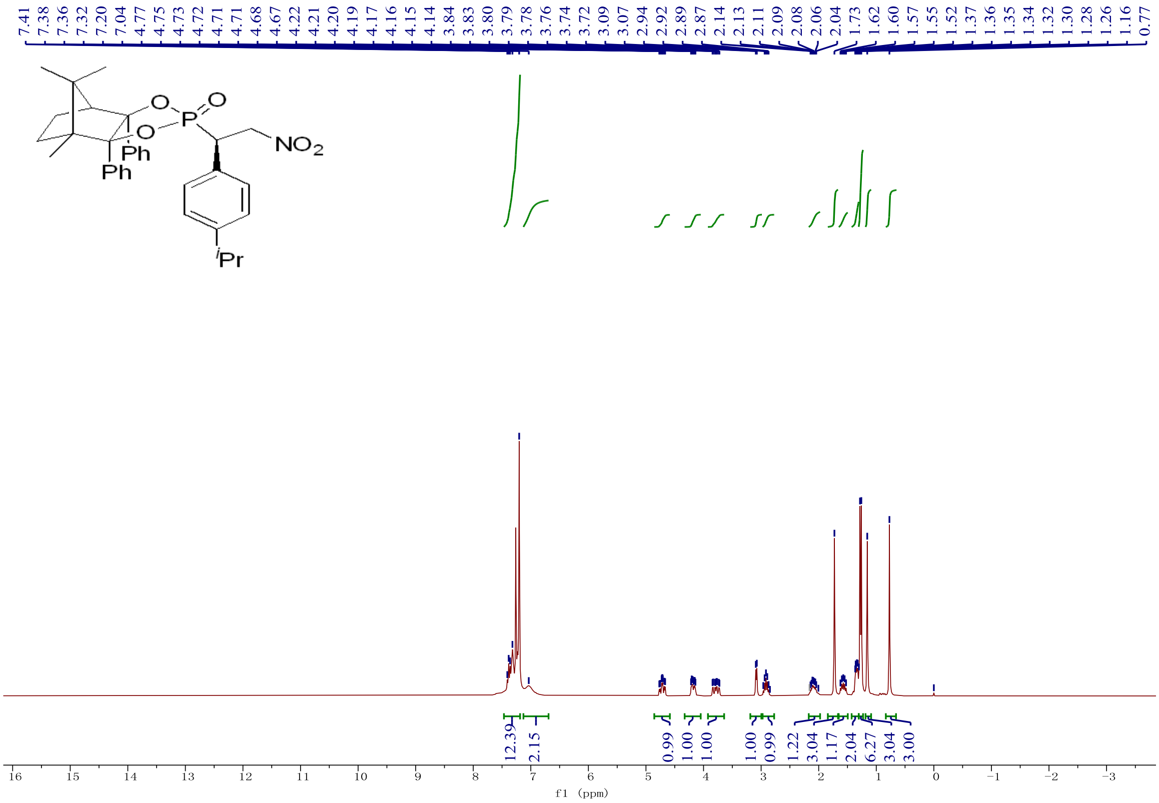

Fig. S272 ^{1}H NMR of compound **9l**

^{13}C NMR (75 MHz, $CDCl_3$)

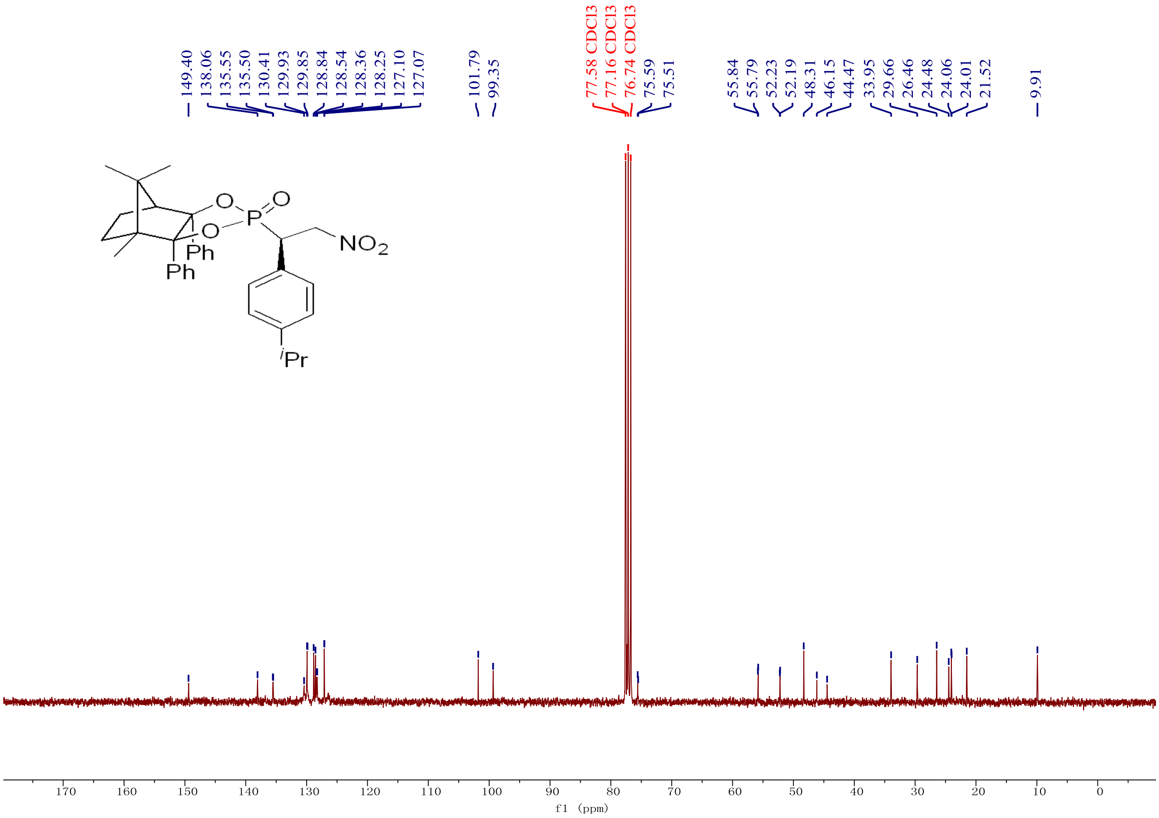

Fig. S273 ^{13}C NMR of compound **9l**

^{31}P NMR (121 MHz, $CDCl_3$)

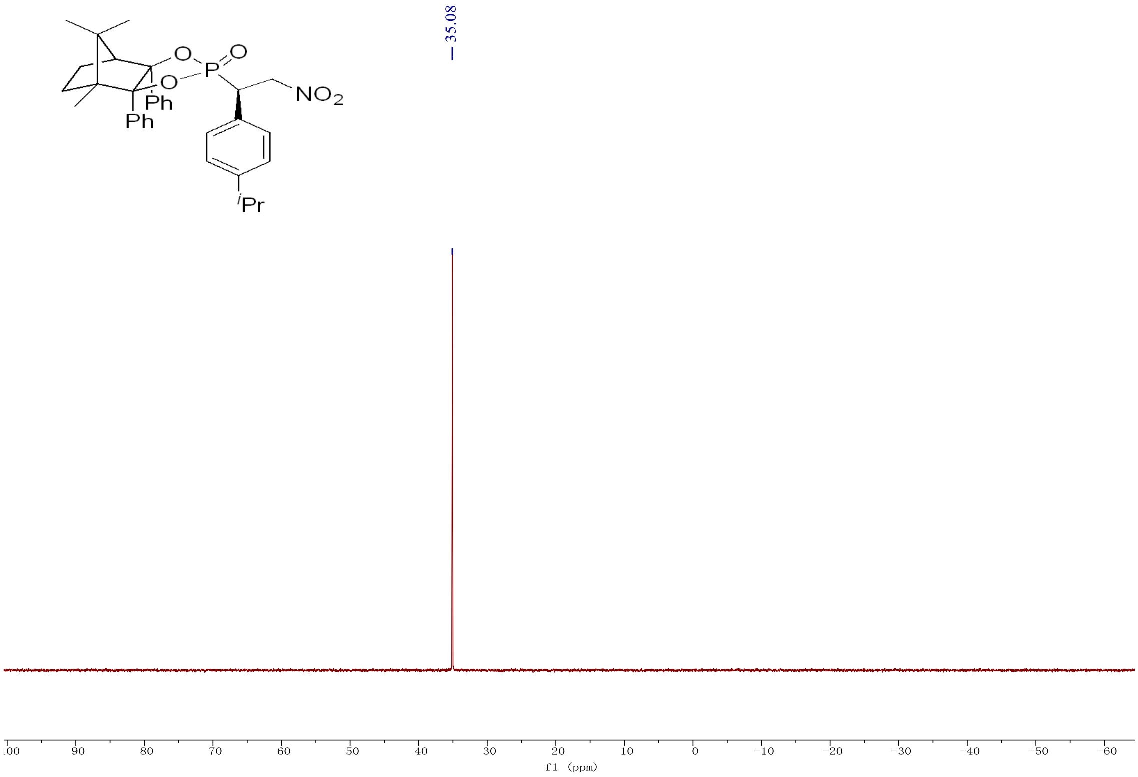

Fig. S274 ^{31}P NMR of compound **9l**

^{1}H NMR (300 MHz, $CDCl_3$)

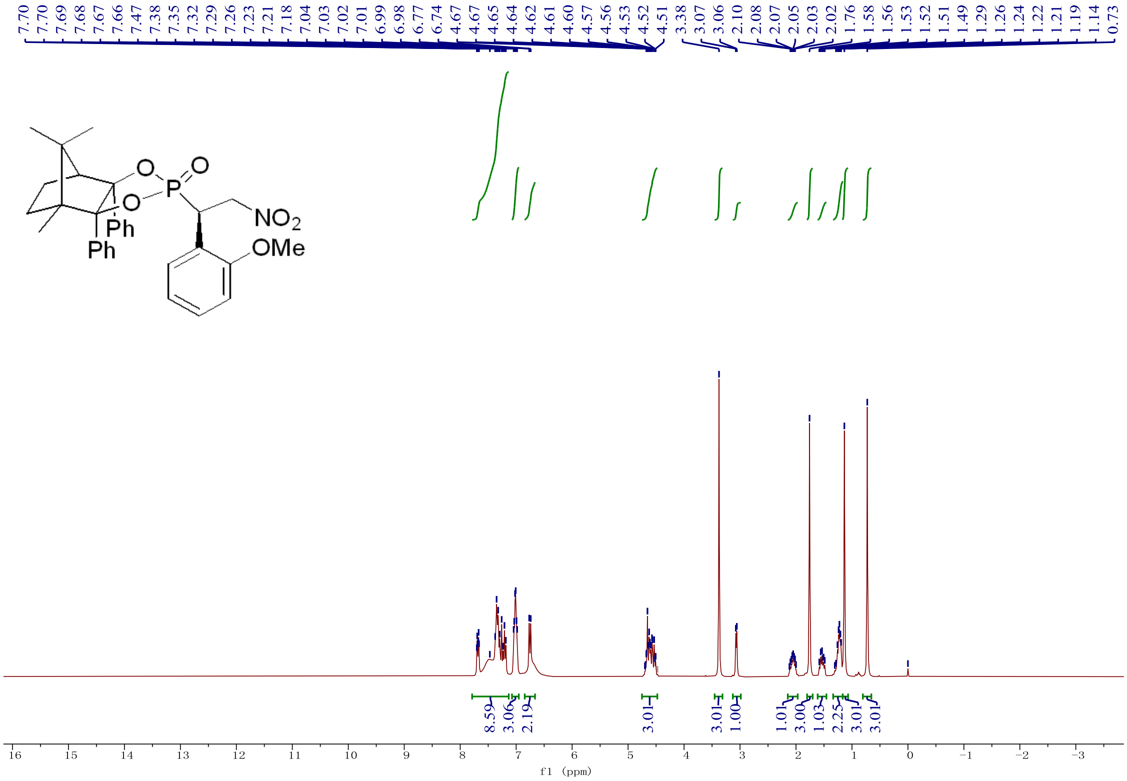

Fig. S275 ^{1}H NMR of compound **9m**

^{13}C NMR (75 MHz, $CDCl_3$)

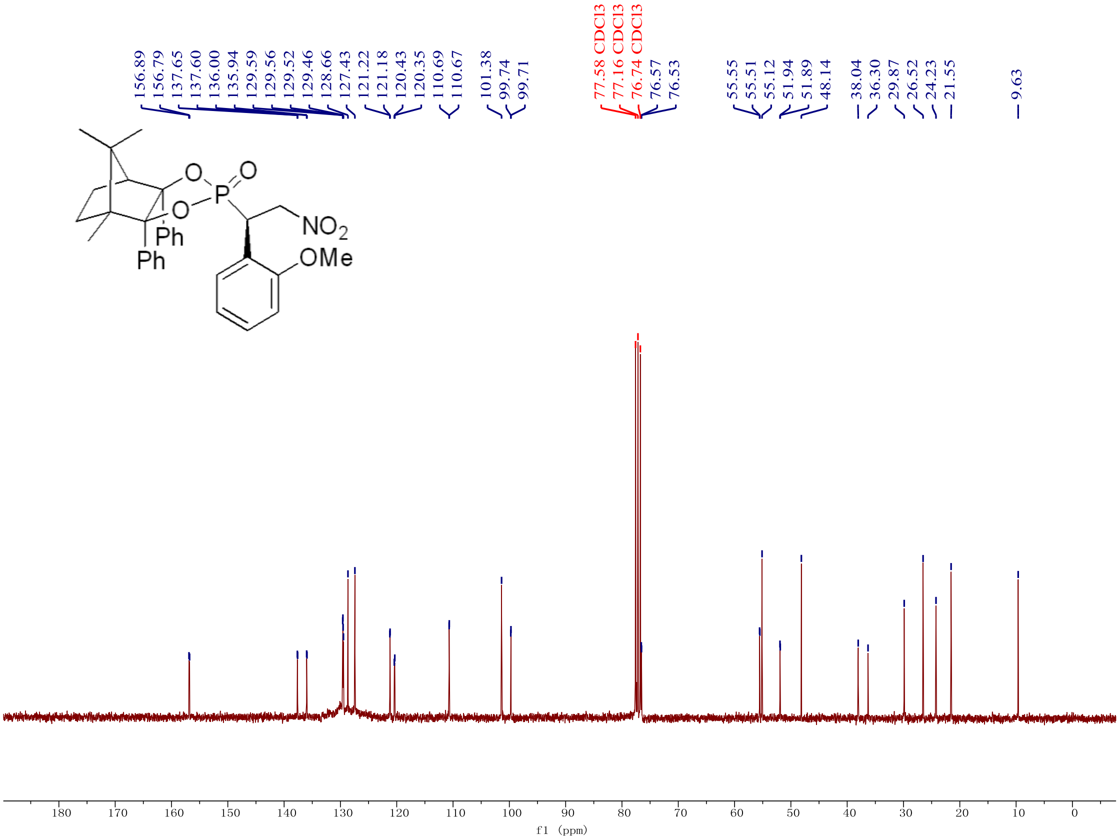

Fig. S276 ^{13}C NMR of compound **9m**

^{31}P NMR (121 MHz, $CDCl_3$)

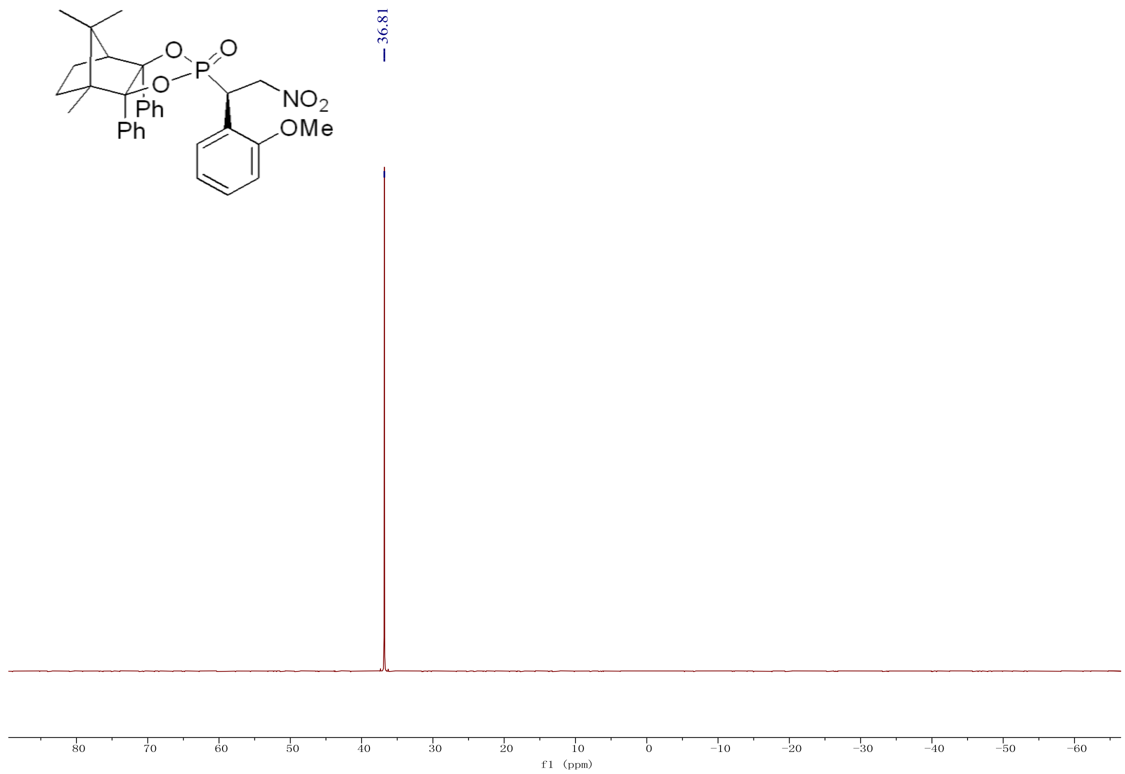

Fig. S277 ^{31}P NMR of compound **9m**

^1H NMR (300 MHz, $CDCl_3$)

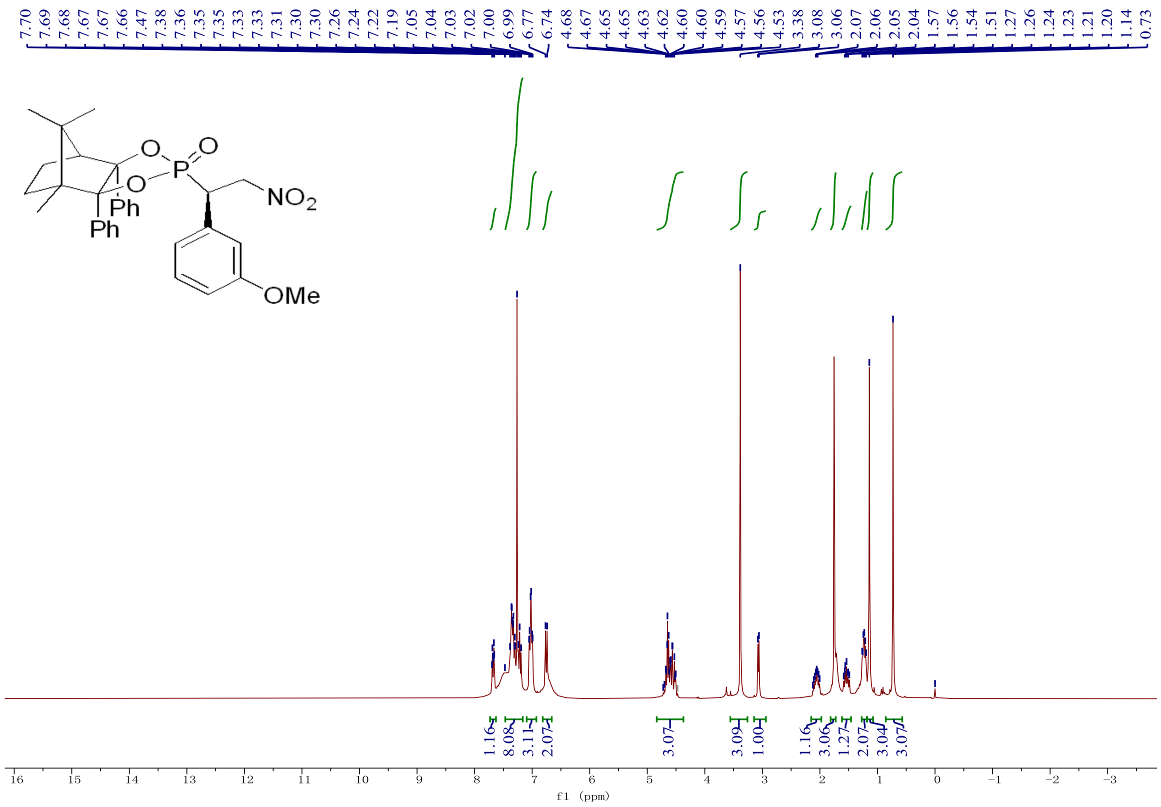

Fig. S278 ^1H NMR of compound **9n**

^{13}C NMR (75 MHz, $CDCl_3$)

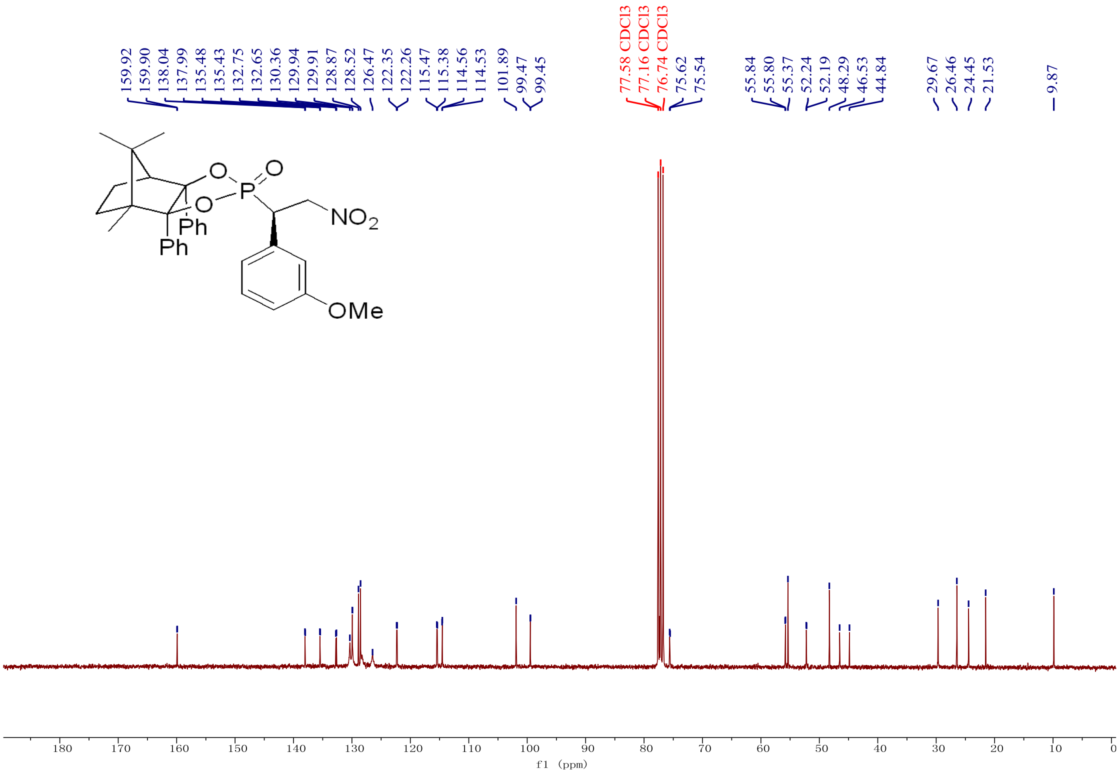

Fig. S279 ^{13}C NMR of compound **9n**

^{31}P NMR (121 MHz, $CDCl_3$)

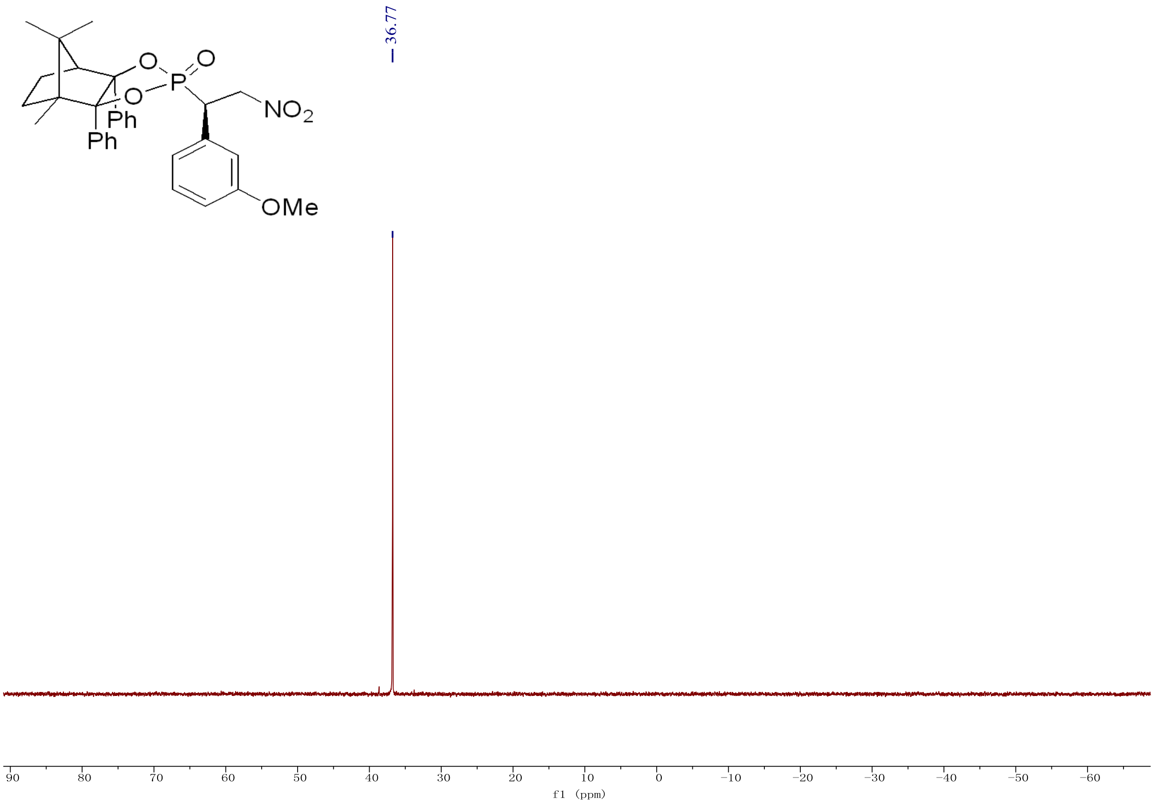

Fig. S280 ^{31}P NMR of compound **9n**

^{1}H NMR (300 MHz, $CDCl_3$)

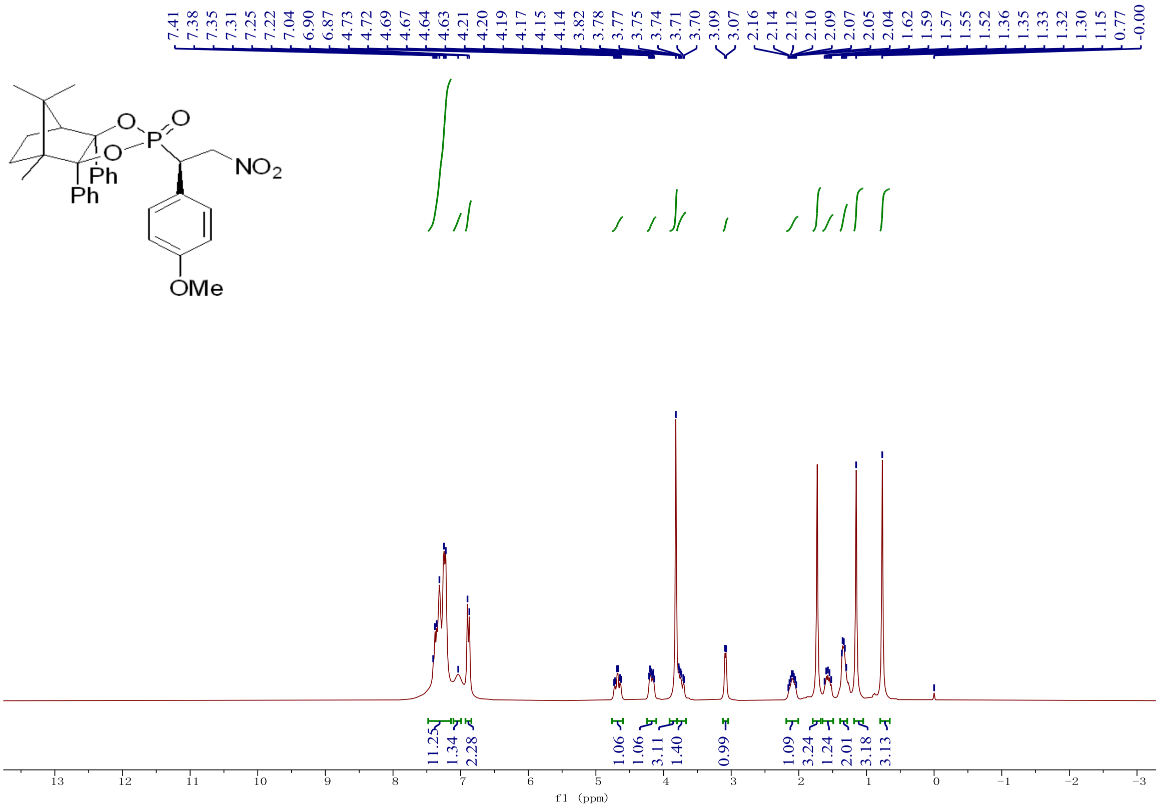

Fig. S281 ^{1}H NMR of compound **9o**

^{13}C NMR (75 MHz, $CDCl_3$)

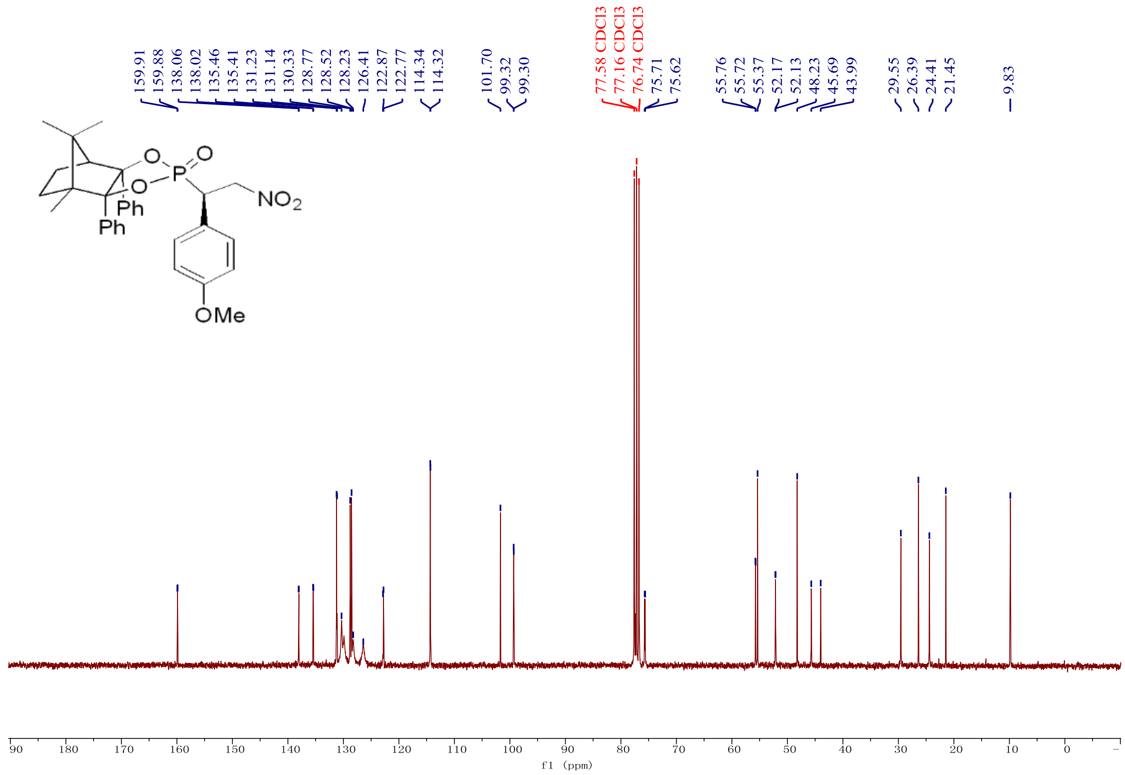

Fig. S282 ^{13}C NMR of compound **9o**

^{31}P NMR (121 MHz, $CDCl_3$)

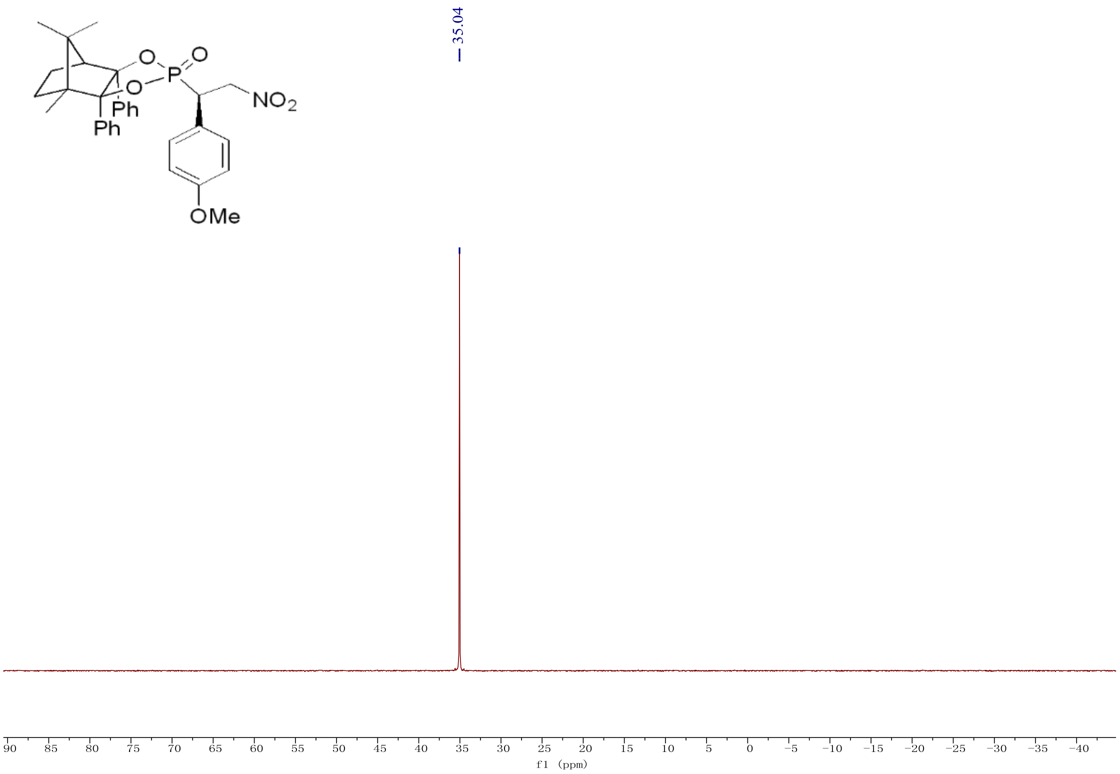

Fig. S283 ^{31}P NMR of compound **9o**

^{1}H NMR (300 MHz, $CDCl_3$)

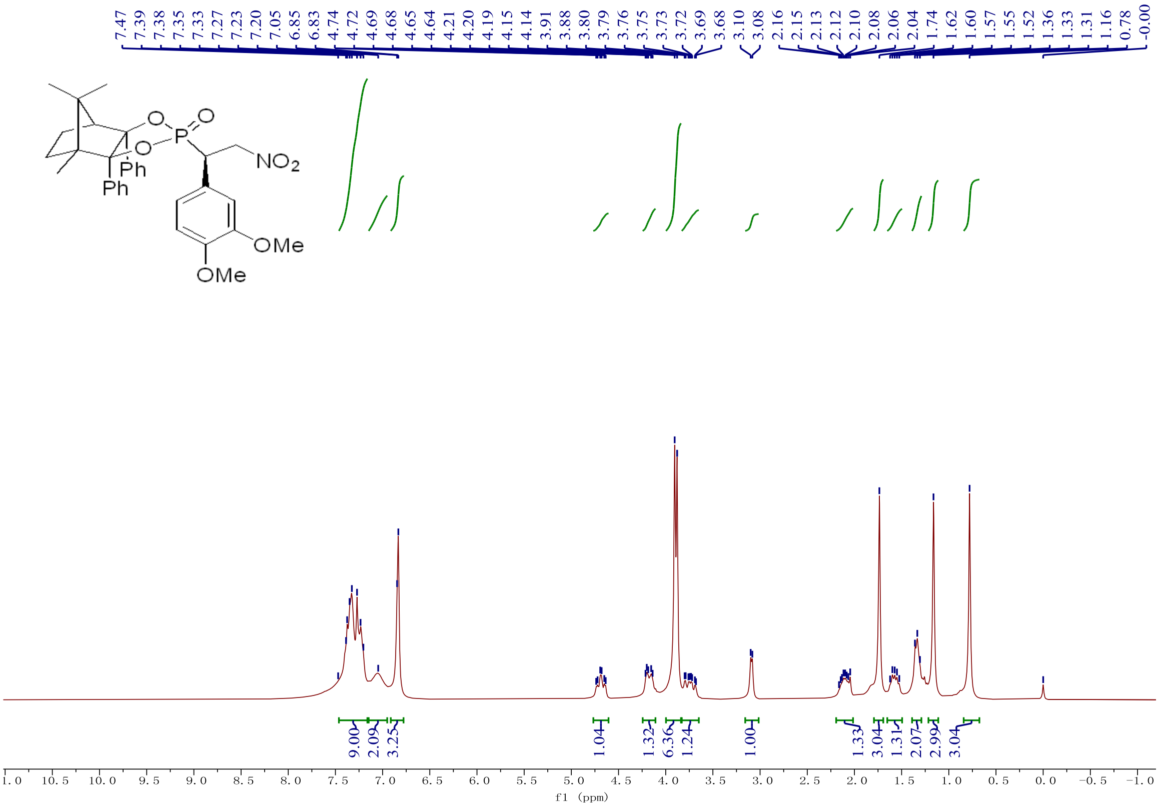

Fig. S284 ^{1}H NMR of compound **9p**

^{13}C NMR (75 MHz, $CDCl_3$)

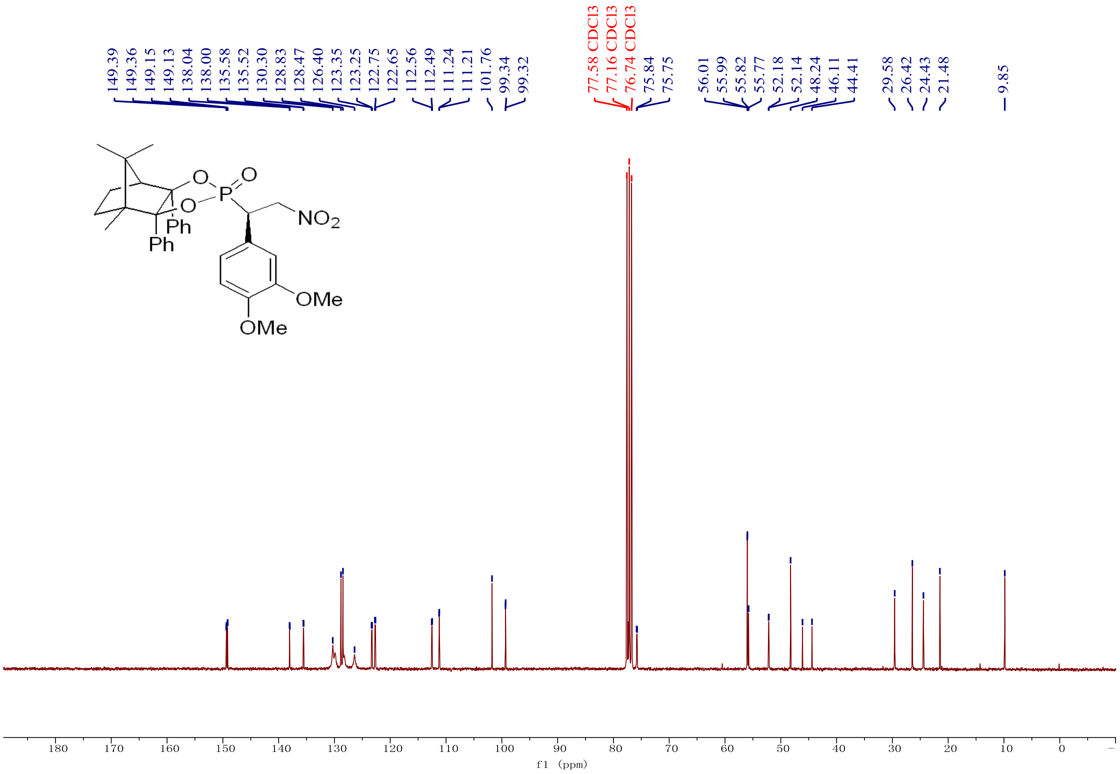

Fig. S285 ^{13}C NMR of compound **9p**

^{31}P NMR (121 MHz, $CDCl_3$)

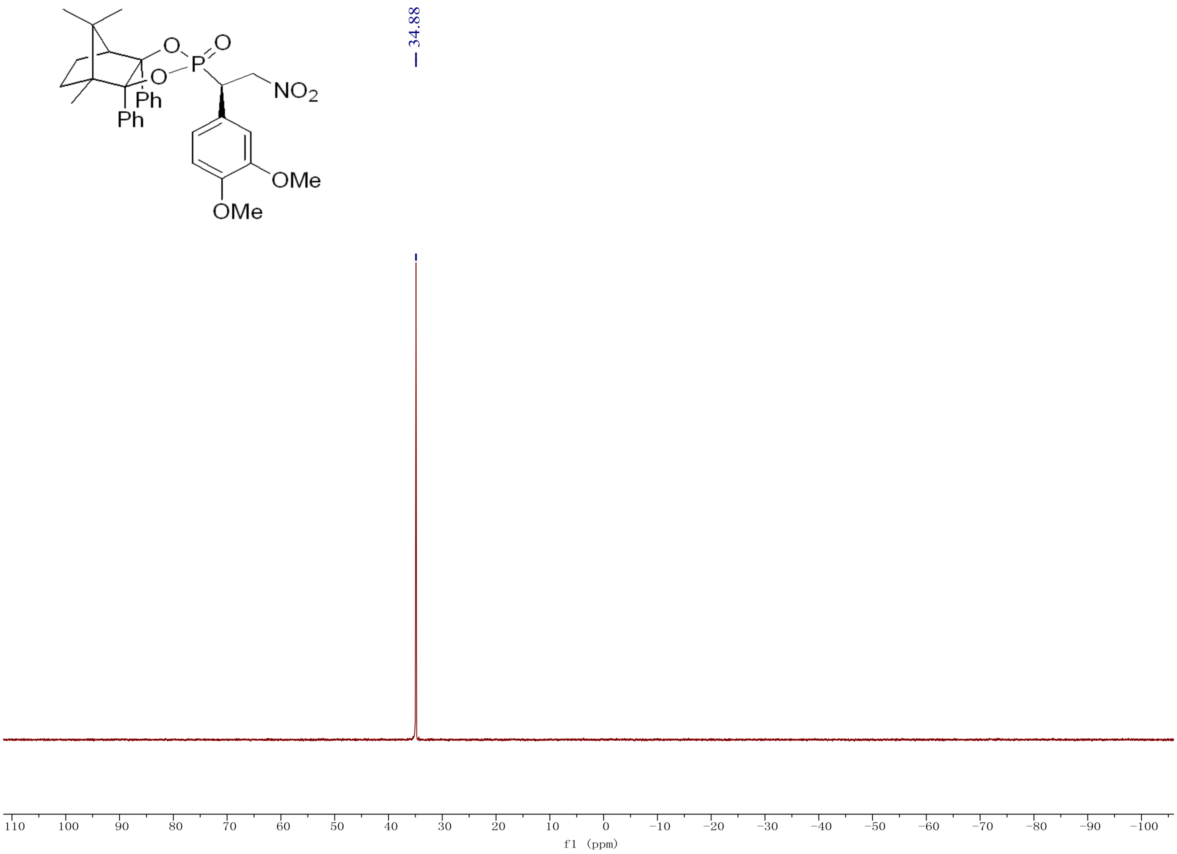

Fig. S286 ^{31}P NMR of compound **9p**

^{1}H NMR (300 MHz, $CDCl_3$)

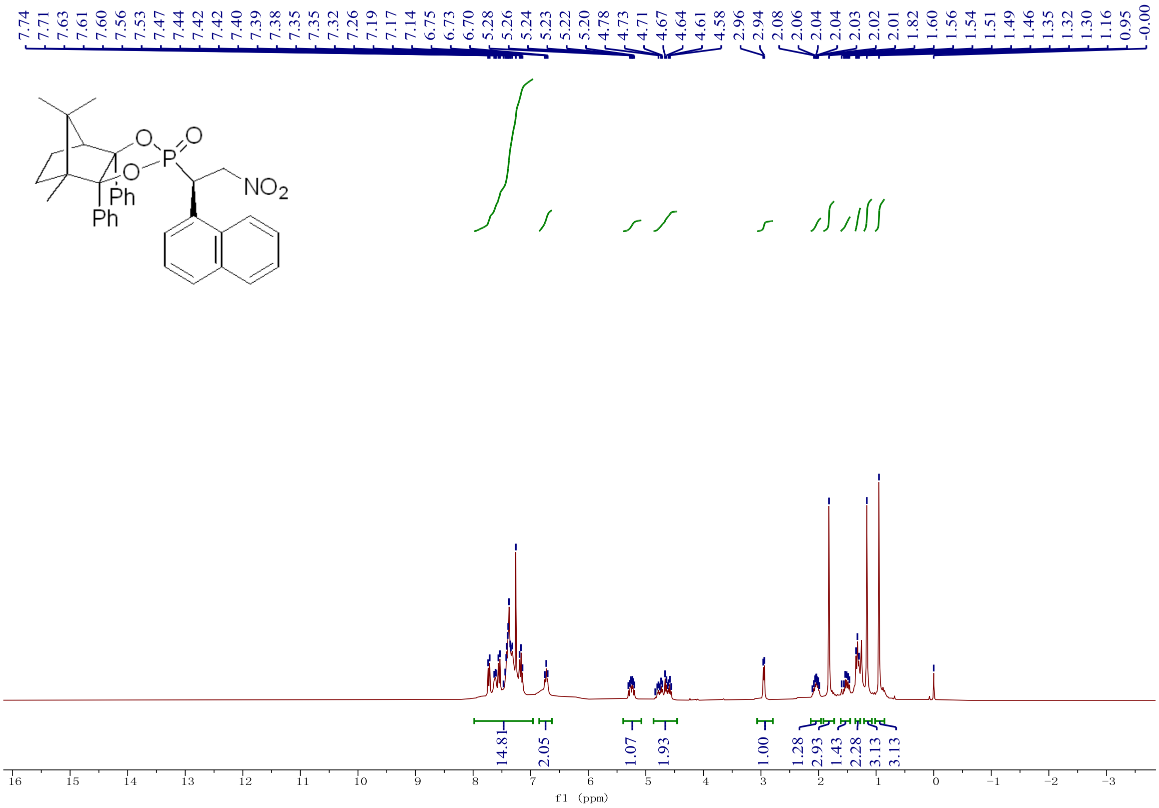

Fig. S287 ^{1}H NMR of compound **9q**

^{13}C NMR (75 MHz, $CDCl_3$)

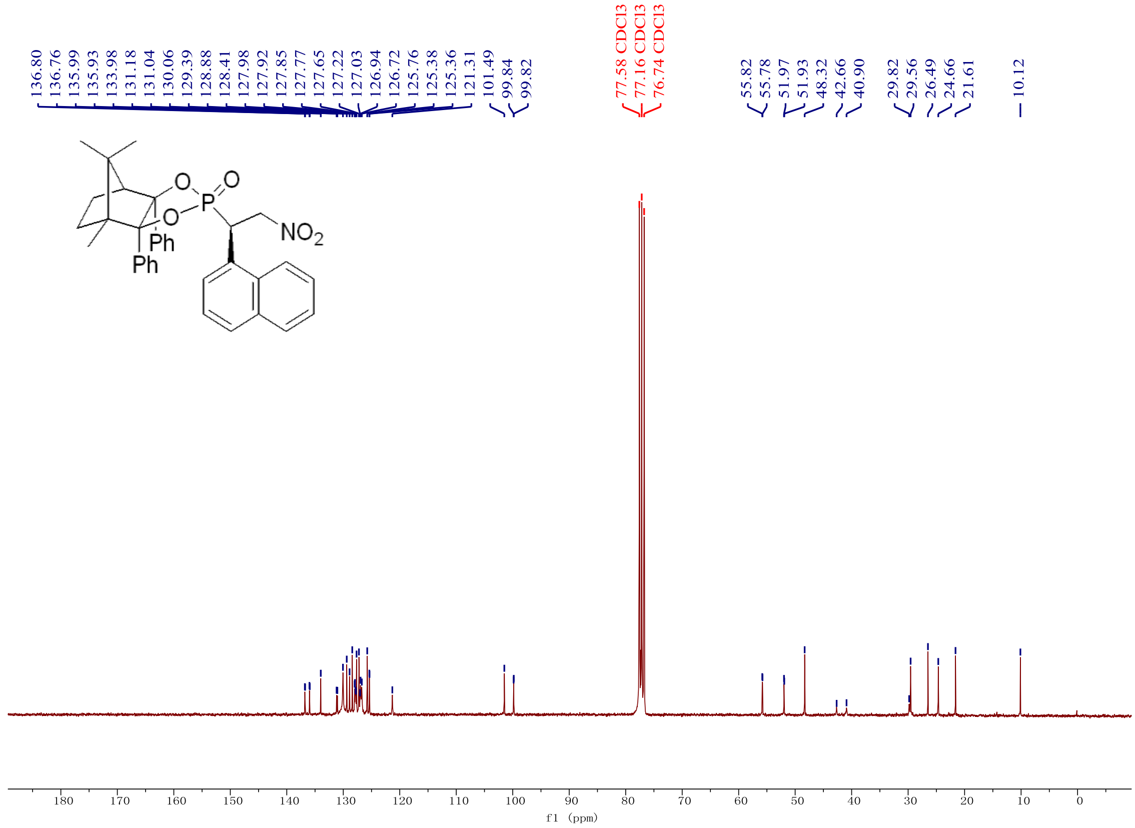

Fig. S288 ^{13}C NMR of compound **9q**

^{31}P NMR (121 MHz, $CDCl_3$)

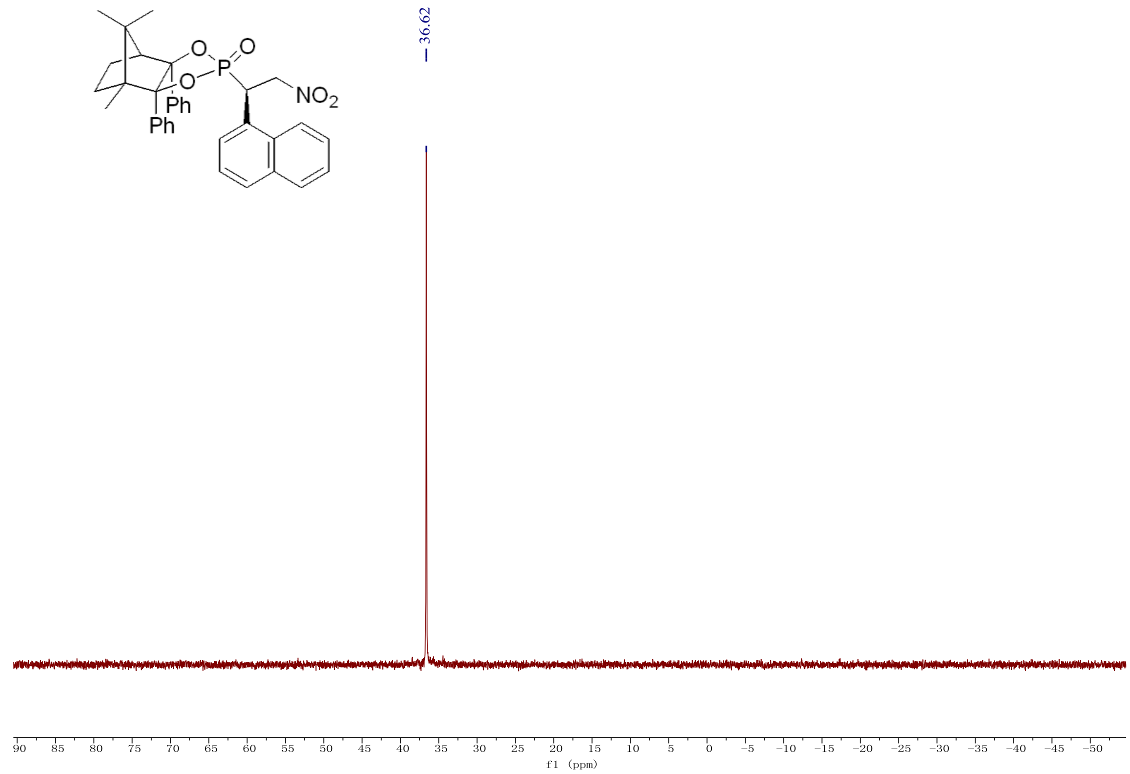

Fig. S289 ^{31}P NMR of compound **9q**

^{1}H NMR (300 MHz, $CDCl_3$)

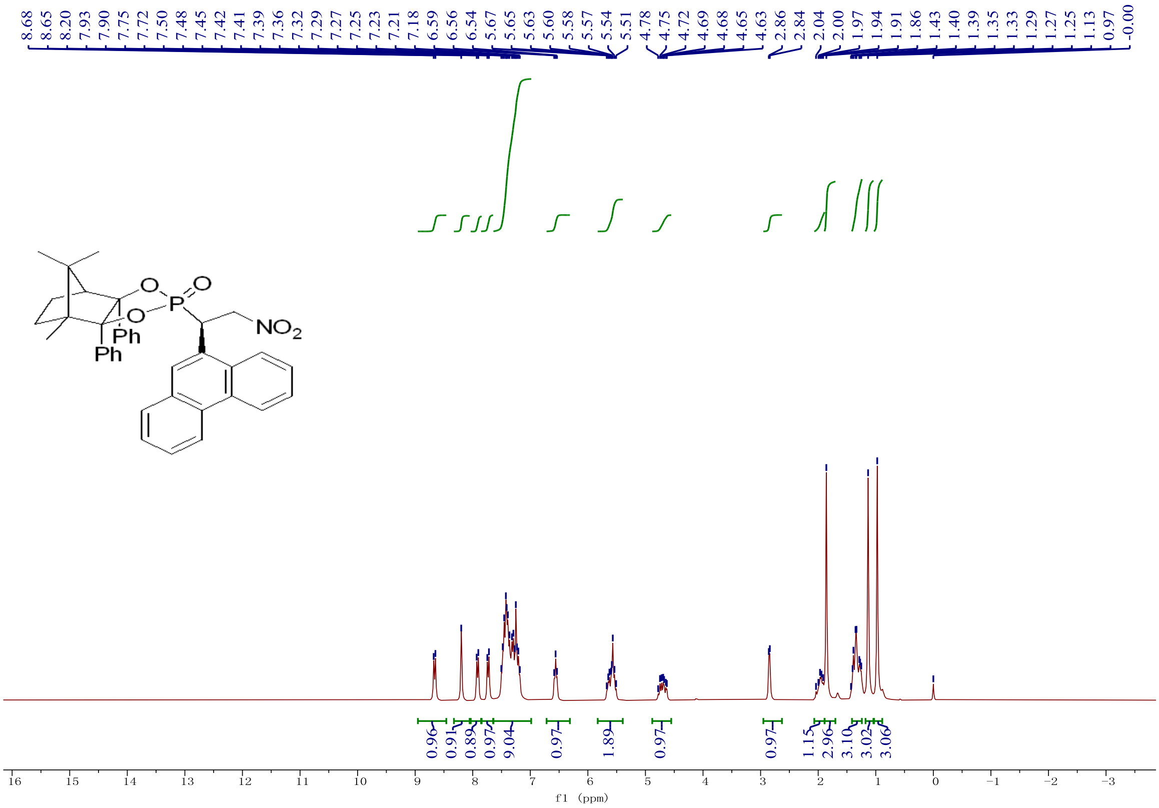

Fig. S290 ^{1}H NMR of compound **9r**

^{13}C NMR (75 MHz, $CDCl_3$)

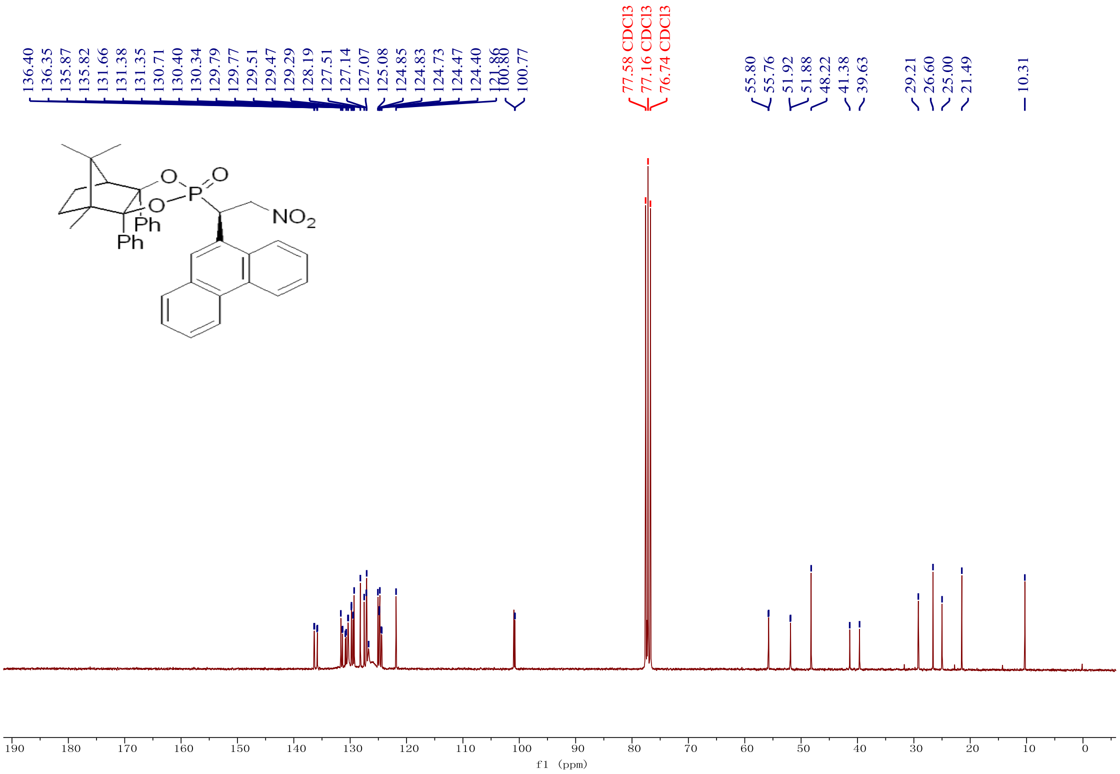

Fig. S291 ^{13}C NMR of compound **9r**

^{31}P NMR (121 MHz, $CDCl_3$)

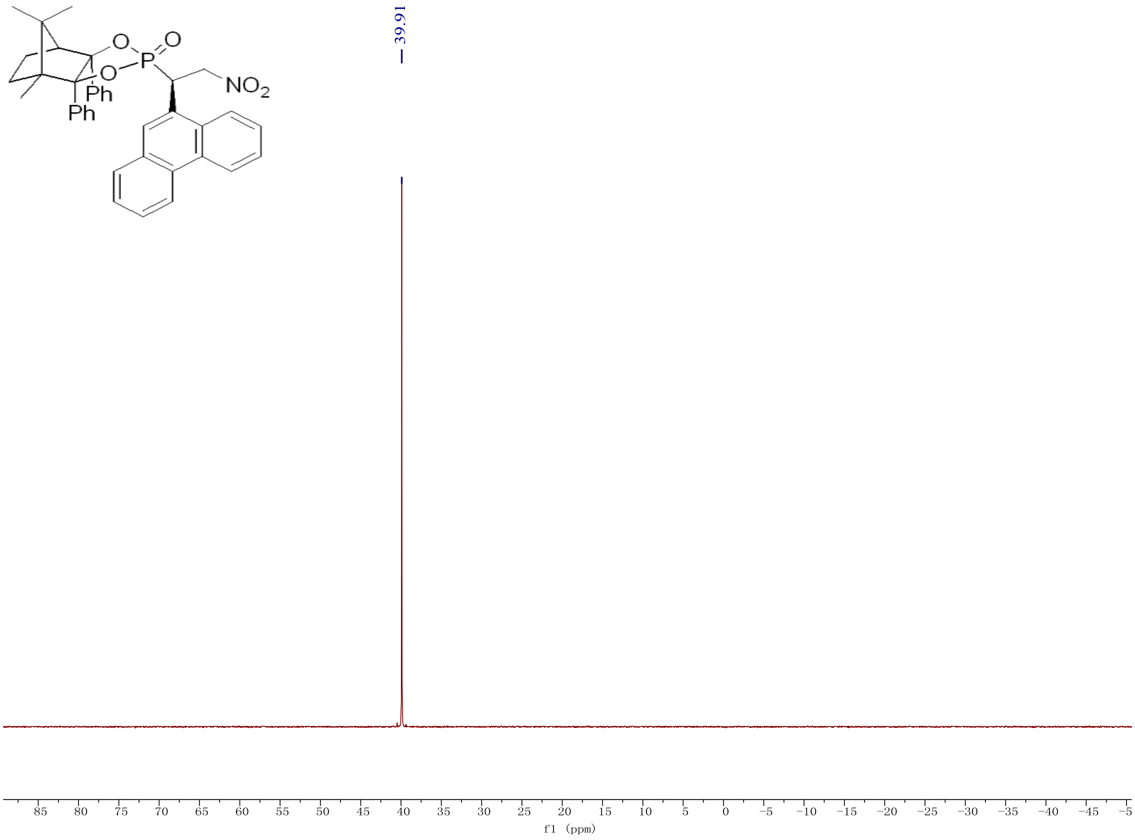

Fig. S292 ^{31}P NMR of compound **9r**

^1H NMR (300 MHz, $CDCl_3$)

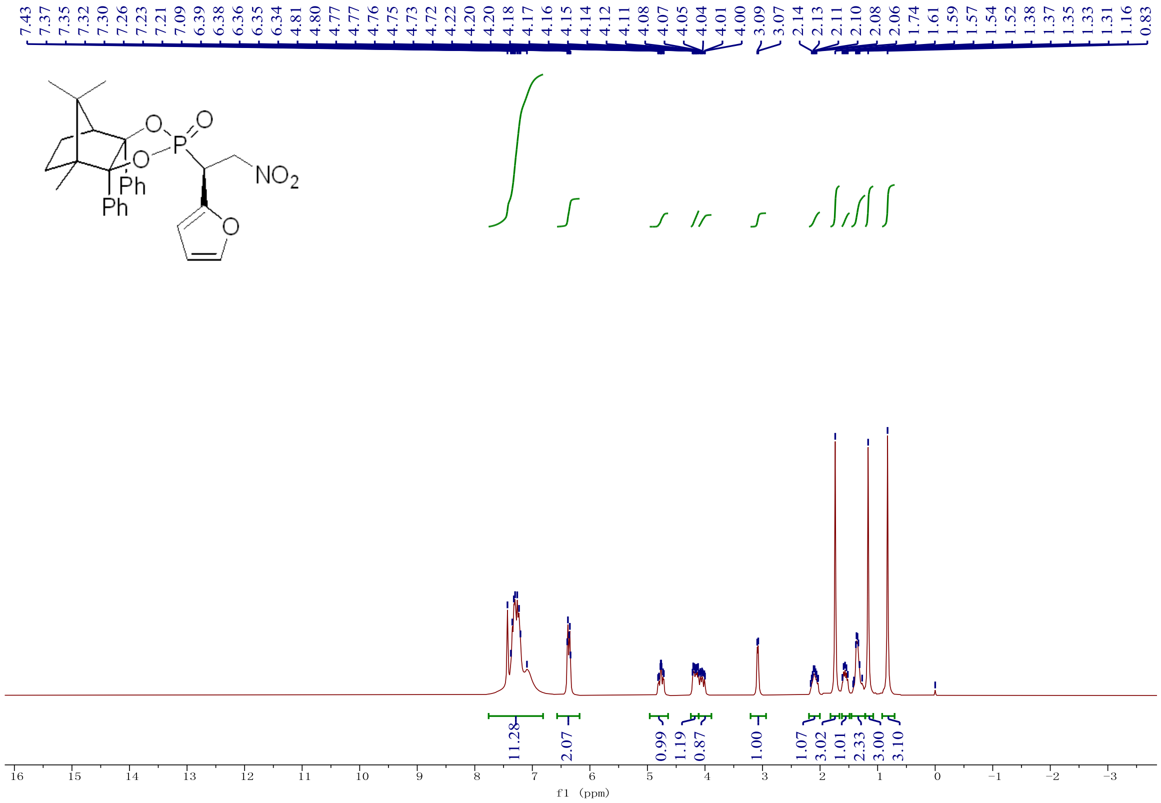

Fig. S293 ^1H NMR of compound **9t**

^{13}C NMR (75 MHz, $CDCl_3$)

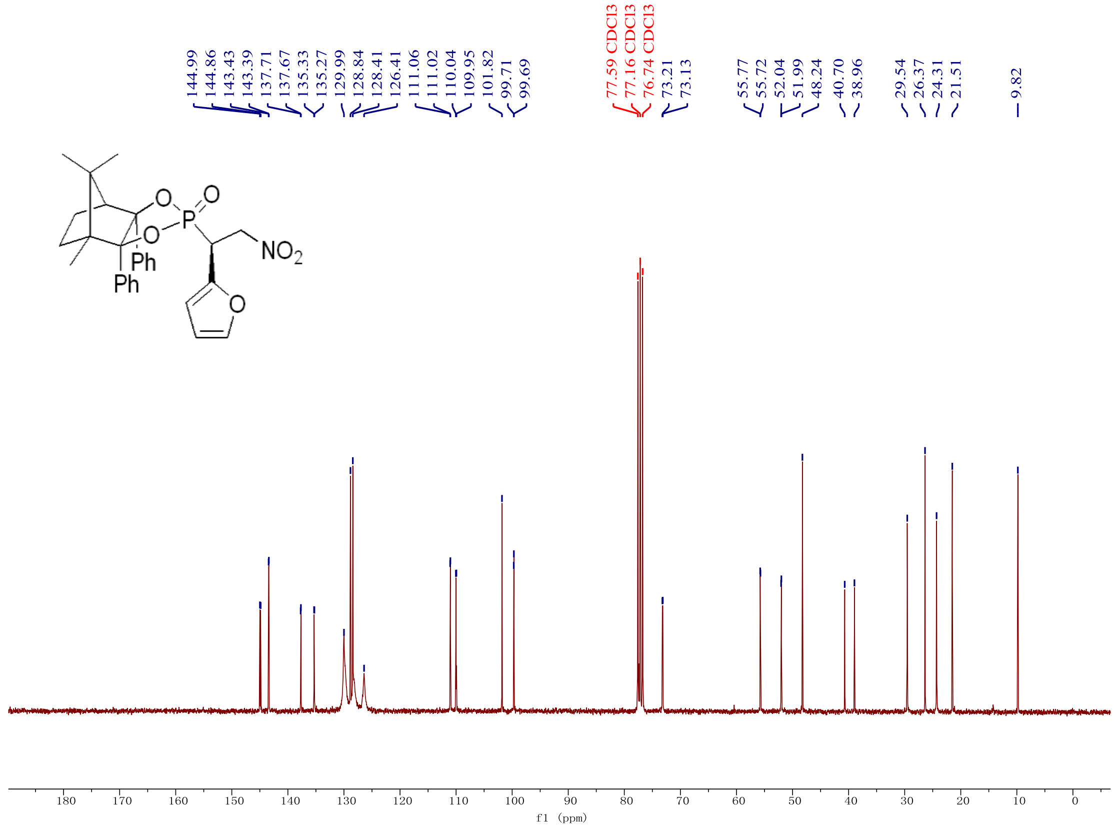

Fig. S294 ^{13}C NMR of compound **9t**

^{31}P NMR (121 MHz, $CDCl_3$)

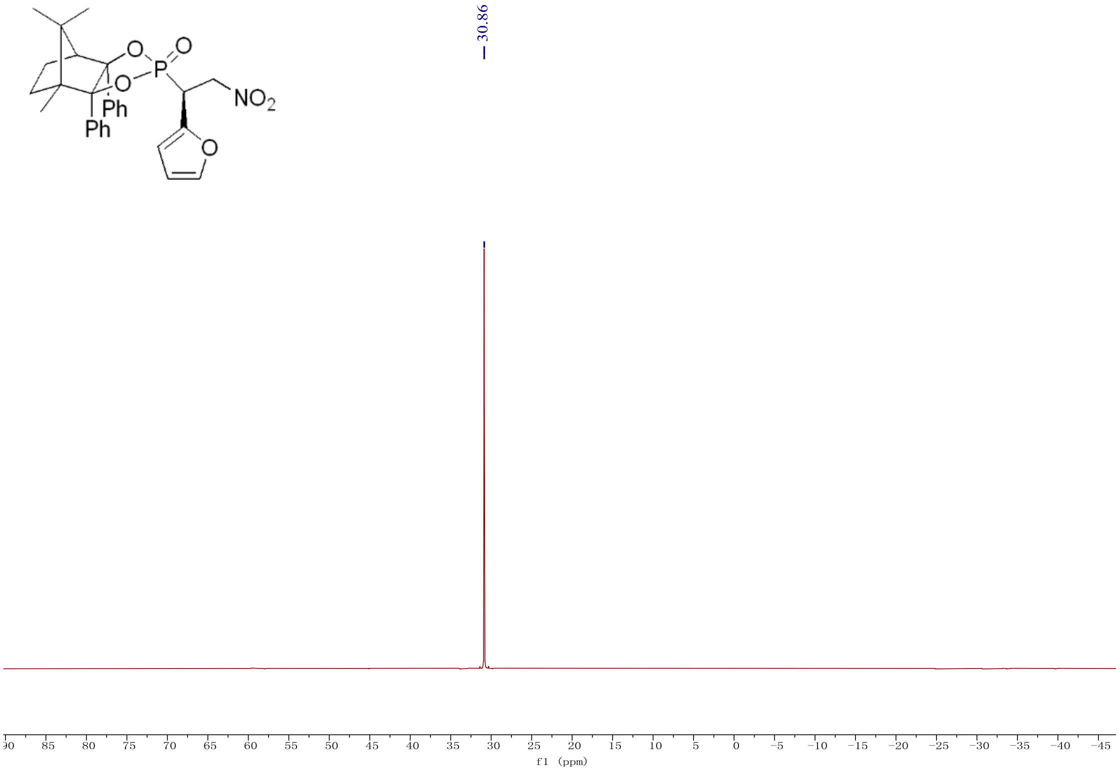

Fig. S295 ^{31}P NMR of compound **9t**

^{1}H NMR (300 MHz, $CDCl_3$)

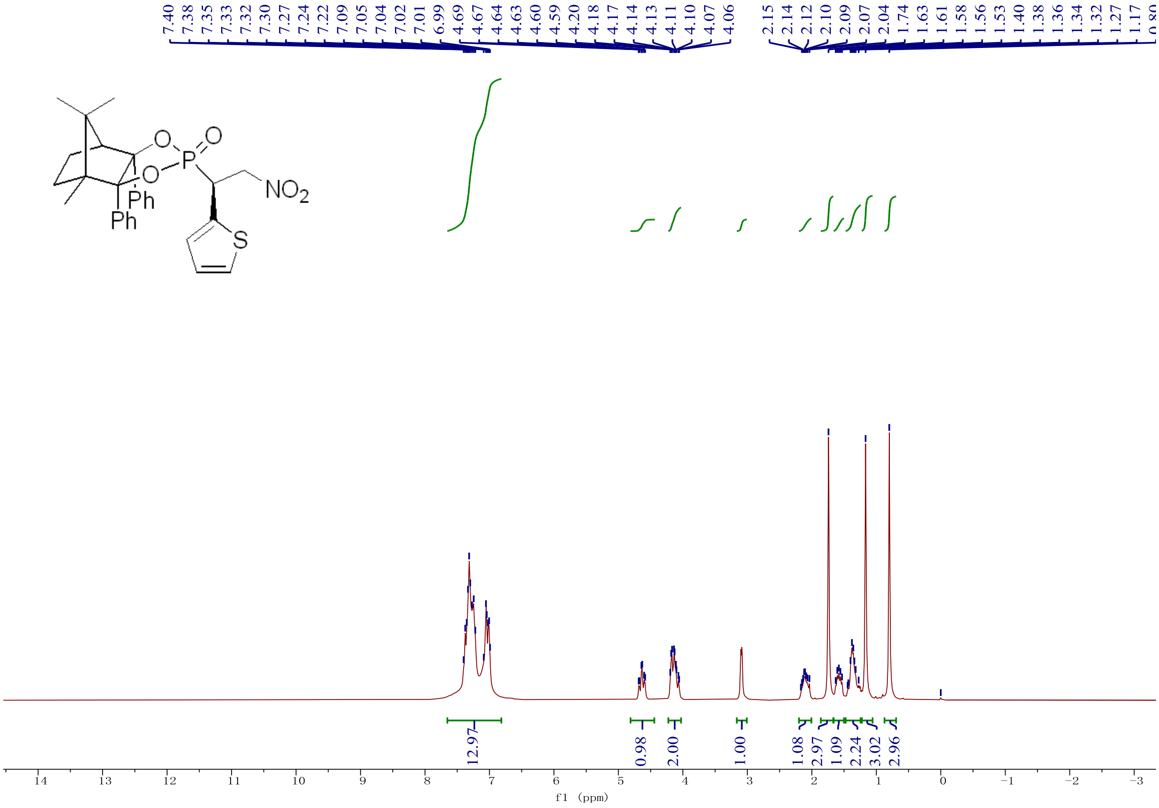

Fig. S296 ^{1}H NMR of compound **9u**

^{13}C NMR (75 MHz, $CDCl_3$)

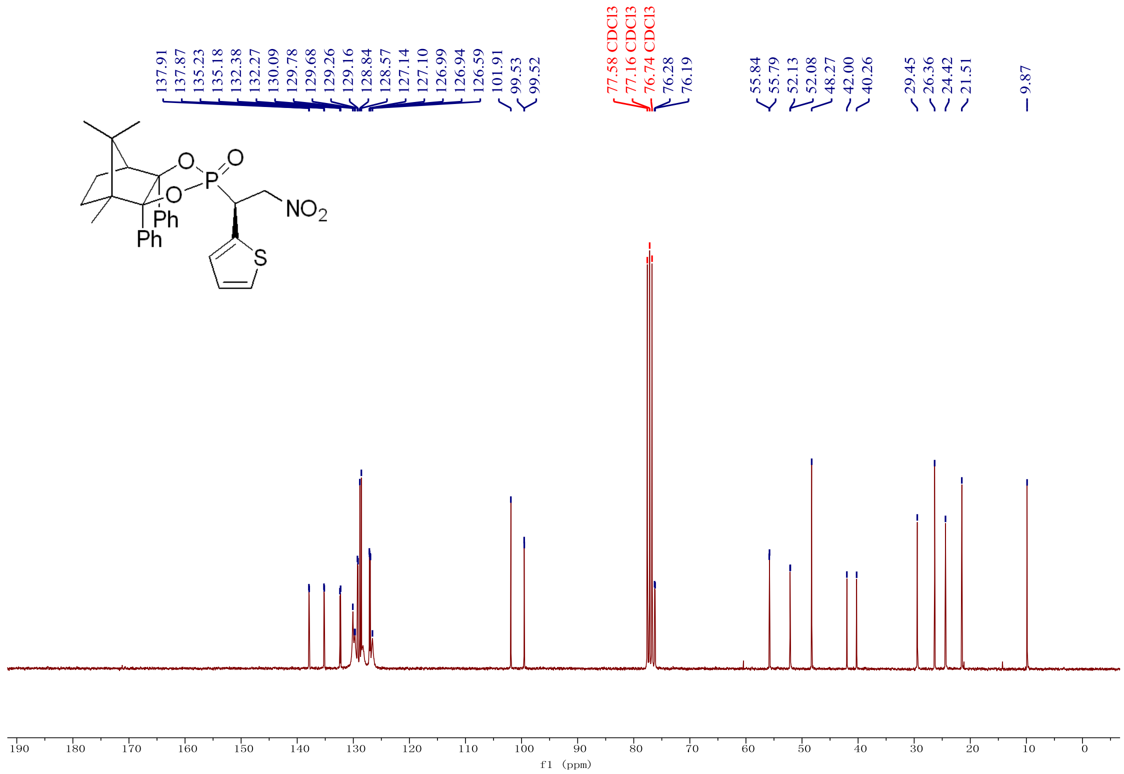

Fig. S297 ^{13}C NMR of compound **9u**

^{31}P NMR (121 MHz, $CDCl_3$)

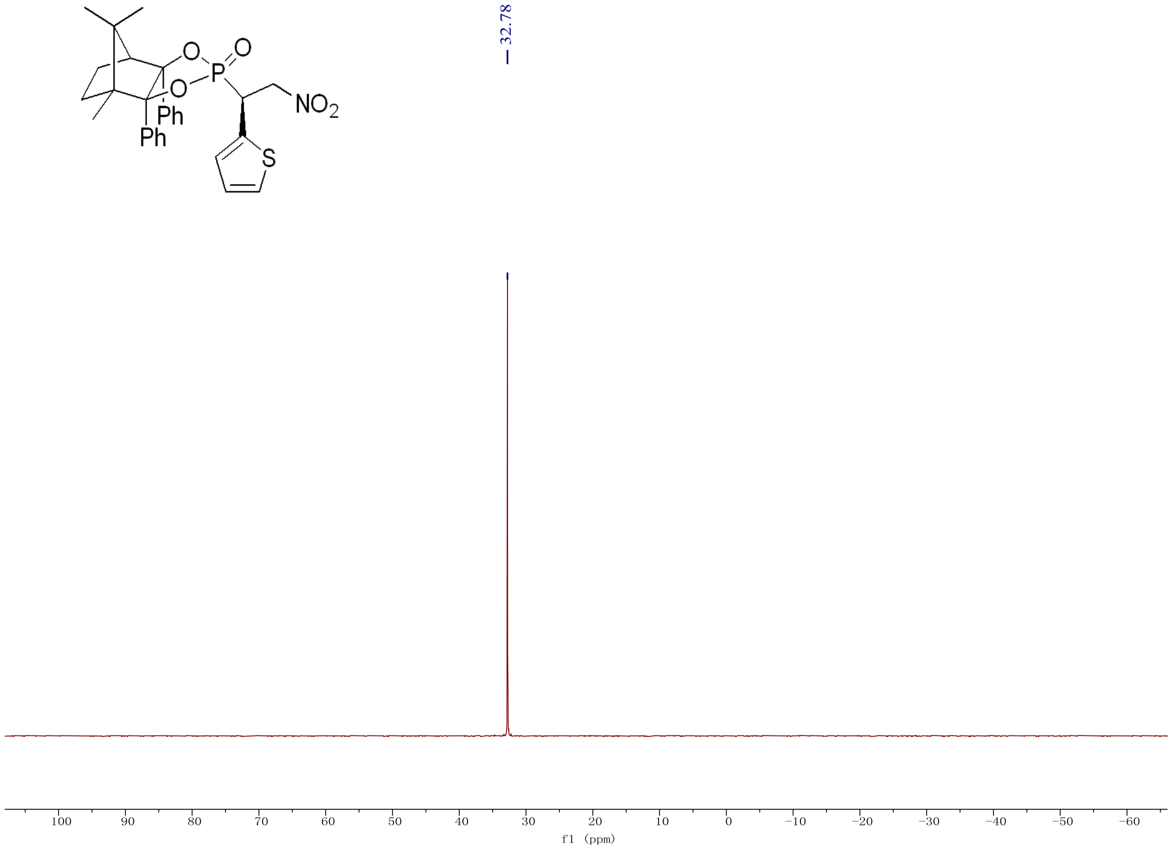

Fig. S298 ^{31}P NMR of compound **9u**

^{1}H NMR (300 MHz, $CDCl_3$)

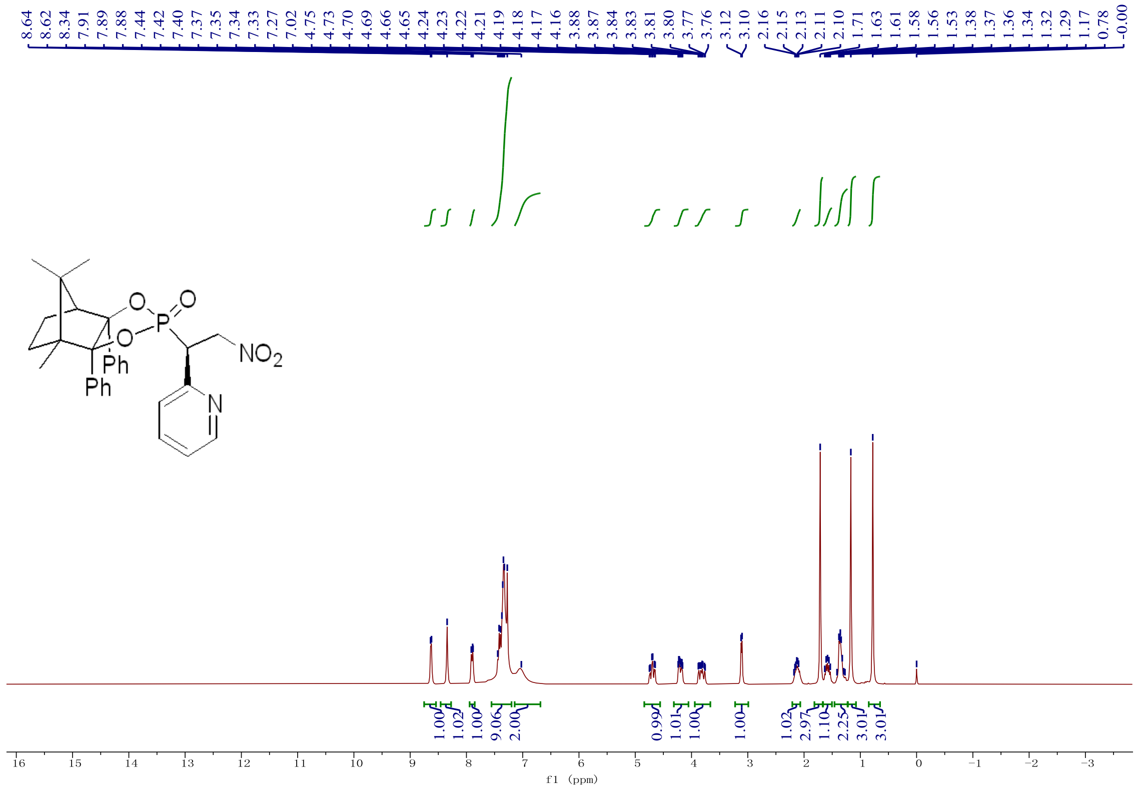

Fig. S299 ^{1}H NMR of compound **9v**

^{13}C NMR (75 MHz, $CDCl_3$)

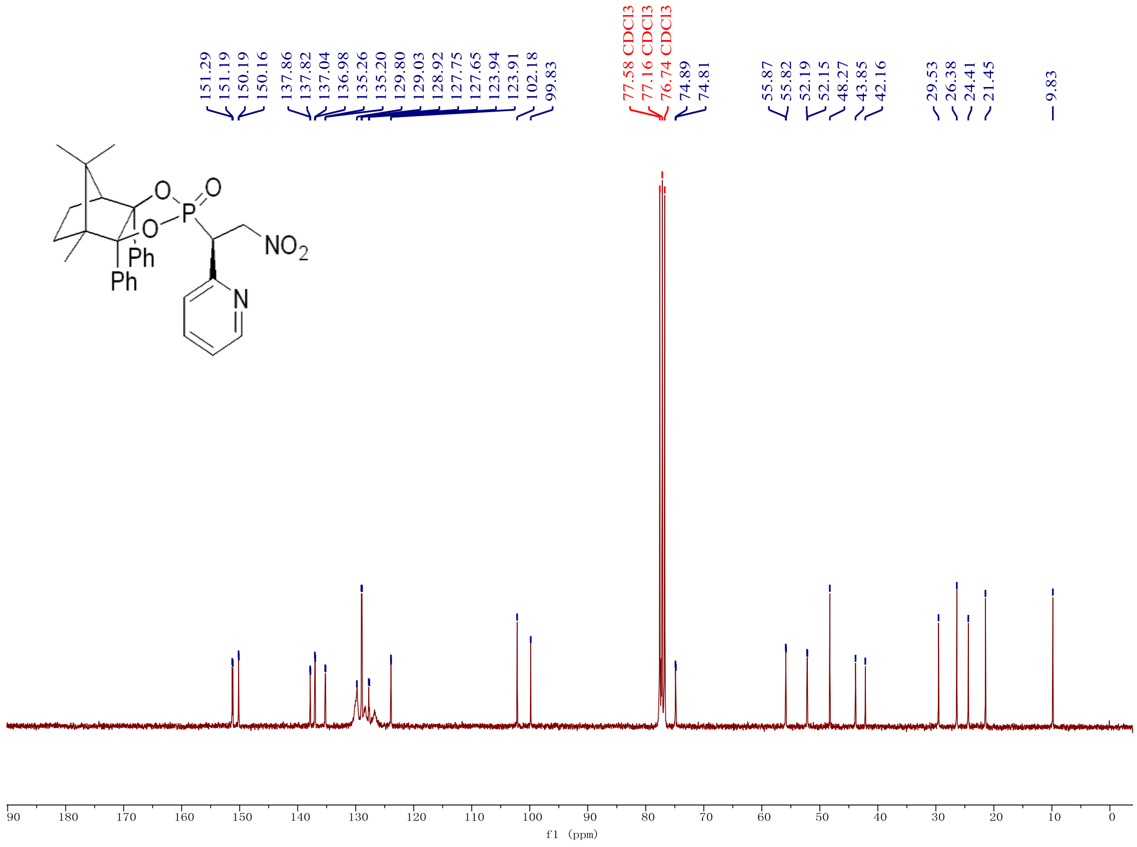

Fig. S300 ^{13}C NMR of compound **9v**

^{31}P NMR (121 MHz, $CDCl_3$)

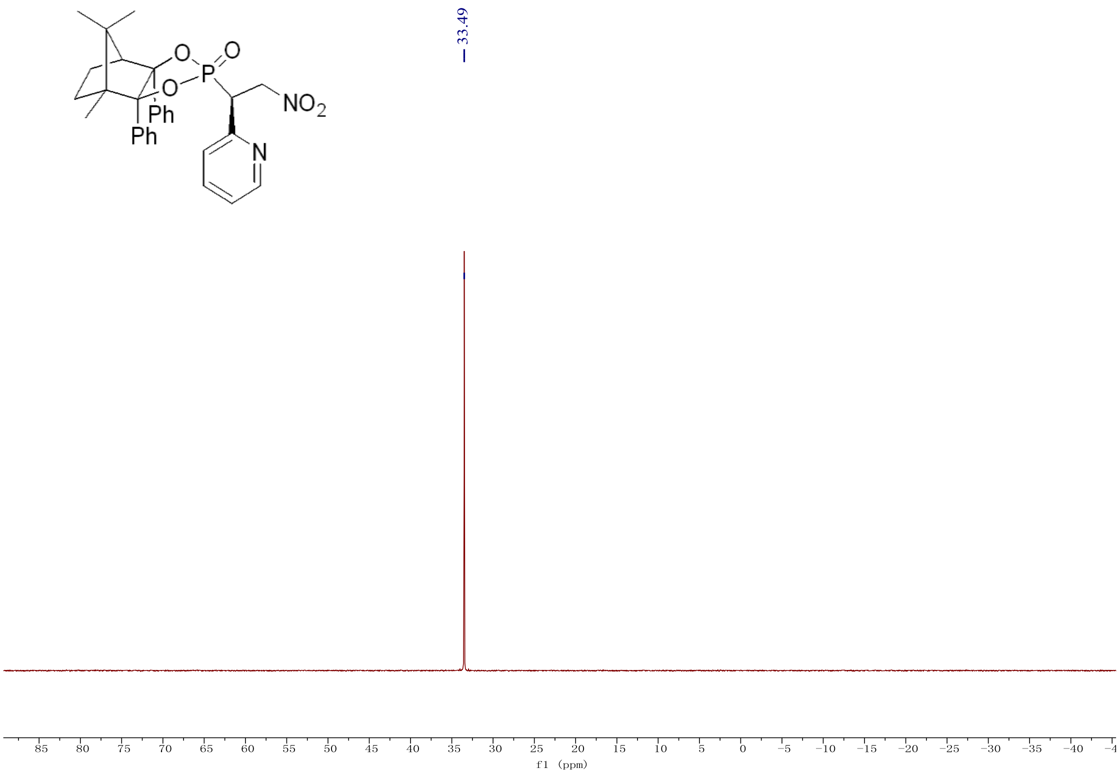

Fig. S301 ^{31}P NMR of compound **9v**

^1H NMR (300 MHz, $CDCl_3$)

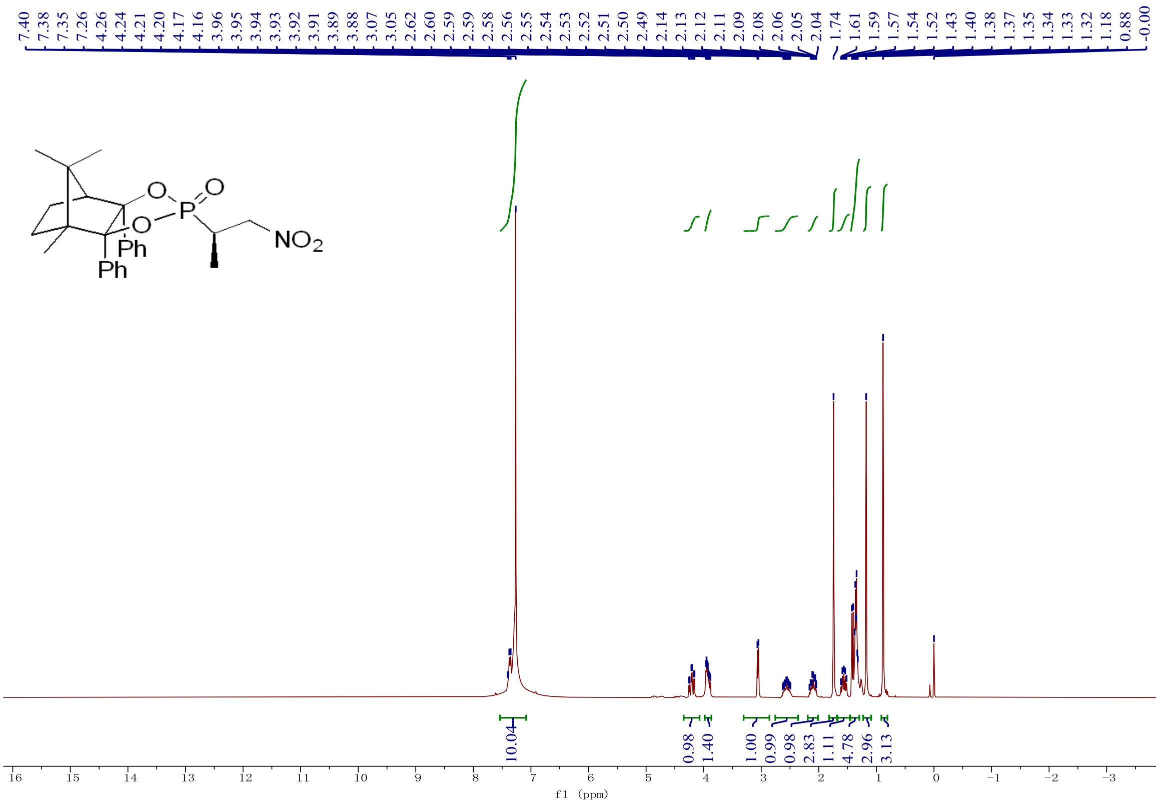

Fig. S302 ^1H NMR of compound **9wa**

^{13}C NMR (75 MHz, $CDCl_3$)

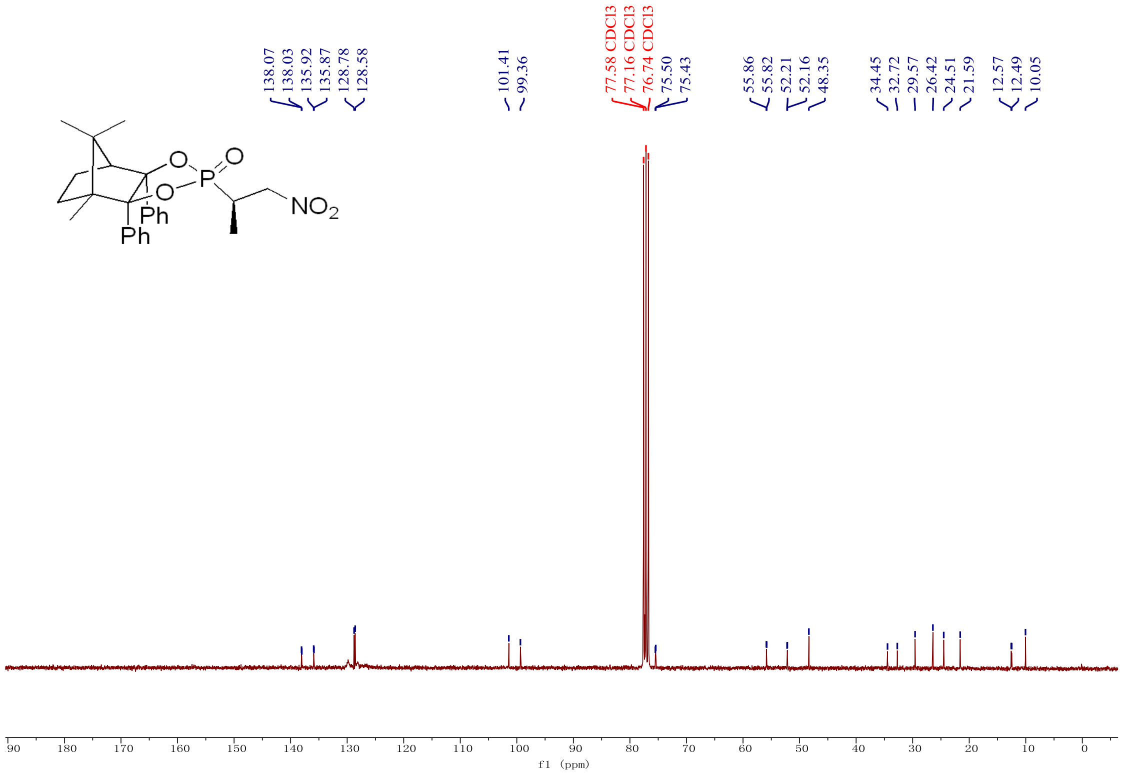

Fig. S303 ^{13}C NMR of compound **9wa**

^{31}P NMR (121 MHz, $CDCl_3$)

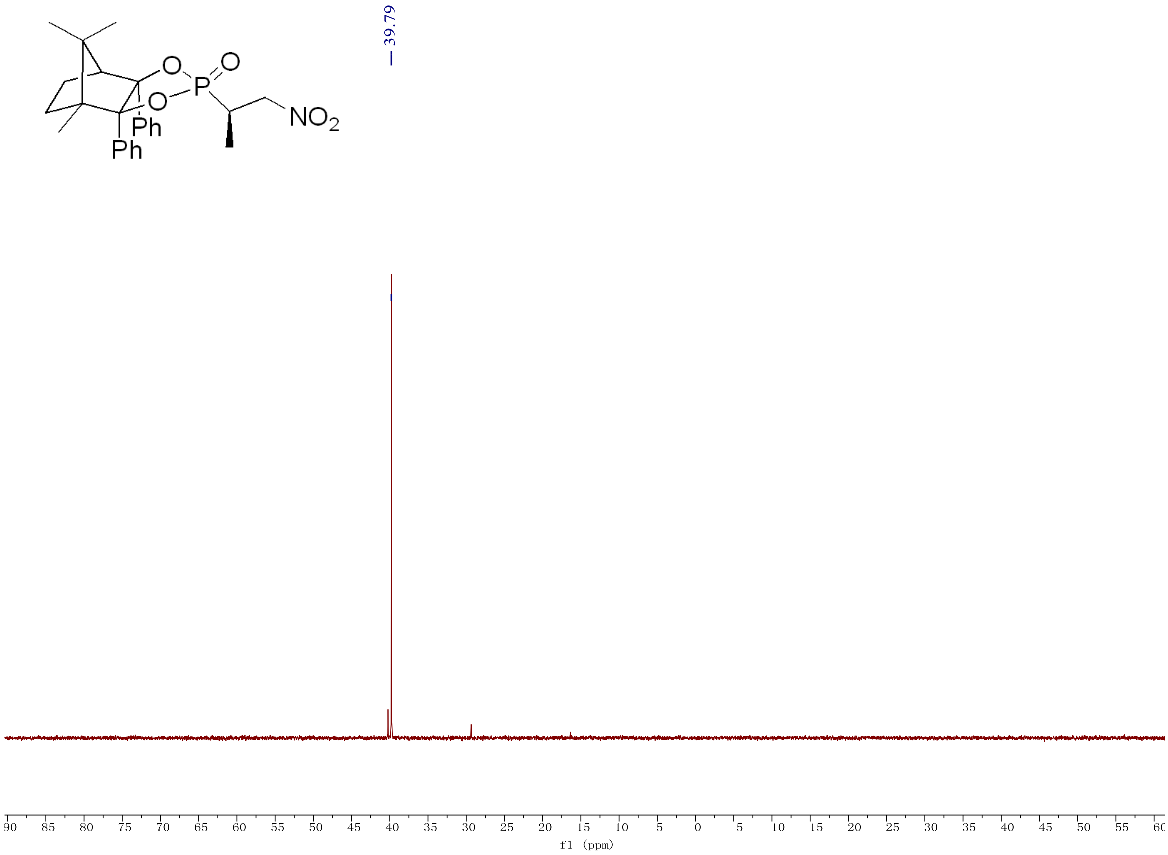

Fig. S304 ^{31}P NMR of compound **9wa**

^1H NMR (300 MHz, $CDCl_3$)

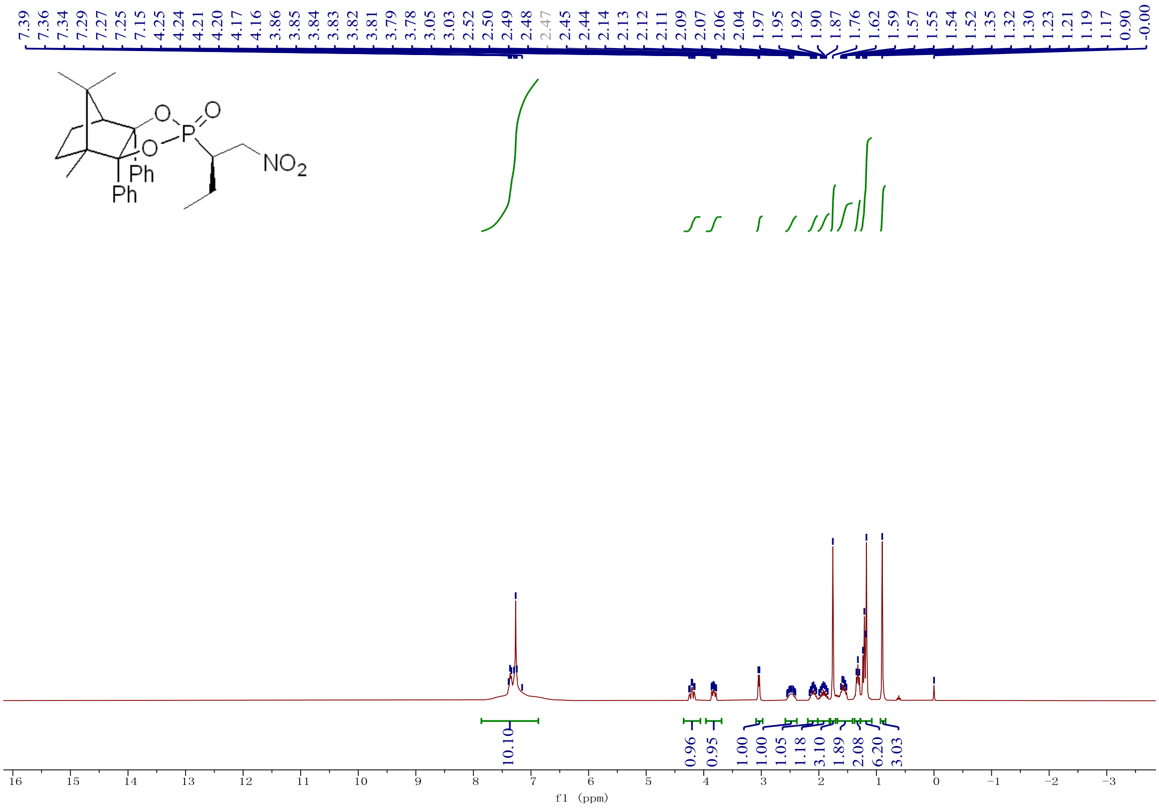

Fig. S305 ^1H NMR of compound **9wb**

^{13}C NMR (75 MHz, $CDCl_3$)

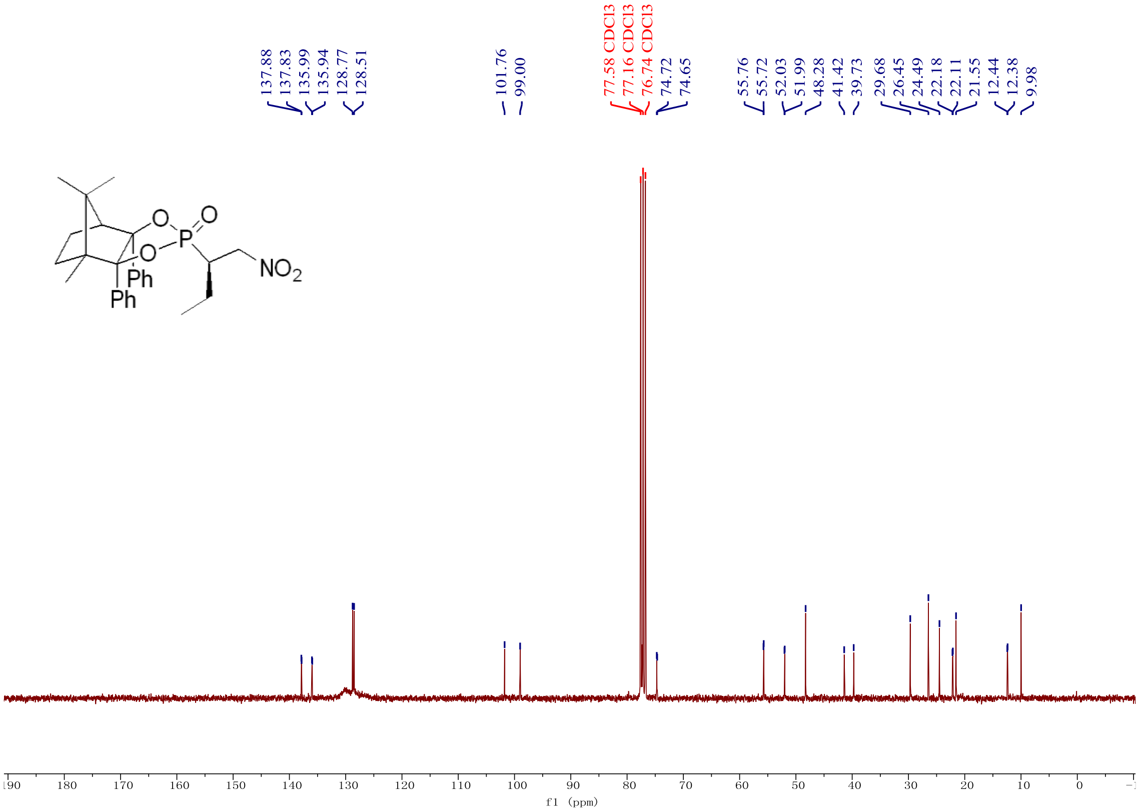

Fig. S306 ^{13}C NMR of compound **9wb**

^{31}P NMR (121 MHz, $CDCl_3$)

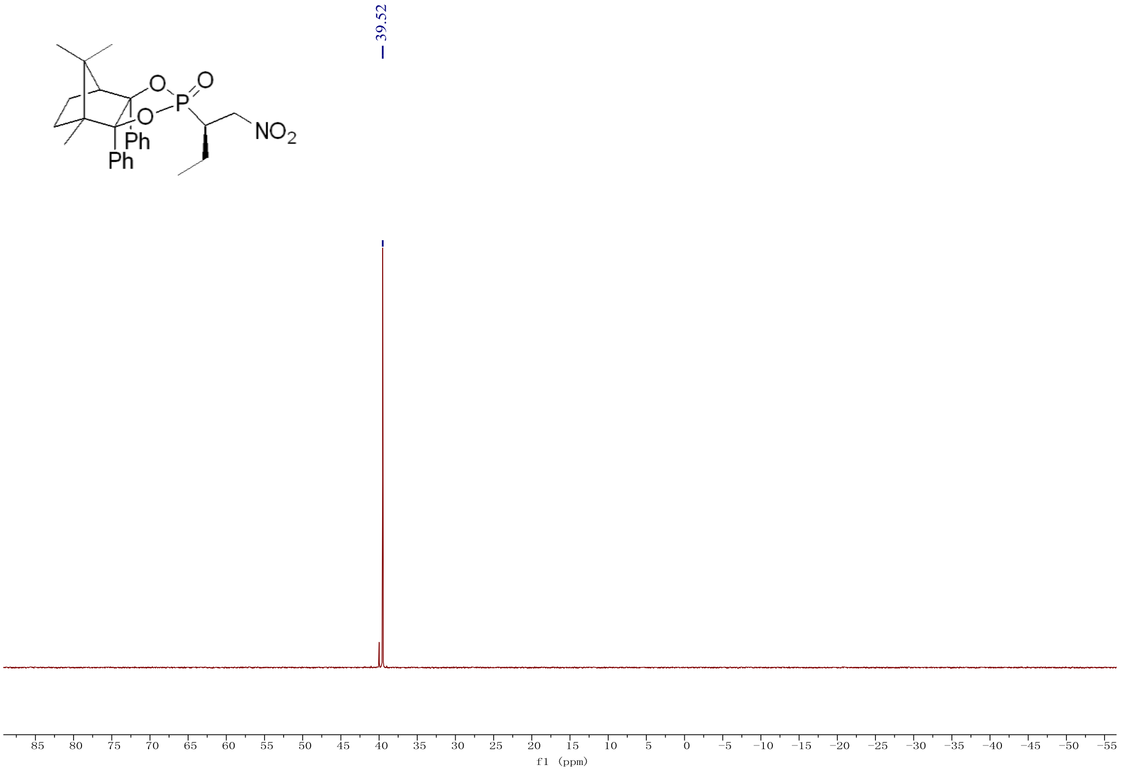

Fig. S307 ^{31}P NMR of compound **9wb**

^{1}H NMR (300 MHz, $CDCl_3$)

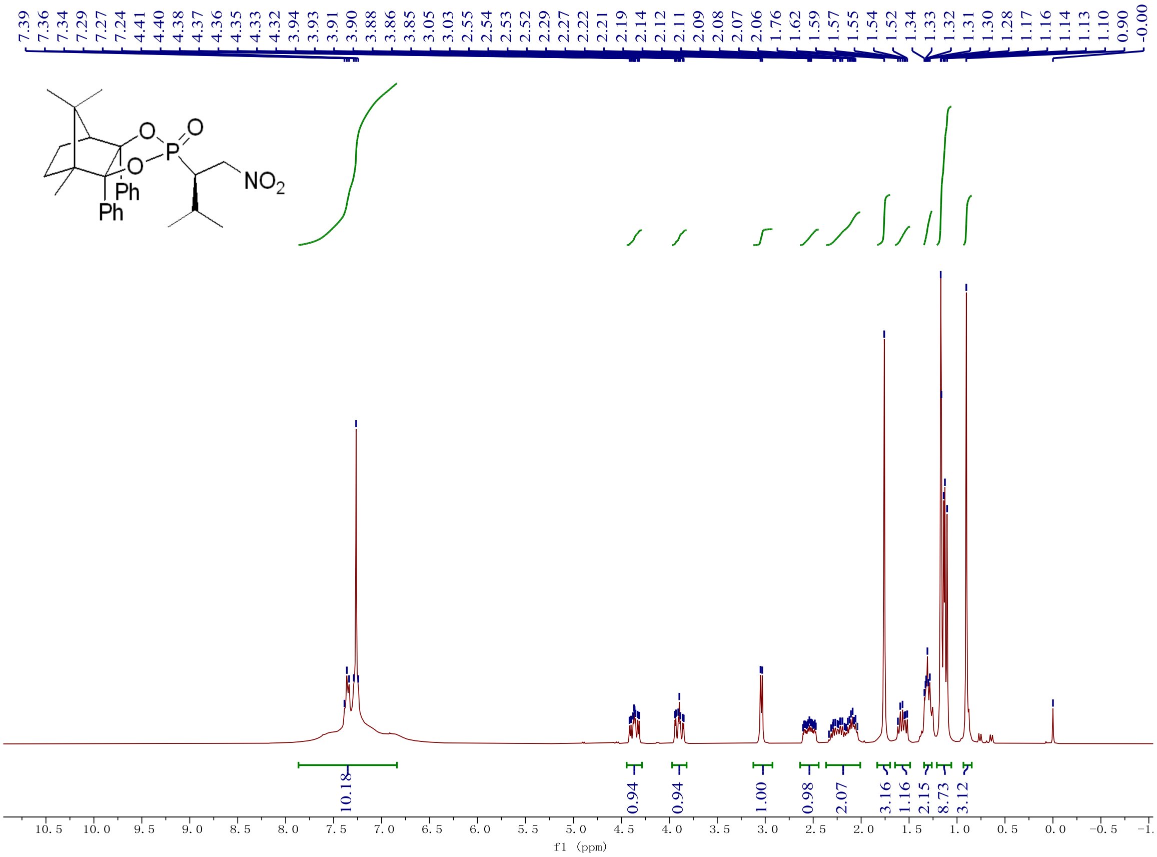

Fig. S308 ^{1}H NMR of compound **9wc**

^{13}C NMR (75 MHz, $CDCl_3$)

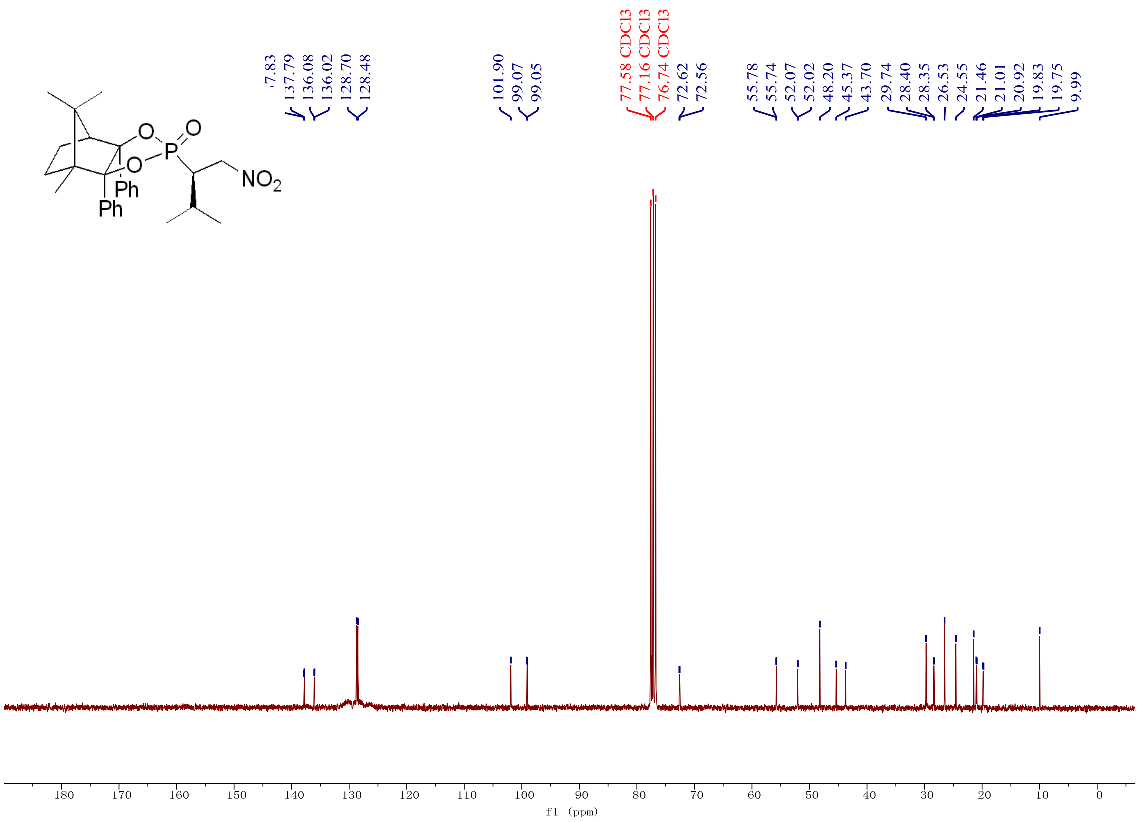

Fig. S309 ^{13}C NMR of compound **9wc**

^{31}P NMR (121 MHz, $CDCl_3$)

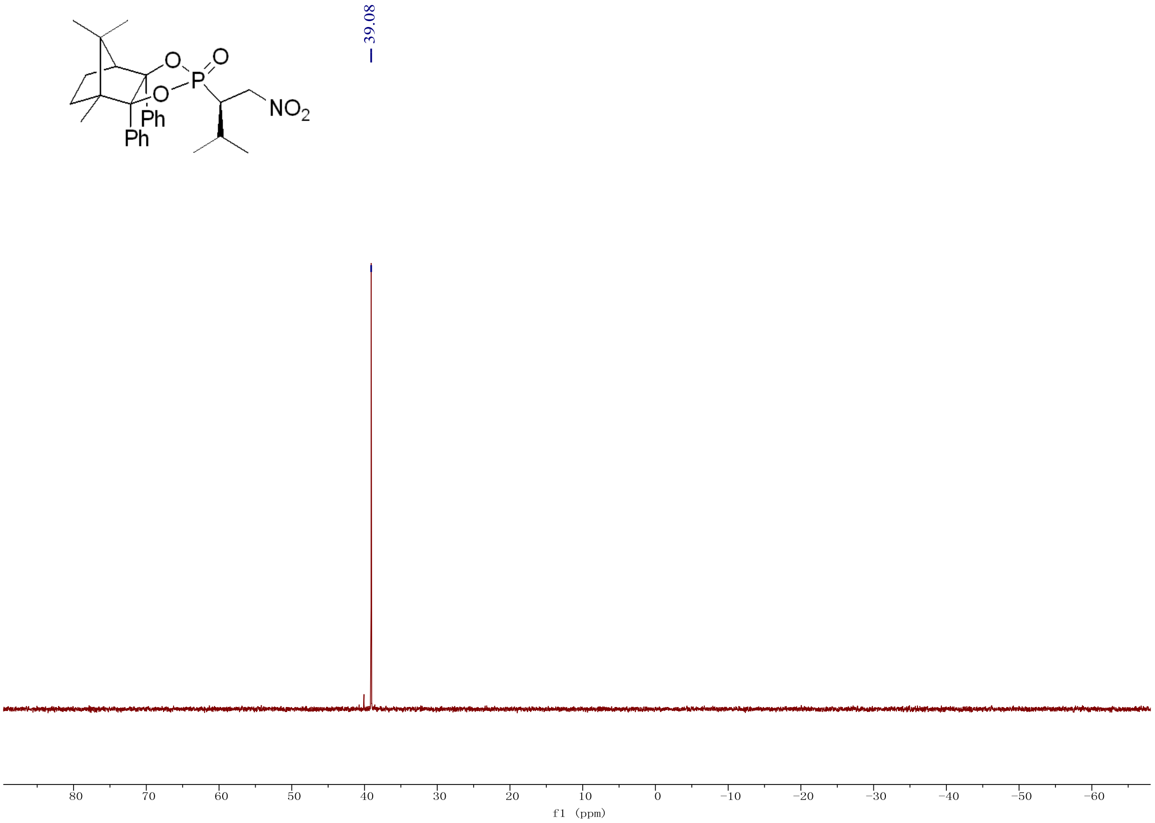

Fig. S310 ^{31}P NMR of compound **9wc**

^{1}H NMR (300 MHz, $CDCl_3$)

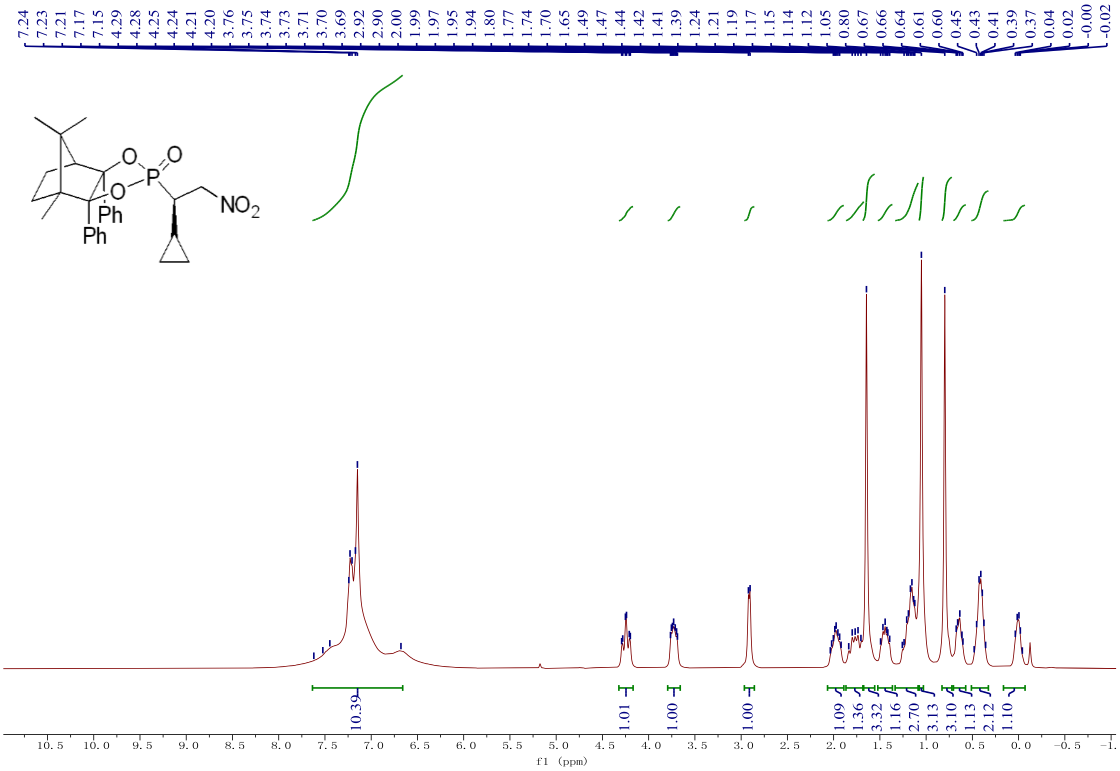

Fig. S311 ^{1}H NMR of compound **9xa**

^{13}C NMR (75 MHz, $CDCl_3$)

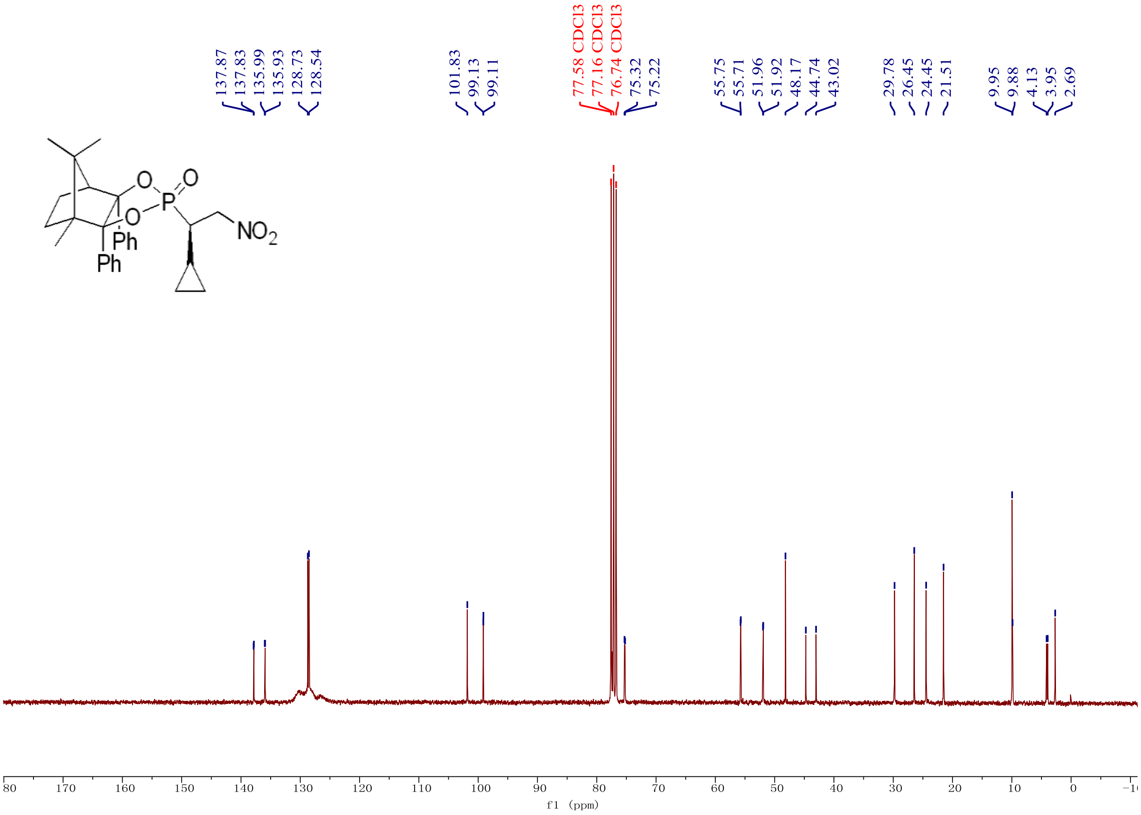

Fig. S312 ^{13}C NMR of compound **9xa**

^{31}P NMR (121 MHz, $CDCl_3$)

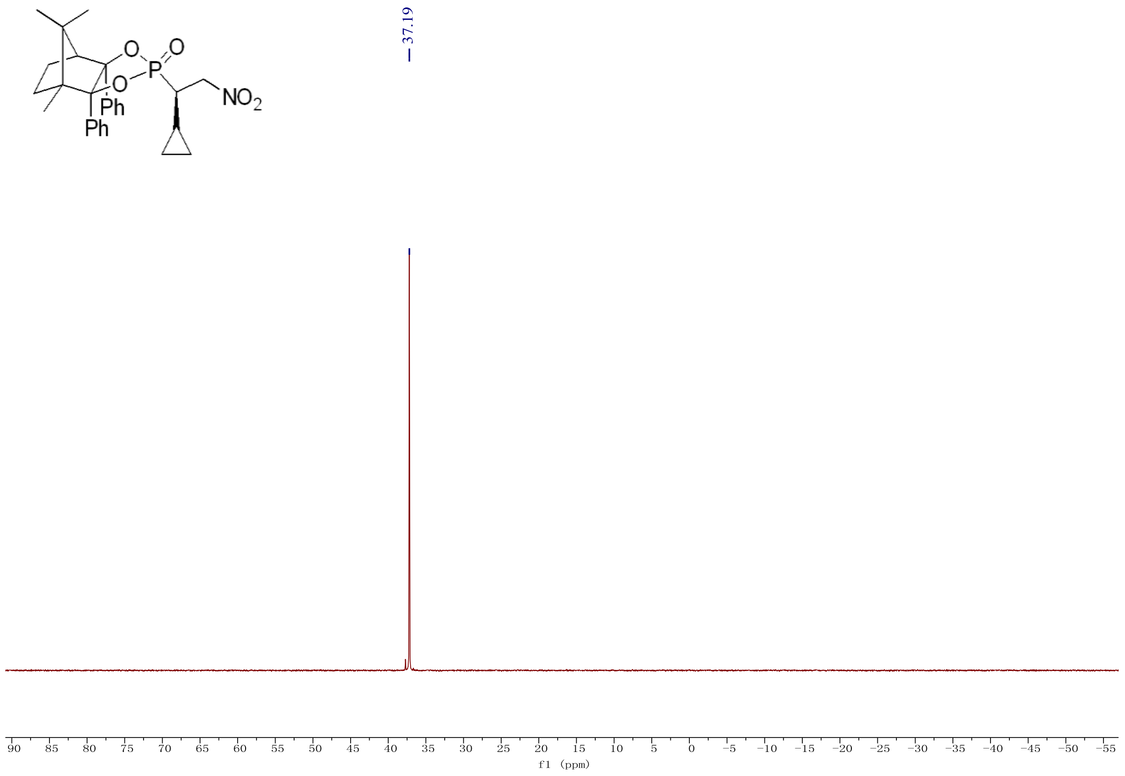

Fig. S313 ^{31}P NMR of compound **9xa**

^{1}H NMR (300 MHz, $CDCl_3$)

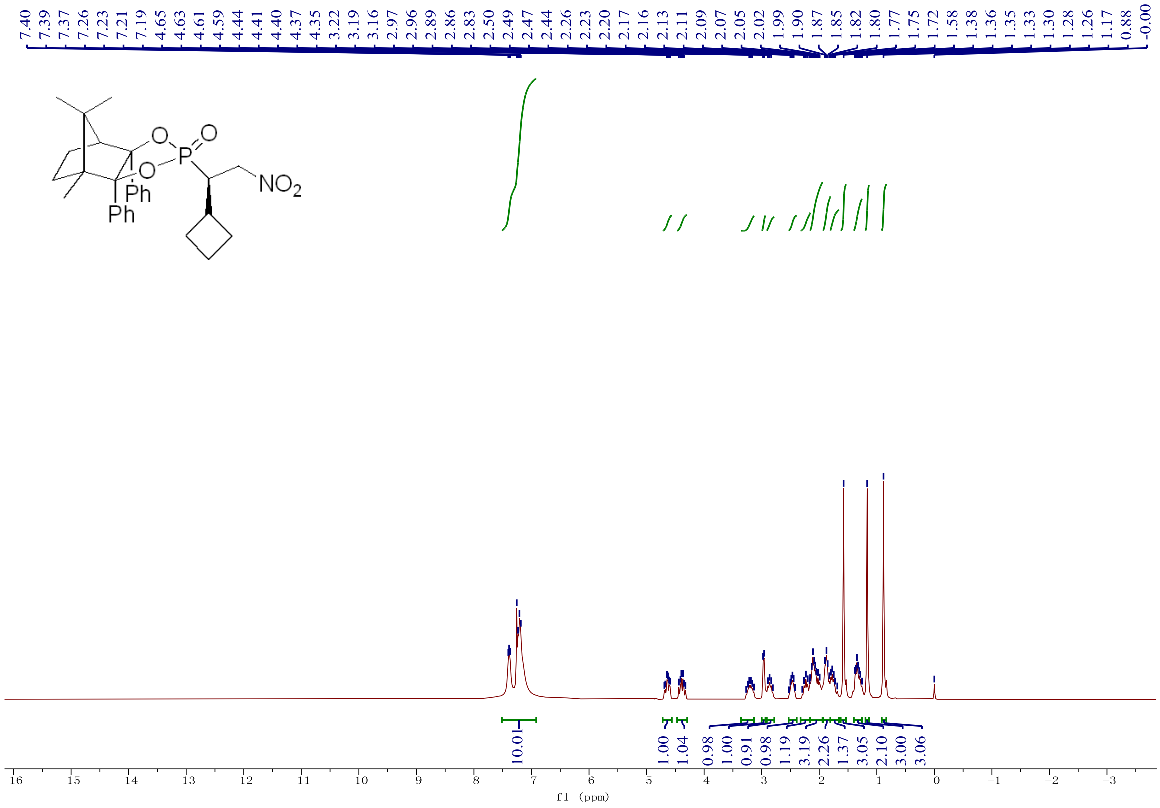

Fig. S314 ^{1}H NMR of compound **9xb**

^{13}C NMR (75 MHz, $CDCl_3$)

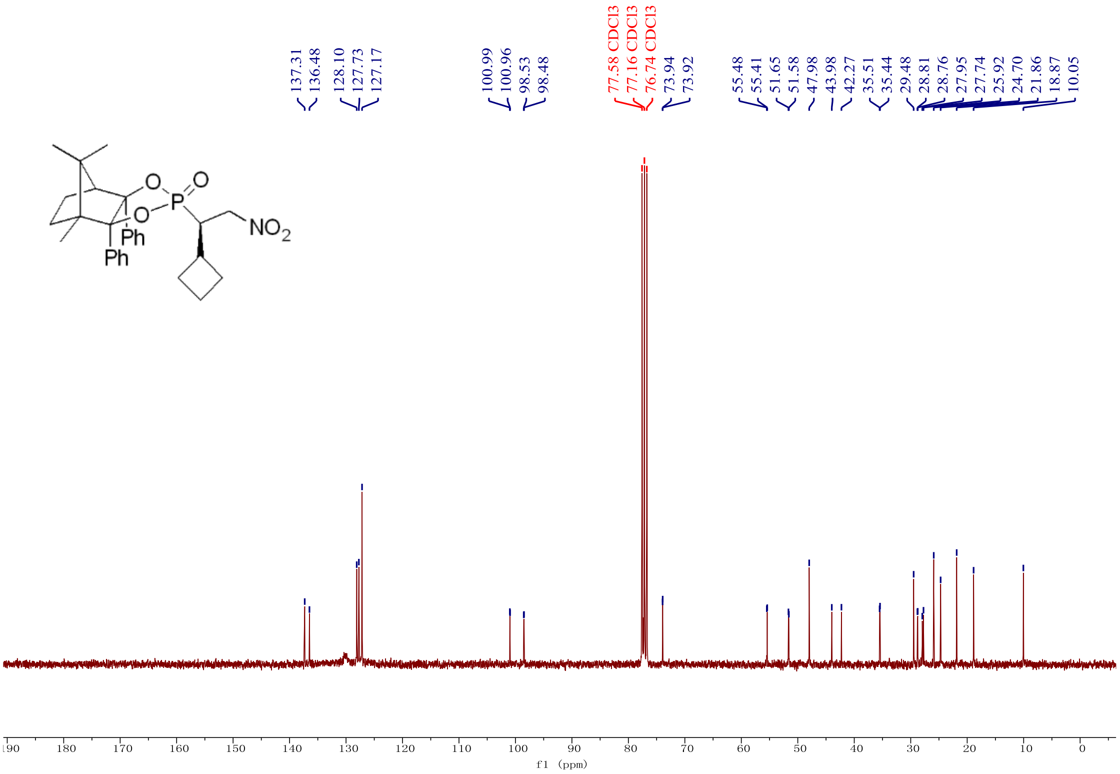

Fig. S315 ^{13}C NMR of compound **9xb**

^{31}P NMR (121 MHz, $CDCl_3$)

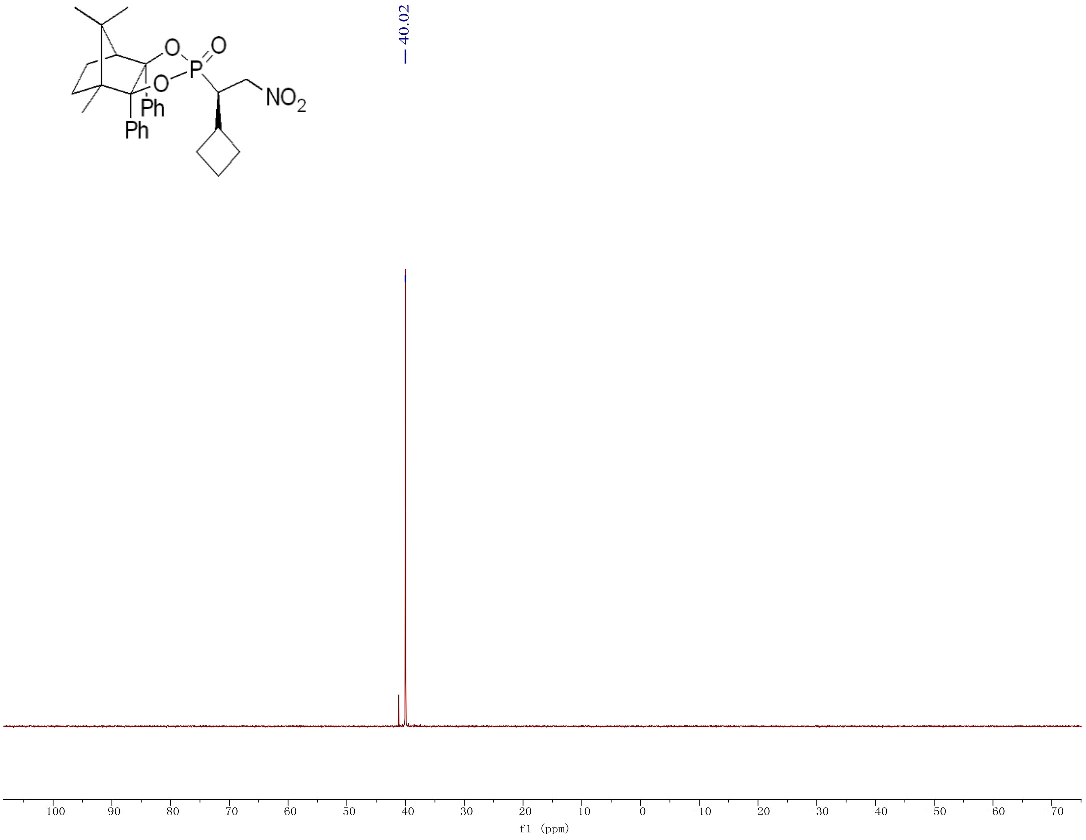

Fig. S316 ^{31}P NMR of compound **9xb**

^{1}H NMR (300 MHz, $CDCl_3$)

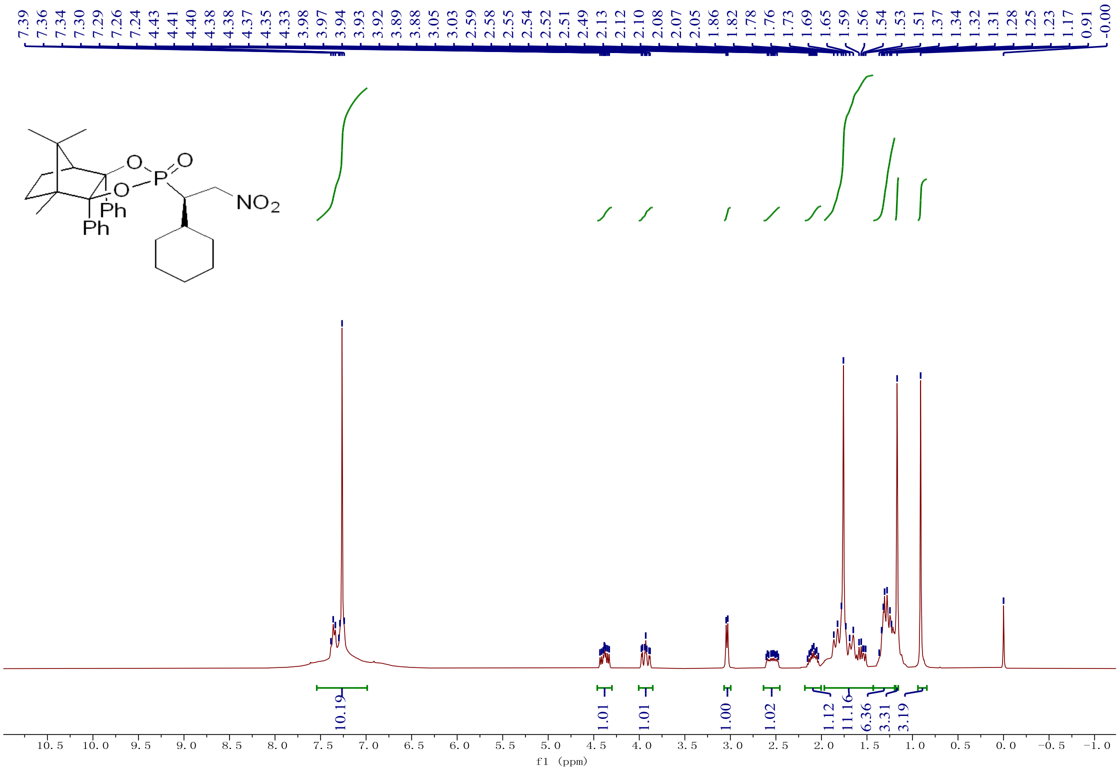

Fig. S317 ^{1}H NMR of compound **9xc**

^{13}C NMR (75 MHz, $CDCl_3$)

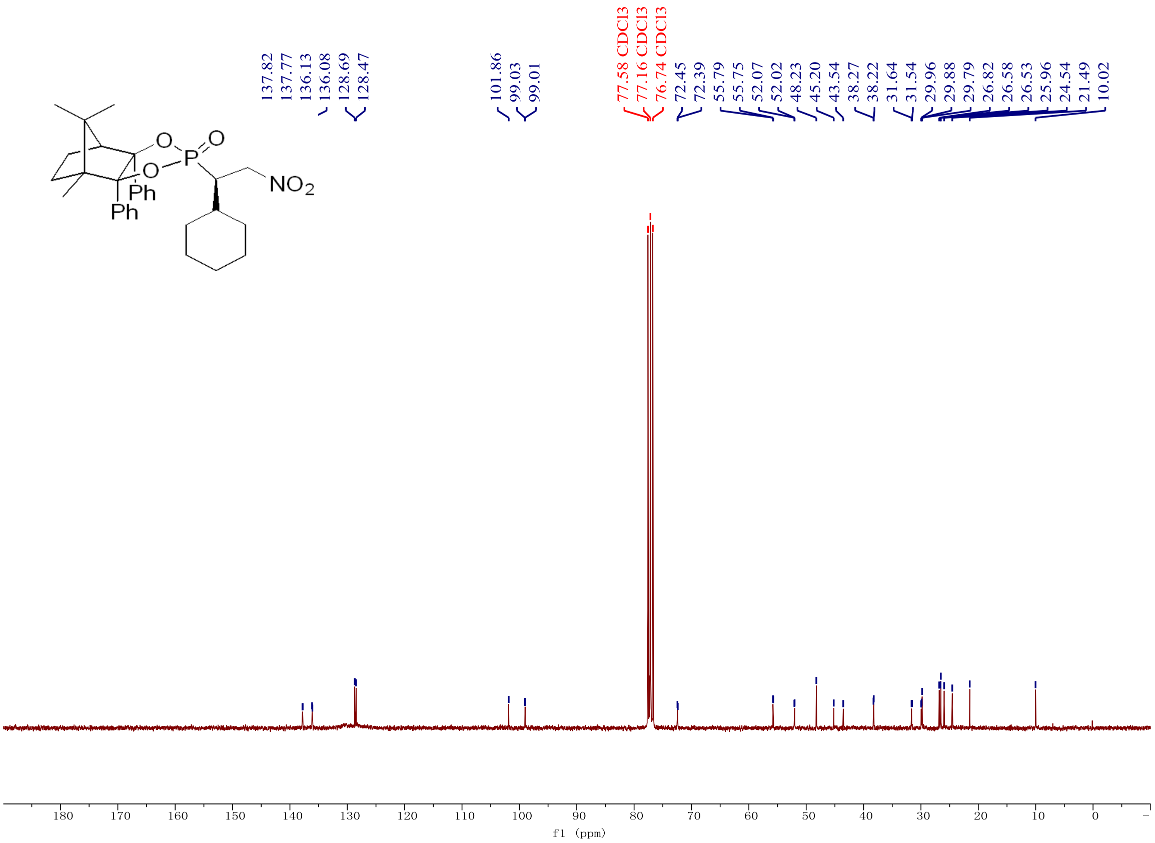

Fig. S318 ^{13}C NMR of compound **9xc**

^{31}P NMR (121 MHz, $CDCl_3$)

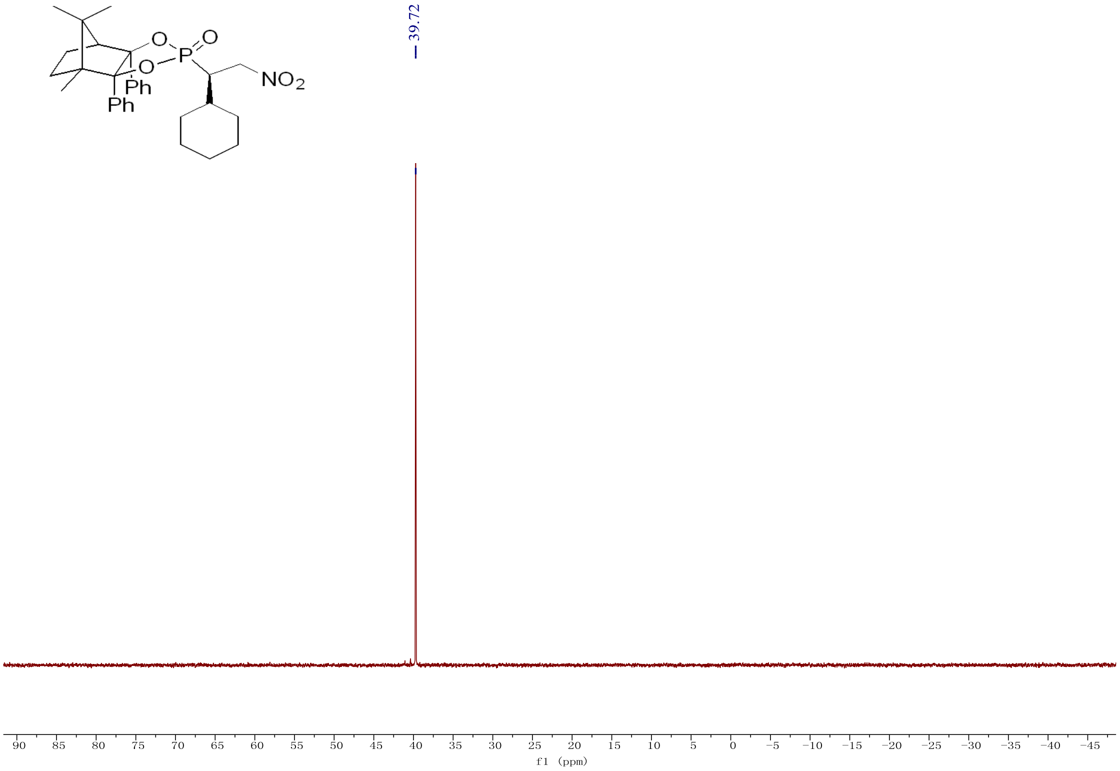

Fig. S319 ^{31}P NMR of compound **9xc**

^{1}H NMR (300 MHz, D_2O)

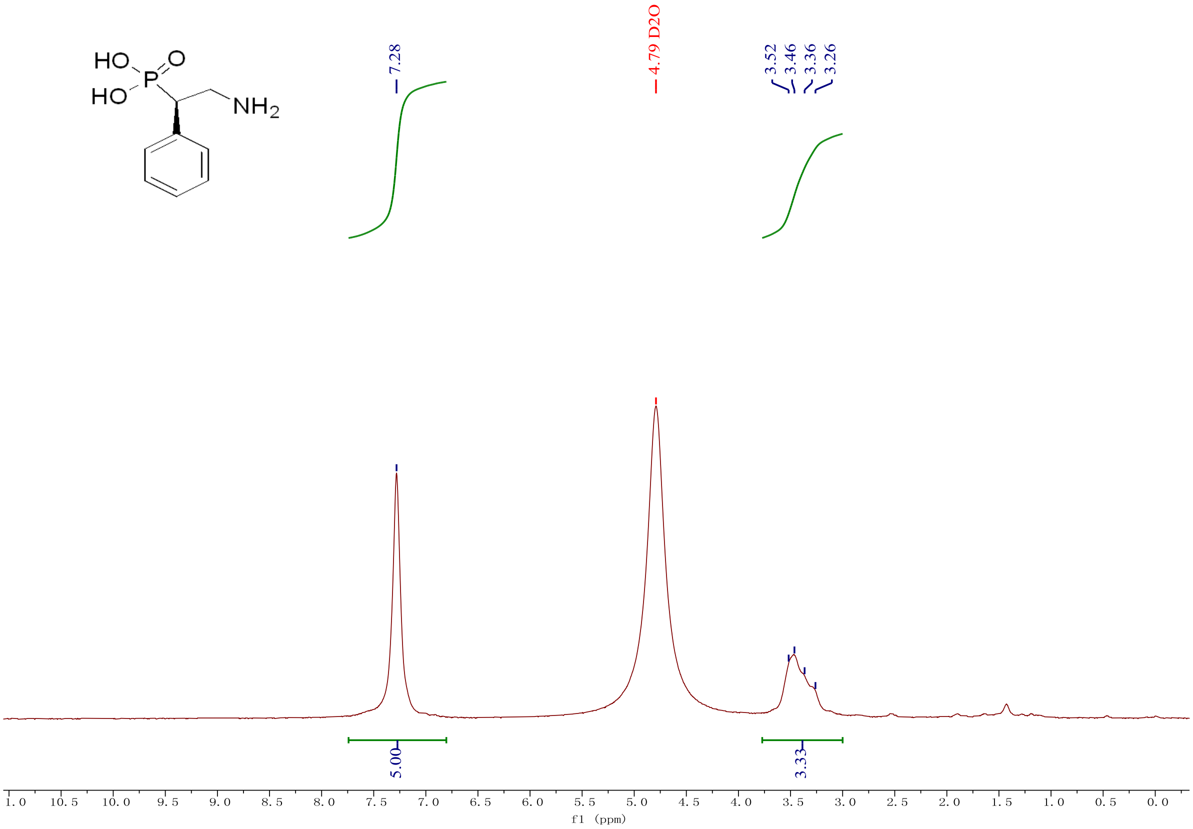

Fig. S320 ^{1}H NMR of compound **10a**

^{13}C NMR (75 MHz, D_2O)

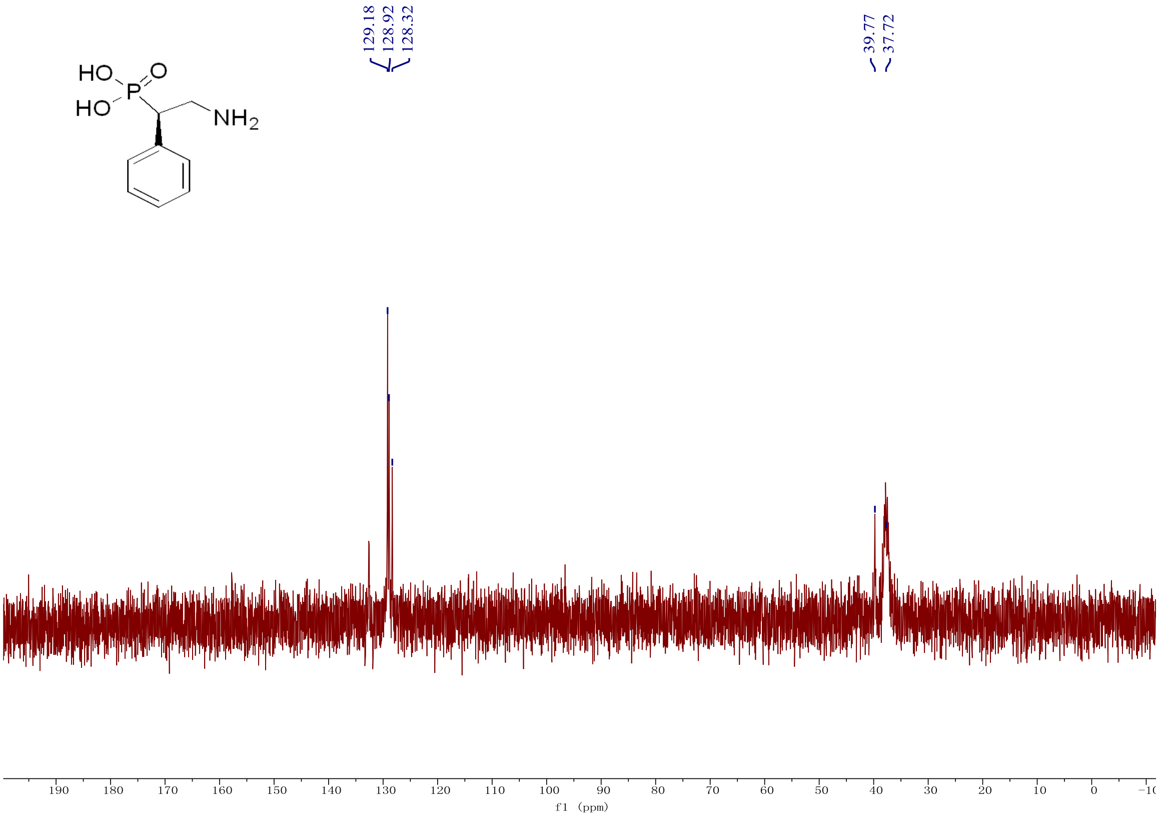

Fig. S321 ^{13}C NMR of compound **10a**

^{31}P NMR (121 MHz, D_2O)

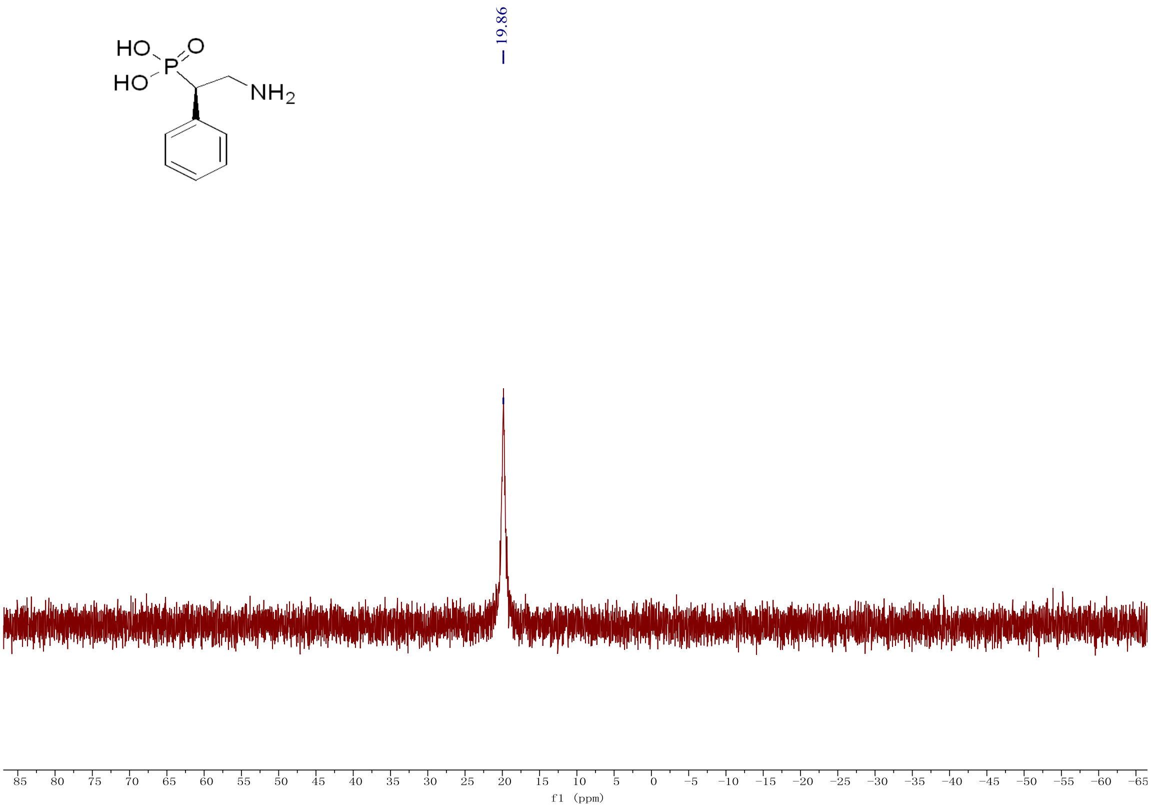

Fig. S322 ^{31}P NMR of compound **10a**

^{1}H NMR (300 MHz, $CDCl_3$)

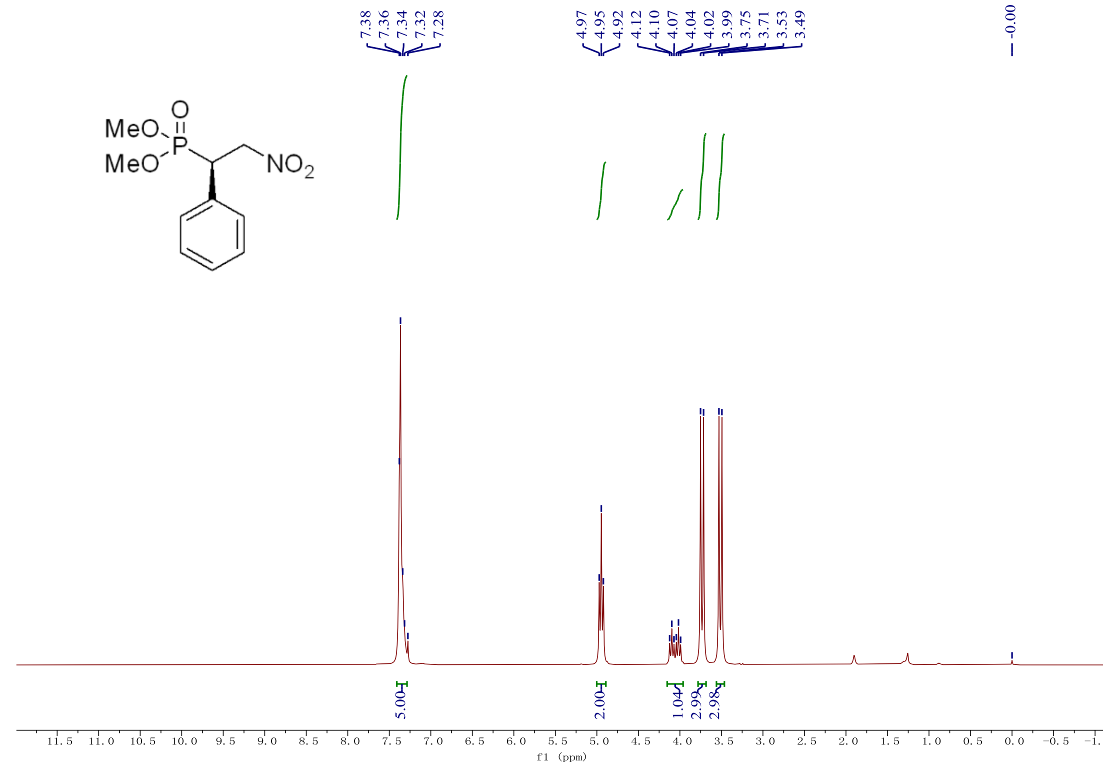

Fig. S323 ^{1}H NMR of compound **10aa**

^{13}C NMR (75 MHz, $CDCl_3$)

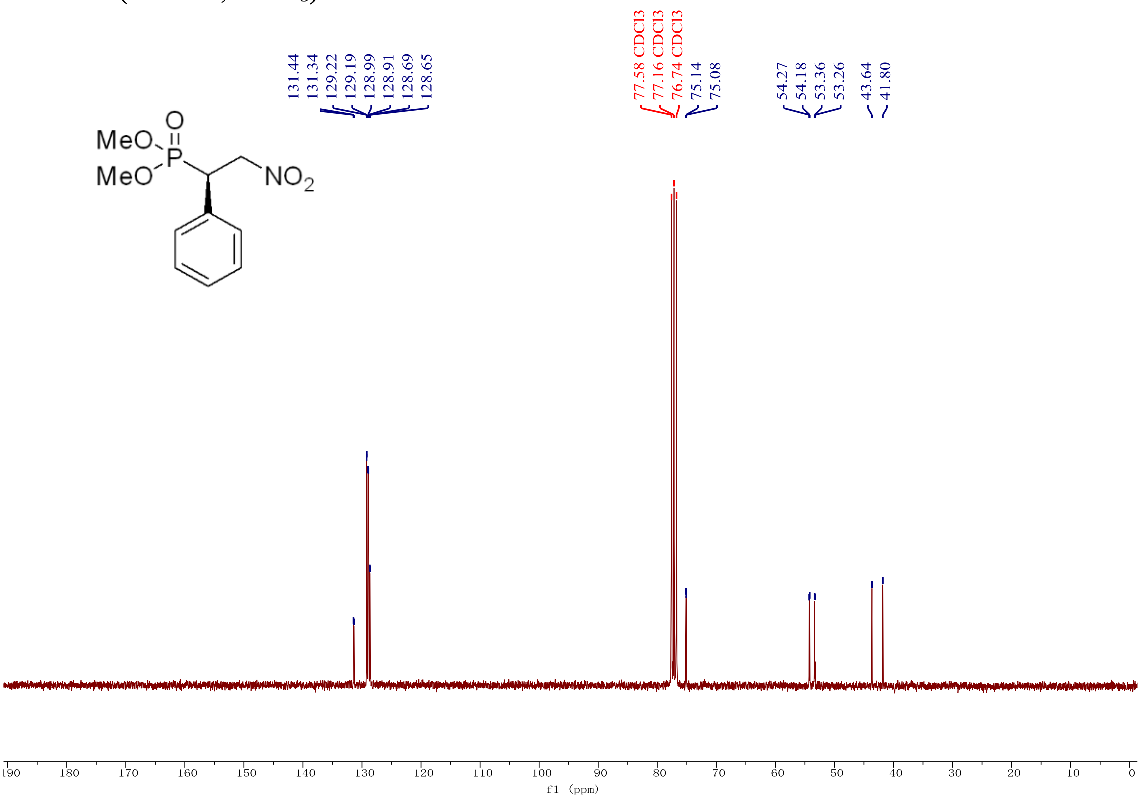

Fig. S324 ^{13}C NMR of compound **10aa**

^{31}P NMR (121 MHz, $CDCl_3$)

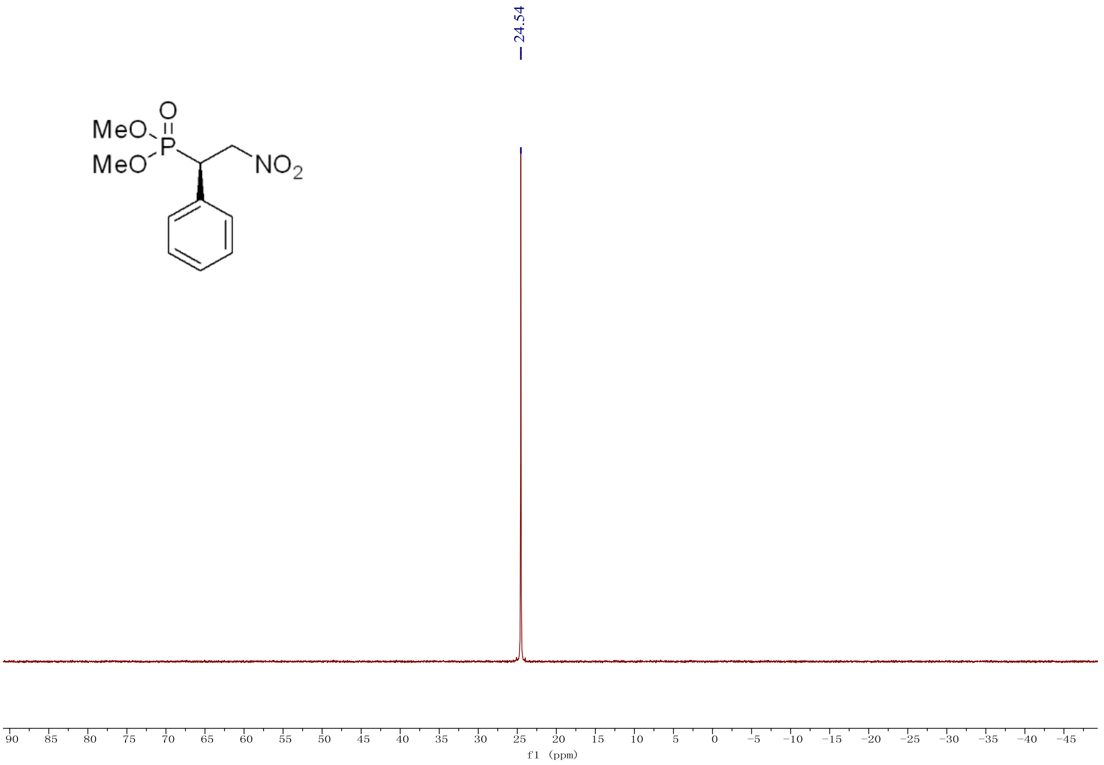

Fig. S325 ^{31}P NMR of compound **10aa**

^{1}H NMR (300 MHz, $CDCl_3$)

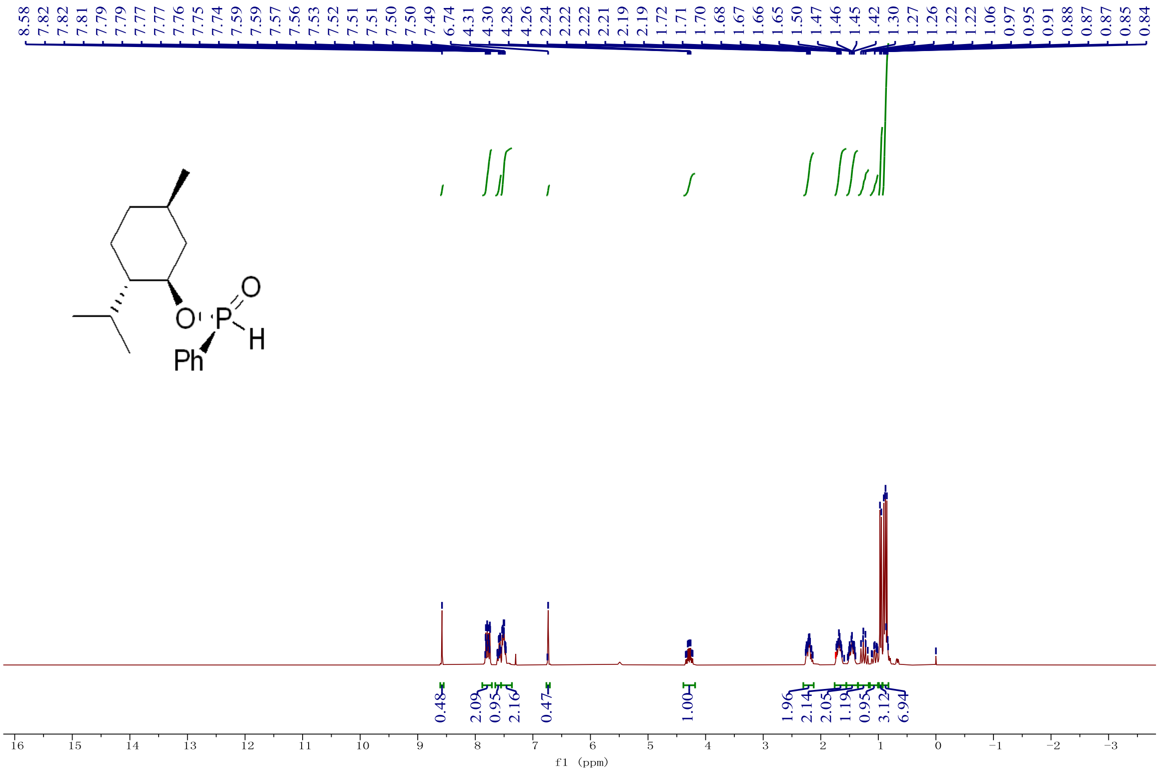

Fig. S326 ^{1}H NMR of compound **Menthyl-PHO**

^{13}P NMR (121 MHz, $CDCl_3$)

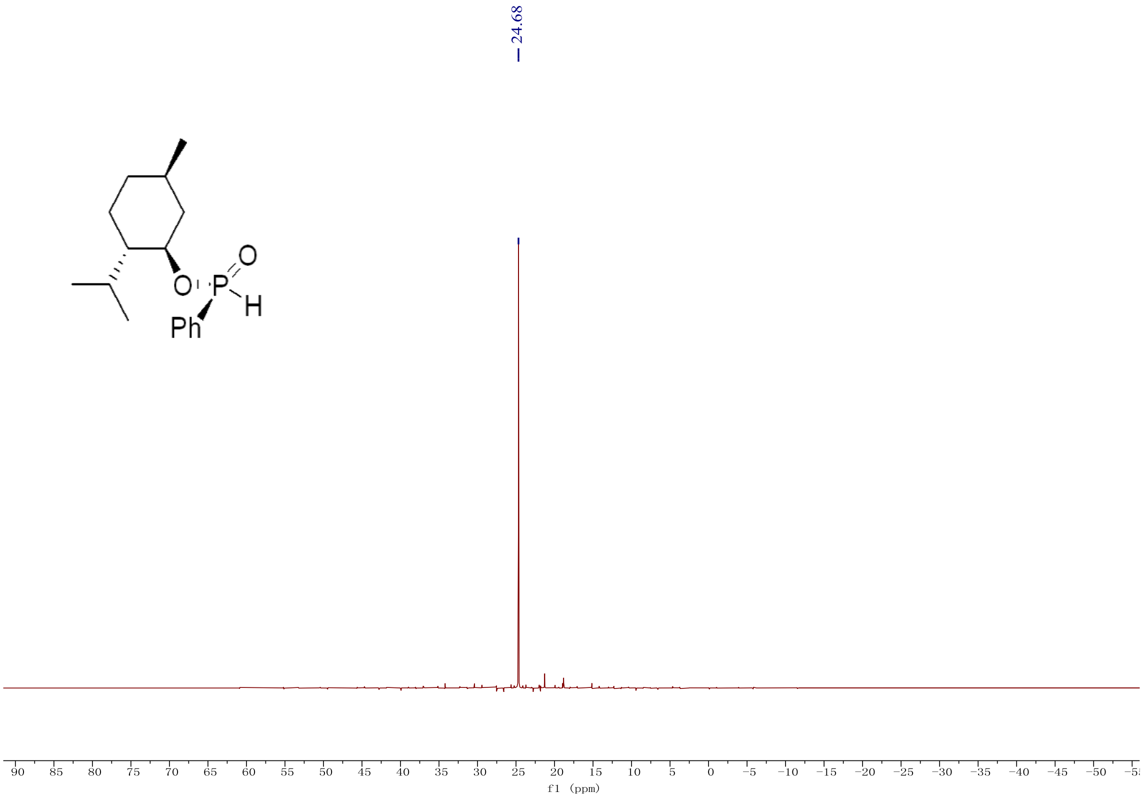

Fig. S327 ^{31}P NMR of compound **Menthyl-PHO**

^{1}H NMR (300 MHz, MeOD-d_4)

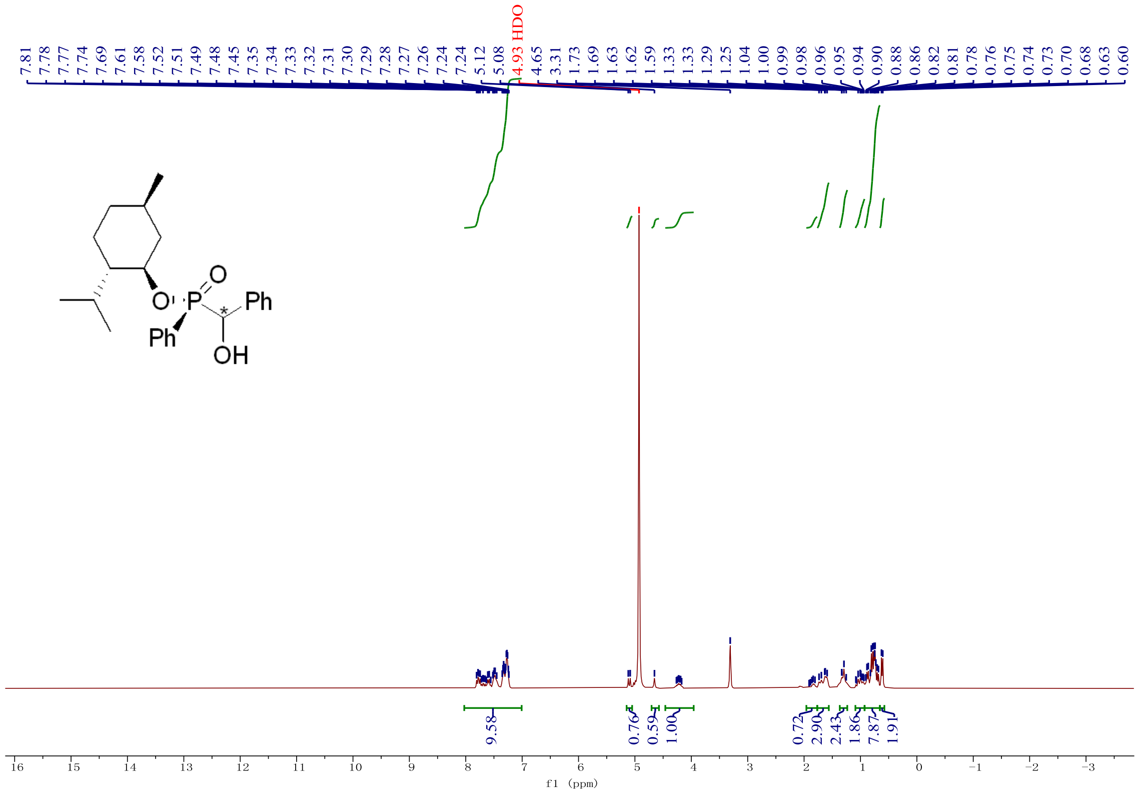

Fig. S328 ^{1}H NMR of compound **(1*R*,2*S*,5*R*)-2-isopropyl-5-methylcyclohexyl (*S*)-((*S*)-hydroxy(phenyl)methyl)(phenyl)phosphinate**

^{13}C NMR (75 MHz, MeOD-d_4)

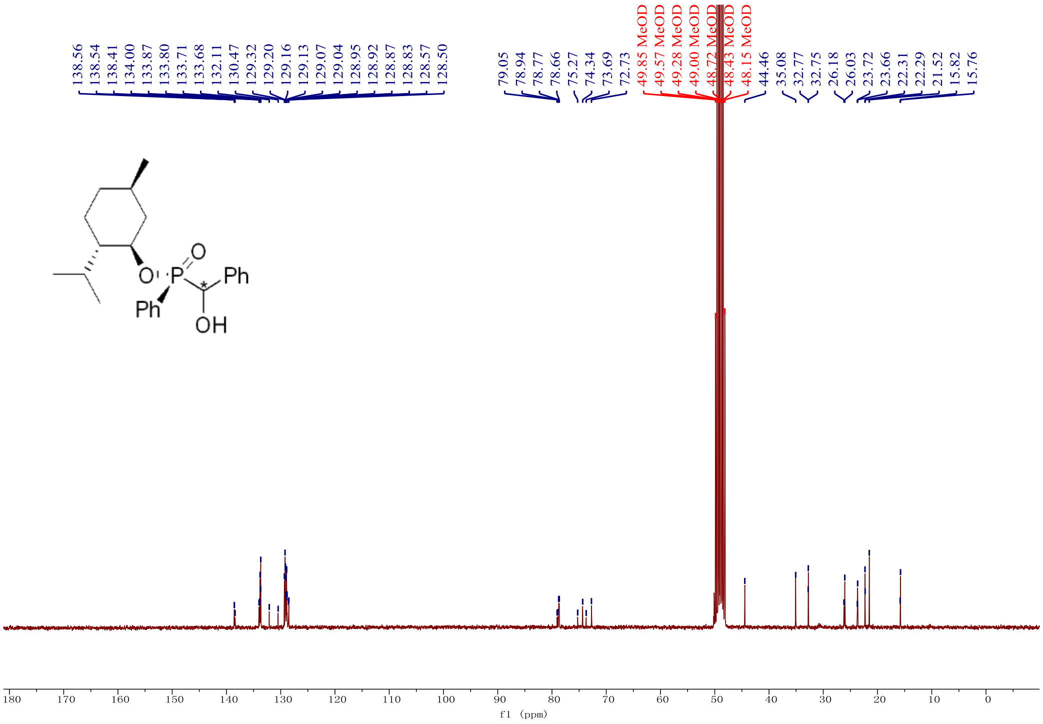

Fig. S329 ^{13}C NMR of compound **(1*R*,2*S*,5*R*)-2-isopropyl-5-methylcyclohexyl (*S*)-((*S*)-hydroxy(phenyl)methyl)(phenyl)phosphinate**

^{31}P NMR (121 MHz, MeOD-d_4)

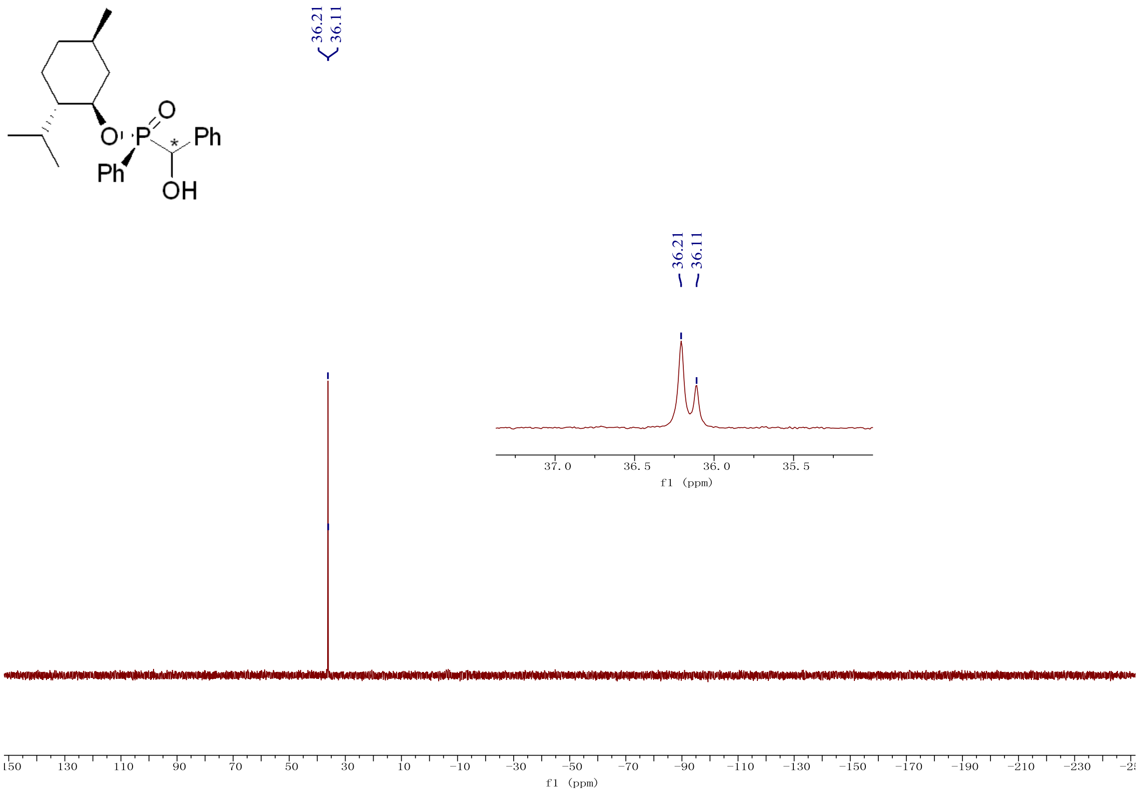

Fig. S330 ^{31}P NMR of compound **(1*R*,2*S*,5*R*)-2-isopropyl-5-methylcyclohexyl (*S*)-((*S*)-hydroxy(phenyl)methyl)(phenyl)phosphinate**

^{1}H NMR (300 MHz, $CDCl_3$)

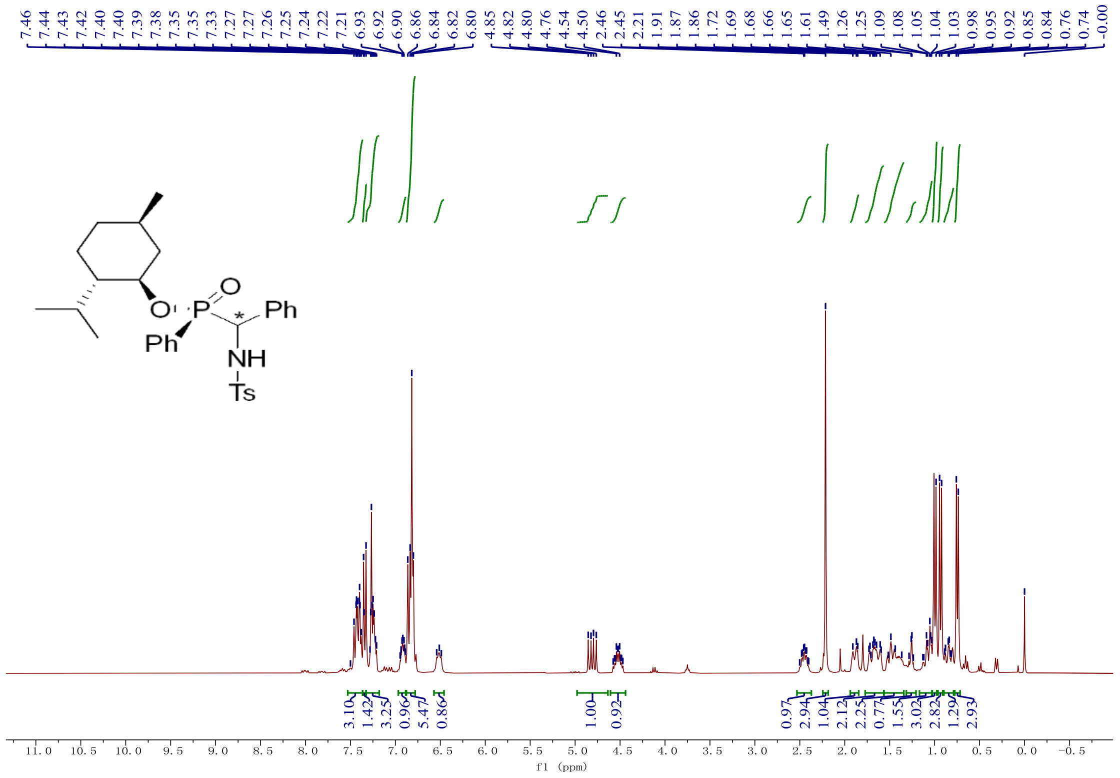

Fig. S331 ^{1}H NMR of compound **(1*R*,2*S*,5*R*)-2-isopropyl-5-methylcyclohexyl (*S*)-((*S*)-((4-methylphenyl)sulfonamido)(phenyl)methyl)(phenyl)phosphinate**

^{13}C NMR (75 MHz, $CDCl_3$)

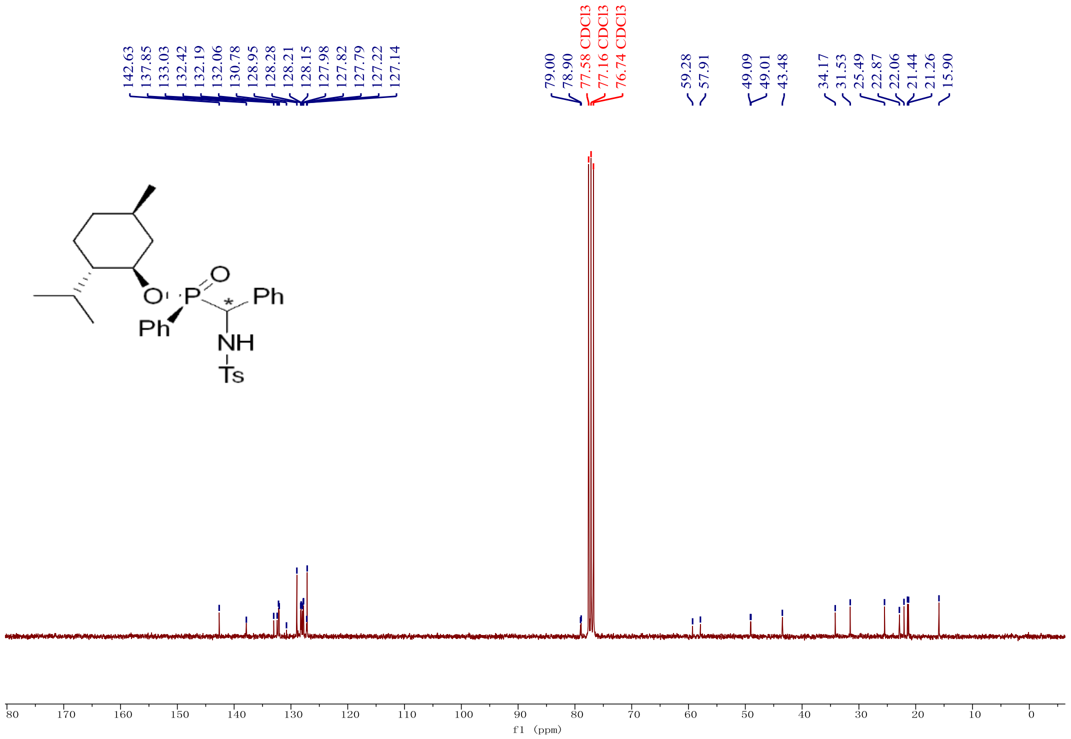

Fig. S332 ^{13}C NMR of compound **(1*R*,2*S*,5*R*)-2-isopropyl-5-methylcyclohexyl (*S*)-((*S*)-((4-methylphenyl)sulfonamido)(phenyl)methyl)(phenyl)phosphinate**

^{31}P NMR (121 MHz, $CDCl_3$)

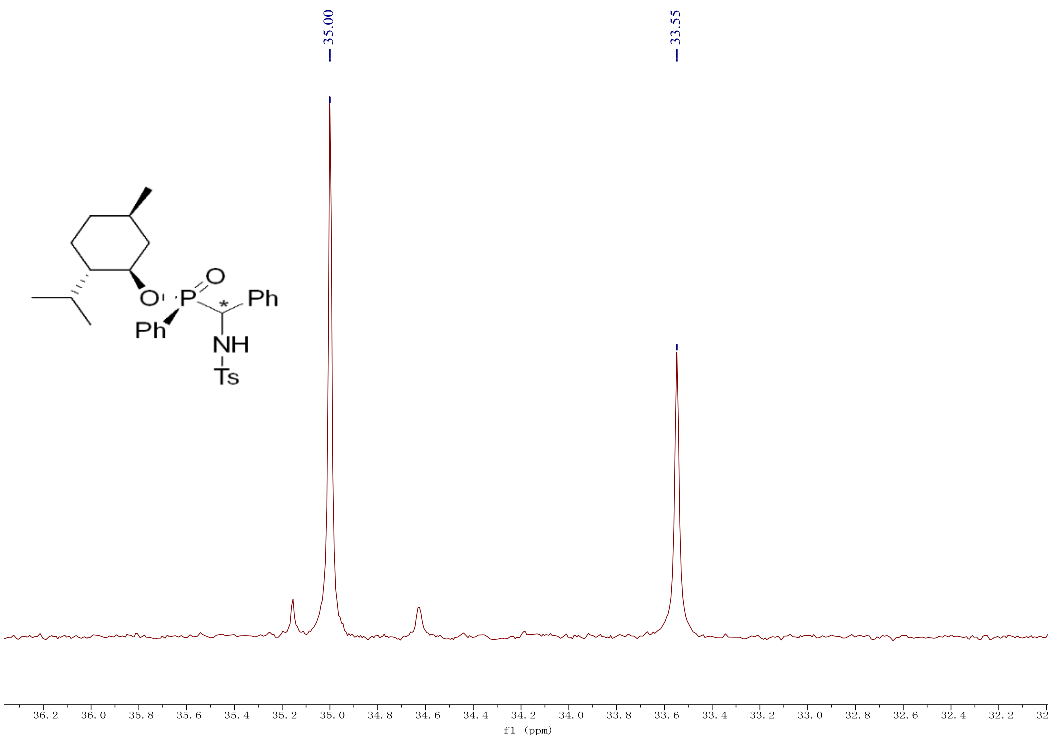

Fig. S333 ^{31}P NMR of compound **(1*R*,2*S*,5*R*)-2-isopropyl-5-methylcyclohexyl (*S*)-((*S*)-((4-methylphenyl)sulfonamido)(phenyl)methyl)(phenyl)phosphinate**

^{1}H NMR (300 MHz, $CDCl_3$)

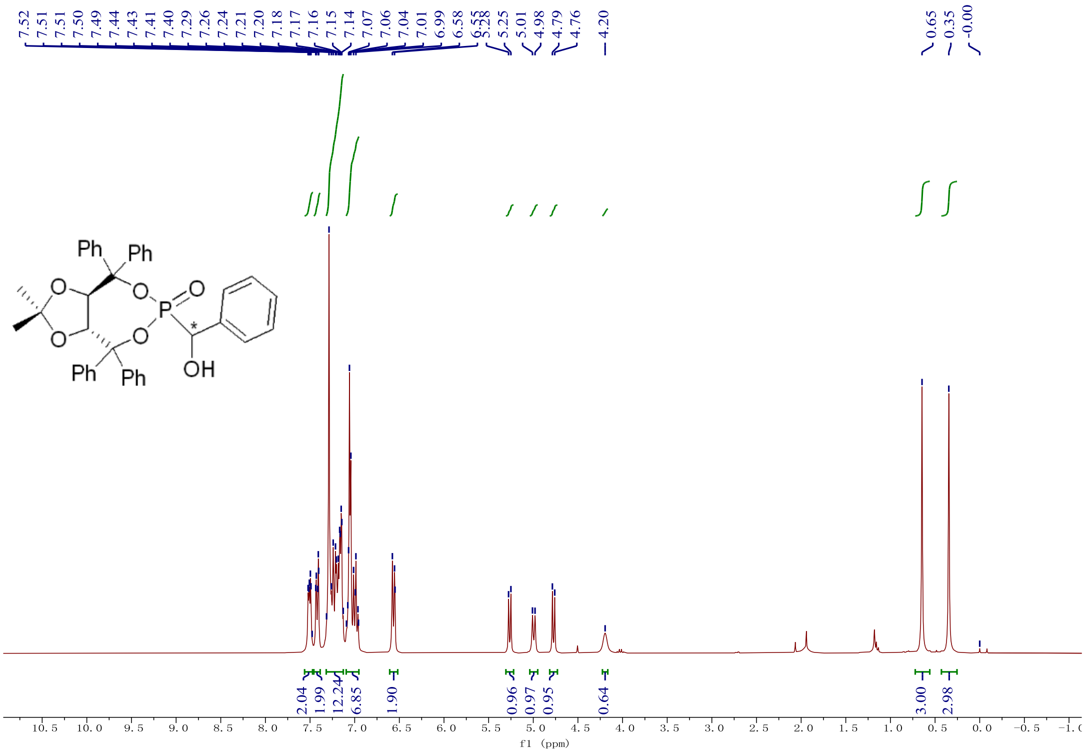

Fig. S334 ^{1}H NMR of compound **(3a*R*,8a*R*)-6-((*R*)-Hydroxy(phenyl)methyl)-2,2-dimethyl-4,4,8,8-tetraphenyltetrahydro-[1,3]dioxolo-[4,5-e][1,3,2]dioxaphosphepine 6-oxide**

^{13}C NMR (75 MHz, $CDCl_3$)

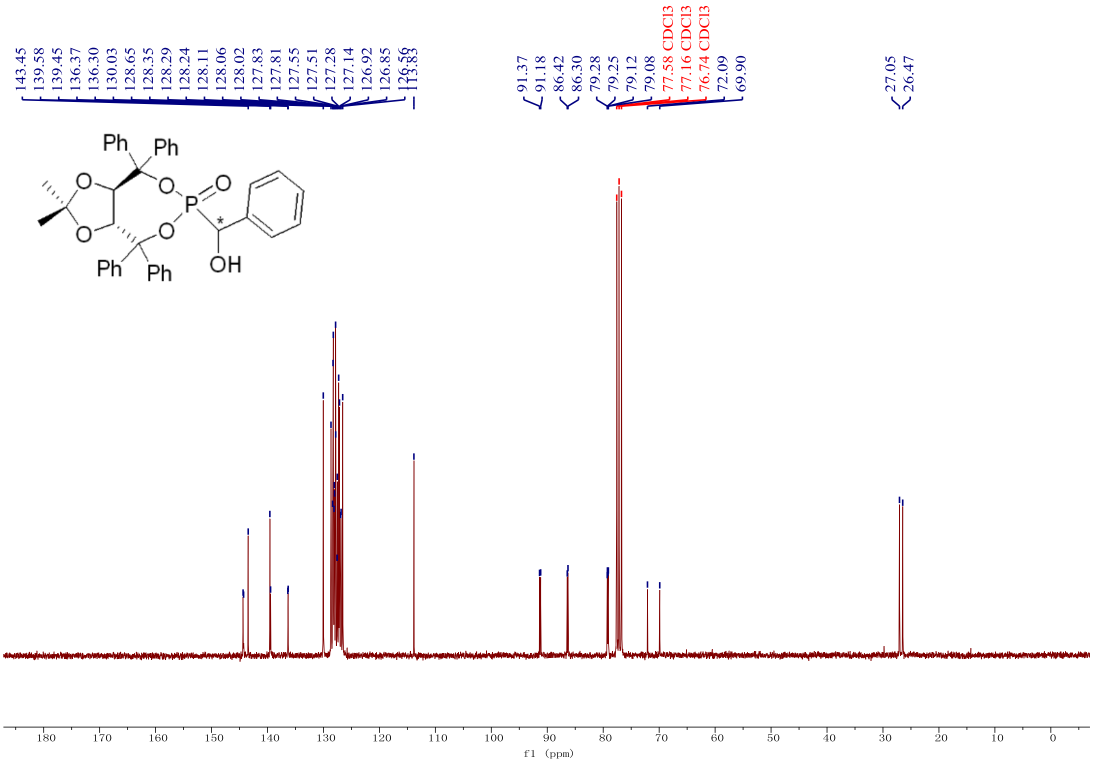

Fig. S335 ^{13}C NMR of compound **(3a*R*,8a*R*)-6-((*R*)-Hydroxy(phenyl)methyl)-2,2-dimethyl-4,4,8,8-tetraphenyltetrahydro-[1,3]dioxolo-[4,5-e][1,3,2]dioxaphosphepine 6-oxide**

^{31}P NMR (121 MHz, $CDCl_3$)

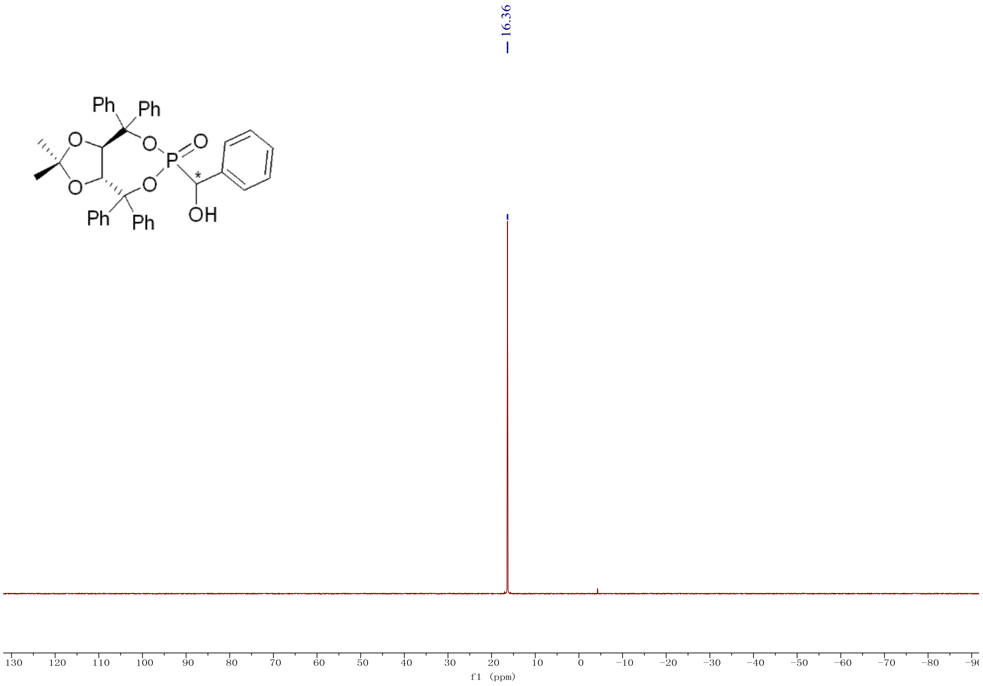

Fig. S336 ^{31}P NMR of compound **(3a*R*,8a*R*)-6-((*R*)-Hydroxy(phenyl)methyl)-2,2-dimethyl-4,4,8,8-tetraphenyltetrahydro-[1,3]dioxolo-[4,5-e][1,3,2]dioxaphosphepine 6-oxide**

^{1}H NMR (300 MHz, $CDCl_3$)

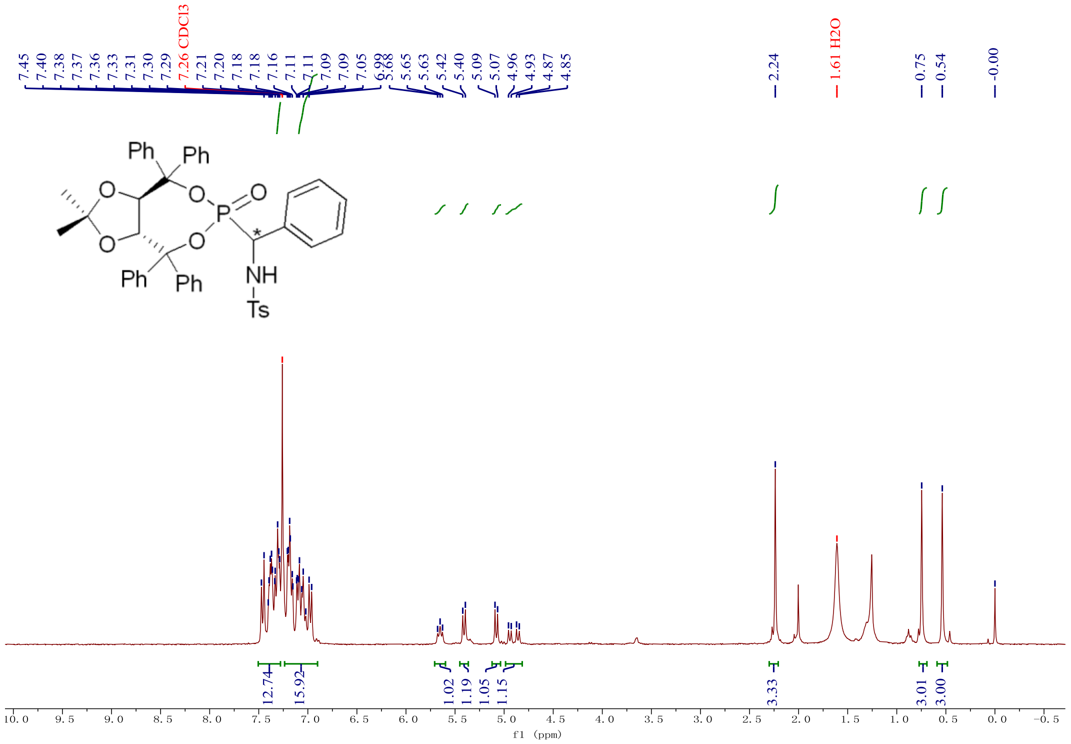

Fig. S337 ^{1}H NMR of compound ***N*-((*R*)-((3a*R*,8a*R*)-2,2-Dimethyl-6-oxido-4,4,8,8-tetraphenyltetrahydro-[1,3]dioxolo[4,5-e][1,3,2]dio-xaphosphepin-6-yl)(phenyl)methyl)-4-methylbenzenesulfonamide**

^{13}C NMR (75 MHz, $CDCl_3$)

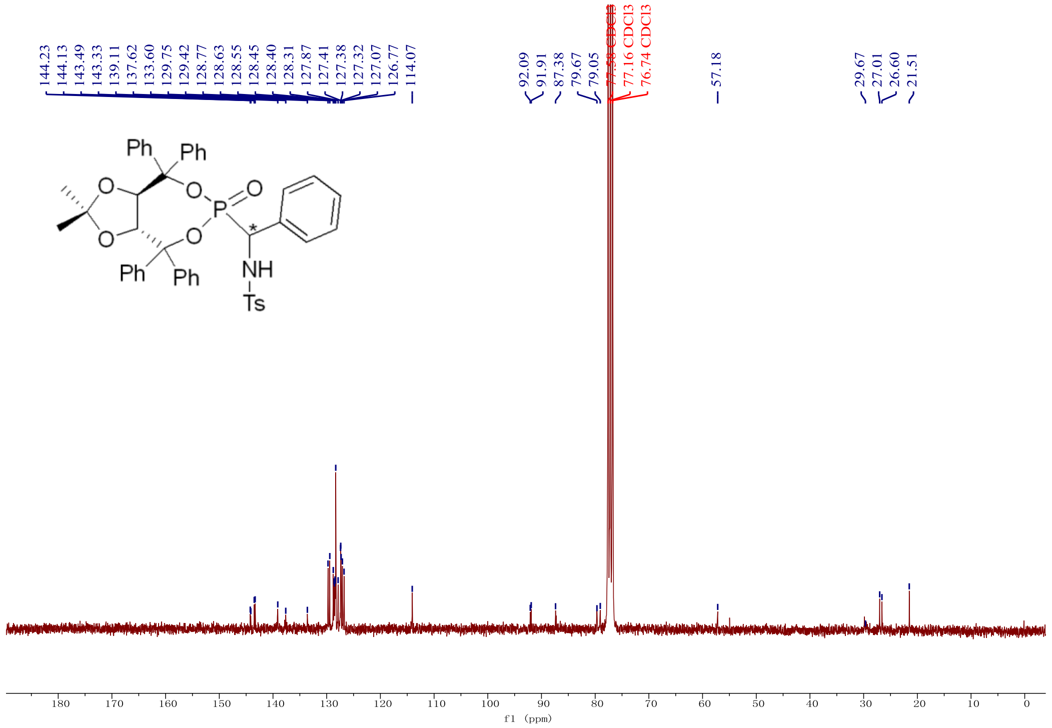

Fig. S338 ^{13}C NMR of compound ***N*-((*R*)-((3a*R*,8a*R*)-2,2-Dimethyl-6-oxido-4,4,8,8-tetraphenyltetrahydro-[1,3]dioxolo[4,5-e][1,3,2]dio-xaphosphepin-6-yl)(phenyl)methyl)-4-methylbenzenesulfonamide**

^{31}P NMR (121 MHz, $CDCl_3$)

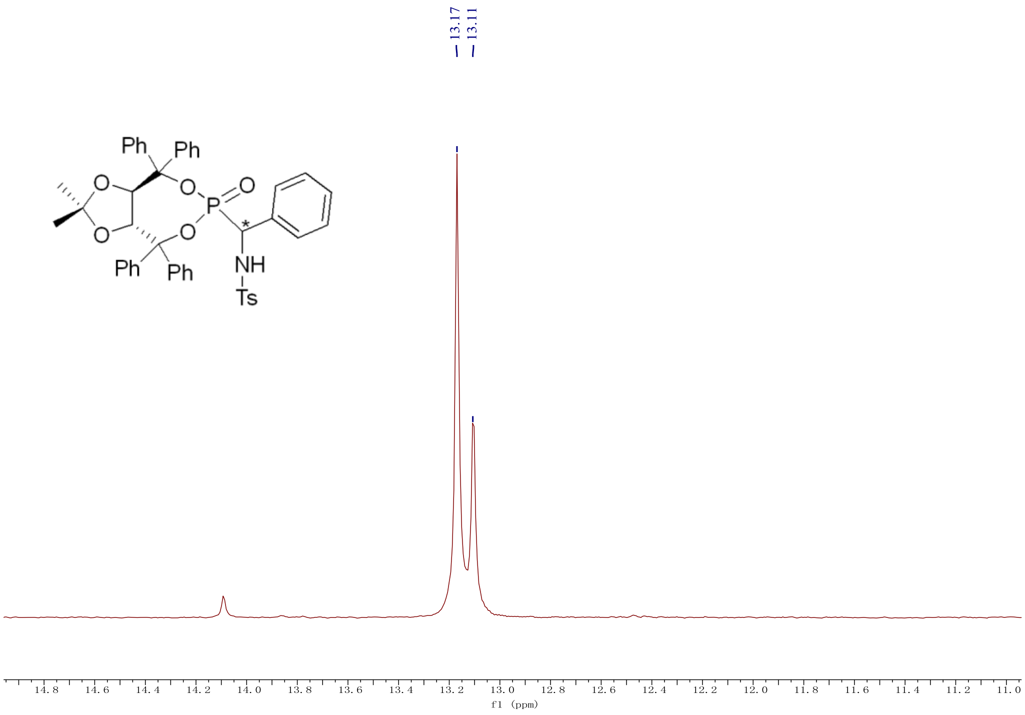

Fig. S339 ^{31}P NMR of compound ***N*-((*R*)-((3a*R*,8a*R*)-2,2-Dimethyl-6-oxido-4,4,8,8-tetraphenyltetrahydro-[1,3]dioxolo[4,5-e][1,3,2]dio-xaphosphepin-6-yl)(phenyl)methyl)-4-methylbenzenesulfonamide**

^{1}H NMR (300 MHz, $CDCl_3$)

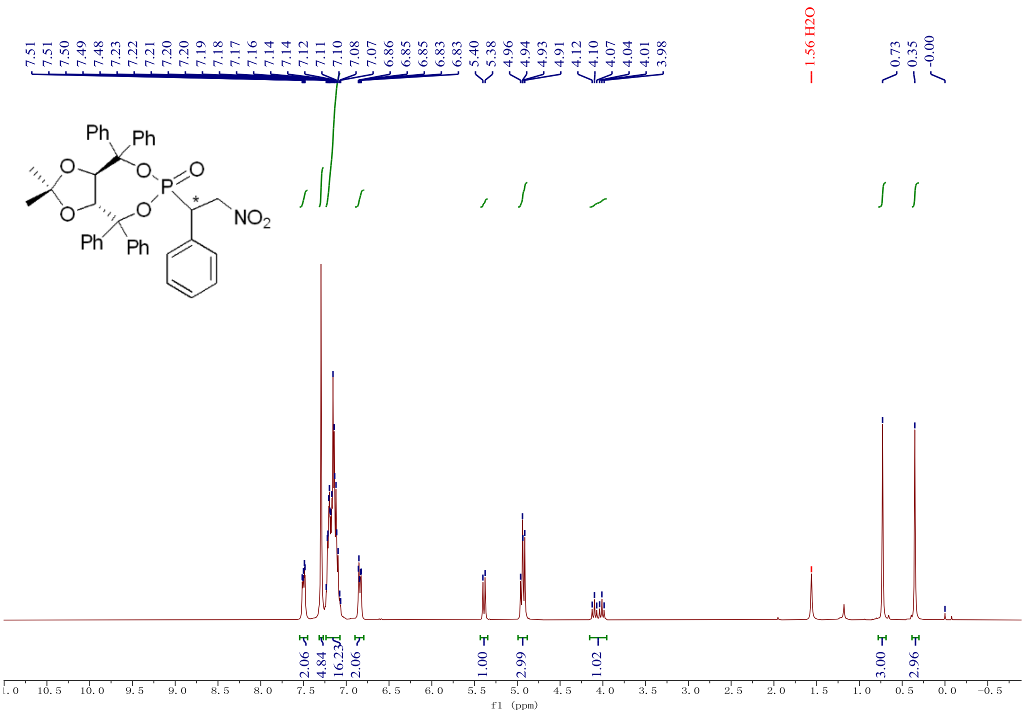

Fig. S340 ^{1}H NMR of compound **(3a*R*,8a*R*)-2,2-Dimethyl-6-((*R*)-2-nitro-1-phenylethyl)-4,4,8,8-tetraphenyltetrahydro-[1,3]dioxolo-[4,5-e][1,3,2]dioxaphosphepine 6-oxide**

^{13}C NMR (75 MHz, $CDCl_3$)

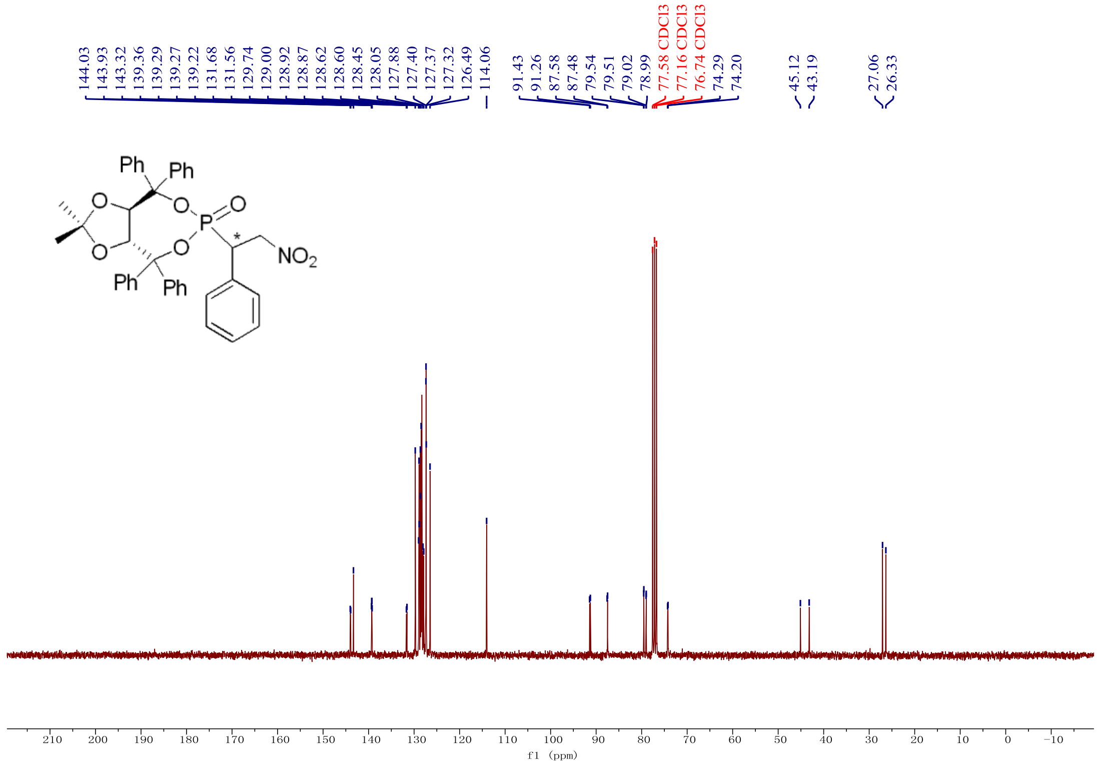

Fig. S341 ^{13}C NMR of compound **(3a*R*,8a*R*)-2,2-Dimethyl-6-((*R*)-2-nitro-1-phenylethyl)-4,4,8,8-tetraphenyltetrahydro-[1,3]dioxolo-[4,5-e][1,3,2]dioxaphosphepine 6-oxide**

^{31}P NMR (121 MHz, $CDCl_3$)

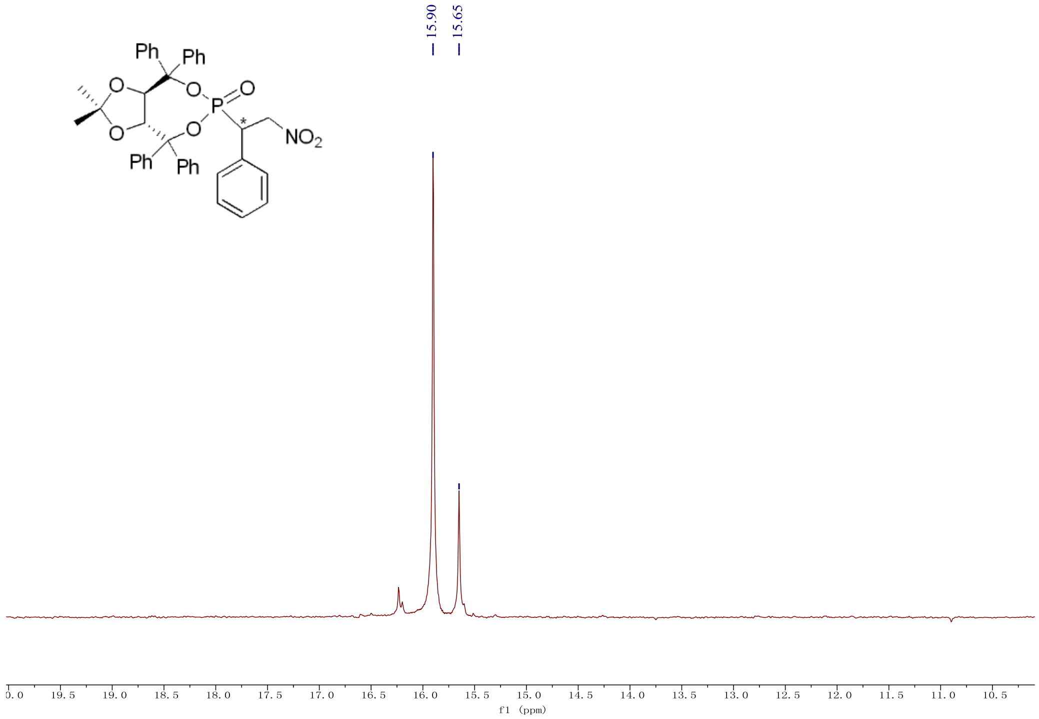

Fig. S342 ^{31}P NMR of compound **(3a*R*,8a*R*)-2,2-Dimethyl-6-((*R*)-2-nitro-1-phenylethyl)-4,4,8,8-tetraphenyltetrahydro-[1,3]dioxolo-[4,5-e][1,3,2]dioxaphosphepine 6-oxide**

^{1}H NMR (300 MHz, $CDCl_3$)

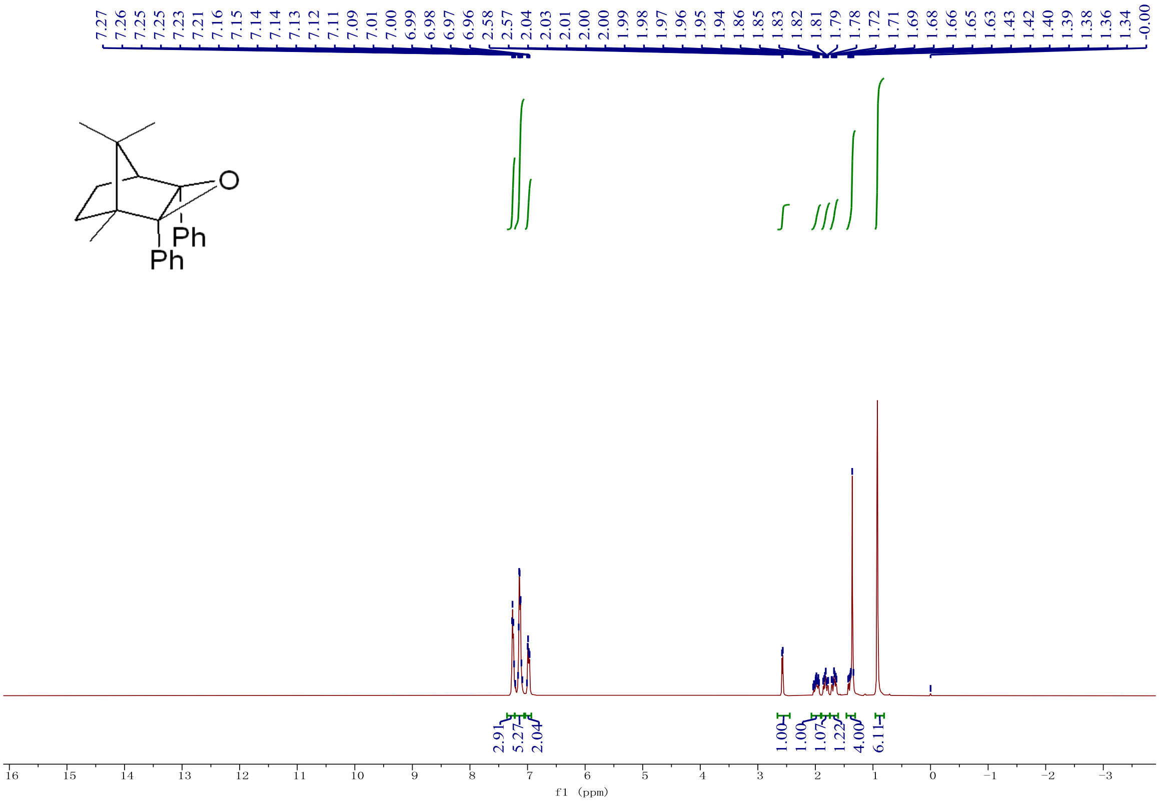

Fig. S343 ^{1}H NMR of compound **Epoxide-CAMDOL**

^{13}C NMR (75 MHz, $CDCl_3$)

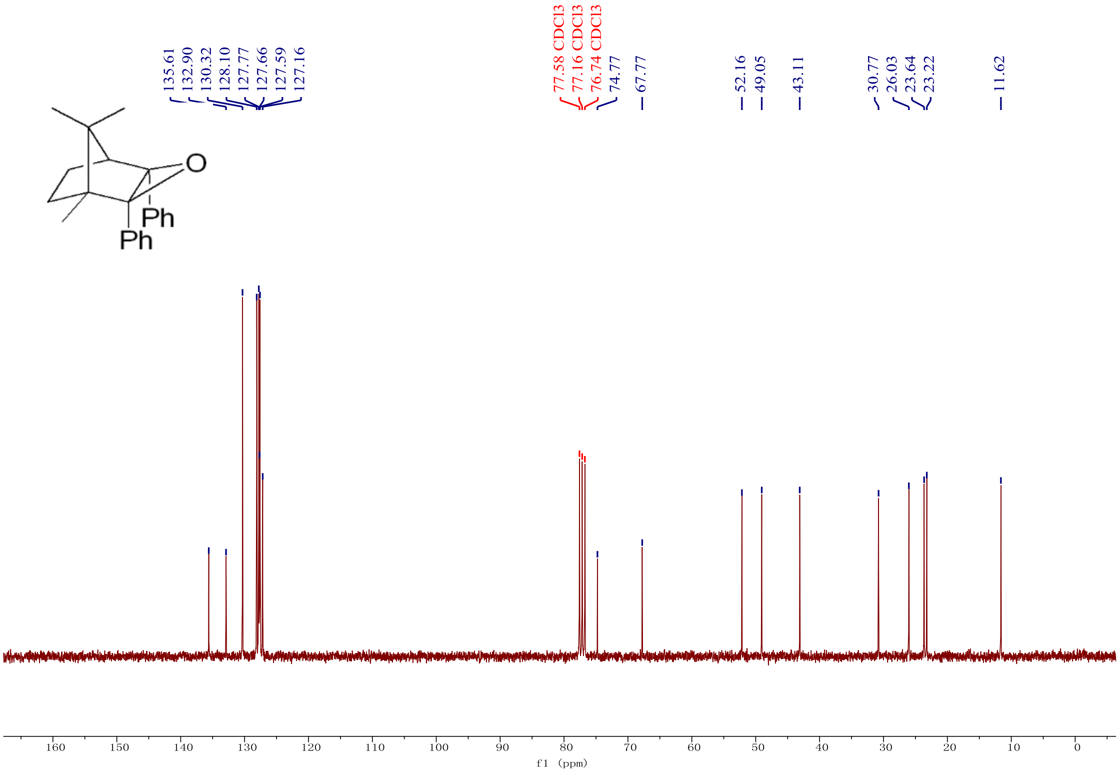

Fig. S344 ^{13}C NMR of compound **Epoxide-CAMDOL**

5. HPLC Diagrams

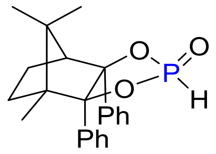

CAMDOL-PHO 1

Chiral HPLC: Chiralpak **AS-RH** column, Water/Acetonitrile= 10/90, flow rate = 1.0 mL/min, λ = 210 nm, 99% *dr*.

RAC-CAMDOL-PHO 1

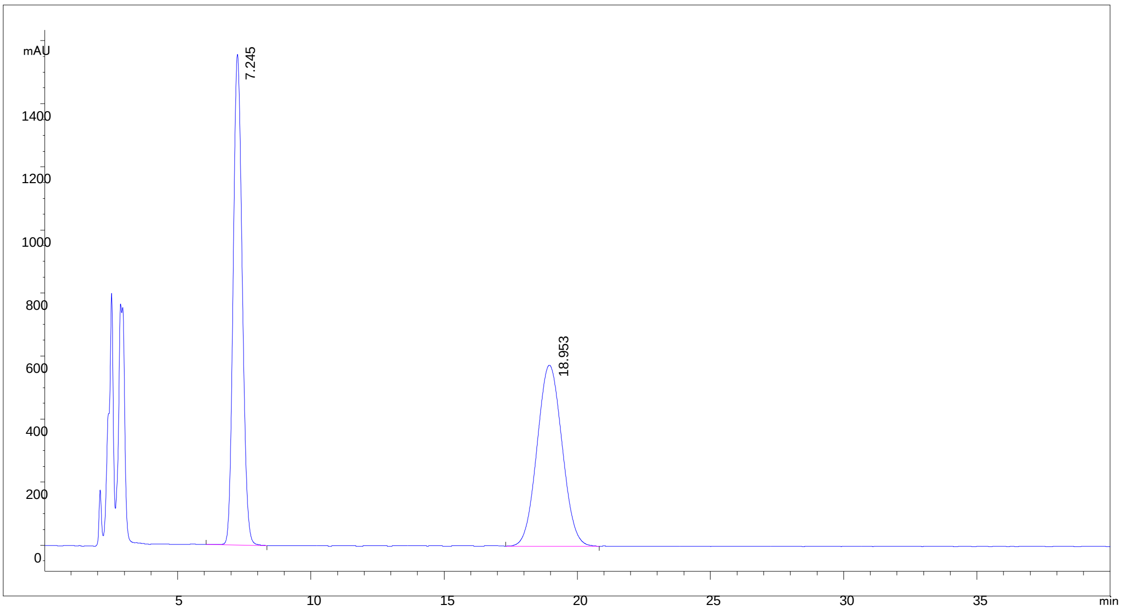

Index	t_R/ (min)	Area / (mv.sec)	Height (mv)	% Area
1	7.245	36839.8	1556	49.597
2	18.953	37439.2	574.1	50.403
Total				100

Chiral- CAMDOL-PHO 1

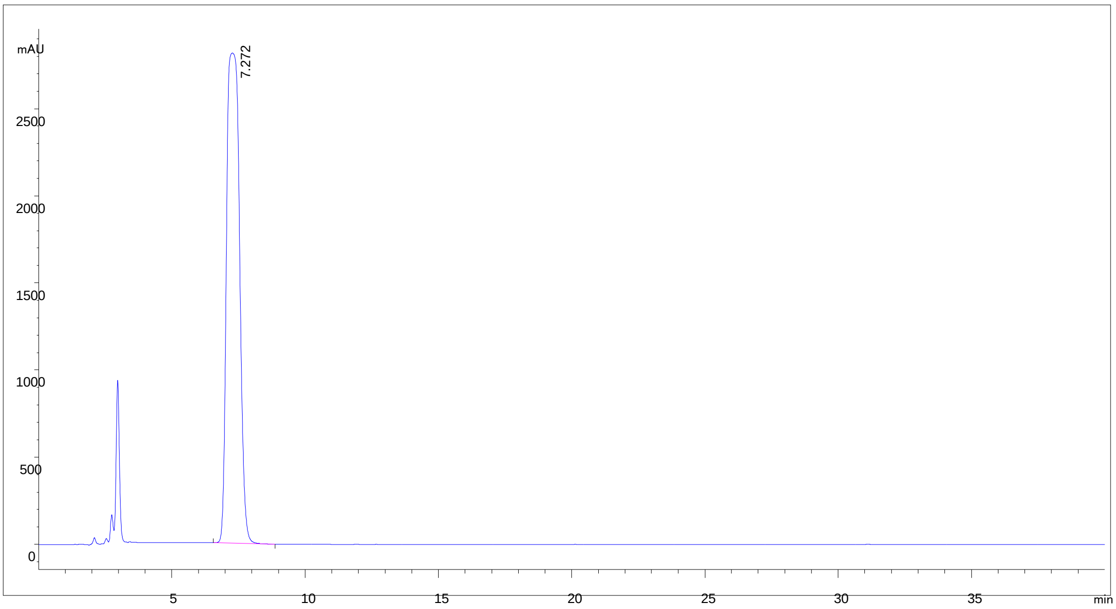

Index	t_R/ (min)	Area / (mv.sec)	Height (mv)	% Area
1	7.272	94856.2	2812.1	100
Total				100

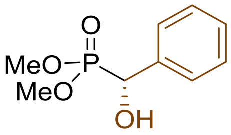

4b

Chiral HPLC: Chiralpak **OD-H** column, Hexane/IPA= 90/10, flow rate = 1.0 mL/min, λ = 210 nm, 97% ee.

RAC-4b

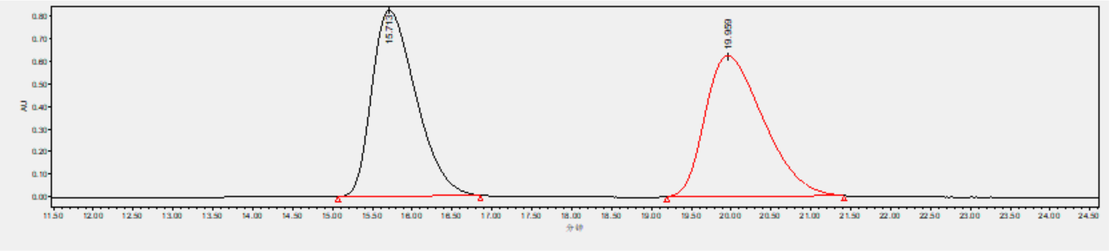

Index	t_R/ (min)	Area / (mv.sec)	Height (mv)	% Area
1	15.713	31592569	822114	49.77
2	19.959	31881572	624613	50.23
Total				100

Chiral-4b

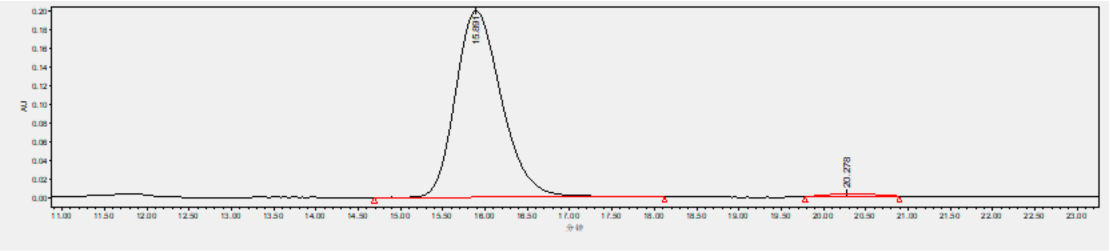

Index	t_R/ (min)	Area / (mv.sec)	Height (mv)	% Area
1	15.891	7566576	200067	98.50
2	20.278	115194	3089	1.50
Total				100

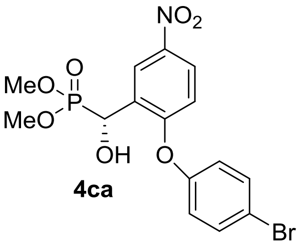

4ca

Chiral HPLC: Chiralpak **IF-H** column, Hexane/EtOH= 75/25, flow rate = 1.0 mL/min, λ = 254 nm, 98% *ee*.

RAC-4ca

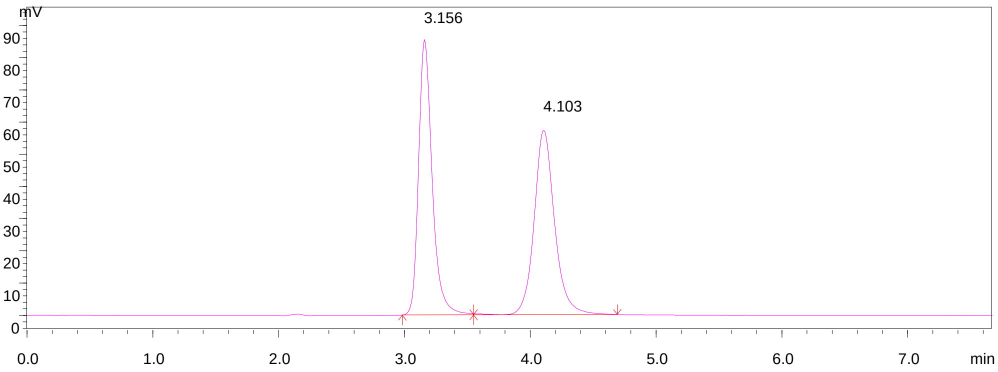

Index	t_R/ (min)	Area / (mv.sec)	T.Plate	% Area
1	3.156	646688	3882.269	50.01
2	4.103	646235	3181.120	49.99
Total				100

Chiral-4ca

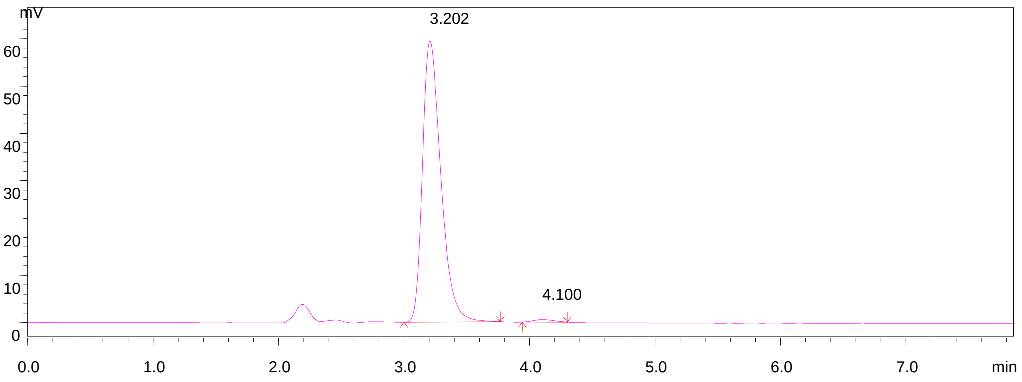

Index	t_R/ (min)	Area / (mv.sec)	T.Plate	% Area
1	3.202	607907	2220.322	99.02
2	4.100	6026	2783.176	0.98
Total				100

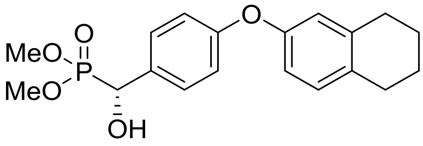

4da

Chiral HPLC: Chiralpak **IK-H** column, Hexane/IPA= 90/10, flow rate = 1.0 mL/min, λ = 210 nm, 97% *ee*.

RAC-4da

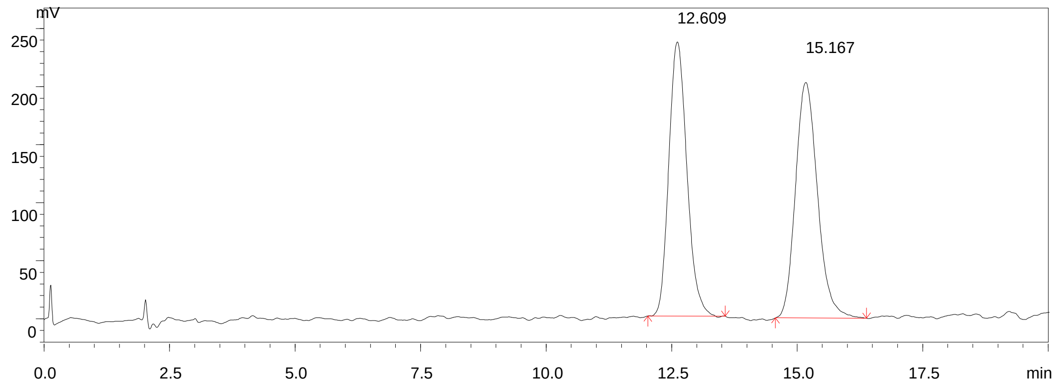

Index	t_R/ (min)	Area / (mv.sec)	T.Plate	% Area
1	12.609	5918366	5832.697	48.83
2	15.167	6202656	5832.132	51.17
Total				100

Chiral-4da

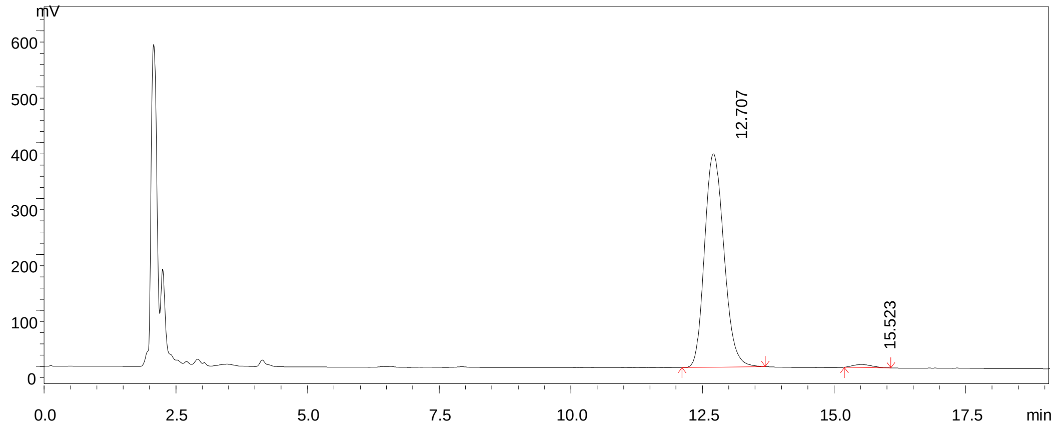

Index	t_R/ (min)	Area / (mv.sec)	T.Plate	% Area
1	12.707	9802891	4197.405	98.67
2	15.523	132316	3100.274	1.33
Total				100

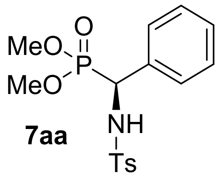

7aa

Chiral HPLC: Chiralpak **AY-H** column, Hexane/EtOH= 60/40, flow rate = 1.0 mL/min, λ = 210 nm, >98% *ee*.

RAC-7aa

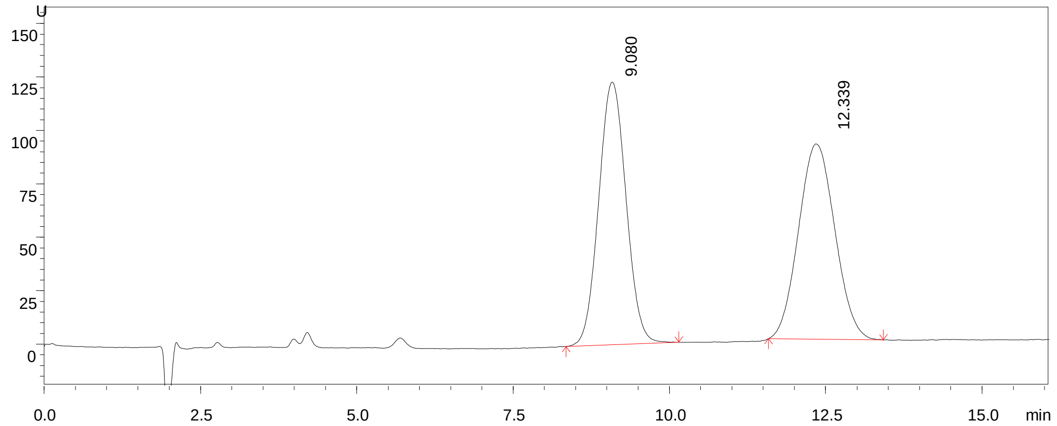

Index	t_R/ (min)	Area / (mv.sec)	T.Plate	% Area
1	9.080	3737911	2039.488	50.29
2	12.339	3695242	2089.182	49.71
Total				100

Chiral-7aa

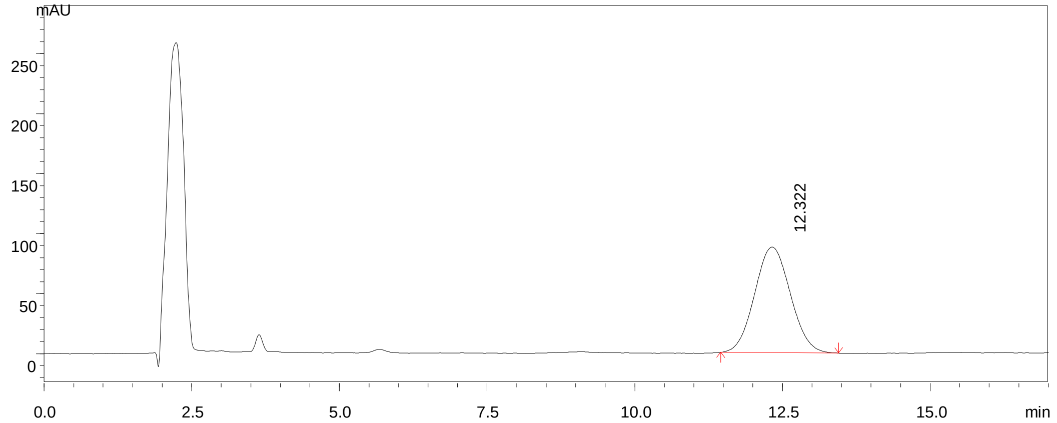

Index	t_R/ (min)	Area / (mv.sec)	T.Plate	% Area
2	12.322	3707614	1944.691	100
Total				100

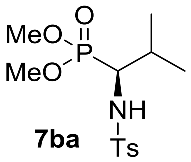

Chiral HPLC: Chiralpak **AY-H** column, Hexane/IPA = 73/27, flow rate = 1.0 mL/min, λ = 210 nm, 98% *ee*.

RAC-7ba

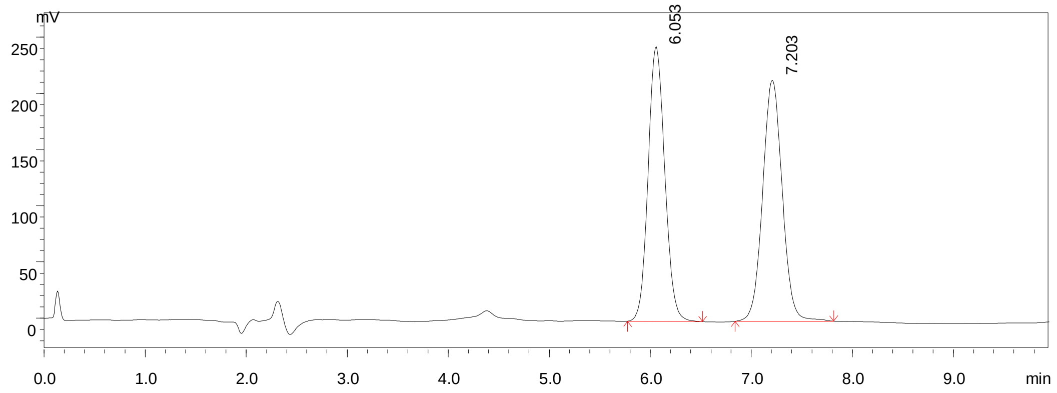

Index	t_R/ (min)	Area / (mv.sec)	T.Plate	% Area
1	6.053	2813286	6126.995	49.31
2	7.203	2892483	6546.004	50.69
Total				100

Chiral-7ba

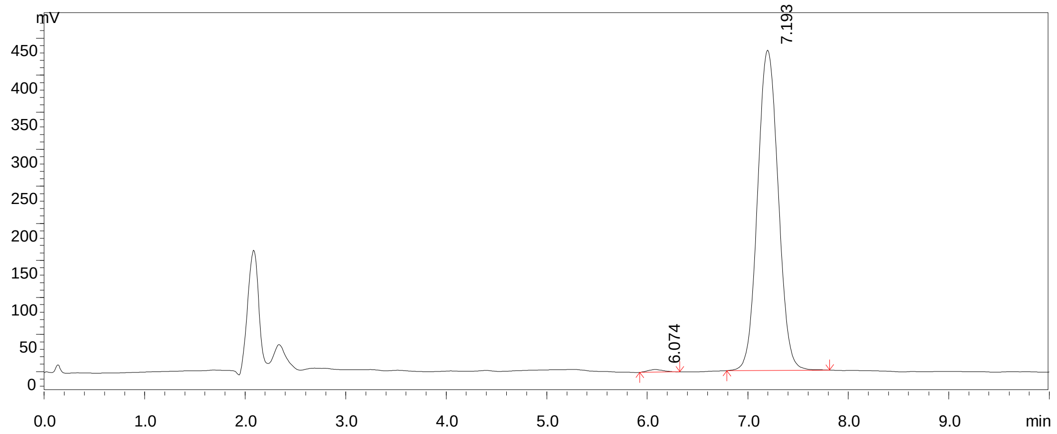

Index	t_R/ (min)	Area / (mv.sec)	T.Plate	% Area
1	6.074	40214	5987.551	0.65
2	7.193	6180959	5790.481	99.35
Total				100

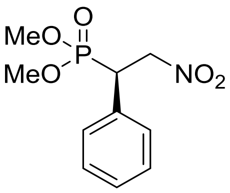

10aa

Chiral HPLC: Chiralpak **AY-H** column, Hexane/IPA= 73/27, flow rate = 1.0 mL/min, λ = 210 nm, 97% ee.

RAC-10aa

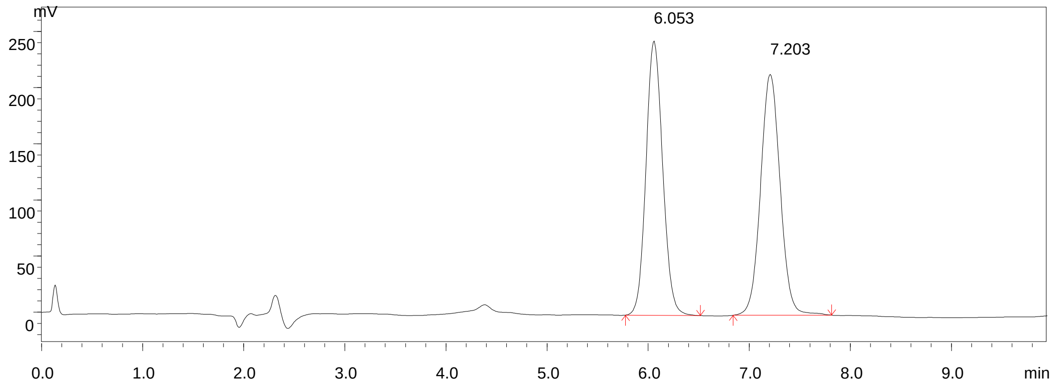

Index	t_R/ (min)	Area / (mv.sec)	T.Plate	% Area
1	6.053	2813286	6126.995	49.31
2	7.203	2892483	6546.004	50.69
Total				100

Chiral-10aa

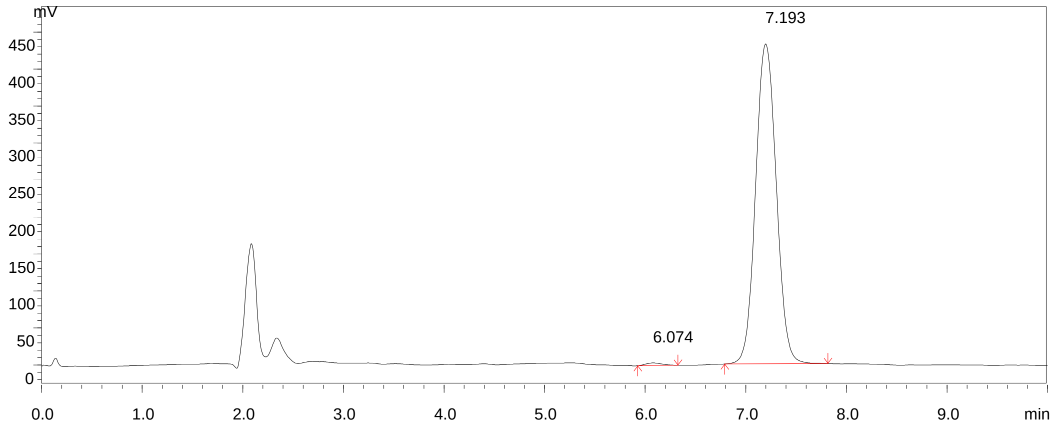

Index	t_R/ (min)	Area / (mv.sec)	T.Plate	% Area
1	6.074	40214	5987.551	1.30
2	7.193	6180959	5790.481	98.7
Total				100

6. X-ray crystallography data

6.1 X-ray crystallography data of CAMDOL-PHO 1

Through **experiment section 2.1**, white single crystal (CCDC 2402270) can be directly generated. A white crystal of **CAMDOL-PHO 1** was mounted on a glass fiber at a random orientation. Thermal ellipsoids are drawn at 50% probability.

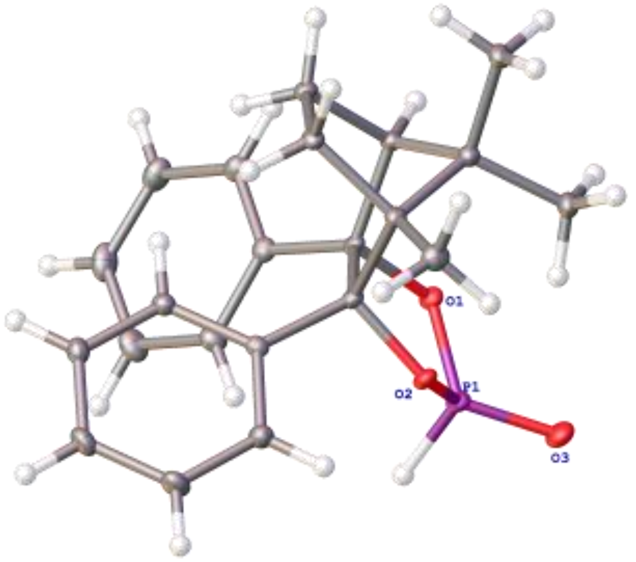

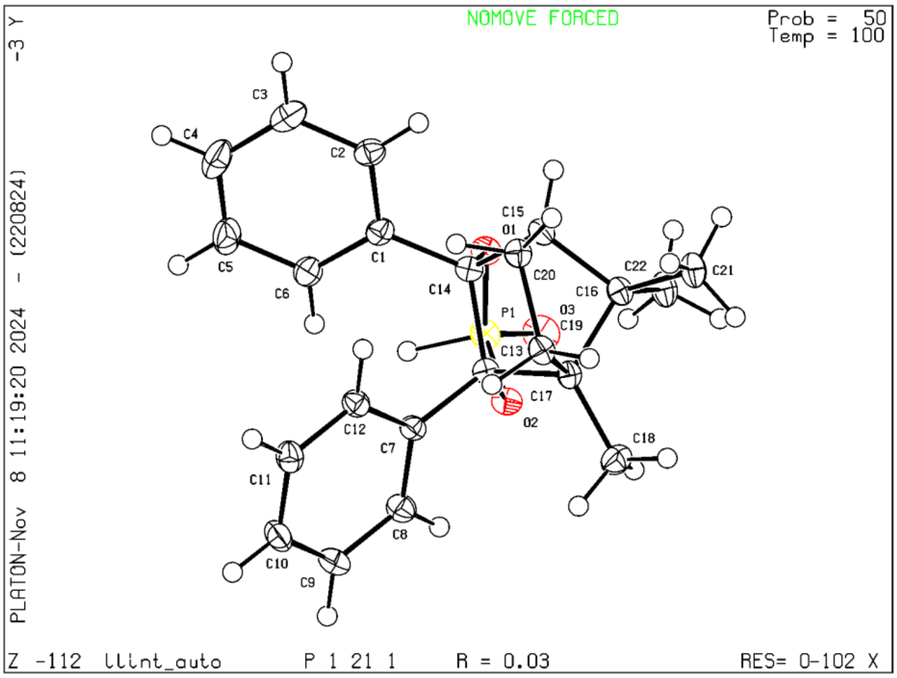

Figure S345. The ellipsoid plot of CAMDOL-PHO 1 and the molecular structure of CAMDOL-PHO 1 with the atomic numbering

Table S1 Crystal data and structure refinement for CAMDOL-PHO 1 after oxidatio

Identification code	**CAMDOL-PHO 1**
Empirical formula	$C_{22}H_{25}O_3P$
Formula weight	368.39
Temperature/K	100.00(10)
Crystal system	monoclinic
Space group	$P2_1$
a/Å	10.20020(10)
b/Å	8.82840(10)
c/Å	10.47620(10)
α/°	90
β/°	93.8840(10)
γ/°	90
Volume/Å^3	941.230(17)
Z	2
ρ_{calc}g/cm^3	1.300
μ/mm^{-1}	1.442
F(000)	392.0
Crystal size/mm^3	0.14 × 0.1 × 0.08
Radiation	Cu Kα (λ = 1.54184)
2Θ range for data collection/°	8.46 to 145.646
Index ranges	$-12 \leq h \leq 12$, $-9 \leq k \leq 10$, $-12 \leq l \leq 12$
Reflections collected	11619
Independent reflections	3368 [R_{int} = 0.0290, R_{sigma} = 0.0160]
Data/restraints/parameters	3368/1/242
Goodness-of-fit on F^2	1.007
Final R indexes [I>=2σ (I)]	R_1 = 0.0323, wR_2 = 0.0867
Final R indexes [all data]	R_1 = 0.0323, wR_2 = 0.0868
Largest diff. peak/hole / e Å^{-3}	0.26/-0.27
Flack/Hooft parameter	-0.015(19)/-0.015(4)

6.2 X-ray crystallography data of 3a

A single crystal of **3a** (CCDC 2330640) was obtained by slowly evaporating a mixture of EtOAC and Hexane solution (5:1) at ambient temperature. A colorless crystal of **3a** was mounted on a glass fiber at a random orientation. Thermal ellipsoids are drawn at 50% probability.

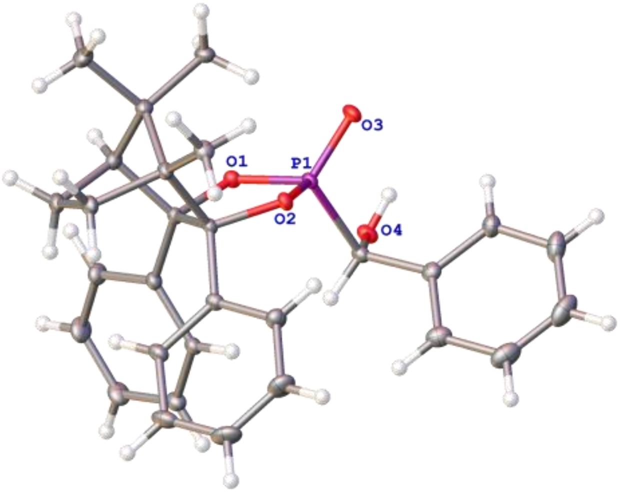

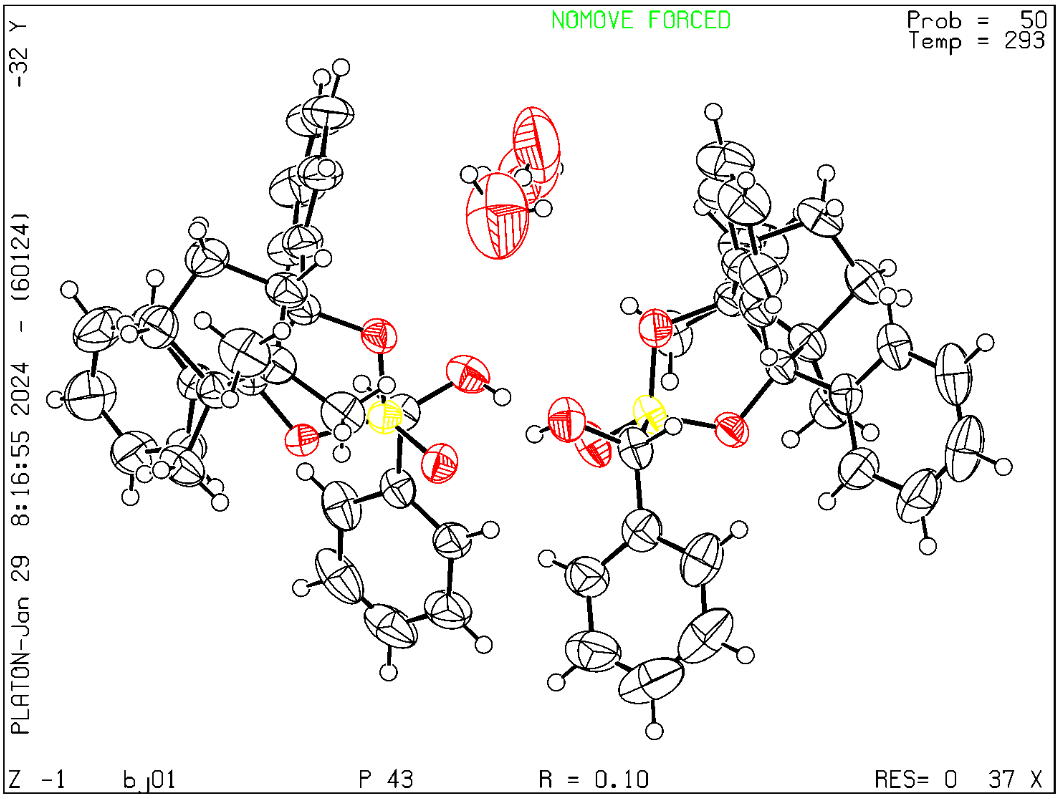

Figure S346. The ellipsoid plot of 3a and the molecular structure of 3a with the atomic numbering

Table S2 Crystal data and structure refinement for 3a after oxidatio

Identification code	**3a**
Empirical formula	$C_{29}H_{31}O_4P$
Formula weight	474.51
Temperature/K	293.15
Crystal system	tetragonal
Space group	$P4_3$
a/Å	16.72503(14)
b/Å	16.72503(14)
c/Å	20.1957(2)
α/°	90
β/°	90
γ/°	90
Volume/Å^3	5649.27(12)
Z	8
ρ_{calc}g/cm^3	1.169
μ/mm^{-1}	1.148
F(000)	2116.0
Crystal size/mm^3	0.16 × 0.13 × 0.11
Radiation	Cu Kα (λ = 1.54184)
2Θ range for data collection/°	5.284 to 152.99
Index ranges	$-19 \le h \le 16$, $-20 \le k \le 21$, $-25 \le l \le 25$
Reflections collected	36222
Independent reflections	11316 [R_{int} = 0.0301, R_{sigma} = 0.0261]
Data/restraints/parameters	11316/1/657
Goodness-of-fit on F^2	1.301
Final R indexes [I>=2σ (I)]	R_1 = 0.1007, wR_2 = 0.2730
Final R indexes [all data]	R_1 = 0.1025, wR_2 = 0.2753
Largest diff. peak/hole / e Å^{-3}	0.86/-0.41
Flack/Hooft parameter	0.02(4)/0.015(5)

6.3 X-ray crystallography data of 6a

A single crystal of **6a** (CCDC 2330642) was obtained by slowly evaporating a mixture of EtOAC and Hexane solution (5:1) at ambient temperature. A colorless crystal of **6a** was mounted on a glass fiber at a random orientation. Thermal ellipsoids are drawn at 50% probability.

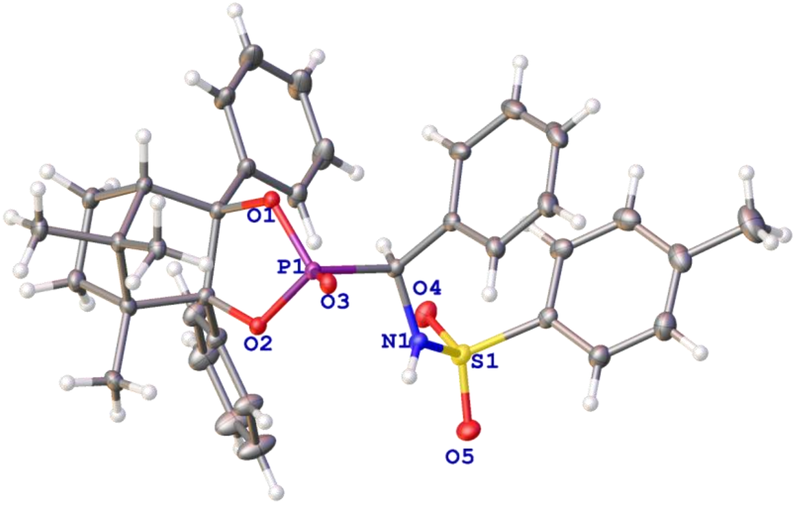

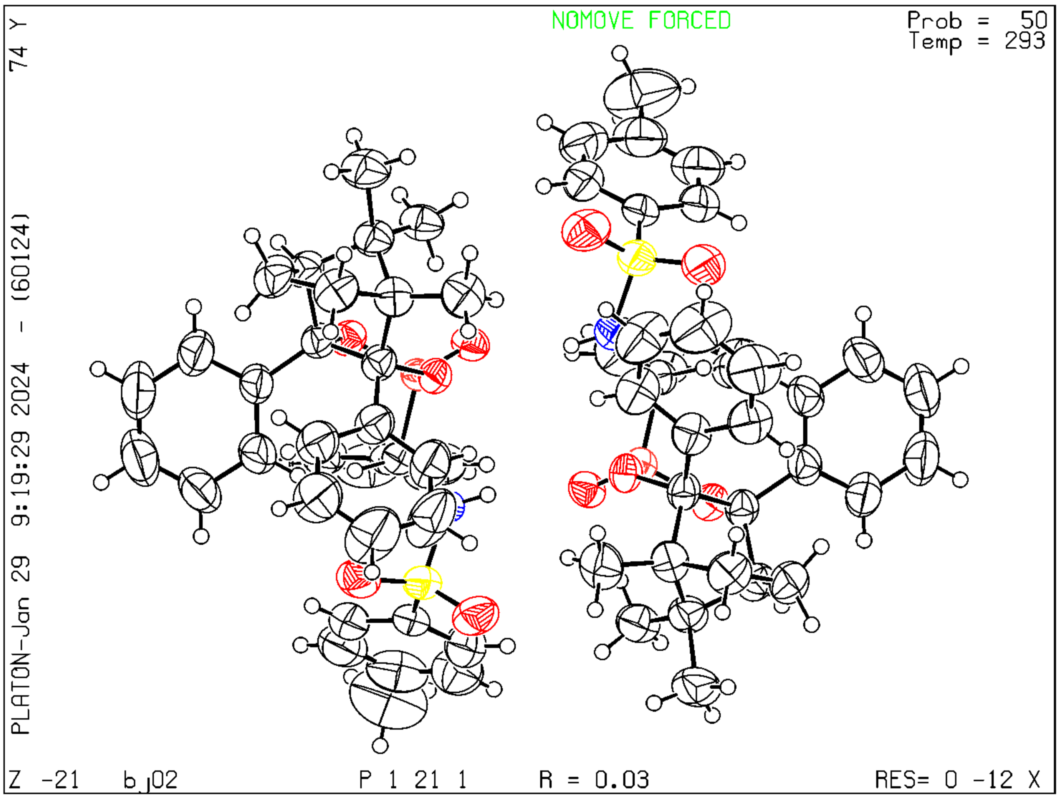

Figure S347. The ellipsoid plot of 6a and the molecular structure of 6a with the atomic numbering

Table S3 Crystal data and structure refinement for 6a after oxidatio

Crystal data and structure refinement for 6a.	
Identification code	6a
Empirical formula	$C_{36}H_{38}NO_5PS$
Formula weight	627.70
Temperature/K	293.15
Crystal system	monoclinic
Space group	$P2_1$
a/Å	12.84397(17)
b/Å	19.93976(19)
c/Å	14.01120(18)
α/°	90
β/°	107.6368(14)
γ/°	90
Volume/Å^3	3419.68(8)
Z	4
ρ_{calc}g/cm^3	1.219
μ/mm^{-1}	1.615
F(000)	1328.0
Crystal size/mm^3	0.16 × 0.14 × 0.12
Radiation	Cu Kα (λ = 1.54184)
2Θ range for data collection/	6.62 to 153.358
Index ranges	$-16 \leq h \leq 16$, $-24 \leq k \leq 24$, $-17 \leq l \leq 16$
Reflections collected	37242
Independent reflections	12615 [R_{int} = 0.0185, R_{sigma} = 0.0147]
Data/restraints/parameters	12615/8/801
Goodness-of-fit on F^2	1.069
Final R indexes [I>=2σ (I)]	R_1 = 0.0332, wR_2 = 0.0962
Final R indexes [all data]	R_1 = 0.0345, wR_2 = 0.0972
Largest diff. peak/hole / eÅ^{-3}	0.24/-0.25
Flack/Hooft parameter	0.007(5)/0.000(2)

6.4 X-ray crystallography data of 9a

A single crystal of **9a** (CCDC 2330643) was obtained by slowly evaporating a mixture of EtOAC and Hexane solution (5:1) at ambient temperature. A colorless crystal of **9a** was mounted on a glass fiber at a random orientation. Thermal ellipsoids are drawn at 50% probability.

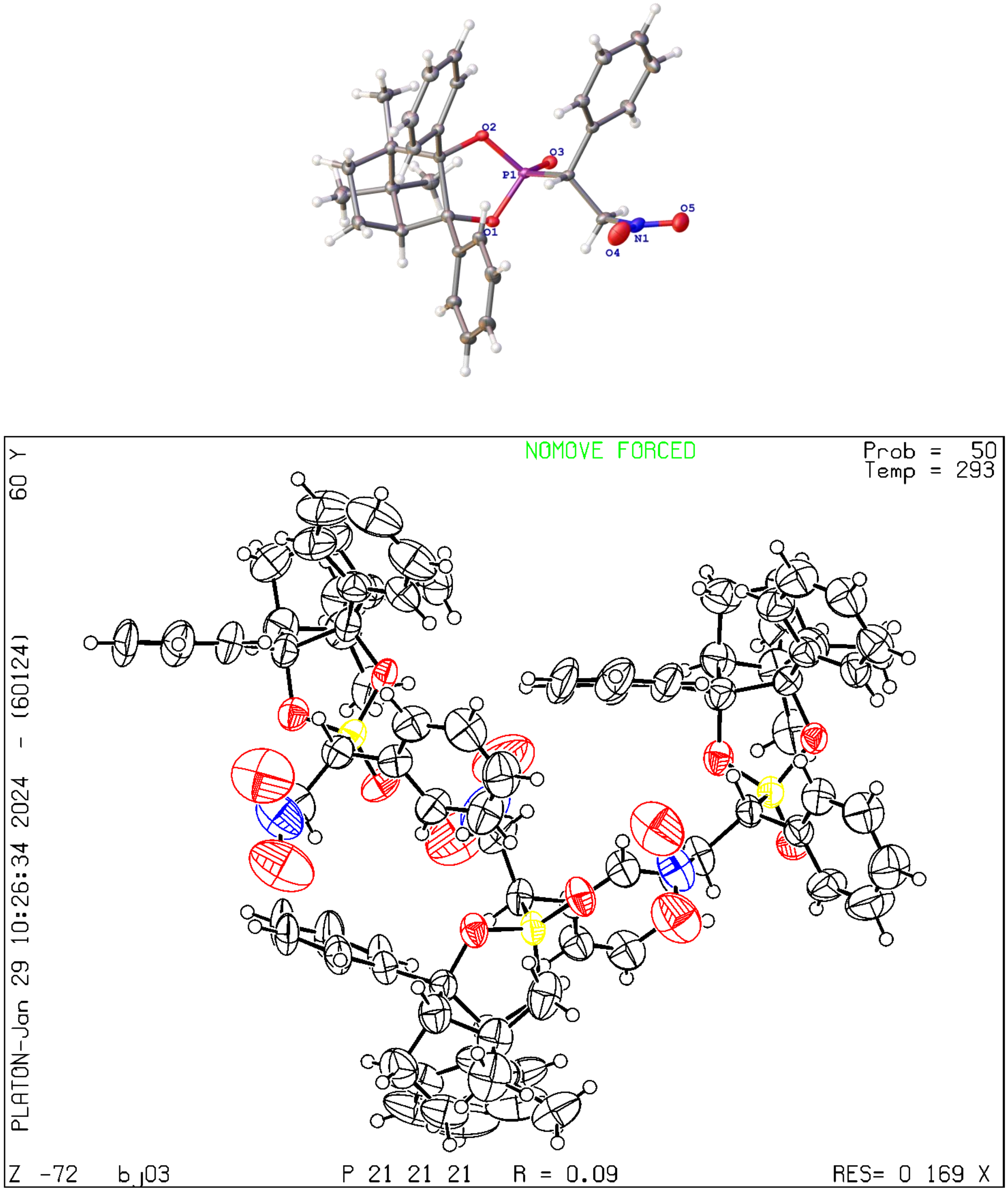

Figure S348. The ellipsoid plot of 9a and the molecular structure of 9a with the atomic numbering

Table S4 Crystal data and structure refinement for 9a after oxidation

**Crystal data and structure refinement for 9a.**	
Identification code	9a
Empirical formula	$C_{30}H_{32}NO_5P$
Formula weight	517.53
Temperature/K	293.15
Crystal system	orthorhombic
Space group	$P2_12_12_1$
a/Å	11.3599(2)
b/Å	29.0067(4)
c/Å	25.1663(6)
α/°	90
β/°	90
γ/°	90
Volume/Å^3	8292.6(3)
Z	12
ρ_{calc}g/cm^3	1.244
μ/mm^{-1}	1.200
F(000)	3288.0
Crystal size/mm^3	0.15 × 0.12 × 0.1
Radiation	Cu Kα (λ = 1.54184)
2Θ range for data collection/°	4.648 to 153.464
Index ranges	$-14 \leq h \leq 14$, $-35 \leq k \leq 27$, $-31 \leq l \leq 29$
Reflections collected	51998
Independent reflections	16681 [R_{int} = 0.0462, R_{sigma} = 0.0417]
Data/restraints/parameters	16681/7/1009
Goodness-of-fit on F^2	1.068
Final R indexes [I>=2σ (I)]	R_1 = 0.0862, wR_2 = 0.2543
Final R indexes [all data]	R_1 = 0.0957, wR_2 = 0.2624
Largest diff. peak/hole / e Å^{-3}	0.62/-0.38
Flack/Hooft parameter	0.054(8)/0.035(7)

7. Results of DFT calculations

The structures reported were optimized using the Gaussian 16 C.01 program[21]. The M062X functional[22] was used in all the DFT calculations. The basis set 6-31G**[23,24] was used for all the main group atoms. Frequency calculations were performed at the same level of theory to verify optimized structures as local minima or transition states and to obtain Gibbs free energy at the reaction temperature of 253.15 K. Transition state structures were confirmed to connect appropriate reactants and products by intrinsic reaction coordinate (IRC) calculations[25]. Structures were visualized using CYLview20[26]. The DFT-calculated Cartesian coordinates in the form of xyz were created in a separate file and submitted as one of the supplementary materials. This file can be opened using the Mercury software (downloaded at https://www.ccdc.cam.ac.uk/support-and-resources/downloads/), which enables the visualization of the 3D structure of all calculated transition states and intermediates.

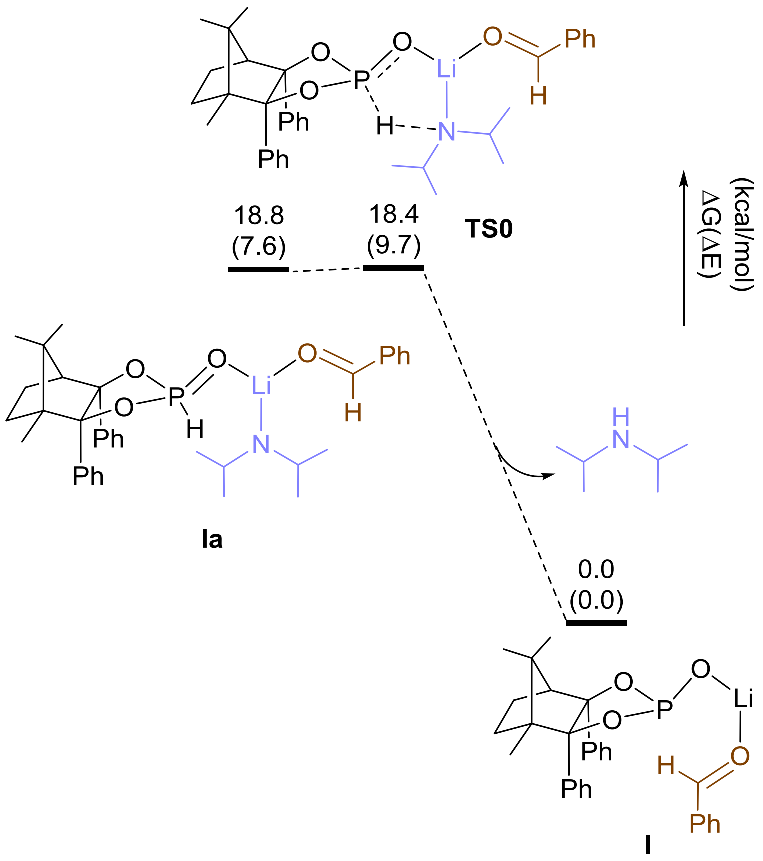

Fig.S349 Energy profile calculated for the deprotonation of CAMDOL-PHO with LDA.

8. References

[1] Y. Zhang, P. Zhao, S. Sun, Q. Wu, E. Shi, J. Xiao, Universal and divergent *P*-stereogenic building with camphor-derived 2,3-diols. *Commun. Chem.* **6**, 133 (2023).

[2] H. Guo, Q. Wu, S. Wang, H. Shu, E. Shi, Facile synthesis of *H*-phosphinates from $P(OR)_3$ or $ClP(OR)_2$ via SiO_2-promoted hydrolysis. *J. Org. Chem.* **89**, 8915-8923 (2024).

[3] T. K. Olszewski, Reactions of Chiral Phosphorous Acid Diamides: The Asymmetric Synthesis of Chiral alpha.-Hydroxy Phosphonamides, Phosphonates, and Phosphonic Acids**.** *J. Org. Chem* **60**, 931-940 (1995).

[4] K. Lee, T. R. Burke. Jr, CD45 protein-tyrosine phosphatase inhibitor development. *Curr. Top. Med. Chem.* **3**, 797-807 (2003).

[5] S. Fujimoto, J. Tsuda, N. Kawakami, H. Tanino, S. Shimohama, *myo*-Inositol monophosphatase in the brain has zinc ion-dependent tyrosine phosphatase activity. *Gen. Pharmacol.* **31**, 469-475 (1998).

[6] A. A. Puhl, R. J. Gruninger, R. Greiner, T. W. Janzen, S. C. Mosimann, L. B. Selinger, Kinetic and structural analysis of a bacterial protein tyrosine phosphatase-like myo-inositol polyphosphatase. *Protein Sci.* **16**, 1368-1378 (2007).

[7] A. Saito, H. Egami, T, K. Katsuki, L. B. Selinger, Synthesis of an Optically Active Al(salalen) Complex and Its Application to Catalytic Hydrophosphonylation of Aldehydes and Aldimines. *J. Am. Chem. Soc.* **129**, 1978-1986 (2007).

[8] T. K. Olszewski, M. Majewski, A highly convenient route to optically pure α-aminophosphonic acids. *Tetrahedron Lett* **36**, 4451-4454 (1995).

[9] P. P. Giannousis, P. A. Bartlett, A new efficient procedure for asymmetric synthesis of α-aminophosphonic acids via addition of lithiated bis(diethylamino)phosphine borane complex to enantiopure sulfinimines. *J. Med. Chem.* **13**, 2571-2576 (2002).

[10] P. P. Giannousis, P. A. Bartlett, Phosphorus amino acid analogs as inhibitors of leucine aminopeptidase. *J. Med. Chem.* **30**, 1603-1609 (1987).

[11] J. Grembecka, A. Mucha, T. Cierpicki, P. Kafarski, The most potent organophosphorus inhibitors of leucine aminopeptidase. Structure-based design, chemistry, and activity. *J. Med. Chem.* **46**, 2641-2655 (2003).

[12] L. A. Cates, V. S. Li, C. C., Yakshe, M. O. Fadeyi, T. H. Andree, E. W. Karbon, S. J. Enna, Phosphorus analogs of γ-aminobutyric acid, a new class of anticonvulsants. *J. Med. Chem.* **27**, 654-659 (1984).

[13] W. Froestl, S. J. Mickel, R. G. Hall, G. von Sprecher, D. Strub, P. A. Baumann, F. Brugger, C. Gentsch, J. Jaekel, Phosphinic acid analogs of GABA. 1. New potent and selective $GABA_B$ agonists. *J. Med. Chem.* **38**, 3297-3312 (1995).

[14] H. Huang, J. Palmas, J.Y. Kang, A Reagent-Controlled Phospha-Michael Addition Reaction of Nitroalkenes with Bifunctional N‑Heterocyclic Phosphine (NHP)-Thioureas. *J. Org. Chem.* **81**, 11932-11939 (2016).

[15] Q. Li, T. Chen, Q. Xu L.-B. Han, Rhodium- and Iridium-Catalyzed Asymmetric Addition of Optically Pure P-Chiral H-Phosphinates to Aldehydes Leading to Optically Active α-Hydroxyphosphinates. *Chem. – Eur. J.* **22**, 6213 (2016).

[16] P. Łyżwa, Double Asymmetric Induction in the Synthesis of Enantiomeric α-Aminophosphonic Acids Mediated by Sulfinimines *Heteroat. Chem*. **25**,15 (2014).

[17] Olszewski, T., K. Asymmetric synthesis of α-hydroxymethylphosphonates and phosphonic acids via hydrophosphonylation of aldehydes with chiral *H*-phosphonate. *Tetrahedron: Asymmetry* **26**, 393-399 (2015).

[18] F. A. Palacios, T. K. Olszewski, R. Greiner, J. Vicario, Diastereoselective hydrophosphonylation of imines using (*R, R*)-TADDOL phosphite. Asymmetric synthesis of α-aminophosphonic acid derivatives. *Org. Biomol. Chem.* **8**, 4255-4258 (2010).

[19] T. K. Olszewski, M. Majewski, Highly diastereoselective addition of chiral *H*-phosphonate to tert-butylsulfinyl aldimines: a convenient approach to (*R*)-a-aminophosphonic acids. *Tetrahedron: Asymmetry* **26**, 846-852 (2015).

[20] D. Enders, L. Tedeschi, D. Förster, Asymmetric Michael additions of a chiral phosphite to nitroalkenes and knoevenagel acceptors. *Synthesis.* **9**, 1447-1460 (2006).

[21] M. J. Frisch, et al. *Gaussian 16, Revision C.01*, Gaussian, Inc., Wallingford, CT, Wallingford, CT (2019).

[22] Y. Zhao, D. G. Truhlar, The M06 suite of density functionals for main group thermochemistry, thermochemical kinetics, noncovalent interactions, excited states, and transition elements: two new functionals and systematic testing of four M06-class functionals and 12 other functionals. *Theor. Chem. Acc.* **120**, 215-241 (2008).

[23] G. A. Petersson, M. A. Al‐Laham, A complete basis set model chemistry. II. Open‐shell systems and the total energies of the first‐row atoms. *J. Chem Phys.* **94**, 6081–6090 (1991).

[24] G. A. Petersson, et al. A complete basis set model chemistry. I. The total energies of closed‐shell atoms and hydrides of the first‐row elements. *J. Chem Phys.* **89**, 2193–2218 (1988).

[25] K. Fukui, The path of chemical reactions-the IRC approach. *Acc. Chem. Res.* **14**, 363–368 (1981).

[26] CYLview20; Legault, C. Y. Université de Sherbrooke, 2020 (http://www.cylview.org).
