# Supplementary material for: Stereoselective Pudovik reaction of aldehydes, aldimines, and nitroalkenes with CAMDOL-derived H-phosphonate
Source: Commun Chem. 2025 Nov 14;8:349. doi: 10.1038/s42004-025-01735-4 (PMC12618634; doi:10.1038/s42004-025-01735-4)
Supplement: Supplementary file 2 — Description of Additional Supplementary Files [file 42004_2025_1735_MOESM2_ESM.pdf]

Description of Additional Supplementary Files

File name: Supplementary Data 1
Description: Cif file of **1**

File name: Supplementary Data 2
Description: Cif file of **3a**

File name: Supplementary Data 3
Description: Cif file of **6a**

File name: Supplementary Data 4
Description: Cif file of **9a**

File name: Supplementary Data 5
Description: Gaussian output files (xyz file)

File name: Supplementary Data 6
Description: Raw data from density functional theory (DFT) calculations
