# Supplementary material for: Stereoselective Pudovik reaction of aldehydes, aldimines, and nitroalkenes with CAMDOL-derived H-phosphonate
Source: Commun Chem. 2025 Nov 14;8:349. doi: 10.1038/s42004-025-01735-4 (PMC12618634; doi:10.1038/s42004-025-01735-4)
Supplement: Supplementary file 3 — Supplementary Data 1 [file 42004_2025_1735_MOESM3_ESM.zip › Supplementary Data 3-the cif file of 1/LLINT晶体学数据.docx]

**LLINT_auto**

| **Table 1 Crystal data and structure refinement for LLINT_auto.** | |
| --- | --- |
| Identification code | LLINT_auto |
| Empirical formula | C_22_H_25_O_3_P |
| Formula weight | 368.39 |
| Temperature/K | 100.00(10) |
| Crystal system | monoclinic |
| Space group | P2_1_ |
| a/Å | 10.20020(10) |
| b/Å | 8.82840(10) |
| c/Å | 10.47620(10) |
| α/° | 90 |
| β/° | 93.8840(10) |
| γ/° | 90 |
| Volume/Å^3^ | 941.230(17) |
| Z | 2 |
| ρ_calc_g/cm^3^ | 1.300 |
| μ/mm^‑1^ | 1.442 |
| F(000) | 392.0 |
| Crystal size/mm^3^ | 0.14 × 0.1 × 0.08 |
| Radiation | Cu Kα (λ = 1.54184) |
| 2Θ range for data collection/° | 8.46 to 145.646 |
| Index ranges | -12 ≤ h ≤ 12, -9 ≤ k ≤ 10, -12 ≤ l ≤ 12 |
| Reflections collected | 11619 |
| Independent reflections | 3368 [R_int_ = 0.0290, R_sigma_ = 0.0160] |
| Data/restraints/parameters | 3368/1/242 |
| Goodness-of-fit on F^2^ | 1.007 |
| Final R indexes [I>=2σ (I)] | R_1_ = 0.0323, wR_2_ = 0.0867 |
| Final R indexes [all data] | R_1_ = 0.0323, wR_2_ = 0.0868 |
| Largest diff. peak/hole / e Å^-3^ | 0.26/-0.27 |
| Flack/Hooft parameter | -0.015(19)/-0.015(4) |

**Crystal structure determination of [LLINT_auto]**

**Crystal Data** for C_22_H_25_O_3_P (*M*=368.39 g/mol): monoclinic, space group P2_1_ (no. 4), *a* = 10.20020(10) Å, *b* = 8.82840(10) Å, *c* = 10.47620(10) Å, *β* = 93.8840(10)°, *V*= 941.230(17) Å^3^, *Z* = 2, *T* = 100.00(10) K, μ(Cu Kα) = 1.442 mm^-1^, *Dcalc* = 1.300 g/cm^3^, 11619 reflections measured (8.46° ≤ 2Θ ≤ 145.646°), 3368 unique (*R*_int_ = 0.0290, R_sigma_ = 0.0160) which were used in all calculations. The final *R*_1_ was 0.0323 (I > 2σ(I)) and *wR*_2_ was 0.0868 (all data).

**Refinement model description**

| **Table 2 Fractional Atomic Coordinates (×10^4^) and Equivalent Isotropic Displacement Parameters (Å^2^×10^3^) for LLINT_auto. U_eq_ is defined as 1/3 of the trace of the orthogonalised U_IJ_ tensor.** | | | | |
| --- | --- | --- | --- | --- |
| **Atom** | ***x*** | ***y*** | ***z*** | **U(eq)** |
| P1 | 2886.1(5) | 2843.5(6) | 8546.5(5) | 19.13(16) |
| O1 | 4040.3(15) | 4013.7(18) | 8832.8(14) | 18.3(3) |
| O2 | 1732.0(15) | 4016.0(18) | 8268.8(14) | 18.1(3) |
| O3 | 2705.4(17) | 1721(2) | 9538.1(17) | 27.3(4) |
| C1 | 4716(2) | 5574(3) | 7172(2) | 18.2(4) |
| C2 | 5876(2) | 6410(3) | 7333(2) | 21.3(5) |
| C3 | 6780(2) | 6403(3) | 6388(2) | 28.3(6) |
| C4 | 6529(3) | 5567(3) | 5281(2) | 31.4(6) |
| C5 | 5402(2) | 4689(3) | 5131(2) | 29.6(6) |
| C6 | 4511(2) | 4678(3) | 6079(2) | 22.4(5) |
| C7 | 1675(2) | 5723(3) | 6493(2) | 16.4(4) |
| C8 | 602(2) | 4875(3) | 6018(2) | 21.2(5) |
| C9 | 118(2) | 5006(3) | 4745(2) | 26.5(5) |
| C10 | 693(2) | 5995(3) | 3927(2) | 25.3(5) |
| C11 | 1752(2) | 6869(3) | 4388(2) | 22.1(5) |
| C12 | 2238(2) | 6726(3) | 5655(2) | 18.8(4) |
| C13 | 2189(2) | 5513(3) | 7885(2) | 15.2(4) |
| C14 | 3763(2) | 5510(3) | 8230(2) | 15.4(4) |
| C15 | 3899(2) | 6763(3) | 9249.6(19) | 16.2(4) |
| C16 | 2680(2) | 6619(3) | 10048(2) | 18.0(4) |
| C17 | 1681(2) | 6721(3) | 8837.4(19) | 15.8(4) |
| C18 | 226(2) | 6573(3) | 9046(2) | 23.9(5) |
| C19 | 2078(2) | 8281(3) | 8318(2) | 17.7(4) |
| C20 | 3608(2) | 8273(3) | 8534(2) | 18.0(5) |
| C21 | 2573(2) | 7979(3) | 10971(2) | 22.6(5) |
| C22 | 2582(2) | 5224(3) | 10924(2) | 22.5(5) |

| **Table 3 Anisotropic Displacement Parameters (Å^2^×10^3^) for LLINT_auto. The Anisotropic displacement factor exponent takes the form: -2π^2^[h^2^a*^2^U_11_+2hka*b*U_12_+…].** | | | | | | |
| --- | --- | --- | --- | --- | --- | --- |
| **Atom** | **U_11_** | **U_22_** | **U_33_** | **U_23_** | **U_13_** | **U_12_** |
| P1 | 24.0(3) | 11.5(3) | 21.6(3) | -0.2(2) | -0.06(19) | 0.5(2) |
| O1 | 20.8(7) | 12.6(8) | 21.0(7) | 2.5(6) | -1.3(6) | 2.7(6) |
| O2 | 19.8(7) | 12.1(8) | 22.3(7) | 2.6(6) | -0.3(6) | -3.0(6) |
| O3 | 34.1(9) | 15.2(8) | 32.3(9) | 4.1(7) | 0.2(7) | -1.1(8) |
| C1 | 17.1(10) | 16.8(12) | 20.8(10) | 2.2(9) | 1.6(8) | 4.6(8) |
| C2 | 18.3(10) | 21.0(13) | 24.4(11) | 2.9(9) | 0.5(8) | 1.5(8) |
| C3 | 19.1(10) | 30.4(16) | 36.1(13) | 8.1(11) | 5.8(9) | 1.8(10) |
| C4 | 29.3(12) | 36.6(16) | 29.7(12) | 8.4(12) | 12.9(10) | 12.9(11) |
| C5 | 30.2(12) | 34.8(16) | 24.4(12) | -3.5(11) | 6.1(10) | 12.7(11) |
| C6 | 21.0(10) | 22.6(13) | 23.7(11) | -3.2(9) | 2.0(8) | 4.3(9) |
| C7 | 15.9(9) | 15.8(12) | 17.2(10) | -1.9(8) | -1.0(7) | 2.9(8) |
| C8 | 22.0(10) | 20.3(12) | 21.1(11) | -0.4(9) | -0.5(8) | -1.8(9) |
| C9 | 22.6(10) | 33.7(15) | 22.3(12) | -4.7(10) | -4.0(9) | -5.1(10) |
| C10 | 27.5(12) | 31.9(14) | 15.8(10) | -2.9(9) | -3.1(8) | 4.0(10) |
| C11 | 25.9(11) | 22.2(13) | 18.4(10) | 1.8(9) | 2.2(8) | 2.3(10) |
| C12 | 19.9(9) | 17.6(11) | 18.6(10) | -1.2(9) | -0.2(7) | 0.3(9) |
| C13 | 16.2(10) | 11.6(11) | 17.6(10) | 0.6(8) | 1.0(8) | 0.0(8) |
| C14 | 17.0(10) | 10.8(11) | 18.1(10) | 2.0(8) | -0.6(8) | -0.1(7) |
| C15 | 19.4(9) | 13.2(10) | 15.5(9) | -0.1(8) | -1.3(7) | -0.4(9) |
| C16 | 21.7(10) | 15.5(11) | 16.7(9) | -0.4(9) | 0.7(8) | -0.4(9) |
| C17 | 19.4(9) | 12.3(10) | 15.9(9) | -1.4(8) | 2.0(7) | 1.8(9) |
| C18 | 20.1(10) | 24.3(13) | 27.6(11) | -1.6(10) | 4.3(8) | 1.7(9) |
| C19 | 22.4(10) | 12.9(11) | 17.8(10) | -0.9(8) | 0.4(8) | 2.4(8) |
| C20 | 21.1(10) | 13.1(12) | 19.6(10) | -1.3(8) | 1.7(8) | -0.9(8) |
| C21 | 31.2(11) | 19.1(12) | 17.7(10) | -2.2(10) | 4.4(8) | 0.3(10) |
| C22 | 31.8(12) | 19.3(12) | 16.7(10) | 2.0(9) | 3.4(9) | 0.3(10) |

| **Table 4 Bond Lengths for LLINT_auto.** | | | | | | |
| --- | --- | --- | --- | --- | --- | --- |
| **Atom** | **Atom** | **Length/Å** |  | **Atom** | **Atom** | **Length/Å** |
| P1 | O1 | 1.5797(16) |  | C8 | C9 | 1.395(3) |
| P1 | O2 | 1.5798(16) |  | C9 | C10 | 1.382(4) |
| P1 | O3 | 1.4563(18) |  | C10 | C11 | 1.387(3) |
| O1 | C14 | 1.483(3) |  | C11 | C12 | 1.391(3) |
| O2 | C13 | 1.466(3) |  | C13 | C14 | 1.622(3) |
| C1 | C2 | 1.395(3) |  | C13 | C17 | 1.572(3) |
| C1 | C6 | 1.396(3) |  | C14 | C15 | 1.538(3) |
| C1 | C14 | 1.523(3) |  | C15 | C16 | 1.550(3) |
| C2 | C3 | 1.398(3) |  | C15 | C20 | 1.548(3) |
| C3 | C4 | 1.384(4) |  | C16 | C17 | 1.575(3) |
| C4 | C5 | 1.387(4) |  | C16 | C21 | 1.549(3) |
| C5 | C6 | 1.390(3) |  | C16 | C22 | 1.543(3) |
| C7 | C8 | 1.391(3) |  | C17 | C18 | 1.520(3) |
| C7 | C12 | 1.398(3) |  | C17 | C19 | 1.544(3) |
| C7 | C13 | 1.527(3) |  | C19 | C20 | 1.561(3) |

| **Table 5 Bond Angles for LLINT_auto.** | | | | | | | | |
| --- | --- | --- | --- | --- | --- | --- | --- | --- |
| **Atom** | **Atom** | **Atom** | **Angle/˚** |  | **Atom** | **Atom** | **Atom** | **Angle/˚** |
| O2 | P1 | O1 | 98.22(9) |  | C7 | C13 | C17 | 114.66(17) |
| O3 | P1 | O1 | 115.99(10) |  | C17 | C13 | C14 | 102.92(16) |
| O3 | P1 | O2 | 116.64(10) |  | O1 | C14 | C1 | 103.33(17) |
| C14 | O1 | P1 | 112.56(12) |  | O1 | C14 | C13 | 104.67(16) |
| C13 | O2 | P1 | 113.22(12) |  | O1 | C14 | C15 | 109.82(16) |
| C2 | C1 | C14 | 120.6(2) |  | C1 | C14 | C13 | 120.57(17) |
| C6 | C1 | C2 | 118.5(2) |  | C1 | C14 | C15 | 116.55(18) |
| C6 | C1 | C14 | 120.6(2) |  | C15 | C14 | C13 | 101.22(16) |
| C1 | C2 | C3 | 120.5(2) |  | C14 | C15 | C16 | 106.18(18) |
| C4 | C3 | C2 | 120.2(2) |  | C14 | C15 | C20 | 106.13(16) |
| C3 | C4 | C5 | 119.8(2) |  | C20 | C15 | C16 | 101.35(17) |
| C4 | C5 | C6 | 120.0(2) |  | C15 | C16 | C17 | 93.42(15) |
| C5 | C6 | C1 | 120.9(2) |  | C15 | C16 | C21 | 111.50(18) |
| C8 | C7 | C12 | 117.59(19) |  | C21 | C16 | C17 | 112.88(19) |
| C8 | C7 | C13 | 119.2(2) |  | C22 | C16 | C15 | 118.23(19) |
| C12 | C7 | C13 | 123.22(19) |  | C22 | C16 | C17 | 117.37(19) |
| C7 | C8 | C9 | 121.1(2) |  | C22 | C16 | C21 | 103.72(17) |
| C10 | C9 | C8 | 120.5(2) |  | C13 | C17 | C16 | 104.44(16) |
| C9 | C10 | C11 | 119.3(2) |  | C18 | C17 | C13 | 113.56(18) |
| C10 | C11 | C12 | 120.0(2) |  | C18 | C17 | C16 | 117.66(18) |
| C11 | C12 | C7 | 121.5(2) |  | C18 | C17 | C19 | 114.12(19) |
| O2 | C13 | C7 | 106.04(17) |  | C19 | C17 | C13 | 106.02(16) |
| O2 | C13 | C14 | 105.41(16) |  | C19 | C17 | C16 | 99.41(17) |
| O2 | C13 | C17 | 108.24(16) |  | C17 | C19 | C20 | 103.19(16) |
| C7 | C13 | C14 | 118.90(17) |  | C15 | C20 | C19 | 103.37(17) |

| **Table 6 Torsion Angles for LLINT_auto.** | | | | | | | | | | |
| --- | --- | --- | --- | --- | --- | --- | --- | --- | --- | --- |
| **A** | **B** | **C** | **D** | **Angle/˚** |  | **A** | **B** | **C** | **D** | **Angle/˚** |
| P1 | O1 | C14 | C1 | -109.60(16) |  | C8 | C7 | C13 | C17 | -97.5(2) |
| P1 | O1 | C14 | C13 | 17.44(18) |  | C8 | C9 | C10 | C11 | -0.5(4) |
| P1 | O1 | C14 | C15 | 125.39(15) |  | C9 | C10 | C11 | C12 | 1.0(4) |
| P1 | O2 | C13 | C7 | 113.32(15) |  | C10 | C11 | C12 | C7 | -0.5(4) |
| P1 | O2 | C13 | C14 | -13.61(19) |  | C12 | C7 | C8 | C9 | 0.9(3) |
| P1 | O2 | C13 | C17 | -123.18(14) |  | C12 | C7 | C13 | O2 | -157.66(19) |
| O1 | P1 | O2 | C13 | 22.52(14) |  | C12 | C7 | C13 | C14 | -39.3(3) |
| O1 | C14 | C15 | C16 | -73.2(2) |  | C12 | C7 | C13 | C17 | 83.0(3) |
| O1 | C14 | C15 | C20 | 179.47(16) |  | C13 | C7 | C8 | C9 | -178.6(2) |
| O2 | P1 | O1 | C14 | -24.02(14) |  | C13 | C7 | C12 | C11 | 179.1(2) |
| O2 | C13 | C14 | O1 | -2.3(2) |  | C13 | C14 | C15 | C16 | 37.0(2) |
| O2 | C13 | C14 | C1 | 113.3(2) |  | C13 | C14 | C15 | C20 | -70.28(19) |
| O2 | C13 | C14 | C15 | -116.46(17) |  | C13 | C17 | C19 | C20 | -68.50(19) |
| O2 | C13 | C17 | C16 | 80.61(19) |  | C14 | C1 | C2 | C3 | 176.7(2) |
| O2 | C13 | C17 | C18 | -48.8(2) |  | C14 | C1 | C6 | C5 | -177.6(2) |
| O2 | C13 | C17 | C19 | -174.93(16) |  | C14 | C13 | C17 | C16 | -30.6(2) |
| O3 | P1 | O1 | C14 | -149.08(14) |  | C14 | C13 | C17 | C18 | -160.09(18) |
| O3 | P1 | O2 | C13 | 147.12(14) |  | C14 | C13 | C17 | C19 | 73.81(18) |
| C1 | C2 | C3 | C4 | 0.2(4) |  | C14 | C15 | C16 | C17 | -54.4(2) |
| C1 | C14 | C15 | C16 | 169.76(18) |  | C14 | C15 | C16 | C21 | -170.64(18) |
| C1 | C14 | C15 | C20 | 62.5(2) |  | C14 | C15 | C16 | C22 | 69.3(2) |
| C2 | C1 | C6 | C5 | -4.0(3) |  | C14 | C15 | C20 | C19 | 77.42(19) |
| C2 | C1 | C14 | O1 | -101.5(2) |  | C15 | C16 | C17 | C13 | 50.66(19) |
| C2 | C1 | C14 | C13 | 142.3(2) |  | C15 | C16 | C17 | C18 | 177.6(2) |
| C2 | C1 | C14 | C15 | 19.0(3) |  | C15 | C16 | C17 | C19 | -58.70(18) |
| C2 | C3 | C4 | C5 | -2.5(4) |  | C16 | C15 | C20 | C19 | -33.31(19) |
| C3 | C4 | C5 | C6 | 1.6(4) |  | C16 | C17 | C19 | C20 | 39.61(19) |
| C4 | C5 | C6 | C1 | 1.7(4) |  | C17 | C13 | C14 | O1 | 111.05(17) |
| C6 | C1 | C2 | C3 | 3.0(3) |  | C17 | C13 | C14 | C1 | -133.4(2) |
| C6 | C1 | C14 | O1 | 72.0(2) |  | C17 | C13 | C14 | C15 | -3.11(19) |
| C6 | C1 | C14 | C13 | -44.2(3) |  | C17 | C19 | C20 | C15 | -4.3(2) |
| C6 | C1 | C14 | C15 | -167.5(2) |  | C18 | C17 | C19 | C20 | 165.75(18) |
| C7 | C8 | C9 | C10 | -0.5(4) |  | C20 | C15 | C16 | C17 | 56.29(18) |
| C7 | C13 | C14 | O1 | -120.96(19) |  | C20 | C15 | C16 | C21 | -60.0(2) |
| C7 | C13 | C14 | C1 | -5.4(3) |  | C20 | C15 | C16 | C22 | 179.98(18) |
| C7 | C13 | C14 | C15 | 124.9(2) |  | C21 | C16 | C17 | C13 | 165.74(17) |
| C7 | C13 | C17 | C16 | -161.25(18) |  | C21 | C16 | C17 | C18 | -67.3(3) |
| C7 | C13 | C17 | C18 | 69.3(2) |  | C21 | C16 | C17 | C19 | 56.4(2) |
| C7 | C13 | C17 | C19 | -56.8(2) |  | C22 | C16 | C17 | C13 | -73.7(2) |
| C8 | C7 | C12 | C11 | -0.5(3) |  | C22 | C16 | C17 | C18 | 53.2(3) |
| C8 | C7 | C13 | O2 | 21.8(3) |  | C22 | C16 | C17 | C19 | 176.93(18) |
| C8 | C7 | C13 | C14 | 140.2(2) |  |  |  |  |  |  |

| **Table 7 Hydrogen Atom Coordinates (Å×10^4^) and Isotropic Displacement Parameters (Å^2^×10^3^) for LLINT_auto.** | | | | |
| --- | --- | --- | --- | --- |
| **Atom** | ***x*** | ***y*** | ***z*** | **U(eq)** |
| H1 | 3040(30) | 2040(30) | 7490(20) | 16(6) |
| H2 | 6053.4 | 6987.59 | 8089.94 | 26 |
| H3 | 7569.02 | 6972.73 | 6506.06 | 34 |
| H4 | 7127.04 | 5595.74 | 4624.23 | 38 |
| H5 | 5238.33 | 4094.14 | 4381.51 | 36 |
| H6 | 3753.49 | 4052.56 | 5980.78 | 27 |
| H8 | 190.73 | 4194.45 | 6569.13 | 25 |
| H9 | -612.8 | 4410.13 | 4437.84 | 32 |
| H10 | 367.09 | 6076.14 | 3058.61 | 30 |
| H11 | 2146.14 | 7564.68 | 3837.56 | 27 |
| H12 | 2968.4 | 7324.36 | 5957.36 | 23 |
| H15 | 4756.21 | 6750.12 | 9772.66 | 19 |
| H18A | 56.28 | 5575.88 | 9410.04 | 36 |
| H18B | -285.93 | 6684.43 | 8225.44 | 36 |
| H18C | -28.96 | 7364.93 | 9636.07 | 36 |
| H19A | 1689.14 | 9114.72 | 8799.21 | 21 |
| H19B | 1798.37 | 8385.11 | 7399.12 | 21 |
| H20A | 4026.62 | 8295.38 | 7709.3 | 22 |
| H20B | 3921.9 | 9149.28 | 9059.2 | 22 |
| H21A | 2726.42 | 8923.47 | 10512.5 | 34 |
| H21B | 3230.88 | 7874.1 | 11690.93 | 34 |
| H21C | 1692.36 | 7999.19 | 11291.55 | 34 |
| H22A | 1889 | 5391.13 | 11512.9 | 34 |
| H22B | 3423.05 | 5069.94 | 11415.17 | 34 |
| H22C | 2369.95 | 4326.46 | 10399.95 | 34 |

**Experimental**

Single crystals of C_22_H_25_O_3_P **[LLINT_auto]** were **[]**. A suitable crystal was selected and **[]** on a **ROD, Synergy Custom system, HyPix-Arc 150** diffractometer. The crystal was kept at 100.00(10) K during data collection. Using Olex2 [1], the structure was solved with the SHELXT [2] structure solution program using Intrinsic Phasing and refined with the SHELXL [3] refinement package using Least Squares minimisation.

1. Dolomanov, O.V., Bourhis, L.J., Gildea, R.J, Howard, J.A.K. & Puschmann, H. (2009), J. Appl. Cryst. 42, 339-341.
2. Sheldrick, G.M. (2015). Acta Cryst. A71, 3-8.
3. Sheldrick, G.M. (2015). Acta Cryst. C71, 3-8.

Number of restraints - 1, number of constraints - unknown.

Details:

1. Fixed Uiso
 At 1.2 times of:
 All C(H) groups, All C(H,H) groups
 At 1.5 times of:
 All C(H,H,H) groups
2.a Ternary CH refined with riding coordinates:
 C15(H15)
2.b Secondary CH2 refined with riding coordinates:
 C19(H19A,H19B), C20(H20A,H20B)
2.c Aromatic/amide H refined with riding coordinates:
 C2(H2), C3(H3), C4(H4), C5(H5), C6(H6), C8(H8), C9(H9), C10(H10), C11(H11),
 C12(H12)
2.d Idealised Me refined as rotating group:
 C18(H18A,H18B,H18C), C21(H21A,H21B,H21C), C22(H22A,H22B,H22C)

This report has been created with Olex2, compiled on 2022.04.07 svn.rca3783a0 for OlexSys. Please [let us know](mailto:support@olex2.org?subject=Olex2%20Report) if there are any errors or if you would like to have additional features.
