# Supplementary material for: Stereoselective Pudovik reaction of aldehydes, aldimines, and nitroalkenes with CAMDOL-derived H-phosphonate
Source: Commun Chem. 2025 Nov 14;8:349. doi: 10.1038/s42004-025-01735-4 (PMC12618634; doi:10.1038/s42004-025-01735-4)

checkCIF/PLATON report

Structure factors have been supplied for datablock(s) llint_auto

THIS REPORT IS FOR GUIDANCE ONLY. IF USED AS PART OF A REVIEW PROCEDURE FOR PUBLICATION, IT SHOULD NOT REPLACE THE EXPERTISE OF AN EXPERIENCED CRYSTALLOGRAPHIC REFEREE.

No syntax errors found. CIF dictionary Interpreting this report

Datablock: llint_auto

Bond precision:	C-C = 0.0031 A		Wavelength=1.54184
Cell:	a=10.2002(1)	b=8.8284(1)	c=10.4762(1)
	alpha=90	beta=93.884(1)	gamma=90
Temperature:	100 K		

	Calculated	Reported
Volume	941.230(17)	941.230(17)
Space group	P 21	P 1 21 1
Hall group	P 2yb	P 2yb
Moiety formula	C22 H25 O3 P	C22 H25 O3 P
Sum formula	C22 H25 O3 P	C22 H25 O3 P
Mr	368.39	368.39
Dx,g cm-3	1.300	1.300
Z	2	2
Mu (mm-1)	1.442	1.442
F000	392.0	392.0
F000’	393.64	
h,k,lmax	12,10,12	12,10,12
Nref	3755[2005]	3368
Tmin,Tmax	0.841,0.891	0.615,1.000
Tmin’	0.817	

Correction method= # Reported T Limits: Tmin=0.615 Tmax=1.000
AbsCorr = MULTI-SCAN

Data completeness= 1.68/0.90 Theta(max)= 72.823

R(reflections)= 0.0323(3356) wR2(reflections)= 0.0868(3368)

S = 1.007 Npar= 242

The following ALERTS were generated. Each ALERT has the format
test-name_ALERT_alert-type_alert-level.
Click on the hyperlinks for more details of the test.

Alert level C

```
PLAT911_ALERT_3_C Missing FCF Refl Between Thmin & STh/L=    0.600          4 Report
               -1  0  1,  12  0  1,  -1  0  2, -12  0  3,
```

Alert level G

```
PLAT143_ALERT_4_G s.u. on c - Axis Small or Missing .............    0.00010 Ang.
PLAT791_ALERT_4_G Model has Chirality at C13        (Sohncke SpGr)          S Verify
PLAT791_ALERT_4_G Model has Chirality at C14        (Sohncke SpGr)          S Verify
PLAT791_ALERT_4_G Model has Chirality at C15        (Sohncke SpGr)          S Verify
PLAT791_ALERT_4_G Model has Chirality at C17        (Sohncke SpGr)          R Verify
PLAT912_ALERT_4_G Missing # of FCF Reflections Above STh/L=  0.600         38 Note
PLAT913_ALERT_3_G Missing # of Very Strong Reflections in FCF ....          1 Note
               -1  0  1,
PLAT933_ALERT_2_G Number of HKL-OMIT Records in Embedded .res File          1 Note
                2 -9  3,
PLAT969_ALERT_5_G The 'Henn et al.' R-Factor-gap value ..........      6.616 Note
              Predicted wR2: Based on SigI**2  1.31 or SHELX Weight  8.62
PLAT978_ALERT_2_G Number C-C Bonds with Positive Residual Density.          6 Info
```

0 **ALERT level A** = Most likely a serious problem - resolve or explain
0 **ALERT level B** = A potentially serious problem, consider carefully
1 **ALERT level C** = Check. Ensure it is not caused by an omission or oversight
10 **ALERT level G** = General information/check it is not something unexpected

0 ALERT type 1 CIF construction/syntax error, inconsistent or missing data
2 ALERT type 2 Indicator that the structure model may be wrong or deficient
2 ALERT type 3 Indicator that the structure quality may be low
6 ALERT type 4 Improvement, methodology, query or suggestion
1 ALERT type 5 Informative message, check

It is advisable to attempt to resolve as many as possible of the alerts in all categories. Often the minor alerts point to easily fixed oversights, errors and omissions in your CIF or refinement strategy, so attention to these fine details can be worthwhile. In order to resolve some of the more serious problems it may be necessary to carry out additional measurements or structure refinements. However, the purpose of your study may justify the reported deviations and the more serious of these should normally be commented upon in the discussion or experimental section of a paper or in the "special_details" fields of the CIF. checkCIF was carefully designed to identify outliers and unusual parameters, but every test has its limitations and alerts that are not important in a particular case may appear. Conversely, the absence of alerts does not guarantee there are no aspects of the results needing attention. It is up to the individual to critically assess their own results and, if necessary, seek expert advice.

Publication of your CIF in IUCr journals

A basic structural check has been run on your CIF. These basic checks will be run on all CIFs submitted for publication in IUCr journals (*Acta Crystallographica*, *Journal of Applied Crystallography*, *Journal of Synchrotron Radiation*); however, if you intend to submit to *Acta Crystallographica Section C* or *E* or *IUCrData*, you should make sure that full publication checks are run on the final version of your CIF prior to submission.

Publication of your CIF in other journals

Please refer to the *Notes for Authors* of the relevant journal for any special instructions relating to CIF submission.

PLATON version of 22/08/2024; check.def file version of 21/08/2024

Datablock llint_auto - ellipsoid plot

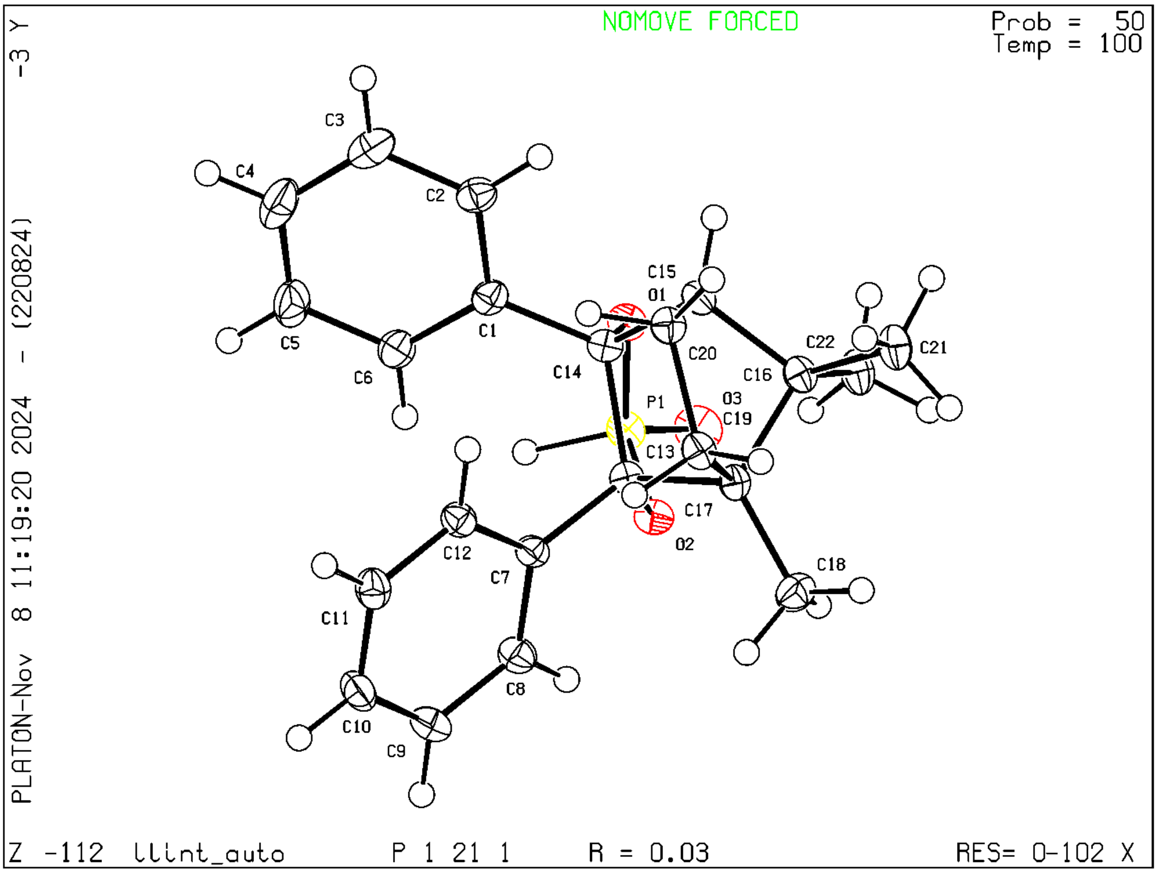

Supplement: Supplementary file 3 — Supplementary Data 1 [file 42004_2025_1735_MOESM3_ESM.zip › Supplementary Data 3-the cif file of 1/checkcif.pdf]
