# Supplementary figures and images for: Stereoselective Pudovik reaction of aldehydes, aldimines, and nitroalkenes with CAMDOL-derived H-phosphonate
Source: Commun Chem. 2025 Nov 14;8:349. doi: 10.1038/s42004-025-01735-4 (PMC12618634; doi:10.1038/s42004-025-01735-4)

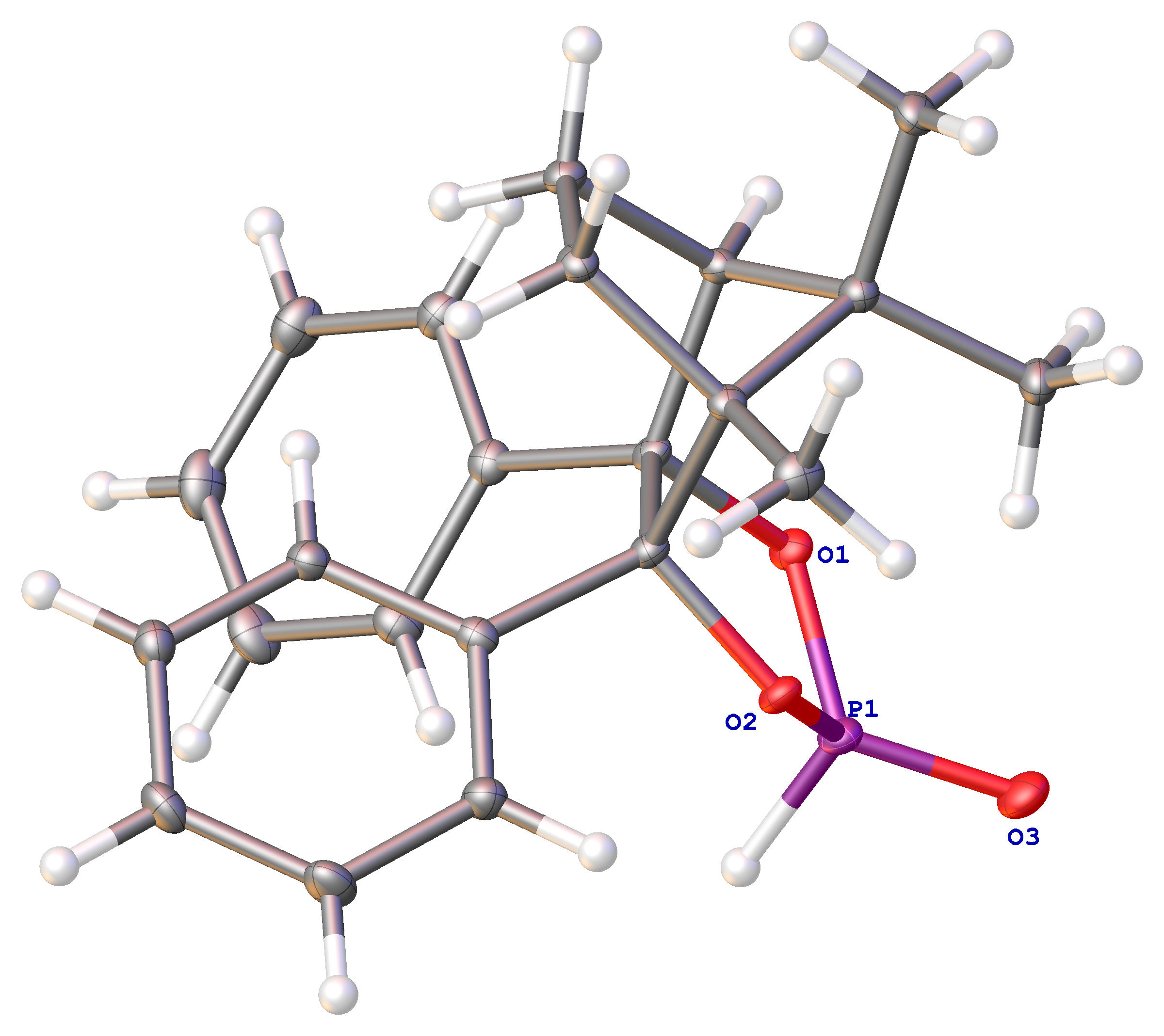

Supplement: Supplementary file 3 — Supplementary Data 1 [file 42004_2025_1735_MOESM3_ESM.zip › Supplementary Data 3-the cif file of 1/LLINT_auto.png]

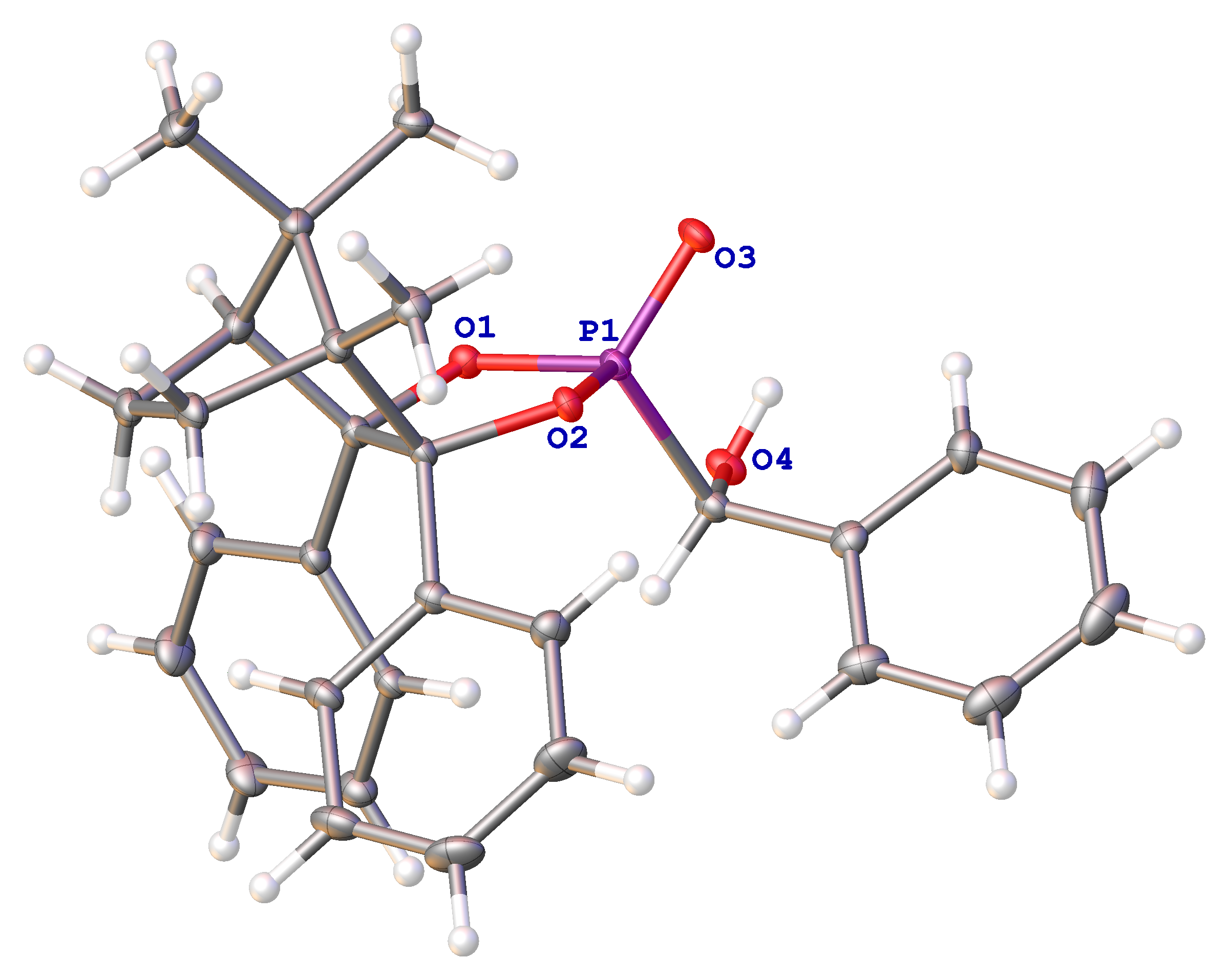

Supplement: Supplementary file 4 — Supplementary Data 2 [file 42004_2025_1735_MOESM4_ESM.zip › Supplementary Data 4-the cif file of 3a/bj01-7.png]

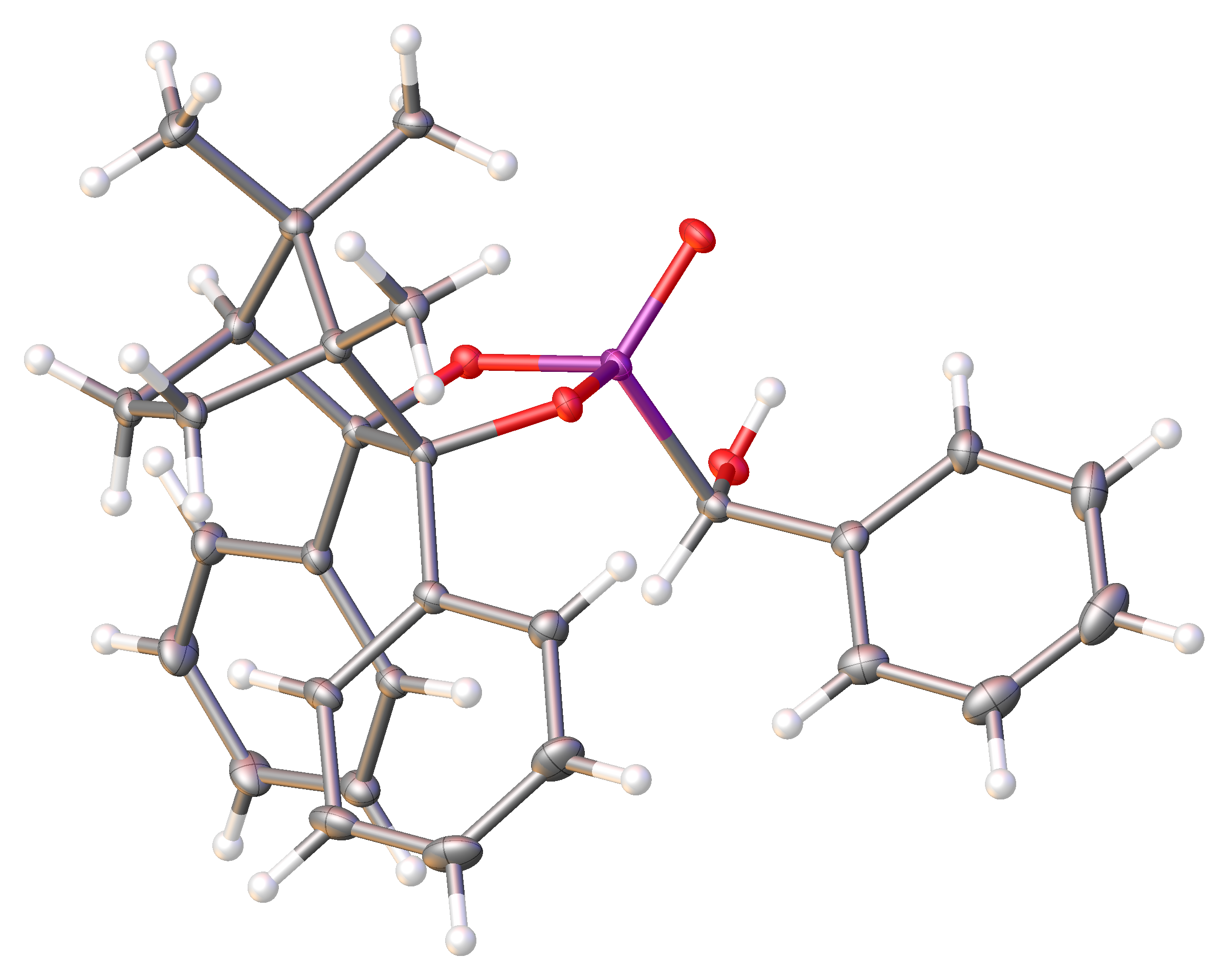

Supplement: Supplementary file 4 — Supplementary Data 2 [file 42004_2025_1735_MOESM4_ESM.zip › Supplementary Data 4-the cif file of 3a/bj01.png]

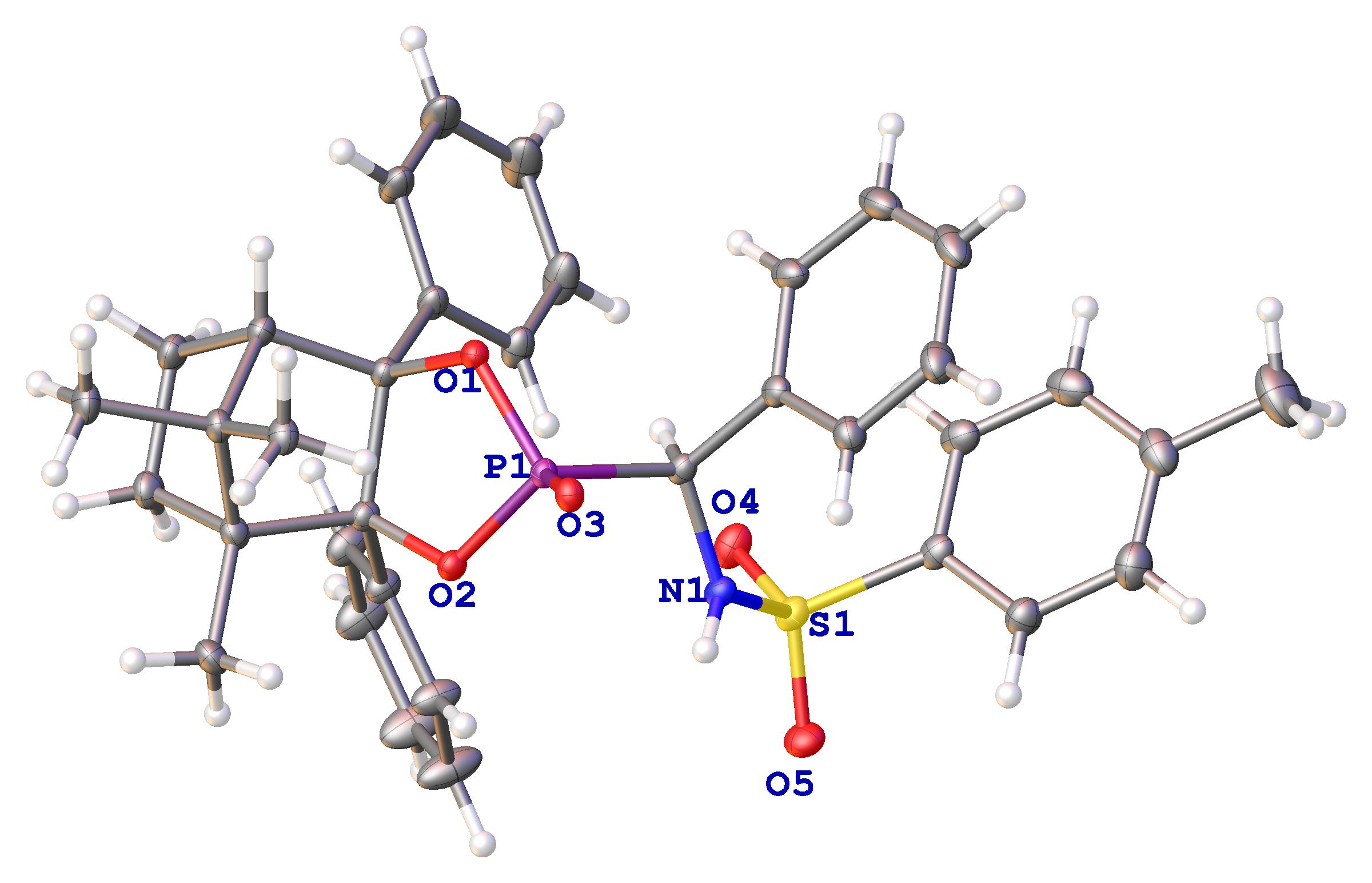

Supplement: Supplementary file 5 — Supplementary Data 3 [file 42004_2025_1735_MOESM5_ESM.zip › Supplementary Data 5-the cif file of 6a/bj02.png]

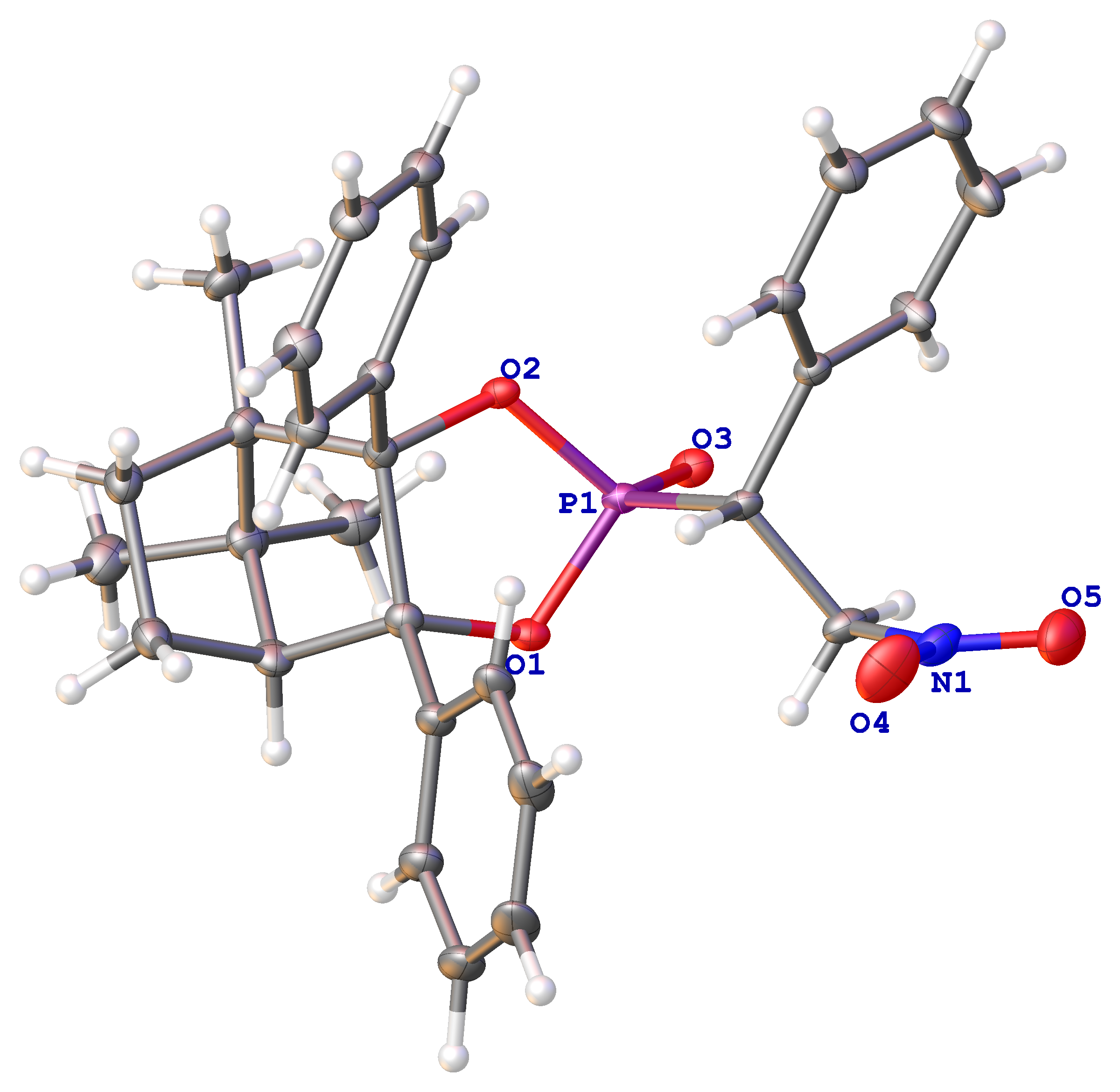

Supplement: Supplementary file 6 — Supplementary Data 4 [file 42004_2025_1735_MOESM6_ESM.zip › Supplementary Data 6-the cif file of 9a/BJ03-0.png]

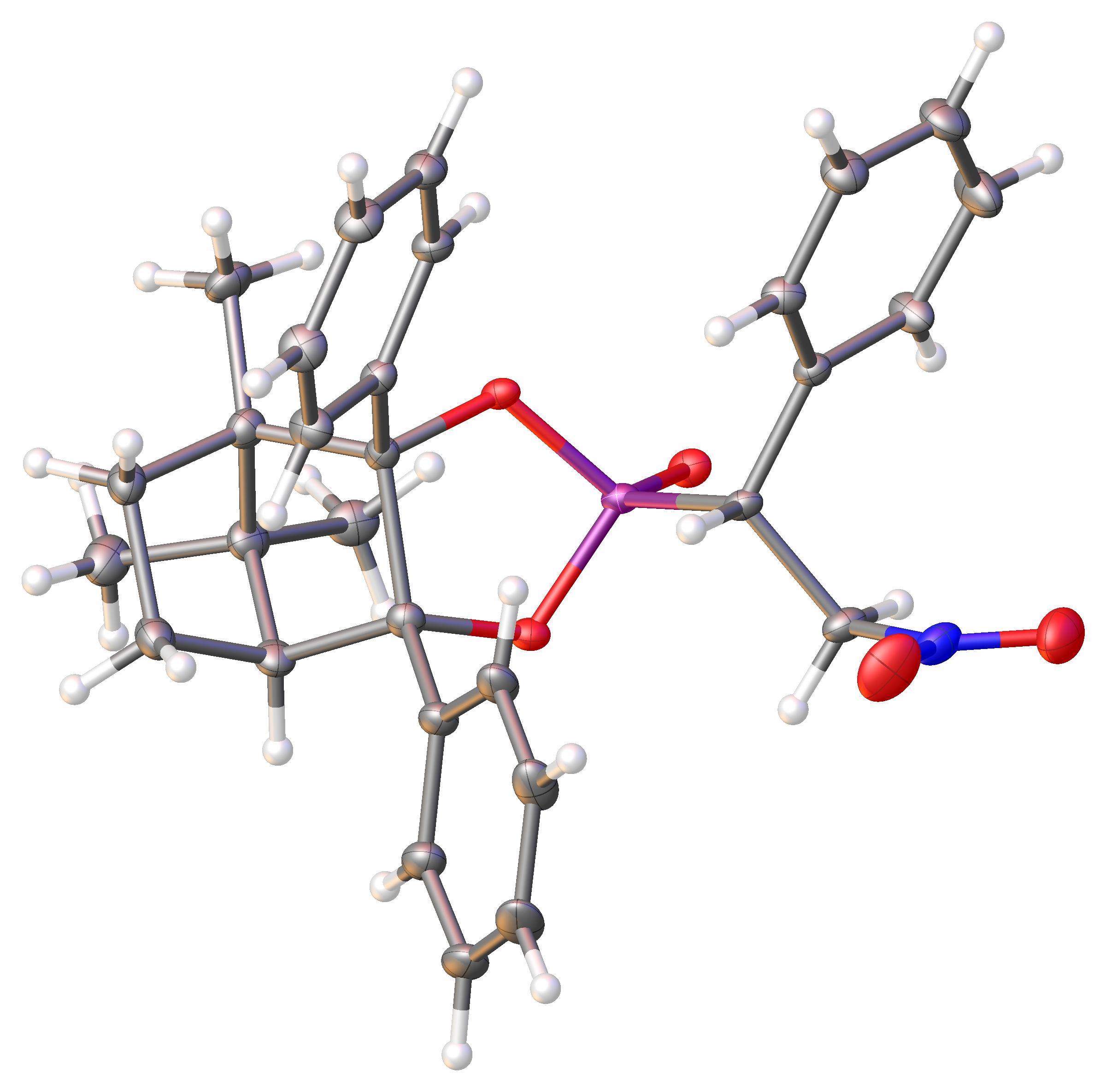

Supplement: Supplementary file 6 — Supplementary Data 4 [file 42004_2025_1735_MOESM6_ESM.zip › Supplementary Data 6-the cif file of 9a/BJ03.png]
