# Supplementary material for: Stereoselective Pudovik reaction of aldehydes, aldimines, and nitroalkenes with CAMDOL-derived H-phosphonate
Source: Commun Chem. 2025 Nov 14;8:349. doi: 10.1038/s42004-025-01735-4 (PMC12618634; doi:10.1038/s42004-025-01735-4)
Supplement: Supplementary file 4 — Supplementary Data 2 [file 42004_2025_1735_MOESM4_ESM.zip › Supplementary Data 4-the cif file of 3a/bj01-晶体学数据.docx]

**bj01**

| **Table 1 Crystal data and structure refinement for bj01.** | |
| --- | --- |
| Identification code | bj01 |
| Empirical formula | C_29_H_31_O_4_P |
| Formula weight | 474.51 |
| Temperature/K | 293.15 |
| Crystal system | tetragonal |
| Space group | P4_3_ |
| a/Å | 16.72503(14) |
| b/Å | 16.72503(14) |
| c/Å | 20.1957(2) |
| α/° | 90 |
| β/° | 90 |
| γ/° | 90 |
| Volume/Å^3^ | 5649.27(12) |
| Z | 8 |
| ρ_calc_g/cm^3^ | 1.169 |
| μ/mm^‑1^ | 1.148 |
| F(000) | 2116.0 |
| Crystal size/mm^3^ | 0.16 × 0.13 × 0.11 |
| Radiation | Cu Kα (λ = 1.54184) |
| 2Θ range for data collection/° | 5.284 to 152.99 |
| Index ranges | -19 ≤ h ≤ 16, -20 ≤ k ≤ 21, -25 ≤ l ≤ 25 |
| Reflections collected | 36222 |
| Independent reflections | 11316 [R_int_ = 0.0301, R_sigma_ = 0.0261] |
| Data/restraints/parameters | 11316/1/657 |
| Goodness-of-fit on F^2^ | 1.301 |
| Final R indexes [I>=2σ (I)] | R_1_ = 0.1007, wR_2_ = 0.2730 |
| Final R indexes [all data] | R_1_ = 0.1025, wR_2_ = 0.2753 |
| Largest diff. peak/hole / e Å^-3^ | 0.86/-0.41 |
| Flack/Hooft parameter | 0.02(4)/0.015(5) |

**Crystal structure determination of [bj01]**

**Crystal Data** for C_29_H_31_O_4_P (*M*=497.03 g/mol): tetragonal, space group P4_3_ (no. 78), *a* = 16.72503(14) Å, *c* = 20.1957(2) Å, *V*= 5649.27(12) Å^3^, *Z* = 8, *T* = 293.15 K, μ(Cu Kα) = 1.148 mm^-1^, *Dcalc* = 1.169 g/cm^3^, 36222 reflections measured (5.284° ≤ 2Θ ≤ 152.99°), 11316 unique (*R*_int_ = 0.0301, R_sigma_ = 0.0261) which were used in all calculations. The final *R*_1_ was 0.1007 (I > 2σ(I)) and *wR*_2_ was 0.2753 (all data).

**Refinement model description**

| **Table 2 Fractional Atomic Coordinates (×10^4^) and Equivalent Isotropic Displacement Parameters (Å^2^×10^3^) for bj01. U_eq_ is defined as 1/3 of the trace of the orthogonalised U_IJ_ tensor.** | | | | |
| --- | --- | --- | --- | --- |
| **Atom** | ***x*** | ***y*** | ***z*** | **U(eq)** |
| P1 | 6657.0(4) | 586.1(5) | 5206.5(3) | 43.59(16) |
| O1 | 6690.1(12) | 1286.2(13) | 5728.0(11) | 46.3(5) |
| O2 | 7569.3(12) | 323.6(13) | 5253.0(10) | 44.5(5) |
| O3 | 6396.4(14) | 801.6(17) | 4537.1(12) | 57.4(6) |
| O4 | 5223.1(14) | 185.7(16) | 5577.3(11) | 55.6(6) |
| C1 | 9192(2) | 851(3) | 5008.2(19) | 59.8(9) |
| C2 | 8701.6(18) | 1225(2) | 5556.7(16) | 47.4(7) |
| C3 | 9179(2) | 1479(2) | 6177.1(19) | 58.8(9) |
| C4 | 8580(2) | 2009(2) | 6564.2(18) | 58.5(9) |
| C5 | 7855(2) | 2070.9(19) | 6088.5(17) | 50.6(7) |
| C6 | 8858(3) | 2759(2) | 5326(2) | 71.1(11) |
| C7 | 8275(2) | 2042(2) | 5404.5(17) | 52.8(8) |
| C8 | 7754(2) | 2080(2) | 4774.9(19) | 60.2(9) |
| C9 | 7400.7(17) | 1271.8(17) | 6165.2(14) | 41.8(6) |
| C10 | 8010.7(16) | 657.7(17) | 5816.6(13) | 39.2(6) |
| C11 | 8332.4(17) | -68(2) | 6195.1(16) | 46.8(7) |
| C12 | 8584(2) | -31(2) | 6857.7(17) | 56.4(9) |
| C13 | 8898(3) | -700(3) | 7167(2) | 78.9(12) |
| C14 | 8971(3) | -1403(3) | 6827(3) | 93.1(14) |
| C15 | 8751(3) | -1448(3) | 6176(4) | 89.1(16) |
| C16 | 8421(2) | -779(2) | 5857(2) | 61.8(10) |
| C17 | 7052.3(18) | 1101.1(19) | 6850.8(15) | 46.5(7) |
| C18 | 6857.1(19) | 333(2) | 7068.0(15) | 48.4(7) |
| C19 | 6486(2) | 207(3) | 7669.9(19) | 61.4(9) |
| C20 | 6287(3) | 853(3) | 8074(2) | 76.0(12) |
| C21 | 6459(3) | 1610(3) | 7859(2) | 80.9(12) |
| C22 | 6836(3) | 1740(2) | 7260(2) | 64.7(10) |
| C23 | 5983.6(18) | -181(2) | 5539.6(16) | 49.0(7) |
| C24 | 5994.1(19) | -953(2) | 5161(2) | 54.3(8) |
| C25 | 6333(2) | -1628(3) | 5448(3) | 73.5(12) |
| C26 | 6296(3) | -2359(3) | 5117(4) | 97.2(17) |
| C27 | 5934(3) | -2418(3) | 4516(4) | 108.1(19) |
| C28 | 5605(3) | -1753(3) | 4237(3) | 90.9(15) |
| C29 | 5629(2) | -1020(3) | 4543(2) | 65.8(10) |
| P2 | 4140.6(4) | 1444.0(4) | 4273.1(3) | 39.93(15) |
| O5 | 5551.9(15) | 2141.8(15) | 4132.2(13) | 57.2(6) |
| O6 | 4440.2(13) | 647.2(13) | 4440.0(12) | 49.1(5) |
| O7 | 3914.0(12) | 1986.5(13) | 4887.1(10) | 45.1(5) |
| O8 | 3295.6(12) | 1461.8(12) | 3916.9(10) | 41.9(5) |
| C30 | 5017.2(17) | 1526(2) | 3115.8(15) | 45.3(7) |
| C31 | 4766(2) | 1839(3) | 2515.7(17) | 61.8(10) |
| C32 | 4924(3) | 1419(4) | 1928.1(18) | 81.1(14) |
| C33 | 5316(3) | 727(3) | 1929(2) | 83.4(13) |
| C34 | 5574(2) | 402(3) | 2523(2) | 72.0(11) |
| C35 | 5428.6(19) | 797(2) | 3120.2(17) | 52.8(8) |
| C36 | 4860.1(18) | 1972.6(19) | 3751.0(15) | 45.2(7) |
| C37 | 3464.6(19) | 3318(2) | 4854.0(18) | 52.5(8) |
| C38 | 3811(3) | 3730(2) | 4319(2) | 65.9(10) |
| C39 | 4129(3) | 4485(3) | 4405(3) | 86.6(15) |
| C40 | 4127(3) | 4832(3) | 5029(4) | 98.7(17) |
| C41 | 3801(3) | 4428(3) | 5564(2) | 80.4(11) |
| C42 | 3469(2) | 3686(2) | 5473(2) | 64.0(9) |
| C43 | 3172.5(17) | 2452.8(18) | 4799.6(14) | 42.6(6) |
| C44 | 2555.1(19) | 2214(2) | 5336.1(16) | 51.2(8) |
| C45 | 1825(2) | 2775(2) | 5220(2) | 62.2(9) |
| C46 | 1397(2) | 2379(3) | 4623(2) | 62.0(10) |
| C47 | 1971.8(19) | 1689(2) | 4429.1(16) | 49.8(8) |
| C48 | 1483(2) | 1158(3) | 5565(2) | 73.3(11) |
| C49 | 2218.0(19) | 1385(2) | 5133.0(17) | 52.5(8) |
| C50 | 2770(2) | 659(2) | 5187.9(19) | 59.2(9) |
| C51 | 1603(2) | 1090(3) | 3954(2) | 65.3(10) |
| C52 | 2751.8(17) | 2095.4(18) | 4144.6(14) | 42.0(6) |
| C53 | 2597(2) | 2617.6(19) | 3539.4(16) | 49.4(7) |
| C54 | 2725(2) | 2302(2) | 2914.3(17) | 56.6(9) |
| C55 | 2568(3) | 2747(3) | 2344(2) | 77.4(13) |
| C56 | 2265(4) | 3503(3) | 2387(3) | 95.9(17) |
| C57 | 2132(4) | 3826(3) | 3014(3) | 89.6(15) |
| C58 | 2297(3) | 3401(2) | 3573(2) | 67.8(10) |
| O1W | 5517(3) | 3205(4) | 6384(4) | 168(2) |
| O2W | 4351(4) | 1946(6) | 6705(4) | 176(3) |
| O3W | 6249(8) | 3790(8) | 5326(11) | 191(7) |

| **Table 3 Anisotropic Displacement Parameters (Å^2^×10^3^) for bj01. The Anisotropic displacement factor exponent takes the form: -2π^2^[h^2^a*^2^U_11_+2hka*b*U_12_+…].** | | | | | | |
| --- | --- | --- | --- | --- | --- | --- |
| **Atom** | **U_11_** | **U_22_** | **U_33_** | **U_23_** | **U_13_** | **U_12_** |
| P1 | 40.2(3) | 54.1(3) | 36.5(3) | 3.4(3) | -7.0(3) | -4.0(3) |
| O1 | 41.9(9) | 50.8(10) | 46.3(10) | 1.1(9) | -4.9(8) | 3.7(8) |
| O2 | 42.6(9) | 54.8(10) | 36.1(8) | -6.3(8) | -4.5(8) | -0.5(8) |
| O3 | 54.7(11) | 77.7(15) | 39.9(10) | 9.3(10) | -13.7(9) | -9.7(10) |
| O4 | 47.9(10) | 77.6(14) | 41.5(9) | 4.4(11) | 2.8(9) | 5.3(10) |
| C1 | 47.7(15) | 76(2) | 56.2(17) | 0.0(16) | 11.7(14) | -4.3(15) |
| C2 | 43.0(13) | 56.0(15) | 43.3(13) | -0.6(13) | -1.0(11) | -8.6(12) |
| C3 | 47.5(14) | 73.4(19) | 55.4(16) | 1.0(16) | -8.1(13) | -16.7(14) |
| C4 | 60.7(16) | 66.5(17) | 48.5(15) | -7.0(15) | -2.8(14) | -21.5(14) |
| C5 | 56.6(15) | 45.9(13) | 49.4(14) | -7.2(12) | 4.7(13) | -6.3(12) |
| C6 | 78(2) | 62.2(19) | 73(2) | 6.2(18) | 7(2) | -20.4(17) |
| C7 | 59.3(16) | 54.7(16) | 44.4(13) | 2.5(13) | 2.8(13) | -9.8(13) |
| C8 | 64.9(18) | 64.5(18) | 51.2(15) | 16.0(15) | -1.0(15) | -5.6(15) |
| C9 | 41.3(12) | 46.4(13) | 37.6(12) | -3.5(11) | -1.5(10) | -1.0(10) |
| C10 | 37.1(11) | 47.9(12) | 32.5(10) | -1.6(10) | -2.9(10) | -2.1(10) |
| C11 | 37.4(12) | 57.0(15) | 45.8(14) | 5.1(13) | -0.5(11) | 2.7(11) |
| C12 | 45.7(14) | 77(2) | 46.5(14) | 9.9(15) | -5.8(13) | -1.5(14) |
| C13 | 56.2(18) | 109(3) | 71(2) | 39.9(19) | -10.5(17) | 0(2) |
| C14 | 68(2) | 90(2) | 122(3) | 52(2) | -5(2) | 18.9(19) |
| C15 | 78(2) | 60.0(19) | 129(4) | 16(2) | -1(3) | 20.7(18) |
| C16 | 59.7(17) | 56.5(17) | 69(2) | -0.8(17) | 0.0(16) | 8.7(14) |
| C17 | 44.7(13) | 55.2(14) | 39.5(13) | -5.4(12) | 0.9(11) | -4.1(12) |
| C18 | 45.8(13) | 61.8(16) | 37.7(12) | 0.4(13) | -1.0(11) | -5.7(13) |
| C19 | 54.3(16) | 78(2) | 51.8(16) | 7.7(17) | 3.4(15) | -10.1(15) |
| C20 | 70(2) | 107(3) | 51.1(17) | -8.3(19) | 22.1(16) | -14(2) |
| C21 | 87(2) | 94(3) | 62.0(19) | -27.8(19) | 26.9(18) | -11(2) |
| C22 | 74(2) | 64.4(18) | 56.3(17) | -14.8(16) | 14.8(16) | -5.6(17) |
| C23 | 41.8(13) | 62.1(16) | 43.2(13) | 6.7(13) | -3.0(11) | -2.9(12) |
| C24 | 40.9(13) | 58.4(16) | 63.7(17) | 3.4(15) | 7.2(14) | -7.3(12) |
| C25 | 53.1(17) | 76(2) | 92(3) | 17(2) | 16.0(18) | 10.1(16) |
| C26 | 80(2) | 67(2) | 144(5) | 10(3) | 38(3) | 17(2) |
| C27 | 90(3) | 66(2) | 168(5) | -33(3) | 50(3) | -7(2) |
| C28 | 68(2) | 96(3) | 108(3) | -44(2) | 12(2) | -17(2) |
| C29 | 59.2(18) | 65.3(19) | 73(2) | -11.3(18) | -2.5(17) | -6.7(16) |
| P2 | 40.5(3) | 44.5(3) | 34.8(3) | -2.0(3) | 1.2(2) | 3.5(3) |
| O5 | 57.1(11) | 57.5(11) | 57.0(12) | -8.4(10) | -3.1(11) | -12.6(9) |
| O6 | 48.9(10) | 51.5(10) | 47.0(10) | 3.6(9) | 0.8(9) | 7.3(9) |
| O7 | 43.3(9) | 56.1(10) | 35.9(8) | -7.2(8) | -1.0(8) | 2.7(8) |
| O8 | 43.3(9) | 42.8(9) | 39.6(8) | -7.3(8) | -2.7(8) | 6.9(7) |
| C30 | 36.9(11) | 59.5(15) | 39.6(12) | 2.4(12) | 2.0(11) | -4.4(11) |
| C31 | 49.9(15) | 89(2) | 46.3(15) | 12.5(16) | -1.1(14) | -7.7(16) |
| C32 | 63.2(19) | 143(4) | 37.2(15) | 2(2) | -4.3(15) | -28(2) |
| C33 | 59.9(19) | 134(3) | 56.6(17) | -40.0(19) | 9.6(16) | -24(2) |
| C34 | 56.8(18) | 95(2) | 64.8(19) | -34.1(17) | 6.4(16) | -2.0(18) |
| C35 | 45.6(14) | 63.4(16) | 49.4(14) | -13.1(14) | 1.3(13) | 3.7(13) |
| C36 | 45.5(13) | 47.3(13) | 42.8(13) | 2.7(12) | 4.3(12) | -0.3(11) |
| C37 | 47.3(14) | 52.6(15) | 57.5(16) | -15.3(13) | 4.4(13) | 1.3(12) |
| C38 | 72(2) | 51.9(16) | 73(2) | -14.6(16) | 18.3(18) | -3.3(15) |
| C39 | 89(3) | 60(2) | 111(3) | -15(2) | 31(2) | -11.4(19) |
| C40 | 88(3) | 67(2) | 140(4) | -49(2) | 18(3) | -19.5(19) |
| C41 | 64(2) | 80(2) | 97(2) | -50.1(17) | 3(2) | -8.5(17) |
| C42 | 56.0(17) | 70.6(19) | 65.5(19) | -28.2(16) | 4.7(15) | 0.9(15) |
| C43 | 39.1(12) | 50.5(13) | 38.1(11) | -10.2(11) | 0.8(10) | 1.6(11) |
| C44 | 48.2(14) | 63.7(16) | 41.8(13) | -13.0(13) | 7.6(12) | -5.0(13) |
| C45 | 51.3(15) | 70.7(19) | 64.6(18) | -19.0(16) | 12.5(15) | 5.8(14) |
| C46 | 42.9(14) | 77(2) | 67(2) | -9.8(18) | 6.2(15) | 5.3(15) |
| C47 | 41.4(13) | 57.0(15) | 51.0(15) | -9.3(13) | -0.8(12) | -0.8(12) |
| C48 | 62.7(18) | 92(3) | 65(2) | -1(2) | 20.0(16) | -20.7(18) |
| C49 | 46.8(13) | 64.0(17) | 46.6(14) | -6.3(14) | 8.1(13) | -8.0(13) |
| C50 | 65.1(18) | 58.0(16) | 54.6(17) | 6.9(15) | 1.1(16) | -9.1(14) |
| C51 | 58.8(17) | 73(2) | 64.0(19) | -11.9(17) | -8.6(16) | -17.1(15) |
| C52 | 41.1(12) | 47.7(13) | 37.2(12) | -7.6(11) | -0.7(10) | 4.3(10) |
| C53 | 54.1(15) | 47.4(13) | 46.6(14) | -2.8(12) | -5.2(13) | 8.8(12) |
| C54 | 70.1(19) | 55.8(16) | 43.7(14) | -5.5(13) | -7.2(14) | 7.9(15) |
| C55 | 109(3) | 80(2) | 42.6(16) | -0.6(17) | -7(2) | 20(2) |
| C56 | 147(4) | 78(2) | 63(2) | 14(2) | -20(3) | 27(3) |
| C57 | 135(4) | 60.4(19) | 74(3) | 5.5(19) | -7(3) | 34(2) |
| C58 | 89(2) | 55.7(16) | 58.8(19) | -7.2(16) | -5.3(19) | 22.3(16) |
| O1W | 120(3) | 146(4) | 238(6) | 21(4) | -92(3) | -3(3) |
| O2W | 123(4) | 267(8) | 139(5) | 39(6) | 23(4) | 21(5) |
| O3W | 140(8) | 131(8) | 302(19) | -65(10) | -61(11) | 22(7) |

| **Table 4 Bond Lengths for bj01.** | | | | | | |
| --- | --- | --- | --- | --- | --- | --- |
| **Atom** | **Atom** | **Length/Å** |  | **Atom** | **Atom** | **Length/Å** |
| P1 | O1 | 1.576(2) |  | P2 | O6 | 1.463(2) |
| P1 | O2 | 1.590(2) |  | P2 | O7 | 1.583(2) |
| P1 | O3 | 1.465(2) |  | P2 | O8 | 1.586(2) |
| P1 | C23 | 1.835(3) |  | P2 | C36 | 1.828(3) |
| O1 | C9 | 1.481(3) |  | O5 | C36 | 1.418(4) |
| O2 | C10 | 1.467(3) |  | O7 | C43 | 1.476(4) |
| O4 | C23 | 1.414(4) |  | O8 | C52 | 1.470(3) |
| C1 | C2 | 1.513(5) |  | C30 | C31 | 1.386(5) |
| C2 | C3 | 1.545(5) |  | C30 | C35 | 1.399(5) |
| C2 | C7 | 1.571(5) |  | C30 | C36 | 1.508(4) |
| C2 | C10 | 1.584(4) |  | C31 | C32 | 1.405(6) |
| C3 | C4 | 1.550(5) |  | C32 | C33 | 1.330(8) |
| C4 | C5 | 1.550(5) |  | C33 | C34 | 1.386(7) |
| C5 | C7 | 1.551(5) |  | C34 | C35 | 1.397(5) |
| C5 | C9 | 1.545(4) |  | C37 | C38 | 1.407(6) |
| C6 | C7 | 1.554(5) |  | C37 | C42 | 1.394(5) |
| C7 | C8 | 1.543(5) |  | C37 | C43 | 1.532(4) |
| C9 | C10 | 1.610(4) |  | C38 | C39 | 1.382(6) |
| C9 | C17 | 1.529(4) |  | C39 | C40 | 1.386(9) |
| C10 | C11 | 1.532(4) |  | C40 | C41 | 1.387(8) |
| C11 | C12 | 1.404(5) |  | C41 | C42 | 1.372(6) |
| C11 | C16 | 1.379(5) |  | C43 | C44 | 1.549(4) |
| C12 | C13 | 1.386(6) |  | C43 | C52 | 1.613(4) |
| C13 | C14 | 1.366(8) |  | C44 | C45 | 1.558(5) |
| C14 | C15 | 1.367(9) |  | C44 | C49 | 1.552(5) |
| C15 | C16 | 1.403(6) |  | C45 | C46 | 1.551(6) |
| C17 | C18 | 1.396(5) |  | C46 | C47 | 1.552(5) |
| C17 | C22 | 1.399(5) |  | C47 | C49 | 1.565(5) |
| C18 | C19 | 1.381(5) |  | C47 | C51 | 1.518(5) |
| C19 | C20 | 1.395(7) |  | C47 | C52 | 1.580(4) |
| C20 | C21 | 1.369(8) |  | C48 | C49 | 1.554(5) |
| C21 | C22 | 1.380(6) |  | C49 | C50 | 1.529(5) |
| C23 | C24 | 1.501(5) |  | C52 | C53 | 1.524(4) |
| C24 | C25 | 1.390(6) |  | C53 | C54 | 1.385(5) |
| C24 | C29 | 1.395(6) |  | C53 | C58 | 1.404(5) |
| C25 | C26 | 1.393(8) |  | C54 | C55 | 1.397(5) |
| C26 | C27 | 1.361(10) |  | C55 | C56 | 1.365(7) |
| C27 | C28 | 1.363(9) |  | C56 | C57 | 1.395(8) |
| C28 | C29 | 1.373(7) |  | C57 | C58 | 1.362(7) |

| **Table 5 Bond Angles for bj01.** | | | | | | | | |
| --- | --- | --- | --- | --- | --- | --- | --- | --- |
| **Atom** | **Atom** | **Atom** | **Angle/˚** |  | **Atom** | **Atom** | **Atom** | **Angle/˚** |
| O1 | P1 | O2 | 97.58(11) |  | O6 | P2 | O7 | 115.07(13) |
| O1 | P1 | C23 | 107.22(14) |  | O6 | P2 | O8 | 115.26(12) |
| O2 | P1 | C23 | 111.98(13) |  | O6 | P2 | C36 | 110.36(14) |
| O3 | P1 | O1 | 116.38(15) |  | O7 | P2 | O8 | 97.54(11) |
| O3 | P1 | O2 | 114.06(13) |  | O7 | P2 | C36 | 109.41(13) |
| O3 | P1 | C23 | 109.12(15) |  | O8 | P2 | C36 | 108.42(13) |
| C9 | O1 | P1 | 114.50(18) |  | C43 | O7 | P2 | 114.23(17) |
| C10 | O2 | P1 | 115.05(17) |  | C52 | O8 | P2 | 115.02(17) |
| C1 | C2 | C3 | 115.3(3) |  | C31 | C30 | C35 | 119.0(3) |
| C1 | C2 | C7 | 117.5(3) |  | C31 | C30 | C36 | 120.2(3) |
| C1 | C2 | C10 | 113.0(3) |  | C35 | C30 | C36 | 120.8(3) |
| C3 | C2 | C7 | 98.8(3) |  | C30 | C31 | C32 | 119.5(4) |
| C3 | C2 | C10 | 105.8(3) |  | C33 | C32 | C31 | 121.8(4) |
| C7 | C2 | C10 | 104.7(2) |  | C32 | C33 | C34 | 119.7(4) |
| C2 | C3 | C4 | 103.4(3) |  | C33 | C34 | C35 | 120.5(4) |
| C3 | C4 | C5 | 103.4(3) |  | C34 | C35 | C30 | 119.5(3) |
| C4 | C5 | C7 | 101.3(3) |  | O5 | C36 | P2 | 108.7(2) |
| C9 | C5 | C4 | 105.4(3) |  | O5 | C36 | C30 | 114.7(2) |
| C9 | C5 | C7 | 106.6(3) |  | C30 | C36 | P2 | 111.4(2) |
| C5 | C7 | C2 | 93.4(2) |  | C38 | C37 | C43 | 122.6(3) |
| C5 | C7 | C6 | 110.5(3) |  | C42 | C37 | C38 | 118.2(3) |
| C6 | C7 | C2 | 114.0(3) |  | C42 | C37 | C43 | 118.9(3) |
| C8 | C7 | C2 | 117.0(3) |  | C39 | C38 | C37 | 120.5(4) |
| C8 | C7 | C5 | 118.4(3) |  | C38 | C39 | C40 | 119.8(5) |
| C8 | C7 | C6 | 103.8(3) |  | C39 | C40 | C41 | 120.3(4) |
| O1 | C9 | C5 | 108.7(2) |  | C42 | C41 | C40 | 119.8(4) |
| O1 | C9 | C10 | 105.0(2) |  | C41 | C42 | C37 | 121.4(4) |
| O1 | C9 | C17 | 103.7(2) |  | O7 | C43 | C37 | 102.9(2) |
| C5 | C9 | C10 | 101.3(2) |  | O7 | C43 | C44 | 109.9(2) |
| C17 | C9 | C5 | 116.1(2) |  | O7 | C43 | C52 | 105.6(2) |
| C17 | C9 | C10 | 121.2(2) |  | C37 | C43 | C44 | 114.0(3) |
| O2 | C10 | C2 | 109.7(2) |  | C37 | C43 | C52 | 123.2(3) |
| O2 | C10 | C9 | 105.3(2) |  | C44 | C43 | C52 | 100.8(2) |
| O2 | C10 | C11 | 105.2(2) |  | C43 | C44 | C45 | 105.1(3) |
| C2 | C10 | C9 | 103.0(2) |  | C43 | C44 | C49 | 106.7(3) |
| C11 | C10 | C2 | 112.5(2) |  | C49 | C44 | C45 | 102.4(3) |
| C11 | C10 | C9 | 120.7(2) |  | C46 | C45 | C44 | 102.8(3) |
| C12 | C11 | C10 | 123.0(3) |  | C45 | C46 | C47 | 103.1(3) |
| C16 | C11 | C10 | 118.3(3) |  | C46 | C47 | C49 | 100.1(3) |
| C16 | C11 | C12 | 118.6(3) |  | C46 | C47 | C52 | 106.4(3) |
| C13 | C12 | C11 | 120.4(4) |  | C49 | C47 | C52 | 104.7(2) |
| C14 | C13 | C12 | 120.2(4) |  | C51 | C47 | C46 | 113.5(3) |
| C13 | C14 | C15 | 120.4(4) |  | C51 | C47 | C49 | 117.9(3) |
| C14 | C15 | C16 | 120.2(5) |  | C51 | C47 | C52 | 112.9(3) |
| C11 | C16 | C15 | 120.1(4) |  | C44 | C49 | C47 | 92.6(3) |
| C18 | C17 | C9 | 123.0(3) |  | C44 | C49 | C48 | 110.9(3) |
| C18 | C17 | C22 | 117.2(3) |  | C48 | C49 | C47 | 112.4(3) |
| C22 | C17 | C9 | 119.4(3) |  | C50 | C49 | C44 | 118.1(3) |
| C19 | C18 | C17 | 121.5(3) |  | C50 | C49 | C47 | 118.9(3) |
| C18 | C19 | C20 | 120.2(4) |  | C50 | C49 | C48 | 104.1(3) |
| C21 | C20 | C19 | 118.8(4) |  | O8 | C52 | C43 | 104.7(2) |
| C20 | C21 | C22 | 121.3(4) |  | O8 | C52 | C47 | 108.3(2) |
| C21 | C22 | C17 | 121.0(4) |  | O8 | C52 | C53 | 105.5(2) |
| O4 | C23 | P1 | 105.6(2) |  | C47 | C52 | C43 | 102.8(2) |
| O4 | C23 | C24 | 114.3(3) |  | C53 | C52 | C43 | 121.3(2) |
| C24 | C23 | P1 | 114.1(2) |  | C53 | C52 | C47 | 113.5(2) |
| C25 | C24 | C23 | 119.5(4) |  | C54 | C53 | C52 | 119.1(3) |
| C25 | C24 | C29 | 119.1(4) |  | C54 | C53 | C58 | 117.0(3) |
| C29 | C24 | C23 | 121.3(3) |  | C58 | C53 | C52 | 123.8(3) |
| C24 | C25 | C26 | 119.7(5) |  | C53 | C54 | C55 | 121.2(3) |
| C27 | C26 | C25 | 120.7(5) |  | C56 | C55 | C54 | 120.8(4) |
| C26 | C27 | C28 | 119.3(5) |  | C55 | C56 | C57 | 118.4(4) |
| C27 | C28 | C29 | 122.0(6) |  | C58 | C57 | C56 | 121.1(4) |
| C28 | C29 | C24 | 119.2(4) |  | C57 | C58 | C53 | 121.4(4) |

| **Table 6 Torsion Angles for bj01.** | | | | | | | | | | |
| --- | --- | --- | --- | --- | --- | --- | --- | --- | --- | --- |
| **A** | **B** | **C** | **D** | **Angle/˚** |  | **A** | **B** | **C** | **D** | **Angle/˚** |
| P1 | O1 | C9 | C5 | 122.3(2) |  | P2 | O7 | C43 | C37 | -118.9(2) |
| P1 | O1 | C9 | C10 | 14.5(3) |  | P2 | O7 | C43 | C44 | 119.3(2) |
| P1 | O1 | C9 | C17 | -113.7(2) |  | P2 | O7 | C43 | C52 | 11.4(3) |
| P1 | O2 | C10 | C2 | -115.9(2) |  | P2 | O8 | C52 | C43 | -10.8(3) |
| P1 | O2 | C10 | C9 | -5.6(3) |  | P2 | O8 | C52 | C47 | -120.0(2) |
| P1 | O2 | C10 | C11 | 122.8(2) |  | P2 | O8 | C52 | C53 | 118.2(2) |
| P1 | C23 | C24 | C25 | 109.9(3) |  | O6 | P2 | O7 | C43 | -138.95(19) |
| P1 | C23 | C24 | C29 | -74.0(4) |  | O6 | P2 | O8 | C52 | 138.72(19) |
| O1 | P1 | O2 | C10 | 13.0(2) |  | O6 | P2 | C36 | O5 | -67.9(2) |
| O1 | P1 | C23 | O4 | 63.8(2) |  | O6 | P2 | C36 | C30 | 59.5(2) |
| O1 | P1 | C23 | C24 | -169.9(2) |  | O7 | P2 | O8 | C52 | 16.4(2) |
| O1 | C9 | C10 | O2 | -5.1(3) |  | O7 | P2 | C36 | O5 | 59.7(2) |
| O1 | C9 | C10 | C2 | 109.9(2) |  | O7 | P2 | C36 | C30 | -172.9(2) |
| O1 | C9 | C10 | C11 | -123.6(3) |  | O7 | C43 | C44 | C45 | 176.8(2) |
| O1 | C9 | C17 | C18 | 81.9(3) |  | O7 | C43 | C44 | C49 | -75.0(3) |
| O1 | C9 | C17 | C22 | -91.1(3) |  | O7 | C43 | C52 | O8 | -0.4(3) |
| O2 | P1 | O1 | C9 | -16.6(2) |  | O7 | C43 | C52 | C47 | 112.8(2) |
| O2 | P1 | C23 | O4 | 169.71(18) |  | O7 | C43 | C52 | C53 | -119.2(3) |
| O2 | P1 | C23 | C24 | -64.0(3) |  | O8 | P2 | O7 | C43 | -16.5(2) |
| O2 | C10 | C11 | C12 | -160.8(3) |  | O8 | P2 | C36 | O5 | 165.00(19) |
| O2 | C10 | C11 | C16 | 22.9(4) |  | O8 | P2 | C36 | C30 | -67.6(2) |
| O3 | P1 | O1 | C9 | -138.3(2) |  | O8 | C52 | C53 | C54 | 21.2(4) |
| O3 | P1 | O2 | C10 | 136.4(2) |  | O8 | C52 | C53 | C58 | -161.6(3) |
| O3 | P1 | C23 | O4 | -63.0(2) |  | C30 | C31 | C32 | C33 | -0.7(6) |
| O3 | P1 | C23 | C24 | 63.3(3) |  | C31 | C30 | C35 | C34 | -0.2(5) |
| O4 | C23 | C24 | C25 | -128.5(3) |  | C31 | C30 | C36 | P2 | 112.2(3) |
| O4 | C23 | C24 | C29 | 47.6(4) |  | C31 | C30 | C36 | O5 | -123.7(3) |
| C1 | C2 | C3 | C4 | 166.9(3) |  | C31 | C32 | C33 | C34 | 0.6(7) |
| C1 | C2 | C7 | C5 | 176.2(3) |  | C32 | C33 | C34 | C35 | -0.3(7) |
| C1 | C2 | C7 | C6 | -69.5(4) |  | C33 | C34 | C35 | C30 | 0.1(6) |
| C1 | C2 | C7 | C8 | 51.8(4) |  | C35 | C30 | C31 | C32 | 0.5(5) |
| C1 | C2 | C10 | O2 | -47.6(3) |  | C35 | C30 | C36 | P2 | -68.9(3) |
| C1 | C2 | C10 | C9 | -159.3(3) |  | C35 | C30 | C36 | O5 | 55.2(4) |
| C1 | C2 | C10 | C11 | 69.2(3) |  | C36 | P2 | O7 | C43 | 96.2(2) |
| C2 | C3 | C4 | C5 | -5.5(4) |  | C36 | P2 | O8 | C52 | -97.0(2) |
| C2 | C10 | C11 | C12 | 79.7(4) |  | C36 | C30 | C31 | C32 | 179.5(3) |
| C2 | C10 | C11 | C16 | -96.5(3) |  | C36 | C30 | C35 | C34 | -179.2(3) |
| C3 | C2 | C7 | C5 | -59.2(3) |  | C37 | C38 | C39 | C40 | 1.9(7) |
| C3 | C2 | C7 | C6 | 55.1(4) |  | C37 | C43 | C44 | C45 | 61.9(3) |
| C3 | C2 | C7 | C8 | 176.4(3) |  | C37 | C43 | C44 | C49 | 170.2(3) |
| C3 | C2 | C10 | O2 | -174.6(2) |  | C37 | C43 | C52 | O8 | 117.0(3) |
| C3 | C2 | C10 | C9 | 73.6(3) |  | C37 | C43 | C52 | C47 | -129.9(3) |
| C3 | C2 | C10 | C11 | -57.9(3) |  | C37 | C43 | C52 | C53 | -1.9(4) |
| C3 | C4 | C5 | C7 | -32.4(3) |  | C38 | C37 | C42 | C41 | -0.1(6) |
| C3 | C4 | C5 | C9 | 78.4(3) |  | C38 | C37 | C43 | O7 | 81.9(4) |
| C4 | C5 | C7 | C2 | 56.2(3) |  | C38 | C37 | C43 | C44 | -159.2(3) |
| C4 | C5 | C7 | C6 | -61.1(4) |  | C38 | C37 | C43 | C52 | -36.8(5) |
| C4 | C5 | C7 | C8 | 179.5(3) |  | C38 | C39 | C40 | C41 | -0.6(8) |
| C4 | C5 | C9 | O1 | 179.5(2) |  | C39 | C40 | C41 | C42 | -0.9(8) |
| C4 | C5 | C9 | C10 | -70.3(3) |  | C40 | C41 | C42 | C37 | 1.3(7) |
| C4 | C5 | C9 | C17 | 63.1(3) |  | C42 | C37 | C38 | C39 | -1.5(6) |
| C5 | C9 | C10 | O2 | -118.2(2) |  | C42 | C37 | C43 | O7 | -91.0(3) |
| C5 | C9 | C10 | C2 | -3.2(3) |  | C42 | C37 | C43 | C44 | 28.0(4) |
| C5 | C9 | C10 | C11 | 123.3(3) |  | C42 | C37 | C43 | C52 | 150.4(3) |
| C5 | C9 | C17 | C18 | -158.9(3) |  | C43 | C37 | C38 | C39 | -174.4(4) |
| C5 | C9 | C17 | C22 | 28.0(4) |  | C43 | C37 | C42 | C41 | 173.0(4) |
| C7 | C2 | C3 | C4 | 40.8(3) |  | C43 | C44 | C45 | C46 | 78.8(3) |
| C7 | C2 | C10 | O2 | 81.5(3) |  | C43 | C44 | C49 | C47 | -54.4(3) |
| C7 | C2 | C10 | C9 | -30.2(3) |  | C43 | C44 | C49 | C48 | -169.6(3) |
| C7 | C2 | C10 | C11 | -161.7(2) |  | C43 | C44 | C49 | C50 | 70.5(4) |
| C7 | C5 | C9 | O1 | -73.5(3) |  | C43 | C52 | C53 | C54 | 139.6(3) |
| C7 | C5 | C9 | C10 | 36.7(3) |  | C43 | C52 | C53 | C58 | -43.2(5) |
| C7 | C5 | C9 | C17 | 170.1(3) |  | C44 | C43 | C52 | O8 | -114.7(2) |
| C9 | C5 | C7 | C2 | -53.8(3) |  | C44 | C43 | C52 | C47 | -1.6(3) |
| C9 | C5 | C7 | C6 | -171.0(3) |  | C44 | C43 | C52 | C53 | 126.4(3) |
| C9 | C5 | C7 | C8 | 69.5(4) |  | C44 | C45 | C46 | C47 | -5.0(4) |
| C9 | C10 | C11 | C12 | -42.3(4) |  | C45 | C44 | C49 | C47 | 55.8(3) |
| C9 | C10 | C11 | C16 | 141.5(3) |  | C45 | C44 | C49 | C48 | -59.4(4) |
| C9 | C17 | C18 | C19 | -174.8(3) |  | C45 | C44 | C49 | C50 | -179.4(3) |
| C9 | C17 | C22 | C21 | 174.6(4) |  | C45 | C46 | C47 | C49 | 40.5(3) |
| C10 | C2 | C3 | C4 | -67.4(3) |  | C45 | C46 | C47 | C51 | 167.0(3) |
| C10 | C2 | C7 | C5 | 49.9(3) |  | C45 | C46 | C47 | C52 | -68.2(3) |
| C10 | C2 | C7 | C6 | 164.2(3) |  | C46 | C47 | C49 | C44 | -58.5(3) |
| C10 | C2 | C7 | C8 | -74.5(3) |  | C46 | C47 | C49 | C48 | 55.4(4) |
| C10 | C9 | C17 | C18 | -35.4(4) |  | C46 | C47 | C49 | C50 | 177.3(3) |
| C10 | C9 | C17 | C22 | 151.6(3) |  | C46 | C47 | C52 | O8 | -176.6(2) |
| C10 | C11 | C12 | C13 | -177.5(3) |  | C46 | C47 | C52 | C43 | 72.9(3) |
| C10 | C11 | C16 | C15 | 176.8(4) |  | C46 | C47 | C52 | C53 | -59.8(3) |
| C11 | C12 | C13 | C14 | 0.5(6) |  | C47 | C52 | C53 | C54 | -97.2(4) |
| C12 | C11 | C16 | C15 | 0.4(6) |  | C47 | C52 | C53 | C58 | 80.0(4) |
| C12 | C13 | C14 | C15 | 1.3(7) |  | C49 | C44 | C45 | C46 | -32.6(3) |
| C13 | C14 | C15 | C16 | -2.2(8) |  | C49 | C47 | C52 | O8 | 77.9(3) |
| C14 | C15 | C16 | C11 | 1.3(7) |  | C49 | C47 | C52 | C43 | -32.5(3) |
| C16 | C11 | C12 | C13 | -1.3(5) |  | C49 | C47 | C52 | C53 | -165.3(3) |
| C17 | C9 | C10 | O2 | 111.6(3) |  | C51 | C47 | C49 | C44 | 178.0(3) |
| C17 | C9 | C10 | C2 | -133.5(3) |  | C51 | C47 | C49 | C48 | -68.1(4) |
| C17 | C9 | C10 | C11 | -7.0(4) |  | C51 | C47 | C49 | C50 | 53.8(4) |
| C17 | C18 | C19 | C20 | 0.8(5) |  | C51 | C47 | C52 | O8 | -51.5(3) |
| C18 | C17 | C22 | C21 | 1.2(6) |  | C51 | C47 | C52 | C43 | -161.9(3) |
| C18 | C19 | C20 | C21 | 0.6(6) |  | C51 | C47 | C52 | C53 | 65.3(4) |
| C19 | C20 | C21 | C22 | -1.2(7) |  | C52 | C43 | C44 | C45 | -72.1(3) |
| C20 | C21 | C22 | C17 | 0.2(7) |  | C52 | C43 | C44 | C49 | 36.1(3) |
| C22 | C17 | C18 | C19 | -1.7(5) |  | C52 | C47 | C49 | C44 | 51.6(3) |
| C23 | P1 | O1 | C9 | 99.3(2) |  | C52 | C47 | C49 | C48 | 165.5(3) |
| C23 | P1 | O2 | C10 | -99.1(2) |  | C52 | C47 | C49 | C50 | -72.6(4) |
| C23 | C24 | C25 | C26 | 175.7(4) |  | C52 | C53 | C54 | C55 | 178.0(4) |
| C23 | C24 | C29 | C28 | -175.4(4) |  | C52 | C53 | C58 | C57 | -176.7(4) |
| C24 | C25 | C26 | C27 | 0.2(7) |  | C53 | C54 | C55 | C56 | -1.6(8) |
| C25 | C24 | C29 | C28 | 0.7(6) |  | C54 | C53 | C58 | C57 | 0.6(7) |
| C25 | C26 | C27 | C28 | 0.0(9) |  | C54 | C55 | C56 | C57 | 1.4(9) |
| C26 | C27 | C28 | C29 | 0.2(9) |  | C55 | C56 | C57 | C58 | -0.2(10) |
| C27 | C28 | C29 | C24 | -0.6(7) |  | C56 | C57 | C58 | C53 | -0.8(9) |
| C29 | C24 | C25 | C26 | -0.5(6) |  | C58 | C53 | C54 | C55 | 0.6(6) |

| **Table 7 Hydrogen Atom Coordinates (Å×10^4^) and Isotropic Displacement Parameters (Å^2^×10^3^) for bj01.** | | | | |
| --- | --- | --- | --- | --- |
| **Atom** | ***x*** | ***y*** | ***z*** | **U(eq)** |
| H4 | 5085.2 | 331.99 | 5206.94 | 83 |
| H1A | 9617.59 | 1206.85 | 4886.55 | 90 |
| H1B | 8856.41 | 755.5 | 4630.68 | 90 |
| H1C | 9412.66 | 353.63 | 5159.44 | 90 |
| H3A | 9652.95 | 1779.02 | 6055.64 | 71 |
| H3B | 9336.92 | 1016.91 | 6436.55 | 71 |
| H4A | 8804.73 | 2532.69 | 6654.18 | 70 |
| H4B | 8427.7 | 1760.77 | 6979.53 | 70 |
| H5 | 7522.79 | 2546.49 | 6157.29 | 61 |
| H6A | 9227.92 | 2650.02 | 4973.84 | 107 |
| H6B | 9147.07 | 2836.68 | 5731.58 | 107 |
| H6C | 8559.11 | 3233.15 | 5223.97 | 107 |
| H8A | 8050.66 | 2329.57 | 4424.46 | 90 |
| H8B | 7280.47 | 2386.1 | 4864.38 | 90 |
| H8C | 7606.05 | 1548.28 | 4644.3 | 90 |
| H12 | 8539.17 | 446.99 | 7090.73 | 68 |
| H13 | 9059.94 | -671.35 | 7606.67 | 95 |
| H14 | 9172.29 | -1853.1 | 7039.4 | 112 |
| H15 | 8819.35 | -1923.18 | 5943.93 | 107 |
| H16 | 8261.12 | -815.98 | 5416.83 | 74 |
| H18 | 6979.95 | -103.68 | 6801.5 | 58 |
| H19 | 6367.6 | -311.06 | 7806.35 | 74 |
| H20 | 6041.4 | 771.97 | 8481.4 | 91 |
| H21 | 6320.2 | 2045.34 | 8121.08 | 97 |
| H22 | 6948.28 | 2260.45 | 7127.09 | 78 |
| H23 | 6156.18 | -297.44 | 5993.1 | 59 |
| H25 | 6582.4 | -1593.02 | 5858.09 | 88 |
| H26 | 6521.53 | -2810.76 | 5310.05 | 117 |
| H27 | 5912.1 | -2907.11 | 4297.62 | 130 |
| H28 | 5356.34 | -1797.13 | 3826.39 | 109 |
| H29 | 5403.51 | -574.41 | 4340.58 | 79 |
| H5A | 5762.99 | 1721.46 | 4246.76 | 86 |
| H31 | 4495.63 | 2324.89 | 2502.08 | 74 |
| H32 | 4748.22 | 1629.24 | 1527.41 | 97 |
| H33 | 5417.28 | 462.86 | 1532.2 | 100 |
| H34 | 5846.42 | -82.42 | 2523.53 | 86 |
| H35 | 5603.7 | 577.9 | 3517.68 | 63 |
| H36 | 4618.68 | 2486.41 | 3630.77 | 54 |
| H38 | 3826.94 | 3491.4 | 3902.84 | 79 |
| H39 | 4343.64 | 4760.12 | 4046.78 | 104 |
| H40 | 4345.7 | 5338.38 | 5088.53 | 118 |
| H41 | 3807.6 | 4658.73 | 5982.77 | 96 |
| H42 | 3241.01 | 3422.98 | 5832.73 | 77 |
| H44 | 2766.05 | 2231.63 | 5788.58 | 61 |
| H45A | 1480.22 | 2791.38 | 5605.53 | 75 |
| H45B | 1996.76 | 3314.18 | 5113.47 | 75 |
| H46A | 1332.58 | 2754.55 | 4260.5 | 74 |
| H46B | 876.01 | 2174.69 | 4749.03 | 74 |
| H48A | 1111.95 | 1595.74 | 5571.5 | 110 |
| H48B | 1656.18 | 1043.93 | 6008.06 | 110 |
| H48C | 1227.34 | 694.29 | 5381.5 | 110 |
| H50A | 2484.27 | 222.29 | 5385.18 | 89 |
| H50B | 3222.9 | 792.53 | 5458.45 | 89 |
| H50C | 2950.15 | 507.39 | 4754.34 | 89 |
| H51A | 1133.57 | 861.33 | 4150.39 | 98 |
| H51B | 1982.7 | 674.46 | 3860.4 | 98 |
| H51C | 1460.54 | 1356.27 | 3549.37 | 98 |
| H54 | 2918.57 | 1782.64 | 2873.55 | 68 |
| H55 | 2671.96 | 2526.61 | 1930.3 | 93 |
| H56 | 2150.48 | 3796.04 | 2007.17 | 115 |
| H57 | 1927.59 | 4341.51 | 3051.41 | 108 |
| H58 | 2208.54 | 3633.14 | 3984.44 | 81 |
| H1WA | 5703 | 3423.2 | 6036.22 | 252 |
| H1WB | 5209.42 | 2794.13 | 6350.31 | 252 |
| H2WA | 4746.81 | 1822.2 | 6948.14 | 265 |
| H2WB | 3949.9 | 2268.4 | 6978.26 | 265 |
| H3WA | 6713.64 | 3948.7 | 5217.11 | 286 |
| H3WB | 5904.16 | 4002.96 | 5071.54 | 286 |

| **Table 8 Atomic Occupancy for bj01.** | | | | | | | |
| --- | --- | --- | --- | --- | --- | --- | --- |
| **Atom** | ***Occupancy*** |  | **Atom** | ***Occupancy*** |  | **Atom** | ***Occupancy*** |
| O3W | 0.5 |  | H3WA | 0.5 |  | H3WB | 0.5 |

**Experimental**

Single crystals of C_29_H_33.5_O_5.25_P **[bj01]** were **[]**. A suitable crystal was selected and **[]** on a **Rigaku R-AXIS SPIDER IP** diffractometer. The crystal was kept at 293.15 K during data collection. Using Olex2 [1], the structure was solved with the SHELXS [2] structure solution program using Direct Methods and refined with the SHELXL [3] refinement package using Least Squares minimisation.

1. Dolomanov, O.V., Bourhis, L.J., Gildea, R.J, Howard, J.A.K. & Puschmann, H. (2009), J. Appl. Cryst. 42, 339-341.
2. Sheldrick, G.M. (2008). Acta Cryst. A64, 112-122.
3. Sheldrick, G.M. (2015). Acta Cryst. C71, 3-8.

Number of restraints - 1, number of constraints - unknown.

Details:

1. Fixed Uiso
 At 1.2 times of:
 All C(H) groups, All C(H,H) groups
 At 1.5 times of:
 All C(H,H,H) groups, All O(H) groups, All O(H,H) groups
2. Others
 Fixed Sof: O3W(0.5) H3WA(0.5) H3WB(0.5)
3.a Free rotating group:
 O1W(H1WA,H1WB), O2W(H2WA,H2WB), O3W(H3WA,H3WB)
3.b Ternary CH refined with riding coordinates:
 C5(H5), C23(H23), C36(H36), C44(H44)
3.c Secondary CH2 refined with riding coordinates:
 C3(H3A,H3B), C4(H4A,H4B), C45(H45A,H45B), C46(H46A,H46B)
3.d Aromatic/amide H refined with riding coordinates:
 C12(H12), C13(H13), C14(H14), C15(H15), C16(H16), C18(H18), C19(H19),
 C20(H20), C21(H21), C22(H22), C25(H25), C26(H26), C27(H27), C28(H28), C29(H29),
 C31(H31), C32(H32), C33(H33), C34(H34), C35(H35), C38(H38), C39(H39),
 C40(H40), C41(H41), C42(H42), C54(H54), C55(H55), C56(H56), C57(H57), C58(H58)
3.e Idealised Me refined as rotating group:
 C1(H1A,H1B,H1C), C6(H6A,H6B,H6C), C8(H8A,H8B,H8C), C48(H48A,H48B,H48C),
 C50(H50A,H50B,H50C), C51(H51A,H51B,H51C)
3.f Idealised tetrahedral OH refined as rotating group:
 O4(H4), O5(H5A)

This report has been created with Olex2, compiled on 2022.04.07 svn.rca3783a0 for OlexSys. Please [let us know](mailto:support@olex2.org?subject=Olex2%20Report) if there are any errors or if you would like to have additional features.
