# Supplementary material for: Stereoselective Pudovik reaction of aldehydes, aldimines, and nitroalkenes with CAMDOL-derived H-phosphonate
Source: Commun Chem. 2025 Nov 14;8:349. doi: 10.1038/s42004-025-01735-4 (PMC12618634; doi:10.1038/s42004-025-01735-4)

checkCIF/PLATON report

You have not supplied any structure factors. As a result the full set of tests cannot be run.

THIS REPORT IS FOR GUIDANCE ONLY. IF USED AS PART OF A REVIEW PROCEDURE FOR PUBLICATION, IT SHOULD NOT REPLACE THE EXPERTISE OF AN EXPERIENCED CRYSTALLOGRAPHIC REFEREE.

No syntax errors found.   CIF dictionary   Interpreting this report

Datablock: bj01

```
Bond precision:   C-C = 0.0057 A          Wavelength=1.54184

Cell:             a=16.72503(14)     b=16.72503(14)     c=20.1957(2)
                  alpha=90           beta=90            gamma=90
Temperature:      293 K

                 Calculated                       Reported
Volume           5649.28(11)                      5649.27(12)
Space group      P 43                             P 43
Hall group       P 4cw                            P 4cw
Moiety formula   4(C29 H31 O4 P), 5(H2 O)         C29 H31 O4 P, 1.25(H2 O)
Sum formula      C116 H134 O21 P4                 C29 H33.50 O5.25 P
Mr               1988.11                          497.03
Dx,g cm-3        1.169                            1.169
Z                2                                8
Mu (mm-1)        1.148                            1.148
F000             2116.0                           2116.0
F000'            2124.41
h,k,lmax         21,21,25                         19,21,25
Nref             11881[ 6116]                     11316
Tmin,Tmax        0.836,0.881                      0.438,1.000
Tmin'            0.832

Correction method= # Reported T Limits: Tmin=0.438 Tmax=1.000
AbsCorr = MULTI-SCAN

Data completeness= 1.85/0.95        Theta(max)= 76.495

R(reflections)= 0.1007( 10648)                        wR2(reflections)=
                                                      0.2753( 11316)
S = 1.301                     Npar= 657
```

The following ALERTS were generated. Each ALERT has the format
test-name_ALERT_alert-type_alert-level.
Click on the hyperlinks for more details of the test.

Alert level B

```
PLAT420_ALERT_2_B D-H Bond Without Acceptor  O2W      --H2WA     .     Please Check
PLAT420_ALERT_2_B D-H Bond Without Acceptor  O2W      --H2WB     .     Please Check
PLAT420_ALERT_2_B D-H Bond Without Acceptor  O3W      --H3WA     .     Please Check
PLAT420_ALERT_2_B D-H Bond Without Acceptor  O3W      --H3WB     .     Please Check
```

Alert level C

```
PLAT042_ALERT_1_C Calc. and Reported MoietyFormula Strings  Differ     Please Check
              Calc: 4(C29 H31 O4 P), 5(H2 O)
              Rep.: C29 H31 O4 P, 1.25(H2 O)
PLAT084_ALERT_3_C High wR2 Value (i.e. > 0.25) ..................       0.28 Report
PLAT094_ALERT_2_C Ratio of Maximum / Minimum Residual Density ....       2.09 Report
PLAT230_ALERT_2_C Hirshfeld Test Diff for    C26      --C27      .       5.7 s.u.
PLAT260_ALERT_2_C Large Average Ueq of Residue Including       O1W      0.168 Check
PLAT260_ALERT_2_C Large Average Ueq of Residue Including       O2W      0.176 Check
PLAT260_ALERT_2_C Large Average Ueq of Residue Including       O3W      0.191 Check
PLAT340_ALERT_3_C Low Bond Precision on  C-C Bonds ..............    0.00569 Ang.
PLAT355_ALERT_3_C Long   O-H (X0.82,N0.98A)  O2W      - H2WB     .       1.02 Ang.
PLAT601_ALERT_2_C Unit Cell Contains Solvent Accessible VOIDS of .         76 Ang**3
```

Alert level G

```
PLAT007_ALERT_5_G Number of Unrefined Donor-H Atoms .............          8 Report
              H4    H5A   H1WA  H1WB  H2WA  H2WB  H3WA  H3WB
PLAT012_ALERT_1_G N.O.K.   _shelx_res_checksum Found in CIF ......     Please Check
PLAT045_ALERT_1_G Calculated and Reported Z Differ by a Factor ...      0.250 Check
PLAT072_ALERT_2_G SHELXL First  Parameter in WGHT  Unusually Large       0.20 Report
PLAT143_ALERT_4_G s.u. on c - Axis Small or Missing .............    0.00020 Ang.
PLAT199_ALERT_1_G Reported _cell_measurement_temperature ..... (K)        293 Check
PLAT200_ALERT_1_G Reported   _diffrn_ambient_temperature ..... (K)        293 Check
PLAT300_ALERT_4_G Atom Site Occupancy of O3W       Constrained at         0.5 Check
PLAT300_ALERT_4_G Atom Site Occupancy of H3WA      Constrained at         0.5 Check
PLAT300_ALERT_4_G Atom Site Occupancy of H3WB      Constrained at         0.5 Check
PLAT302_ALERT_4_G Anion/Solvent/Minor-Residue Disorder (Resd    5)       100% Note
PLAT304_ALERT_4_G Non-Integer Number of Atoms in ..... (Resd    5)       1.50 Check
PLAT720_ALERT_4_G Number of Unusual/Non-Standard Labels ..........          6 Note
              H1WA    H1WB    H2WA    H2WB    H3WA    H3WB
PLAT950_ALERT_5_G Calculated (ThMax) and CIF-Reported Hmax Differ           2 Units
```

0 **ALERT level A** = Most likely a serious problem - resolve or explain
4 **ALERT level B** = A potentially serious problem, consider carefully
10 **ALERT level C** = Check. Ensure it is not caused by an omission or oversight
14 **ALERT level G** = General information/check it is not something unexpected

5 ALERT type 1 CIF construction/syntax error, inconsistent or missing data

```
11 ALERT type 2 Indicator that the structure model may be wrong or deficient
 3 ALERT type 3 Indicator that the structure quality may be low
 7 ALERT type 4 Improvement, methodology, query or suggestion
 2 ALERT type 5 Informative message, check
```

It is advisable to attempt to resolve as many as possible of the alerts in all categories. Often the minor alerts point to easily fixed oversights, errors and omissions in your CIF or refinement strategy, so attention to these fine details can be worthwhile. In order to resolve some of the more serious problems it may be necessary to carry out additional measurements or structure refinements. However, the purpose of your study may justify the reported deviations and the more serious of these should normally be commented upon in the discussion or experimental section of a paper or in the "special_details" fields of the CIF. checkCIF was carefully designed to identify outliers and unusual parameters, but every test has its limitations and alerts that are not important in a particular case may appear. Conversely, the absence of alerts does not guarantee there are no aspects of the results needing attention. It is up to the individual to critically assess their own results and, if necessary, seek expert advice.

Publication of your CIF in IUCr journals

A basic structural check has been run on your CIF. These basic checks will be run on all CIFs submitted for publication in IUCr journals (*Acta Crystallographica*, *Journal of Applied Crystallography*, *Journal of Synchrotron Radiation*); however, if you intend to submit to *Acta Crystallographica Section C* or *E* or *IUCrData*, you should make sure that full publication checks are run on the final version of your CIF prior to submission.

Publication of your CIF in other journals

Please refer to the *Notes for Authors* of the relevant journal for any special instructions relating to CIF submission.

PLATON version of 06/01/2024; check.def file version of 05/01/2024

Datablock bj01 - ellipsoid plot

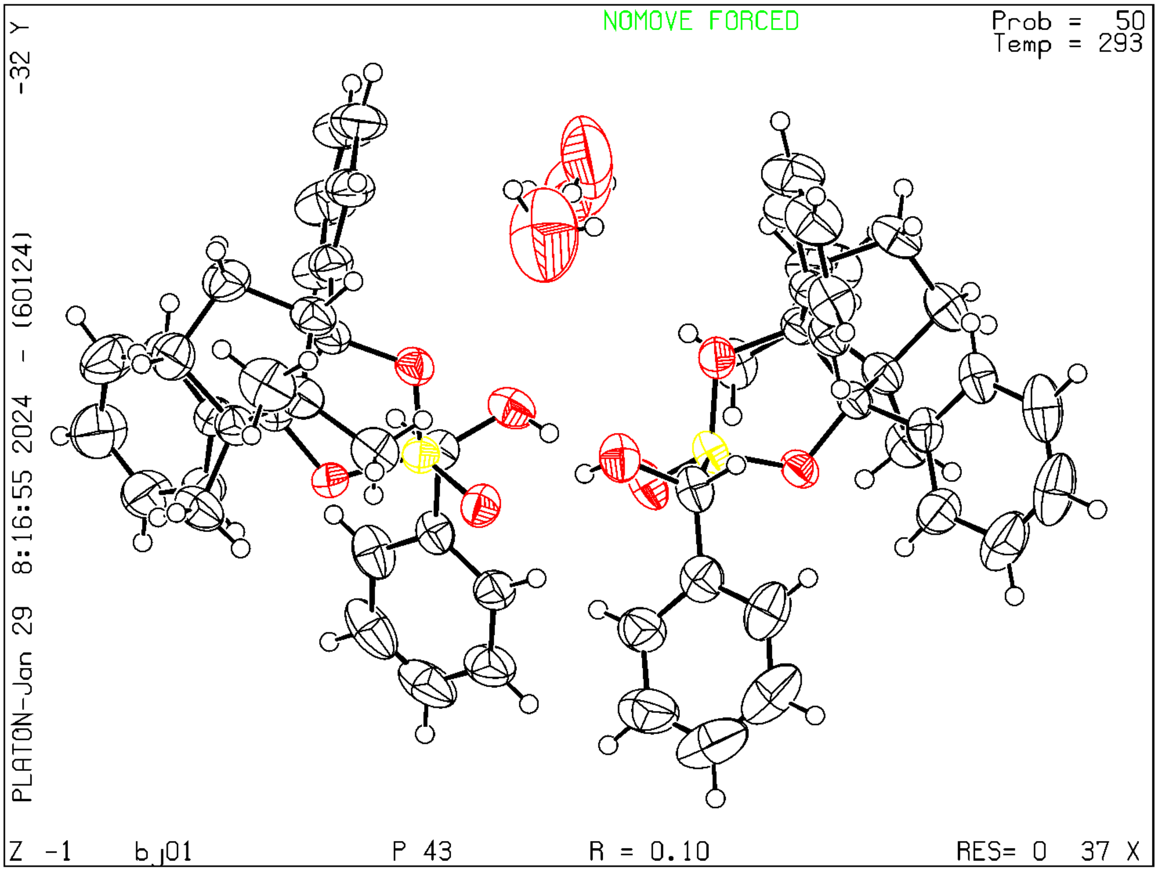

Supplement: Supplementary file 4 — Supplementary Data 2 [file 42004_2025_1735_MOESM4_ESM.zip › Supplementary Data 4-the cif file of 3a/checkcif.pdf]
