# Supplementary material for: Stereoselective Pudovik reaction of aldehydes, aldimines, and nitroalkenes with CAMDOL-derived H-phosphonate
Source: Commun Chem. 2025 Nov 14;8:349. doi: 10.1038/s42004-025-01735-4 (PMC12618634; doi:10.1038/s42004-025-01735-4)
Supplement: Supplementary file 5 — Supplementary Data 3 [file 42004_2025_1735_MOESM5_ESM.zip › Supplementary Data 5-the cif file of 6a/bj0晶体学数据.docx]

**bj02**

| **Table 1 Crystal data and structure refinement for bj02.** | |
| --- | --- |
| Identification code | bj02 |
| Empirical formula | C_36_H_38_NO_5_PS |
| Formula weight | 627.70 |
| Temperature/K | 293.15 |
| Crystal system | monoclinic |
| Space group | P2_1_ |
| a/Å | 12.84397(17) |
| b/Å | 19.93976(19) |
| c/Å | 14.01120(18) |
| α/° | 90 |
| β/° | 107.6368(14) |
| γ/° | 90 |
| Volume/Å^3^ | 3419.68(8) |
| Z | 4 |
| ρ_calc_g/cm^3^ | 1.219 |
| μ/mm^‑1^ | 1.615 |
| F(000) | 1328.0 |
| Crystal size/mm^3^ | 0.16 × 0.14 × 0.12 |
| Radiation | Cu Kα (λ = 1.54184) |
| 2Θ range for data collection/° | 6.62 to 153.358 |
| Index ranges | -16 ≤ h ≤ 16, -24 ≤ k ≤ 24, -17 ≤ l ≤ 16 |
| Reflections collected | 37242 |
| Independent reflections | 12615 [R_int_ = 0.0185, R_sigma_ = 0.0147] |
| Data/restraints/parameters | 12615/8/801 |
| Goodness-of-fit on F^2^ | 1.069 |
| Final R indexes [I>=2σ (I)] | R_1_ = 0.0332, wR_2_ = 0.0962 |
| Final R indexes [all data] | R_1_ = 0.0345, wR_2_ = 0.0972 |
| Largest diff. peak/hole / e Å^-3^ | 0.24/-0.25 |
| Flack/Hooft parameter | 0.007(5)/0.000(2) |

**Crystal structure determination of [bj02]**

**Crystal Data** for C_36_H_38_NO_5_PS (*M*=627.70 g/mol): monoclinic, space group P2_1_ (no. 4), *a* = 12.84397(17) Å, *b* = 19.93976(19) Å, *c* = 14.01120(18) Å, *β* = 107.6368(14)°, *V*= 3419.68(8) Å^3^, *Z* = 4, *T* = 293.15 K, μ(Cu Kα) = 1.615 mm^-1^, *Dcalc* = 1.219 g/cm^3^, 37242 reflections measured (6.62° ≤ 2Θ ≤ 153.358°), 12615 unique (*R*_int_ = 0.0185, R_sigma_ = 0.0147) which were used in all calculations. The final *R*_1_ was 0.0332 (I > 2σ(I)) and *wR*_2_ was 0.0972 (all data).

| **Table 2 Fractional Atomic Coordinates (×10^4^) and Equivalent Isotropic Displacement Parameters (Å^2^×10^3^) for bj02. U_eq_ is defined as 1/3 of the trace of the orthogonalised U_IJ_ tensor.** | | | | |
| --- | --- | --- | --- | --- |
| **Atom** | ***x*** | ***y*** | ***z*** | **U(eq)** |
| S1 | 6081.7(7) | 4196.8(3) | 7797.7(5) | 62.57(18) |
| P1 | 5725.0(5) | 5979.5(3) | 6169.2(4) | 46.49(14) |
| O1 | 5773.8(15) | 6666.9(9) | 6744.9(14) | 53.1(4) |
| O2 | 6983.0(14) | 5919.4(9) | 6287.6(12) | 49.8(4) |
| O3 | 5035.0(14) | 5938.1(10) | 5126.9(13) | 54.3(4) |
| O4 | 6582(2) | 4590.5(12) | 8663.0(17) | 81.7(7) |
| O5 | 6657(2) | 3648.7(12) | 7549(2) | 81.8(6) |
| N1 | 5788(2) | 4703.0(11) | 6838.3(17) | 54.2(5) |
| C1 | 7700(2) | 6357.0(13) | 7042(2) | 53.4(6) |
| C2 | 8301(2) | 6859.4(15) | 6511(2) | 59.5(6) |
| C3 | 7451(2) | 7436.1(14) | 6091(2) | 59.6(6) |
| C4 | 7207(3) | 7534.2(14) | 7097(2) | 59.5(6) |
| C5 | 6882(2) | 6841.4(13) | 7398(2) | 53.1(6) |
| C6 | 6801(3) | 6804.7(15) | 8463(2) | 64.4(7) |
| C7 | 6553(4) | 7383.2(19) | 8896(3) | 92.3(13) |
| C8 | 6443(6) | 7368(3) | 9848(4) | 126(2) |
| C9 | 6548(6) | 6789(3) | 10369(4) | 127(2) |
| C10 | 6777(5) | 6204(2) | 9951(3) | 108.2(17) |
| C11 | 6891(3) | 6211.1(18) | 9001(2) | 76.5(9) |
| C12 | 8375(3) | 7676.6(16) | 7816(3) | 72.6(8) |
| C13 | 9122(3) | 7248.6(18) | 7364(3) | 74.2(8) |
| C14 | 7951(3) | 8085.3(19) | 5781(3) | 83.7(10) |
| C15 | 6503(3) | 7279.9(16) | 5155(2) | 66.9(7) |
| C16 | 8785(3) | 6520.4(19) | 5778(3) | 77.8(9) |
| C17 | 8501(2) | 5886.1(16) | 7766(2) | 65.2(7) |
| C18 | 9096(3) | 6053.9(19) | 8744(3) | 81.5(10) |
| C19 | 9834(5) | 5612(3) | 9347(3) | 115.0(17) |
| C20 | 9994(5) | 5004(3) | 9009(4) | 147(3) |
| C21 | 9418(5) | 4816(3) | 8033(4) | 138(3) |
| C22 | 8670(3) | 5253.6(19) | 7437(3) | 89.5(12) |
| C23 | 5310(2) | 5357.8(12) | 6943.0(19) | 51.6(6) |
| C24 | 4086(3) | 5357.3(13) | 6752(2) | 57.2(6) |
| C25 | 3657(3) | 5712.5(17) | 7403(3) | 79.6(10) |
| C26 | 2535(5) | 5687(3) | 7260(5) | 105.4(15) |
| C27 | 1864(4) | 5338(3) | 6499(5) | 113.9(18) |
| C28 | 2291(3) | 4993(2) | 5851(4) | 97.5(13) |
| C29 | 3393(3) | 5003.4(19) | 5963(3) | 74.6(8) |
| C30 | 4831(3) | 3893.8(14) | 7905(2) | 62.0(7) |
| C31 | 4318(3) | 3366.7(19) | 7317(3) | 79.6(9) |
| C32 | 3316(4) | 3158(3) | 7361(4) | 101.3(13) |
| C33 | 2806(4) | 3464(3) | 7962(4) | 104.0(15) |
| C34 | 4352(4) | 4200.1(18) | 8543(3) | 80.7(10) |
| C35 | 3335(4) | 3994(3) | 8557(4) | 101.2(14) |
| C36 | 1691(5) | 3241(5) | 7994(6) | 165(3) |
| S2 | 6034.0(7) | 5808.6(3) | 2705.7(6) | 64.50(19) |
| P2 | 5706.0(5) | 4021.3(3) | 4161.9(4) | 45.68(13) |
| O6 | 6969.3(13) | 4069.0(9) | 4722.7(13) | 50.9(4) |
| O7 | 5716.3(14) | 3326.8(9) | 3610.5(14) | 53.0(4) |
| O8 | 5025.2(14) | 4071.6(10) | 4830.2(13) | 54.9(4) |
| O9 | 6447(3) | 5406.3(13) | 2062(2) | 91.7(8) |
| O10 | 6675(2) | 6340.5(12) | 3270(2) | 83.1(7) |
| N2 | 5778.0(19) | 5299.9(11) | 3522.0(17) | 52.3(5) |
| C37 | 8408(2) | 4100.0(16) | 3989(2) | 62.5(7) |
| C38 | 8986(3) | 3910(2) | 3332(3) | 77.1(9) |
| C39 | 9646(3) | 4356(3) | 3050(4) | 101.4(14) |
| C40 | 9759(4) | 5004(3) | 3401(5) | 123.7(19) |
| C41 | 9210(4) | 5208(2) | 4049(4) | 103.4(15) |
| C42 | 8544(3) | 4757.8(18) | 4346(3) | 76.0(9) |
| C43 | 7656(2) | 3627.6(13) | 4332(2) | 51.4(5) |
| C44 | 8280(2) | 3126.1(16) | 5192(2) | 60.8(7) |
| C45 | 9077(3) | 2736.9(18) | 4759(3) | 73.8(8) |
| C46 | 8309(3) | 2308.0(18) | 3920(3) | 74.9(9) |
| C47 | 7153(2) | 2450.2(15) | 4029(2) | 60.6(6) |
| C48 | 6815(2) | 3145.1(13) | 3545.3(19) | 51.6(6) |
| C49 | 6688(3) | 3169.8(16) | 2426(2) | 62.5(7) |
| C50 | 6381(4) | 2591(2) | 1867(3) | 92.0(12) |
| C51 | 6211(6) | 2585(3) | 849(3) | 131(2) |
| C52 | 6342(6) | 3165(3) | 357(3) | 134(2) |
| C53 | 6605(4) | 3748(2) | 887(3) | 100.4(15) |
| C54 | 6776(3) | 3752.9(18) | 1914(2) | 70.0(8) |
| C55 | 6499(3) | 2710.1(18) | 5604(3) | 71.7(8) |
| C56 | 7435(3) | 2549.3(15) | 5166(2) | 63.0(7) |
| C57 | 7951(4) | 1905(2) | 5750(3) | 89.7(11) |
| C58 | 8792(3) | 3473(2) | 6182(3) | 81.5(10) |
| C59 | 5306(2) | 4644.5(12) | 3160.4(19) | 48.3(5) |
| C60 | 4077(2) | 4639.6(13) | 2669.3(19) | 51.3(5) |
| C61 | 3647(3) | 4261.8(16) | 1809(2) | 66.9(7) |
| C62 | 2531(3) | 4267(2) | 1331(3) | 86.5(11) |
| C63 | 1859(3) | 4654(3) | 1708(3) | 94.1(13) |
| C64 | 2286(3) | 5029(3) | 2556(3) | 90.6(12) |
| C65 | 3399(2) | 5016.9(19) | 3047(3) | 69.7(8) |
| C66 | 4188(4) | 5876.9(18) | 1075(3) | 86.6(11) |
| C67 | 3186(4) | 6163(2) | 560(3) | 102.6(11) |
| C68 | 2763(4) | 6710(2) | 925(3) | 102.6(11) |
| C69 | 4774(3) | 6153.8(15) | 1985(2) | 65.5(7) |
| C70 | 4360(3) | 6695.0(18) | 2347(3) | 77.0(9) |
| C71 | 3366(4) | 6979(2) | 1807(3) | 96.1(12) |
| C72 | 1696(6) | 7023(3) | 313(6) | 162(3) |

| **Table 3 Anisotropic Displacement Parameters (Å^2^×10^3^) for bj02. The Anisotropic displacement factor exponent takes the form: -2π^2^[h^2^a*^2^U_11_+2hka*b*U_12_+…].** | | | | | | |
| --- | --- | --- | --- | --- | --- | --- |
| **Atom** | **U_11_** | **U_22_** | **U_33_** | **U_23_** | **U_13_** | **U_12_** |
| S1 | 77.8(4) | 51.6(3) | 54.6(3) | 7.2(3) | 14.4(3) | 1.0(3) |
| P1 | 49.5(3) | 44.9(3) | 46.1(3) | 2.1(2) | 16.1(2) | -3.8(2) |
| O1 | 53.4(10) | 46.6(9) | 62.9(10) | -1.5(8) | 23.4(8) | -3.7(7) |
| O2 | 51.7(9) | 47.5(8) | 48.9(8) | -4.9(7) | 13.2(7) | -5.1(7) |
| O3 | 53.8(9) | 57.3(10) | 51.0(9) | 5.6(8) | 14.8(7) | -6.6(8) |
| O4 | 102.0(18) | 72.7(14) | 55.8(12) | 6.3(10) | 2.1(11) | -13.0(13) |
| O5 | 89.6(16) | 62.6(12) | 92.9(17) | 12.3(11) | 27.1(13) | 19.0(11) |
| N1 | 68.1(13) | 46.6(11) | 49.3(11) | 3.4(9) | 19.8(10) | 2.0(10) |
| C1 | 53.3(13) | 50.0(13) | 54.4(14) | -5.6(10) | 12.5(11) | -4.8(11) |
| C2 | 48.0(13) | 65.8(16) | 66.1(16) | -2.3(13) | 19.3(12) | -7.3(12) |
| C3 | 59.0(15) | 55.8(14) | 69.5(16) | 1.9(12) | 27.7(13) | -10.9(12) |
| C4 | 64.3(16) | 46.1(13) | 71.1(17) | -4.5(12) | 24.8(14) | -9.0(11) |
| C5 | 57.6(14) | 46.9(13) | 56.9(14) | -4.1(10) | 20.8(12) | -8.2(11) |
| C6 | 82(2) | 58.7(16) | 58.7(16) | -13.2(12) | 30.8(15) | -16.8(14) |
| C7 | 137(4) | 66(2) | 94(3) | -25.6(18) | 65(3) | -28(2) |
| C8 | 197(6) | 104(3) | 114(3) | -55(3) | 102(4) | -56(4) |
| C9 | 193(6) | 131(4) | 84(3) | -38(3) | 81(4) | -64(4) |
| C10 | 167(5) | 102(3) | 62(2) | -8(2) | 45(3) | -54(3) |
| C11 | 103(3) | 70.2(18) | 56.2(16) | -9.0(14) | 24.7(17) | -26.4(18) |
| C12 | 75.6(19) | 60.9(17) | 82(2) | -14.4(15) | 24.4(16) | -21.8(15) |
| C13 | 58.1(16) | 74.0(19) | 86(2) | -5.8(16) | 16.1(15) | -15.5(15) |
| C14 | 84(2) | 73(2) | 99(3) | 9.5(18) | 36(2) | -23.3(17) |
| C15 | 65.3(17) | 65.4(17) | 69.2(18) | 11.6(14) | 19.1(14) | -7.4(14) |
| C16 | 64.8(18) | 86(2) | 95(2) | -4.0(18) | 42.4(18) | -6.0(16) |
| C17 | 62.3(15) | 61.0(16) | 63.3(16) | -4.0(13) | 5.6(13) | 0.0(13) |
| C18 | 88(2) | 71(2) | 65.9(18) | -6.5(15) | -5.9(16) | -0.8(17) |
| C19 | 133(4) | 99(3) | 76(2) | -6(2) | -24(2) | 11(3) |
| C20 | 164(5) | 108(4) | 110(4) | -7(3) | -48(4) | 57(4) |
| C21 | 154(5) | 95(3) | 112(4) | -28(3) | -40(3) | 58(3) |
| C22 | 95(3) | 80(2) | 70(2) | -13.5(17) | -10.9(18) | 26(2) |
| C23 | 67.3(16) | 45.2(12) | 44.6(12) | -0.1(9) | 20.3(11) | -4.9(11) |
| C24 | 69.3(16) | 46.6(13) | 63.0(15) | 9.9(11) | 31.0(14) | -0.9(11) |
| C25 | 101(3) | 60.4(17) | 96(2) | 11.7(16) | 58(2) | 13.8(17) |
| C26 | 113(4) | 90(3) | 144(4) | 23(3) | 84(3) | 30(3) |
| C27 | 73(2) | 103(3) | 182(5) | 48(3) | 62(3) | 19(2) |
| C28 | 64(2) | 87(3) | 139(4) | 13(3) | 27(2) | -4.8(19) |
| C29 | 65.8(18) | 74.0(19) | 86(2) | 5.1(17) | 26.2(16) | -5.6(15) |
| C30 | 86(2) | 51.3(14) | 49.1(14) | 10.1(11) | 21.3(13) | 0.1(13) |
| C31 | 89(2) | 76(2) | 73(2) | -5.2(16) | 21.2(18) | -8.3(18) |
| C32 | 100(3) | 100(3) | 97(3) | 3(2) | 18(2) | -23(2) |
| C33 | 78(2) | 114(4) | 119(4) | 46(3) | 29(3) | 9(2) |
| C34 | 116(3) | 66.1(18) | 71.1(19) | 9.2(15) | 44(2) | 6.8(18) |
| C35 | 124(4) | 99(3) | 96(3) | 28(3) | 57(3) | 30(3) |
| C36 | 85(3) | 222(8) | 184(6) | 58(6) | 36(4) | -6(4) |
| S2 | 77.5(4) | 56.5(4) | 70.7(4) | 3.9(3) | 39.3(4) | -6.7(3) |
| P2 | 42.8(3) | 49.1(3) | 47.7(3) | 1.6(2) | 17.6(2) | 3.9(2) |
| O6 | 44.6(8) | 56.3(9) | 54.9(9) | -6.6(8) | 19.6(7) | 5.0(7) |
| O7 | 45.5(9) | 52.2(9) | 59.8(10) | -1.8(8) | 13.6(8) | 5.1(7) |
| O8 | 53.0(9) | 62.3(10) | 56.2(10) | 7.4(8) | 26.8(8) | 9.8(8) |
| O9 | 128(2) | 75.2(14) | 104.5(19) | 7.9(13) | 83.6(19) | 4.6(14) |
| O10 | 80.9(15) | 70.0(14) | 101.9(18) | 2.0(12) | 32.8(14) | -24.3(12) |
| N2 | 59.8(12) | 51.7(11) | 50.0(11) | 0.8(9) | 23.6(10) | -3.4(9) |
| C37 | 47.9(13) | 71.4(17) | 72.9(17) | -3.5(14) | 25.4(12) | 1.7(12) |
| C38 | 65.5(17) | 81(2) | 99(2) | 3.5(18) | 45.7(17) | 11.4(16) |
| C39 | 80(2) | 111(3) | 140(4) | 13(3) | 73(3) | 13(2) |
| C40 | 103(3) | 101(3) | 204(6) | 10(4) | 102(4) | -11(3) |
| C41 | 84(3) | 84(3) | 162(4) | -16(3) | 67(3) | -21(2) |
| C42 | 58.3(17) | 78(2) | 100(3) | -12.8(17) | 35.7(17) | -11.7(15) |
| C43 | 48.0(12) | 54.0(13) | 54.0(13) | -5.2(11) | 18.0(11) | 6.9(10) |
| C44 | 49.9(14) | 72.9(17) | 57.1(15) | 0.2(13) | 12.4(12) | 13.6(13) |
| C45 | 57.3(16) | 78(2) | 85(2) | 6.2(17) | 20.9(15) | 23.1(15) |
| C46 | 80(2) | 70.5(19) | 76(2) | 0.9(15) | 27.5(17) | 29.7(17) |
| C47 | 61.3(15) | 53.0(14) | 65.3(16) | -1.5(12) | 16.1(13) | 9.3(12) |
| C48 | 49.1(13) | 53.6(13) | 52.4(13) | -3.7(10) | 15.9(11) | 6.6(10) |
| C49 | 68.0(17) | 67.1(17) | 51.9(14) | -3.3(12) | 17.3(13) | 18.5(14) |
| C50 | 134(4) | 70(2) | 61.7(18) | -11.4(16) | 15(2) | 22(2) |
| C51 | 207(6) | 100(3) | 64(2) | -24(2) | 10(3) | 50(4) |
| C52 | 215(7) | 127(4) | 53(2) | -4(2) | 32(3) | 70(4) |
| C53 | 137(4) | 109(3) | 65(2) | 18(2) | 46(2) | 56(3) |
| C54 | 81(2) | 77.0(19) | 59.5(16) | 3.6(14) | 32.4(15) | 24.3(16) |
| C55 | 72.9(19) | 74.1(19) | 72.7(19) | 13.3(15) | 29.0(16) | 8.3(15) |
| C56 | 63.0(16) | 63.5(16) | 61.7(15) | 9.0(13) | 17.7(13) | 14.2(13) |
| C57 | 101(3) | 84(2) | 84(2) | 26.5(19) | 27(2) | 32(2) |
| C58 | 68.1(19) | 96(3) | 67.4(19) | -2.7(17) | 0.2(15) | 9.0(18) |
| C59 | 49.2(12) | 50.9(12) | 49.4(13) | 0.6(10) | 22.0(10) | 2.6(10) |
| C60 | 53.4(13) | 53.1(13) | 48.5(13) | 8.8(10) | 17.2(11) | -0.4(11) |
| C61 | 71.0(17) | 58.9(16) | 64.4(16) | 5.4(13) | 10.8(14) | -1.8(14) |
| C62 | 80(2) | 86(2) | 75(2) | 15.2(18) | -3.9(17) | -20(2) |
| C63 | 50.7(17) | 124(3) | 96(3) | 41(3) | 3.6(18) | -8(2) |
| C64 | 52.5(17) | 126(3) | 97(3) | 18(3) | 27.9(18) | 12(2) |
| C65 | 54.4(15) | 89(2) | 68.1(18) | 6.2(16) | 22.6(13) | 8.6(15) |
| C66 | 135(3) | 61.9(19) | 61.3(18) | 0.9(14) | 28(2) | -16(2) |
| C67 | 121(3) | 84.5(18) | 81.2(19) | 15.0(15) | -0.7(17) | -27.9(19) |
| C68 | 120(3) | 83.8(18) | 83.0(19) | 16.4(15) | -0.1(17) | -26.9(19) |
| C69 | 96(2) | 52.0(14) | 52.5(15) | 5.6(11) | 27.7(15) | -9.4(14) |
| C70 | 93(2) | 63.4(18) | 71(2) | -3.7(15) | 18.8(17) | -1.7(17) |
| C71 | 112(3) | 72(2) | 98(3) | 7(2) | 21(3) | 10(2) |
| C72 | 123(5) | 127(5) | 186(7) | 39(5) | -27(5) | -12(4) |

| **Table 4 Bond Lengths for bj02.** | | | | | | |
| --- | --- | --- | --- | --- | --- | --- |
| **Atom** | **Atom** | **Length/Å** |  | **Atom** | **Atom** | **Length/Å** |
| S1 | O4 | 1.424(2) |  | S2 | O9 | 1.424(2) |
| S1 | O5 | 1.421(3) |  | S2 | O10 | 1.426(3) |
| S1 | N1 | 1.631(2) |  | S2 | N2 | 1.635(2) |
| S1 | C30 | 1.765(3) |  | S2 | C69 | 1.767(4) |
| P1 | O1 | 1.5820(18) |  | P2 | O6 | 1.5768(17) |
| P1 | O2 | 1.5781(18) |  | P2 | O7 | 1.5878(19) |
| P1 | O3 | 1.4631(19) |  | P2 | O8 | 1.4654(17) |
| P1 | C23 | 1.830(2) |  | P2 | C59 | 1.828(3) |
| O1 | C5 | 1.481(3) |  | O6 | C43 | 1.465(3) |
| O2 | C1 | 1.462(3) |  | O7 | C48 | 1.486(3) |
| N1 | C23 | 1.469(3) |  | N2 | C59 | 1.465(3) |
| C1 | C2 | 1.581(4) |  | C37 | C38 | 1.399(4) |
| C1 | C5 | 1.614(4) |  | C37 | C42 | 1.396(5) |
| C1 | C17 | 1.529(4) |  | C37 | C43 | 1.527(4) |
| C2 | C3 | 1.572(4) |  | C38 | C39 | 1.367(5) |
| C2 | C13 | 1.542(4) |  | C39 | C40 | 1.375(7) |
| C2 | C16 | 1.512(4) |  | C40 | C41 | 1.369(7) |
| C3 | C4 | 1.546(4) |  | C41 | C42 | 1.388(5) |
| C3 | C14 | 1.563(4) |  | C43 | C44 | 1.584(4) |
| C3 | C15 | 1.527(4) |  | C43 | C48 | 1.608(4) |
| C4 | C5 | 1.539(4) |  | C44 | C45 | 1.548(4) |
| C4 | C12 | 1.559(4) |  | C44 | C56 | 1.574(5) |
| C5 | C6 | 1.528(4) |  | C44 | C58 | 1.510(4) |
| C6 | C7 | 1.385(5) |  | C45 | C46 | 1.543(5) |
| C6 | C11 | 1.389(5) |  | C46 | C47 | 1.564(4) |
| C7 | C8 | 1.383(6) |  | C47 | C48 | 1.546(4) |
| C8 | C9 | 1.350(8) |  | C47 | C56 | 1.536(4) |
| C9 | C10 | 1.376(8) |  | C48 | C49 | 1.527(4) |
| C10 | C11 | 1.383(5) |  | C49 | C50 | 1.384(5) |
| C12 | C13 | 1.555(5) |  | C49 | C54 | 1.389(5) |
| C17 | C18 | 1.392(4) |  | C50 | C51 | 1.376(6) |
| C17 | C22 | 1.382(5) |  | C51 | C52 | 1.382(8) |
| C18 | C19 | 1.381(6) |  | C52 | C53 | 1.365(7) |
| C19 | C20 | 1.340(7) |  | C53 | C54 | 1.389(5) |
| C20 | C21 | 1.395(7) |  | C55 | C56 | 1.540(4) |
| C21 | C22 | 1.377(5) |  | C56 | C57 | 1.559(4) |
| C23 | C24 | 1.513(4) |  | C59 | C60 | 1.519(4) |
| C24 | C25 | 1.393(4) |  | C60 | C61 | 1.386(4) |
| C24 | C29 | 1.385(5) |  | C60 | C65 | 1.372(4) |
| C25 | C26 | 1.395(6) |  | C61 | C62 | 1.386(5) |
| C26 | C27 | 1.344(8) |  | C62 | C63 | 1.377(6) |
| C27 | C28 | 1.379(8) |  | C63 | C64 | 1.369(7) |
| C28 | C29 | 1.376(5) |  | C64 | C65 | 1.386(5) |
| C30 | C31 | 1.374(5) |  | C66 | C67 | 1.393(7) |
| C30 | C34 | 1.373(5) |  | C66 | C69 | 1.384(5) |
| C31 | C32 | 1.371(6) |  | C67 | C68 | 1.385(7) |
| C32 | C33 | 1.357(7) |  | C68 | C71 | 1.354(6) |
| C33 | C35 | 1.391(7) |  | C68 | C72 | 1.513(8) |
| C33 | C36 | 1.513(7) |  | C69 | C70 | 1.367(5) |
| C34 | C35 | 1.375(6) |  | C70 | C71 | 1.393(6) |

| **Table 5 Bond Angles for bj02.** | | | | | | | | |
| --- | --- | --- | --- | --- | --- | --- | --- | --- |
| **Atom** | **Atom** | **Atom** | **Angle/˚** |  | **Atom** | **Atom** | **Atom** | **Angle/˚** |
| O4 | S1 | N1 | 107.01(13) |  | O9 | S2 | O10 | 120.47(18) |
| O4 | S1 | C30 | 107.71(15) |  | O9 | S2 | N2 | 106.61(13) |
| O5 | S1 | O4 | 119.93(18) |  | O9 | S2 | C69 | 108.04(17) |
| O5 | S1 | N1 | 106.32(14) |  | O10 | S2 | N2 | 106.16(14) |
| O5 | S1 | C30 | 108.21(15) |  | O10 | S2 | C69 | 107.57(16) |
| N1 | S1 | C30 | 107.03(13) |  | N2 | S2 | C69 | 107.34(13) |
| O1 | P1 | C23 | 104.98(11) |  | O6 | P2 | O7 | 97.82(9) |
| O2 | P1 | O1 | 97.53(9) |  | O6 | P2 | C59 | 110.43(11) |
| O2 | P1 | C23 | 111.07(11) |  | O7 | P2 | C59 | 105.15(11) |
| O3 | P1 | O1 | 118.43(11) |  | O8 | P2 | O6 | 113.62(10) |
| O3 | P1 | O2 | 113.07(10) |  | O8 | P2 | O7 | 117.72(11) |
| O3 | P1 | C23 | 110.86(12) |  | O8 | P2 | C59 | 111.13(11) |
| C5 | O1 | P1 | 113.64(16) |  | C43 | O6 | P2 | 115.02(15) |
| C1 | O2 | P1 | 115.55(16) |  | C48 | O7 | P2 | 112.99(15) |
| C23 | N1 | S1 | 117.82(17) |  | C59 | N2 | S2 | 117.48(17) |
| O2 | C1 | C2 | 109.2(2) |  | C38 | C37 | C43 | 123.6(3) |
| O2 | C1 | C5 | 104.7(2) |  | C42 | C37 | C38 | 117.1(3) |
| O2 | C1 | C17 | 105.2(2) |  | C42 | C37 | C43 | 119.3(3) |
| C2 | C1 | C5 | 102.9(2) |  | C39 | C38 | C37 | 120.9(4) |
| C17 | C1 | C2 | 112.0(2) |  | C38 | C39 | C40 | 121.0(4) |
| C17 | C1 | C5 | 122.2(2) |  | C41 | C40 | C39 | 119.8(4) |
| C3 | C2 | C1 | 104.9(2) |  | C40 | C41 | C42 | 119.6(4) |
| C13 | C2 | C1 | 105.7(2) |  | C41 | C42 | C37 | 121.5(3) |
| C13 | C2 | C3 | 99.0(2) |  | O6 | C43 | C37 | 104.9(2) |
| C16 | C2 | C1 | 113.3(3) |  | O6 | C43 | C44 | 108.6(2) |
| C16 | C2 | C3 | 117.0(3) |  | O6 | C43 | C48 | 105.08(19) |
| C16 | C2 | C13 | 115.2(3) |  | C37 | C43 | C44 | 114.0(2) |
| C4 | C3 | C2 | 93.5(2) |  | C37 | C43 | C48 | 120.7(2) |
| C4 | C3 | C14 | 111.2(3) |  | C44 | C43 | C48 | 102.9(2) |
| C14 | C3 | C2 | 114.4(3) |  | C45 | C44 | C43 | 105.1(2) |
| C15 | C3 | C2 | 117.1(3) |  | C45 | C44 | C56 | 99.1(2) |
| C15 | C3 | C4 | 118.6(3) |  | C56 | C44 | C43 | 104.9(2) |
| C15 | C3 | C14 | 102.6(3) |  | C58 | C44 | C43 | 112.9(3) |
| C3 | C4 | C12 | 101.0(2) |  | C58 | C44 | C45 | 115.7(3) |
| C5 | C4 | C3 | 106.5(2) |  | C58 | C44 | C56 | 117.4(3) |
| C5 | C4 | C12 | 105.9(2) |  | C46 | C45 | C44 | 103.3(2) |
| O1 | C5 | C1 | 105.17(19) |  | C45 | C46 | C47 | 103.4(2) |
| O1 | C5 | C4 | 109.3(2) |  | C48 | C47 | C46 | 105.6(2) |
| O1 | C5 | C6 | 104.8(2) |  | C56 | C47 | C46 | 101.1(2) |
| C4 | C5 | C1 | 101.3(2) |  | C56 | C47 | C48 | 106.6(2) |
| C6 | C5 | C1 | 121.1(2) |  | O7 | C48 | C43 | 105.37(19) |
| C6 | C5 | C4 | 114.5(2) |  | O7 | C48 | C47 | 109.7(2) |
| C7 | C6 | C5 | 118.7(3) |  | O7 | C48 | C49 | 104.3(2) |
| C7 | C6 | C11 | 117.6(3) |  | C47 | C48 | C43 | 101.2(2) |
| C11 | C6 | C5 | 123.5(3) |  | C49 | C48 | C43 | 121.7(2) |
| C8 | C7 | C6 | 120.6(4) |  | C49 | C48 | C47 | 114.1(2) |
| C9 | C8 | C7 | 121.2(4) |  | C50 | C49 | C48 | 118.6(3) |
| C8 | C9 | C10 | 119.5(4) |  | C50 | C49 | C54 | 117.4(3) |
| C9 | C10 | C11 | 120.0(4) |  | C54 | C49 | C48 | 123.9(3) |
| C10 | C11 | C6 | 121.0(4) |  | C51 | C50 | C49 | 121.5(4) |
| C13 | C12 | C4 | 103.2(2) |  | C50 | C51 | C52 | 120.3(4) |
| C2 | C13 | C12 | 103.3(3) |  | C53 | C52 | C51 | 119.3(4) |
| C18 | C17 | C1 | 124.3(3) |  | C52 | C53 | C54 | 120.3(4) |
| C22 | C17 | C1 | 118.8(3) |  | C49 | C54 | C53 | 121.2(4) |
| C22 | C17 | C18 | 116.9(3) |  | C47 | C56 | C44 | 93.6(2) |
| C19 | C18 | C17 | 121.3(4) |  | C47 | C56 | C55 | 118.2(3) |
| C20 | C19 | C18 | 120.7(4) |  | C47 | C56 | C57 | 111.5(3) |
| C19 | C20 | C21 | 119.9(4) |  | C55 | C56 | C44 | 117.0(3) |
| C22 | C21 | C20 | 119.3(4) |  | C55 | C56 | C57 | 102.8(3) |
| C21 | C22 | C17 | 121.9(3) |  | C57 | C56 | C44 | 114.2(3) |
| N1 | C23 | P1 | 109.69(17) |  | N2 | C59 | P2 | 110.50(17) |
| N1 | C23 | C24 | 115.2(2) |  | N2 | C59 | C60 | 115.3(2) |
| C24 | C23 | P1 | 111.70(18) |  | C60 | C59 | P2 | 110.81(17) |
| C25 | C24 | C23 | 118.7(3) |  | C61 | C60 | C59 | 118.9(3) |
| C29 | C24 | C23 | 121.6(3) |  | C65 | C60 | C59 | 121.0(3) |
| C29 | C24 | C25 | 119.8(3) |  | C65 | C60 | C61 | 120.1(3) |
| C24 | C25 | C26 | 118.9(4) |  | C62 | C61 | C60 | 119.8(3) |
| C27 | C26 | C25 | 121.3(4) |  | C63 | C62 | C61 | 119.7(4) |
| C26 | C27 | C28 | 119.5(4) |  | C64 | C63 | C62 | 120.3(3) |
| C29 | C28 | C27 | 121.2(5) |  | C63 | C64 | C65 | 120.3(4) |
| C28 | C29 | C24 | 119.2(4) |  | C60 | C65 | C64 | 119.8(4) |
| C31 | C30 | S1 | 119.6(3) |  | C69 | C66 | C67 | 118.2(4) |
| C34 | C30 | S1 | 120.1(3) |  | C68 | C67 | C66 | 122.4(4) |
| C34 | C30 | C31 | 120.2(3) |  | C67 | C68 | C72 | 120.3(5) |
| C32 | C31 | C30 | 119.4(4) |  | C71 | C68 | C67 | 118.0(5) |
| C33 | C32 | C31 | 121.9(5) |  | C71 | C68 | C72 | 121.6(6) |
| C32 | C33 | C35 | 118.2(4) |  | C66 | C69 | S2 | 121.3(3) |
| C32 | C33 | C36 | 121.8(6) |  | C70 | C69 | S2 | 119.1(3) |
| C35 | C33 | C36 | 120.0(6) |  | C70 | C69 | C66 | 119.5(4) |
| C30 | C34 | C35 | 119.4(4) |  | C69 | C70 | C71 | 121.0(4) |
| C34 | C35 | C33 | 120.8(4) |  | C68 | C71 | C70 | 120.8(4) |

| **Table 6 Torsion Angles for bj02.** | | | | | | | | | | |
| --- | --- | --- | --- | --- | --- | --- | --- | --- | --- | --- |
| **A** | **B** | **C** | **D** | **Angle/˚** |  | **A** | **B** | **C** | **D** | **Angle/˚** |
| S1 | N1 | C23 | P1 | 149.07(15) |  | S2 | N2 | C59 | P2 | 151.16(14) |
| S1 | N1 | C23 | C24 | -83.9(3) |  | S2 | N2 | C59 | C60 | -82.3(2) |
| S1 | C30 | C31 | C32 | -176.2(3) |  | S2 | C69 | C70 | C71 | -179.4(3) |
| S1 | C30 | C34 | C35 | 174.6(3) |  | P2 | O6 | C43 | C37 | 119.0(2) |
| P1 | O1 | C5 | C1 | 15.4(2) |  | P2 | O6 | C43 | C44 | -118.8(2) |
| P1 | O1 | C5 | C4 | 123.49(19) |  | P2 | O6 | C43 | C48 | -9.2(2) |
| P1 | O1 | C5 | C6 | -113.3(2) |  | P2 | O7 | C48 | C43 | 15.3(2) |
| P1 | O2 | C1 | C2 | -117.3(2) |  | P2 | O7 | C48 | C47 | 123.57(19) |
| P1 | O2 | C1 | C5 | -7.7(2) |  | P2 | O7 | C48 | C49 | -113.9(2) |
| P1 | O2 | C1 | C17 | 122.3(2) |  | P2 | C59 | C60 | C61 | -95.0(3) |
| P1 | C23 | C24 | C25 | -97.8(3) |  | P2 | C59 | C60 | C65 | 86.9(3) |
| P1 | C23 | C24 | C29 | 83.5(3) |  | O6 | P2 | O7 | C48 | -19.34(17) |
| O1 | P1 | O2 | C1 | 15.63(18) |  | O6 | P2 | C59 | N2 | -46.50(19) |
| O1 | P1 | C23 | N1 | -149.13(17) |  | O6 | P2 | C59 | C60 | -175.54(16) |
| O1 | P1 | C23 | C24 | 81.9(2) |  | O6 | C43 | C44 | C45 | -173.8(2) |
| O1 | C5 | C6 | C7 | -92.6(4) |  | O6 | C43 | C44 | C56 | 82.3(3) |
| O1 | C5 | C6 | C11 | 82.9(4) |  | O6 | C43 | C44 | C58 | -46.8(3) |
| O2 | P1 | O1 | C5 | -18.60(17) |  | O6 | C43 | C48 | O7 | -3.8(3) |
| O2 | P1 | C23 | N1 | -44.7(2) |  | O6 | C43 | C48 | C47 | -118.0(2) |
| O2 | P1 | C23 | C24 | -173.72(17) |  | O6 | C43 | C48 | C49 | 114.3(2) |
| O2 | C1 | C2 | C3 | 81.6(3) |  | O7 | P2 | O6 | C43 | 17.05(18) |
| O2 | C1 | C2 | C13 | -174.3(2) |  | O7 | P2 | C59 | N2 | -151.06(16) |
| O2 | C1 | C2 | C16 | -47.1(3) |  | O7 | P2 | C59 | C60 | 79.91(18) |
| O2 | C1 | C5 | O1 | -4.6(2) |  | O7 | C48 | C49 | C50 | -90.5(3) |
| O2 | C1 | C5 | C4 | -118.4(2) |  | O7 | C48 | C49 | C54 | 84.1(3) |
| O2 | C1 | C5 | C6 | 113.6(3) |  | O8 | P2 | O6 | C43 | 141.97(17) |
| O2 | C1 | C17 | C18 | -159.1(3) |  | O8 | P2 | O7 | C48 | -141.27(16) |
| O2 | C1 | C17 | C22 | 22.0(4) |  | O8 | P2 | C59 | N2 | 80.53(19) |
| O3 | P1 | O1 | C5 | -140.01(16) |  | O8 | P2 | C59 | C60 | -48.5(2) |
| O3 | P1 | O2 | C1 | 140.96(17) |  | O9 | S2 | N2 | C59 | -39.2(2) |
| O3 | P1 | C23 | N1 | 81.9(2) |  | O9 | S2 | C69 | C66 | 19.4(3) |
| O3 | P1 | C23 | C24 | -47.1(2) |  | O9 | S2 | C69 | C70 | -162.1(3) |
| O4 | S1 | N1 | C23 | -43.2(3) |  | O10 | S2 | N2 | C59 | -168.8(2) |
| O4 | S1 | C30 | C31 | -163.1(3) |  | O10 | S2 | C69 | C66 | 150.9(3) |
| O4 | S1 | C30 | C34 | 19.4(3) |  | O10 | S2 | C69 | C70 | -30.6(3) |
| O5 | S1 | N1 | C23 | -172.4(2) |  | N2 | S2 | C69 | C66 | -95.2(3) |
| O5 | S1 | C30 | C31 | -32.1(3) |  | N2 | S2 | C69 | C70 | 83.2(3) |
| O5 | S1 | C30 | C34 | 150.4(3) |  | N2 | C59 | C60 | C61 | 138.5(2) |
| N1 | S1 | C30 | C31 | 82.1(3) |  | N2 | C59 | C60 | C65 | -39.5(3) |
| N1 | S1 | C30 | C34 | -95.4(3) |  | C37 | C38 | C39 | C40 | -0.1(7) |
| N1 | C23 | C24 | C25 | 136.2(3) |  | C37 | C43 | C44 | C45 | -57.3(3) |
| N1 | C23 | C24 | C29 | -42.5(4) |  | C37 | C43 | C44 | C56 | -161.2(2) |
| C1 | C2 | C3 | C4 | 49.5(2) |  | C37 | C43 | C44 | C58 | 69.7(3) |
| C1 | C2 | C3 | C14 | 164.6(3) |  | C37 | C43 | C48 | O7 | -121.8(2) |
| C1 | C2 | C3 | C15 | -75.3(3) |  | C37 | C43 | C48 | C47 | 124.0(3) |
| C1 | C2 | C13 | C12 | -67.7(3) |  | C37 | C43 | C48 | C49 | -3.7(4) |
| C1 | C5 | C6 | C7 | 149.0(3) |  | C38 | C37 | C42 | C41 | 1.1(6) |
| C1 | C5 | C6 | C11 | -35.5(4) |  | C38 | C37 | C43 | O6 | -162.2(3) |
| C1 | C17 | C18 | C19 | -177.8(4) |  | C38 | C37 | C43 | C44 | 79.2(4) |
| C1 | C17 | C22 | C21 | 176.8(5) |  | C38 | C37 | C43 | C48 | -44.1(4) |
| C2 | C1 | C5 | O1 | 109.5(2) |  | C38 | C39 | C40 | C41 | 0.4(9) |
| C2 | C1 | C5 | C4 | -4.3(3) |  | C39 | C40 | C41 | C42 | 0.0(9) |
| C2 | C1 | C5 | C6 | -132.3(2) |  | C40 | C41 | C42 | C37 | -0.8(7) |
| C2 | C1 | C17 | C18 | 82.4(4) |  | C42 | C37 | C38 | C39 | -0.6(5) |
| C2 | C1 | C17 | C22 | -96.6(4) |  | C42 | C37 | C43 | O6 | 18.3(4) |
| C2 | C3 | C4 | C5 | -54.1(3) |  | C42 | C37 | C43 | C44 | -100.3(3) |
| C2 | C3 | C4 | C12 | 56.3(2) |  | C42 | C37 | C43 | C48 | 136.4(3) |
| C3 | C2 | C13 | C12 | 40.7(3) |  | C43 | C37 | C38 | C39 | 179.9(4) |
| C3 | C4 | C5 | O1 | -72.9(3) |  | C43 | C37 | C42 | C41 | -179.4(4) |
| C3 | C4 | C5 | C1 | 37.7(3) |  | C43 | C44 | C45 | C46 | -68.2(3) |
| C3 | C4 | C5 | C6 | 169.8(3) |  | C43 | C44 | C56 | C47 | 49.2(2) |
| C3 | C4 | C12 | C13 | -32.6(3) |  | C43 | C44 | C56 | C55 | -75.1(3) |
| C4 | C5 | C6 | C7 | 27.3(5) |  | C43 | C44 | C56 | C57 | 164.8(3) |
| C4 | C5 | C6 | C11 | -157.3(3) |  | C43 | C48 | C49 | C50 | 150.9(3) |
| C4 | C12 | C13 | C2 | -5.4(3) |  | C43 | C48 | C49 | C54 | -34.5(4) |
| C5 | C1 | C2 | C3 | -29.2(3) |  | C44 | C43 | C48 | O7 | 109.8(2) |
| C5 | C1 | C2 | C13 | 74.9(3) |  | C44 | C43 | C48 | C47 | -4.5(2) |
| C5 | C1 | C2 | C16 | -158.0(3) |  | C44 | C43 | C48 | C49 | -132.1(2) |
| C5 | C1 | C17 | C18 | -40.2(5) |  | C44 | C45 | C46 | C47 | -5.0(3) |
| C5 | C1 | C17 | C22 | 140.9(3) |  | C45 | C44 | C56 | C47 | -59.2(2) |
| C5 | C4 | C12 | C13 | 78.2(3) |  | C45 | C44 | C56 | C55 | 176.5(3) |
| C5 | C6 | C7 | C8 | 178.2(4) |  | C45 | C44 | C56 | C57 | 56.4(3) |
| C5 | C6 | C11 | C10 | -177.9(4) |  | C45 | C46 | C47 | C48 | 78.0(3) |
| C6 | C7 | C8 | C9 | -1.7(9) |  | C45 | C46 | C47 | C56 | -33.0(3) |
| C7 | C6 | C11 | C10 | -2.4(6) |  | C46 | C47 | C48 | O7 | 179.9(2) |
| C7 | C8 | C9 | C10 | 0.5(10) |  | C46 | C47 | C48 | C43 | -69.1(3) |
| C8 | C9 | C10 | C11 | -0.4(9) |  | C46 | C47 | C48 | C49 | 63.4(3) |
| C9 | C10 | C11 | C6 | 1.4(8) |  | C46 | C47 | C56 | C44 | 56.1(3) |
| C11 | C6 | C7 | C8 | 2.5(7) |  | C46 | C47 | C56 | C55 | 179.5(3) |
| C12 | C4 | C5 | O1 | -179.9(2) |  | C46 | C47 | C56 | C57 | -61.6(4) |
| C12 | C4 | C5 | C1 | -69.3(3) |  | C47 | C48 | C49 | C50 | 29.2(4) |
| C12 | C4 | C5 | C6 | 62.8(3) |  | C47 | C48 | C49 | C54 | -156.2(3) |
| C13 | C2 | C3 | C4 | -59.6(2) |  | C48 | C43 | C44 | C45 | 75.2(3) |
| C13 | C2 | C3 | C14 | 55.6(3) |  | C48 | C43 | C44 | C56 | -28.8(2) |
| C13 | C2 | C3 | C15 | 175.7(2) |  | C48 | C43 | C44 | C58 | -157.8(3) |
| C14 | C3 | C4 | C5 | -172.0(3) |  | C48 | C47 | C56 | C44 | -54.0(3) |
| C14 | C3 | C4 | C12 | -61.5(3) |  | C48 | C47 | C56 | C55 | 69.4(3) |
| C15 | C3 | C4 | C5 | 69.4(3) |  | C48 | C47 | C56 | C57 | -171.7(3) |
| C15 | C3 | C4 | C12 | 179.9(3) |  | C48 | C49 | C50 | C51 | 177.4(4) |
| C16 | C2 | C3 | C4 | 176.0(3) |  | C48 | C49 | C54 | C53 | -177.2(3) |
| C16 | C2 | C3 | C14 | -68.8(4) |  | C49 | C50 | C51 | C52 | -0.2(9) |
| C16 | C2 | C3 | C15 | 51.3(3) |  | C50 | C49 | C54 | C53 | -2.6(5) |
| C16 | C2 | C13 | C12 | 166.3(3) |  | C50 | C51 | C52 | C53 | -2.2(10) |
| C17 | C1 | C2 | C3 | -162.2(2) |  | C51 | C52 | C53 | C54 | 2.1(9) |
| C17 | C1 | C2 | C13 | -58.1(3) |  | C52 | C53 | C54 | C49 | 0.3(7) |
| C17 | C1 | C2 | C16 | 69.0(3) |  | C54 | C49 | C50 | C51 | 2.5(7) |
| C17 | C1 | C5 | O1 | -123.7(3) |  | C56 | C44 | C45 | C46 | 40.0(3) |
| C17 | C1 | C5 | C4 | 122.4(3) |  | C56 | C47 | C48 | O7 | -73.1(3) |
| C17 | C1 | C5 | C6 | -5.5(4) |  | C56 | C47 | C48 | C43 | 37.9(3) |
| C17 | C18 | C19 | C20 | -0.4(9) |  | C56 | C47 | C48 | C49 | 170.3(2) |
| C18 | C17 | C22 | C21 | -2.2(7) |  | C58 | C44 | C45 | C46 | 166.6(3) |
| C18 | C19 | C20 | C21 | 0.7(12) |  | C58 | C44 | C56 | C47 | 175.5(3) |
| C19 | C20 | C21 | C22 | -1.7(12) |  | C58 | C44 | C56 | C55 | 51.2(4) |
| C20 | C21 | C22 | C17 | 2.5(10) |  | C58 | C44 | C56 | C57 | -69.0(4) |
| C22 | C17 | C18 | C19 | 1.2(7) |  | C59 | P2 | O6 | C43 | -92.38(18) |
| C23 | P1 | O1 | C5 | 95.65(18) |  | C59 | P2 | O7 | C48 | 94.38(18) |
| C23 | P1 | O2 | C1 | -93.66(18) |  | C59 | C60 | C61 | C62 | -177.9(3) |
| C23 | C24 | C25 | C26 | -176.9(3) |  | C59 | C60 | C65 | C64 | 176.8(3) |
| C23 | C24 | C29 | C28 | 176.8(3) |  | C60 | C61 | C62 | C63 | 0.7(5) |
| C24 | C25 | C26 | C27 | -1.1(6) |  | C61 | C60 | C65 | C64 | -1.2(5) |
| C25 | C24 | C29 | C28 | -1.9(5) |  | C61 | C62 | C63 | C64 | -0.5(6) |
| C25 | C26 | C27 | C28 | 0.5(7) |  | C62 | C63 | C64 | C65 | -0.6(6) |
| C26 | C27 | C28 | C29 | -0.5(7) |  | C63 | C64 | C65 | C60 | 1.5(6) |
| C27 | C28 | C29 | C24 | 1.3(6) |  | C65 | C60 | C61 | C62 | 0.1(4) |
| C29 | C24 | C25 | C26 | 1.8(5) |  | C66 | C67 | C68 | C71 | 1.3(7) |
| C30 | S1 | N1 | C23 | 72.1(2) |  | C66 | C67 | C68 | C72 | 177.0(5) |
| C30 | C31 | C32 | C33 | 0.8(7) |  | C66 | C69 | C70 | C71 | -1.0(6) |
| C30 | C34 | C35 | C33 | 2.5(6) |  | C67 | C66 | C69 | S2 | 178.1(3) |
| C31 | C30 | C34 | C35 | -2.8(5) |  | C67 | C66 | C69 | C70 | -0.3(5) |
| C31 | C32 | C33 | C35 | -1.1(7) |  | C67 | C68 | C71 | C70 | -2.5(7) |
| C31 | C32 | C33 | C36 | 178.9(5) |  | C69 | S2 | N2 | C59 | 76.4(2) |
| C32 | C33 | C35 | C34 | -0.5(6) |  | C69 | C66 | C67 | C68 | 0.1(6) |
| C34 | C30 | C31 | C32 | 1.2(5) |  | C69 | C70 | C71 | C68 | 2.5(7) |
| C36 | C33 | C35 | C34 | 179.5(5) |  | C72 | C68 | C71 | C70 | -178.2(5) |

| **Table 7 Hydrogen Atom Coordinates (Å×10^4^) and Isotropic Displacement Parameters (Å^2^×10^3^) for bj02.** | | | | |
| --- | --- | --- | --- | --- |
| **Atom** | ***x*** | ***y*** | ***z*** | **U(eq)** |
| H1 | 5909.64 | 4590.23 | 6289.12 | 65 |
| H4 | 6676.24 | 7889.44 | 7084.77 | 71 |
| H7 | 6458.84 | 7785.38 | 8543.76 | 111 |
| H8 | 6294.63 | 7763.21 | 10134.03 | 151 |
| H9 | 6465.85 | 6785.83 | 11006.18 | 153 |
| H10 | 6854.14 | 5804.23 | 10307.3 | 130 |
| H11 | 7029.79 | 5812.15 | 8717.67 | 92 |
| H12A | 8554.03 | 8149.34 | 7818.95 | 87 |
| H12B | 8439.41 | 7535.97 | 8494.1 | 87 |
| H13A | 9589.92 | 7531.82 | 7106.51 | 89 |
| H13B | 9573.53 | 6944.8 | 7859.28 | 89 |
| H14A | 7384.11 | 8411.87 | 5531.69 | 126 |
| H14B | 8278.2 | 7978.48 | 5268.77 | 126 |
| H14C | 8496.35 | 8264.33 | 6354.09 | 126 |
| H15A | 6202.9 | 6848.65 | 5227.21 | 100 |
| H15B | 6761.2 | 7276.28 | 4580 | 100 |
| H15C | 5947.78 | 7616.7 | 5069.76 | 100 |
| H16A | 8219.93 | 6286.63 | 5281.39 | 117 |
| H16B | 9335.72 | 6207.17 | 6128.82 | 117 |
| H16C | 9106.22 | 6852.03 | 5457.24 | 117 |
| H18 | 8993.96 | 6472.18 | 8995.12 | 98 |
| H19 | 10225.21 | 5736.96 | 9996.55 | 138 |
| H20 | 10487.95 | 4707.59 | 9423.58 | 177 |
| H21 | 9538.64 | 4399.13 | 7786.21 | 165 |
| H22 | 8266.28 | 5119.96 | 6795.83 | 107 |
| H23 | 5637.08 | 5496.95 | 7640.76 | 62 |
| H25 | 4112.01 | 5962.38 | 7924.8 | 96 |
| H26 | 2245.12 | 5916.6 | 7698.57 | 127 |
| H27 | 1116.76 | 5328.71 | 6411.16 | 137 |
| H28 | 1826.26 | 4748.1 | 5329.44 | 117 |
| H29 | 3668.77 | 4775.61 | 5512.53 | 89 |
| H31 | 4647.76 | 3153.03 | 6893.97 | 96 |
| H32 | 2976.95 | 2797.91 | 6967.97 | 122 |
| H34 | 4712.95 | 4543.88 | 8962.81 | 97 |
| H35 | 2997.07 | 4212.57 | 8970.29 | 121 |
| H36A | 1271.34 | 3081.47 | 7344.79 | 247 |
| H36B | 1320.91 | 3612.5 | 8182.51 | 247 |
| H36C | 1773.4 | 2886.45 | 8475.43 | 247 |
| H2 | 5915.28 | 5412.77 | 4140.25 | 63 |
| H38 | 8921.65 | 3474.3 | 3084.61 | 93 |
| H39 | 10024.48 | 4219.28 | 2613.53 | 122 |
| H40 | 10206.81 | 5303.44 | 3199.26 | 148 |
| H41 | 9283.66 | 5646.16 | 4289.87 | 124 |
| H42 | 8180.18 | 4897.46 | 4792.22 | 91 |
| H45A | 9507.35 | 3041.47 | 4491.38 | 89 |
| H45B | 9565.19 | 2456.22 | 5265.93 | 89 |
| H46A | 8492.56 | 1835.79 | 4016.87 | 90 |
| H46B | 8345.99 | 2445.26 | 3267.14 | 90 |
| H47 | 6617.46 | 2094.8 | 3760.77 | 73 |
| H50 | 6287.9 | 2196.49 | 2186.42 | 110 |
| H51 | 6006.88 | 2188.82 | 490.36 | 157 |
| H52 | 6250.74 | 3158.06 | -327.01 | 160 |
| H53 | 6671.37 | 4143.3 | 557.89 | 120 |
| H54 | 6951.29 | 4153.9 | 2265.12 | 84 |
| H55A | 5905.79 | 2403.77 | 5334.67 | 108 |
| H55B | 6250.8 | 3160.92 | 5430.23 | 108 |
| H55C | 6756.41 | 2666.04 | 6319.5 | 108 |
| H57A | 8221.94 | 2003.83 | 6452.35 | 135 |
| H57B | 8542.37 | 1755.28 | 5517.44 | 135 |
| H57C | 7406.96 | 1559.55 | 5638.46 | 135 |
| H58A | 9325.18 | 3790.38 | 6111.53 | 122 |
| H58B | 9139.53 | 3145.68 | 6679.46 | 122 |
| H58C | 8236.1 | 3702.09 | 6384.26 | 122 |
| H59 | 5634.47 | 4503.28 | 2646.37 | 58 |
| H61 | 4105.49 | 4005.27 | 1553.63 | 80 |
| H62 | 2237.37 | 4010.84 | 757.94 | 104 |
| H63 | 1110.42 | 4661.53 | 1385.28 | 113 |
| H64 | 1828.51 | 5292.34 | 2802.86 | 109 |
| H65 | 3685.46 | 5263.8 | 3631.33 | 84 |
| H66 | 4456.47 | 5509.75 | 813.58 | 104 |
| H67 | 2785.71 | 5979.26 | -51.2 | 123 |
| H70 | 4749.07 | 6876.41 | 2963.54 | 92 |
| H71 | 3112.43 | 7357.63 | 2055.15 | 115 |
| H72A | 1137.04 | 6923.39 | 616.41 | 243 |
| H72B | 1491.04 | 6843.67 | -354.55 | 243 |
| H72C | 1783.83 | 7500.05 | 287.01 | 243 |

| **Table 8 Solvent masks information for bj02.** | | | | | | |
| --- | --- | --- | --- | --- | --- | --- |
| **Number** | **X** | **Y** | **Z** | **Volume** | **Electron count** | **Content** |
| 1 | 0.000 | -0.883 | 1.000 | 404.5 | 12.9 | ? |
| 2 | 0.095 | 0.371 | 0.566 | 11.3 | 0.0 | ? |
| 3 | -0.095 | 0.871 | 0.434 | 11.3 | 0.0 | ? |

**Experimental**

Single crystals of C_36_H_38_NO_5_PS **[bj02]** were **[]**. A suitable crystal was selected and **[]** on a **XtaLAB Synergy R, DW system, HyPix** diffractometer. The crystal was kept at 293.15 K during data collection. Using Olex2 [1], the structure was solved with the SHELXS [2] structure solution program using Direct Methods and refined with the SHELXL [3] refinement package using Least Squares minimisation.

1. Dolomanov, O.V., Bourhis, L.J., Gildea, R.J, Howard, J.A.K. & Puschmann, H. (2009), J. Appl. Cryst. 42, 339-341.
2. Sheldrick, G.M. (2008). Acta Cryst. A64, 112-122.
3. Sheldrick, G.M. (2015). Acta Cryst. C71, 3-8.

**Refinement model description**

Number of restraints - 8, number of constraints - unknown.

Details:

1. Fixed Uiso
 At 1.2 times of:
 All C(H) groups, All C(H,H) groups, All N(H) groups
 At 1.5 times of:
 All C(H,H,H) groups
2. Rigid bond restraints
 C67, C68
 with sigma for 1-2 distances of 0.0008 and sigma for 1-3 distances of 0.0008
3. Uiso/Uaniso restraints and constraints
C67 ≈ C68: within 2A with sigma of 0.0007 and sigma for terminal atoms of
0.0014 within 2A
4.a Ternary CH refined with riding coordinates:
 C4(H4), C23(H23), C47(H47), C59(H59)
4.b Secondary CH2 refined with riding coordinates:
 C12(H12A,H12B), C13(H13A,H13B), C45(H45A,H45B), C46(H46A,H46B)
4.c Aromatic/amide H refined with riding coordinates:
 N1(H1), C7(H7), C8(H8), C9(H9), C10(H10), C11(H11), C18(H18), C19(H19),
 C20(H20), C21(H21), C22(H22), C25(H25), C26(H26), C27(H27), C28(H28), C29(H29),
 C31(H31), C32(H32), C34(H34), C35(H35), N2(H2), C38(H38), C39(H39), C40(H40),
 C41(H41), C42(H42), C50(H50), C51(H51), C52(H52), C53(H53), C54(H54), C61(H61),
 C62(H62), C63(H63), C64(H64), C65(H65), C66(H66), C67(H67), C70(H70), C71(H71)
4.d Idealised Me refined as rotating group:
 C14(H14A,H14B,H14C), C15(H15A,H15B,H15C), C16(H16A,H16B,H16C), C36(H36A,H36B,
 H36C), C55(H55A,H55B,H55C), C57(H57A,H57B,H57C), C58(H58A,H58B,H58C), C72(H72A,
 H72B,H72C)

This report has been created with Olex2, compiled on 2022.04.07 svn.rca3783a0 for OlexSys. Please [let us know](mailto:support@olex2.org?subject=Olex2%20Report) if there are any errors or if you would like to have additional features.
