# Supplementary material for: Stereoselective Pudovik reaction of aldehydes, aldimines, and nitroalkenes with CAMDOL-derived H-phosphonate
Source: Commun Chem. 2025 Nov 14;8:349. doi: 10.1038/s42004-025-01735-4 (PMC12618634; doi:10.1038/s42004-025-01735-4)

checkCIF/PLATON report

Structure factors have been supplied for datablock(s) bj02

THIS REPORT IS FOR GUIDANCE ONLY. IF USED AS PART OF A REVIEW PROCEDURE FOR PUBLICATION, IT SHOULD NOT REPLACE THE EXPERTISE OF AN EXPERIENCED CRYSTALLOGRAPHIC REFEREE.

No syntax errors found. CIF dictionary Interpreting this report

Datablock: bj02

```
Bond precision:   C-C = 0.0057 A          Wavelength=1.54184

Cell:           a=12.84397(17)   b=19.93976(19)      c=14.01120(18)
                alpha=90         beta=107.6368(14)   gamma=90
Temperature:    293 K
```

	Calculated	Reported
Volume	3419.68(8)	3419.68(8)
Space group	P 21	P 1 21 1
Hall group	P 2yb	P 2yb
Moiety formula	C36 H38 N O5 P S [+ solvent]	C36 H38 N O5 P S
Sum formula	C36 H38 N O5 P S [+ solvent]	C36 H38 N O5 P S
Mr	627.70	627.70
Dx,g cm-3	1.219	1.219
Z	4	4
Mu (mm-1)	1.615	1.615
F000	1328.0	1328.0
F000'	1334.08	
h,k,lmax	16,25,17	16,24,17
Nref	14405[7418]	12615
Tmin,Tmax	0.772,0.824	0.568,1.000
Tmin'	0.772	

```
Correction method= # Reported T Limits: Tmin=0.568 Tmax=1.000
AbsCorr = MULTI-SCAN

Data completeness= 1.70/0.88        Theta(max)= 76.679
```

```
R(reflections)= 0.0332( 12029)                      wR2(reflections)=
                                                    0.0972( 12615)
S = 1.069                 Npar= 801
```

The following ALERTS were generated. Each ALERT has the format
test-name_ALERT_alert-type_alert-level.
Click on the hyperlinks for more details of the test.

Alert level C

```
PLAT220_ALERT_2_C NonSolvent   Resd 1  C   Ueq(max)/Ueq(min) Range       3.2 Ratio
PLAT220_ALERT_2_C NonSolvent   Resd 2  C   Ueq(max)/Ueq(min) Range       3.4 Ratio
PLAT222_ALERT_3_C NonSolvent Resd 2  H   Uiso(max)/Uiso(min) Range       4.2 Ratio
PLAT230_ALERT_2_C Hirshfeld Test Diff for    C68      --C71      .       6.7 s.u.
PLAT241_ALERT_2_C High   'MainMol' Ueq as Compared to Neighbors of        C8 Check
PLAT241_ALERT_2_C High   'MainMol' Ueq as Compared to Neighbors of       C20 Check
PLAT241_ALERT_2_C High   'MainMol' Ueq as Compared to Neighbors of       C21 Check
PLAT241_ALERT_2_C High   'MainMol' Ueq as Compared to Neighbors of       C40 Check
PLAT241_ALERT_2_C High   'MainMol' Ueq as Compared to Neighbors of       C51 Check
PLAT241_ALERT_2_C High   'MainMol' Ueq as Compared to Neighbors of       C52 Check
PLAT242_ALERT_2_C Low    'MainMol' Ueq as Compared to Neighbors of       C33 Check
PLAT242_ALERT_2_C Low    'MainMol' Ueq as Compared to Neighbors of       C68 Check
PLAT334_ALERT_2_C Small <C-C> Benzene Dist.   C30      -C34      .      1.37 Ang.
PLAT340_ALERT_3_C Low Bond Precision on  C-C Bonds ...............   0.00572 Ang.
PLAT911_ALERT_3_C Missing FCF Refl Between Thmin & STh/L=    0.600         2 Report
                1  0  0,  -3 23  4,
PLAT915_ALERT_3_C No Flack x Check Done: Low Friedel Pair Coverage        78 %
```

Alert level G

```
PLAT003_ALERT_2_G Number of Uiso or Uij Restrained non-H Atoms ...         2 Report
PLAT007_ALERT_5_G Number of Unrefined Donor-H Atoms .............          2 Report
              H1    H2
PLAT142_ALERT_4_G s.u. on b - Axis Small or Missing .............    0.00019 Ang.
PLAT177_ALERT_4_G The CIF-Embedded .res File Contains DELU Records         1 Report
PLAT178_ALERT_4_G The CIF-Embedded .res File Contains SIMU Records         1 Report
PLAT188_ALERT_3_G A Non-default SIMU Restraint Value has been used    0.0007 Report
PLAT192_ALERT_3_G A Non-default DELU Restraint Value for First Par    0.0008 Report
PLAT192_ALERT_3_G A Non-default DELU Restraint Value for SecondPar    0.0008 Report
PLAT199_ALERT_1_G Reported _cell_measurement_temperature ..... (K)       293 Check
PLAT200_ALERT_1_G Reported   _diffrn_ambient_temperature ..... (K)       293 Check
PLAT605_ALERT_4_G Largest Solvent Accessible VOID in the Structure       363 A**3
PLAT860_ALERT_3_G Number of Least-Squares Restraints ............          8 Note
PLAT868_ALERT_4_G ALERTS Due to the Use of _smtbx_masks Suppressed         ! Info
PLAT912_ALERT_4_G Missing # of FCF Reflections Above STh/L=  0.600       215 Note
PLAT913_ALERT_3_G Missing # of Very Strong Reflections in FCF ....         1 Note
                1  0  0,
PLAT969_ALERT_5_G The 'Henn et al.' R-Factor-gap value ..........       5.52 Note
              Predicted wR2: Based on SigI**2  1.76 or SHELX Weight  9.40
PLAT978_ALERT_2_G Number C-C Bonds with Positive Residual Density.         3 Info
```

---

0 **ALERT level A** = Most likely a serious problem - resolve or explain
0 **ALERT level B** = A potentially serious problem, consider carefully
16 **ALERT level C** = Check. Ensure it is not caused by an omission or oversight

```
17 ALERT level G = General information/check it is not something unexpected

 2 ALERT type 1 CIF construction/syntax error, inconsistent or missing data
14 ALERT type 2 Indicator that the structure model may be wrong or deficient
 9 ALERT type 3 Indicator that the structure quality may be low
 6 ALERT type 4 Improvement, methodology, query or suggestion
 2 ALERT type 5 Informative message, check
```

It is advisable to attempt to resolve as many as possible of the alerts in all categories. Often the minor alerts point to easily fixed oversights, errors and omissions in your CIF or refinement strategy, so attention to these fine details can be worthwhile. In order to resolve some of the more serious problems it may be necessary to carry out additional measurements or structure refinements. However, the purpose of your study may justify the reported deviations and the more serious of these should normally be commented upon in the discussion or experimental section of a paper or in the "special_details" fields of the CIF. checkCIF was carefully designed to identify outliers and unusual parameters, but every test has its limitations and alerts that are not important in a particular case may appear. Conversely, the absence of alerts does not guarantee there are no aspects of the results needing attention. It is up to the individual to critically assess their own results and, if necessary, seek expert advice.

Publication of your CIF in IUCr journals

A basic structural check has been run on your CIF. These basic checks will be run on all CIFs submitted for publication in IUCr journals (*Acta Crystallographica*, *Journal of Applied Crystallography*, *Journal of Synchrotron Radiation*); however, if you intend to submit to *Acta Crystallographica Section C* or *E* or *IUCrData*, you should make sure that full publication checks are run on the final version of your CIF prior to submission.

Publication of your CIF in other journals

Please refer to the *Notes for Authors* of the relevant journal for any special instructions relating to CIF submission.

PLATON version of 06/01/2024; check.def file version of 05/01/2024

Datablock bj02 - ellipsoid plot

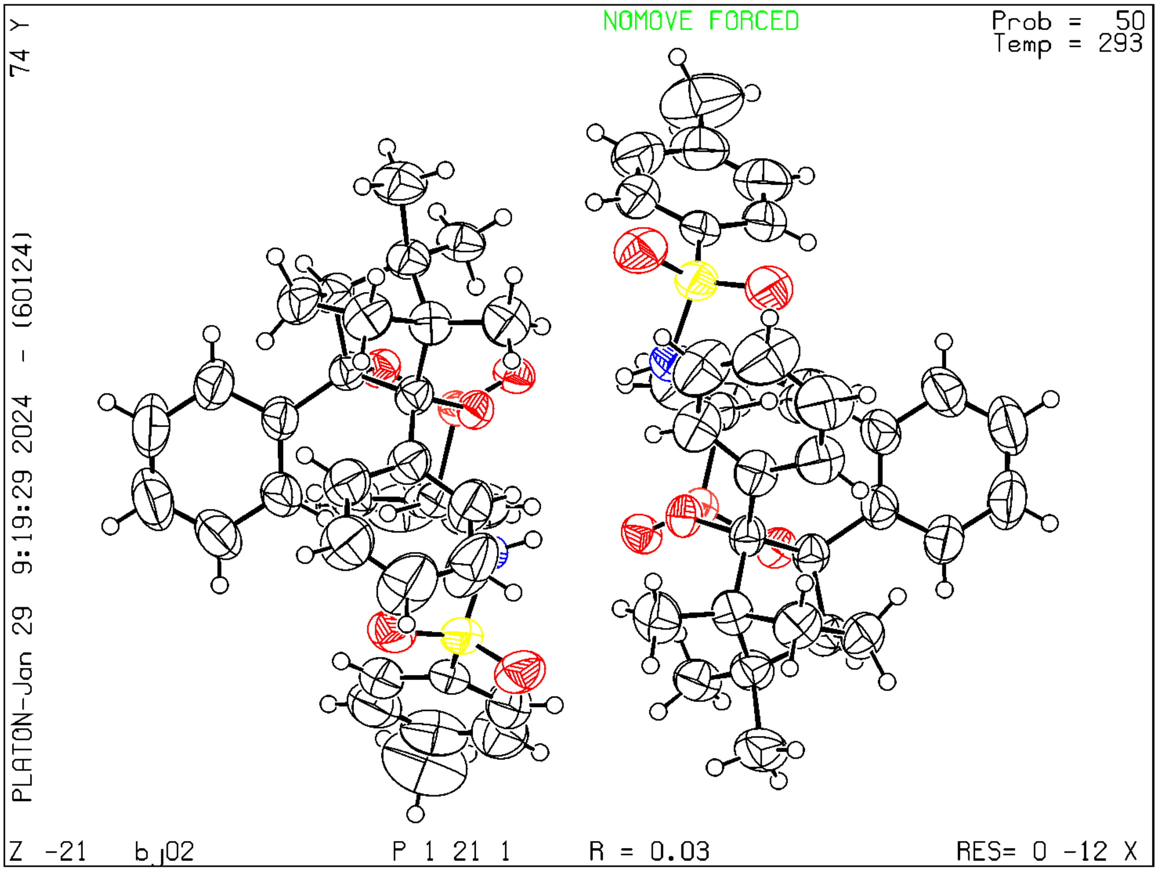

Supplement: Supplementary file 5 — Supplementary Data 3 [file 42004_2025_1735_MOESM5_ESM.zip › Supplementary Data 5-the cif file of 6a/checkcif.pdf]
