# Supplementary material for: Stereoselective Pudovik reaction of aldehydes, aldimines, and nitroalkenes with CAMDOL-derived H-phosphonate
Source: Commun Chem. 2025 Nov 14;8:349. doi: 10.1038/s42004-025-01735-4 (PMC12618634; doi:10.1038/s42004-025-01735-4)
Supplement: Supplementary file 6 — Supplementary Data 4 [file 42004_2025_1735_MOESM6_ESM.zip › Supplementary Data 6-the cif file of 9a/BJ03晶体学数据.docx]

**BJ03**

| **Table 1 Crystal data and structure refinement for BJ03.** | |
| --- | --- |
| Identification code | BJ03 |
| Empirical formula | C_30_H_32_NO_5_P |
| Formula weight | 517.53 |
| Temperature/K | 293.15 |
| Crystal system | orthorhombic |
| Space group | P2_1_2_1_2_1_ |
| a/Å | 11.3599(2) |
| b/Å | 29.0067(4) |
| c/Å | 25.1663(6) |
| α/° | 90 |
| β/° | 90 |
| γ/° | 90 |
| Volume/Å^3^ | 8292.6(3) |
| Z | 12 |
| ρ_calc_g/cm^3^ | 1.244 |
| μ/mm^‑1^ | 1.200 |
| F(000) | 3288.0 |
| Crystal size/mm^3^ | 0.15 × 0.12 × 0.1 |
| Radiation | Cu Kα (λ = 1.54184) |
| 2Θ range for data collection/° | 4.648 to 153.464 |
| Index ranges | -14 ≤ h ≤ 14, -35 ≤ k ≤ 27, -31 ≤ l ≤ 29 |
| Reflections collected | 51998 |
| Independent reflections | 16681 [R_int_ = 0.0462, R_sigma_ = 0.0417] |
| Data/restraints/parameters | 16681/7/1009 |
| Goodness-of-fit on F^2^ | 1.068 |
| Final R indexes [I>=2σ (I)] | R_1_ = 0.0862, wR_2_ = 0.2543 |
| Final R indexes [all data] | R_1_ = 0.0957, wR_2_ = 0.2624 |
| Largest diff. peak/hole / e Å^-3^ | 0.62/-0.38 |
| Flack/Hooft parameter | 0.054(8)/0.035(7) |

**Crystal structure determination of [BJ03]**

**Crystal Data** for C_30_H_32_NO_5_P (*M*=517.53 g/mol): orthorhombic, space group P2_1_2_1_2_1_ (no. 19), *a* = 11.3599(2) Å, *b* = 29.0067(4) Å, *c* = 25.1663(6) Å, *V*= 8292.6(3) Å^3^, *Z* = 12, *T* = 293.15 K, μ(Cu Kα) = 1.200 mm^-1^, *Dcalc* = 1.244 g/cm^3^, 51998 reflections measured (4.648° ≤ 2Θ ≤ 153.464°), 16681 unique (*R*_int_ = 0.0462, R_sigma_ = 0.0417) which were used in all calculations. The final *R*_1_ was 0.0862 (I > 2σ(I)) and *wR*_2_ was 0.2624 (all data).

**Refinement model description**

| **Table 2 Fractional Atomic Coordinates (×10^4^) and Equivalent Isotropic Displacement Parameters (Å^2^×10^3^) for BJ03. U_eq_ is defined as 1/3 of the trace of the orthogonalised U_IJ_ tensor.** | | | | |
| --- | --- | --- | --- | --- |
| **Atom** | ***x*** | ***y*** | ***z*** | **U(eq)** |
| P1 | 6019.6(8) | 5215.3(3) | 3224.9(4) | 47.6(2) |
| O1 | 5673(2) | 4786.7(9) | 3580.6(11) | 56.1(7) |
| O2 | 6746(2) | 5482.9(7) | 3670.5(10) | 48.6(6) |
| O3 | 5040(2) | 5479.7(10) | 2987.3(12) | 61.9(8) |
| O4 | 8040(4) | 4262.5(18) | 2130(2) | 140.5(18) |
| O5 | 7022(5) | 4532.9(19) | 1498.6(18) | 124.4(17) |
| N1 | 7219(4) | 4478.8(15) | 1964.7(19) | 88.1(13) |
| C1 | 7169(3) | 5205.4(12) | 4113.5(15) | 51.1(9) |
| C2 | 6656(4) | 5386.6(16) | 4653.5(18) | 65.4(12) |
| C3 | 7219(4) | 5105.6(18) | 5095.9(19) | 73.8(14) |
| C4 | 6728(5) | 4624.8(19) | 4995(2) | 82.3(15) |
| C5 | 5809(4) | 4692.8(16) | 4559.1(19) | 68.6(12) |
| C6 | 6497(4) | 4712.2(13) | 4035.8(17) | 57.2(10) |
| C8 | 8154(5) | 4208.6(15) | 3634(2) | 77.0(15) |
| C9 | 8558(6) | 3776.4(17) | 3475(2) | 97.1(18) |
| C10 | 7882(8) | 3391.8(15) | 3550(3) | 113(2) |
| C11 | 6772(7) | 3430.1(16) | 3790(2) | 96(2) |
| C12 | 6410(6) | 3856.1(16) | 3962(2) | 82.2(16) |
| C13 | 5392(5) | 5186.9(18) | 4676(2) | 76.2(14) |
| C14 | 4817(5) | 5216(2) | 5236(2) | 100(2) |
| C15 | 4412(4) | 5402.4(19) | 4301(2) | 85.4(16) |
| C16 | 6745(5) | 5900.7(17) | 4723(2) | 84.1(16) |
| C17 | 8514(3) | 5263.1(13) | 4092.0(16) | 52.3(9) |
| C18 | 9309(4) | 4955.4(18) | 4328(2) | 72.9(13) |
| C19 | 10497(4) | 5051.5(19) | 4330(2) | 79.7(15) |
| C20 | 10939(4) | 5437.7(19) | 4104(2) | 79.9(15) |
| C21 | 10171(4) | 5741.8(18) | 3869(2) | 72.5(13) |
| C22 | 8975(4) | 5660.2(14) | 3874.2(18) | 61.1(11) |
| C23 | 7098(4) | 4255.0(13) | 3890.5(18) | 63.7(12) |
| C24 | 6378(4) | 4687.8(15) | 2349.1(19) | 67.3(12) |
| C25 | 7053(3) | 5018.1(12) | 2724.1(16) | 51.0(9) |
| C26 | 7672(3) | 5402.3(13) | 2444.1(16) | 52.9(10) |
| C27 | 8854(3) | 5479.8(14) | 2524.5(19) | 62.0(11) |
| C28 | 9464(5) | 5823(2) | 2276(2) | 84.6(16) |
| C29 | 8889(5) | 6099(2) | 1907(3) | 97.7(18) |
| C30 | 7718(6) | 6026(2) | 1808(3) | 113(2) |
| C31 | 7083(4) | 5696.4(17) | 2080(2) | 75.0(13) |
| P40 | 4078.1(8) | 3529.5(3) | 1909.7(4) | 49.3(2) |
| O39 | 4483(2) | 3047.8(8) | 1682.2(11) | 53.1(7) |
| O41 | 3307(2) | 3689.5(8) | 1418.6(11) | 53.8(7) |
| O66 | 1961(6) | 2931(3) | 3276(3) | 171.5(5) |
| O67 | 3304(8) | 3182(2) | 3808(2) | 176(3) |
| O68 | 5047(3) | 3838.5(10) | 2068.1(13) | 66.9(8) |
| N65 | 2930(7) | 3097(2) | 3365(2) | 134.0(5) |
| C38 | 3769(3) | 2880.3(12) | 1238.2(16) | 51.7(9) |
| C42 | 2971(3) | 3312.8(13) | 1057.6(16) | 53.5(10) |
| C43 | 3463(5) | 3424.8(17) | 484.9(19) | 72.5(13) |
| C44 | 2996(5) | 3033(2) | 132.7(19) | 86.7(17) |
| C45 | 3699(6) | 2604.5(19) | 325(2) | 85.2(16) |
| C46 | 4503(4) | 2809.0(15) | 743.2(19) | 69.8(13) |
| C47 | 3235(4) | 2426.7(12) | 1465.7(17) | 60.5(11) |
| C48 | 3828(4) | 2016.8(13) | 1366(2) | 71.3(13) |
| C49 | 3413(5) | 1611.4(13) | 1601(2) | 84.9(17) |
| C50 | 2487(6) | 1606.3(15) | 1928(3) | 102(2) |
| C51 | 1919(6) | 2010.4(16) | 2053(3) | 94.7(19) |
| C52 | 2287(4) | 2419.3(13) | 1812(2) | 74.2(14) |
| C53 | 1627(4) | 3299.9(18) | 1073.9(17) | 66.7(12) |
| C54 | 968(5) | 2900(2) | 953(2) | 83.8(16) |
| C55 | -257(5) | 2924(3) | 952(2) | 115(3) |
| C56 | -822(5) | 3348(4) | 1061(3) | 139(3) |
| C57 | -185(5) | 3734(3) | 1168(2) | 113(2) |
| C58 | 1024(4) | 3707.9(19) | 1177.3(18) | 80.6(14) |
| C59 | 4792(4) | 3294.6(16) | 514(2) | 72.8(13) |
| C60 | 3131(6) | 3914(2) | 290(2) | 100.4(19) |
| C61 | 5359(6) | 3261(2) | -39(2) | 99.9(19) |
| C62 | 5629(5) | 3614.8(17) | 827(3) | 85.0(16) |
| C63 | 3072(4) | 3441.1(12) | 2469.5(18) | 56.0(10) |
| C64 | 3725(5) | 3185.0(17) | 2915(2) | 79.8(14) |
| C69 | 2559(3) | 3899.3(12) | 2642.2(16) | 51.1(9) |
| C70 | 3196(3) | 4229.6(14) | 2906.3(19) | 64.2(12) |
| C71 | 2721(4) | 4647.1(15) | 3051(2) | 79.5(15) |
| C72 | 1566(4) | 4744.4(16) | 2925(2) | 78.8(14) |
| C73 | 896(4) | 4419.1(17) | 2660(2) | 75.8(14) |
| C74 | 1397(4) | 4000.2(15) | 2525.9(19) | 63.2(11) |
| P78 | 5597.5(8) | 1861.0(3) | 3212.5(4) | 50.6(2) |
| O77 | 6253(2) | 2042.4(7) | 3723.4(11) | 52.6(7) |
| O79 | 5108(2) | 1401.6(9) | 3460.5(11) | 55.8(7) |
| O103 | 7883(5) | 1096(2) | 2051(3) | 162(2) |
| O104 | 6823(6) | 1367(3) | 1422(2) | 163(3) |
| O105 | 4714(3) | 2168.3(10) | 2978.1(12) | 67.1(8) |
| N102 | 7030(5) | 1299.7(19) | 1884(2) | 105.0(16) |
| C75 | 5832(4) | 1858.3(14) | 4673.7(16) | 60.1(11) |
| C76 | 6443(4) | 1701.3(12) | 4138.9(16) | 54.6(10) |
| C80 | 5663(4) | 1258.9(12) | 3962.7(17) | 56.7(10) |
| C81 | 4762(4) | 1243.7(16) | 4424(2) | 71.5(13) |
| C82 | 5451(6) | 1077.6(19) | 4908(2) | 90.0(17) |
| C83 | 6126(6) | 1513.0(18) | 5097(2) | 84.1(16) |
| C84 | 4495(4) | 1749.7(17) | 4581(2) | 71.2(13) |
| C85 | 7778(4) | 1661.6(15) | 4195.7(17) | 60.3(11) |
| C86 | 8322(5) | 1288(2) | 4438(2) | 81.3(15) |
| C87 | 9528(5) | 1283(3) | 4509(3) | 112(2) |
| C88 | 10216(5) | 1660(3) | 4326(2) | 109(2) |
| C89 | 9675(5) | 2023(2) | 4084(2) | 98.2(19) |
| C90 | 8457(4) | 2023.8(16) | 4024.0(18) | 65.8(12) |
| C91 | 6191(4) | 786.9(13) | 3805.9(19) | 65.0(12) |
| C92 | 7243(5) | 743.9(13) | 3540(2) | 75.0(14) |
| C93 | 7612(6) | 317.2(17) | 3351(3) | 96(2) |
| C94 | 6951(6) | -71.6(15) | 3439(3) | 102(2) |
| C95 | 5913(6) | -33.4(15) | 3713(3) | 100(2) |
| C96 | 5538(6) | 390.0(14) | 3894(2) | 85.1(16) |
| C97 | 6151(6) | 2364.4(17) | 4807(2) | 91.1(17) |
| C98 | 3753(5) | 1768(2) | 5100(2) | 100.8(19) |
| C99 | 3798(4) | 2048.5(17) | 4194(2) | 80.9(15) |
| C100 | 6734(3) | 1721.4(13) | 2721.7(17) | 56.8(10) |
| C101 | 6147(4) | 1460.5(18) | 2272(2) | 74.3(13) |
| C106 | 7376(3) | 2153.7(14) | 2558.9(16) | 54.8(10) |
| C107 | 6833(4) | 2516.9(15) | 2293(2) | 67.1(12) |
| C108 | 7390(6) | 2912.7(18) | 2173(3) | 89.8(18) |
| C109 | 8570(5) | 2959(2) | 2292(2) | 91.9(17) |
| C110 | 9147(4) | 2610(2) | 2544(2) | 85.4(16) |
| C111 | 8548(4) | 2205.5(18) | 2670(2) | 70.7(13) |

| **Table 3 Anisotropic Displacement Parameters (Å^2^×10^3^) for BJ03. The Anisotropic displacement factor exponent takes the form: -2π^2^[h^2^a*^2^U_11_+2hka*b*U_12_+…].** | | | | | | |
| --- | --- | --- | --- | --- | --- | --- |
| **Atom** | **U_11_** | **U_22_** | **U_33_** | **U_23_** | **U_13_** | **U_12_** |
| P1 | 41.3(4) | 38.0(3) | 63.3(5) | -2.2(4) | -5.5(4) | 0.2(3) |
| O1 | 50.3(13) | 49.2(12) | 68.9(15) | 1.6(12) | 3.0(12) | -7.1(11) |
| O2 | 43.7(11) | 37.5(10) | 64.6(14) | -2.1(10) | -9.0(11) | 2.7(9) |
| O3 | 47.6(13) | 59.4(14) | 78.7(17) | -2.7(13) | -10.7(13) | 6.3(11) |
| O4 | 105(3) | 154(3) | 162(4) | -67(3) | 1(3) | 55(2) |
| O5 | 134(4) | 148(4) | 91(3) | -26(3) | 29(3) | -25(3) |
| N1 | 85(3) | 88(2) | 91(3) | -34(2) | 15(2) | -19(2) |
| C1 | 55.4(19) | 45.5(16) | 52.6(18) | 1.3(15) | -6.9(16) | 6.2(15) |
| C2 | 64(2) | 69(2) | 64(2) | -9.5(19) | 3.4(19) | 1(2) |
| C3 | 66(2) | 93(3) | 62(2) | 0(2) | -1(2) | -8(2) |
| C4 | 81(3) | 98(3) | 68(3) | 16(2) | -2(2) | -19(3) |
| C5 | 58(2) | 76(2) | 72(2) | 6(2) | 4(2) | -20.1(19) |
| C6 | 56(2) | 51.8(18) | 63(2) | 1.5(17) | -0.7(18) | 5.5(16) |
| C8 | 110(4) | 48.1(19) | 73(3) | 7.4(19) | 8(3) | 17(2) |
| C9 | 137(4) | 69(2) | 86(3) | 10(2) | 18(3) | 41(3) |
| C10 | 201(7) | 40(2) | 97(4) | 7(2) | 2(4) | 30(3) |
| C11 | 147(5) | 47(2) | 95(3) | 11(2) | -19(4) | -9(3) |
| C12 | 106(4) | 58(2) | 83(3) | 4(2) | -8(3) | -2(2) |
| C13 | 74(3) | 82(3) | 73(3) | -3(2) | 20(2) | -7(2) |
| C14 | 79(3) | 127(5) | 94(4) | -17(3) | 16(3) | -16(3) |
| C15 | 57(2) | 85(3) | 114(4) | -12(3) | 14(3) | 9(2) |
| C16 | 90(3) | 73(3) | 89(3) | -30(2) | 8(3) | -8(2) |
| C17 | 43.2(16) | 56.1(18) | 57.4(19) | -4.4(16) | -6.4(15) | 2.2(15) |
| C18 | 58(2) | 77(3) | 84(3) | 9(2) | -15(2) | 3(2) |
| C19 | 44(2) | 96(3) | 99(3) | 0(3) | -7(2) | 12(2) |
| C20 | 43(2) | 96(3) | 100(3) | -6(3) | -4(2) | -4(2) |
| C21 | 62(2) | 82(3) | 74(3) | -6(2) | 7(2) | -19(2) |
| C22 | 56(2) | 53.6(19) | 74(2) | -2.9(18) | -4(2) | -5.2(17) |
| C23 | 78(3) | 46.1(18) | 67(2) | 5.7(17) | -4(2) | -1.6(18) |
| C24 | 63(2) | 58(2) | 81(3) | -18.7(19) | 2(2) | -9.9(18) |
| C25 | 48.9(17) | 38.9(15) | 65(2) | -6.3(15) | -4.8(16) | 0.9(14) |
| C26 | 49.2(18) | 49.2(17) | 60(2) | -3.4(16) | -5.6(16) | -2.3(15) |
| C27 | 42.8(18) | 61(2) | 82(3) | 0(2) | 3.5(18) | 2.1(17) |
| C28 | 59(2) | 99(3) | 95(3) | 7(3) | 7(2) | -13(3) |
| C29 | 88(3) | 99(3) | 106(4) | 25(3) | 12(3) | -28(3) |
| C30 | 103(4) | 135(4) | 101(3) | 53(3) | -29(3) | -29(4) |
| C31 | 49.1(19) | 88(3) | 88(3) | 27(2) | -21(2) | -14(2) |
| P40 | 43.8(4) | 34.8(3) | 69.3(5) | -4.5(4) | -3.2(4) | -3.3(3) |
| O39 | 41.7(11) | 40.8(11) | 76.7(16) | -10.6(11) | -3.9(11) | 6.1(9) |
| O41 | 54.1(13) | 35.7(10) | 71.5(15) | -3.8(11) | -3.4(12) | 5.2(10) |
| O66 | 162.0(7) | 195.1(10) | 157.4(10) | 58.8(10) | 43.3(9) | -5.2(8) |
| O67 | 244(7) | 189(5) | 94(3) | 37(3) | 32(4) | 48(5) |
| O68 | 56.2(14) | 57.4(14) | 87.2(19) | -12.2(13) | -0.7(14) | -20.8(11) |
| N65 | 166.7(7) | 119.9(10) | 115.5(10) | 55.9(9) | 42.7(9) | 5.3(8) |
| C38 | 50.1(18) | 41.9(16) | 63(2) | -4.4(15) | -3.3(16) | 2.8(14) |
| C42 | 49.9(18) | 50.9(17) | 60(2) | -6.9(16) | -5.1(16) | 3.8(15) |
| C43 | 75(3) | 80(3) | 63(2) | 2(2) | -2(2) | 8(2) |
| C44 | 84(3) | 127(4) | 49(2) | -7(3) | 4(2) | 0(3) |
| C45 | 104(4) | 86(3) | 66(3) | -16(2) | 11(3) | -7(3) |
| C46 | 71(3) | 58(2) | 81(3) | -9(2) | 5(2) | 4(2) |
| C47 | 73(2) | 38.9(16) | 69(2) | -5.6(16) | -14(2) | -7.1(16) |
| C48 | 74(3) | 42.4(17) | 97(3) | -14.7(19) | -12(2) | 2.2(18) |
| C49 | 109(4) | 30.5(16) | 115(4) | -7(2) | -21(3) | -3(2) |
| C50 | 128(4) | 35.4(18) | 143(5) | -6(2) | 14(4) | -23(2) |
| C51 | 113(4) | 54(2) | 117(4) | 1(3) | 17(3) | -25(2) |
| C52 | 80(3) | 38.4(16) | 105(3) | -12(2) | 13(3) | -12.3(17) |
| C53 | 49(2) | 94(3) | 57(2) | 1(2) | -8.8(17) | 2(2) |
| C54 | 68(3) | 120(4) | 64(2) | -9(3) | -7(2) | -9(3) |
| C55 | 55(2) | 223(7) | 68(3) | -11(4) | -11(2) | -32(4) |
| C56 | 51(3) | 283(10) | 83(4) | 20(5) | -7(3) | 30(4) |
| C57 | 57(2) | 206(6) | 77(3) | 26(4) | 10(2) | 44(3) |
| C58 | 67(2) | 112(3) | 63(2) | 21(2) | 4(2) | 35(2) |
| C59 | 74(3) | 68(2) | 76(3) | 2(2) | 21(2) | -1(2) |
| C60 | 106(4) | 115(4) | 80(3) | 30(3) | 13(3) | 34(3) |
| C61 | 102(4) | 100(4) | 97(3) | 6(3) | 45(3) | -2(3) |
| C62 | 65(3) | 62(2) | 128(4) | 8(3) | 22(3) | -1(2) |
| C63 | 51.0(19) | 39.7(16) | 77(2) | -0.5(16) | 6.2(18) | -3.4(14) |
| C64 | 92(3) | 68(2) | 80(3) | 19(2) | 8(3) | 23(2) |
| C69 | 45.0(17) | 49.9(17) | 58.4(19) | -3.0(15) | 1.6(15) | 2.1(15) |
| C70 | 38.6(17) | 64(2) | 90(3) | -16(2) | -6.6(18) | 12.3(16) |
| C71 | 72(3) | 54(2) | 113(3) | -29(2) | -8(3) | -8.7(19) |
| C72 | 65(2) | 65(2) | 107(3) | -24(2) | 1(2) | 17(2) |
| C73 | 47(2) | 85(3) | 95(3) | -12(3) | 0(2) | 6(2) |
| C74 | 43.1(18) | 68(2) | 78(3) | -13(2) | -3.6(18) | 1.7(17) |
| P78 | 48.9(4) | 42.4(4) | 60.5(5) | 6.1(4) | 0.6(4) | 8.2(3) |
| O77 | 63.7(14) | 32.0(10) | 62.0(14) | 4.5(10) | 2.2(12) | 5.8(10) |
| O79 | 47.3(12) | 50.8(12) | 69.4(15) | 3.7(12) | -3.1(12) | 1.0(11) |
| O103 | 114(3) | 204(5) | 169(5) | -41(4) | 9(3) | 79(3) |
| O104 | 152(5) | 245(6) | 92(3) | -46(4) | 29(3) | -7(5) |
| O105 | 59.5(14) | 69.5(15) | 72.5(16) | 14.4(13) | 5.6(13) | 27.8(12) |
| N102 | 91(3) | 130(3) | 95(3) | -46(2) | 17(2) | 8(3) |
| C75 | 66(2) | 57.6(19) | 56(2) | 4.7(17) | 7.0(18) | -2.2(19) |
| C76 | 65(2) | 37.1(15) | 62(2) | 3.1(15) | 3.7(18) | -2.9(15) |
| C80 | 58(2) | 40.2(16) | 72(2) | 5.9(16) | -2.5(18) | -4.1(15) |
| C81 | 62(2) | 70(2) | 83(3) | 11(2) | 14(2) | -13(2) |
| C82 | 108(4) | 91(3) | 72(3) | 30(2) | 12(3) | -7(3) |
| C83 | 100(4) | 90(3) | 63(2) | 15(2) | -1(3) | -8(3) |
| C84 | 59(2) | 77(3) | 77(3) | 8(2) | 16(2) | -3(2) |
| C85 | 53(2) | 67(2) | 61(2) | -3.2(19) | -10.5(18) | 5.9(18) |
| C86 | 67(3) | 98(3) | 79(3) | 6(3) | -13(2) | 19(2) |
| C87 | 76(3) | 159(5) | 101(4) | -2(4) | -27(3) | 54(3) |
| C88 | 51(2) | 178(6) | 97(4) | -44(4) | -11(2) | 23(3) |
| C89 | 62(3) | 141(5) | 91(3) | -36(3) | -2(3) | -3(3) |
| C90 | 57(2) | 73(2) | 68(2) | -15(2) | -5.3(19) | -7.0(19) |
| C91 | 71(2) | 40.4(16) | 84(3) | 9.9(17) | -18(2) | -2.2(17) |
| C92 | 88(3) | 37.7(17) | 100(3) | 4(2) | 7(3) | 8.7(19) |
| C93 | 124(5) | 55(2) | 109(4) | -1(3) | 10(4) | 16(3) |
| C94 | 129(5) | 31.5(18) | 146(5) | -1(3) | -17(4) | 7(2) |
| C95 | 123(4) | 39.9(19) | 136(4) | 17(2) | -30(4) | -10(3) |
| C96 | 103(3) | 39.7(18) | 113(4) | 17(2) | -20(3) | -10(2) |
| C97 | 116(4) | 72(3) | 85(3) | -22(2) | 26(3) | -4(3) |
| C98 | 92(3) | 125(4) | 86(3) | 1(3) | 43(2) | -1(3) |
| C99 | 65(2) | 74(3) | 104(3) | 3(3) | 25(2) | 8(2) |
| C100 | 49.6(18) | 56.2(19) | 64(2) | -3.2(17) | 1.7(17) | 12.4(16) |
| C101 | 58(2) | 81(3) | 84(3) | -22(2) | 8(2) | -2(2) |
| C106 | 39.4(16) | 65(2) | 59(2) | 1.1(17) | 5.2(15) | 2.4(16) |
| C107 | 45.0(19) | 67(2) | 89(3) | 17(2) | -4(2) | -3.3(18) |
| C108 | 94(4) | 69(3) | 106(4) | 14(3) | 8(3) | -2(3) |
| C109 | 92(3) | 84(3) | 99(4) | 5(3) | 22(3) | -21(3) |
| C110 | 53(2) | 103(3) | 100(3) | -12(3) | 11(2) | -8(2) |
| C111 | 49(2) | 83(3) | 80(3) | 5(2) | 1(2) | 4(2) |

| **Table 4 Bond Lengths for BJ03.** | | | | | | |
| --- | --- | --- | --- | --- | --- | --- |
| **Atom** | **Atom** | **Length/Å** |  | **Atom** | **Atom** | **Length/Å** |
| P1 | O1 | 1.582(3) |  | C47 | C52 | 1.385(7) |
| P1 | O2 | 1.594(3) |  | C48 | C49 | 1.399(6) |
| P1 | O3 | 1.478(3) |  | C49 | C50 | 1.336(9) |
| P1 | C25 | 1.815(4) |  | C50 | C51 | 1.374(7) |
| O1 | C6 | 1.495(5) |  | C51 | C52 | 1.396(7) |
| O2 | C1 | 1.456(4) |  | C53 | C54 | 1.414(8) |
| O4 | N1 | 1.199(7) |  | C53 | C58 | 1.391(7) |
| O5 | N1 | 1.205(7) |  | C54 | C55 | 1.394(7) |
| N1 | C24 | 1.489(6) |  | C55 | C56 | 1.413(12) |
| C1 | C2 | 1.569(6) |  | C56 | C57 | 1.361(12) |
| C1 | C6 | 1.633(5) |  | C57 | C58 | 1.376(7) |
| C1 | C17 | 1.538(5) |  | C59 | C61 | 1.536(8) |
| C2 | C3 | 1.521(7) |  | C59 | C62 | 1.545(8) |
| C2 | C13 | 1.549(7) |  | C63 | C64 | 1.536(6) |
| C2 | C16 | 1.505(7) |  | C63 | C69 | 1.515(5) |
| C3 | C4 | 1.524(7) |  | C69 | C70 | 1.372(6) |
| C4 | C5 | 1.527(7) |  | C69 | C74 | 1.383(6) |
| C5 | C6 | 1.532(6) |  | C70 | C71 | 1.374(6) |
| C5 | C13 | 1.538(7) |  | C71 | C72 | 1.380(7) |
| C6 | C23 | 1.536(6) |  | C72 | C73 | 1.383(7) |
| C8 | C9 | 1.394(7) |  | C73 | C74 | 1.384(7) |
| C8 | C23 | 1.368(7) |  | P78 | O77 | 1.577(3) |
| C9 | C10 | 1.367(9) |  | P78 | O79 | 1.573(3) |
| C10 | C11 | 1.403(11) |  | P78 | O105 | 1.466(3) |
| C11 | C12 | 1.372(7) |  | P78 | C100 | 1.832(4) |
| C12 | C23 | 1.408(7) |  | O77 | C76 | 1.456(4) |
| C13 | C14 | 1.556(8) |  | O79 | C80 | 1.472(5) |
| C13 | C15 | 1.588(8) |  | O103 | N102 | 1.211(8) |
| C17 | C18 | 1.402(6) |  | O104 | N102 | 1.202(7) |
| C17 | C22 | 1.379(6) |  | N102 | C101 | 1.476(7) |
| C18 | C19 | 1.379(7) |  | C75 | C76 | 1.582(6) |
| C19 | C20 | 1.352(8) |  | C75 | C83 | 1.499(7) |
| C20 | C21 | 1.375(7) |  | C75 | C84 | 1.568(6) |
| C21 | C22 | 1.378(6) |  | C75 | C97 | 1.549(7) |
| C24 | C25 | 1.548(6) |  | C76 | C80 | 1.622(5) |
| C25 | C26 | 1.494(5) |  | C76 | C85 | 1.527(6) |
| C26 | C27 | 1.376(6) |  | C80 | C81 | 1.548(6) |
| C26 | C31 | 1.419(6) |  | C80 | C91 | 1.546(5) |
| C27 | C28 | 1.365(7) |  | C81 | C82 | 1.527(7) |
| C28 | C29 | 1.389(8) |  | C81 | C84 | 1.550(7) |
| C29 | C30 | 1.370(9) |  | C82 | C83 | 1.552(8) |
| C30 | C31 | 1.380(8) |  | C84 | C98 | 1.555(7) |
| P40 | O39 | 1.579(2) |  | C84 | C99 | 1.525(7) |
| P40 | O41 | 1.584(3) |  | C85 | C86 | 1.389(7) |
| P40 | O68 | 1.474(3) |  | C85 | C90 | 1.373(6) |
| P40 | C63 | 1.832(4) |  | C86 | C87 | 1.382(8) |
| O39 | C38 | 1.464(5) |  | C87 | C88 | 1.420(10) |
| O41 | C42 | 1.471(5) |  | C88 | C89 | 1.364(10) |
| O66 | N65 | 1.221(10) |  | C89 | C90 | 1.391(7) |
| O67 | N65 | 1.217(9) |  | C91 | C92 | 1.375(7) |
| N65 | C64 | 1.471(8) |  | C91 | C96 | 1.387(6) |
| C38 | C42 | 1.613(5) |  | C92 | C93 | 1.391(7) |
| C38 | C46 | 1.513(6) |  | C93 | C94 | 1.373(8) |
| C38 | C47 | 1.558(5) |  | C94 | C95 | 1.370(10) |
| C42 | C43 | 1.580(6) |  | C95 | C96 | 1.377(7) |
| C42 | C53 | 1.529(6) |  | C100 | C101 | 1.516(6) |
| C43 | C44 | 1.535(8) |  | C100 | C106 | 1.507(6) |
| C43 | C59 | 1.558(7) |  | C106 | C107 | 1.392(6) |
| C43 | C60 | 1.549(8) |  | C106 | C111 | 1.369(6) |
| C44 | C45 | 1.556(8) |  | C107 | C108 | 1.346(7) |
| C45 | C46 | 1.514(7) |  | C108 | C109 | 1.379(9) |
| C46 | C59 | 1.557(7) |  | C109 | C110 | 1.363(8) |
| C47 | C48 | 1.390(6) |  | C110 | C111 | 1.393(8) |

| **Table 5 Bond Angles for BJ03.** | | | | | | | | |
| --- | --- | --- | --- | --- | --- | --- | --- | --- |
| **Atom** | **Atom** | **Atom** | **Angle/˚** |  | **Atom** | **Atom** | **Atom** | **Angle/˚** |
| O1 | P1 | O2 | 96.52(14) |  | C48 | C47 | C38 | 117.8(4) |
| O1 | P1 | C25 | 107.84(15) |  | C52 | C47 | C38 | 123.1(3) |
| O2 | P1 | C25 | 107.87(16) |  | C52 | C47 | C48 | 118.6(4) |
| O3 | P1 | O1 | 116.68(16) |  | C47 | C48 | C49 | 118.6(5) |
| O3 | P1 | O2 | 114.96(15) |  | C50 | C49 | C48 | 122.4(4) |
| O3 | P1 | C25 | 111.71(18) |  | C49 | C50 | C51 | 120.1(5) |
| C6 | O1 | P1 | 113.0(2) |  | C50 | C51 | C52 | 119.0(6) |
| C1 | O2 | P1 | 116.1(2) |  | C47 | C52 | C51 | 121.3(4) |
| O4 | N1 | O5 | 123.4(5) |  | C54 | C53 | C42 | 122.9(4) |
| O4 | N1 | C24 | 119.2(5) |  | C58 | C53 | C42 | 118.4(4) |
| O5 | N1 | C24 | 117.4(5) |  | C58 | C53 | C54 | 118.6(4) |
| O2 | C1 | C2 | 110.8(3) |  | C55 | C54 | C53 | 119.1(6) |
| O2 | C1 | C6 | 103.8(3) |  | C54 | C55 | C56 | 119.9(7) |
| O2 | C1 | C17 | 103.9(3) |  | C57 | C56 | C55 | 120.8(5) |
| C2 | C1 | C6 | 102.9(3) |  | C56 | C57 | C58 | 119.3(7) |
| C17 | C1 | C2 | 111.3(3) |  | C57 | C58 | C53 | 122.3(6) |
| C17 | C1 | C6 | 123.7(3) |  | C43 | C59 | C46 | 91.9(4) |
| C3 | C2 | C1 | 107.4(4) |  | C61 | C59 | C43 | 112.3(4) |
| C3 | C2 | C13 | 99.3(4) |  | C61 | C59 | C46 | 111.5(4) |
| C13 | C2 | C1 | 104.5(4) |  | C61 | C59 | C62 | 104.0(4) |
| C16 | C2 | C1 | 114.1(4) |  | C62 | C59 | C43 | 118.3(4) |
| C16 | C2 | C3 | 114.7(4) |  | C62 | C59 | C46 | 118.9(4) |
| C16 | C2 | C13 | 115.4(4) |  | C64 | C63 | P40 | 109.1(3) |
| C2 | C3 | C4 | 102.4(4) |  | C69 | C63 | P40 | 109.7(3) |
| C3 | C4 | C5 | 104.6(4) |  | C69 | C63 | C64 | 113.6(4) |
| C4 | C5 | C6 | 105.9(4) |  | N65 | C64 | C63 | 110.5(5) |
| C4 | C5 | C13 | 101.1(4) |  | C70 | C69 | C63 | 123.3(3) |
| C6 | C5 | C13 | 106.7(4) |  | C70 | C69 | C74 | 117.3(4) |
| O1 | C6 | C1 | 104.9(3) |  | C74 | C69 | C63 | 119.5(4) |
| O1 | C6 | C5 | 110.2(3) |  | C69 | C70 | C71 | 122.4(4) |
| O1 | C6 | C23 | 102.7(3) |  | C70 | C71 | C72 | 119.5(4) |
| C5 | C6 | C1 | 99.7(3) |  | C71 | C72 | C73 | 119.6(4) |
| C5 | C6 | C23 | 113.6(3) |  | C72 | C73 | C74 | 119.4(4) |
| C23 | C6 | C1 | 125.2(3) |  | C69 | C74 | C73 | 121.8(4) |
| C23 | C8 | C9 | 120.8(5) |  | O77 | P78 | C100 | 106.89(17) |
| C10 | C9 | C8 | 120.7(6) |  | O79 | P78 | O77 | 97.24(14) |
| C9 | C10 | C11 | 119.9(5) |  | O79 | P78 | C100 | 109.22(17) |
| C12 | C11 | C10 | 118.4(5) |  | O105 | P78 | O77 | 116.66(16) |
| C11 | C12 | C23 | 122.3(6) |  | O105 | P78 | O79 | 115.66(16) |
| C2 | C13 | C14 | 113.7(4) |  | O105 | P78 | C100 | 110.21(18) |
| C2 | C13 | C15 | 118.7(4) |  | C76 | O77 | P78 | 115.4(2) |
| C5 | C13 | C2 | 93.2(4) |  | C80 | O79 | P78 | 115.3(2) |
| C5 | C13 | C14 | 110.7(4) |  | O103 | N102 | C101 | 118.0(5) |
| C5 | C13 | C15 | 118.0(4) |  | O104 | N102 | O103 | 124.9(6) |
| C14 | C13 | C15 | 102.9(4) |  | O104 | N102 | C101 | 117.1(6) |
| C18 | C17 | C1 | 123.8(4) |  | C83 | C75 | C76 | 108.3(4) |
| C22 | C17 | C1 | 118.9(3) |  | C83 | C75 | C84 | 100.8(4) |
| C22 | C17 | C18 | 117.1(4) |  | C83 | C75 | C97 | 115.3(4) |
| C19 | C18 | C17 | 120.2(5) |  | C84 | C75 | C76 | 104.0(3) |
| C20 | C19 | C18 | 122.0(5) |  | C97 | C75 | C76 | 110.8(4) |
| C19 | C20 | C21 | 118.5(4) |  | C97 | C75 | C84 | 116.7(4) |
| C20 | C21 | C22 | 120.7(5) |  | O77 | C76 | C75 | 110.5(3) |
| C21 | C22 | C17 | 121.5(4) |  | O77 | C76 | C80 | 105.1(3) |
| C8 | C23 | C6 | 125.9(4) |  | O77 | C76 | C85 | 105.4(3) |
| C8 | C23 | C12 | 117.8(4) |  | C75 | C76 | C80 | 102.7(3) |
| C12 | C23 | C6 | 115.6(4) |  | C85 | C76 | C75 | 112.2(3) |
| N1 | C24 | C25 | 109.3(4) |  | C85 | C76 | C80 | 120.6(3) |
| C24 | C25 | P1 | 107.3(3) |  | O79 | C80 | C76 | 104.3(3) |
| C26 | C25 | P1 | 113.4(2) |  | O79 | C80 | C81 | 111.6(3) |
| C26 | C25 | C24 | 114.0(3) |  | O79 | C80 | C91 | 101.3(3) |
| C27 | C26 | C25 | 120.7(4) |  | C81 | C80 | C76 | 100.3(3) |
| C27 | C26 | C31 | 117.2(4) |  | C91 | C80 | C76 | 124.0(3) |
| C31 | C26 | C25 | 122.1(4) |  | C91 | C80 | C81 | 115.0(3) |
| C28 | C27 | C26 | 123.1(4) |  | C80 | C81 | C84 | 107.1(4) |
| C27 | C28 | C29 | 119.2(5) |  | C82 | C81 | C80 | 105.6(4) |
| C30 | C29 | C28 | 119.3(5) |  | C82 | C81 | C84 | 101.3(4) |
| C29 | C30 | C31 | 121.6(6) |  | C81 | C82 | C83 | 103.9(4) |
| C30 | C31 | C26 | 119.4(4) |  | C75 | C83 | C82 | 102.5(4) |
| O39 | P40 | O41 | 97.90(14) |  | C81 | C84 | C75 | 92.2(3) |
| O39 | P40 | C63 | 109.68(15) |  | C81 | C84 | C98 | 110.6(4) |
| O41 | P40 | C63 | 107.22(17) |  | C98 | C84 | C75 | 113.2(4) |
| O68 | P40 | O39 | 114.76(15) |  | C99 | C84 | C75 | 118.9(4) |
| O68 | P40 | O41 | 116.44(16) |  | C99 | C84 | C81 | 118.5(4) |
| O68 | P40 | C63 | 110.06(19) |  | C99 | C84 | C98 | 103.7(4) |
| C38 | O39 | P40 | 114.1(2) |  | C86 | C85 | C76 | 122.8(4) |
| C42 | O41 | P40 | 114.1(2) |  | C90 | C85 | C76 | 118.1(4) |
| O66 | N65 | O67 | 124.2(7) |  | C90 | C85 | C86 | 119.0(4) |
| O66 | N65 | C64 | 118.7(6) |  | C87 | C86 | C85 | 120.5(5) |
| O67 | N65 | C64 | 117.1(7) |  | C86 | C87 | C88 | 119.6(6) |
| O39 | C38 | C42 | 105.6(3) |  | C89 | C88 | C87 | 119.5(5) |
| O39 | C38 | C46 | 111.6(3) |  | C88 | C89 | C90 | 119.9(6) |
| O39 | C38 | C47 | 102.5(3) |  | C85 | C90 | C89 | 121.6(5) |
| C46 | C38 | C42 | 100.6(3) |  | C92 | C91 | C80 | 122.8(4) |
| C46 | C38 | C47 | 113.7(3) |  | C92 | C91 | C96 | 117.9(4) |
| C47 | C38 | C42 | 122.8(3) |  | C96 | C91 | C80 | 119.1(4) |
| O41 | C42 | C38 | 105.0(3) |  | C91 | C92 | C93 | 120.6(5) |
| O41 | C42 | C43 | 108.6(3) |  | C94 | C93 | C92 | 120.7(6) |
| O41 | C42 | C53 | 105.1(3) |  | C95 | C94 | C93 | 119.0(5) |
| C43 | C42 | C38 | 102.6(3) |  | C94 | C95 | C96 | 120.3(5) |
| C53 | C42 | C38 | 122.3(3) |  | C95 | C96 | C91 | 121.5(6) |
| C53 | C42 | C43 | 112.5(4) |  | C101 | C100 | P78 | 107.7(3) |
| C44 | C43 | C42 | 104.6(4) |  | C106 | C100 | P78 | 109.9(3) |
| C44 | C43 | C59 | 100.5(4) |  | C106 | C100 | C101 | 115.2(4) |
| C44 | C43 | C60 | 114.3(4) |  | N102 | C101 | C100 | 110.6(4) |
| C59 | C43 | C42 | 104.5(4) |  | C107 | C106 | C100 | 123.1(3) |
| C60 | C43 | C42 | 113.0(4) |  | C111 | C106 | C100 | 120.4(4) |
| C60 | C43 | C59 | 118.2(5) |  | C111 | C106 | C107 | 116.5(4) |
| C43 | C44 | C45 | 103.5(4) |  | C108 | C107 | C106 | 123.0(4) |
| C46 | C45 | C44 | 102.3(4) |  | C107 | C108 | C109 | 119.4(5) |
| C38 | C46 | C45 | 107.1(4) |  | C110 | C109 | C108 | 119.7(5) |
| C38 | C46 | C59 | 107.3(3) |  | C109 | C110 | C111 | 119.8(5) |
| C45 | C46 | C59 | 102.9(4) |  | C106 | C111 | C110 | 121.4(5) |

| **Table 6 Torsion Angles for BJ03.** | | | | | | | | | | |
| --- | --- | --- | --- | --- | --- | --- | --- | --- | --- | --- |
| **A** | **B** | **C** | **D** | **Angle/˚** |  | **A** | **B** | **C** | **D** | **Angle/˚** |
| P1 | O1 | C6 | C1 | 19.5(3) |  | C43 | C42 | C53 | C58 | -89.1(5) |
| P1 | O1 | C6 | C5 | 126.0(3) |  | C43 | C44 | C45 | C46 | -2.1(5) |
| P1 | O1 | C6 | C23 | -112.7(3) |  | C44 | C43 | C59 | C46 | -57.1(4) |
| P1 | O2 | C1 | C2 | -120.3(3) |  | C44 | C43 | C59 | C61 | 57.1(5) |
| P1 | O2 | C1 | C6 | -10.5(3) |  | C44 | C43 | C59 | C62 | 178.3(4) |
| P1 | O2 | C1 | C17 | 120.1(3) |  | C44 | C45 | C46 | C38 | 77.7(4) |
| P1 | C25 | C26 | C27 | 110.4(4) |  | C44 | C45 | C46 | C59 | -35.2(5) |
| P1 | C25 | C26 | C31 | -70.7(5) |  | C45 | C46 | C59 | C43 | 57.1(4) |
| O1 | P1 | O2 | C1 | 20.6(3) |  | C45 | C46 | C59 | C61 | -57.8(5) |
| O1 | P1 | C25 | C24 | 66.9(3) |  | C45 | C46 | C59 | C62 | -178.8(4) |
| O1 | P1 | C25 | C26 | -166.2(3) |  | C46 | C38 | C42 | O41 | -116.2(3) |
| O1 | C6 | C23 | C8 | 95.2(5) |  | C46 | C38 | C42 | C43 | -2.7(4) |
| O1 | C6 | C23 | C12 | -75.1(5) |  | C46 | C38 | C42 | C53 | 124.6(4) |
| O2 | P1 | O1 | C6 | -23.7(3) |  | C46 | C38 | C47 | C48 | 27.1(6) |
| O2 | P1 | C25 | C24 | 170.2(2) |  | C46 | C38 | C47 | C52 | -160.8(4) |
| O2 | P1 | C25 | C26 | -63.0(3) |  | C47 | C38 | C42 | O41 | 116.4(4) |
| O2 | C1 | C2 | C3 | -175.3(3) |  | C47 | C38 | C42 | C43 | -130.1(4) |
| O2 | C1 | C2 | C13 | 79.8(4) |  | C47 | C38 | C42 | C53 | -2.8(6) |
| O2 | C1 | C2 | C16 | -47.1(5) |  | C47 | C38 | C46 | C45 | 60.7(5) |
| O2 | C1 | C6 | O1 | -5.4(4) |  | C47 | C38 | C46 | C59 | 170.6(4) |
| O2 | C1 | C6 | C5 | -119.4(3) |  | C47 | C48 | C49 | C50 | -1.6(9) |
| O2 | C1 | C6 | C23 | 112.5(4) |  | C48 | C47 | C52 | C51 | -0.7(8) |
| O2 | C1 | C17 | C18 | -159.3(4) |  | C48 | C49 | C50 | C51 | -1.4(10) |
| O2 | C1 | C17 | C22 | 27.2(5) |  | C49 | C50 | C51 | C52 | 3.3(10) |
| O3 | P1 | O1 | C6 | -145.9(2) |  | C50 | C51 | C52 | C47 | -2.3(9) |
| O3 | P1 | O2 | C1 | 144.0(2) |  | C52 | C47 | C48 | C49 | 2.6(7) |
| O3 | P1 | C25 | C24 | -62.5(3) |  | C53 | C42 | C43 | C44 | -60.1(5) |
| O3 | P1 | C25 | C26 | 64.3(3) |  | C53 | C42 | C43 | C59 | -165.4(4) |
| O4 | N1 | C24 | C25 | 58.2(6) |  | C53 | C42 | C43 | C60 | 64.8(5) |
| O5 | N1 | C24 | C25 | -122.7(5) |  | C53 | C54 | C55 | C56 | 1.3(8) |
| N1 | C24 | C25 | P1 | -174.2(3) |  | C54 | C53 | C58 | C57 | 0.3(7) |
| N1 | C24 | C25 | C26 | 59.4(5) |  | C54 | C55 | C56 | C57 | -0.1(10) |
| C1 | C2 | C3 | C4 | -66.0(4) |  | C55 | C56 | C57 | C58 | -1.0(10) |
| C1 | C2 | C13 | C5 | 51.1(4) |  | C56 | C57 | C58 | C53 | 0.9(8) |
| C1 | C2 | C13 | C14 | 165.4(4) |  | C58 | C53 | C54 | C55 | -1.4(7) |
| C1 | C2 | C13 | C15 | -73.4(5) |  | C59 | C43 | C44 | C45 | 38.4(5) |
| C1 | C6 | C23 | C8 | -23.7(7) |  | C60 | C43 | C44 | C45 | 166.1(5) |
| C1 | C6 | C23 | C12 | 166.0(4) |  | C60 | C43 | C59 | C46 | 177.8(4) |
| C1 | C17 | C18 | C19 | -174.5(4) |  | C60 | C43 | C59 | C61 | -67.9(6) |
| C1 | C17 | C22 | C21 | 176.6(4) |  | C60 | C43 | C59 | C62 | 53.3(6) |
| C2 | C1 | C6 | O1 | 110.2(3) |  | C63 | P40 | O39 | C38 | 93.8(3) |
| C2 | C1 | C6 | C5 | -3.8(4) |  | C63 | P40 | O41 | C42 | -95.8(3) |
| C2 | C1 | C6 | C23 | -131.9(4) |  | C63 | C69 | C70 | C71 | 178.7(4) |
| C2 | C1 | C17 | C18 | 81.4(5) |  | C63 | C69 | C74 | C73 | -178.0(4) |
| C2 | C1 | C17 | C22 | -92.1(4) |  | C64 | C63 | C69 | C70 | 50.2(6) |
| C2 | C3 | C4 | C5 | -7.7(5) |  | C64 | C63 | C69 | C74 | -130.8(4) |
| C3 | C2 | C13 | C5 | -59.7(4) |  | C69 | C63 | C64 | N65 | 58.8(5) |
| C3 | C2 | C13 | C14 | 54.6(5) |  | C69 | C70 | C71 | C72 | -0.6(8) |
| C3 | C2 | C13 | C15 | 175.8(4) |  | C70 | C69 | C74 | C73 | 1.1(7) |
| C3 | C4 | C5 | C6 | 81.0(5) |  | C70 | C71 | C72 | C73 | 0.7(8) |
| C3 | C4 | C5 | C13 | -30.2(5) |  | C71 | C72 | C73 | C74 | 0.0(8) |
| C4 | C5 | C6 | O1 | -179.0(3) |  | C72 | C73 | C74 | C69 | -1.0(8) |
| C4 | C5 | C6 | C1 | -69.1(4) |  | C74 | C69 | C70 | C71 | -0.4(7) |
| C4 | C5 | C6 | C23 | 66.3(5) |  | P78 | O77 | C76 | C75 | -120.7(3) |
| C4 | C5 | C13 | C2 | 54.5(4) |  | P78 | O77 | C76 | C80 | -10.6(4) |
| C4 | C5 | C13 | C14 | -62.4(5) |  | P78 | O77 | C76 | C85 | 117.8(3) |
| C4 | C5 | C13 | C15 | 179.6(4) |  | P78 | O79 | C80 | C76 | 10.8(4) |
| C5 | C6 | C23 | C8 | -145.8(5) |  | P78 | O79 | C80 | C81 | 118.2(3) |
| C5 | C6 | C23 | C12 | 43.9(6) |  | P78 | O79 | C80 | C91 | -118.9(3) |
| C6 | C1 | C2 | C3 | 74.3(4) |  | P78 | C100 | C101 | N102 | -174.9(4) |
| C6 | C1 | C2 | C13 | -30.6(4) |  | P78 | C100 | C106 | C107 | -64.3(5) |
| C6 | C1 | C2 | C16 | -157.5(4) |  | P78 | C100 | C106 | C111 | 115.7(4) |
| C6 | C1 | C17 | C18 | -41.9(6) |  | O77 | P78 | O79 | C80 | -15.8(3) |
| C6 | C1 | C17 | C22 | 144.6(4) |  | O77 | P78 | C100 | C101 | 169.1(3) |
| C6 | C5 | C13 | C2 | -56.1(4) |  | O77 | P78 | C100 | C106 | -64.8(3) |
| C6 | C5 | C13 | C14 | -172.9(4) |  | O77 | C76 | C80 | O79 | -0.1(4) |
| C6 | C5 | C13 | C15 | 69.1(5) |  | O77 | C76 | C80 | C81 | -115.8(3) |
| C8 | C9 | C10 | C11 | -0.2(10) |  | O77 | C76 | C80 | C91 | 114.4(4) |
| C9 | C8 | C23 | C6 | -173.4(5) |  | O77 | C76 | C85 | C86 | -161.4(4) |
| C9 | C8 | C23 | C12 | -3.3(8) |  | O77 | C76 | C85 | C90 | 22.2(5) |
| C9 | C10 | C11 | C12 | -2.3(10) |  | O79 | P78 | O77 | C76 | 15.7(3) |
| C10 | C11 | C12 | C23 | 2.0(9) |  | O79 | P78 | C100 | C101 | 64.9(3) |
| C11 | C12 | C23 | C6 | 171.9(5) |  | O79 | P78 | C100 | C106 | -169.0(3) |
| C11 | C12 | C23 | C8 | 0.8(8) |  | O79 | C80 | C81 | C82 | 178.3(3) |
| C13 | C2 | C3 | C4 | 42.5(5) |  | O79 | C80 | C81 | C84 | -74.4(4) |
| C13 | C5 | C6 | O1 | -71.9(4) |  | O79 | C80 | C91 | C92 | 81.1(5) |
| C13 | C5 | C6 | C1 | 38.1(4) |  | O79 | C80 | C91 | C96 | -93.1(5) |
| C13 | C5 | C6 | C23 | 173.5(4) |  | O103 | N102 | C101 | C100 | 49.9(7) |
| C16 | C2 | C3 | C4 | 166.1(4) |  | O104 | N102 | C101 | C100 | -132.7(6) |
| C16 | C2 | C13 | C5 | 177.2(4) |  | O105 | P78 | O77 | C76 | 139.2(3) |
| C16 | C2 | C13 | C14 | -68.5(6) |  | O105 | P78 | O79 | C80 | -140.0(3) |
| C16 | C2 | C13 | C15 | 52.7(6) |  | O105 | P78 | C100 | C101 | -63.2(3) |
| C17 | C1 | C2 | C3 | -60.2(4) |  | O105 | P78 | C100 | C106 | 62.9(3) |
| C17 | C1 | C2 | C13 | -165.1(3) |  | C75 | C76 | C80 | O79 | 115.5(3) |
| C17 | C1 | C2 | C16 | 68.0(5) |  | C75 | C76 | C80 | C81 | -0.1(4) |
| C17 | C1 | C6 | O1 | -122.9(4) |  | C75 | C76 | C80 | C91 | -130.0(4) |
| C17 | C1 | C6 | C5 | 123.1(4) |  | C75 | C76 | C85 | C86 | 78.2(5) |
| C17 | C1 | C6 | C23 | -5.0(6) |  | C75 | C76 | C85 | C90 | -98.2(4) |
| C17 | C18 | C19 | C20 | -0.5(8) |  | C76 | C75 | C83 | C82 | -68.2(5) |
| C18 | C17 | C22 | C21 | 2.6(6) |  | C76 | C75 | C84 | C81 | 52.9(4) |
| C18 | C19 | C20 | C21 | 0.2(9) |  | C76 | C75 | C84 | C98 | 166.5(4) |
| C19 | C20 | C21 | C22 | 1.6(8) |  | C76 | C75 | C84 | C99 | -71.6(5) |
| C20 | C21 | C22 | C17 | -3.1(7) |  | C76 | C80 | C81 | C82 | -71.8(4) |
| C22 | C17 | C18 | C19 | -0.9(7) |  | C76 | C80 | C81 | C84 | 35.6(4) |
| C23 | C8 | C9 | C10 | 3.1(9) |  | C76 | C80 | C91 | C92 | -34.8(7) |
| C24 | C25 | C26 | C27 | -126.4(4) |  | C76 | C80 | C91 | C96 | 151.0(4) |
| C24 | C25 | C26 | C31 | 52.5(5) |  | C76 | C85 | C86 | C87 | -175.9(5) |
| C25 | P1 | O1 | C6 | 87.5(3) |  | C76 | C85 | C90 | C89 | 177.0(5) |
| C25 | P1 | O2 | C1 | -90.6(3) |  | C80 | C76 | C85 | C86 | -43.0(6) |
| C25 | C26 | C27 | C28 | 179.5(4) |  | C80 | C76 | C85 | C90 | 140.6(4) |
| C25 | C26 | C31 | C30 | -175.8(5) |  | C80 | C81 | C82 | C83 | 78.6(5) |
| C26 | C27 | C28 | C29 | -2.8(8) |  | C80 | C81 | C84 | C75 | -55.2(4) |
| C27 | C26 | C31 | C30 | 3.1(7) |  | C80 | C81 | C84 | C98 | -170.9(4) |
| C27 | C28 | C29 | C30 | 1.4(9) |  | C80 | C81 | C84 | C99 | 69.7(5) |
| C28 | C29 | C30 | C31 | 2.2(10) |  | C80 | C91 | C92 | C93 | -172.2(5) |
| C29 | C30 | C31 | C26 | -4.5(9) |  | C80 | C91 | C96 | C95 | 173.3(5) |
| C31 | C26 | C27 | C28 | 0.5(7) |  | C81 | C80 | C91 | C92 | -158.3(5) |
| P40 | O39 | C38 | C42 | 12.0(3) |  | C81 | C80 | C91 | C96 | 27.4(6) |
| P40 | O39 | C38 | C46 | 120.4(3) |  | C81 | C82 | C83 | C75 | -4.9(5) |
| P40 | O39 | C38 | C47 | -117.6(3) |  | C82 | C81 | C84 | C75 | 55.2(4) |
| P40 | O41 | C42 | C38 | -11.9(3) |  | C82 | C81 | C84 | C98 | -60.6(5) |
| P40 | O41 | C42 | C43 | -121.1(3) |  | C82 | C81 | C84 | C99 | 180.0(4) |
| P40 | O41 | C42 | C53 | 118.3(3) |  | C83 | C75 | C76 | O77 | -176.0(4) |
| P40 | C63 | C64 | N65 | -178.4(4) |  | C83 | C75 | C76 | C80 | 72.3(4) |
| P40 | C63 | C69 | C70 | -72.3(5) |  | C83 | C75 | C76 | C85 | -58.7(5) |
| P40 | C63 | C69 | C74 | 106.8(4) |  | C83 | C75 | C84 | C81 | -59.2(4) |
| O39 | P40 | O41 | C42 | 17.7(3) |  | C83 | C75 | C84 | C98 | 54.3(5) |
| O39 | P40 | C63 | C64 | 62.2(3) |  | C83 | C75 | C84 | C99 | 176.3(4) |
| O39 | P40 | C63 | C69 | -172.7(3) |  | C84 | C75 | C76 | O77 | 77.4(4) |
| O39 | C38 | C42 | O41 | 0.0(4) |  | C84 | C75 | C76 | C80 | -34.3(4) |
| O39 | C38 | C42 | C43 | 113.4(3) |  | C84 | C75 | C76 | C85 | -165.3(3) |
| O39 | C38 | C42 | C53 | -119.3(4) |  | C84 | C75 | C83 | C82 | 40.6(5) |
| O39 | C38 | C46 | C45 | 176.0(3) |  | C84 | C81 | C82 | C83 | -32.9(5) |
| O39 | C38 | C46 | C59 | -74.1(4) |  | C85 | C76 | C80 | O79 | -118.8(4) |
| O39 | C38 | C47 | C48 | -93.5(4) |  | C85 | C76 | C80 | C81 | 125.6(4) |
| O39 | C38 | C47 | C52 | 78.6(5) |  | C85 | C76 | C80 | C91 | -4.2(6) |
| O41 | P40 | O39 | C38 | -17.7(3) |  | C85 | C86 | C87 | C88 | -0.8(9) |
| O41 | P40 | C63 | C64 | 167.5(3) |  | C86 | C85 | C90 | C89 | 0.5(7) |
| O41 | P40 | C63 | C69 | -67.4(3) |  | C86 | C87 | C88 | C89 | 0.2(10) |
| O41 | C42 | C43 | C44 | -176.1(4) |  | C87 | C88 | C89 | C90 | 0.7(9) |
| O41 | C42 | C43 | C59 | 78.7(4) |  | C88 | C89 | C90 | C85 | -1.1(8) |
| O41 | C42 | C43 | C60 | -51.2(5) |  | C90 | C85 | C86 | C87 | 0.5(8) |
| O41 | C42 | C53 | C54 | -155.6(4) |  | C91 | C80 | C81 | C82 | 63.6(5) |
| O41 | C42 | C53 | C58 | 28.9(5) |  | C91 | C80 | C81 | C84 | 171.0(4) |
| O66 | N65 | C64 | C63 | 48.3(8) |  | C91 | C92 | C93 | C94 | -1.9(9) |
| O67 | N65 | C64 | C63 | -134.0(6) |  | C92 | C91 | C96 | C95 | -1.2(8) |
| O68 | P40 | O39 | C38 | -141.7(2) |  | C92 | C93 | C94 | C95 | 0.6(10) |
| O68 | P40 | O41 | C42 | 140.5(3) |  | C93 | C94 | C95 | C96 | 0.4(10) |
| O68 | P40 | C63 | C64 | -64.9(3) |  | C94 | C95 | C96 | C91 | 0.0(10) |
| O68 | P40 | C63 | C69 | 60.2(3) |  | C96 | C91 | C92 | C93 | 2.1(8) |
| C38 | C42 | C43 | C44 | 73.2(4) |  | C97 | C75 | C76 | O77 | -48.7(5) |
| C38 | C42 | C43 | C59 | -32.1(4) |  | C97 | C75 | C76 | C80 | -160.4(4) |
| C38 | C42 | C43 | C60 | -161.9(4) |  | C97 | C75 | C76 | C85 | 68.6(5) |
| C38 | C42 | C53 | C54 | -36.4(6) |  | C97 | C75 | C83 | C82 | 167.2(4) |
| C38 | C42 | C53 | C58 | 148.1(4) |  | C97 | C75 | C84 | C81 | 175.2(4) |
| C38 | C46 | C59 | C43 | -55.6(4) |  | C97 | C75 | C84 | C98 | -71.3(6) |
| C38 | C46 | C59 | C61 | -170.5(4) |  | C97 | C75 | C84 | C99 | 50.7(6) |
| C38 | C46 | C59 | C62 | 68.4(5) |  | C100 | P78 | O77 | C76 | -97.0(3) |
| C38 | C47 | C48 | C49 | 175.0(4) |  | C100 | P78 | O79 | C80 | 95.0(3) |
| C38 | C47 | C52 | C51 | -172.7(5) |  | C100 | C106 | C107 | C108 | 176.4(5) |
| C42 | C38 | C46 | C45 | -72.4(4) |  | C100 | C106 | C111 | C110 | -177.2(4) |
| C42 | C38 | C46 | C59 | 37.5(4) |  | C101 | C100 | C106 | C107 | 57.5(5) |
| C42 | C38 | C47 | C48 | 148.5(4) |  | C101 | C100 | C106 | C111 | -122.6(5) |
| C42 | C38 | C47 | C52 | -39.4(6) |  | C106 | C100 | C101 | N102 | 62.1(5) |
| C42 | C43 | C44 | C45 | -69.7(5) |  | C106 | C107 | C108 | C109 | 3.2(9) |
| C42 | C43 | C59 | C46 | 51.1(4) |  | C107 | C106 | C111 | C110 | 2.7(7) |
| C42 | C43 | C59 | C61 | 165.4(4) |  | C107 | C108 | C109 | C110 | -2.0(9) |
| C42 | C43 | C59 | C62 | -73.4(5) |  | C108 | C109 | C110 | C111 | 1.2(9) |
| C42 | C53 | C54 | C55 | -176.9(5) |  | C109 | C110 | C111 | C106 | -1.7(8) |
| C42 | C53 | C58 | C57 | 176.0(5) |  | C111 | C106 | C107 | C108 | -3.6(7) |
| C43 | C42 | C53 | C54 | 86.4(5) |  |  |  |  |  |  |

| **Table 7 Hydrogen Atom Coordinates (Å×10^4^) and Isotropic Displacement Parameters (Å^2^×10^3^) for BJ03.** | | | | |
| --- | --- | --- | --- | --- |
| **Atom** | ***x*** | ***y*** | ***z*** | **U(eq)** |
| H3A | 6988.46 | 5219.53 | 5442.97 | 89 |
| H3B | 8070.87 | 5108.93 | 5069.08 | 89 |
| H4A | 6368.74 | 4500.67 | 5314.07 | 99 |
| H4B | 7343.9 | 4416.71 | 4877.98 | 99 |
| H5 | 5176.89 | 4462.95 | 4562.6 | 82 |
| H8 | 8607.45 | 4468.7 | 3565.23 | 92 |
| H9 | 9293.92 | 3749.4 | 3316.48 | 117 |
| H10 | 8158.1 | 3104.96 | 3441.05 | 135 |
| H11 | 6291.99 | 3172.76 | 3831.67 | 116 |
| H12 | 5686.03 | 3882.26 | 4131.64 | 99 |
| H14A | 4065.68 | 5064.73 | 5229.89 | 150 |
| H14B | 4713.95 | 5533.33 | 5333.3 | 150 |
| H14C | 5317.97 | 5067.21 | 5491.6 | 150 |
| H15A | 4779.65 | 5541.06 | 3997.19 | 128 |
| H15B | 3979.06 | 5632.5 | 4493.51 | 128 |
| H15C | 3884.36 | 5163.87 | 4186.17 | 128 |
| H16A | 6343.44 | 5990.19 | 5042.47 | 126 |
| H16B | 6390.46 | 6051.96 | 4423.99 | 126 |
| H16C | 7558.68 | 5988.05 | 4747.3 | 126 |
| H18 | 9033.33 | 4685.48 | 4483.91 | 88 |
| H19 | 11010.56 | 4844.5 | 4490.86 | 96 |
| H20 | 11743.64 | 5496.33 | 4108.47 | 96 |
| H21 | 10460.8 | 6005.48 | 3704.35 | 87 |
| H22 | 8469.15 | 5877.65 | 3727.56 | 73 |
| H24A | 5998.67 | 4447.34 | 2555.08 | 81 |
| H24B | 5773.51 | 4856.85 | 2158.68 | 81 |
| H25 | 7655.5 | 4835.15 | 2906.62 | 61 |
| H27 | 9256.66 | 5289 | 2759.4 | 74 |
| H28 | 10254.58 | 5872.3 | 2353.32 | 102 |
| H29 | 9295.41 | 6329.91 | 1727.86 | 117 |
| H30 | 7340.99 | 6203.78 | 1551.82 | 136 |
| H31 | 6276.82 | 5667.05 | 2025.68 | 90 |
| H44A | 3146.43 | 3095.86 | -239.83 | 104 |
| H44B | 2156.86 | 2989.96 | 183.93 | 104 |
| H45A | 3181.5 | 2373.34 | 476.42 | 102 |
| H45B | 4146.86 | 2467.87 | 37.01 | 102 |
| H46 | 5207.25 | 2621.96 | 807.42 | 84 |
| H48 | 4487.55 | 2012.13 | 1146.45 | 86 |
| H49 | 3796.8 | 1335.39 | 1528.06 | 102 |
| H50 | 2226.21 | 1329.03 | 2071.43 | 123 |
| H51 | 1298.37 | 2011.43 | 2293.83 | 114 |
| H52 | 1887.5 | 2691.98 | 1884.64 | 89 |
| H54 | 1348.07 | 2624.06 | 874.51 | 101 |
| H55 | -701.16 | 2662.3 | 879.78 | 138 |
| H56 | -1640.23 | 3362.53 | 1059.13 | 167 |
| H57 | -562.45 | 4012.57 | 1233.79 | 136 |
| H58 | 1454.15 | 3972.2 | 1255.53 | 97 |
| H60A | 3568.54 | 4139.49 | 487.89 | 151 |
| H60B | 2303.37 | 3964.23 | 342.66 | 151 |
| H60C | 3314.03 | 3942.66 | -80.77 | 151 |
| H61A | 5456.04 | 3564.98 | -183.83 | 150 |
| H61B | 4860.8 | 3083.07 | -269.33 | 150 |
| H61C | 6113.26 | 3114.25 | -10.25 | 150 |
| H62A | 5960.13 | 3839.59 | 590.2 | 127 |
| H62B | 6249.09 | 3435.13 | 982.26 | 127 |
| H62C | 5196.25 | 3768.59 | 1102.5 | 127 |
| H63 | 2423.14 | 3244.8 | 2347.85 | 67 |
| H64A | 4025.94 | 2894.71 | 2780.1 | 96 |
| H64B | 4388.13 | 3368.61 | 3033.82 | 96 |
| H70 | 3977.5 | 4168.7 | 2990.65 | 77 |
| H71 | 3175.89 | 4862.31 | 3232.32 | 95 |
| H72 | 1238.75 | 5027.25 | 3017 | 95 |
| H73 | 116.36 | 4481.31 | 2573.32 | 91 |
| H74 | 940.39 | 3780.61 | 2352.83 | 76 |
| H81 | 4058.3 | 1060.16 | 4346.53 | 86 |
| H82A | 5993.16 | 832.88 | 4812.32 | 108 |
| H82B | 4924.61 | 966.26 | 5183.1 | 108 |
| H83A | 5852.8 | 1614.46 | 5442.39 | 101 |
| H83B | 6967.28 | 1456.71 | 5112.92 | 101 |
| H86 | 7871.73 | 1039.99 | 4553.71 | 98 |
| H87 | 9888.04 | 1033.51 | 4676.06 | 134 |
| H88 | 11028.29 | 1658.61 | 4371.29 | 130 |
| H89 | 10118.59 | 2269.66 | 3959.33 | 118 |
| H90 | 8095.31 | 2275.46 | 3863.74 | 79 |
| H92 | 7710.96 | 1002.62 | 3485.5 | 90 |
| H93 | 8313.94 | 294.94 | 3162.79 | 115 |
| H94 | 7204.23 | -356.87 | 3315.33 | 122 |
| H95 | 5459.42 | -294.54 | 3777.49 | 120 |
| H96 | 4831.23 | 410.43 | 4078.99 | 102 |
| H97A | 6966.4 | 2382.19 | 4905.98 | 137 |
| H97B | 5672.1 | 2469.62 | 5097.17 | 137 |
| H97C | 6013.65 | 2554.88 | 4501.68 | 137 |
| H98A | 4103.93 | 1570.97 | 5362.59 | 151 |
| H98B | 2966.33 | 1664.6 | 5026.88 | 151 |
| H98C | 3730.53 | 2078.48 | 5230.52 | 151 |
| H99A | 3661.74 | 2345.81 | 4349.92 | 121 |
| H99B | 3057.58 | 1903.58 | 4117.87 | 121 |
| H99C | 4237.2 | 2084.3 | 3870.65 | 121 |
| H100 | 7303.76 | 1514.34 | 2890.71 | 68 |
| H10A | 5723.3 | 1197.82 | 2415.16 | 89 |
| H10B | 5583.43 | 1659.79 | 2096.02 | 89 |
| H107 | 6048.22 | 2484.6 | 2194.66 | 80 |
| H108 | 6984.09 | 3152.97 | 2011.03 | 108 |
| H109 | 8969.36 | 3227.34 | 2200.48 | 110 |
| H110 | 9938.68 | 2641.52 | 2630.65 | 102 |
| H111 | 8952.85 | 1965.58 | 2833.4 | 85 |

**Experimental**

Single crystals of C_30_H_32_NO_5_P **[BJ03]** were **[]**. A suitable crystal was selected and **[]** on a **XtaLAB Synergy R, DW system, HyPix** diffractometer. The crystal was kept at 293.15 K during data collection. Using Olex2 [1], the structure was solved with the SHELXS [2] structure solution program using Direct Methods and refined with the SHELXL [3] refinement package using Least Squares minimisation.

1. Dolomanov, O.V., Bourhis, L.J., Gildea, R.J, Howard, J.A.K. & Puschmann, H. (2009), J. Appl. Cryst. 42, 339-341.
2. Sheldrick, G.M. (2008). Acta Cryst. A64, 112-122.
3. Sheldrick, G.M. (2015). Acta Cryst. C71, 3-8.

Number of restraints - 7, number of constraints - unknown.

Details:

1. Fixed Uiso
 At 1.2 times of:
 All C(H) groups, All C(H,H) groups
 At 1.5 times of:
 All C(H,H,H) groups
2. Rigid bond restraints
 N65, O66
 with sigma for 1-2 distances of 0.0009 and sigma for 1-3 distances of 0.0009
3. Uiso/Uaniso restraints and constraints
O66 ≈ N65: within 2A with sigma of 0.0006 and sigma for terminal atoms of
0.0012 within 2A
4.a Ternary CH refined with riding coordinates:
 C5(H5), C25(H25), C46(H46), C63(H63), C81(H81), C100(H100)
4.b Secondary CH2 refined with riding coordinates:
 C3(H3A,H3B), C4(H4A,H4B), C24(H24A,H24B), C44(H44A,H44B), C45(H45A,H45B),
 C64(H64A,H64B), C82(H82A,H82B), C83(H83A,H83B), C101(H10A,H10B)
4.c Aromatic/amide H refined with riding coordinates:
 C8(H8), C9(H9), C10(H10), C11(H11), C12(H12), C18(H18), C19(H19), C20(H20),
 C21(H21), C22(H22), C27(H27), C28(H28), C29(H29), C30(H30), C31(H31), C48(H48),
 C49(H49), C50(H50), C51(H51), C52(H52), C54(H54), C55(H55), C56(H56),
 C57(H57), C58(H58), C70(H70), C71(H71), C72(H72), C73(H73), C74(H74), C86(H86),
 C87(H87), C88(H88), C89(H89), C90(H90), C92(H92), C93(H93), C94(H94),
 C95(H95), C96(H96), C107(H107), C108(H108), C109(H109), C110(H110), C111(H111)
4.d Idealised Me refined as rotating group:
 C14(H14A,H14B,H14C), C15(H15A,H15B,H15C), C16(H16A,H16B,H16C), C60(H60A,H60B,
 H60C), C61(H61A,H61B,H61C), C62(H62A,H62B,H62C), C97(H97A,H97B,H97C), C98(H98A,
 H98B,H98C), C99(H99A,H99B,H99C)

This report has been created with Olex2, compiled on 2022.04.07 svn.rca3783a0 for OlexSys. Please [let us know](mailto:support@olex2.org?subject=Olex2%20Report) if there are any errors or if you would like to have additional features.
