# Supplementary material for: Stereoselective Pudovik reaction of aldehydes, aldimines, and nitroalkenes with CAMDOL-derived H-phosphonate
Source: Commun Chem. 2025 Nov 14;8:349. doi: 10.1038/s42004-025-01735-4 (PMC12618634; doi:10.1038/s42004-025-01735-4)

checkCIF/PLATON report

You have not supplied any structure factors. As a result the full set of tests cannot be run.

THIS REPORT IS FOR GUIDANCE ONLY. IF USED AS PART OF A REVIEW PROCEDURE FOR PUBLICATION, IT SHOULD NOT REPLACE THE EXPERTISE OF AN EXPERIENCED CRYSTALLOGRAPHIC REFEREE.

No syntax errors found.   CIF dictionary   Interpreting this report

Datablock: bj03

```
Bond precision:   C-C = 0.0070 A          Wavelength=1.54184

Cell:              a=11.3599(2)      b=29.0067(4)      c=25.1663(6)
                   alpha=90          beta=90           gamma=90
Temperature:       293 K

                  Calculated                  Reported
Volume            8292.6(3)                   8292.6(3)
Space group       P 21 21 21                  P 21 21 21
Hall group        P 2ac 2ab                   P 2ac 2ab
Moiety formula    C30 H32 N O5 P              C30 H32 N O5 P
Sum formula       C30 H32 N O5 P              C30 H32 N O5 P
Mr                517.54                      517.53
Dx,g cm-3         1.244                       1.244
Z                 12                          12
Mu (mm-1)         1.200                       1.200
F000              3288.0                      3288.0
F000'             3300.99
h,k,lmax          14,36,31                    14,35,31
Nref              17486[ 9565]                16681
Tmin,Tmax         0.841,0.887                 0.427,1.000
Tmin'             0.835

Correction method= # Reported T Limits: Tmin=0.427 Tmax=1.000
AbsCorr = MULTI-SCAN

Data completeness= 1.74/0.95       Theta(max)= 76.732

R(reflections)= 0.0862( 14202)                         wR2(reflections)=
                                                       0.2624( 16681)
S = 1.068                    Npar= 1009
```

The following ALERTS were generated. Each ALERT has the format
test-name_ALERT_alert-type_alert-level.
Click on the hyperlinks for more details of the test.

Alert level C

```
PLAT084_ALERT_3_C High wR2 Value (i.e. > 0.25) ...................       0.26 Report
PLAT220_ALERT_2_C NonSolvent   Resd 2  O   Ueq(max)/Ueq(min) Range        3.3 Ratio
PLAT220_ALERT_2_C NonSolvent   Resd 3  O   Ueq(max)/Ueq(min) Range        3.1 Ratio
PLAT230_ALERT_2_C Hirshfeld Test Diff for    C1       --C17      .        6.5 s.u.
PLAT230_ALERT_2_C Hirshfeld Test Diff for    C76      --C85      .        5.3 s.u.
PLAT234_ALERT_4_C Large Hirshfeld Difference C56      --C57      .       0.18 Ang.
PLAT241_ALERT_2_C High   'MainMol' Ueq as Compared to Neighbors of        C10 Check
PLAT241_ALERT_2_C High   'MainMol' Ueq as Compared to Neighbors of        C30 Check
PLAT241_ALERT_2_C High   'MainMol' Ueq as Compared to Neighbors of        C56 Check
PLAT241_ALERT_2_C High   'MainMol' Ueq as Compared to Neighbors of        C87 Check
PLAT242_ALERT_2_C Low    'MainMol' Ueq as Compared to Neighbors of         N1 Check
PLAT242_ALERT_2_C Low    'MainMol' Ueq as Compared to Neighbors of       N102 Check
PLAT340_ALERT_3_C Low Bond Precision on  C-C Bonds ...............    0.00698 Ang.
PLAT601_ALERT_2_C Unit Cell Contains Solvent Accessible VOIDS of .         42 Ang**3
PLAT987_ALERT_1_C The Flack x is >> 0 -  Do a BASF/TWIN Refinement     Please Check
```

Alert level G

```
PLAT003_ALERT_2_G Number of Uiso or Uij Restrained non-H Atoms ...          2 Report
PLAT012_ALERT_1_G N.O.K.   _shelx_res_checksum Found in CIF ......     Please Check
PLAT033_ALERT_4_G Flack x Value Deviates > 3.0 * sigma from Zero .      0.054 Note
PLAT072_ALERT_2_G SHELXL First  Parameter in WGHT  Unusually Large       0.15 Report
PLAT083_ALERT_2_G SHELXL Second Parameter in WGHT  Unusually Large       6.74 Why ?
PLAT177_ALERT_4_G The CIF-Embedded .res File Contains DELU Records          1 Report
PLAT178_ALERT_4_G The CIF-Embedded .res File Contains SIMU Records          1 Report
PLAT188_ALERT_3_G A Non-default SIMU Restraint Value has been used     0.0006 Report
PLAT192_ALERT_3_G A Non-default DELU Restraint Value for First Par     0.0009 Report
PLAT192_ALERT_3_G A Non-default DELU Restraint Value for SecondPar     0.0009 Report
PLAT199_ALERT_1_G Reported _cell_measurement_temperature ..... (K)        293 Check
PLAT200_ALERT_1_G Reported   _diffrn_ambient_temperature ..... (K)        293 Check
PLAT343_ALERT_2_G Unusual sp3      Angle Range in Main Residue for         C6 Check
PLAT432_ALERT_2_G Short Inter X...Y Contact  O68      ..C24      .       2.98 Ang.
                                                        x,y,z  =      1_555 Check
PLAT860_ALERT_3_G Number of Least-Squares Restraints ............           7 Note
PLAT933_ALERT_2_G Number of HKL-OMIT Records in Embedded .res File         15 Note
                3 12  0,   0 12  1,   0 24  7,   8  9  7,   1  6  7,  -8  9  7,
                6 12  0,  -1  6  7,   3 12 12,  -5 12  1,   0  3  1,  -3 12 12,
                9 15  0,   0 18  5,   4  3  1,
```

```
   0 ALERT level A = Most likely a serious problem - resolve or explain
   0 ALERT level B = A potentially serious problem, consider carefully
  15 ALERT level C = Check. Ensure it is not caused by an omission or oversight
  16 ALERT level G = General information/check it is not something unexpected

   4 ALERT type 1 CIF construction/syntax error, inconsistent or missing data
  17 ALERT type 2 Indicator that the structure model may be wrong or deficient
   6 ALERT type 3 Indicator that the structure quality may be low
   4 ALERT type 4 Improvement, methodology, query or suggestion
   0 ALERT type 5 Informative message, check
```

It is advisable to attempt to resolve as many as possible of the alerts in all categories. Often the minor alerts point to easily fixed oversights, errors and omissions in your CIF or refinement strategy, so attention to these fine details can be worthwhile. In order to resolve some of the more serious problems it may be necessary to carry out additional measurements or structure refinements. However, the purpose of your study may justify the reported deviations and the more serious of these should normally be commented upon in the discussion or experimental section of a paper or in the "special_details" fields of the CIF. checkCIF was carefully designed to identify outliers and unusual parameters, but every test has its limitations and alerts that are not important in a particular case may appear. Conversely, the absence of alerts does not guarantee there are no aspects of the results needing attention. It is up to the individual to critically assess their own results and, if necessary, seek expert advice.

Publication of your CIF in IUCr journals

A basic structural check has been run on your CIF. These basic checks will be run on all CIFs submitted for publication in IUCr journals (*Acta Crystallographica*, *Journal of Applied Crystallography*, *Journal of Synchrotron Radiation*); however, if you intend to submit to *Acta Crystallographica Section C* or *E* or *IUCrData*, you should make sure that full publication checks are run on the final version of your CIF prior to submission.

Publication of your CIF in other journals

Please refer to the *Notes for Authors* of the relevant journal for any special instructions relating to CIF submission.

PLATON version of 06/01/2024; check.def file version of 05/01/2024

Datablock bj03 - ellipsoid plot

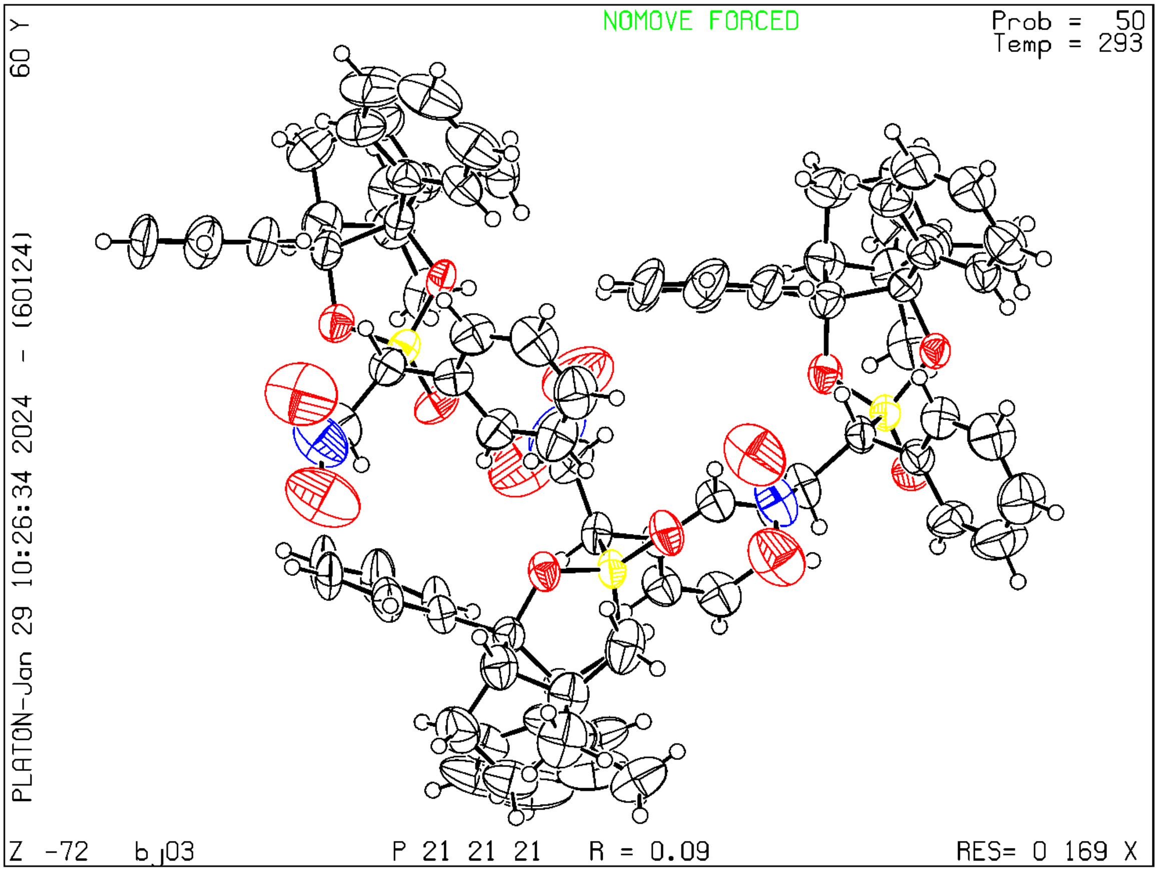

Supplement: Supplementary file 6 — Supplementary Data 4 [file 42004_2025_1735_MOESM6_ESM.zip › Supplementary Data 6-the cif file of 9a/checkcif.pdf]
